# Novel Pyrazino[1,2-*a*]indole-1,3(2*H*,4*H*)-dione Derivatives Targeting the Replication of *Flaviviridae* Viruses: Structural and Mechanistic Insights

**DOI:** 10.3390/v16081238

**Published:** 2024-08-01

**Authors:** Erofili Giannakopoulou, Ifigeneia Akrani, George Mpekoulis, Efseveia Frakolaki, Marios Dimitriou, Vassilios Myrianthopoulos, Niki Vassilaki, Grigoris Zoidis

**Affiliations:** 1 Department of Pharmaceutical Chemistry, Faculty of Pharmacy, School of Health Sciences, National and Kapodistrian University of Athens, Panepistimiopolis-Zografou, GR-15771 Athens, Greece; evgian@pharm.uoa.gr (E.G.); iakrani@pharm.uoa.gr (I.A.); vmyriant@pharm.uoa.gr (V.M.); 2 Molecular Virology Laboratory, Hellenic Pasteur Institute, Vas. Sofias Avenue, GR-11521 Athens, Greece; g.mpekoulis@pasteur.gr (G.M.); mardimitriou7@gmail.com (M.D.)

**Keywords:** antivirals, novel heterocycles, HCV, DENV, YFV, metal chelators, high genetic barrier to resistance, drug design and synthesis, SAR, NMR

## Abstract

Infections with *Flaviviridae* viruses, such as hepatitis C (HCV), dengue (DENV), and yellow fever (YFV) viruses, are major public health problems worldwide. In the case of HCV, treatment is associated with drug resistance and high costs, while there is no clinically approved therapy for DENV and YFV. Consequently, there is still a need for new chemotherapies with alternative modes of action. We have previously identified novel 2-hydroxypyrazino[1,2-*a*]indole-1,3(2*H*,4*H*)-diones as metal-chelating inhibitors targeting HCV RNA replication. Here, by utilizing a structure-based approach, we rationally designed a second series of compounds by introducing various substituents at the indole core structure and at the imidic nitrogen, to improve specificity against the RNA-dependent RNA polymerase (RdRp). The resulting derivatives were evaluated for their potency against HCV genotype 1b, DENV2, and YFV-17D using stable replicon cell lines. The most favorable substitution was nitro at position 6 of the indole ring (compound **36**), conferring EC_50_ 1.6 μM against HCV 1b and 2.57 μΜ against HCV 1a, with a high selectivity index. Compound **52**, carrying the acetohydroxamic acid functionality (-CH_2_CONHOH) on the imidic nitrogen, and compound **78**, the methyl-substituted molecule at the position 4 indolediketopiperazine counterpart, were the most effective against DENV and YFV, respectively. Interestingly, compound **36** had a high genetic barrier to resistance and only one resistance mutation was detected, T181I in NS5B, suggesting that the compound target HCV RdRp is in accordance with our predicted model.

## 1. Introduction

The *Flaviviridae* family includes major human pathogens that are widely distributed. Hepatitis C virus (HCV, Hepacivirus genus) is a main factor of chronic liver disease, leading to liver cirrhosis and hepatocellular carcinoma [1,2]. The mosquito-borne dengue (DENV) and yellow fever (YFV) viruses (Flavivirus genus) cause hemorrhagic fevers that can be life-threatening when left untreated [3,4]. DENV infection leads to visceral and central nervous system diseases [5], while YFV is primarily viscerotropic, causing severe liver damage and jaundice [6]. Other pathogenic flaviviruses are the West Nile virus (WNV), Japanese encephalitis virus (JEV), and tick-borne encephalitis virus (TBEV), which are responsible for a significant number of human infections, accompanied by high mortality and morbidity [7,8]. The *Flaviviridae* genome is a positive-sense single-stranded RNA and encodes a polyprotein precursor, which is processed into structural and non-structural (NS) proteins [9,10,11,12,13]. The NS proteins form a membrane-associated replicase complex (RC) [14].

For HCV, the standard of care until recently was ribavirin and pegylated-interferon alpha (PEG-IFN), which had severe side effects and low sustained virologic response (SVR) rates [15]. Since 2011, direct-acting antivirals (DAAs) targeting HCV NS3A/4A protease, NS5B polymerase, and NS5A phosphoprotein were introduced [16,17,18], which attain more than 90% SVR [19]. However, there is a risk for the development of drug resistance [20], while the high cost limits the widespread clinical use of DAAs [21]. Thus, there is still a need for novel drugs. In the case of DENV and YFV, there is no approved antiviral therapy [22,23].

The HCV NS5B is an RNA-dependent RNA polymerase (RdRp), while Flavivirus NS5, apart from the RdRp domain, also bears a *N*-terminal methyltransferase (MTase) domain. RdRp synthesizes negative-strand RNA intermediates and positive-strand progeny RNA genomes [14,24,25]. In the crystal structure of RdRp, three domains known as palm, fingers, and thumb domains can be discerned. The polymerase catalytic activity depends on the presence of two divalent metal ions (Mg^2+^ or Mn^2+^) in the active site [26].

Thus, metal-chelating pharmacophores targeting the *Flaviviridae* RdRp could be a successful and widely effective therapeutic strategy. Moreover, a number of *N*-hydroxyimides with a metal-chelating functional group have been reported as potent inhibitors of other viral genome-editing enzymes [27,28], such as HBV ribonuclease H [29,30,31,32], HIV ribonuclease H (RNH) [33], HIV integrase (IN) [34], influenza A and B endonuclease [35], or Rift Valley fever virus viral nuclease [36].

Our previous data have shown that by incorporating a 2,6-diketopiperazine moiety into the pharmacophore indole ring, potent inhibitors against HCV RNA replication, as well as influenza PA endonuclease activity, were obtained [37]. Based on these results and SAR analysis, we designed a new series of *N*-hydroxyimide and *N*-acetohydroxamic acid analogues with different substitutions introduced at the indole core structure that were evaluated against HCV, DENV, and YFV RNA replication in reporter replicon assays. The most promising compound was further characterized for its resistance mutation profile and mechanism of action.

## 2. Materials and Methods

### 2.1. Cells and Viral Constructs

The subgenomic replicon stable cell lines, Huh5-2 (genotype 1b; Con1) [38] and Huh7-JFH1 (genotype 2a, JFH1) [39], have been described previously. The subgenomic reporter replicons of HCV 3a (S52) S52-SG(Feo)(AII) [40], HCV 4a (ED43) ED43-SG(Feo)(VYG) [40], DENV2 16681 pD2-hRUPac [41], and YFV-17D pYF-hRUPac [42] have been described previously and were used to construct the stable cell lines Huh7.5-3a [43], Huh7.5-4a [43], Huh7-D2 [44], and Huh7-YF, respectively. Plasmids pH77S.3/GLuc2A [45] and pHCV-N.2/GLuc2A [46] encode the full-length HCV reporter genome sequences of genotypes 1a (H77) and 1b (HCV-N), respectively.

The cultivation of all cell lines stably expressing the subgenomic viral replicons as well as the replication permissive Huh7-Lunet cells [47], was performed in high glucose (25 mM) Dulbecco’s modified minimal essential medium (Invitrogen, Waltham, MA, USA), containing 10% (*v*/*v*) fetal calf serum, 2 mM L-glutamine, 100 U/mL penicillin, 100 µg/mL streptomycin, and 0.1 mM non-essential amino acids (referred to as complete DMEM). The following concentrations of geneticin (G418) were added to the culture medium in order to maintain the stable expression of the HCV viral replicons: 500 μg/mL for Huh5.2 cells, 1 mg/mL for Huh7-JFH1, 750 μg/mL for Huh7.5-3a, and 350 μg/mL for Huh7.5-4a. On the other hand, for the stable cell lines Huh7-D2 and Huh7-YF, 0.5 μg/mL puromycin was added into the medium.

### 2.2. In Vitro Transcription

Full-length viral constructs were linearized with XbaI and subsequently used in in vitro transcription reactions as described previously [48].

### 2.3. Transfection with In Vitro Transcribed RNA

Transfection of full-length viral RNAs into cultured cells was performed using electroporation, as described elsewhere [49]. Briefly, 4 × 10^6^ cells were trypsinized and resuspended in Cytomix solution [50] supplemented with 2 mM ATP and 5 mM glutathione. Cells suspension in Cytomix was mixed with 10 μg of viral RNA and electroporated with a Gene Pulser system (Bio-Rad, Hercules, CA, USA). Cells were immediately transferred to 20 mL of complete DMEM and subsequently seeded as required for the assay.

### 2.4. Cell-Based Antiviral and Cytotoxicity Assays

Assays were performed in the stable replicon cell lines or cells transiently transfected with full-length viral RNA. Serial dilutions of the test compounds, or the solvent DMSO, were added for 72 h. More specifically, for the cytotoxicity experiments, we used 3-fold serial concentrations from 7 μΜ to 200 μΜ, while for the antiviral assays, 2-fold serial concentrations ranging from 0.8 μΜ to 200 μΜ were used. Antiviral potency and cytotoxicity of the compounds was determined by measuring virus-expressed luciferase activity or intracellular ATP levels, respectively. The half maximal effective concentration (EC_50_) values of the compounds, defined as the ones reducing luciferase signal by 50%, as well as the compound concentration causing 50% cell death (CC_50_), were determined using a nonlinear regression analysis using the Prism 9.0 software (GraphPad Software Inc., San Diego, CA, USA). For each compound, the ratio of the evaluated CC_50_ to EC_50_ values (referred to as the selectivity index: SI) was calculated. Compounds with higher SI values are theoretically safer and more effective during in vivo treatment.

### 2.5. Chemicals

Pegylated interferon a2b was obtained from Merck (Merck & Co., Inc., Rahway, NJ, USA). Daclatasvir was kindly provided by Dr. Marc Windisch (Institute Pasteur Korea).

### 2.6. Luciferase and Bradford Assays

Firefly luciferase (F-Luc) activity was measured in cell lysates using the Luciferase Assay System (Promega Corporation, Madison, WI, USA), according to the manufacturer’s instructions. The activity of Gaussia luciferase (G-Luc) was quantified in cell supernatants by the use of coelenterazine in a 12 μM concentration (Promega Corporation, Madison, WI, USA) diluted in assay buffer consisted of 500 mM NaCl, 50 mM potassium phosphate, 1 mM EDTA, pH 7.4. A GloMax 20/20 single tube luminometer (Promega Corporation, Madison, WI, USA) was used to perform all measurements for 10 s. Total protein levels as determined by the Bradford assay (Bio-Rad, Hercules, CA, USA) were utilized for normalization.

### 2.7. Measurement of Intracellular ATP Levels

The ViaLight HS BioAssay kit (Lonza, Basel, Switzerland) was used to determine the levels of endogenous ATP according to the manufacturer’s instructions. The measurements were performed for 1 s in a GloMax 20/20 single-tube luminometer (Promega Corporation, Madison, WI, USA). Total protein amounts were determined using the Bradford assay and used for normalization of the results.

### 2.8. Indirect Immunofluorescence

The HCV Con1 NS5A was analyzed using an indirect immunofluorescence analysis with the HCV NS5A (9E10) monoclonal antibody (kindly provided by Prof. C. Rice) at a dilution of 1:1000, as described elsewhere [49]. The bound primary antibody was detected by the use of goat anti-mouse antibody which was conjugated to Alexa-Fluor 488, at a dilution of 1:1000. Propidium iodide (Sigma-Aldrich, St. Louis, MO, USA) was used to stain DNA, which in turn was used for the determination of the total number of cells. All images were captured using the Leica TCS-SP5II Two-photon Confocal Microscope (Thermo Fisher Scientific, Waltham, MA, USA), which was equipped with a Spectra Physics Mai Tai infrared laser source. For each condition, 10 separate images were captured at ×20 magnification and processed using the image analysis software Fiji (Version 2.9.0).

### 2.9. Gel Electrophoresis and Western Blot Analysis

Denaturing SDS-polyacrylamide gel electrophoresis and Western blotting was performed as described elsewhere [51]. Dilutions of 1:2000 for the HCV NS5A (9E10) monoclonal antibody, and 1:6000 for the β-actin monoclonal antibody (Merck-Millipore, Rahway, NJ, USA), respectively, were used. A dilution of 1:2000 for the secondary anti-mouse horseradish peroxidase-conjugated antibody (Cell Signaling, Danvers, MA, USA) was used. Imaging quantification was performed by using Quantity I software (Bio-Rad, Hercules, CA, USA).

### 2.10. Total RNA Extraction and Quantification of Viral Replicons

Total RNA was extracted from Huh5-2 cells using TRIzol reagent (Thermo Fisher Scientific, Waltham, MA, USA), according to the manufacturer’s instructions. Replicon RNA was quantified with reverse-transcription (RT) and quantitative real-time polymerase chain reaction (qPCR). RT was performed using Moloney Murine Leukemia Virus (MMLV) reverse transcriptase (Promega) and the reverse primers for Con1 IRES (Con1-IRES-R: 5′-GGATTCGTGCTCATGGTGCA-3′) and the housekeeping gene YWHAZ (YWHAZ-R: 5′-GGATGTGTTGGTTGCATTTCCT-3′). For qPCR, the Con1 IRES specific primers Con1-IRES-F (5′-GGCCTTGTGGTACTGCCTGATA-3′) and Con1-IRES-R and KAPA SYBR FAST qPCR Master Mix (Sigma-Aldrich, St. Louis, MO, USA) were used. YWHAZ was employed as an internal control (primers YWHAZ-F: 5′-GCTGGTGATGACAAGAAAGG-3′ and YWHAZ-R).

### 2.11. Selection of Drug-Resistance Mutations

Huh5-2 replicon cells were treated with compound **36** at gradually increasing multiple concentrations of the EC_50_ value (1-2-5-10-25 × EC_50_: 1.61–40.25 μΜ) in the presence of 500 μg/mL G418 for 30 passages. Cells treated with DMSO were used as negative control. The resulting cell populations were used for the antiviral assay to quantify compound **36** activity or lysed for RNA isolation and subsequent NGS analysis.

### 2.12. Next Generation Sequencing

Total RNA extracted from replicon cells that were treated with compound **36** at 25×EC_50_ or DMSO was used to perform Reverse transcription, PCR amplification, and next generation sequencing (NGS) of HCV RNA, as described previously [43]. In brief, the reverse transcription reactions were carried out by the use of the MMLV reverse transcriptase (Promega) and the specific reverse primer A9413 (5′-CAGGATGGCCTATTGGCCTGGAG-3′). LA taq polymerase (Takara, San Jose, CA, USA) and specific primers [35,52], were employed for the amplification of the sequence encoding the Con1 HCV protein (NS3-NS5B). The PCR amplification was conducted in four 1.5–2 kb sized segments with overlapping regions, followed by the agarose gel-electrophoresis separation of the derived PCR products. The appropriately sized products were subjected to purification by the use of the commercially available NucleoSpin Gel and PCR Clean-up kits (Macherey-Nagel, Düren, Germany), according to the manufacturer’s instructions. Next, the purified PCR products from each sample were quantified and pooled together at equimolar ratios. The Ion Xpress™ Plus Fragment Library Kit (Life Technologies, Carlsbad, CA, USA) and Ion Xpress™ Barcode Adapters Kits (Life Technologies) were used for the preparation of the cDNA libraries, which were then sequenced using the Ion PGM™ System (Life Technologies). Finally, the derived data were processed by the Ion PGM™ System software (Life Technologies, Carlsbad, CA, USA) in order to identify the presence of any drug resistance mutations.

### 2.13. Eradication of HCV RNA

Huh5.2 cells were cultured in the presence of increasing concentrations of compound **36** (1×-2×-5×-10×-25×EC_50_) or DMSO for 30 passages (cell adaptation). HCV RNA was successfully eradicated from DMSO- and compound **36**-adapted cells after serial passaging in the presence of daclatasvir at a concentration of 10 nM for 7 days. The efficiency of the eradication was confirmed by the quantitation of the replication-derived renilla luciferase activity and the viral RNA levels via RT-qPCR.

### 2.14. Construction of Viral Plasmids Containing NS5B Mutant T181I

Amino acid and nucleotide numbers refer to the Con1 (GenBank accession no. AJ238799) or HCV-N (GenBank accession no. AF139594) isolates. The nucleotide substitution generating the NS5B mutant T181I was C8140T in Con1 (codon 181 ACC) and was introduced into pHCV-N.2/GLuc at the corresponding position of HCV-N (C8155T) by PCR-based site-directed mutagenesis. Overlapping PCR fragments were generated by using the template pHCV-N.2/GLuc2A and combining each oligonucleotide primer carrying the T181I substitution, mutT181I-F (5′-GTGGTCTCCA**T**CCTTCCTCAAGCCG-3′) or mutT181I-R (5′-CGGCTTGAGGAAGG**A**TGGAGACCAC-3′), with the corresponding outer primer S7697-HCV-N (5′-CTCTTTGCTGCGCCATCACAAC-3′) or A10472 (5′-CTGTTGGGAAGGGCGATCGGTGCG-3′). DNA fragments were combined by PCR using the outer primers S7697-HCV-N and A10472 and, after restriction with AflII and XbaI, were inserted into pHCV-N.2/GLuc2A. The resulting mutated clones were validated by nucleotide sequencing.

### 2.15. Statistical Analysis

**Figure 1:**
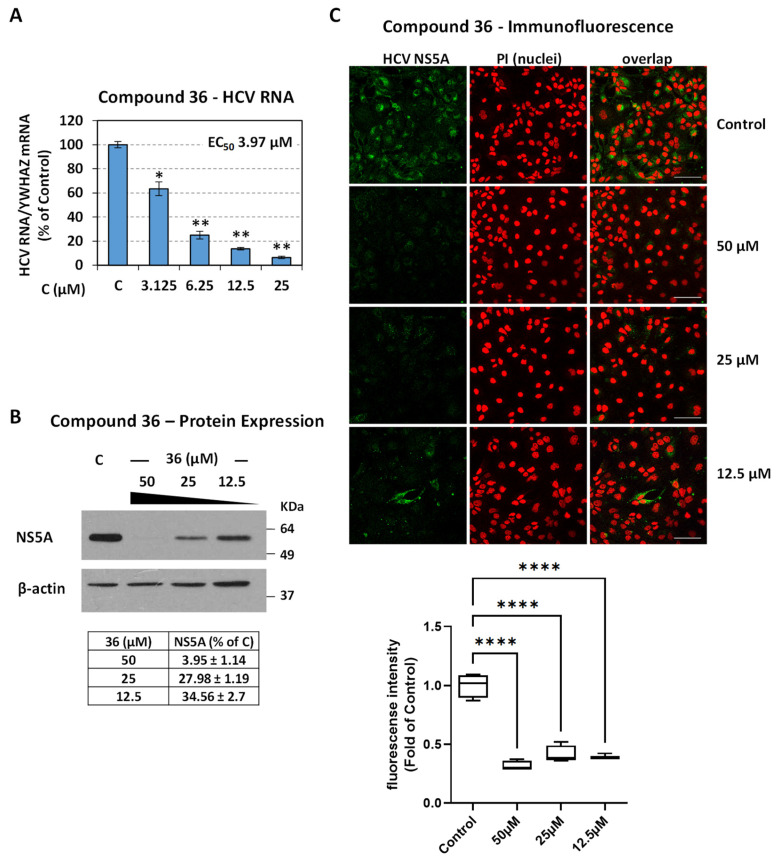
Inhibition profile of compound **36** at the level of HCV RNA and protein in a subgenomic Con1 replicon assay. Huh5-2 cells treated with serial dilutions of 36 or the solvent DMSO (control—C). (**A**) Quantification of (+) strand HCV RNA by RT-qPCR. Values are expressed as percentage of those obtained from control cells. mRNA levels of the housekeeping gene 14-3-3-zeta polypeptide (YWHAZ) were used for normalization. Bars represent mean values obtained from three separate experiments in triplicate. Error bars stand for standard deviation (SD). Statistical analysis was performed using One Way ANOVA, followed the appropriate post hoc analysis for multiple comparisons. * *p* < 0.001, ** *p* < 0.0001 vs. control. (**B**) Western blot analysis for NS5A. β-actin was used as loading control. (**C**) Indirect immunofluorescence for NS5A (left panels). Nuclei were stained with propidium iodide (PI; middle panels) as a cell viability control. Merged images are shown on the right. Bar, 100 μm. Quantification of the captured images was performed using Fiji software (Version 2.9.0) and statistical analysis of the results was carried out using One-Way ANOVA, followed by the appropriate post hoc analysis. **** *p* < 0.0001 vs. control.

**Figure 2:**
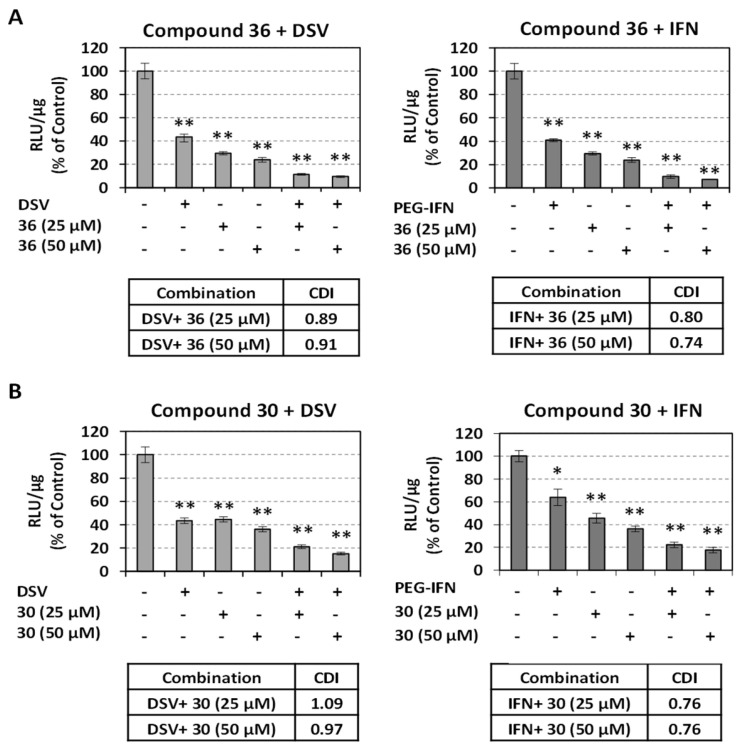
Combinatory activity of compounds **36** (**A**) or **30** (**B**) with DSV (left) or Peg-IFN (right) against HCV replication. Huh5-2 cells were treated or not with 10 IU/mL Peg-IFN or 10 pM DSV in the presence or absence of the analogues. F-Luc activity was determined and calculated as relative light units (RLU) per μg of total protein. Values were expressed as percentage of those obtained with cells treated with the solvent DMSO (control—C). CDI: coefficient of drug interaction. Bars represent mean values obtained from three separate experiments in triplicate. Error bars represent standard deviation (SD). Statistical analysis in all panels was carried out using One Way ANOVA, followed by Bonferroni correction. * *p* < 0.001, ** *p* < 0.0001 vs. control.

**Figure 3:**
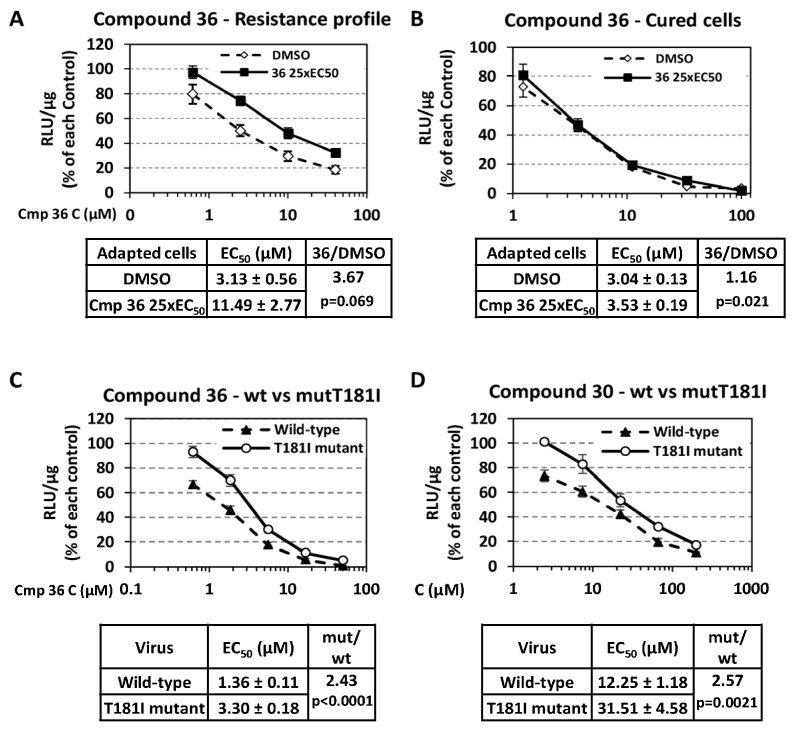
(**A**) Resistance profile of **36** in HCV GT 1b (Con1). Huh5-2 replicon cells, adapted in the presence of increasing concentrations of **36** (1×-2×-5×-10×-25×EC_50_ of the EC_50_) or the solvent DMSO for 30 culture passages, were subsequently treated with serial dilutions of **36** for 72 h. Top: F-Luc activity was quantified and normalized to total protein. For each population, values are expressed as percentage of those obtained from cells treated with DMSO (control) for 72 h. Bottom: comparison in EC_50_ values of **36** between cells adapted with **36** (25 × EC_50_) and those with DMSO. *p* = 0.069 vs. DMSO-adapted cells. (**B**) Anti-HCV activity of **36** in “cured” Huh5-2 cells. The **36**- and DMSO-adapted cell populations were cured from viral replicon after treatment with daclatasvir (10 nΜ) for two weeks. Then, cells were electroporated with wild-type HCV GT 1b full-length RNA (HCV-N.2-GLuc) and subsequently treated with serial dilutions of **36** for 72 h. Top: Gaussia luciferase (G-Luc) activity was quantified and normalized to total protein. For each population, values are expressed as percentage of those obtained from cells treated with DMSO (control) for 72 h. Bottom: comparison in EC_50_ values of **36** between cured cells previously adapted with **36** (25 × EC_50_) and those with DMSO. *p* = 0.021 vs. DMSO-adapted cells. (**C**,**D**) Comparison of the activity of analogue **36** (**C**) and **30** (**D**) against the wild-type HCV-N.2-GLuc and T181I mutated variant. Huh7-Lunet cells were electroporated with the HCV-N.2-GLuc/T181I mutated RNA (mutT181I) or the respective wild-type (wt) and subsequently treated with serial dilutions of **36** or **30** for 72 h. Top: Gaussia luciferase (G-Luc) activity was quantified and normalized to total protein. For each viral construct, values are expressed as percentage of those obtained from cells treated with DMSO (control) for 72 h. Bottom: comparison of EC_50_ values between the wild-type and mutated viruses. (**C**) *p* < 0.0001 vs. cells infected with wild-type virus. (**D**) *p* = 0.0021 vs. cells infected with wild-type virus. In all panels, the differences detected in the EC_50_ values of compound 36 in each separate panel were statistically compared using un-paired *t*-test.

Results are presented as bar graphs. Bars correspond to mean values from at least two independent experiments in triplicate; error bars stand for standard deviation values. The statistical analysis of the presented results in Figures 1 and 2 was performed using One Way ANOVA, followed by the appropriate post hoc analysis for multiple comparisons. In Figure 3, statistical comparisons of the evaluated EC_50_ values in each panel were carried out using the unpaired *t*-test. *p* values ≤ 0.05 were considered statistically significant.

### 2.16. In Silico Studies

The crystal structure of HCV RNA polymerase (PDB ID: 1GX6) was prepared and minimized using the Protein Preparation Wizard tool within Maestro (Schrödinger 2021-2, LLC, New York, NY, USA) [53]. The inhibitor **36** was prepared for docking using the LigPrep tool (Schrödinger 2021-2, LLC, New York, NY, USA) [54]. Induced Fit Docking (IFD) calculations were performed with the standard protocol of the IFD tool (Schrödinger 2021-2, LLC, New York, NY, USA). Initially, Glide docking calculations were conducted, utilizing a more flexible Van der Waals radii scaling, resulting in a default maximum of 20 poses per ligand. Subsequently, side-chain prediction and minimization were carried out for each protein–ligand complex using Prime. Finally, the redocking of every complex energetically close to the lowest-energy structure was implemented using Glide, and the IFDScore (estimation of binding energy) was calculated. MMGBSA ΔG_bind_ was calculated using the Prime MM-GBSA tool (Schrödinger 2021-2, LLC, New York, NY, USA) [54]. The simulation was conducted without constraints regarding the protein flexibility. MD simulations were carried out using Desmond Molecular Dynamics System 2020-1 (D. E. Shaw Research, New York, NY, USA) [55]. Initially, the receptor–ligand complexes were prepared using the System Builder tool in Desmond Molecular Dynamics System 2020-1 (D. E. Shaw Research, New York, NY, USA). TIP3P water molecules were chosen as a solvent model, and the system was embedded in a triclinic-shaped box, with OPLS_2005 assigned as the force field. Salt was added at a concentration of 0.15 M, and the negative charges were neutralized by the addition of Na^+^ ions. Subsequently, different MD simulations of 250 ns and 1000 ns were performed using an NPT ensemble. Temperature and pressure were maintained at 300 Kelvin and 1.01325 bar after applying the Nose–Hoover chain thermostat and Martyna–Tobias–Klein barostat, respectively. ADME property prediction was conducted using the SwissADME (http://www.swissadme.ch, accessed on 10 March 2024) with default parameters.

## 3. Chemistry

**Scheme 1:**
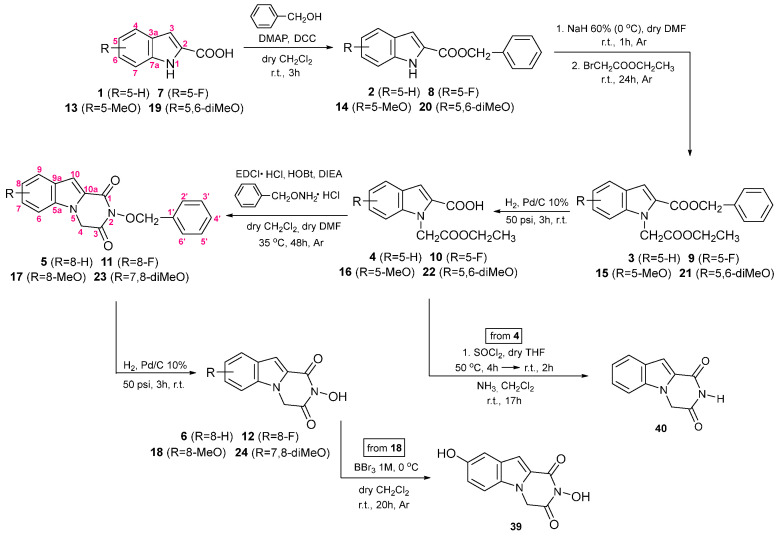
Synthesis of the target 1,2-annulated indole heterocycles **6**, **12**, **18**, **24**, **39** and **40**.

Carboxylic acids **4**, **10**, **16**, and **22** were the key intermediates to prepare the new target compounds (as depicted in Scheme 1), and were prepared from the respective, commercially available, 5-substituted-*1H*-indole-2-carboxylic acids. The latter compounds were esterified with benzyl alcohol using 4-(dimethylamino)pyridine (DMAP) and *N*,*N′*-dicyclohexylcarbodiimide (DCC) in dichloromethane [37,56]. Benzyl indole-2-carboxylates were converted to the corresponding diesters (**3**, **9**, **15** and **21**) by reacting with ethylbromoacetate in the presence of sodium hydride in DMF. The deprotection of the diesters using hydrogenolysis (Pd/C 10%) yielded the key intermediate 5-substituted-1-(2-ethoxy-2-oxoethyl)-1*H*-indole-2-carboxylic acids (**4**, **10**, **16**, and **22**), in almost quantitative yield. The latter were coupled with *O*-benzyl hydroxylamine in the presence of EDCI∙HCl and HOBt in CH_2_Cl_2_/DMF to give the corresponding *O*-benzyl hydroxamates, which were intramolecularly cyclized, in one pot, in the presence of diisopropylethylamine (excess), to the corresponding diketopiperazine analogues **5**, **11**, **17**, and **23** in high yields (59–80%, two steps). Finally, catalytic debenzylation (Pd/C 10%) gave the respective hydroxyimide analogues **6**, **12**, **18**, and **24** in almost quantitative yield. The target compound **39** (Scheme 1) was synthesized by the demethylation of the methylarylether **18** using boron tribromide.

The amidation of acid ester **4** (via the respective chloride) was carried out using thionyl chloride and gaseous NH_3_. The intermediate amide underwent intramolecular cyclization in one pot, as illustrated in Scheme 1, leading to the formation of pyrazino[1,2-*a*]indole-1,3(2*H*,4*H*)-dione **26** with an excellent overall yield.

**Scheme 2:**
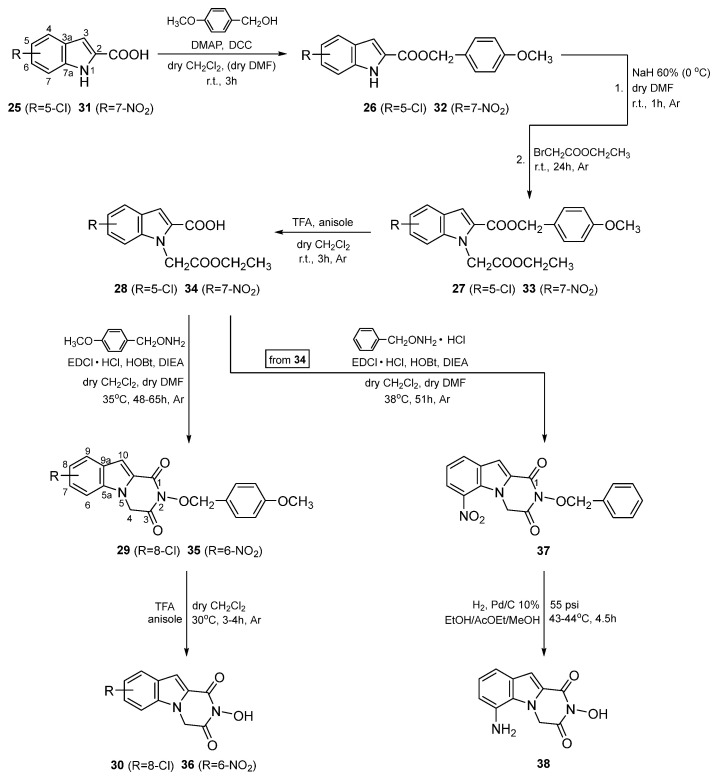
Synthesis of the target 1,2-annulated indole heterocycles **30**, **36**, and **38**.

In the case of the chloro- and nitro-indole derivatives, the synthetic route was modified to avoid the dechlorination of the aromatic ring and correspondingly the reduction of the nitro group during the catalytic hydrogenation (Scheme 2). The esterification of indole-2-carboxylic acids **25** and **31** was therefore carried out with 4-methoxybenzyl alcohol and the resulting esters were treated with ethyl bromoacetate to afford the corresponding diesters **27** and **33**. Deprotection was achieved using trifluoroacetic acid (TFA) in the presence of anisole (methoxybenzene) as a scavenger [57] in anhydrous dichloromethane. *N*-substituted indole-2-carboxylic acids **28** and **34** were coupled with O-(4-methoxybenzyl)-hydroxylamine in the presence of EDCI·HCl, HOBt, and DIEA and led to the corresponding O-4-methoxybenzyl hydroxamates which were intramolecularly cyclized in the presence of diisopropylethylamine (excess), in one pot, to the corresponding diketopiperazine analogues **29** and **35** in high yields (59–80%, two steps). The deprotection of the *N*-[(4-methoxybenzyl)oxy]imides **29** and **35** in the presence of TFA led to the desired chloro- and nitro-substituted *N*-hydroxy-imides **30** and **36**. The 7-nitro-substituted indole-2-carboxylic acid **34** was also treated with O-benzylhydroxylamine and was then catalytically hydrogenated, aiming for its deprotection and the simultaneous reduction of the nitro group to yield the desired amino derivative **38**.

**Scheme 3:**
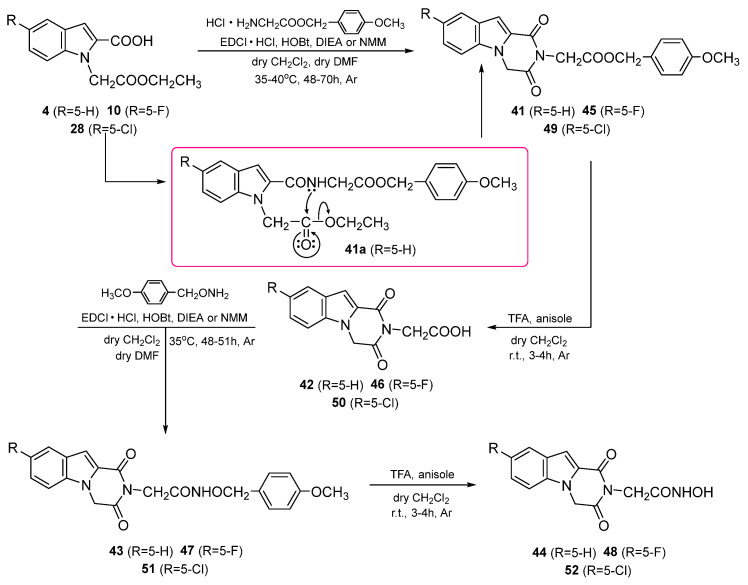
Synthesis of the target 1,2-annulated indole heterocyclic *N*-acetohydroxamic acids **44**, **48**, and **52**.

As shown in the Scheme 3, *N*-substituted carboxylic acids **4**, **10**, and **28** are coupled with 4-methoxybenzyl glycine hydrochloride in the presence of EDCI·HCl, HOBt, and DIEA or *N*-methylmorpholine (NMM) as a base, and after intramolecular cyclization, they afford the 4-methoxy benzyl esters **41**, **45**, and **49**. The treatment of the latter with trifluoroacetic acid, in the presence of anisole in anhydrous dichloromethane, leads to the corresponding condensed indole-diketopiperazine carboxylic acidsm which are converted to acetamides **43**, **47**, and **51** by the reaction with 4-methoxybenzylhydroxylamine, as described before. In the last step, they are deprotected with TFA and anisole to give the desired *N*-acetohydroxamic acids **44**, **48**, and **52**.

The choice of 4-methoxybenzyl glycine and the corresponding hydroxylamine in the case of the chloro-substituted indole is obvious. The preparation of the unsubstituted analogue was initially attempted using benzylglycine but problems in the deprotection step (catalytic hydrogenolysis) indicated the modification of the synthetic route and thus deprotection in both steps was carried out with TFA.

The synthesis of the indole-diketopiperazine 4-methoxybenzyl esters **41**, **45**, and **49** again takes place via the formation and intramolecular cyclization of the intermediate amido-esters according to the mechanism illustrated in Scheme 3. In the case of the unsubstituted derivative, it was possible to isolate and identify intermediate **41a**, and its ^1^H NMR spectrum is depicted in the SI part.

**Scheme 4:**
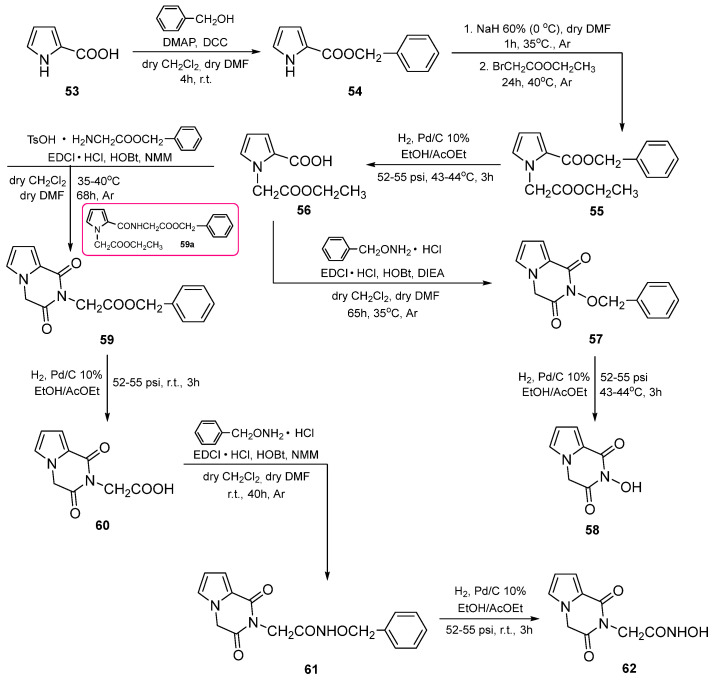
Synthesis of the target 1,2-annulated pyrrole heterocyclic analogues **58** and **62**.

As illustrated in Scheme 4, the common key precursor for the preparation of the two pyrrole analogs **58** and **62** is *N*-substituted-pyrrole-2-carboxylic acid **56**. The synthetic route followed for its preparation is based on the synthesis of carboxylic acids **4**, **10**, **16**, and **22**, which is shown in Scheme 1. Shortly, in the first step, a Steglich esterification with benzyl alcohol is carried out, followed by a nucleophilic nitrogen substitution in the presence of NaH and ethyl bromoacetate. Diester **55** undergoes catalytic hydrogenation using Pd/C, yielding the carboxylic acid intermediate **56**. The coupling of acid **56** with O-benzylhydroxylamine, followed by the intramolecular cyclization of the amide, leads to *N*-benzyloxy substituted dione **57**, which is hydrogenated to afford 2-hydroxypyrrolo[1,2-a]pyrazine-1,3(2H,4H)-dione **58**. Alternatively, the treatment of the carboxylic acid intermediate **56** with tosylate O-benzyl glycine leads to the formation of ester **59**, which is debenzylated by catalytic hydrogenation. The resulting carboxylic acid **60**, after coupling with O-benzyl-hydroxylamine hydrochloride, yielded the corresponding acetamide **61**, which in the last step is hydrogenated, whereupon the target *N*-acetohydroxamic acid **62** was isolated in a quantitative yield. The amido-ester intermediate **59a** formed prior to intramolecular cyclization to afford benzyl ester **59** was also isolated in this case and its ^1^H NMR spectrum is depicted in the SI part.

**Scheme 5:**
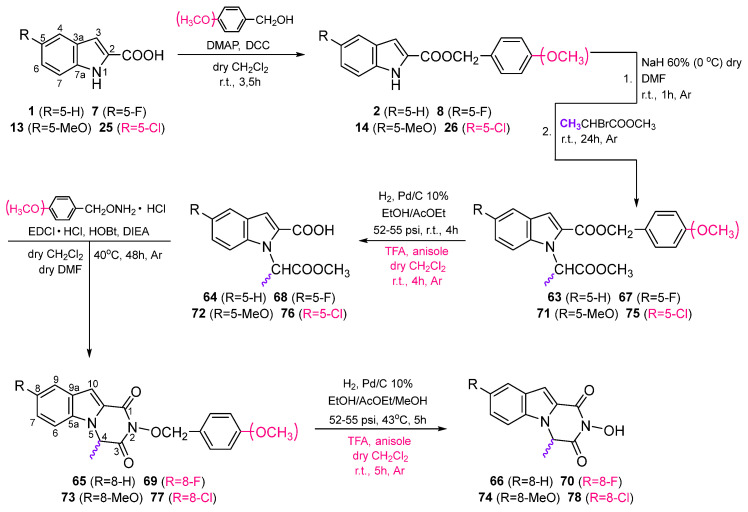
Synthesis of the target 1,2-annulated indole heterocycles **66**, **70**, **74** and **78**.

As depicted in the Scheme 5, the esterification of indole-2-carboxylic acids **1**, **7**, **13**, and **25** was carried out with benzyl alcohol or 4-methoxybenzyl alcohol in the presence of DMAP and DCC in dry CH_2_Cl_2_ and the resulting esters were treated with sodium hydride and then methyl 2-bromopropionate to afford the corresponding diesters **63**, **67**, **71**, and **75**. Deprotection was achieved either by hydrogenolysis or with trifluoroacetic acid (TFA) in the presence of anisole (methoxybenzene) as a scavenger [57] in anhydrous dichloromethane, and yielded the corresponding carboxylic acids **64**, **68**, **72**, and **76**. The treatment of the latter with benzylhydroxylamine or 4-methoxybenzylhydroxylamine, as described before, led to the corresponding benzyl hydroxamates or O-4-methoxybenzyl hydroxamates. which were intramolecularly cyclized in the presence of diisopropylethylamine (excess), in one pot, to the corresponding diketopiperazine analogues **65**, **69**, **73**, and **77** in good yields. In the last step, they were deprotected by catalytic hydrogenolysis or with TFA and anisole to give the desired *N*-hydroxyimides **66**, **70**, **74**, and **78**.

## 4. Results

### 4.1. Biological Evaluation

#### 4.1.1. Screening of Compounds in HCV Genotype 1b Replicon System

Our previously reported results regarding the antiviral activity of indole–flutimide heterocyclic derivatives (1,2-annulated indolediketopiperazines) [37], which were rationally designed to target HCV RdRp, prompted us to evaluate the anti-*Flaviviridae* activity of a new series of compounds bearing different substitutions on the core scaffold.

First, the effect of the newly synthesized compounds on HCV RNA replication and on cell viability was determined in Huh5-2 cells. This cell line harbors a HCV genotype 1b (strain Con1) subgenomic replicon that expresses firefly luciferase (F-Luc) as a marker of viral RNA replication [38]. In brief, Huh5.2 cells were seeded at a 40% confluency, and 24 h later were treated with serial dilutions of the tested compounds, followed by further incubation for 72 h at 37 °C and 5% CO_2_. The half maximal effective concentration (EC_50_) and the median cytotoxic concentration (CC_50_) were determined by measuring the HCV replication-derived luciferase activity and intracellular ATP levels, respectively (Table 1), followed by a dose–response curve analysis. Selectivity indices (SI) = CC_50_/EC_50_ were also calculated.

Our group has already highlighted the significance of the hydroxyl substituent on the imidic nitrogen, which participates in the metal-chelating moiety of the compounds for their potency against HCV replication [37]. Here, to identify the structural features of the privileged pyrazino[1,2-*a*]indole-1,3(2*H*,4*H*)-dione scaffold required for potent antiviral activity, we increased the flexibility of the chelating moiety by replacing this hydroxyl group with the carboxylic (-CH_2_COOH) or acetohydroxamic acid (-CH_2_CONHOH) moieties in analogues **6**, **12**, and **30**, which concomitantly carry hydrogen, fluorine, and chlorine substitutions at position 8 of the indole ring, respectively. These modifications were detrimental for the antiviral activity of these compounds, as observed by comparing their EC_50_ values to those estimated for their hydroxyl counterparts. More specifically, we compared compounds **6** (-OH) to **44** (-CH_2_CONHOH), **12** (-OH) to **46** (-CH_2_COOH) and **48** (-CH_2_CONHOH), and finally **30** (-OH) to **52** (-CH_2_CONHOH).

As these modifications increase the size of the derivatives, this could account for the loss of activity. To verify the latter, we replaced the indole ring with the smaller pyrrole ring. However, the resulting compounds **58** and **62**, bearing hydroxyl and acetoxydroxamic acid (-CH_2_CONHOH) substitutions on the imidic nitrogen, respectively, were inactive against HCV.

Based on these results, we retained the hydroxyl group on the imidic nitrogen and inserted further substitutions in the core structure. In an attempt to increase the lipophilicity of the compounds, we introduced a methyl group at position 4 of the diketopiperazine ring. Comparing the activity of the methyl-substituted derivatives **66**, **74**, **70**, and **78** with the respective non-substituted counterparts **6**, **18**, **12**, and **30**, we observed that this modification caused an increase in anti-HCV activity for most analogues, except for the chloro-substituted one, where the methyl-substitution had no significant effect. A slight increase in cytotoxicity was concomitantly detected.

Next, we introduced substituents in other positions of the indole ring. First, a second methoxy group was inserted on compound **18** at position 7 to obtain **24**; however, this did not improve the antiviral potency. Moreover, we synthesized compounds **38** and **36**, bearing two different nitrogen-containing groups at position 6 of the core scaffold, amino and nitro, respectively. Interestingly, derivative **36** exhibited high anti-HCV activity, with an EC_50_ value of 1.61 μΜ, which, combined with low cytotoxicity (CC_50_ 175.4 μΜ), resulted in the favorable SI of 108.9.

As compared to the chloro-substituted molecule **30**, which we previously reported as the most promising indolediketopiperazine derivative [37], the nitro-substituted **36** showed a significantly higher potency of about 6-fold. Thus, **36** was selected for further characterization.

#### 4.1.2. Comparing the Anti-HCV Intergenotypic Activity of Compounds **36** to **30**

The activity of the most active compound, **36** (nitro-substituted), was further characterized in other HCV genotypes and compared to that of **30** (chloro-substituted), which was previously reported as the most active against HCV 1b [37]. Specifically, we determined the effect on viral replication-driven luciferase activity in Huh7-JFH1, Huh7.5-3a, and Huh7.5-4a stable cell lines containing subgenomic replicons of HCV GT 2a (strain JFH1), GT 3a (strain S52), and GT 4a (strain ED43), respectively. In addition, the activity of the compounds was determined in Huh7-Lunet cells electroporated with the in vitro transcribed full-length reporter viral RNA of HCV genotype 1a (strain H77S.3).

Similar to the results obtained for GT 1b (EC_50_ 1.61 μΜ), compound **36** was also very effective against HCV GT 1a (EC_50_ 2.57 μΜ), while 30 showed similar activity (Table 2). In the case of GTs 2a and 3a, derivative **36** showed moderate activity in contrast to **30**, which was inactive. Neither compound showed significant potency against GT 4a. In conclusion, the nitro substitution at position 8 seems to be more favorable for antiviral potency than chloro substitution at position 6.

#### 4.1.3. Screening of Compounds against DENV and YFV Replicon Systems

Taking into account that the pyrazino[1,2-*a*]indole-1,3(2*H*,4*H*)-dione derivatives have been designed to target *Flaviviridae* RdRp, which is well conserved among HCV and *Flaviviruses* [24,26], we sought to determine their activity against DENV and YFV RNA replication as well. Specifically, we determined viral replication-derived luciferase activity in Huh7-D2 and Huh7-YF stable cell lines containing the subgenomic replicon of DENV serotype 2 (strain 16681) or YFV (strain 17D).

In the case of YFV, among the previously reported derivatives, only **6**, carrying a hydrogen substitution at position 8 of the indole ring, was active (EC_50_ 57.55 μM, Table 3). Interestingly, a ~2-fold higher potency was exerted by the corresponding compound **66**, bearing a methyl substitution at position 4 of the diketopiperazine ring. The other methyl-substituted molecules (compounds **66**, **70**, **74**, and **78**) were also effective, with the 8-chloro-substituted **78** showing the highest potency (EC_50_ 6.57 μM) and selectivity (SI 15.22). Interestingly, compound **78** was also active against HCV genotype 1b at a low micromolar range. However, the most promising compound against HCV (compound **36**) exhibited a not significant efficacy against YFV and DENV viruses. For DENV replication, in contrast to HCV and YFV, only **52**, carrying the acetohydroxamic group (-CH_2_CONHOH) on the imidic nitrogen, and the amino-substituted compound **38** at position 6, showed moderate activity.

### 4.2. Further Characterization and Mechanism of Action Studies

#### 4.2.1. Validation of Compound Activity with Additional Assays

The inhibition profile of analogue **36** in Huh5-2 cells, as measured using the luciferase assay, was confirmed by determining HCV RNA and NS5A protein levels using reverse transcription-quantitative polymerase chain reaction (RT-qPCR) or indirect immunofluorescence and Western blot analysis, respectively. We observed that **36** reduced HCV RNA replication (Figure 1A), with the EC_50_ value similar to that calculated on the basis of virus-derived luciferase activity (Table 1). A consistent reduction was observed in the levels of NS5A, as depicted in the Figure 1B,C panels.

#### 4.2.2. Drug–Drug Interaction Studies of Pyrazino[1,2-*a*]indole-1,3(2*H*,4*H*)-dione Derivatives with Approved HCV Inhibitors

Next, we investigated the effect of a combinatory treatment of compound **36** or **30** with the approved HCV treatment pegylated interferon a2b (Peg-IFN), or daclatasvir (DSV), in Huh5-2 replicon cells. As shown in Figure 2, **36** and DSV had a synergistic effect, which was determined by calculating the coefficient of drug interaction (CDI ≈ 0.9), while **30** and DSV had an additive effect (CDI ≈ 1). Interestingly, both compounds had a more pronounced synergistic effect when combined with Peg-IFN (CDI ≈ 0.75). The coefficient of the drug interaction in the conducted experimental procedures was determined based on the following formula: CDI = AB/(A × B), where AB corresponds to the ratio of luciferase activity levels, which is indicative of the viral replication evaluated in cells treated in combination with the two studied drugs. Cells treated with DMSO were used for the normalization of the derived results. A or B stands for the effect of the single drug treatment on viral replication, normalized to the control group.

#### 4.2.3. Compound **36** Shows a High Barrier to Resistance and Targets RdRp Polymerase

To verify the possible mechanism of action for compound **36** on viral RNA replication and to determine its resistance profile, we cultured Huh5-2 replicon cells for 30 passages in the presence of the selection antibiotic, G418, and increasing concentrations of **36** up to 25-fold of EC_50_ (1×-2×-5×-10×-25×EC_50_), or DMSO as control. Next, in both **36**- and DMSO-adapted cell populations, the efficacy of the compound was determined by measuring the luciferase activity. Interestingly, the activity of **36** was maintained after 15 weeks of treatment, as only a ~3-fold reduction was observed (Figure 3A). This indicates that this derivative has a high genetic barrier to resistance in GT 1b. As the high barrier of resistance is characteristic of host-targeting inhibitors [44,58], we investigated whether the resistance profile of **36** is due to a cellular adaptation. For this, the viral RNA was eradicated from **36**- and DMSO-adapted cell populations. Cells were subsequently transfected with the wild-type HCV GT 1b full-length RNA (HCV-N.2-GLuc) and then the activity of **36** was determined. As shown in Figure 3B, the EC_50_ of **36** is quite similar in both cell populations, which excludes the implication of cellular factor(s) in the target mechanism.

Next, we analyzed the resistance mutations selected in the GT 1b Con1 replicon after 15 weeks of treatment with compound 36, by using RT-PCR and subsequent next generation sequencing (NGS). Interestingly, only one non-synonymous amino acid substitution, T181I, located in NS5B, was detected in the viral RNA of 36-adapted cells, but not in DMSO-adapted cells. To validate that T181I is responsible for the resistance profile, we introduced the mutation to the wild-type HCV GT 1b full-length RNA (HCV-N.2-GLuc). After transfection in Huh7-Lunet cells, the HCV-N.2-GLuc/T181I mutated virus was 2.43-fold less susceptible to 36 than the wild-type one. Interestingly, T181I also reduced virus susceptibility to 30 by 2.57-fold, suggesting a common target for the two compounds. T181I is not a known resistance-associated substitution (RAS) [59] and is conserved only in genotype 1b. T181I is located close to the active site of NS5B RdRp, which is in accordance with the predicted mode of action for 36 (Figure 3).

### 4.3. In Silico Studies

A hepatitis C Virus RNA polymerase crystallographic structure, in complex with Uridine 5′-Triphosphate (UTP) (PDB ID: 1GX6), was selected for in silico calculations [60]. A T181I mutant was created and prepared through a 250 ns molecular dynamics simulation (Desmond Schrodinger). Subsequently, the **36** inhibitor was docked in both the wild-type and the mutant proteins utilizing Induced Fit Docking software (Schrodinger Inc., New York, NY, USA). MM-GBSA energy properties were obtained for every receptor–ligand complex. MD simulations of 1 μs were performed for the lowest energy wild-type and mutant polymerase–**36** complexes. Finally, ADMET properties were predicted using SwissADME (http://www.swissadme.ch/) and IFD studies of the entire set of compounds to the wild-type polymerase were conducted.

**Figure 4:**
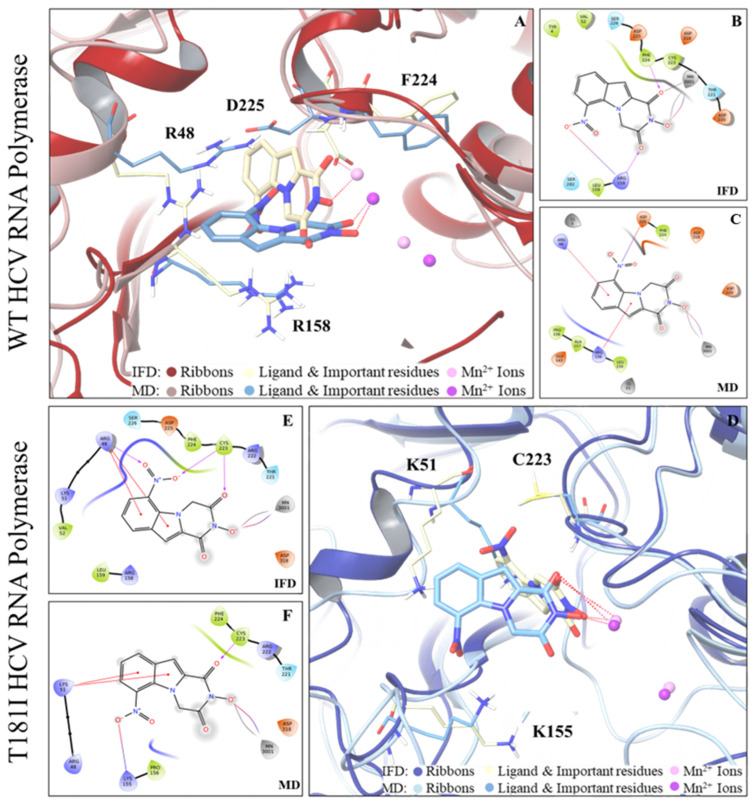
Predicted binding mode of **36** in both the wild-type and the T181I mutant HCV RNA polymerase produced by the IFD calculation and the molecular dynamics simulations. In the visual representation of the IFD results, the ligands and important residues are depicted in yellow, while the ribbons are shown in a dark red and dark blue color. The two Mn^2+^ ions are highlighted in light purple. Conversely, the MD simulation results are illustrated with light blue ligands and amino acids, lighter red and blue ribbons, and dark purple Mn^2+^ ions. The interactions facilitating the chelation effect of the ligand are shown as red dash lines. (**A**) Three-dimensional diagrams illustrating IFD and MD results for the wild-type protein. (**B**) Two-dimensional representation of the wild-type protein generated by IFD. (**C**) 2-Dimensional representation of the wild-type protein generated by MD. (**D**) Three-dimensional diagrams presenting IFD and MD results for the T181I mutant protein. (**E**) Two-dimensional representation of the T181I mutant protein produced by IFD. (**F**) Two-dimensional representation of the T181I mutant protein generated by MD.

The Induced Fit Docking (IFD) results, combined with MM-GBSA dG Bind Energy values, support the role of **36** as a chelator, with the ionized hydroxyl group of the diketopiperazine analogue positioned opposite to the manganese ion located in the Thr221, Asp220, Asp318 pocket in both the wild-type and mutated protein (Figure 4). The molecular dynamics (MD) simulation confirms the stability of the ionized hydroxyl group’s position, as the distance between the Mn^2+^ ion maintains a stable orientation, and the O^−^ ion remains below 2.15 Å (wild-type) and 2.30 Å (mutant) throughout the 1 μs duration (Supplementary—Figure S3). The IFD results indicate that the nitro group of **36** and the C1 carbonyl oxygen form hydrogen bonds with the wild-type polymerase through the Arg158 side chain and Phe224, respectively (Figure 4B). Conversely, the mutant RNA polymerase interacts with the same compound by forming hydrogen bonds between Cys223 and both the nitro substitute and C3 carbonyl oxygen, along with a Pi–cation interaction between the indole rings and Arg48 (Figure 4E). During the molecular dynamics calculations, the binding geometry of **36** undergoes changes in both protein structures. In the wild-type polymerase, a flip in **36** occurs, with the nitro group oriented near Arg48, forming a salt bridge with Asp225, and the indole rings developing Pi–cation interactions with both Arg48 and Arg158 (Figure 4C). In the mutated protein, the ring system flips, and the nitro group shifts from the Lys51, Cys223, Arg48 pocket to the Lys155, Pro156, Arg48 pocket, and remains stable for the rest of the simulation (Figure 4F).

Based on the presented data, the binding geometry of **36** as well as the rest of the active compounds to the hepatitis C Virus RNA polymerase is in agreement with the previously described model [37] (Supplementary Figure S1). ADMET property prediction indicates favorable pharmacokinetics for the active compounds (Supplementary Table S1). The molecular dynamics simulation results suggest that the T181I mutation could induce a different binding mode of compound **36**, potentially leading to slight variations to binding affinity, which possibly explains the EC_50_ value variation. Further investigation into the significance of specific mutations is needed to fully elucidate the impact of protein dynamics on inhibitor binding regarding the enzyme.

## 5. Discussion and Conclusions

In the present study, we further modified our previously reported 1,2-annulated indolediketopiperazines [37], in order to improve their selectivity against the *Flaviviridae* RdRp polymerase. The activity and mechanism of action studies for the novel compounds were performed using viral replicon assays.

First, we increased the flexibility of the chelating moiety of the compounds, by replacing the hydroxyl group in the imidic nitrogen with either a carboxylic or acetohydroxamic acid. The observed minimal anti-HCV efficacy of the modified analogues highlighted the pivotal function of the hydroxyl substituent on the imidic nitrogen. Prior to rejecting the aforementioned alterations, we verified that the observed poor antiviral efficacy of these compounds was not due to their increased size, by replacing the indole ring with a smaller pyrrole one. Moreover, in the analogues bearing -OH on the imidic nitrogen, the elevation of the lipophilicity (addition of a methyl substituent at position 4) resulted in an enhancement of their antiviral activity against HCV genotypes 1b and 3a, accompanied by a slight increase in their cytotoxicity. The introduction of additional substituents into the indole ring led to the discovery of the most promising compound in terms of antiviral potency and selectivity. Specifically, this was compound **36**, bearing a nitro substitution at position 6 of the indole ring. The concomitant presence of an amino substitution at position 6 and of an acetohydroxamic acid group (-CH_2_CONHOH) on the imidic nitrogen conferred moderate activity against DENV. In the case of YFV, the methyl substitution at position 4 of the diketopiperazine ring (-OH substituent on imidic nitrogen) was favorable for compound efficacy, especially when combined with the electron-withdrawing group’s chlorine and fluorine at position 8.

Over the years, a number of effective treatments against HCV have been developed and approved [13,14,15,16,17,18,19]. It is noteworthy that our two most effective compounds (**30** and **36**), when used in combination with peg-IFN or daclatasvir, exhibited significant synergistic activity, leading to diminished viral propagation. Thus, these compounds could serve as promising candidates for combinatorial anti-HCV therapies.

Finally, the adaptation of the HCV GT 1b replicon cell line to compound **36** indicated a high genetic barrier to resistance. The developed resistance was not retained after the eradication of the replicon from the adapted cell line and the re-insertion of the wild-type full-length viral sequence, which suggests that there is no implication of a cellular factor. Instead, we identified the presence of the resistance mutation T181I near the active site of the NS5B polymerase. This, in combination with the crucial role of the metal-chelating moiety for the antiviral activity and the in silico data, confirm the predicted mode of action that involves the chelation of the Mg^2+^/Mn^2+^ in the catalytic pocket. In total, the new series of pyrazino[1,2-*a*]indole-1,3(2*H*,4*H*)-dione derivatives appear to be active against *Flaviviridae* family members; however, there are differences in specificity for each virus, which possibly reflect differences in the respective RdRp structures. Finally, the selectivity indices and the favorable drug-like properties of some of the tested compounds suggest that the novel scaffold of an appropriately substituted pyrazino[1,2-*a*]indole-1,3(2*H*,4*H*)-dione ring combined with a metal-binding moiety could be a successful strategy for producing multifunctional drugs and may be the key to producing therapeutics against a wide range of pathogens.

A limitation of the current study is that the activity of the compounds was not evaluated against full-length viruses, except in the case of HCV GT 1a. Moreover, a more precise determination of the drug synergism could be performed by testing a broad range of different concentrations of the compounds and the use of more appropriate synergy tools.

## Figures and Tables

**Table 1:** EC_50_, CC_50_, and SI values of pyrazino[1,2-*a*]indole-1,3(2*H*,4*H*)-dione derivatives in HCV GT 1b (Con1) replicon cells.

Compound	Structure	EC_50_ ^a^ (μΜ)	CC_50_ ^b^ (μΜ)	SI ^c^
**6**	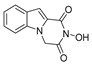	126.70 ± 8.98	>200	>1.6
**12**	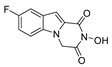	80.98 ± 8.24	>200	>2.5
**18**	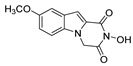	>200	>200	-
**24**	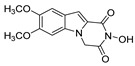	>200	>200	-
**25**	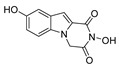	>200	>200	-
**26**	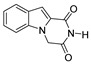	>200	>200	-
**30**	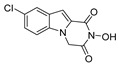	9.9 ± 1.75	>200	>20.2
**36**	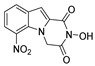	1.61 ± 0.32	175.4 ± 13.45	108.9
**38**	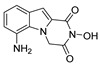	>200	>200	-
**44**	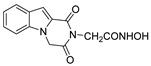	>200	>200	-
**46**	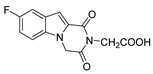	>200	>200	-
**48**	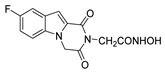	>200	>200	-
**52**	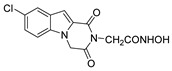	>200	>200	-
**58**	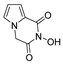	>200	>200	-
**62**	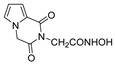	>200	>200	-
**66**	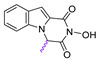	99.68 ± 5.52	177.3 ± 5.71	1.8
**70**	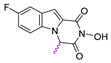	32.87 ± 1.93	86.45 ± 3.36	2.6
**74**	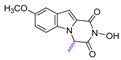	61.15 ± 1.72	100.6 ± 4.05	1.6
**78**	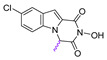	12.86 ± 0.39	99.91 ± 3.54	7.8

^a^ half maximal effective concentration (EC_50_) expressed as mean ± standard deviation values; ^b^ compound concentration causing 50% cell death presented as mean ± standard deviation values; ^c^ selectivity index determined using the CC_50_/EC_50_ formula.

**Table 2:** EC_50_ of compounds **30** and **36** against HCV GT 1a (H77S.3), 2a (JFH1), 3a (S52), and 4a (ED43) replication.

Compound	Structure	EC_50_ ^a^ (μM)			
		GT 1a	GT 2a	GT 3a	GT 4a
**30**	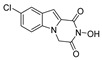	2.79 ± 0.48	>100	>100	84.73 ± 5.28
**36**	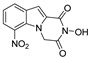	2.57 ± 0.34	78.31 ± 3.44	27.86 ± 1.64	>100

^a^ half maximal effective concentration (EC_50_) expressed as mean ± standard deviation values.

**Table 3:** EC_50_, CC_50_, and SI values of compounds against DENV and YFV replication.

Compound	Structure	CC_50_ ^a^ (μΜ)	YFV		DENV	
			EC_50_ ^b^ (μΜ)	SI ^c^	EC_50_ (μΜ)	SI
**6**	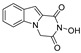	>200	57.55 ± 2.64	>3.48	>200	-
**12**	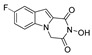	>200	>200	-	>200	-
**18**	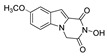	>200	>200	-	>200	-
**24**	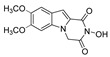	>200	>200	-	>200	-
**39**	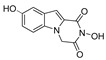	>200	>200	-	>200	-
**40**	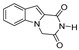	>200	>200	-	>200	-
**30**	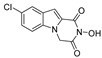	>200	>200	-	>200	-
**36**	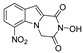	175.4 ± 13.45	143.9 ± 4.90	1.22	124.3 ± 6.49	1.41
**38**	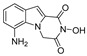	>200	>200	-	75.24 ± 6.58	>2.66
**44**	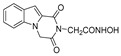	>200	>200	-	>200	-
**46**	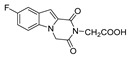	>200	>200	-	>200	-
**48**	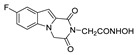	>200	>200	-	>200	-
**52**	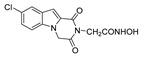	>200	>200	-	61.3 ± 3.10	>3.26
**58**	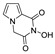	>200	>200	-	131.2 ± 10.09	>1.52
**62**	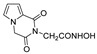	>200	>200	-	>200	-
**66**	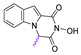	177.3 ± 5.71	30.33 ± 2.10	5.85	>100	-
**70**	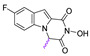	86.45 ± 3.36	11.67 ± 0.66	7.41	>50	-
**74**	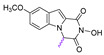	100.6 ± 4.05	20.87 ± 1.28	4.82	>50	-
**78**	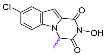	99.91 ± 3.54	6.57 ± 0.33	15.22	>50	-

^a^ compound concentration causing 50% cell death presented as mean ± standard deviation values; ^b^ half maximal effective concentration (EC_50_) expressed as mean ± standard deviation values; ^c^ selectivity index determined using the CC_50_/EC_50_ formula.

## Data Availability

All relevant data are within the manuscript.

## Supplementary Materials

The following supporting information can be downloaded at: https://www.mdpi.com/article/10.3390/v16081238/s1. I. Experimental methods; II. Preparation procedures and characterization data of compounds; III. In silico studies—calculation methods—predicted drug-likeness properties and ADME; Figure S1: The experimental binding mode of the active compounds based on the lowest energy IFD result; Figure S2: RMSD diagrams of both the wild-type and the T181I mutant HCV RNA polymerase in complex with **36**; Figure S3: Distance between the Mn^2+^ ion and the O^−^ ion of both the wild-type and the T181I mutant HCV RNA polymerase; Table S1: ADMET property prediction for the active compounds. Calculations were performed with the SwissADME tool and Copies of NMR spectra of all synthesized compounds are supplied as supporting information.
