# Supplementary material for: Novel Pyrazino[1,2-a]indole-1,3(2H,4H)-dione Derivatives Targeting the Replication of Flaviviridae Viruses: Structural and Mechanistic Insights
Source: Viruses. 2024 Aug 1;16(8):1238. doi: 10.3390/v16081238 (PMC11360281; doi:10.3390/v16081238)
Supplement: Supplementary file 1 [file viruses-16-01238-s001.zip › viruses-3043194-supplementary.pdf]

Supporting Information

Research Article

Novel pyrazino[1,2-a]indole-1,3(2*H*,4*H*)-dione derivatives targeting the replication of *Flaviviridae* viruses: Structural and mechanistic insights

Erofili Giannakopoulou [1], Ifigeneia Akrani [1,&], George Mpekoulis [2,&], Efseveia Frakolaki [2,‡], Marios Dimitriou [2], Vassilios Myrianthopoulos [1], Niki Vassilaki [2,#,*] and Grigoris Zoidis [1,#,*]

1 School of Health Sciences, Faculty of Pharmacy, Department of Pharmaceutical Chemistry, National and Kapodistrian University of Athens, Panepistimiopolis-Zografou, GR-15771 Athens, Greece; evgian@pharm.uoa.gr (E.G.); iakrani@pharm.uoa.gr (I.A.); vmyriant@pharm.uoa.gr (V.M.)

2 Molecular Virology Laboratory, Hellenic Pasteur Institute, Vas. Sofias Avenue, GR-11521, Athens, Greece; g.mpekoulis@pasteur.gr (G.M.); niki.vassilaki@runbox.com (E.F.); mardimitriou7@gmail.com (M.D.)

* Correspondence: Prof. Grigoris Zoidis (zoidis@pharm.uoa.gr), Tel.: +302107274809; Dr. Niki Vassilaki (nikiv@pasteur.gr)

& Both authors contributed equally to this work.

# Both authors contributed equally to this work.

‡ Deceased.

List of Contents

I. Experimental methods 4

II. Preparation procedures and characterization data of compounds 5

III. *In-silico* studies - Calculations Methods - Predicted drug-likeness properties and ADME 29

Figure S1: The experimental binding mode of the active compounds based on the lowest energy IFD result 29

Figure S2: RMSD diagrams of both the Wild type and the T181I mutant HCV RNA polymerase in complex with **36** 31

Figure S3: Distance between the Mn^{2+} ion and the O^{-} ion of both the Wild type and the T181I mutant HCV RNA polymerase 32

Figure S4: Position of mutation T181I in the HCV RNA polymerase structure 32

Table S1: ADMET properties prediction for the active compounds. Calculations were performed with the SwissADME tool 33

IV. Copies of NMR spectra 36

NMR spectra of compound **2** (^{1}H, ^{13}C, DEPT, COSY, HSQC, HMBC) 36

NMR spectra of compound **8** (^{1}H, ^{13}C, DEPT, COSY, HSQC, HMBC) 42

NMR spectra of compound **14** (^{1}H, ^{13}C, COSY, HSQC, HMBC) 48

NMR spectra of compound **20** (^{1}H, ^{13}C, DEPT, COSY, HSQC, HMBC) 53

NMR spectra of compound **26** (^{1}H, ^{13}C, DEPT, COSY, HSQC, HMBC) 59

NMR spectra of compound **32** (^{1}H, ^{13}C, COSY, HSQC, HMBC) 65

NMR spectra of compound **3** (^{1}H, ^{13}C, COSY, HSQC) 71

NMR spectra of compound **9** (^{1}H, ^{13}C, DEPT, COSY, HSQC, HMBC) 75

NMR spectra of compound **15** (^{1}H, ^{13}C, COSY, HSQC, HMBC) 81

NMR spectra of compound **21** (^{1}H, ^{13}C, DEPT, COSY, HSQC, HMBC) 86

NMR spectra of compound **27** (^{1}H, ^{13}C, COSY, HSQC, HMBC) 92
NMR spectra of compound **33** (^{1}H, ^{13}C, DEPT, COSY, HSQC, HMBC) 97
NMR spectra of compound **4** (^{1}H, ^{13}C, DEPT, COSY, HSQC, HMBC) 103
NMR spectra of compound **10** (^{1}H, ^{13}C, DEPT, COSY, HSQC, HMBC) 109
NMR spectra of compound **16** (^{1}H, ^{13}C, DEPT, COSY, HSQC, HMBC) 115
NMR spectra of compound **22** (^{1}H, ^{13}C, COSY, HSQC-DEPT, HMBC) 121
NMR spectra of compound **28** (^{1}H, ^{13}C, COSY, HSQC, HMBC) 127
NMR spectra of compound **34** (^{1}H, ^{13}C, DEPT, COSY, HSQC, HMBC) 132
NMR spectra of compound **5** (^{1}H, ^{13}C, COSY, HSQC-DEPT, HMBC) 138
NMR spectra of compound **11** (^{1}H, ^{13}C, COSY, HSQC-DEPT, HMBC) 143
NMR spectra of compound **17** (^{1}H, ^{13}C, DEPT, COSY, HSQC, HMBC) 149
NMR spectra of compound **23** (^{1}H, ^{13}C, DEPT, COSY, HSQC, HMBC) 155
NMR spectra of compound **23a** (^{1}H, ^{13}C, DEPT, COSY, HSQC, HMBC) 161
NMR spectra of compound **29** (^{1}H, ^{13}C, COSY, HSQC, HMBC) 167
NMR spectra of compound **35** (^{1}H, ^{13}C, DEPT, COSY, HSQC, HMBC) 171
NMR spectra of compound **6** (^{1}H, ^{13}C, DEPT, COSY, HSQC, HMBC) 177
NMR spectra of compound **12** (^{1}H, ^{13}C, DEPT, COSY, HSQC-DEPT, HMBC) 183
NMR spectra of compound **18** (^{1}H, ^{13}C, DEPT, COSY, HSQC) 189
NMR spectra of compound **24** (^{1}H, ^{13}C, DEPT, COSY, HSQC, HMBC) 194
NMR spectra of compound **40** (^{1}H, ^{13}C, COSY, HSQC) 200
NMR spectra of compound **30** (^{1}H, ^{13}C, COSY, HSQC) 204
NMR spectra of compound **36** (^{1}H, ^{13}C, DEPT, COSY, HSQC-DEPT, HMBC) 208
NMR spectra of compound **38** (^{1}H, ^{13}C, DEPT, COSY, HSQC, HMBC) 214
NMR spectra of compound **41** (^{1}H, ^{13}C, DEPT, COSY, HSQC, HMBC) 220

NMR spectra of compound **41a** (^{1}H, ^{13}C, DEPT, COSY, HSQC-DEPT, HMBC) 226
NMR spectra of compound **45** (^{1}H, ^{13}C, DEPT, COSY, HSQC) 232
NMR spectra of compound **49** (^{1}H, ^{13}C, DEPT, COSY, HSQC, HMBC) 236
NMR spectra of compound **42** (^{1}H, ^{13}C, DEPT, COSY, HSQC-DEPT, HMBC) 242
NMR spectra of compound **46** (^{1}H, ^{13}C, DEPT, COSY, HSQC, HMBC) 248
NMR spectra of compound **50** (^{1}H, ^{13}C, DEPT, COSY, HSQC, HMBC) 254
NMR spectra of compound **43** (^{1}H, ^{13}C, DEPT, COSY, HSQC-DEPT, HMBC) 260
NMR spectra of compound **16** (^{1}H, ^{13}C, DEPT, COSY, HSQC-DEPT, HMBC) 266
NMR spectra of compound **51** (^{1}H, ^{13}C, COSY, HSQC, HMBC) 272
NMR spectra of compound **44** (^{1}H, ^{13}C, DEPT, COSY, HSQC- DEPT, HMBC) 277
NMR spectra of compound **48** (^{1}H, ^{13}C, DEPT, COSY, HSQC-DEPT, HMBC) 283
NMR spectra of compound **52** (^{1}H, ^{13}C, COSY, HSQC- DEPT, HMBC) 289
NMR spectra of compound **54** (^{1}H, ^{13}C, DEPT, COSY, HSQC- DEPT, HMBC) 295
NMR spectra of compound **55** (^{1}H, ^{13}C, DEPT, COSY, HSQC- DEPT, HMBC) 301
NMR spectra of compound **56** (^{1}H, ^{13}C, DEPT, COSY, HSQC-DEPT, HMBC) 307
NMR spectra of compound **57** (^{1}H, ^{13}C, COSY, HSQC- DEPT, HMBC) 313
NMR spectra of compound **58** (^{1}H, ^{13}C, DEPT, COSY, HSQC- DEPT, HMBC) 318
NMR spectra of compound **59** (^{1}H, ^{13}C, DEPT, COSY, HSQC- DEPT, HMBC) 324
NMR spectra of compound **59a** (^{1}H, ^{13}C, DEPT, COSY, HSQC- DEPT, HMBC) 330
NMR spectra of compound **60** (^{1}H, ^{13}C, DEPT, COSY, HSQC-DEPT, HMBC) 336
NMR spectra of compound **61** (^{1}H, ^{13}C, DEPT, COSY, HSQC-DEPT, HMBC) 342
NMR spectra of compound **62** (^{1}H, ^{13}C, DEPT, HSQC- DEPT, HMBC) 348
V. References 353

I. Experimental methods

During the conduct of the experimental part of the present study were used the following materials, apparatuses and techniques. Melting points were determined using a Büchi capillary apparatus and are uncorrected. NMR experiments were performed to elucidate the structure and determine the purity of the newly synthesized compounds. ^{1}H NMR and 2D NMR spectra (COSY, HSQC-DEPT, HMBC) were recorded on a Bruker DRX400 spectrometer (400.13 MHz, ^{1}H NMR) and a Bruker Ultrashield™ Plus Avance III 600 spectrometer (600.11 MHz, ^{1}H NMR). ^{13}C NMR spectra were recorded on a Bruker Avance 200 spectrometer (50.32 MHz, ^{13}C NMR), a Bruker DRX400 spectrometer (100.61 MHz, ^{13}C NMR) and a Bruker Ultrashield™ Plus Avance III 600 spectrometer (150.9 MHz, ^{13}C NMR). Chemical shifts δ *(delta)* are reported in parts per million (ppm) downfield from the NMR solvent, with the tetramethylsilane or solvent (DMSO-d_6) as internal standard. Data processing including Fourier transformation, baseline correction, phasing, peak peaking and integrations were performed using MestReNova software v.12.0.0. Splitting patterns are designated as follows: s, singlet; d, doublet; t, triplet; q, quartet; dd, doublet of doublets; td, triplet of doublets; tt, triplet of triplets; tq, triplet of quartets; ddt, doublet of doublets of triplets; m, multiplet; complex m, complex multiplet. Coupling constants (*J*) are expressed in units of Hertz (Hz). The spectra were recorded at 293 K (20 °C) unless otherwise specified. The solvent used to obtain the spectra was deuterated DMSO, DMSO-d_6 (quin, 2.50 ppm, ^{1}H NMR; septet, 39.52 ppm, ^{13}C NMR). Analytical thin-layer chromatography (TLC) was used to monitor the progress of the reactions, as well as to authenticate the compounds. TLCs were conducted on, precoated with normal-phase silica gel, aluminium sheets (Silica gel 60 F_{254}, Merck) (layer thickness 0.2 mm), precoated with reverse phase silica gel, aluminium sheets (Silica gel 60 RP-18 F_{254}s, Merck) and precoated aluminum oxide plates (TLC Aluminium oxide 60 F_{254}, neutral). Developed plates were examined under a UV light source, at wavelengths of 254 nm, or after being stained by iodine vapors. The Retention factor (R_f) of the newly synthesized compounds, that equals to the distance migrated over the total distance covered by the solvent, was also measured on the chromatoplates. Elemental analyses (C, H, N) were performed by the Service Central de Microanalyse at CNRS (France), and were within ±0.4% of the theoretical values. Elemental analysis results for the tested compounds correspond to >95% purity. The commercial reagents were purchased from Alfa Aesar, Sigma-Aldrich, and Merck, and were used without further purification. Solvent abbreviations: ACN, acetonitrile; AcOEt, ethyl acetate; Et_2O, diethyl ether; EtOH, ethanol; MeOH, methanol.

II. Preparation procedures and characterization data of compounds

General procedure for the preparation of benzylesters 2, 8, 14, 20, 26 and 32 (Steglich esterification)
A solution of (5-substituted-) 1*H*-indole-2-carboxylic acid (10 mmol), benzyl alcohol (12.5 mmol), and DMAP (2.0 mmol) in 65 mL of dichloromethane was treated with DCC (10.0 mmol) and stirred at room temperature for 3 h. The resulting mixture was filtered, concentrated in vacuo, taken up in 90 mL of ethyl acetate, and filtered. The solution was subsequently washed sequentially with 1 N HCl (2x6 mL), H_2O (2x12 mL), saturated aqueous solution of $NaHCO_3$ (2x15 mL), and brine (2x15 mL), dried over Na_2SO_4, and concentrated. The residue was purified by flash column chromatography on silica gel.

Benzyl 1*H*-indole-2-carboxylate (2)
It was prepared by reacting 1*H*-indole-2-carboxylic acid (**1**) with benzyl alcohol, following the general esterification procedure. Column chromatography on silica gel, using CH_2Cl_2 as an eluent, gave **2** (87% yield) as a pale yellow crystalline solid, of which the characteristics are consistent with the literature.(Kempf and Condon, 1990)

Benzyl 5-fluoro-1*H*-indole-2-carboxylate (8)
It was prepared by reacting 5-fluoro-1*H*-indole-2-carboxylic acid (**13**) with benzyl alcohol, following the general esterification procedure. Column chromatography on silica gel, using a mixture of eluents *n*-hexane/AcOEt (4:1), gave **14** (70% yield) as a white crystalline solid; mp 151-153 °C (AcOEt/*n*-pentane), R_f = 0.46 (*n*-hexane/AcOEt 4:1).
^{1}H NMR (400 MHz, $CDCl_3$) δ (ppm) 5.41 (s, 2H, COOCH_2Ph), 7.09 (td, 1H, J_1=9.1 Hz, J_2=2.4 Hz, H_6), 7.24 (d, 1H, J=1.9 Hz, H_3), 7.28-7.35 (m, 2H, H_4, H_7), 7.35-7.51 (complex m, 5H, $H_{2'}$, $H_{3'}$, $H_{4'}$, $H_{5'}$, $H_{6'}$), 9.18 (brs, 1H, N*H*); ^{13}C NMR (50 MHz, $CDCl_3$) δ (ppm) 66.8 (COO*C*H_2Ph), 106.5, 107.0 (d, J_{C-F}=23.3 Hz, C_4), 108.9, 109.0 (d, J_{C-F}= 5.5 Hz, C_3), 112.7, 112.9 (d, J_{C-F}=9.6 Hz, C_7), 114.4, 114.9 (d, J_{C-F}=27.0 Hz, C_6), 127.5, 127.7 (d, J_{C-F}=10.5 Hz, C_{3a}), 128.3 ($C_{2'}$, $C_{6'}$), 128.5 ($C_{4'}$), 128.7 ($C_{3'}$, $C_{5'}$), 131.7 (C_2), 133.5 (C_{7a}), 135.6 ($C_{1'}$), 155.8, 160.5 (d, J_{C-F}=236.8 Hz, C_5), 161.6 (*C*OOCH_2Ph). Anal. Calcd for $C_{16}H_{12}FNO_2$: C, 71.37; H, 4.49; N, 5.20. Found: C, 71.48; H, 4.57; N, 5.34.

Benzyl 5-methoxy-1*H*-indole-2-carboxylate (14)
It was prepared by reacting 5-methoxy-1*H*-indole-2-carboxylic acid (**7**) with benzyl alcohol, following the general esterification procedure. Column chromatography on silica gel, using a mixture of eluents *n*-hexane/AcOEt (4:1), gave **8** (69% yield) as a pale yellow crystalline solid; mp 140-142 °C (AcOEt/Et_2O, *n*-pentane), R_f = 0.45 (*n*-hexane/AcOEt 4:1).
^{1}H NMR (400 MHz, $CDCl_3$) δ (ppm) 3.76 (s, 3H, OCH_3), 5.31 (s, 2H, COOCH_2Ph), 6.91 (dd, 1H, J_1=8.9 Hz, J_2=2.4 Hz, H_6), 6.98 (d, 1H, J=2.2 Hz, H_4), 7.12 (d, 1H, J=2.1 Hz, H_3), 7.20 (dd, 1H, J_1=8.9 Hz, J_2=0.5 Hz, H_7), 7.24-7.40 (complex m, 5H, $H_{2'}$, $H_{3'}$, $H_{4'}$, $H_{5'}$, $H_{6'}$), 8.96 (brs, 1H, N*H*); ^{13}C NMR (50 MHz, $CDCl_3$) δ (ppm) 55.6 (O*C*H_3), 66.6 (COO*C*H_2Ph), 102.5 (C_4), 108.7 (C_3), 112.8 (C_7), 117.2 (C_6), 127.4 (C_2), 127.8 (C_3), 128.2 ($C_{2'}$, $C_{6'}$), 128.4 ($C_{4'}$), 128.6 ($C_{3'}$, $C_{5'}$), 132.3 (C_{7a}), 135.8 ($C_{1'}$), 154.7 (C_5), 161.8 (*C*OOCH_2Ph). Anal. Calcd for $C_{17}H_{15}NO_3$: C, 72.58; H, 5.37; N, 4.98. Found: C, 72.49; H, 5.48; N, 4.82.

Benzyl 5,6-dimethoxy-1*H*-indole-2-carboxylate (20)

The benzyl ester **20** is prepared from the corresponding 5,6-dimethoxy-1*H*-indole-2-carboxylic acid (**19**) upon reaction with benzyl alcohol, according to the general Steglich esterification method described. The obtained residue is chromatographed on a silica column with gradient elution starting from CH_2Cl_2 100% to CH_2Cl_2/AcOEt 100:6, finally affording the desired product (83%) as a white iridescent solid. Mp 146-148 °C (AcOEt/*n*-pentane), R_f = 0.58 (CH_2Cl_2/AcOEt 6:1).

^{1}H NMR (400 MHz, $CDCl_3$) δ (ppm) 3.908 (s, 3H, 6-O*CH*$_3$), 3.913 (s, 3H, 5-O*CH*$_3$), 5.38 (s, 2H, COOC*H*$_2$Ph), 6.83 (s, 1H, H_7), 7.03 (s, 1H, H_4), 7.18 (t, 1H, *J*=1.0 Hz, H_3), 7.32-7.42 (complex m, 3H, $H_{3'}$, $H_{4'}$, $H_{5'}$), 7.45 (d, 2H, *J*=7.4 Hz, $H_{2'}$, $H_{6'}$), 8.95 (brs, 1H, N*H*); ^{13}C NMR (50 MHz, $CDCl_3$) δ (ppm) 56.1 (O*C*H$_3$), 56.3 (O*C*H$_3$), 66.5 (COO*C*H$_2$Ph), 93.9 (C_7), 102.7 (C_4), 109.4 (C_3), 120.6 (C_{3a}), 125.6 (C_2), 128.3 ($C_{2'}$, $C_{6'}$), 128.4 ($C_{4'}$), 128.7 ($C_{3'}$, $C_{5'}$), 132.4 (C_{7a}), 136.1 ($C_{1'}$), 146.4 (C_5 ή C_6 για H 3.913), 150.3 (C_5 ή C_6 για H 3.908), 161.8 (*C*OOCH$_2$Ph). Anal. Calcd for $C_{18}H_{17}NO_4$: C, 69.44; H, 5.50; N, 4.50. Found: C, 69.48; H, 5.63; N, 4.46.

4-Methoxy-benzyl 5-chloro-1*H*-indole-2-carboxylate (26)

To obtain the desired 4-methoxybenzyl ester, the general method described is followed with the difference that 5-chloro-1*H*-indole-2-carboxylic acid (**25**) is esterified with 4-methoxybenzyl alcohol. Purification of the residue by silica column chromatography (*n*-hexane/AcOEt 4:1) afforded the desired product **26** (62%) as a pale yellow crystalline solid.Mp 160-162 °C (AcOEt/*n*-pentane), R_f = 0.34 (*n*-hexane/AcOEt 4:1).

^{1}H NMR (400 MHz, $CDCl_3$) δ (ppm) 3.82 (s, 3H, OC*H*$_3$), 5.33 (s, 2H, COOC*H*$_2$Ph), 6.92 (d, 2H, *J*=8.0 Hz, $H_{3'}$, $H_{5'}$), 7.17 (s, 1H, H_3), 7.25 (d, 1H, *J*=8.5 Hz, H_6), 7.31 (d, 1H, *J*=8.6 Hz, H_7), 7.40 (d, 2H, *J*=8.1 Hz, $H_{2'}$, $H_{6'}$), 7.63 (s, 1H, H_4), 9.14 (brs, 1H, N*H*); ^{13}C NMR (50 MHz, $CDCl_3$) δ (ppm) 55.1 (O*C*H$_3$), 66.2 (COO*C*H$_2$Ph), 107.6 (C_3), 113.5 (C_7), 113.8 ($C_{3'}$, $C_{5'}$), 121.1 (C_4), 125.1 (C_6), 126.7 (C_{3a}), 127.7 (C_5), 127.9 ($C_{1'}$), 128.5 (C_2), 130.0 ($C_{2'}$, $C_{6'}$), 135.7 (C_{7a}), 159.5 ($C_{4'}$), 161.5 (*C*OOCH$_2$Ph). Anal. Calcd for $C_{17}H_{14}ClNO_3$: C, 64.67; H, 4.47; N, 4.44. Found: C, 64.79; H, 4.53; N, 4.48.

4-Methoxybenzyl 7-nitro-1*H*-indole-2-carboxylate (32)

To a solution of 7-nitro-1*H*-indole-2-carboxylic acid (**31**) (1450 mg, 7 mmol) in anhydrous CH_2Cl_2/DMF (10:1, 45 mL) solvent mixture, 4-methoxybenzyl alcohol (1209 mg , 8.75 mmol, 1.25 eq) was added. This is followed by the addition of a catalytic amount of DMAP (171 mg, 1.4 mmol, 0.2 eq) and an equimolar amount of DCC (1445 mg, 7 mmol, 1 eq). The reaction mixture is stirred for 3.5 hours at 38-40 °C and after these hours is filtered out through a Hirsch filter and washed thoroughly with CH_2Cl_2. The filtrate is evaporated to remove CH_2Cl_2 and the remaining residue is then poured into 30 mL mixture of ice/water. The mixture is extracted with AcOEt (3x30 mL) and the combined organic phases are then washed with 1N HCl (2x6 mL), water (2x12 mL), saturated aqueous $NaHCO_3$ (2x15 mL), and saturated aqueous NaCl (2x15 mL). The organic layer is dried over anhydrous Na_2SO_4 and concentrated to dryness under reduced pressure. The resulting dark brown solid residue was purified by silica column chromatography eluting isocratically with CH_2Cl_2 to afford benzylester **32** as a bright yellow solid (82%). Mp 151-153 °C (AcOEt/*n*-pentane), R_f = 0.55 (CH_2Cl_2).

^{1}H NMR (400 MHz, $CDCl_3$) δ (ppm) 3.83 (s, 3H, OC*H*$_3$), 5.36 (s, 2H, COOC*H*$_2$Ph), 6.93 (dd, 2H, J_1=6.7 Hz, J_2=1.9 Hz, $H_{3'}$, $H_{5'}$), 7.27 (t, 1H, H_5), 7.36 (d, 1H, *J*=2.2 Hz, H_3), 7.42 (dd, 2H, J_1=6.7 Hz, J_2=2.0 Hz, $H_{2'}$, $H_{6'}$), 8.03 (dd, 1H, J_1=7.9 Hz, J_2=0.8 Hz, H_4), 8.28 (dd, 1H, J_1=8.0 Hz, J_2=0.8 Hz, H_6), 10.34 (brs, 1H, N*H*); ^{13}C NMR (50 MHz, $CDCl_3$) δ (ppm) 55.5 (O*C*H$_3$), 67.3 (COO*C*H$_2$Ph), 109.8 (C_3), 114.2 ($C_{3'}$, $C_{5'}$), 120.3 (C_5), 122.5 (C_6), 127.5 ($C_{1'}$),

129.9 (C_{3a}), 130.1 (C_{7a}), 130.6 ($C_{2'}$, $C_{6'}$), 130.9 (C_4), 131.1 (C_2), 133.6 (C_7), 160.1 ($C_{4'}$), 160.9 (*C*OOCH$_2$Ph). Anal. Calcd for $C_{17}H_{14}N_2O_5$: C, 62.57; H, 4.32; N, 8.59. Found: C, 62.54; H, 4.33; N, 8.55.

REMARK
Carrying out the reaction, under the same conditions, in dry CH_2Cl_2 instead of a mixture of solvents gave a much lower yield of 26%, possibly due to insolubility of the starting material.

General procedure for the preparation of diesters 3, 9, 15, 21, 27 and 33
Sodium hydride (5.5 mmol, 60% in mineral oil) was added portionwise to a stirred, ice-cold, solution of benzyl (5-substituted-) 1*H*-indole-2-carboxylate (5 mmol) in dry DMF (5 mL). After stirring at room temperature for 1 h under argon, ethyl bromoacetate (5.45 mmol, 1.09 eq), dissolved in dry DMF (1.5 mL) was added dropwise. Stirring was continued at rt for 24 h under argon, and the reaction mixture was then poured onto ice/water mixture (40 mL), and extracted with AcOEt (4x30 mL). The combined organic extracts were washed with water (3x40 mL), brine (3x40 mL), dried (Na_2SO_4), and evaporated *in vacuo*. The crude residue was purified by flash column chromatography on silica gel.

Benzyl 1-(2-ethoxy-2-oxoethyl)-1*H*-indole-2-carboxylate (3)
It was prepared by reacting benzyl ester **2** with ethyl-bromoacetate, following the general procedure for the preparation of diesters. Column chromatography on silica gel, using a mixture of eluents *n*-hexane/AcOEt (4:1), gave **3** (86% yield) as a colorless, clear, viscous oil. R_f = 0.46 (*n*-hexane/AcOEt 3:1).
^{1}H NMR (400 MHz, $CDCl_3$) δ (ppm) 1.24 (t, 3H, *J*=7.1 Hz, COOCH$_2$C*H*$_3$), 4.19 (q, 2H, *J*=7.1 Hz, COOC*H*$_2$CH$_3$), 5.32 (s, 2H, NC*H*$_2$COO), 5.35 (s, 2H, COOC*H*$_2$Ph), 7.18 (td, 1H, J_1=7.9 Hz, J_2=0.8 Hz, H_5), 7.29 (d, 1H, *J*=7.9 Hz, H_7), 7.32-7.48 (complex m, 7H, H_3, $H_{2'}$, $H_{3'}$, $H_{4'}$, $H_{5'}$, $H_{6'}$ and H_6), 7.69 (d, 1H, *J*=8.0 Hz, H_4); ^{13}C NMR (50 MHz, $CDCl_3$) δ (ppm) 14.2 (COOCH$_2$*C*H$_3$), 46.3 (N*C*H$_2$COO), 61.5 (COO*C*H$_2$CH$_3$), 66.4 (COO*C*H$_2$Ph), 109.7 (C_7), 111.7 (C_3), 121.2 (C_5), 123.0 (C_4), 125.7 (C_6), 126.2 (C_2), 127.4 (C_{3a}), 128.2 ($C_{3'}$, $C_{5'}$), 128.3 ($C_{4'}$), 128.7 ($C_{2'}$, $C_{6'}$), 136.0 ($C_{1'}$), 139.6 (C_{7a}), 162.0 (*C*OOCH$_2$Ph), 168.9 (*C*OOCH$_2$CH$_3$). HRMS/ESI$^+$ (m/z): Calcd for $C_{20}H_{19}NO_4$: 353.1627; Found: 353.1633.

Benzyl 1-[(ethoxycarbonyl)methyl]-5-fluoro-1*H*-indole-2-carboxylate (9)
The desired product is prepared by the reaction of ethyl bromoacetate on the corresponding benzyl ester **8**. The yellow solid residue is subjected to flash column chromatography, with an eluent system of *n*-hexane/AcOEt, starting at 7:1 and finishing with 6:1. Diester **9** is isolated as a colorless viscuous oily product (86%) which solidifies upon cooling. Mp 81-83 °C (Et$_2$O/*n*-pentane), R_f = 0.43 (*n*-hexane/AcOEt 6:1).
^{1}H NMR (400 MHz, $CDCl_3$) δ (ppm) 1.24 (t, 3H, *J*=7.1 Hz, COOCH$_2$C*H*$_3$), 4.19 (q, 2H, *J*=7.1 Hz, COOC*H*$_2$CH$_3$), 5.30 (s, 2H, NC*H*$_2$COO), 5.34 (s, 2H, COOC*H*$_2$Ph), 7.12 (td, 1H, J_1=9.0 Hz, J_2=2.5 Hz, H_6), 7.22 (dd, 1H, J_1=9.1 Hz, J_2=4.2 Hz, H_7), 7.32 (dd, 1H, J_1=9.0 Hz, J_2=2.3 Hz, H_4), 7.34-7.47 (complex m, 5H, $H_{2'}$, $H_{3'}$, $H_{4'}$, $H_{5'}$, $H_{6'}$), 7.36 (s, 1H, H_3); ^{13}C NMR (50 MHz, $CDCl_3$) δ (ppm) 14.1 (COOCH$_2$*C*H$_3$), 46.3 (N*C*H$_2$COO), 61.6 (COO*C*H$_2$CH$_3$), 66.5 (COO*C*H$_2$Ph), 106.9, 107.3 (d, J_{C-F}=23.3 Hz, C_4), 110.5, 110.7 (d, J_{C-F}=9.6 Hz, C_7), 111.1, 111.3 (d, J_{C-F}=5.3 Hz, C_3), 114.3, 114.9 (d, J_{C-F}=27.0 Hz, C_6), 126.1, 126.3 (d, J_{C-F}=10.2 Hz, C_{3a}), 128.1 ($C_{2'}$, $C_{6'}$), 128.3 ($C_{4'}$), 128.6 ($C_{3'}$, $C_{5'}$), 130.4 (C_2), 135.7 ($C_{1'}$), 136.1 (C_{7a}), 156.0, 160.7 (d, J_{C-F}=237.4 Hz, C_5), 161.6 (*C*OOCH$_2$Ph), 168.6 (*C*OOCH$_2$CH$_3$). Anal. Calcd for $C_{20}H_{18}FNO_4$: C, 67.60; H, 5.11; N, 3.94. Found: C, 67.49; H, 5.14; N, 3.92.

Benzyl 1-[(ethoxycarbonyl)methyl]-5-methoxy-1*H*-indole-2-carboxylate (15)

The preparation of **15** from the corresponding benzyl ester **14** and ethyl bromoacetate is analogous to that described in the general methodology. Workup affords a yellow, almost solid residue which is then purified by flash column chromatography with a 7:1 *n*-hexane/AcOEt solvent mixture, the polarity of which is then increased to 6:1. A colorless, viscous oily product is obtained (83%) which crystallizes to a white solid upon addition of *n*-pentane and cooling. Mp 88-90 °C (Et_2O/*n*-pentane), R_f = 0.30 (*n*-hexane/AcOEt 6:1).

^{1}H NMR (400 MHz, $CDCl_3$) δ (ppm) 1.24 (t, 3H, *J*=7.1 Hz, $COOCH_2CH_3$), 3.84 (s, 3H, OCH_3), 4.19 (q, 2H, *J*=7.1 Hz, $COOCH_2CH_3$), 5.29 (s, 2H, NCH_2COO), 5.34 (s, 2H, $COOCH_2Ph$), 7.04 (dd, 1H, J_1=9.0 Hz, J_2=2.4 Hz, H_6), 7.07 (d, 1H, *J*=2.2 Hz, H_4), 7.19 (d, 1H, *J*=9.0 Hz, H_7), 7.31-7.48 (complex m, 5H, $H_{2'}$, $H_{3'}$, $H_{4'}$, $H_{5'}$, $H_{6'}$), 7.34 (d, 1H, *J*=0.6 Hz, H_3); ^{13}C NMR (50 MHz, $CDCl_3$) δ (ppm) 14.2 ($COOCH_2CH_3$), 46.4 (NCH_2COO), 55.8 (OCH_3), 61.6 ($COOCH_2CH_3$), 66.3 ($COOCH_2Ph$), 103.1 (C_4), 110.7 (C_7), 111.1 (C_3), 117.2 (C_6), 126.5 (C_{3a}), 127.7 (C_2), 128.2 ($C_{2'}$, $C_{6'}$), 128.3 ($C_{4'}$), 128.7 ($C_{3'}$, $C_{5'}$), 135.2 (C_{7a}), 136.1 ($C_{1'}$), 155.1 (C_5), 161.9 ($COOCH_2Ph$), 169.0 ($COOCH_2CH_3$). Anal. Calcd for $C_{21}H_{21}NO_5$: C, 68.65; H, 5.76; N, 3.81. Found: C, 68.67; H, 5.89; N, 3.92.

Benzyl 1-[(ethoxycarbonyl)methyl]-5,6-dimethoxy-1*H*-indole-2-carboxylate (21)

A solution of the benzyl ester **20** is treated with NaH followed by ethyl bromoacetate and allowed to stir at 35 °C for 48 h under an argon atmosphere. The mixture is worked up as described in the general procedure finally yielding an off-white solid product which is chromatographed, eluting isocratically with CH_2Cl_2. The desired diester **21** (83%) is obtained as a viscous oil which solidifies over time at room temperature. Mp 133-135 °C (AcOEt/n-pentane | After recrystallization a white cotton-like solid is finally obtained), R_f = 0.60 (CH_2Cl_2/AcOEt 10:1).

^{1}H NMR (400 MHz, $CDCl_3$) δ (ppm) 1.24 (t, 3H, *J*=7.1 Hz, $COOCH_2CH_3$), 3.91 (s, 3H, 5-OCH_3), 3.95 (s, 3H, 6-OCH_3), 4.20 (q, 2H, *J*=7.1 Hz, $COOCH_2CH_3$), 5.27 (s, 2H, NCH_2COO), 5.32 (s, 2H, $COOCH_2Ph$), 6.66 (s, 1H, H_7), 7.04 (s, 1H, H_4), 7.32 (s, 1H, H_3) 7.33-7.41 (complex m, 3H, $H_{3'}$, $H_{4'}$, $H_{5'}$), 7.41-7.46 (m, 2H, $H_{2'}$, $H_{6'}$); ^{13}C NMR (50 MHz, $CDCl_3$) δ (ppm) 14.3 ($COOCH_2CH_3$), 46.6 (NCH_2COO), 56.25 (6-OCH_3), 56.34 (5-OCH_3), 61.6 ($COOCH_2CH_3$), 66.1 ($COOCH_2Ph$), 91.9 (C_7), 103.1 (C_4), 111.7 (C_3), 119.1 (C_{3a}), 125.8 (C_2), 128.1 ($C_{2'}$, $C_{6'}$), 128.3 ($C_{4'}$), 128.7 ($C_{3'}$, $C_{5'}$), 135.1 (C_{7a}), 136.3 ($C_{1'}$), 146.6 (C_5), 150.5 (C_6), 161.9 ($COOCH_2Ph$), 169.0 ($COOCH_2CH_3$). Anal. Calcd for $C_{22}H_{23}NO_6$: C, 66.49; H, 5.83; N, 3.52. Found: C, 66.56; H, 5.88; N, 3.51.

4-Methoxybenzyl 5-chloro-1-[(ethoxycarbonyl)methyl]-1*H*-indole-2-carboxylate (27)

It is prepared by the reaction of ethyl bromoacetate on the 4-methoxybenzyl ester **26**, which has been previously treated with NaH, to form the corresponding anion, and stirring this mixture for 24 hours as described in the general method. The resulting oily residue is subjected to column chromatography on silica gel eluting with CH_2Cl_2, to afford **27** (2.24 g, 70%) as a white crystalline solid. Mp 112-114 °C (AcOEt/*n*-pentane, Et_2O | After recrystallization, a white cotton-like solid is finally obtained), R_f = 0.47 (CH_2Cl_2).

^{1}H NMR (400 MHz, $CDCl_3$) δ (ppm) 1.24 (t, 3H, *J*=7.1 Hz, $COOCH_2CH_3$), 3.82 (s, 3H, OCH_3), 4.19 (q, 2H, *J*=7.1 Hz, $COOCH_2CH_3$), 5.27 (s, 2H, $COOCH_2Ph$), 5.28 (s, 2H, NCH_2COO), 6.92 (dd, 2H, J_1=8.7 Hz, J_2=2.1 Hz, $H_{3'}$, $H_{5'}$), 7.20 (d, 1H, *J*=8.9 Hz, H_7), 7.25-7.32 (m, 2H, H_3, H_6), 7.38 (dd, 2H, J_1=8.7 Hz, J_2=2.1 Hz, $H_{2'}$, $H_{6'}$), 7.63 (d, 1H, *J*=1.5 Hz, H_4); ^{13}C NMR (50 MHz, $CDCl_3$) δ (ppm) 14.2 ($COOCH_2CH_3$), 46.4 (NCH_2COO), 55.4 (OCH_3), 61.7 ($COOCH_2CH_3$), 66.6 ($COOCH_2Ph$), 110.8 (C_3), 110.9 (C_7), 114.1 ($C_{3'}$, $C_{5'}$), 122.1 (C_4), 126.1 (C_6), 126.9 (C_{3a}), 127.1 (C_5), 127.9 ($C_{1'}$), 128.8 (C_2), 130.2

($C_{2'}$, $C_{6'}$), 137.9 (C_{7a}), 159.9 ($C_{4'}$), 161.8 (COOCH$_2$Ph), 168.7 (COOCH$_2$CH$_3$). Anal. Calcd for $C_{21}H_{20}ClNO_5$: C, 62.77; H, 5.02; N, 3.49. Found: C, 62.63; H, 5.04; N, 3.35.

4-Methoxybenzyl 1-[(ethoxycarbonyl)methyl]-7-nitro-1*H*-indole-2-carboxylate (33)
An amount of 4-methoxybenzyl ester **32** (326 mg, 1 mmol) was suspended in 5 mL of anhydrous DMF and then gently heated in a water bath (40 °C) until dissolved. The system is allowed to stir until it reaches room temperature. This is followed by the addition of NaH (44 mg, 1.1 mmol, 60% w/w dispersion in mineral oil) portionwise and under cooling.* The mixture is stirred under argon at 0 °C until hydrogen evolution ceases, and then, after removal of ice, stirring is continued at room temperature for 1 additional hour under inert atmosphere. The amount of ethyl bromoacetate (182 mg, 1.09 mmol) dissolved in anhydrous DMF (1 mL) is then added dropwise. Stirring is continued for 24 hours. Excess NaH is neutralized by pouring the reaction mixture into ice/water (20 mL). Then the mixture is extracted with AcOEt (3x20 mL). The combined organic phases are washed with water (3x30 mL) and saturated aqueous NaCl solution (3x20 mL), dried over anhydrous Na_2SO_4, and the organic solvents are evaporated under reduced pressure. Finally, the orange viscuouc oily residue is purified by column chromatography on silica gel. Isocratic elution with CH_2Cl_2 afforded the desired product **33** (82%) as a yellow, clear, viscous oil which crystallized to a yellow solid. Mp 84-86 °C (AcOEt/*n*-pentane, Et_2O), R_f = 0.59 (CH_2Cl_2).
*The round-bottomed flask is cooled just prior to the addition of the NaH in order to prevent precipitation of the dissolved benzyl ester.
^{1}H NMR (400 MHz, CDCl$_3$) δ (ppm) 1.28 (t, 3H, J=7.1 Hz, COOCH$_2$CH_3), 3.82 (s, 3H, OCH_3), 4.23 (q, 2H, J=7.1 Hz, COOCH_2CH$_3$), 5.28 (s, 2H, COOCH_2Ph), 5.46 (brs, 2H, NCH_2COO), 6.92 (d, 2H, J=8.7 Hz, $H_{3'}$, $H_{5'}$), 7.19 (t, 1H, J=7.9 Hz, H_5), 7.38 (d, 2H, J=8.6 Hz, $H_{2'}$, $H_{6'}$), 7.52 (s, 1H, H_3), 7.90 (dd, 1H, J_1=7.9 Hz, J_2=0.8 Hz, H_4), 7.93 (dd, 1H, J_1=7.8 Hz, J_2=0.8 Hz, H_6); ^{13}C NMR (50 MHz, CDCl$_3$) δ (ppm) 14.2 (COOCH$_2$$CH_3$), 48.9 (N$CH_2$COO), 55.4 (O$CH_3$), 61.7 (COO$CH_2CH_3$), 66.9 (COO$CH_2$Ph), 113.1 ($C_3$), 114.1 ($C_{3'}$, $C_{5'}$), 120.1 (C_5), 123.7 (C_6), 127.5 ($C_{1'}$), 129.2 (C_4), 130.3 ($C_{2'}$, $C_{6'}$, C_2), 130.5 (C_{3a}), 130.8 (C_{7a}), 137.6 (C_7), 159.9 ($C_{4'}$), 161.2 (COOCH$_2$Ph), 168.9 (COOCH$_2$CH$_3$). Anal. Calcd for $C_{21}H_{20}N_2O_7$: C, 61.16; H, 4.89; N, 6.79. Found: C, 61.10; H, 4.96; N, 6.82.

General procedure for the preparation carboxylic acids 4, 10, 16 and 22 by hydrogenolysis
A solution of the respective benzyl 1-(2-ethoxy-2-oxoethyl)-1*H*-indole-2-carboxylate (1 mmol) in a mixture of absolute EtOH/AcOEt (2:1, 30 mL) was hydrogenated Pd/C (10% w/w) for 3 h, at room temperature and 50 psi pressure. The catalyst was filtered off (sintered glass funnel), washed with hot EtOH (3x10 mL), and the combined filtrates were evaporated *in vacuo* to afford pure the respective indolecarboxylic acids.

1-(2-Ethoxy-2-oxoethyl)-1*H*-indole-2-carboxylic acid (4)
It was prepared by hydrogenolysis of diester **3** following the general procedure. Evaporation of the solvents gave **4** (96% yield) as a white crystalline solid. Mp 189-191 °C (EtOH/Et_2O), R_f = 0.26 (*n*-hexane/AcOEt 3:1).

^{1}H NMR (400 MHz, CDCl$_3$) δ (ppm) 1.28 (t, 3H, J=7.1 Hz, COOCH$_2$CH_3), 4.24 (q, 2H, J=7.1 Hz, COOCH_2CH$_3$), 5.31 (s, 2H, NCH_2COO), 7.20 (t, 1H, J=7.5 Hz, H_5), 7.31 (d, 1H, J=8.4 Hz, H_7), 7.40 (td, 1H, J_1= 7.7 Hz, J_2=1.0 Hz, H_6), 7.54 (s, 1H, H_3), 7.33 (d, 1H, J=8.0 Hz, H_4); ^{13}C NMR (50 MHz, CDCl$_3$) δ (ppm) 14.3 (COOCH$_2$$CH_3$), 46.4 (N$CH_2$COO), 61.7 (COO$CH_2CH_3$), 109.9 ($C_7$), 113.7 ($C_3$), 121.5 ($C_5$), 123.3 ($C_4$), 126.3 ($C_{3a}$), 126.4 ($C_6$) 126.6 ($C_2$) 140.2 ($C_{7a}$), 167.1 ($C$OOH), 169.0 ($C$OOCH$_2CH_3$). Anal. Calcd for $C_{13}H_{13}NO_4$: C, 63.15; H, 5.30; N, 5.67. Found: C, 63.27; H, 5.63; N, 5.75.

1-(2-Ethoxy-2-oxoethyl)-5-fluoro-1*H*-indole-2-carboxylic acid (10)

It was prepared by hydrogenolysis of diester **9** following the general procedure. Evaporation of the solvents gave **10** (93% yield) as a white crystalline solid. Mp 178-180 °C dec (Et_2O/*n*-pentane), R_f= 0.14 (*n*-hexane/AcOEt 2:1).

^{1}H NMR (400 MHz, DMSO-d_6) δ (ppm) 1.19 (t, 3H, *J*=7.1 Hz, COOCH$_2$C*H*$_3$), 4.13 (q, 2H, *J*=7.1 Hz, COOC*H*$_2$CH$_3$), 5.39 (s, 2H, NC*H*$_2$COO), 7.19 (td, 1H, J_1=9.2 Hz, J_2=2.3 Hz, H_6), 7.24 (s, 1H, H_3), 7.46 (dd, 1H, J_1= 9.4 Hz, J_2=2.3 Hz, H_4), 7.64 (dd, 1H, J_1=9.1 Hz, J_2=4.3 Hz, H_7); ^{13}C NMR (50 MHz, DMSO-d_6) δ (ppm) 14.1 (COOCH$_2$*C*H$_3$), 46.3 (N*C*H$_2$COO), 60.7 (COO*C*H$_2$CH$_3$), 106.1, 106.5 (d, $J_{C\text{-}F}$=23.2 Hz, C_4), 109.6, 109.7 (d, $J_{C\text{-}F}$=5.2 Hz, C_3), 112.1, 112.3 (d, $J_{C\text{-}F}$=9.6 Hz, C_7), 113.1, 113.7 (d, $J_{C\text{-}F}$=26.6 Hz, C_6), 125.4, 125.7 (d, $J_{C\text{-}F}$=10.6 Hz, C_{3a}), 130.2 (C_2), 135.9 (C_{7a}), 155.2, 159.8 (d, $J_{C\text{-}F}$=234.3 Hz, C_5), 162.7 (*C*OOH), 168.9 (*C*OOCH$_2$CH$_3$). Anal. Calcd for $C_{13}H_{12}FNO_4$: C, 58.87; H, 4.56; N, 5.28. Found: C, 58.73; H, 4.55; N, 5.34.

1-(2-Ethoxy-2-oxoethyl)-5-methoxy-1*H*-indole-2-carboxylic acid (16)

It was prepared by hydrogenolysis of diester **15** following the general procedure. Evaporation of the solvents gave **16** (97% yield) as a white crystalline solid. Mp 189-191 °C (AcOEt/*n*-pentane, Et_2O), R_f= 0.06 (*n*-hexane/AcOEt 2:1).

^{1}H NMR (400 MHz, DMSO-d_6) δ (ppm) 1.19 (t, 3H, *J*=7.1 Hz, COOCH$_2$C*H*$_3$), 3.77 (s, 3H, OC*H*$_3$), 4.12 (q, 2H, *J*=7.1 Hz, COOC*H*$_2$CH$_3$), 5.33 (s, 2H, NC*H*$_2$COO), 6.96 (dd, 1H, J_1=9.1 Hz, J_2=2.3 Hz, H_6), 7.15 (d, 1H, *J*=2.4 Hz, H_4), 7.18 (s, 1H, H_3), 7.52 (d, 1H, *J*=9.1 Hz, H_7), 12.94 (brs, 1H, O*H*); ^{13}C NMR (50 MHz, DMSO-d_6) δ (ppm) 14.1 (COOCH$_2$*C*H$_3$), 46.1 (N*C*H$_2$COO), 55.3 (O*C*H$_3$), 60.7 (COO*C*H$_2$CH$_3$), 102.4 (C_4), 109.6 (C_3), 111.7 (C_7), 116.0 (C_6), 125.8 (C_{3a}), 128.4 (C_2), 134.7 (C_{7a}), 154.3 (C_5), 162.8 (*C*OOH), 169.1 (*C*OOCH$_2$CH$_3$). Anal. Calcd for $C_{14}H_{15}NO_5$: C, 60.64; H, 5.45; N, 5.05. Found: C, 60.59; H, 5.42; N, 5.08.

1-[(Ethoxycarbonyl)methyl]-5,6-dimethoxy-1*H*-indole-2-carboxylic acid (22)

A solution of **21** in absolute EtOH/AcOEt is catalytically hydrogenated, in the presence of Pd/C, for 3 h under 50 psi according to the general method. After removal of the catalyst, the filter is washed thoroughly with hot MeOH (~60 °C) and the filtrate is evaporated to dryness under reduced pressure. The carboxylic acid is obtained as a white crystalline solid (almost quantitative yield). Mp 198-200 °C dec (AcOEt/*n*-pentane), R_f= 0.15 (CH_2Cl_2/AcOEt 10:1).

^{1}H NMR (400 MHz, $CDCl_3$) δ (ppm) 1.27 (t, 3H, *J*=7.1 Hz, COOCH$_2$C*H*$_3$), 3.93 (s, 3H, 5-OC*H*$_3$), 3.96 (s, 3H, 6-OC*H*$_3$), 4.24 (q, 2H, *J*=7.1 Hz, COOC*H*$_2$CH$_3$), 5.25, 5.29 (2s, 2H, NC*H*$_2$COO), 6.65 (s, 1H, H_7), 7.07 (s, 1H, H_4), 7.41 (s, 1H, H_3); ^{13}C NMR (50 MHz, DMSO-d_6) δ (ppm) 14.1 (COOCH$_2$*C*H$_3$), 46.3 (N*C*H$_2$COO), 55.7 (5-O*C*H$_3$), 55.8 (6-O*C*H$_3$), 60.5 (COO*C*H$_2$CH$_3$), 93.5 (C_7), 102.8 (C_4), 110.2 (C_3), 118.1 ($C_{3\alpha}$), 126.2 (C_2), 134.7 ($C_{7\alpha}$), 145.8 (C_5), 149.7 (C_6), 162.7 (*C*OOH), 169.1 (*C*OOCH$_2$CH$_3$). Anal. Calcd for $C_{15}H_{17}NO_6$: C, 58.63; H, 5.58; N, 4.56. Found: C, 58.51; H, 5.42; N, 4.72.

General procedure for the preparation carboxylic acids 28 and 34 with TFA

To a stirred solution of the corresponding 4-methoxy-benzyl ester (1 mmol) in 10 mL of anhydrous CH_2Cl_2, an amount of anisole (40 mmol) was added as a scavenger. TFA (54 mmol) is then injected dropwise over a period of 2-3 minutes under an inert atmosphere, while stirring was continued for 3 hours under the same conditions. At the end of the reaction, the solvents and by-products are evaporated under vacuum to a minimum volume. Distilled water is added to the heterogeneous residue, in a 1:1 volume ratio with anisole, to create an azeotropic mixture, which was then concentrated to dryness, to yield the desired carboxylic acids.

5-chloro-1-(2-ethoxy-2-oxoethyl)-1*H*-indole-2-carboxylic acid (28)
Carboxylic acid **28** was prepared by the reaction of TFA with diester **27** in the presence of anisol as scavenger, as described in the general method. The resulting off-white solid residue is subjected to column chromatography on silica gel with a CH_2Cl_2/MeOH 10:1 eluting system. A white crystalline solid (94%) was obtained. Mp 190-192 °C (AcOEt/*n*-pentane, Et_2O), R_f = 0.28 (CH_2Cl_2/MeOH 10:1).
^{1}H NMR (400 MHz, DMSO-d_6) δ (ppm) 1.19 (t, 3H, *J*=7.1 Hz, $COOCH_2CH_3$), 4.13 (q, 2H, *J*=7.1 Hz, $COOCH_2CH_3$), 5.38 (s, 2H, NCH_2COO), 7.25 (s, 1H, H_3), 7.32 (dd, 1H, J_1=8.9 Hz, J_2=2.1 Hz, H_6), 7.69 (d, 1H, *J*=8.9 Hz, H_7), 7.77 (s, 1H, *J*=2.0 Hz, H_4), 13.24 (brs, 1H, COO*H*); ^{13}C NMR (50 MHz, DMSO-d_6) δ (ppm) 14.1 ($COOCH_2CH_3$), 46.3 (NCH_2COO), 60.8 ($COOCH_2CH_3$), 109.5 (C_3), 112.6 (C_7), 121.2 (C_4), 124.8 (C_6), 125.1 (C_5), 126.4 (C_{3a}), 129.7 (C_2), 137.6 (C_{7a}), 162.6 (*C*OOH), 168.8 ($COOCH_2CH_3$). Anal. Calcd for $C_{13}H_{12}ClNO_4$: C, 55.43; H, 4.29; N, 4.97. Found: C, 55.28; H, 4.36; N, 4.99.

1-[(Ethoxycarbonyl)methyl]-7-nitro-1*H*-indole-2-carboxylic acid (34)
A solution of diester **33** is treated first with anisole and then with TFA in a manner analogous to that described in the general methodology. The yellow residue obtained after evaporation of the solvents is subjected to column chromatography on silica gel with a mobile phase of increasing polarity from CH_2Cl_2 to AcOEt. A yellow clear oily product is obtained (98%) which after drying under high vacuum turns into a yellow foamy solid. Mp 155-157 °C (Et_2O/*n*-pentane | After recrystallization a yellow fine crystalline solid is obtained), R_f = 0.08 (CH_2Cl_2).
^{1}H NMR (400 MHz, $CDCl_3$) δ (ppm) 1.31 (t, 3H, *J*=7.1 Hz, $COOCH_2CH_3$), 4.26 (q, 2H, *J*=7.1 Hz, $COOCH_2CH_3$), 5.46 (brs, 2H, NCH_2COO), 7.26 (t, 1H, *J*=7.9 Hz, H_5), 7.69 (s, 1H, H_3), 7.99 (d, 1H, *J*=2.8 Hz, H_4), 8.00 (d, 1H, *J*=2.7 Hz, H_6); ^{13}C NMR (50 MHz, $CDCl_3$) δ (ppm) 14.2 ($COOCH_2CH_3$), 49.1 (NCH_2COO), 62.0 ($COOCH_2CH_3$), 115.2 (C_3), 120.5 (C_5), 124.5 (C_6), 129.6 (C_4), 129.8 (C_2), 130.4 (C_{3a}), 131.0 (C_{7a}), 137.8 (C_7), 165.9 (*C*OOH), 168.9 ($COOCH_2CH_3$). Anal. Calcd for $C_{13}H_{12}N_2O_6$: C, 53.43; H, 4.14; N, 9.59. Found: C, 53.49; H, 4.26; N, 9.61.

General procedure for the preparation of *O*-benzyl hydroxamates 5, 11, 17, 23, 29, 35 and 37
To a solution of the respective carboxylic acid (1 mmol) in a mixture of CH_2Cl_2 and anhydrous DMF (4:1, 10 mL), were added sequentially EDCI·HCl (1.19 mmol), HOBt (1.2 mmol, hydrate 15% wt), DIEA (4 mmol) and finally O-benzyl-hydroxylamine hydrochloride (1.2 mmol). The reaction mixture was heated to 40 °C, and stirred for 48 h under argon atmosphere. The reaction mixture was concentrated under reduced pressure, poured onto ice/water mixture (40 mL), and extracted with AcOEt (4x30 mL). The combined organic extracts were washed sequentially with water (3x30 mL), 10% aqueous Na_2CO_3 (2x30 mL), and saturated aqueous NaCl (2x30 mL). The organic layer is separated, dried with anhydrous Na_2SO_4, and concentrated *in vacuo*. The resulting solid residue was purified through flash column chromatography on silica gel.

2-(Benzyloxy)pyrazino[1,2-a]indole-1,3(2*H*,4*H*)-dione (5)
It was prepared by reacting the respective acid ester **4** with *O*-benzyl hydroxylamine hydrochloride following the general procedure for the preparation of *O*-benzyl hydroxamates. The residue was purified through flash column chromatography on silica gel, using a mixture of eluents *n*-hexane/AcOEt 2:1 increased to AcOEt 100%, to afford **5** (68%) as a white crystalline solid. Mp 219-221 °C (AcOEt/*n*-pentane), R_f = 0.44 (CH_2Cl_2).
^{1}H NMR (600 MHz, DMSO-d_6) δ (ppm) 5.09 (s, 2H, OCH_2Ph), 5.35 (s, 2H, H_4), 7.22 (t, 1H, *J*=7.4 Hz, H_8), 7.39-7.46 (complex m, 5H, $H_{3'}$, $H_{4'}$, $H_{5'}$, H_7, H_{10}), 7.59 (d, 2H, *J*=6.6 Hz, $H_{2'}$, $H_{6'}$), 7.61 (dd, 1H, J_1=8.4 Hz, J_2=0.5 Hz, H_6), 7.79 (d, 1H, *J*=8.0 Hz, H_9); ^{13}C NMR (50 MHz, DMSO-d_6) δ (ppm) 47.8

(C_4), 77.8 (OCH$_2$Ph), 106.7 (C_7), 111.2 (C_6), 121.4 (C_8), 122.7 (C_9), 125.48 (C_{10a}), 125.53 (C_{10}), 126.5 (C_{9a}), 128.4 ($C_{3'}$, $C_{5'}$), 128.9 ($C_{4'}$), 129.4 ($C_{2'}$, $C_{6'}$), 134.4 ($C_{1'}$), 136.6 (C_{5a}), 163.2 (C_1=O, C_3=O). Anal. Calcd for $C_{18}H_{14}N_2O_3$: C, 70.58; H, 4.61; N, 9.15. Found: C, 70.49; H, 4.73; N, 9.33.

2-(Benzyloxy)-8-fluoropyrazino[1,2-a]indole-1,3(2*H*,4*H*)-dione (11)

It was prepared by reacting the respective acid ester **10** with *O*-benzyl hydroxylamine hydrochloride following the general procedure for the preparation of *O*-benzyl hydroxamates. The residue was purified through flash column chromatography on silica gel, using a mixture of eluents *n*-hexane/AcOEt (7:1 and then 2:1), to afford **11** (58%) as a white crystalline solid. Mp 235-237 °C (THF/*n*-pentane), R_f= 0.31 (*n*-hexane/AcOEt 2:1).

^{1}H NMR (400 MHz, DMSO-d_6) δ (ppm) 5.09 (s, 2H, OCH_2Ph), 5.36 (s, 2H, H_4), 7.31 (t, 1H, J_1=9.2 Hz, J_2=2.4 Hz, H_7), 7.37 (s, 1H, H_{10}), 7.38-7.48 (complex m, 3H, $H_{3'}$, $H_{4'}$, $H_{5'}$), 7.52-7.62 (complex m, 3H, H_9, $H_{2'}$, $H_{6'}$), 7.67 (dd, 1H, J_1=9.1 Hz, J_2=4.5 Hz, H_6); ^{13}C NMR (50 MHz, DMSO-d_6) δ (ppm) 48.1 (C_4), 77.8 (OCH$_2$Ph), 106.4, 106.5 (d, J_{C-F}=5.3 Hz, C_{10}), 106.6, 107.1 (d, J_{C-F}=24.0 Hz, C_9), 112.8, 112.9 (d, J_{C-F}=9.6 Hz, C_6), 114.3, 114.8 (d, J_{C-F}=27.1 Hz, C_7), 126.6, 126.8 (d, J_{C-F}=10.8 Hz, C_{9a}), 127.1 (C_{10a}), 128.4 ($C_{3'}$, $C_{5'}$), 128.9 ($C_{4'}$), 129.4 ($C_{2'}$, $C_{6'}$), 133.5 (C_{5a}), 134.4 ($C_{1'}$), 155.2 (C_1=O), 155.5, 160.2 (d, J_{C-F}=235.3 Hz, C_8), 163.1 (C_3=O). Anal. Calcd for $C_{18}H_{13}FN_2O_3$: C, 66.66; H, 4.04; N, 8.64. Found: C, 66.62; H, 4.09; N, 8.56.

2-(Benzyloxy)-8-methoxypyrazino[1,2-a]indole-1,3(2*H*,4*H*)-dione (17)

It was prepared by reacting the respective acid ester **16** with *O*-benzyl hydroxylamine hydrochloride following the general procedure for the preparation of *O*-benzyl hydroxamates. The residue was purified through flash column chromatography on silica gel, using a mixture of eluents starting with CH_2Cl_2 100%, then CH_2Cl_2/AcOEt 10:1 and finally CH_2Cl_2/AcOEt 10:2 to give **17** (59%) as a white crystalline solid. Mp 220-222 °C (THF/*n*-pentane), R_f= 0.31 (CH_2Cl_2).

^{1}H NMR (400 MHz, DMSO-d_6) δ (ppm) 3.79 (s, 3H, OCH_3), 5.08 (s, 2H, OCH_2Ph), 5.31 (s, 2H, H_4), 7.07 (dd, 1H, J_1=9.0 Hz, J_2=2.4 Hz, H_7), 7.23 (d, 1H, J=2.3 Hz, H_9), 7.30 (s, 1H, H_{10}), 7.37-7.48 (complex m, 3H, $H_{3'}$, $H_{4'}$, $H_{5'}$), 7.52 (d, 1H, J=9.1 Hz, H_6), 7.58 (m, 2H, $H_{2'}$, $H_{6'}$); ^{13}C NMR (50 MHz, DMSO-d_6) δ (ppm) 47.9 (C_4), 55.3 (OCH$_3$), 77.8 (OCH$_2$Ph), 102.5 (C_9), 106.2 (C_{10}), 112.2 (C_6), 117.2 (C_7), 125.6 (C_{10a}), 127.1 (C_{9a}), 128.4 ($C_{3'}$, $C_{5'}$), 128.9 ($C_{4'}$), 129.4 ($C_{2'}$, $C_{6'}$), 132.2 (C_{5a}), 134.5 ($C_{1'}$), 154.8 (C_8), 155.2 (C_1=O), 163.3 (C_3=O). Anal. Calcd for $C_{19}H_{16}N_2O_4$: C, 67.85; H, 4.80; N, 8.33. Found: C, 67.92; H, 4.73; N, 8.45.

2-(Benzyloxy)-7,8-dimethoxypyrazino[1,2-a]indole-1,3(2*H*,4*H*)dione (23)
Ethyl 2-{2-[(benzyloxy)carbamoyl]-5,6-dimethoxy-1H-indol-1-yl}acetate (23a)

To prepare the desired derivative **23**, the corresponding carboxylic acid **22** was treated with O-benzylhydroxylamine hydrochloride, as described in the general synthetic methodology. The resulting pale yellow solid residue was purified through flash column chromatography on silica gel, using a mixture of eluents starting with CH_2Cl_2 100% and gradually increased to CH_2Cl_2/AcOEt 100:3. The desired cyclized derivative **23** (48%) was obtained as an off-white crystalline solid. Further elution of the column with CH_2Cl_2/AcOEt 100:8 afforded a beige solid product, which was identified by NMR experiments as the non-cyclized derivative **23a** (8.5%).

2-(Benzyloxy)-7,8-dimethoxypyrazino[1,2-a]indole-1,3(2H,4H)dione (23)

Mp 249-251°C dec (CH_2Cl_2/*n*-pentane), R_f= 0.50 (CH_2Cl_2/AcOEt 10:2).

^{1}H NMR (400 MHz, DMSO-d_6) δ (ppm) 3.79 (s, 3H, 8-OCH_3), 3.86 (s, 3H, 7-OCH_3), 5.07 (s, 2H, OCH_2Ph), 5.26 (s, 2H, H_4), 7.16 (s, 1H, H_6), 7.19 (s, 1H, H_9), 7.26 (s, 1H, H_{10}), 7.37-7.48 (complex m, 3H, $H_{3'}$, $H_{4'}$, $H_{5'}$), 7.58 (d, 2H, J=6.7 Hz, $H_{2'}$, $H_{6'}$); ^{13}C NMR (50 MHz, DMSO-d_6) δ (ppm) 48.0 (C_4), 55.6 (8-OCH$_3$), 55.8 (7-OCH$_3$), 77.7 (C_6), 102.6 (C_9), 107.0 (C_{10}), 119.6 (C_{9a}), 123.4 (C_{10a}), 128.4 ($C_{3'}$, $C_{5'}$), 128.9 ($C_{4'}$), 129.4 ($C_{2'}$, $C_{6'}$), 132.3 (C_{5a}), 134.5 ($C_{1'}$), 146.4 (C_8), 150.3 (C_7), 154.9 (C_1=O), 163.2 (C_3=O). Anal. Calcd for $C_{20}H_{18}N_2O_5$: C, 65.57; H, 4.95; N, 7.65. Found: C, 65.52; H, 4.90; N, 7.65.

Ethyl 2-{2-[(benzyloxy)carbamoyl]-5,6-dimethoxy-1H-indol-1-yl}acetate (23a)

Mp 154-156 °C (CH_2Cl_2/*n*-pentane, Et_2O | After recrystallization, a beige cotton-like solid is finally obtained), R_f = 0.36 (CH_2Cl_2/AcOEt 10:2).
^{1}H NMR (400 MHz, $CDCl_3$) δ (ppm) 1.27 (t, 3H, J=7.1 Hz, CH$_2$CH_3), 3.88 (s, 3H, 5-OCH_3), 3.93 (s, 3H, 6-OCH_3), 4.21 (q, 2H, J=7.1 Hz, COOCH_2CH$_3$), 4.99 (s, 2H, COOCH_2Ph), 5.26 (s, 2H, NCH_2COO), 6.65 (s, 1H, H_7), 6.72 (s, 1H, H_3), 6.96 (s, 1H, H_4), 7.32-7.50 (complex m, 5H, $H_{2'}$, $H_{3'}$, $H_{4'}$, $H_{5'}$, $H_{6'}$), 8.57 (s, 1H, NH); ^{13}C NMR (50 MHz, $CDCl_3$) δ (ppm) 14.3 (COOCH$_2$$CH_3$), 46.3 (N$CH_2$COO), 56.3 (5-O$CH_3$, 6-O$CH_3$), 61.7 (COO$CH_2CH_3$), 78.7 (COO$CH_2$Ph), 92.0 ($C_7$), 102.7 ($C_4$), 106.0 ($C_3$), 118.9 ($C_{3a}$), 126.3 ($C_2$), 128.8 ($C_{3'}$, $C_{5'}$), 128.9 ($C_{4'}$), 129.5 ($C_{2'}$, $C_{6'}$), 134.3 (C_{7a}), 135.4 ($C_{1'}$), 146.5 (C_5), 149.5 (C_6), 162.0 (CONHOCH$_2$Ph), 169.1 (COOCH$_2$CH$_3$). Anal. Calcd for $C_{22}H_{24}N_2O_6$: C, 64.07; H, 5.87; N, 6.79. Found: C, 64.13; H, 5.94; N, 6.82.

2-(Benzyloxy)-6-nitropyrazino[1,2-a]indole-1,3(2*H*,4*H*)-dione (37)

A solution of acid **34** in a mixture of anhydrous CH_2Cl_2/DMF, was treated with EDCI·HCl, HOBt, DIEA and then with O-benzyl-hydroxylamine hydrochloride, in a manner analogous to that described in the general method. The mixture was stirred at 38 °C for ~50 h under an argon atmosphere. After workup, the orange oily residue was chromatographed on a silica gel column, isocratically with CH_2Cl_2. The product obtained by the column chromatography after trituration with *n*-pentane/Et_2O (5:1) afforded a bright yellow crystalline solid (42%).
Mp 188-190 °C dec (AcOEt/*n*-pentane, Et_2O | Small acicular crystals), R_f = 0.33 (*n*-hexane/AcOEt 3:1).
^{1}H NMR (400 MHz, $CDCl_3$) δ (ppm) 5.19 (s, 2H, OCH_2Ph), 5.35 (s, 2H, H_4), 7.34 (t, 1H, J=7.9 Hz, H_8), 7.37-7.43 (complex m, 3H, $H_{3'}$, $H_{4'}$, $H_{5'}$), 7.54-7.62 (complex m, 2H, $H_{2'}$, $H_{6'}$), 7.69 (s, 1H, H_{10}), 8.08 (dd, 1H, J_1=8.0 Hz, J_2=0.8 Hz, H_9), 8.12 (dd, 1H, J_1=7.8 Hz, J_2=0.8 Hz, H_7); ^{13}C NMR (50 MHz, $CDCl_3$) δ (ppm) 51.0 (C_4), 79.3 (OCH$_2$Ph), 111.0 (C_{10}), 121.4 (C_8), 124.6 (C_7), 125.4 (C_{10a}), 128.1 (C_{5a}), 128.8 ($C_{3'}$, $C_{5'}$), 129.7 ($C_{4'}$), 130.22 ($C_{2'}$, $C_{6'}$), 130.24 (C_9), 131.7 (C_{9a}), 133.4 ($C_{1'}$), 137.2 (C_6), 155.9 (C_1=O), 161.9 (C_3=O). Anal. Calcd for $C_{18}H_{13}N_3O_5$: C, 61.54; H, 3.73; N, 11.96. Found: C, 61.49; H, 3.78; N, 11.99.

General method for the synthesis of N-[(4-methoxybenzyl)oxy] imides 29 and 35

To a stirred solution of the corresponding carboxylic acid (1 mmol) in a mixture of anhydrous solvents CH_2Cl_2/DMF (4:1, 10 mL), EDCI·HCl (1.19 mmol), HOBt (1.2 mmol, hydrate 15% wt) and DIEA (2.5 mmol) were added sequentially. Next, O-(4-methoxybenzyl)hydroxylamine (1.2 mmol) dissolved in 1.5 mL of anhydrous CH_2Cl_2 was added. The mixture was heated to 35 °C and stirred for 48-65 h under an inert argon atmosphere. The reaction mixture was then cooled to room temperature and the CH_2Cl_2 is removed *in vacuo*. The residue was poured onto ice/water mixture (20 mL) and extracted with AcOEt (3x30 mL). The combined organic layers were then washed with water (3x30 mL), 10% aqueous Na_2CO_3 (2x30 mL), and saturated aqueous NaCl (2x30 mL). The combined organic exctracts were dried with anhydrous Na_2SO_4 and evaporated *in vacuo*. The residue was purified through flash column chromatography on silica gel.

8-Chloro-2-[(4-methoxybenzyl)oxy]pyrazino[1,2-a]indole-1,3(2*H*,4*H*)-dione (29)

The titled compound **29** was prepared by the reaction of O-(4-methoxybenzyl)hydroxylamine on the acid **28** obtained from the previous step, according to the general synthetic method described (stirring for 48h). After flash column chromatography (CH_2Cl_2/MeOH 10:1) a white solid was obtained (78%). Mp 238-240 °C dec (AcOEt/*n*-pentane), R_f= 0.75 (CH_2Cl_2/MeOH 9.5:0.5).
^{1}H NMR (400 MHz, DMSO-d_6) δ (ppm) 3.78 (s, 3H, OCH_3), 5.00 (s, 2H, OCH_2Ph), 5.35 (s, 2H, H_4), 6.98 (d, 2H, *J*=8.4 Hz, $H_{3'}$, $H_{5'}$), 7.36 (s, 1H, H_{10}), 7.43 (dd, 1H, J_1=8.9 Hz, J_2=1.4 Hz, H_7), 7.50 (d, 2H, *J*=8.4 Hz, $H_{2'}$, $H_{6'}$), 7.66 (d, 1H, *J*=8.9 Hz, H_6), 7.86 (d, 1H, *J*=1.5 Hz, H_9); ^{13}C NMR (50 MHz, DMSO-d_6) δ (ppm) 48.0 (C_4), 55.1 (OCH_3), 77.5 (OCH_2Ph), 106.0 (C_{10}), 113.1 (C_6), 113.8 ($C_{3'}$, $C_{5'}$), 121.7 (C_9), 125.6 (C_7), 126.0 (C_{3a}), 126.4 (C_5), 127.0 (C_{10a}), 127.5 ($C_{1'}$), 131.3 ($C_{2'}$, $C_{6'}$), 135.0 (C_{5a}), 155.2 ($C_{4'}$), 159.8 (C_1=O), 163.1 (C_3=O). Anal. Calcd for $C_{19}H_{15}ClN_2O_4$: C, 61.55; H, 4.08; Cl, 9.56; N, 7.56. Found: C, 61.66; H, 4.12; N, 7.62.

2-[(4-Methoxybenzyl)oxy]-6-nitropyrazino[1,2-a]indole-1,3(2H,4H)-dione (35)
The title compound **35** was obtained from the carboxylic acid **34** upon reaction with EDCI·HCl, HOBt, DIEA and O-(4-methoxybenzyl)hydroxylamine, in a manner similar to that described in the general methodology. The system was stirred at 35 °C for 65 h under argon atmosphere. The dark viscuos oily residue, resulting from the work-up, was purified by flash column chromatography using CH_2Cl_2 as an eluent. The cyclized derivative **35** was obtained as a yellow crystalline solid (26%). Mp 173-175 °C dec (AcOEt/*n*-pentane, Et_2O), R_f= 0.41 (CH_2Cl_2).
^{1}H NMR (400 MHz, $CDCl_3$) δ (ppm) 3.81 (s, 3H, OCH_3), 5.14 (s, 2H, OCH_2Ph), 5.35 (s, 2H, H_4), 6.91 (d, 2H, *J*=8.7 Hz, $H_{3'}$, $H_{5'}$), 7.34 (t, 1H, *J*=7.9 Hz, H_8), 7.51 (d, 2H, *J*=8.6 Hz, $H_{2'}$, $H_{6'}$), 7.70 (s, 1H, H_{10}), 8.08 (dd, 1H, J_1=8.0 Hz, J_2=0.9 Hz, H_9), 8.12 (dd, 1H, J_1=7.8 Hz, J_2=0.9 Hz, H_7); ^{13}C NMR (50 MHz, $CDCl_3$) δ (ppm) 51.0 (C_4), 55.4 (OCH_3), 78.9 (OCH_2Ph), 110.9 (C_{10}), 114.1 ($C_{3'}$, $C_{5'}$), 121.3 (C_8), 124.6 (C_7), 125.5 ($C_{1'}$), 128.3 (C_{10a}), 130.2 (C_9), 131.6 (C_{5a}), 132.0 ($C_{2'}$, $C_{6'}$), 134.0 (C_{9a}), 137.2 (C_6), 154.8 (C_1=O), 160.7 ($C_{4'}$), 162.0 (C_3=O). Anal. Calcd for $C_{19}H_{15}N_3O_6$: C, 59.84; H, 3.96; N, 11.02. Found: C, 59.80; H, 3.99; N, 11.10.

General method for the synthesis of N-hydroxy-imides 6, 12, 18, 24 and 38 by catalytic hydrogenation
1 mmol of the corresponding N-(benzyloxy)-substituted diketopiperazine of the previous step was dissolved in a hot mixture of abs. EtOH/AcOEt/MeOH (2:1:0.5, 135 mL) and placed in a hydrogenation flask. Pd/C catalyst (10% w/w) was added and the mixture was hydrogenated for 3 hours at 43 °C and 52-55 psi. After the completion of the reaction, the catalyst was filtered off, through a sintered glass funnel, washed sufficiently with a mixture of hot abs. EtOH/MeOH (3x10 mL). The combined filtrates were concentrated under reduced pressure to give the final hydroxamic acids which were then chromatographed on a silica gel column to provide pure the final *N*-hydroxyimides. The experimental procedure differs in the case of the dimethoxy analogue **24**.

2-Hydroxypyrazino[1,2-a]indole-1,3(2*H*,4*H*)-dione (6)
It was prepared by hydrogenolysis of the corresponding diketopiperazine analogue **5** following the general procedure. After evaporation of the solvents, the residue was purified by column chromatography on silica gel using a mixture of eluents AcOEt/MeOH (5:1), to afford **6** (almost quantitative yield) as a pale yellow crystalline solid. Mp 215-216 °C dec (MeOH/Et_2O), R_f= 0.31 (AcOEt).
^{1}H NMR (400 MHz, DMSO-d_6) δ (ppm) 5.33 (s, 2H, H_4), 7.20 (td, 1H, J_1=7.8 Hz, J_2=0.6 Hz, H_8), 7.34 (s, 1H, H_{10}), 7.39 (td, 1H, J_1=7.7 Hz, J_2=1.0 Hz, H_7), 7.57 (dd, 1H, J_1=8.4 Hz, J_2=0.7 Hz, H_6), 7.76 (d, 1H, *J*=8.1 Hz, H_9), 10.64 (brs, 1H, N-O*H*); ^{13}C NMR (50 MHz, DMSO-d_6) δ (ppm) 47.4 (C_4), 106.2

(C_{10}), 111,2 (C_6), 121.3 (C_8), 122.6 (C_9), 125.3 (C_7), 125.6 (C_{10a}), 126.6 (C_{9a}), 136.6 (C_{5a}), 156.0 (C_1=O), 163.5 (C_3=O). Anal. Calcd for $C_{11}H_8N_2O_3$: C, 61.11; H, 3.73; N, 12.96. Found: C, 61.15; H, 3.81; N, 12.92.

8-Fluoro-2-hydroxypyrazino[1,2-a]indole-1,3(2*H*,4*H*)-dione (12)
It was prepared by hydrogenolysis of the corresponding diketopiperazine analogue **11** following the general procedure. After evaporation of the solvents the residue was purified by column chromatography on silica gel, using a mixture of eluents AcOEt/MeOH (5:1), to afford **12** (almost quantitative yield) as a pale yellow crystalline solid. Mp 205-207 °C dec (AcOEt, MeOH/*n*-pentane), R_f= 0.30 (AcOEt).
^{1}H NMR (600 MHz, DMSO-d_6) δ (ppm) 5.34 (s, 2H, H_4), 7.28 (td, 1H, J_1=9.2 Hz, J_2=2.5 Hz, H_7), 7.30 (s, 1H, H_{10}), 7.53 (dd, 1H, J_1=9.6 Hz, J_2=2.5 Hz, H_9), 7.62 (dd, 1H, J_1=9.1 Hz, J_2=4.4 Hz, H_6), 10.70 (brs, 1H, N-OH); ^{13}C NMR (50 MHz, DMSO-d_6) δ (ppm) 47.4 (C_4), 105.9, 106.0 (d, J_{C-F}=5.0 Hz, C_{10}), 106.5, 107.0 (d, J_{C-F}=23.7 Hz, C_9), 112.7, 112.9 (d, J_{C-F}=9.7 Hz, C_6), 113.9, 114.5 (d, J_{C-F}=27.1 Hz, C_7), 126.7, 126.9 (d, J_{C-F}=10.7 Hz, C_{9a}), 127.2 (C_{10a}), 155.5, 160.2 (d, J_{C-F}=235.2 Hz, C_8), 155.8 (C_1=O), 163.4 (C_3=O). Anal. Calcd for $C_{11}H_7FN_2O_3$: C, 56.42; H, 3.01; N, 11.96. Found: C, 56.45; H, 3.08; N, 11.92.

2-Hydroxy-8-methoxypyrazino[1,2-a]indole-1,3(2*H*,4*H*)-dione (18)
It was prepared by hydrogenolysis of the corresponding diketopiperazine analogue **17** following the general procedure. After evaporation of the solvents the residue was purified by column chromatography on silica gel using a mixture of eluents AcOEt/MeOH (5:1), to afford **18** (almost quantitative yield) as a pale yellow crystalline solid. Mp 228-230 °C dec (AcOEt, MeOH/*n*-pentane), R_f= 0.26 (AcOEt).
^{1}H NMR (400 MHz, DMSO-d_6) δ (ppm) 3.78 (s, 3H, OCH_3), 5.29 (s, 2H, H_4), 7.04 (dd, 1H, J_1=9.0 Hz, J_2=1.8 Hz, H_7), 7.20 (d, 1H, J=1.5 Hz, H_9), 7.23 (s, 1H, H_{10}), 7.48 (d, 1H, J=9.1 Hz, H_6), 10.62 (brs, 1H, N-OH); ^{13}C NMR (50 MHz, DMSO-d_6) δ (ppm) 47.5 (C_4), 55.3 (OCH$_3$), 102.5 (C_9), 105.7 (C_{10}), 112.2 (C_6), 116.9 (C_7), 125.7 (C_{10a}), 127.1 (C_{9a}), 132.1 (C_{5a}), 154.8 (C_8), 155.9 (C_1=O), 163.5 (C_3=O). Anal. Calcd for $C_{12}H_{10}N_2O_4$: C, 58.54; H, 4.09; N, 11.38. Found: C, 58.57; H, 4.11; N, 11.42.

2-Hydroxy-7,8-dimethoxypyrazino[1,2-a]indole-1,3(2H,4H)-dione (24)
To prepare the desired final product **24**, (0.35 mmol) of the precursor diketopiperazine analogue **23** was dissolved in a solvent mixture of CH_3COOH/AcOEt/MeOH (8.5:2:1, 150 mL) with heating at 50-60 °C. Pd/C catalyst (10% w/w) was then added and the mixture was hydrogenated for 4 h at 45 °C and 55 psi. The catalyst was removed with vacuum filtration and washed with a mixture of hot abs. EtOH/MeOH (4x10 mL) to give a pale yellow filtrate which is evaporated under reduced pressure. The excess of CH_3COOH was achieved by adding cyclohexane in a 9:1 (cyclohexane/CH_3COOH) volume ratio, and subsequent evaporation of the azeotropic mixture. The resulting yellow solid residue was subjected to regular phase preparative TLC with 10:1 AcOEt/MeOH mobile phase. The first band was scratched from the baseline with R_f=0.37 (AcOEt/MeOH 10:1) and the desired product is extracted with a mixture of dry THF/$CHCl_3$/Acetone 1:1:1 +2% CH_3COOH and with the aid of ultrasound. The silica is filtered under vacuum and washed sufficiently with the same mixture of solvents and finally the filtrate is evaporated under vacuum to dryness with the aid of cyclohexane. The resulting solid product is treated with dry Et_2O to give the desired N-hydroxy-imide (90%) as an orange crystalline solid. Mp 238-240 °C dec (Acetone, MeOH/*n*-pentane), R_f= 0.24 (CH_2Cl_2/Acetone 1:1).
^{1}H NMR (400 MHz, DMSO-d_6) δ (ppm) 3.78 (s, 3H, 8-OCH_3), 3.85 (s, 3H, 7-OCH_3), 5.26 (s, 2H, NCH_2CO), 7.13 (s, 1H, H_6), 7.18 (s, 1H, H_9), 7.20 (s, 1H, H_{10}), 10.54 (brs, 1H, N-OH); ^{13}C NMR (50 MHz, DMSO-d_6) δ (ppm) 47.6 (NCH$_2$CO), 55.7 (8-OCH$_3$), 55.8 (7-OCH$_3$), 93.4 (C_6), 102.7 (C_9), 106.5

(C_{10}), 119.7 (C_{9a}), 123.6 (C_{10a}), 132.2 (C_{5a}), 146.4 (C_8), 150.1 (C_7), 155.6 (C_1=O), 163.5 (C_3=O). Anal. Calcd for $C_{13}H_{12}N_2O_5$: C, 56.52; H, 4.38; N, 10.14. Found: C, 56.49; H, 4.39; N, 10.12.

Synthesis of 2,8-dihydroxypyrazino[1,2-a]indole-1,3(2*H*,4*H*)-dione (39)

Compound **12** (2-hydroxy-8-methoxypyrazino[1,2-a]indole-1,3(2*H*,4*H*)-dione) (100 mg, 0.41 mmol) was suspended in dry CH_2Cl_2 (2 mL) and cooled at 0 ^oC. BBr_3 (1M in CH_2Cl_2, 1.35 mL, 1.35 mmol) was added dropwise and the mixture was stirred at rt, for 20 h under argon atmosphere. Ice-water (20 mL) was then added and CH_2Cl_2 was evaporated *in vacuo*. The residue was extracted with AcOEt (3x15 mL). The combined organic extracts were washed with water (2x10 mL), brine (2x10 mL), dried (Na_2SO_4), and evaporated *in vacuo* to afford **39** (93 mg, almost quantitative yield) as a pale yellow crystalline solid. Mp >250 °C (dec, AcOEt, MeOH/*n*-pentane), R_f= 0.14 (AcOEt).

^{1}H NMR (400 MHz, DMSO-d_6) δ (ppm) 5.29 (s, 2H, H_4), 6.92 (dd, 1H, J_1=8.9 Hz, J_2=1.9 Hz, H_7), 7.00 (d, 1H, J=1.8 Hz, H_9), 7.14 (s, 1H, H_{10}), 7.33 (d, 1H, J=8.9 Hz, H_6), 9.18 (bs, 1H, C-O*H*), 10.58 (bs, 1H, N-O*H*); ^{13}C NMR (100 MHz, DMSO-d_6) δ (ppm) 47.4 (C_4), 105.0 (C_9), 105.2 (C_{10}), 111.8 (C_6), 116.9 (C_7), 125.6 (C_{10a}), 127.5 (C_{9a}), 131.7 (C_{5a}), 152.3 (C_8), 155.9 (C_1=O), 163.6 (C_3=O). Anal. Calcd for $C_{11}H_8N_2O_4$: C, 56.90; H, 3.47; N, 12.06. Found C, 56.82; H, 3.31; N, 12.10.

Synthesis of pyrazino[1,2-a]indole-1,3(2*H*,4*H*)-dione (40)

To a solution of **4** (1-(2-ethoxy-2-oxoethyl)-1*H*-indole-2-carboxylic acid) (600 mg, 2.43 mmol) in dry THF (7 mL) was added dropwise, under ice-cooling, a solution of thionyl chloride (524 mg, 4.40 mmol) in dry THF (0.6 mL). The mixture was stirred at 50 ^oC for 4 h and then at room temperature for another 2 h. The mixture was evaporated *in vacuo*, at low temperature (<35 ^oC) and the crude chloride was dissolved in dry THF (5 mL). To this solution was added, in one portion, a saturated solution of ammonia in dichloromethane (5 mL) and the mixture was stirred at 30 °C for 17 h. After removal of the solvents the crude residue was purified by flash column chromatography on silica gel, using a mixture of eluents *n*-hexane/THF (2:1), to afford pure the target compound **40** (410 mg, 84%) as a white crystalline solid; mp 239-241 °C (dec, THF/*n*-pentane| After recrystallization a white cotton-like solid is finally obtained), R_f= 0.57 (*n*-hexane/THF 1:1).

^{1}H NMR (400 MHz, DMSO-d_6) δ (ppm) 5.11 (s, 2H, H_4), 7.19 (td, 1H, J_1=7.8 Hz, J_2=0.6 Hz, H_8), 7.31 (s, 1H, H_{10}), 7.38 (td, 1H, J_1=7.7 Hz, J_2=1.0 Hz, H_7), 7.57 (dd, 1H, J_1=8.4 Hz, J_2=0.7 Hz, H_6), 7.76 (d, 1H, J=8.1 Hz, H_9), 11.69 (s, 1H, N*H*); ^{13}C NMR (100 MHz, DMSO-d_6) δ (ppm) 46.3 (C_4), 105.6 (C_{10}), 111,2 (C_6), 121.3 (C_8), 122.7 (C_9), 125.1 (C_7), 125.6 (C_{10a}), 126.7 (C_{9a}), 136.7 (C_{5a}), 159.0 (C_1=O), 168.2 (C_3=O). Anal. Calcd for $C_{11}H_8N_2O_2$: C, 66.00; H, 4.03; N, 13.99. Found: C, 66.13; H, 4.01; N, 13.81.

6-Amino-2-hydroxypyrazino[1,2-a]indole-1,3(2H,4H)-dione (38)

A solution of the diketopiperazine precursor **37** was subjected to catalytic hydrogenation for 4.5 h at 43-44 °C and 55 psi. After removing the catalyst and evaporating the filtrate to dryness, a yellow solid product was obtained which was purified on a silica gel column using a mixture of increasing polarity eluents from AcOEt 100% to AcOEt/MeOH 70:30. The desired hydroxy-imide is finally obtained as a yellow-orange fine crystalline solid (92%). Mp >250 °C dec (trit. *n*-pentane/Et_2O), R_f= 0.21 (AcOEt).

^{1}H NMR (600 MHz, DMSO-d_6) δ (ppm) 5.10 (brs, 2H, NH_2), 5.73 (s, 2H, H_4), 6.61 (dd, 1H, J_1=7.4 Hz, J_2=0.9 Hz, H_7), 6.88 (t, 1H, J=7.7 Hz, H_8), 7.02 (dd, 1H, J_1=8.0 Hz, J_2=0.9 Hz, H_9), 7.21 (s, 1H, H_{10}), 10.58 (brs, 1H, N-O*H*); ^{13}C NMR (50 MHz, DMSO-d_6) δ (ppm) 49.9 (C_4), 107.1 (C_{10}), 111.5 (C_7), 112.0 (C_9), 122.3 (C_8), 125.6 (C_{10a}), 128.5 (C_{5a}, C_{9a}), 135.8 (C_6), 156.0 (C_1=O), 163.7 (C_3=O). Anal. Calcd for $C_{11}H_9N_3O_3$: C, 57.14; H, 3.92; N, 18.17. Found: C, 57.20; H, 3.99; N, 18.20.

General method for the synthesis of N-hydroxy-imides 30, 36 using TFA

To a stirred solution of the appropriately substituted N-[(4-methoxybenzyl)oxy]diketopiperazine (1 mmol) in 28 mL anhydrous CH_2Cl_2, anisole (40 mmol) was first added, and then TFA (54 mmol) was added dropwise in 2-3 minutes. Stirring was continued for 3 to 4 hours under an inert atmosphere and at 30 °C. The progress of the reaction was monitored by normal phase TLC. After the end of the reaction, the volatile solvents c are evaporated under vacuum to a minimum volume, while distilled water was added to the heterogeneous residue, in a 1:1 volume ratio with anisole, to create an azeotropic mixture. This was then concentrated to dryness and the resulting solid residue was chromatographed on a silica gel column to give pure the desired final product.

8-Chloro-2-hydroxypyrazino[1,2-*a*]indole-1,3(2*H*,4*H*)-dione (30)

To a solution of 8-chloro-2-((4-methoxybenzyl)oxy)pyrazino[1,2-*a*]indole-1,3(2*H*,4*H*)-dione **29** (200 mg, 0.54 mmol) and anisole (2.34 g, 21.6 mmol) in dry dichloromethane (15.2 mL) was added dropwise TFA (4 mL, 53.00 mmol) and the mixture was stirred for 3 h at 30 °C under argon. After removal of the solvents the crude residue was purified by flash column chromatography using a mixture of eluents AcOEt/MeOH (5:1), to afford **30** (112 mg, 83%) as a pale yellow crystalline. Mp 209-211 °C dec (AcOEt, MeOH/*n*-pentane), R_f = 0.29 (CH_2Cl_2/AcOEt 1:1).
^{1}H NMR (400 MHz, DMSO-d_6) δ (ppm) 5.34 (s, 2H, H_4), 7.30 (s, 1H, H_{10}), 7.40 (dd, 1H, J_1=8.8 Hz, J_2=1.0 Hz, H_7), 7.63 (d, 1H, J=8.9 Hz, H_6), 7.83 (s, 1H, H_9), 10.71 (s, 1H, N-O*H*); ^{13}C NMR (50 MHz, DMSO-d_6) δ (ppm) 47.6 (C_4), 105.5 (C_{10}), 113.0 (C_6), 121.6 (C_9), 125.3 (C_7), 125.9 (C_{9a}), 127.0 (C_{10a}), 127.5 (C_8), 134.9 (C_{5a}), 155.8 (C_1=O), 163.3 (C_3=O). Anal. Calcd for $C_{11}H_7ClN_2O_3$: C, 52.71; H, 2.82; N, 11.18. Found: C, 52.76; H, 2.83; N, 11.22.

2-Hydroxy-6-nitropyrazino[1,2-a]indole-1,3(2H-4H)-dione (36)

A solution of the diketopiperazine precursor **35** was treated sequentially with anisole and TFA as described in the general method. The dark yellow solid residue obtained after evaporation of the solvents was chromatographed on a silica gel column using a mixture of increasing polarity eluents from 100% CH_2Cl_2 to 100% AcOEt and finally to 10% AcOEt/MeOH. A dark brown fine crystalline solid was obtained (almost quantitative yield) which was identified as the desired product. Mp 196-198 °C dec (trit. *n*-pentane/Et_2O), R_f = 0.32 (AcOEt).
^{1}H NMR (600 MHz, DMSO-d_6) δ (ppm) 5.39 (s, 2H, H_4), 7.38 (t, 1H, J=7.9 Hz, H_8), 7.66 (s, 1H, H_{10}), 8.12 (dd, 1H, J_1=7.8 Hz, J_2=1.0 Hz, H_7), 8.22 (dd, 1H, J_1=7.9 Hz, J_2=1.1 Hz, H_9), 10.89 (brs, 1H, N-O*H*); ^{13}C NMR (50 MHz, DMSO-d_6) δ (ppm) 50.7 (C_4), 108.2 (C_{10}), 120.6 (C_8), 123.5 (C_7), 127.0 (C_{10a}), 129.2 (C_{5a}), 129.9 (C_9), 130.9 (C_{9a}), 136.8 (C_6), 155.6 (C_1=O), 163.3 (C_3=O). Anal. Calcd for $C_{11}H_7N_3O_5$: C, 50.58; H, 2.70; N, 16.09. Found: C, 50.49; H, 2.65; N, 16.03.

Indole ring acetohydroxamic acids

General method for the synthesis of analogues 41, 45, 49

To a stirred solution of 1 mmol of the corresponding carboxylic acid (**4**, **10**, **28**) in a mixture of anhydrous CH_2Cl_2 and anhydrous DMF (4:1, 10 mL), EDCI·HCl (1.19 mmol), HOBt (1.2 mmol, hydrate 15 % wt) and DIEA or NMM (*N*-methyl morpholine) (3.6 mmol) were sequentially added. This was followed by the addition of O-(4-methoxybenzyl)glycine hydrochloride (1.2 mmol) dissolved in 1.5 mL dry CH_2Cl_2. The reaction mixture was heated to 35-40 °C and stirred for 48-70 h (monitoring by TLC) under argon atmosphere. The mixture was then allowed to cool to room temperature, the CH_2Cl_2 was removed by evaporation *in vacuo* and the residue was poured into ice/water (20 mL). The mixture was then

extracted with AcOEt (3x30 mL) and the combined organic extracts were washed sequentially with water (3x20 mL), 10% aqueous Na_2CO_3 (2x20 mL), and saturated aqueous NaCl (2x20 mL). The organic phase dried with anhydrous Na_2SO_4 and evaporated under reduced pressure. The resulting residue was purified through flash column chromatography on silica gel.

2-[1,3-dioxo-3,4-dihydropyrazino[1,2-a]indol-2(1*H*)-yl]acetate 4-Methoxybenzyl ester (41) and ethyl 2-{2-[[((4-methoxybenzyl)oxycarbonyl)methyl]carbamoyl]-1*H*-indol-1-yl}acetate (41a)

To prepare the desired derivative **41**, the corresponding carboxylic acid **4** was treated with EDCI·HCl, HOBt, NMM and O-(4-methoxybenzyl)glycine hydrochloride. The reaction mixture was stirred at 40 °C under argon for 70 h. This was followed by treatment of the mixture as described in the general synthetic methodology. The resulting pale yellow solid residue was purified by silica gel flash column chromatography using initially 100% CH_2Cl_2, and then a mixture of CH_2Cl_2 /AcOEt 100:1 to afford the desired cyclized product **41** (39%) as an off-white crystalline solid. Further elution of the column with a CH_2Cl_2 /AcOEt mixture of gradually increasing polarity up to 100:10 yielded a colorless, clear, viscuous oily product which solidified over time at room temperature. After performing a ^{1}H NMR experiment it was identified as the non-cyclized derivative **41a** (5.8%).

2-[1,3-dioxo-3,4-dihydropyrazino[1,2-a]indol-2(1*H*)-yl]acetate 4-Methoxybenzyl ester (41)

Mp 201-203 °C (AcOEt/*n*-pentane), R_f = 0.75 (CH_2Cl_2/AcOEt 10:1).

^{1}H NMR (400 MHz, $CDCl_3$) δ (ppm) 3.81 (s, 3H, OCH_3), 4.79 (s, 2H, NCH_2COO), 5.12 (s, 2H, H_4), 5.15 (s, 2H, COOCH_2Ph), 6.90 (d, 2H, *J*=8.4 Hz, $H_{3'}$, $H_{5'}$), 7.26 (t, 1H, *J*=7.6 Hz, H_8), 7.30 (d, 2H, *J*=8.4 Hz, $H_{2'}$, $H_{6'}$), 7.35 (d, 1H, *J*=8.4 Hz, H_6), 7.45 (t, 1H, *J*=7.6 Hz, H_7), 7.51 (s, 1H, H_{10}), 7.79 (d, 1H, *J*=8.1 Hz, H_9); ^{13}C NMR (50 MHz, $CDCl_3$) δ (ppm) 40.7 (NCH_2COO), 46.5 (C_4), 55.4 (OCH_3), 67.6 (COOCH_2Ph), 109.5 (C_{10}), 110.0 (C_6), 114.2 ($C_{3'}$, $C_{5'}$), 122.3 (C_8), 123.7 (C_9), 124.1 (C_{10a}), 126.6 (C_7), 127.3 ($C_{1'}$), 127.4 (C_{9a}), 130.4 ($C_{2'}$, $C_{6'}$), 137.2 (C_{5a}), 157.8 (C_1=O), 160.0 ($C_{4'}$), 166.2 (C_3=O), 167.5 (*C*OOCH$_2$Ph). Anal. Calcd for $C_{21}H_{18}N_2O_5$: C, 66.66; H, 4.80; N, 7.40. Found: C, 66.71; H, 4.89; N, 7.48.

Ethyl 2-{2-[[((4-methoxybenzyl)oxycarbonyl)methyl]carbamoyl]-1*H*-indol-1-yl}acetate (41a)

Mp 121-123 °C (*n*-pentane | Λευκό κρυσταλλικό στερεό), R_f = 0.61 (CH_2Cl_2/AcOEt 10:1).

^{1}H NMR (600 MHz, $CDCl_3$) δ (ppm) 1.25 (t, 3H, *J*=7.1 Hz, COOCH$_2$$CH_3$), 3.81 (s, 3H, O$CH_3$), 4.15 (d, 2H, *J*=5.3 Hz, NHCH_2COO), 4.19 (q, 2H, *J*=7.1 Hz, COOCH_2CH$_3$), 5.16 (s, 2H, COOCH_2Ph), 5.26 (s, 2H, NCH_2COO), 6.84 (brt, 1H, *J*=5.3 Hz, CON*H*CH$_2$), 6.90 (dd, 2H, J_1=6.6 Hz, J_2=2.1 Hz, $H_{3'}$, $H_{5'}$), 7.00 (d, 1H, *J*=0.6 Hz, H_3), 7.17 (td, 1H, J_1=7.9 Hz, J_2=0.9 Hz, H_5), 7.25 (dd, 1H, J_1=8.4 Hz, J_2=0.7 Hz, H_7), 7.31 (dd, 2H, J_1=6.6 Hz, J_2=2.1 Hz, $H_{2'}$, $H_{6'}$) overlapped with 7.33 (td, 1H, J_1=8.3 Hz, J_2=1.1 Hz, H_6), 7.65 (dt, 1H, J_1=8.0 Hz, J_2=0.9 Hz, H_4); ^{13}C NMR (50 MHz, $CDCl_3$) δ (ppm) 14.2 (COOCH$_2$$CH_3$), 41.5 (NH$CH_2$COO), 46.1 (N$CH_2$COO), 55.4 (O$CH_3$), 61.6 (COO$CH_2CH_3$), 67.3 (COO$CH_2$Ph), 105.8 ($C_3$), 109.6 ($C_7$), 114.1 ($C_{3'}$, $C_{5'}$), 121.2 (C_5), 122.4 (C_4), 124.9 (C_6), 126.3 (C_{3a}), 127.3 ($C_{1'}$), 130.5 (C_2, $C_{2'}$, $C_{6'}$), 139.0 (C_{7a}), 160.0 ($C_{4'}$), 162.3 (*C*ONHCH$_2$), 169.3 (*C*OOCH$_2$CH$_3$), 170.0 (*C*OOCH$_2$Ph). Anal. Calcd for $C_{23}H_{24}N_2O_6$: C, 65.08; H, 5.70; N, 6.60. Found: C, 65.12; H, 5.80; N, 6.67.

REMARK

Stirring the reaction with NMM at room temperature for 43 h gave 41.3% yield for the cyclized product of and 11% for the non-cyclized **41a**. When the reaction was carried out under the same conditions (rt, 43h) in the presence of DIEA, the yield was even lower for the cyclized product **41** (26%) and correspondingly increased in favor of the non-cyclized **41a** (17%).

4-Methoxybenzyl 2-[8-fluoro-1,3-dioxo-3,4-dihydropyrazino[1,2-a]indol-2(1*H*)-yl]acetate (45)

The title compound was obtained from the carboxylic acid precursor **10** after reaction with EDCI·HCl, HOBt, DIEA and O-(4-methoxybenzyl)glycine hydrochloride. Stirring of the reaction mixture at 35 °C for 48 h under argon and subsequent work-up afforded a dark viscous residue which was chromatographed. The chromatographic column was eluted with a mobile phase of increasing polarity from CH_2Cl_2 100% to CH_2Cl_2 /AcOEt 100:3 to give the desired product **45** (35%) as a white crystalline solid.
Mp 173-175 °C (AcOEt/*n*-pentane), R_f = 0.72 (CH_2Cl_2/AcOEt 10:1).
^{1}H NMR (400 MHz, $CDCl_3$) δ (ppm) 3.81 (s, 3H, OCH_3), 4.78 (s, 2H, NCH_2COO), 5.08 (s, 2H, H_4), 5.14 (s, 2H, COOCH_2Ph), 6.89 (d, 2H, J=8.6 Hz, $H_{3'}$, $H_{5'}$), 7.21 (td, 1H, J_1=8.9 Hz, J_2=2.4 Hz, H_7), 7.25-7.33 (complex m, 3H, H_6, $H_{2'}$, $H_{6'}$), 7.41 (dd, 1H, J_1=9.1 Hz, J_2=2.4 Hz, H_9), 7.44 (s, 1H, H_{10}); ^{13}C NMR (50 MHz, $CDCl_3$) δ (ppm) 40.7 (NCH$_2$COO), 46.5 (C_4), 55.4 (OCH$_3$), 67.6 (COOCH$_2$Ph), 107.7, 108.2 (d, $J_{C\text{-}F}$=23.7 Hz, C_9), 108.9, 109.0 (d, $J_{C\text{-}F}$=5.7 Hz, C_{10}), 111.0, 111.2 (d, $J_{C\text{-}F}$=9.6 Hz, C_6), 114.2 ($C_{3'}$, $C_{5'}$), 115.6, 116.2 (d, $J_{C\text{-}F}$=27.4 Hz, C_7), 125.5 (C_{10a}), 127.3 ($C_{1'}$), 127.5, 127.7 (d, $J_{C\text{-}F}$=8.5 Hz, C_{9a}), 130.3 ($C_{2'}$, $C_{6'}$), 133.9 (C_{5a}), 156.6, 161.3 (d, $J_{C\text{-}F}$=239.3 Hz, C_8), 157.6 (C_1=O), 160.0 ($C_{4'}$), 165.8 (C_3=O), 167.4 (COOCH$_2$Ph). Anal. Calcd for $C_{21}H_{17}FN_2O_5$: C, 63.63; H, 4.32; N, 7.07. Found: C, 63.55; H, 4.38; N, 7.11.

4-Methoxybenzyl 2-[8-chloro-1,3-dioxo-3,4-dihydropyrazino[1,2-a]indol-2(1*H*)-yl]acetate (49)
Following the general procedure described above, treatment of the corresponding carboxylic acid **28** with DIEA and O-(4-methoxybenzyl)glycine hydrochloride, and after stirring of the reaction mixture at 35 °C for 48 h under argon, the desired product was prepared. The dark residue resulting from the work-up was purified by flash column chromatography, eluting isocratically with CH_2Cl_2. Compound **49** was obtained as a white crystalline solid (15%). Mp 189-191 °C (AcOEt/*n*-pentane), R_f = 0.69 (CH_2Cl_2/AcOEt 10:1).
^{1}H NMR (400 MHz, $CDCl_3$) δ (ppm) 3.74 (s, 3H, OCH_3), 4.71 (s, 2H, NCH_2COO), 5.01 (s, 2H, H_4), 5.07 (s, 2H, COOCH_2Ph), 6.82 (d, 2H, J=8.6 Hz, $H_{3'}$, $H_{5'}$), 7.20 (d, 1H, J=9.4 Hz, H_6) overlapped with 7.22 (d, 2H, J=8.7 Hz, $H_{2'}$, $H_{6'}$), 7.32 (dd, 1H, J_1=8.8 Hz, J_2=2.0 Hz, H_7), 7.34 (s, 1H, H_{10}), 7.68 (d, 1H, J=1.9 Hz, H_9); ^{13}C NMR (50 MHz, $CDCl_3$) δ (ppm) 40.8 (NCH$_2$COO), 46.5 (C_4), 55.5 (OCH$_3$), 67.6 (COOCH$_2$Ph), 108.6 (C_{10}), 111.2 (C_6), 114.2 ($C_{3'}$, $C_{5'}$), 122.9 (C_9), 125.3 (C_{10a}), 127.2 (C_7), 127.3 ($C_{1'}$), 128.1 (C_8 or C_{9a}), 128.2 (C_8 or C_{9a}), 130.4 ($C_{2'}$, $C_{6'}$), 135.5 (C_{5a}), 157.5 (C_1=O), 160.0 ($C_{4'}$), 165.7 (C_3=O), 167.4 (COOCH$_2$Ph). Anal. Calcd for $C_{21}H_{17}ClN_2O_5$: C, 61.10; H, 4.15; N, 6.79. Found: C, 61.20; H, 4.19; N, 6.85.

General method for the synthesis of carboxylic acids 42, 46, 50 using TFA
1 mmol of the 4-methoxybenzyl ester (**41**, **45**, **49**), obtained from the previous step, was dissolved in 20 mL of dry CH_2Cl_2 and stirred at room temperature under argon. An excess of anisole (40 mmol) was added and then TFA (54 mmol) was injected dropwise over a period of 2-3 minutes. Stirring of the reaction mixture was continued while the progress of the reaction was monitored by normal phase TLC. After 3-4 hours and after the reaction was complete, the solvents and by-products were removed under reduced pressure. Distilled water was added to the heterogeneous residue of anisole, in a 1:1 volume ratio, to create an azeotropic mixture, and this concentrated to dryness. The resulting solid residue was triturated with *n*-pentane/ Et_2O 5:1 whereby the respective carboxylic acid was obtained pure.

2-[1,3-dioxo-3,4-dihydropyrazino[1,2-a]indol-2(1*H*)-yl]acetic acid (42)

Precursor molecule **41** was treated with TFA as described in the general methodology. The resulting off-white solid residue was triturated with *n*-pentane/Et_2O (5:1) to afford the pure carboxylic acid as a white crystalline solid (73%). Mp >250 °C (AcOEt/*n*-pentane), R_f = 0.16 (AcOEt).
^{1}H NMR (600 MHz, DMSO-d_6) δ (ppm) 3.90 (d, 2H, *J*=6.0 Hz, CH_2COOH), 5.31 (s, 2H, H_4), 7.12 (td, 1H, J_1=7.9 Hz, J_2=0.9 Hz, H_8), 7.25 (s, 1H, H_{10}) overlapped with 7.27 (td, 1H, J_1=8.3 Hz, J_2=1.2 Hz, H_7), 7.53 (d, 1H, *J*=8.5 Hz, H_6), 7.67 (d, 1H, *J*=7.9 Hz, H_9), 8.86 (t, 1H, *J*=6.0 Hz, COO*H*); ^{13}C NMR (50 MHz, DMSO-d_6) δ (ppm) 40.7 (*C*H_2COOH), 45.9 (C_4), 105.3 (C_{10}), 110.5 (C_6), 120.4 (C_8), 121.7 (C_9), 123.9 (C_7), 125.6 (C_{9a}), 130.7 (C_{10a}), 138.6 (C_{5a}), 161.9 (*C*OOH), 170.3 (C_3=O), 171.2 (C_1=O). Anal. Calcd for $C_{13}H_{10}N_2O_4$: C, 60.47; H, 3.90; N, 10.85. Found: C, 60.43; H, 3.98; N, 10.91.

2-[8-Fluoro-1,3-dioxo-3,4-dihydropyrazino[1,2-a]indol-2(1*H*)-yl]acetic acid (46)

A solution of precursor **45** in dry CH_2Cl_2 was treated first with anisole and then with TFA as described in the general methodology. The off-white solid resulting after azeotropic removal of the solvents was triturated with *n*-pentane/Et_2O (5:1). Compound **46** was obtained as a white crystalline solid (80%). Mp >250 °C (MeOH/Et_2O), R_f = 0.14 (CH_2Cl_2/MeOH 9:1).
^{1}H NMR (400 MHz, DMSO-d_6) δ (ppm) 4.56 (s, 2H, CH_2COOH), 5.41 (s, 2H, H_4), 7.32 (td, 1H, J_1=9.1 Hz, J_2=1.8 Hz, H_7), 7.38 (s, 1H, H_{10}), 7.57 (dd, 1H, J_1=9.3 Hz, J_2=1.8 Hz, H_9), 7.66 (dd, 1H, J_1=8.9 Hz, J_2=4.3 Hz, H_6), 13.12 (brs, 1H, COO*H*); ^{13}C NMR (50 MHz, DMSO-d_6) δ (ppm) 40.2 (*C*H_2COOH), 46.5 (C_4), 106.60, 106.64 (d, J_{C-F}=2.3 Hz, C_{10}), 106.8, 107.1 (d, J_{C-F}=15.8 Hz, C_9), 112.9, 113.1 (d, J_{C-F}=9.8 Hz, C_6), 114.3, 114.8 (d, J_{C-F}=27.1 Hz, C_7), 125.8 (C_{10a}), 126.5, 126.8 (d, J_{C-F}=10.9 Hz, C_{9a}), 133.5 (C_{5a}), 155.6, 160.3 (d, J_{C-F}=235.7 Hz, C_8), 157.6 (C_1=O), 166.6 (C_3=O), 168.8 (*C*OOH). Anal. Calcd for $C_{13}H_9FN_2O_4$: C, 56.53; H, 3.28; N, 10.14. Found: C, 56.62; H, 3.34; N, 10.19.

2-[8-chloro-1,3-dioxo-3,4-dihydropyrazino[1,2-a]indol-2(1H)-yl]acetic acid (50)

The preparation of carboxylic acid **50** from its precursor **49** was carried out as described in the general methodology. Workup yielded an off-white solid residue which was then triturated with *n*-pentane/Et_2O (5:1) to afford the pure carboxylic acid as a white leptocrystalline solid (almost quantitative yield). Mp >250 °C (MeOH/Et_2O), R_f = 0.11 (AcOEt).
^{1}H NMR (400 MHz, DMSO-d_6) δ (ppm) 4.52 (s, 2H, CH_2COOH), 5.40 (s, 2H, H_4), 7.37 (s, 1H, H_{10}), 7.44 (dd, 1H, J_1=8.9 Hz, J_2=1.7 Hz, H_7), 7.66 (d, 1H, J_1=9.0 Hz, J_2=1.2 Hz, H_6), 7.87 (d, 1H, *J*=1.6 Hz, H_9); ^{13}C NMR (150 MHz, DMSO-d_6) δ (ppm) 40.1 (*C*H_2COOH), 46.5 (C_4), 106.3 (C_{10}), 113.3 (C_6), 121.8 (C_9), 125.6 (C_7), 125.7 (C_{10a}), 126.1 (C_8), 127.4 (C_{9a}), 135.1 (C_{5a}), 157.6 (C_1=O), 166.5 (C_3=O), 168.8 (CH_2*C*OOH). Anal. Calcd for $C_{13}H_9ClN_2O_4$: C, 53.35; H, 3.10; N, 9.57. Found: C, 53.30; H, 3.12; N, 9.52.

General method of synthesis of acetamides 43, 47, 51

1 mmol of the carboxylic acid obtained from the previous step (**42**, **46**, **50**) was dissolved in a mixture of anhydrous CH_2Cl_2/DMF (4:1, 15 mL) and stirred under argon. EDCI·HCl (1.19 mmol), HOBt (1.2 mmol, hydrate 15% wt) and DIEA or NMM (1.5 mmol) were added. Then, O-(4-methoxybenzyl)hydroxylamine (1.2 mmol) dissolved in 1.5 mL dry CH_2Cl_2 was added. The reaction mixture was heated to 35 °C and stirred for 48-50 h under argon. The CH_2Cl_2 was then evaporated under reduced pressure and the heterogeneous residue was poured into ice/water (25 mL). the mixture was then extracted with AcOEt (3x30 mL) and the combined organic phases were washed with water (3x20 mL), 10% Na_2CO_3 aqueous solution (2x20 mL) and saturated NaCl aqueous solution (2x20 mL). The organic layer was separated and dried with anhydrous Na_2SO_4 and then concentrated *in vacuo*. The resulting residue was chromatographed on a silica gel column.

***N*-[(4-methoxybenzyl)oxy]-2-[1,3-dioxo-3,4-dihydropyrazino[1,2-*a*]indol-2(1*H*)-yl] acetamide (43)**

To prepare acetamide **43**, the corresponding carboxylic acid **42** was treated with EDCI·HCl, HOBt, NMM and O-(4-methoxybenzyl)hydroxylamine, following the general procedure described. The resulting residue after work-up was purified by flash column chromatography using initially *n*-hexane/AcOEt 1:1, and then 100% AcOEt to obtain the desired product as white crystalline solid (17%). Mp 192-194 °C (AcOEt/Et_2O, *n*-pentane), R_f = 0.26 (*n*-hexane/AcOEt 1:1).
^{1}H NMR (600 MHz, Acetone-d_6) δ (ppm) 3.80 (s, 3H, OCH_3), 4.61, 4.83 (2s, 2H, NCH_2CO, E/Z isomers respectively, 3/1), 4.80, 4.99 (2s, 2H, CONHOCH_2Ph, E/Z isomers respectively, 3/1), 5.30 (s, 2H, H_4), 6.86-7.00 (complex m, 2H, $H_{3'}$, $H_{5'}$), 7.24 (td, 1H, J_1=8.0 Hz, J_2=0.8 Hz, H_8), 7.32-7.39 (complex m, 2H, $H_{2'}$, $H_{6'}$), 7.41 (s, 1H, H_{10}), 7.44 (td, 1H, J_1=8.2 Hz, J_2=1.1 Hz, H_7), 7.61 (dd, 1H, J_1=8.4 Hz, J_2=0.9 Hz, H_6), 7.80 (d, 1H, J=7.8 Hz, H_9), 10.42 (s, 1H, N*H*); ^{13}C NMR (50 MHz, Acetone-d_6) δ (ppm) 40.6 (N*C*H_2CO), 47.2 (C_4), 55.5 (O*C*H_3), 78.1 (CONHO*C*H_2Ph), 108.0 (C_{10}), 111.6 (C_6), 114.5 ($C_{3'}$, $C_{5'}$), 122.5 (C_8), 123.7 (C_9), 125.9 (C_{10a}), 126.5 (C_7), 127.3 ($C_{1'}$), 128.0 (C_{9a}), 131.9 ($C_{2'}$, $C_{6'}$), 138.0 (C_{5a}), 158.9 (C_1=O), 160.8 ($C_{4'}$), 164.8 (*C*ONHOCH_2Ph), 167.5 (C_3=O). Anal. Calcd for $C_{21}H_{19}N_3O_5$: C, 64.12; H, 4.87; N, 10.68. Found: C, 64.19; H, 4.93; N, 10.71.

N-[(4-Methoxybenzyl)oxy]-2-[8-fluoro-1,3-dioxo-3,4-dihydropyrazino[1,2-*a*]indol-2(1*H*)-yl]acetamide (47)
A solution of carboxylic acid **46** in CH_2Cl_2/DMF, was treated with EDCI·HCl, HOBt, NMM and O-(4-methoxybenzyl)hydroxylamine and stirred at 35 °C for 50 h under argon. Subsequent extraction and removal of the organic solvents, as described in the general methodology, yielded a solid residue which was purified by flash column chromatography on silica gel. Initially CH_2Cl_2 was used as eluent, while the polarity was then increased to CH_2Cl_2/MeOH 100:0.5 to obtain a white crystalline solid which was identified by NMR experiments as the desired amide (75%). Mp 233-235 °C dec (AcOEt/Et_2O, *n*-pentane), R_f = 0.51 (CH_2Cl_2/MeOH 10:0.5).
^{1}H NMR (600 MHz, DMSO-d_6) δ (ppm) 3.75 (s, 3H, OCH_3), 4.43, 4.65 (2s, 2H, NCH_2CO, E/Z isomers respectively, 7/1), 4.70, 4.83 (2s, 2H, CONHOCH_2Ph, E/Z isomers respectively, 7/1), 5.34, 5.38 (2s, 2H, H_4, E/Z isomers respectively, 7/1), 6.93 (d, 2H, J=8.2 Hz, $H_{3'}$, $H_{5'}$), 7.29-7.35 (complex m, 3H, H_7, $H_{2'}$, $H_{6'}$), 7.38 (s, 1H, H_{10}), 7.57 (dd, 1H, J_1=9.6 Hz, J_2=2.5 Hz, H_9), 7.68 (dd, 1H, J_1=9.2 Hz, J_2=4.4 Hz, H_6), 10.98, 11.31 (2s, 1H, N*H*, Z/E isomers respectively, 1/7); ^{13}C NMR (50 MHz, DMSO-d_6) δ (ppm) 39.6 (N*C*H_2CO, Z isomer), 39.7(N*C*H_2CO, E isomer), 46.8 (C_4, E/Z isomers overlapped), 55.1 (O*C*H_3), 76.7 (CONHO*C*H_2Ph, E isomer), 78.3 (CONHO*C*H_2Ph, Z isomer), 106.4, 106.5 (d, $J_{C\text{-}F}$=2.3 Hz, C_{10}), 106.6, 107.1 (d, $J_{C\text{-}F}$=24.8 Hz, C_9), 112.9, 113.1 (d, $J_{C\text{-}F}$=8.9 Hz, C_6), 113.7 ($C_{3'}$, $C_{5'}$), 114.2, 114.8 (d, $J_{C\text{-}F}$=27.3 Hz, C_7), 126.2 (C_{10a}), 126.5, 126.8 (d, $J_{C\text{-}F}$=11.3 Hz, C_{9a}), 127.7 ($C_{1'}$), 130.8 ($C_{2'}$, $C_{6'}$), 133.4 (C_{5a}), 155.6, 160.3 (d, $J_{C\text{-}F}$=235.9 Hz, C_8), 157.9 (C_1=O), 159.4 ($C_{4'}$), 163.6 (*C*ONHOCH_2Ph), 166.8 (C_3=O). Anal. Calcd for $C_{21}H_{18}FN_3O_5$: C, 61.31; H, 4.41; N, 10.21. Found: C, 61.29; H, 4.43; N, 10.23.

N-[(4-Methoxybenzyl)oxy]-2-[8-chloro-1,3-dioxo-3,4-dihydropyrazino[1,2-a]indol-2(1H)-yl]acetamide (51)
The title compound **51** was prepared by reaction of carboxylic acid **50** with DIEA and O-(4-methoxybenzyl)hydroxylamine, according to the general amidation procedure described. After workup, the obtained off-white solid residue was chromatographed on a silica gel column and the desired amide was eluted with a mobile phase of increasing polarity from CH_2Cl_2 100% to CH_2Cl_2 /MeOH 100:0.5. After the purification a white crystalline solid was isolated (80%). Mp 221-223 °C dec (AcOEt/Et2O, n-pentane), Rf = 0.61 (CH_2Cl_2 /MeOH 10:0.5).
^{1}H NMR (400 MHz, DMSO-d_6) δ (ppm) 3.75 (s, 3H, OCH_3), 4.42, 4.65 (2s, 2H, NCH_2CO, E/Z isomers respectively, 6/1), 4.69, 4.83 (2s, 2H, CONHOCH_2Ph, E/Z isomers respectively, 6/1), 5.34, 5.39 (2s, 2H, H_4, E/Z isomers respectively, 6/1), 6.93 (d, 2H, J=8.2 Hz, $H_{3'}$, $H_{5'}$), 7.32 (d, 2H, J=8.2 Hz, $H_{2'}$, $H_{6'}$), 7.38 (s, 1H, H_{10}), 7.44 (dd, 1H, J_1=8.9 Hz, J_2=2.0 Hz, H_7), 7.68 (d, 1H, J=8.9 Hz, H_6), 7.87 (d, 1H, J=1.3 Hz, H_9), 11.02, 11.34 (2s, 1H, N*H*, Z/E isomers respectively, 1/6); ^{13}C NMR (50 MHz, DMSO-d_6) δ (ppm) 39.4 (N*C*H_2CO, Z isomer), 39.8 (N*C*H_2CO, E isomer), 46.7 (C_4, E/Z isomers overlapped), 55.1 (O*C*H_3), 76.7 (CONHO*C*H_2Ph, E isomer), 78.2 (CONHO*C*H_2Ph, Z isomer), 106.0 (C_{10}), 113.2 (C_6), 113.7 ($C_{3'}$, $C_{5'}$), 121.8

(C_9), 125.5 (C_7), 126.0 (C_{9a}, C_{10a}), 127.4 (C_8), 127.6 ($C_{1'}$), 130.8 ($C_{2'}$, $C_{6'}$), 134.9 (C_{5a}), 157.9 (C_1=O), 159.4 ($C_{4'}$), 163.6 (*C*ONHOCH$_2$Ph), 166.7 (C_3=O). Anal. Calcd for $C_{21}H_{18}ClN_3O_5$: C, 58.95; H, 4.24; N, 9.82. Found: C, 58.99; H, 4.33; N, 9.90.

General method for the synthesis of acetohydroxamic acids 44, 48, 52

To a stirred suspension of 1 mmol of the amide obtained from the previous step (**43**, **47**, **51**), in dry CH_2Cl_2 (30 mL), an excess of anisole (40 mmol) was added and the system was stirred at room temperature under argon. This was followed by the dropwise addition of TFA (54 mmol) over 2-3 minutes and stirring was continued for 3-4 hours under the same conditions. Completion of the reaction was controlled by TLC. Then, the volatile solvents and by-products were removed under vacuum and distilled water was then added to the residue, in a 1:1 volume ratio to create an azeotropic mixture with the remaining anisole. Evaporation under vacuum was continued, and the solid residue resulting after complete removal of the solvents was chromatographed over silica gel.

N-hydroxy-2-[1,3-dioxo-3,4-dihydropyrazino[1,2-*a*]indol-2(1*H*)-yl]acetamide (44)

The final acetohydroxamic acid **44** was prepared by the reaction of TFA on the amide **43** in a manner similar to that described in the general method. The solid residue resulting after removal of the solvents was chromatographed on a silica gel column under pressure. The polarity of the mobile phase was increased gradually from *n*-hexane/AcOEt 5:1 to AcOEt 100% and finally to AcOEt/MeOH 70:30 to quantitatively obtain the final product as a pale yellow crystalline solid (93%). Mp 160-162 °C dec (AcOEt, MeOH/*n*-pentane, Et_2O), R_f = 0.45 (AcOEt).
^{1}H NMR (600 MHz, DMSO-d_6) δ (ppm) 4.42, 4.74 (2s, 2H, NCH_2CO, E/Z isomers respectively, 5/1), 5.30, 5.38 (2s, 2H, H_4, E/Z isomers respectively, 5/1), 7.22 (t, 1H, J=7.9 Hz, H_8), 7.39 (s, 1H, H_{10}), 7.42 (td, 1H, J_1=8.1 Hz, J_2=0.9 Hz, H_7), 7.62 (d, 1H, J=8.1 Hz, H_6), 7.79 (d, 1H, J=8.1 Hz, H_9), 8.92, 9.44 (2s, 1H, CONHO*H*, E/Z isomers respectively, 5/1), 10.33, 10.74 (2s, 1H, CON*H*OH, Z/E isomers respectively, 1/5); ^{13}C NMR (50 MHz, DMSO-d_6) δ (ppm) 40.8 (N*C*H$_2$CO, E/Z isomers overlapped), 46.3 (C_4, Z isomer), 46.6 (C_4, E isomer), 106.5 (C_{10}), 111.3 (C_6), 121.5 (C_8), 122.7 (C_9), 124.8 (C_{10a}), 125.4 (C_7), 126.5 (C_{9a}), 136.5 (C_{5a}), 158.1 (C_1=O), 163.3 (*C*ONHOH, E isomer), 167.0 (C_3=O), 168.9 (*C*ONHOH, Z isomer). Anal. Calcd for $C_{13}H_{11}N_3O_4$: C, 57.14; H, 4.06; N, 15.38. Found: C, 57.19; H, 4.11; N, 15.40.

N-hydroxy-2-[8-fluoro-1,3-dioxo-3,4-dihydropyrazino[1,2-*a*]indol-2(1*H*)-yl]- acetamide (48)

The corresponding acetohydroxamic acid **48** was prepared by reaction of TFA with the acetamide precursor **47**, in the presence of anisole as described above. Column chromatography on silica gel starting with 100% AcOEt as eluent, the polarity of which was then increased to 70:30 AcOEt/MeOH, afforded **48** (almost quantitatively) as a pale yellow crystalline solid. Mp 172-174 °C dec (AcOEt, MeOH/*n*-pentane, Et_2O), R_f = 0.20 (AcOEt).
^{1}H NMR (600 MHz, DMSO-d_6) δ (ppm) 4.41, 4.73 (2s, 2H, NCH_2CO, E/Z isomers respectively, 4/1), 5.32, 5.39 (2s, 2H, H_4, E/Z isomers respectively, 4/1), 7.31 (td, 1H, J_1=9.2 Hz, J_2=2.4 Hz, H_7), 7.36 (s, 1H, H_{10}), 7.56 (dd, 1H, J_1=9.5 Hz, J_2=2.4 Hz, H_9), 7.67 (dd, 1H, J_1=9.0 Hz, J_2=4.3 Hz, H_6), 8.91, 9.42 (2s, 1H, CONHO*H*, E/Z isomers respectively, 4/1), 10.32, 10.73 (2s, 1H, CON*H*OH, Z/E isomers respectively, 1/4); ^{13}C NMR (50 MHz, DMSO-d_6) δ (ppm) 39.8 (N*C*H$_2$CO, Z isomer), 39.9 (N*C*H$_2$CO, E isomer), 46.5 (C_4, Z isomer), 46.8 (C_4, E isomer), 106.2, 106.3 (d, $J_{C\text{-}F}$=5.0 Hz, C_{10}), 106.6, 107.1 (d, $J_{C\text{-}F}$=23.8 Hz, C_9), 112.8, 113.0 (d, $J_{C\text{-}F}$=9.4 Hz, C_6), 114.1, 114.7 (d, $J_{C\text{-}F}$=27.0 Hz, C_7), 126.3 (C_{10a}), 126.5, 126.7 (d, $J_{C\text{-}F}$=11.5 Hz, C_{9a}), 133.3 (C_{5a}), 155.6, 160.2 (d, $J_{C\text{-}F}$=235.2 Hz, C_8), 157.9 (C_1=O), 163.3 (*C*ONHOH, E isomer), 166.8 (C_3=O), 168.7 (*C*ONHOH, Z isomer). Anal. Calcd for $C_{13}H_{10}FN_3O_4$: C, 53.61; H, 3.46; N, 14.43. Found: C, 53.67; H, 3.44; N, 14.49.

N-Hydroxy-2-[8-chloro-1,3-dioxo-3,4-dihydropyrazino[1,2-a]indol-2(1*H*)-yl]-acetamide (52)
A solution of acetamide **51** was treated first with anisole and then with TFA as described in the general method. The solid residue obtained, after stirring for 3 hours at room temperature and subsequent removal of the solvents, was purified by preparative thin layer chromatography. The chromatographic plates were developed in a mobile phase of AcOEt/MeOH 9:1 and then the band with R_f = 0.61, which corresponded to the desired product, was scraped and extracted with dry THF and with the help of ultrasounds. The silica was vacuum filtered and the filtrate was collected and concentrated to dryness. The resulting solid product was triturated with *n*-pentane/Et_2O (5:1) to finally give the desired acetohydroxamic acid as an off-white crystalline solid (93%). Mp 162-164 °C dec (AcOEt, MeOH/*n*-pentane, Et_2O), R_f = 0.4 (AcOEt).
^1H NMR (600 MHz, DMSO-d_6) δ (ppm) 4.41, 4.73 (2s, 2H, NCH_2CO, E/Z isomers respectively, 5/1), 5.32, 5.39 (2s, 2H, H_4, E/Z isomers respectively, 5/1), 7.35 (s, 1H, H_{10}), 7.43 (dd, 1H, J_1=8.9 Hz, J_2=2.0 Hz, H_7), 7.67 (d, 1H, J=8.8 Hz, H_6), 7.86 (d, 1H, J=1.6 Hz, H_9), 8.91, 9.42 (2s, 1H, CONHO*H*, E/Z isomers respectively, 5/1), 10.33, 10.73 (2s, 1H, CON*H*OH, Z/E isomers respectively, 1/5); ^{13}C NMR (150 MHz, DMSO-d_6) δ (ppm) 39.4 (N*C*H_2CO, Z isomer), 40.1 (N*C*H_2CO, E isomer), 46.2 (C_4, Z isomer), 46.8 (C_4, E isomer), 105.9 (C_{10}), 113.1 (C_6), 121.7 (C_9), 125.5 (C_7), 126.0 (C_{10a}), 126.1 (C_8), 126.5 (C_{9a}), 134.9 (C_{5a}), 157.9 (C_1=O), 163.3 (*C*ONHOH, E isomer), 166.7 (C_3=O), 169.0 (*C*ONHOH, Z isomer). Anal. Calcd for $C_{13}H_{10}ClN_3O_4$: C, 50.75;H, 3.28; N, 13.66. Found: C, 50.81; H, 3.35; N, 13.70.

Synthetic route to pyrrole acetohydroxamic acid analogue 62

Benzyl 1H-pyrrole-2-carboxylate (54)
1H-pyrrole-2-carboxylic acid (**53**) (300 mg, 2.7 mmol) was suspended in 17.5 mL of dry CH_2Cl_2 and dissolved by adding a small amount of dry DMF (1 mL) while heating gently (~40 °C). Benzyl alcohol (365 mg, 3.38 mmol) was then added followed by the addition of a catalytic amount of DMAP (66 mg, 0.54 mmol) and DCC (560 mg, 2.7 mmol). The mixture was stirred at room temperature under argon atmosphere for 4 hours and then filtered through a Hirsch filter. The filtrate was concentrated *in vacuo*. The residue was poured into a mixture of ice/water (20 mL) and extracted with AcOEt (3x20 mL) while the combined organic layers were then washed with 1N HCl (2x4 mL), water (2x5 mL), saturated aqueous $NaHCO_3$ (2x7 mL) and saturated aqueous NaCl solution (2x7 mL). The organic layer was dried over anhydrous Na_2SO_4, filtered and concentrated under reduced pressure. The final mixture was subjected to silica gel column chromatography eluting isocratically with *n*-hexane/AcOEt 5:1. Target compound **54** (52%) was obtained as a colorless, clear, viscous oil which solidified over time at room temperature.
Mp 53-55 °C (Et_2O/*n*-pentane | Small acicular crystals), R_f= 0.54 (*n*-hexane/AcOEt 2:1).
^1H NMR (600 MHz, $CDCl_3$) δ (ppm) 5.32 (s, 2H, COOCH_2Ph), 6.27 (dt overlapping, 1H, J_1=3.7 Hz, J_2=2.5 Hz, H_4), 6.95 (td, 1H, J_1=2.7 Hz, J_2=1.5 Hz, H_5), 6.99 (ddd overlapping, 1H, J_1=3.8 Hz, J_2=2.4 Hz, J_3=1.5 Hz, H_3), 7.32-7.36 (m, 1H, $H_{4'}$), 7.36-7.41 (complex m, 2H, $H_{3'}$, $H_{5'}$), 7.43 (dd, 2H, J_1=7.3 Hz, J_2=1.5 Hz, $H_{2'}$, $H_{6'}$), 9.45 (brs, 1H, N*H*); ^{13}C NMR (50 MHz, $CDCl_3$) δ (ppm) 66.1 (COO*C*H_2Ph), 110.6 (C_4), 115.8 (C_3), 122.6 (C_2), 123.3 (C_5), 128.3 ($C_{3'}$, $C_{4'}$, $C_{5'}$), 128.7 ($C_{2'}$, $C_{6'}$), 136.3 ($C_{1'}$), 161.2 (*C*OOCH_2Ph). Anal. Calcd for $C_{12}H_{11}NO_2$: C, 71.63; H, 5.51; N, 6.96. Found: C, 71.72; H, 5.60; N, 6.99.

Benzyl 1-[(ethoxycarbonyl)methyl]-1*H*-pyrrole-2-carboxylate (55)
A stirred solution of the benzyl ester **54** obtained from the previous step (202 mg, 1.0 mmol) in 4 mL dry DMF, was cooled to 0 °C. The required amount of sodium hydride (44 mg, 1.1 mmol, 60% w/w dispersion in mineral oils) was then added to it portionwise and under continuous cooling. The reaction mixture was stirred under the same conditions until the evolution of hydrogen ceased and was then allowed to gradually

rise to ambient temperature and stirred for an additional 1 hour at 35 °C under argon. This was followed by the dropwise addition of ethyl bromoacetate (182 mg, 1.09 mmol) previously dissolved in 1 mL of dry DMF, and stirring was continued at 40 °C under an argon atmosphere for 4 days. After the end of the reaction, the excess sodium hydride was neutralized by pouring the mixture into ice/water (20 mL) followed by extraction with AcOEt (3x20 mL). The combined organic layers were washed with water (3x20 mL) and saturated aqueous NaCl (3x20 mL), dried over anhydrous Na_2SO_4, and evaporated under reduced pressure to afford a crude pale yellow oil. Column chromatography of this residue, eluting isocratically with n-hexane/AcOEt 6:1, afforded the desired diester **55** (91%) as a colorless, clear, viscous oil. R_f = 0.57 (*n*-hexane/AcOEt 2:1).

^{1}H NMR (600 MHz, $CDCl_3$) δ (ppm) 1.25 (t, 3H, *J*=7.1 Hz, $COOCH_2CH_3$), 4.20 (q, 2H, *J*=7.1 Hz, $COOCH_2CH_3$), 5.03 (s, 2H, NCH_2COO), 5.26 (s, 2H, $COOCH_2Ph$), 6.20 (dd, 1H, J_1=4.0 Hz, J_2=2.6 Hz, H_4), 6.83 (dd, 1H, J_1=2.5 Hz, J_2=1.9 Hz, H_5), 7.06 (dd, 1H, J_1=4.0 Hz, J_2=1.8 Hz, H_3), 7.29-7.34 (m, 1H, $H_{4'}$), 7.34-7.38 (complex m, 2H, $H_{3'}$, $H_{5'}$), 7.38-7.43 (complex m, 2H, $H_{2'}$, $H_{6'}$); ^{13}C NMR (50 MHz, $CDCl_3$) δ (ppm) 14.2 ($COOCH_2CH_3$), 50.7 (NCH_2COO), 61.6 ($COOCH_2CH_3$), 65.7 ($COOCH_2Ph$), 108.9 (C_4), 118.7 (C_3), 122.4 (C_2), 128.1 ($C_{2'}$, $C_{6'}$), 128.2 ($C_{4'}$), 128.6 ($C_{3'}$, $C_{5'}$), 129.8 (C_5), 136.4 ($C_{1'}$), 161.6 ($COOCH_2Ph$), 168.8 ($COOCH_2CH_3$). HRMS/ESI$^+$ (m/z): Calcd for $C_{16}H_{17}NO_4$: 287.1158; Found: 287.1155.

1-[(ethoxycarbonyl)methyl]-1*H*-pyrrole-2-carboxylic acid (56)

(195 mg, 0.68 mmol)of diester, were dissolved in a mixture of abs. EtOH/AcOEt (2:1, 15 mL) and transferred to a hydrogenation flask. A catalytic amount of Pd/C (10% w/w) was then added and the mixture was hydrogenated for 3 hours at 43-44 °C and 52-55 psi. After the reaction is complete, the catalyst was filtered through a porous bottom funnel (under vacuum), and washed with hot abs. EtOH (3x10 mL). Finally, the combined filtrates were concentrated *in vacuo* to afford the title carboxylic acid **56** (almost quantitative yield) as a white, fine crystalline solid. Mp 157-159 °C (AcOEt/*n*-pentane, Et_2O), R_f = 0.31 (AcOEt).

^{1}H NMR (600 MHz, $CDCl_3$) δ (ppm) 1.28 (t, 3H, *J*=7.1 Hz, $COOCH_2CH_3$), 4.23 (q, 2H, *J*=7.1 Hz, $COOCH_2CH_3$), 5.01 (s, 2H, NCH_2COO), 6.23 (dd, 1H, J_1=4.0 Hz, J_2=2.6 Hz, H_4), 6.87 (dd, 1H, J_1=2.5 Hz, J_2=1.7 Hz, H_5), 7.14 (dd, 1H, J_1=4.0 Hz, J_2=1.7 Hz, H_3); ^{13}C NMR (150 MHz, $CDCl_3$) δ (ppm) 14.3 ($COOCH_2CH_3$), 50.9 (NCH_2COO), 61.7 ($COOCH_2CH_3$), 109.3 (C_4), 120.5 (C_3), 121.7 (C_2), 130.8 (C_5), 165.6 ($COOH$), 168.7 ($COOCH_2CH_3$). Anal. Calcd for $C_9H_{11}NO_4$: C, 54.82; H, 5.62; N, 7.10. Found: C, 54.76; H, 5.68; N, 7.08.

2-(Benzyloxy)pyrrolo[1,2-a]pyrazine-1,3(2*H*,4*H*)-dione (57)

To a solution of carboxylic acid **56** (150 mg, 0.76 mmol) in a mixture of CH_2Cl_2 and anhydrous DMF (4:1, 8 mL), were added sequentially EDCI·HCl (173 mg, 0.90 mmol), HOBt (145 mg, 0.91 mmol, hydrate 15% wt), DIEA (344 mg, 2.66 mmol) and finally O-benzyl-hydroxylamine hydrochloride (145 mg, 0.91 mmol). The reaction mixture was heated to 35 °C, and stirred for 65 h under argon atmosphere. The reaction mixture was concentrated under reduced pressure, poured onto ice/water mixture (8 mL), and extracted with AcOEt (4x10 mL). The combined organic extracts were washed sequentially with water (3x10 mL), 10% aqueous Na_2CO_3 (2x10 mL), and saturated aqueous NaCl (2x10 mL). The organic layer is separated, dried with anhydrous Na_2SO_4, and concentrated *in vacuo*. The resulting solid residue was purified through flash column chromatography on silica gel using CH_2Cl_2 as eluent to afford the desired analog **57** as a white crystalline solid (49%). Mp 156-158 °C (AcOEt/*n*-pentane, Et_2O), R_f = 0.3 (CH_2Cl_2).

^{1}H NMR (600 MHz, $CDCl_3$) δ (ppm) 5.02 (s, 2H, H_4), 5.13 (s, 2H, OCH_2Ph), 6.42 (dd, 1H, J_1=4.0 Hz, J_2=2.6 Hz, H_7), 6.90 (dd, 1H, J_1=2.5 Hz, J_2=1.6 Hz, H_6), 7.15 (dd, 1H, J_1=4.0 Hz, J_2=1.5 Hz, H_8), 7.35-7.42 (complex m, 3H, $H_{3'}$, $H_{4'}$, $H_{5'}$), 7.56-7.60 (complex m, 2H, $H_{2'}$, $H_{6'}$); ^{13}C NMR (150 MHz, $CDCl_3$)

δ (ppm) 49.8 (*C4*), 79.1 (O*C*H$_2$Ph), 112.9 (*C7*), 116.7 (*C8*), 121.0 (*C8a*), 125.7 (*C6*), 128.6 (*C3′*, *C5′*), 129.4 (*C4′*), 130.2 (*C2′*, *C6′*), 133.8 (*C1′*), 154.0 (*C1*=O), 162.1 (*C3*=O). Anal. Calcd for $C_{14}H_{12}N_2O_3$: C, 65.62; H, 4.72; N, 10.93. Found: C, 65.71; H, 4.79; N, 10.99.

2-Hydroxypyrrolo[1,2-a]pyrazine-1,3(2*H*,4*H*)-dione (58)
N-(benzyloxy)-substituted diketopiperazine **57** (100 mg, 0.39 mmol) was dissolved in a hot mixture of abs. EtOH/AcOEt (2:1, 60 mL) and placed in a hydrogenation flask. Pd/C catalyst (10% w/w) was added and the mixture was hydrogenated for 3 hours at 43 °C and 52-55 psi. After the completion of the reaction, the catalyst was filtered off, through a sintered glass funnel, washed sufficiently with a mixture of hot abs. EtOH (3x10 mL). The combined filtrates were concentrated under reduced pressure to give a solid residue which was then chromatographed on a silica gel column using first AcOEt and then 10:1 AcOEt/MeOH as eluents to afford **58** (almost quantitative yield) as an off-white/beige crystalline solid. Mp 215-217 °C dec (EtOH/*n*-pentane, Et_2O), R_f = 0.2 (AcOEt).
^{1}H NMR (600 MHz, DMSO-*d6*) δ (ppm) 5.22 (s, 2H, *H4*), 6.36 (dd, 1H, J_1=4.0 Hz, J_2=2.5 Hz, *H7*), 6.92 (dd, 1H, J_1=3.9 Hz, J_2=1.6 Hz, *H8*), 7.17 (dd, 1H, J_1=2.3 Hz, J_2=1.9 Hz, *H6*), 10.38 (brs, 1H, N-O*H*); ^{13}C NMR (150 MHz, DMSO-*d6*) δ (ppm) 49.5 (*C4*), 111.2 (*C7*), 113.9 (*C8*), 120.7 (*C8a*), 126.1 (*C6*), 154.7 (*C1*=O), 163.2 (*C3*=O). Anal. Calcd for $C_7H_6N_2O_3$: C, 50.61; H, 3.64; N, 16.86. Found: C, 50.60; H, 3.68; N, 16.89.

Benzyl 2-[1,3-dioxo-3,4-dihydropyrrolo[1,2-a]pyrazin-2(1H)-yl]acetate (59) and
Benzyl 2-{[1-(2-ethoxy-2-oxoethyl)-1H-pyrrol-2-yl]formamido}acetate (59a)
Carboxylic acid **56** (175 mg, 0.89 mmol), the preparation of which was described previously, was dissolved in a mixture of anhydrous CH_2Cl_2 and anhydrous DMF (4:1, 10 mL) and stirred at room temperature under argon. EDCI·HCl (203 mg, 1.06 mmol), HOBt (170 mg, 1.07 mmol, hydrate 15% wt), NMM (360 mg, 3.56 mmol) and finally O-benzylglycine tosylate (361 mg, 1.07 mmol) were added to the solution. Stirring of the reaction mixture was continued for 68 hours under the same conditions and then the mixture was then evaporated *in vacuo*. The heterogeneous residue was poured into ice/water (20 mL) and extracted with AcOEt (3x30 mL). The combined organic extracts were washed sequentially with water (3x20 mL), 10% aqueous Na_2CO_3 (2x20 mL), and saturated aqueous NaCl (2x20 mL). The organic layer was dried over anhydrous Na_2SO_4 and concentrated *in vacuo*. The viscuous orange oily residue was purified with a silica gel column and using as eluent CH_2Cl_2 100% to afford the cyclized derivative **59** (28%) as a white crystalline solid. Further elution of the column with gradually increasing polarity of the mobile phase up to CH_2Cl_2 /AcOEt 10:1 afforded a colorless, viscuous oil product which solidified upon cooling and which by NMR experiments was identified as the non-cyclized product **59a** (19%).

Benzyl 2-[1,3-dioxo-3,4-dihydropyrrolo[1,2-a]pyrazin-2(1H)-yl]acetate (59)
Mp 132-134 °C (AcOEt/*n*-pentane), R_f = 0.63 (CH_2Cl_2/AcOEt 10:1).
^{1}H NMR (600 MHz, $CDCl_3$) δ (ppm) 4.75 (s, 2H, NC*H2*COO), 5.02 (s, 2H, *H4*), 5.20 (s, 2H, COOC*H2*Ph), 6.43 (dd, 1H, J_1=4.0 Hz, J_2=2.6 Hz, *H7*), 6.93 (dd, 1H, J_1=2.6 Hz, J_2=1.6 Hz, *H6*), 7.15 (dd, 1H, J_1=4.0 Hz, J_2=1.5 Hz, *H8*), 7.31-7.39 (complex m, 5H, *H2′*, *H3′*, *H4′*, *H5′*, *H6′*); ^{13}C NMR (50 MHz, $CDCl_3$) δ (ppm) 40.4 (N*C*H$_2$COO), 48.7 (*C4*), 67.6 (COO*C*H$_2$Ph), 112.6 (*C7*), 116.5 (*C8*), 120.5 (*C8a*), 125.5 (*C6*), 128.4 (*C2′*, *C6′*), 128.6 (*C4′*), 128.8 (*C3′*, *C5′*), 135.3 (*C1′*), 156.4 (*C1*=O), 166.0 (*C3*=O), 167.7 (*C*OOCH$_2$Ph). Anal. Calcd for $C_{16}H_{14}N_2O_4$: C, 64.42; H, 4.73; N, 9.39. Found: C, 64.39; H, 4.78; N, 9.44.

Benzyl 2-{[1-(2-ethoxy-2-oxoethyl)-1*H*-pyrrol-2-yl]formamido}acetate (59a)

Mp 92-94 °C (*n*-pentane | Off-white crystalline solid), R_f = 0.41 (CH_2Cl_2/AcOEt 10:1).
^1H NMR (400 MHz, $CDCl_3$) δ (ppm) 1.26 (t, 3H, *J*=7.1 Hz, $COOCH_2CH_3$), 4.15 (d, 2H, *J*=5.3 Hz, $NHCH_2COO$), 4.21 (q, 2H, J=7.1 Hz, $COOCH_2CH_3$), 5.05 (s, 2H, NCH_2COO), 5.19 (s, 2H, $COOCH_2Ph$), 6.17 (dd, 1H, J_1=3.9 Hz, J_2=2.7 Hz, H_4), 6.46 (brt, 1H, *J*=4.7 Hz, $CONHCH_2$), 6.69 (dd, 1H, J_1=4.0 Hz, J_2=1.6 Hz, H_3), 6.75 (dd, 1H, J_1=2.5 Hz, J_2=1.6 Hz, H_5), 7.29-7.41 (complex m, 5H, $H_{2'}$, $H_{3'}$, $H_{4'}$, $H_{5'}$, $H_{6'}$); ^{13}C NMR (50 MHz, $CDCl_3$) δ (ppm) 14.2 ($COOCH_2CH_3$), 41.3 ($NHCH_2COO$), 50.7 (NCH_2COO), 61.5 ($COOCH_2CH_3$), 67.3 ($COOCH_2Ph$), 108.3 (C_4), 112.9 (C_3), 124.8 (C_2), 128.4 (C_5), 128.5 ($C_{2'}$, $C_{6'}$), 128.6 ($C_{4'}$), 128.7 ($C_{3'}$, $C_{5'}$), 161.6 ($CONHCH_2$), 169.2 ($COOCH_2CH_3$), 170.1 ($COOCH_2Ph$). Anal. Calcd for $C_{18}H_{20}N_2O_5$: C, 62.78; H, 5.85; N, 8.13. Found: C, 62.67; H, 5.82; N, 8.18.

REMARK
Carrying out the reaction at 40 °C and stirring for nearly 85 h gave a 32% yield for the non-cyclized product **59a** , whereas the yield for the desired cyclized product **59** dropped to 22%.
ΠΑΡΑΤΗΡΗΣΗ

2-[1,3-dioxo-3,4-dihydropyrrolo[1,2-a]pyrazin-2(1*H*)-yl]acetic acid (60)
Benzylester **59** (150 mg, 0.50 mmol) was dissolved in a mixture of abs. EtOH/AcOEt (2:1, 50 mL) and brought into a hydrogenation flask along with a catalytic amount of Pd/C (10% w/w). The flask was saturated with H_2 gas and stirred for 3 hours at room temperature under pressure 52-55 psi. Upon completion of the reaction, the catalyst was filtered off under vacuum (sintered glass funnel) and washed with warm abs. EtOH (3x10 mL). The combined filtrates were concentrated under reduced pressure to give carboxylic acid **60** (almost quantitative yield) as a white crystalline solid which was used in the next step without further purification. Mp >250 °C dec (AcOEt/*n*-pentane), R_f = 0.1 (AcOEt).
^1H NMR (600 MHz, $CDCl_3$) δ (ppm) 4.47 (s, 2H, CH_2COOH), 5.24 (s, 2H, H_4), 6.40 (dd, 1H, J_1=4.0 Hz, J_2=2.5 Hz, H_7), 6.98 (dd, 1H, J_1=4.0 Hz, J_2=1.6 Hz, H_8), 7.23 (dd, 1H, J_1=2.4 Hz, J_2=1.6 Hz, H_6), 13.03 (brs, 1H, COO*H*); ^{13}C NMR (50 MHz, $CDCl_3$) δ (ppm) 40.8 (CH_2COOH), 48.5 (C_4), 111.4 (C_7), 114.5 (C_8), 119.8 (C_{8a}), 126.5 (C_6), 156.3 (C_1=O), 166.7 (C_3=O), 169.1 (*C*OOH). Anal. Calcd for $C_9H_8N_2O_4$: C, 51.93; H, 3.87; N, 13.46. Found: C, 51.96; H, 3.91; N, 13.47.

N-(benzyloxy)-2-[1,3-dioxo-3,4-dihydropyrrolo[1,2-a]pyrazin-2(1H)-yl]acetamide (61)
To a stirred solution of **60** (100 mg, 0.48 mmol) in a mixture of anhydrous CH_2Cl_2/dry DMF (4:1, 10 mL), EDCI·HCl (110 mg, 0.57 mmol), HOBt (92 mg, 0.58 mmol, hydrate 15% wt), NMM (146 mg, 1.44 mmol) and finally O-benzyl-hydroxylamine hydrochloride (93 mg, 0.58 mmol) were added. Stirring of the mixture was continued for 40 hours at room temperature and under argon atmosphere. The reaction mixture was evaporated *in vacuo* and the residue was first poured into ice/water (15 mL) and then extracted with AcOEt (4x15 mL). The organic phases were combined and washed with water (3x15 mL), 10% aqueous Na_2CO_3 (2x15 mL), and saturated aqueous NaCl (2x15 mL). The organic layer was dried over anhydrous Na_2SO_4, and evaporated to dryness. The resulting white solid residue was chromatographed on a silica gel column with increasing polarity eluents starting with *n*-hexane/AcOEt 5:1 to 100% AcOEt, to afford the desired amide **61** (15%) as a white crystalline solid. Mp 183-185 °C dec (AcOEt/*n*-pentane), R_f = 0.61 (AcOEt).
^1H NMR (600 MHz, Acetone-d_6) δ (ppm) 4.51 (2s, 2H, NCH_2CO), 4.86 (s, 2H, $CONHOCH_2Ph$), 5.18 (s, 2H, H_4), 6.39 (dd, 1H, J_1=4.0 Hz, J_2=2.5 Hz, H_7), 6.98 (dd, 1H, J_1=4.0 Hz, J_2=1.6 Hz, H_8), 7.18 (dd, 1H, J_1=2.5 Hz, J_2=1.6 Hz, H_6), 7.29-7.53 (complex m, 5H, $H_{2'}$, $H_{3'}$, $H_{4'}$, $H_{5'}$, $H_{6'}$), 10.45, 10.60 (2s, 1H, $CONHOCH_2Ph$); ^{13}C NMR (50 MHz, Acetone-d_6) δ (ppm) 40.2 (NCH_2CO), 49.3 (C_4), 78.4 ($CONHOCH_2Ph$), 112.1 (C_7), 115.3 (C_8), 121.6 (C_{8a}),

126.6 (C_6), 129.2 ($C_{2'}$, $C_{4'}$, $C_{6'}$), 130.1 ($C_{3'}$, $C_{5'}$), 136.9 ($C_{1'}$), 157.4 (C_1=O), 164.1 (*C*ONHOCH$_2$Ph), 167.4 (C_3=O). Anal. Calcd for $C_{16}H_{15}N_3O_4$: C, 61.34; H, 4.83; N, 13.41. Found: C, 61.39; H, 4.87; N, 13.44.

N-hydroxy-2-[1,3-dioxo-3,4-dihydropyrrolo[1,2-a]pyrazin-2(1*H*)-yl]acetamide (62)
A solution of the amide precursor **61** (50 mg, 0.16 mmol) in a mixture of abs. EtOH/AcOEt (2:1, 30 mL) was catalytically hydrogenated in the presence of Pd/C (10% w/w) for 3 h, at room temperature and under a pressure of 52-55 psi. Completion of the reaction was followed by vacuum filtration of the catalyst and washing of it with hot abs. EtOH (3x10 mL). The combined filtrates were evaporated to dryness under reduced pressure to yield the desired acetohydroxamic acid which was further purified chromatographically. It was eluted with gradually increased polarity eluents, starting initially with a mixture of CH_2Cl_2/AcOEt 5:1 and finishing with AcOEt 100%. An off-white crystalline solid was finally isolated (41%). Mp 193-195 °C (AcOEt, MeOH/*n*-pentane, Et$_2$O), R_f = 0.38 (AcOEt).
^{1}H NMR (600 MHz, DMSO-d_6) δ (ppm) 4.32, 4.64 (2s, 2H, NCH_2CO, E/Z isomers respectively, 4/1), 5.15, 5.22 (2s, 2H, H_4, E/Z isomers respectively, 4/1), 6.39 (dd, 1H, J_1=4.0 Hz, J_2=2.5 Hz, H_7), 6.95 (dd, 1H, J_1=3.9 Hz, J_2=1.6 Hz, H_8), 7.22 (dd, 1H, J_1=2.5 Hz, J_2=1.6 Hz, H_6), 8.86, 9.36 (2s, 1H, CONHO*H*, E/Z isomers respectively, 4/1), 10.26, 10.67 (2s, 1H, CON*H*OH, Z/E isomers respectively, 1/4); ^{13}C NMR (50 MHz, DMSO-d_6) δ (ppm) 39.4 (N*C*H$_2$CO, E/Z isomers overlapped), 48.4 (C_4, Z isomer), 48.7 (C_4, E isomer), 111.2 (C_7), 114.1 (C_8), 120.2 (C_{8a}), 126.1 (C_6), 156.6 (C_1=O), 163.6 (*C*ONHOH, E isomer), 166.9 (C_3=O), 169.2 (*C*ONHOH, Z isomer). Anal. Calcd for $C_9H_9N_3O_4$: C, 48.43; H, 4.06; N, 18.83. Found: C, 48.46; H, 4.10; N, 18.82.

III. *In-silico* studies - Calculations Methods
Predicted drug-likeness properties and ADME

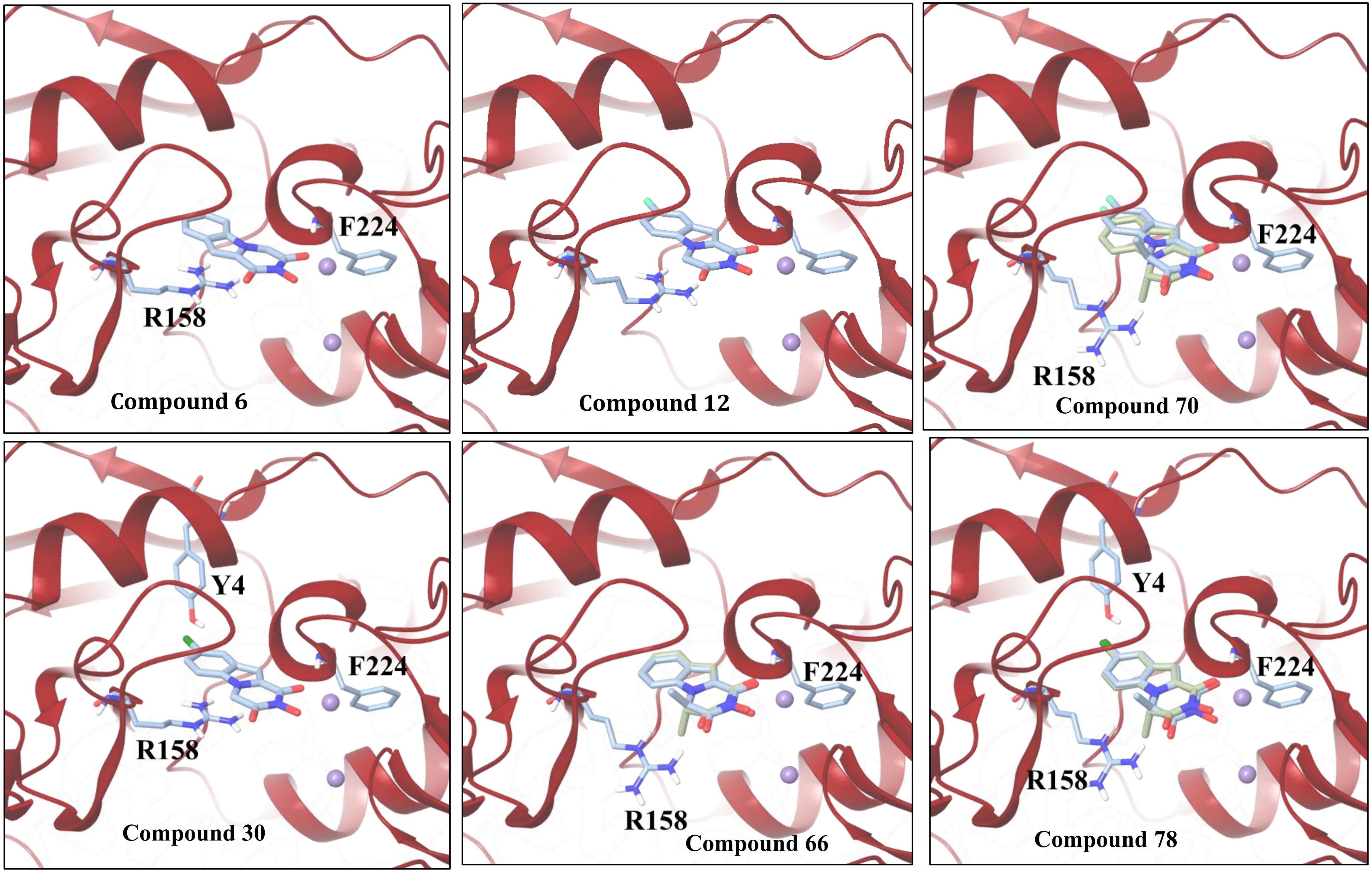

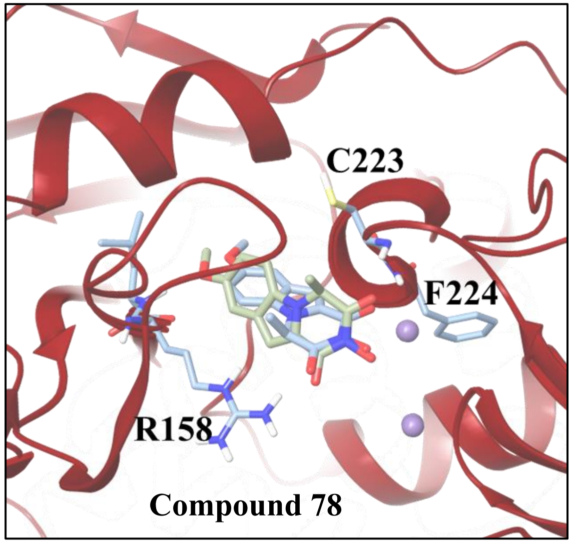

Figure S1: The experimental binding mode of the active compounds based on the lowest energy IFD result. Compound name is written on every figure. In cases where chirality could have been both R and S in specific atoms the ligands with S chirality are represented with green and the ligands with R with light blue.

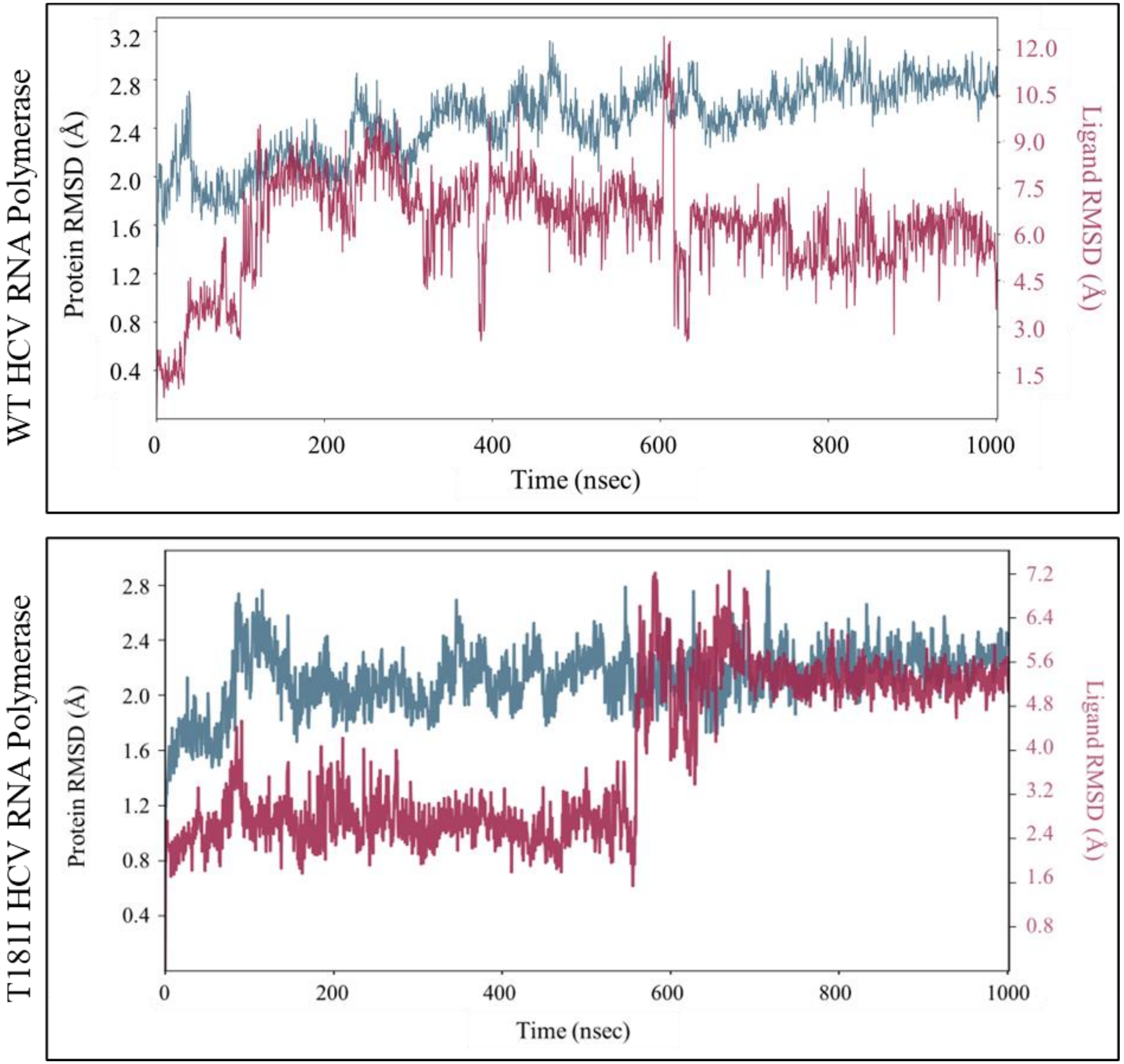

Figure S2: RMSD diagrams of both the Wild type and the T181I mutant HCV RNA polymerase in complex with **36** during the 1μs Molecular Dynamics Simulation.

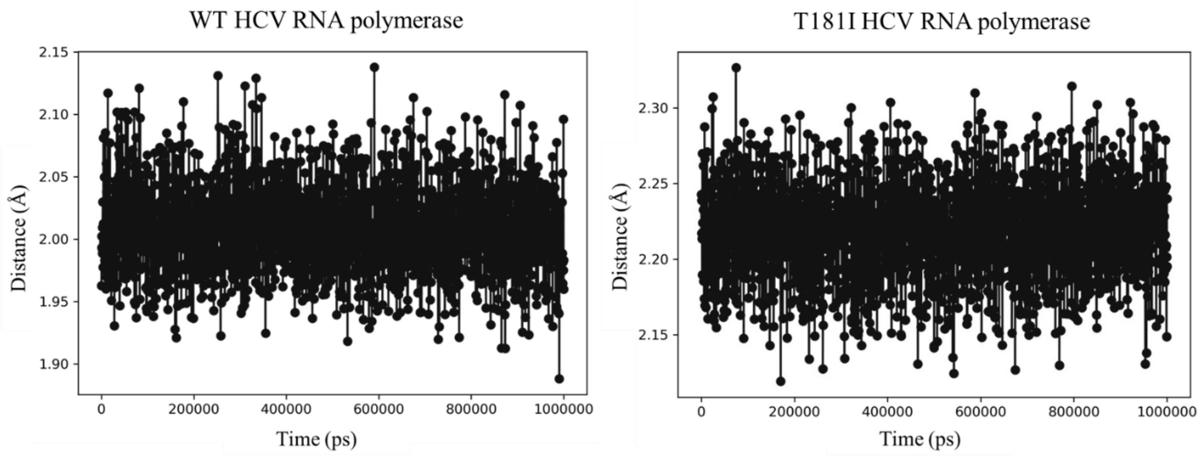

Figure S3: Distance between the Mn^{2+} ion and the O^{-} ion of both the Wild type and the T181I mutant HCV RNA polymerase in complex with **36** during the 1μs Molecular Dynamics Simulation

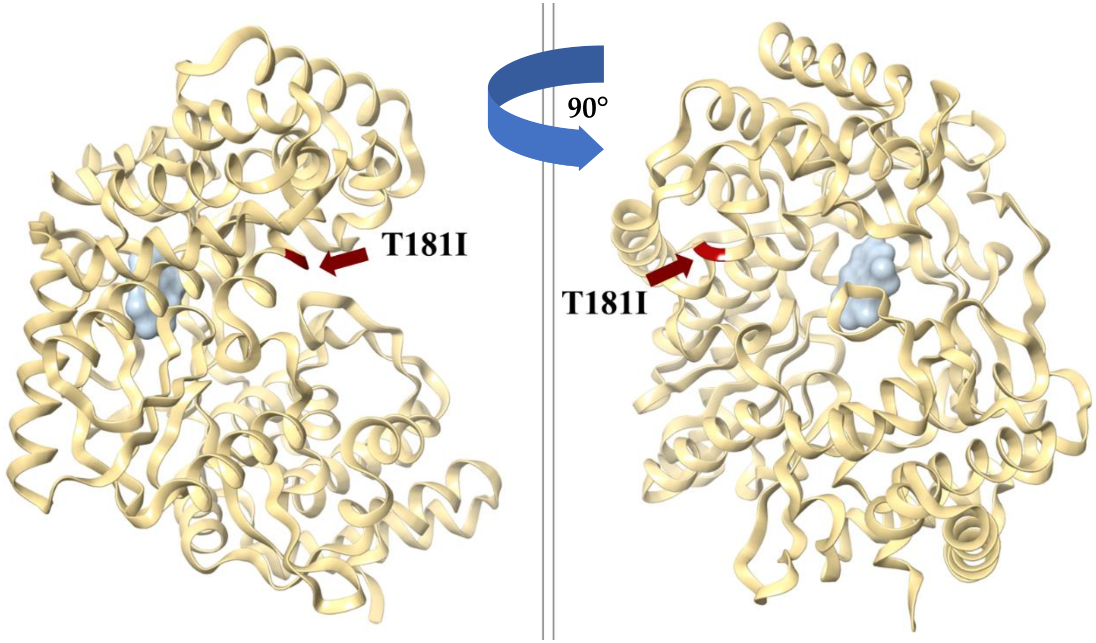

Figure S4: Position of mutation T181I in the HCV RNA polymerase structure. The protein is represented as a yellow ribbon, the bound ligand as a light blue surface and the T181I position with dark red color.

Table S1: ADMET properties prediction for the active compounds. Calculations were performed with the SwissADME tool.

	**Compound Number**	**6**	**12**	**30**	**36**	**66**	**70**	**74**	**78**
**Physicochemical Properties**	MW	216,19	234,18	250,64	261,19	230,22	248,21	260,25	264,66
	Heavy atoms	16	17	17	19	17	18	19	18
	Aromatic heavy atoms	9	9	9	9	9	9	9	9
	Fraction Csp3	0,09	0,09	0,09	0,09	0,17	0,17	0,23	0,17
	Rotatable bonds	0	0	0	1	0	0	1	0
	H-bond acceptors	3	4	3	5	3	4	4	3
	H-bond donors	1	1	1	1	1	1	1	1
	MR	59,02	58,98	64,03	67,84	63,83	63,79	70,32	68,84
	TPSA	62,54	62,54	62,54	108,36	62,54	62,54	71,77	62,54
**Lipophilicity**	Log Po/w (iLOGP)	1,04	1,42	1,44	0,78	1,50	1,40	1,75	1,78
	Log Po/w (XLOGP3)	0,99	1,09	1,62	0,82	1,39	1,49	1,36	2,02
	Log Po/w (WLOGP)	0,63	1,19	1,29	0,54	1,19	1,75	1,20	1,85
	Log Po/w (MLOGP)	1,13	1,54	1,68	0,21	1,41	1,82	1,14	1,95
	Log Po/w (SILICOS-IT)	0,20	0,61	0,83	-1,98	0,26	0,67	0,28	0,89
	Consensus Log Po/w	0,80	1,17	1,37	0,07	1,15	1,43	1,14	1,70
**Water Solubility**	ESOL Log S	-2,22	-2,37	-2,81	-2,26	-2,53	-2,69	-2,59	-3,12
	ESOL Solubility (mg/ml)	1,30e+00	9,98e-01	3,91e-01	1,43e+00	6,72e-01	5,10e-01	6,62e-01	1,99e-01
	ESOL Class	Soluble	Soluble	Soluble	Soluble	Soluble	Soluble	Soluble	Soluble

	Ali Log S	-1,89	-2,00	-2,55	-2,68	-2,31	-2,41	-2,47	-2,96
	Ali Solubility (mg/ml)	2,77e+00	2,37e+00	7,14e-01	5,49e-01	1,14e+00	9,64e-01	8,83e-01	2,90e-01
	Ali Class	Very soluble	Very soluble	Soluble	Soluble	Soluble	Soluble	Soluble	Soluble
	Silicos-IT LogSw	-1,83	-2,11	-2,45	-1,21	-1,99	-2,27	-2,12	-2,60
	Silicos-IT Solubility (mg/ml)	3,17e+00	1,80e+00	8,91e-01	1,61e+01	2,35e+00	1,34e+00	2,00e+00	6,62e-01
	Silicos-IT class	Soluble	Soluble	Soluble	Soluble	Soluble	Soluble	Soluble	Soluble
**Pharmacokinetics**	GI absorption	High	High	High	High	High	High	High	High
	BBB permeant	No	Yes	Yes	No	Yes	Yes	No	Yes
	Pgp substrate	No	No	No	No	No	No	No	No
	CYP1A2 inhibitor	No	No	Yes	No	No	No	No	Yes
	CYP2C19 inhibitor	No	No	No	No	No	No	No	No
	CYP2C9 inhibitor	No	No	No	No	No	No	No	No
	CYP2D6 inhibitor	No	No	No	No	No	No	No	No
	CYP3A4 inhibitor	No	No	No	No	No	No	No	No
	log Kp (cm/s)	-6,92	-6,95	-6,68	-7,31	-6,72	-6,76	-6,92	-6,48
**Druglikeness**	Lipinski violations	0	0	0	0	0	0	0	0
	Ghose violations	0	0	0	0	0	0	0	0
	Veber violations	0	0	0	0	0	0	0	0
	Egan violations	0	0	0	0	0	0	0	0
	Muegge violations	0	0	0	0	0	0	0	0
	Bioavailability Score	0,55	0,55	0,55	0,55	0,55	0,55	0,55	0,55
**Med icin**	PAINS alerts	0	0	0	0	0	0	0	0

	Brenk alerts	2*	2*	2*	4**	2*	2*	2*	2*
	Leadlikeness violations	1***	1***	0	0	1***	1***	0	0
	Synthetic Accessibility	2,22	2,29	2,24	2,48	2,76	2,82	2,85	2,77

*hydroxamic_acid and phthalimide

**hydroxamic_acid, nitro_group, oxygen-nitrogen_single_bond and phthalimide

***MW<250

IV. Copies of NMR spectra

^1H NMR of **2** (400.13 MHz, $CDCl_3$)

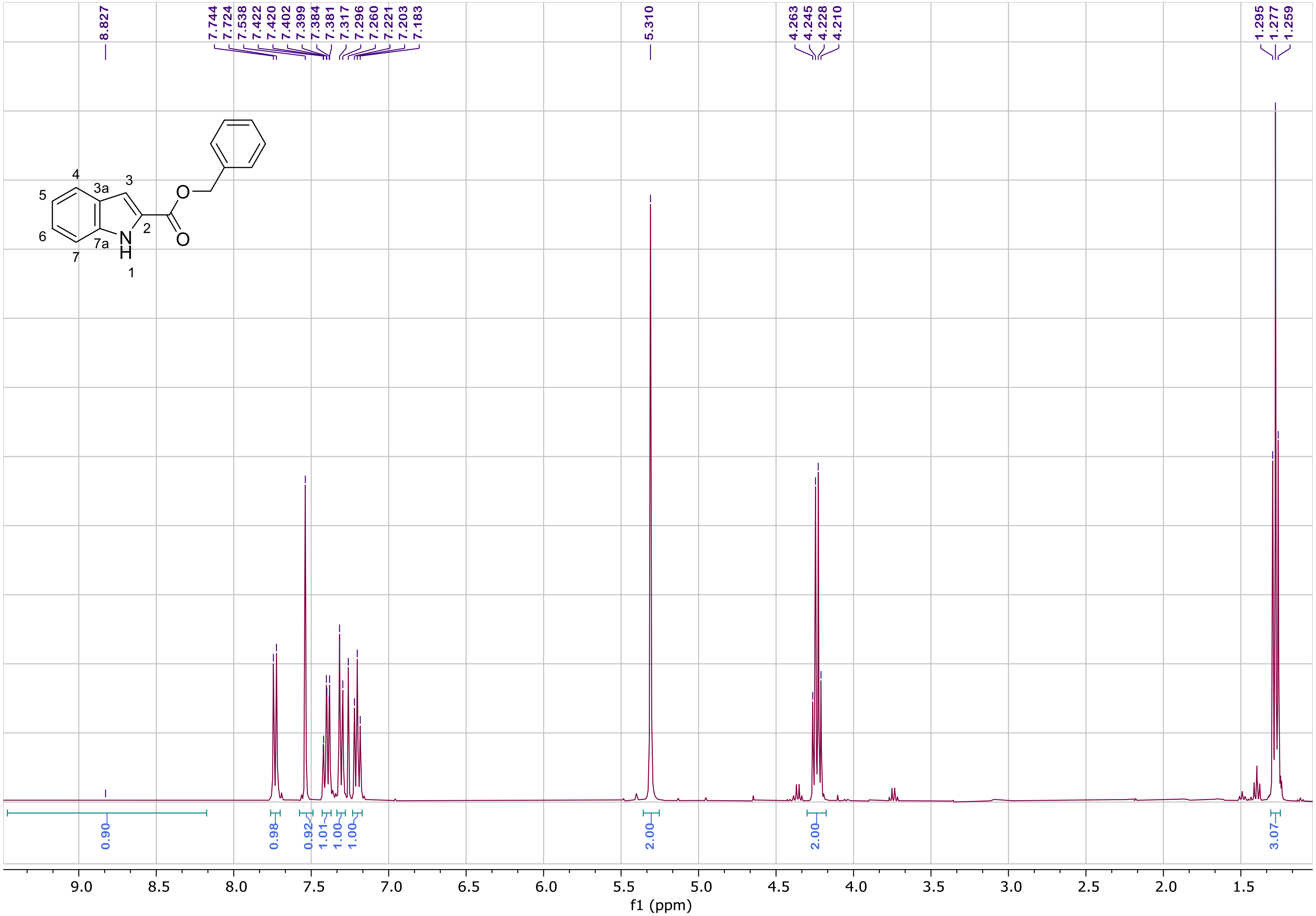

^{13}C NMR of **2** (50.32 MHz, CDCl$_3$)

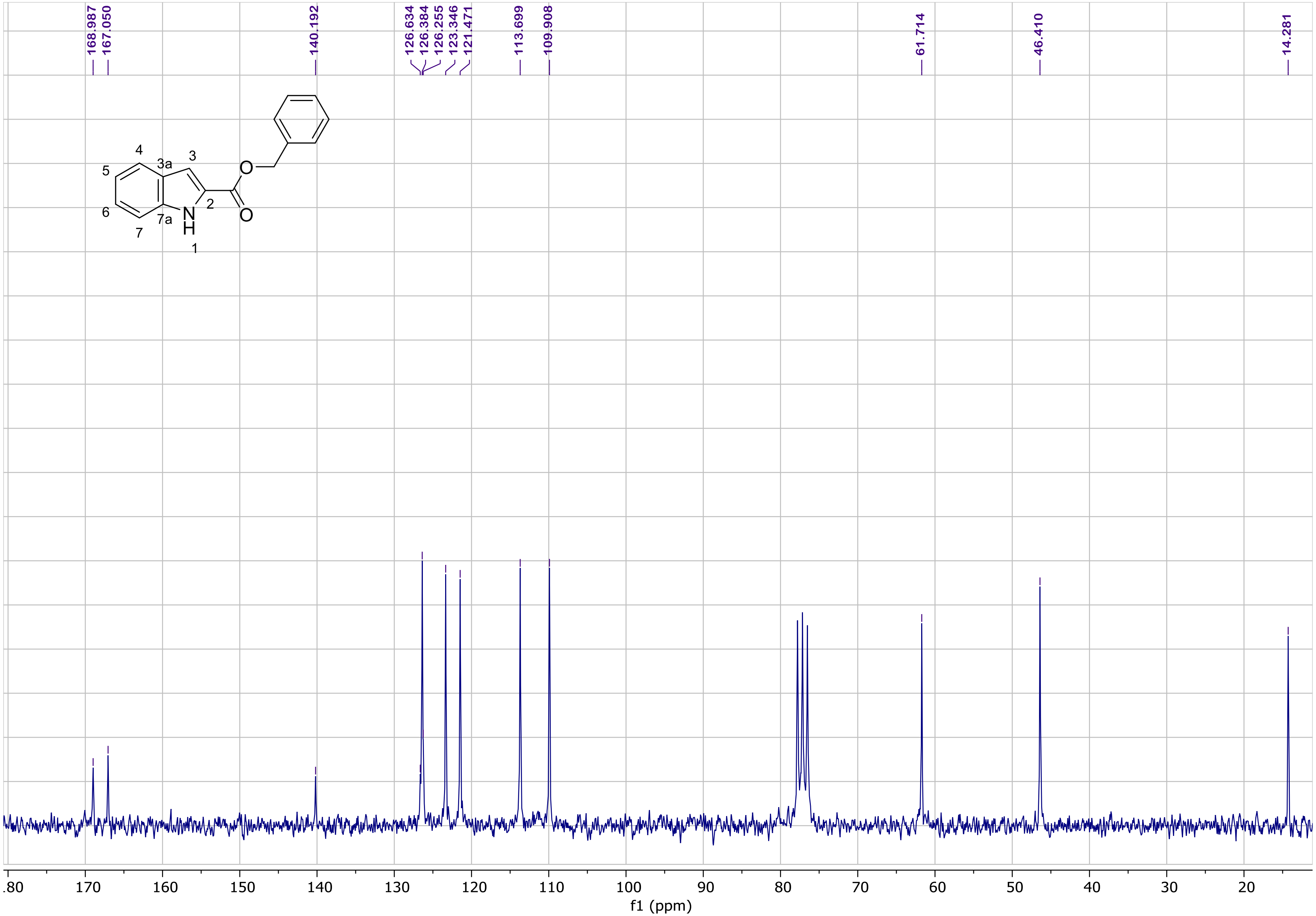

DEPT NMR of **2** (50.32 MHz, $CDCl_3$)

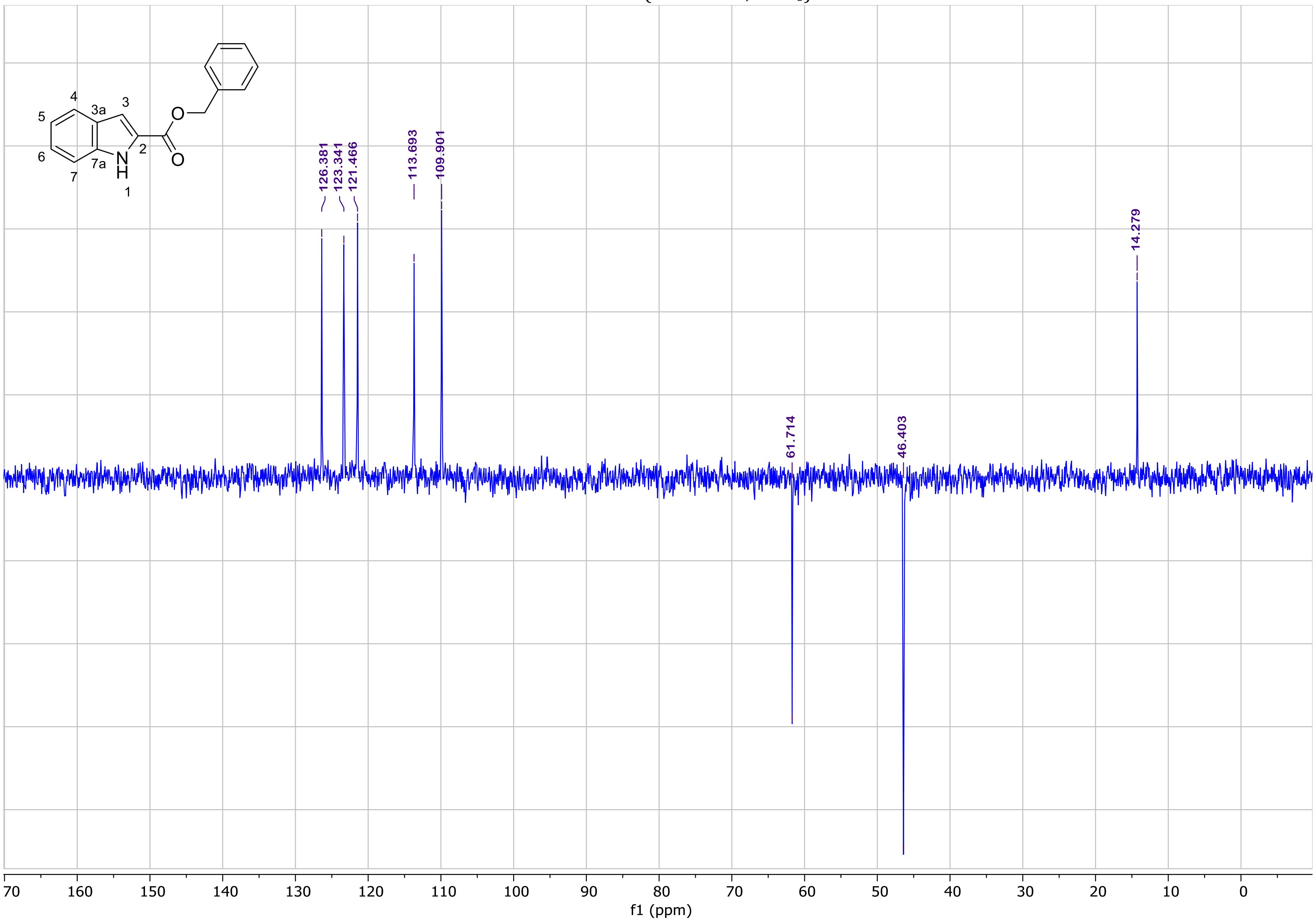

COSY NMR of **2** (400.13 MHz, $CDCl_3$)

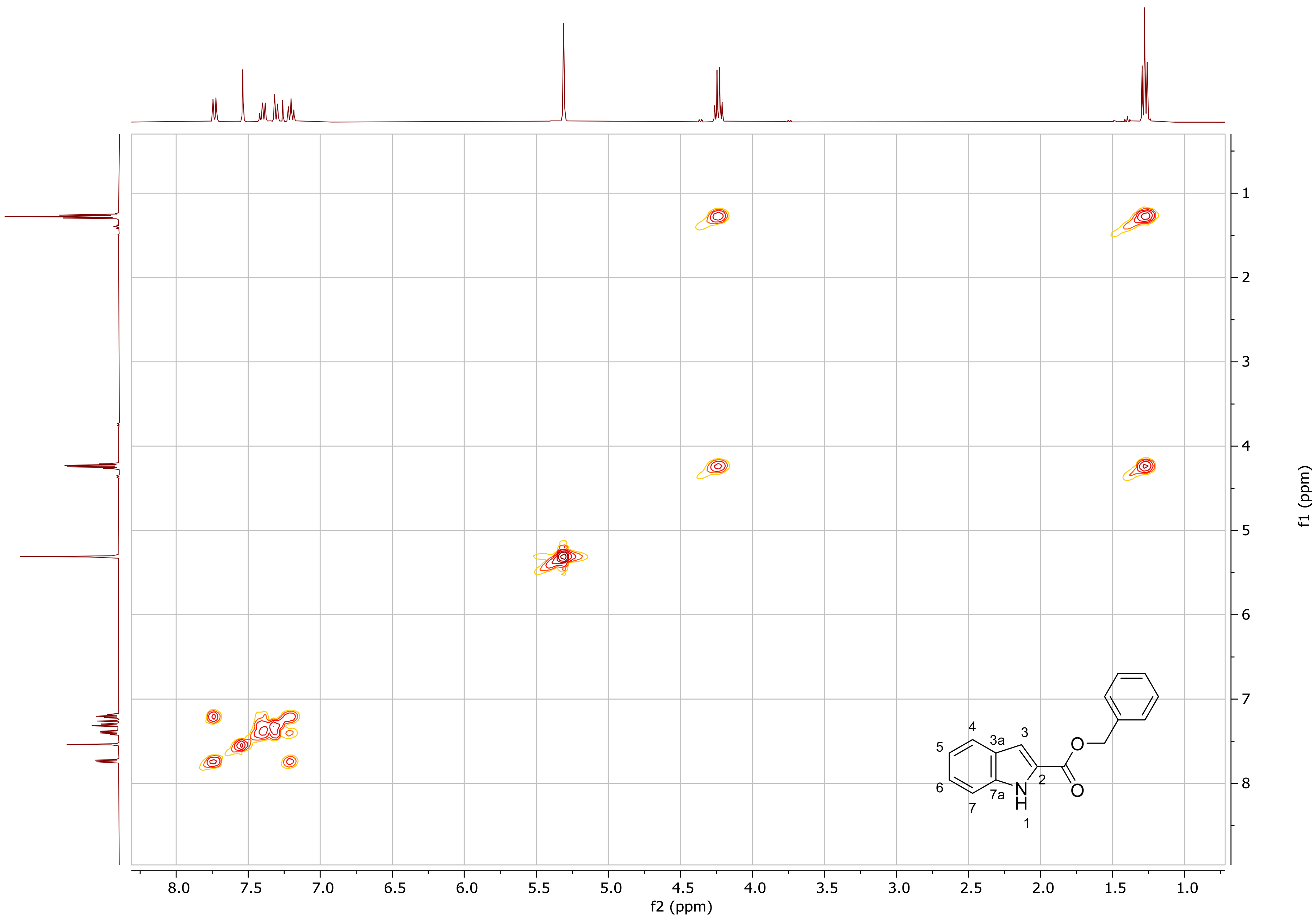

HSQC NMR of **2** (400.13 MHz, $CDCl_3$)

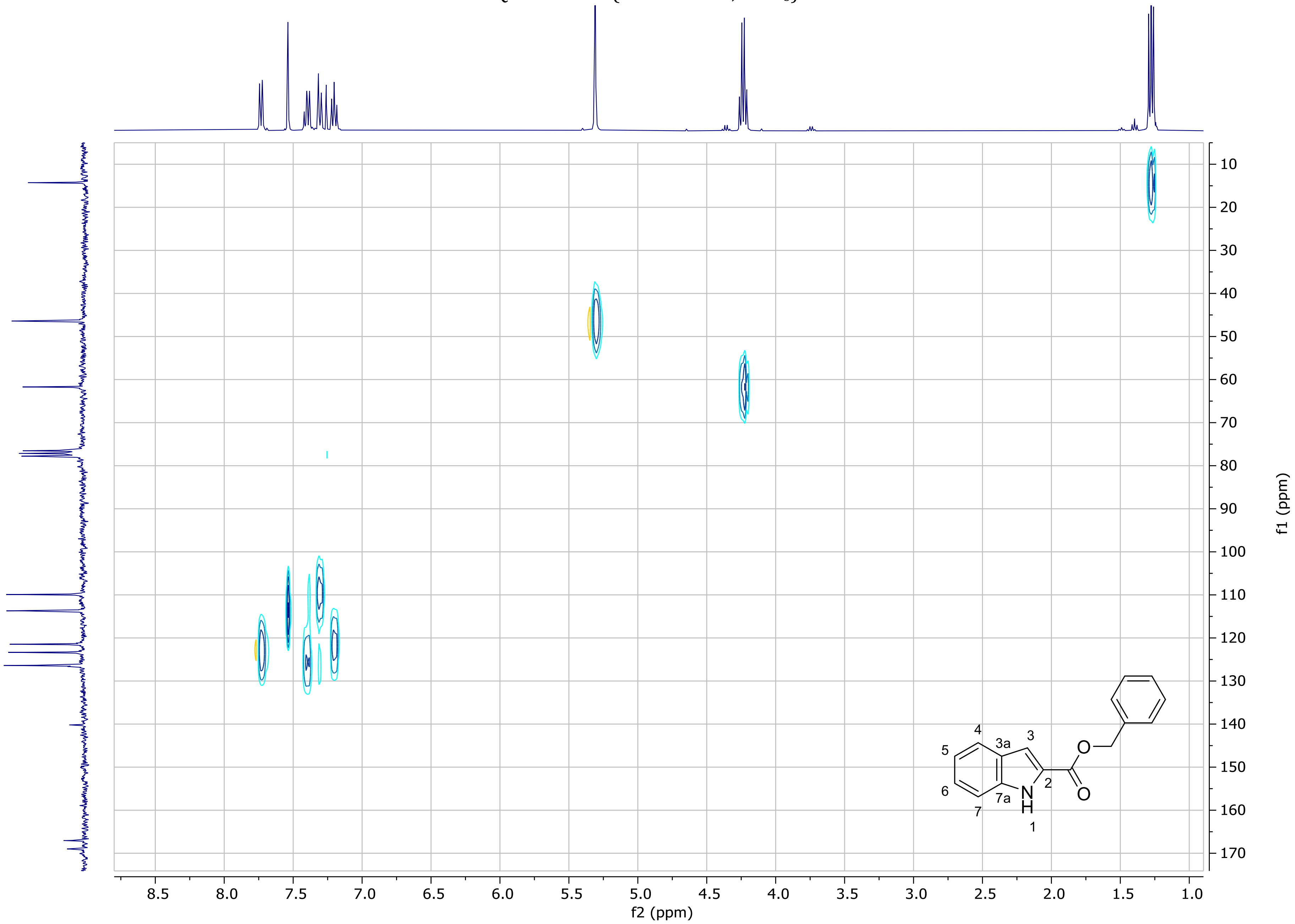

HMBC NMR of of **2** (400.13 MHz, $CDCl_3$)

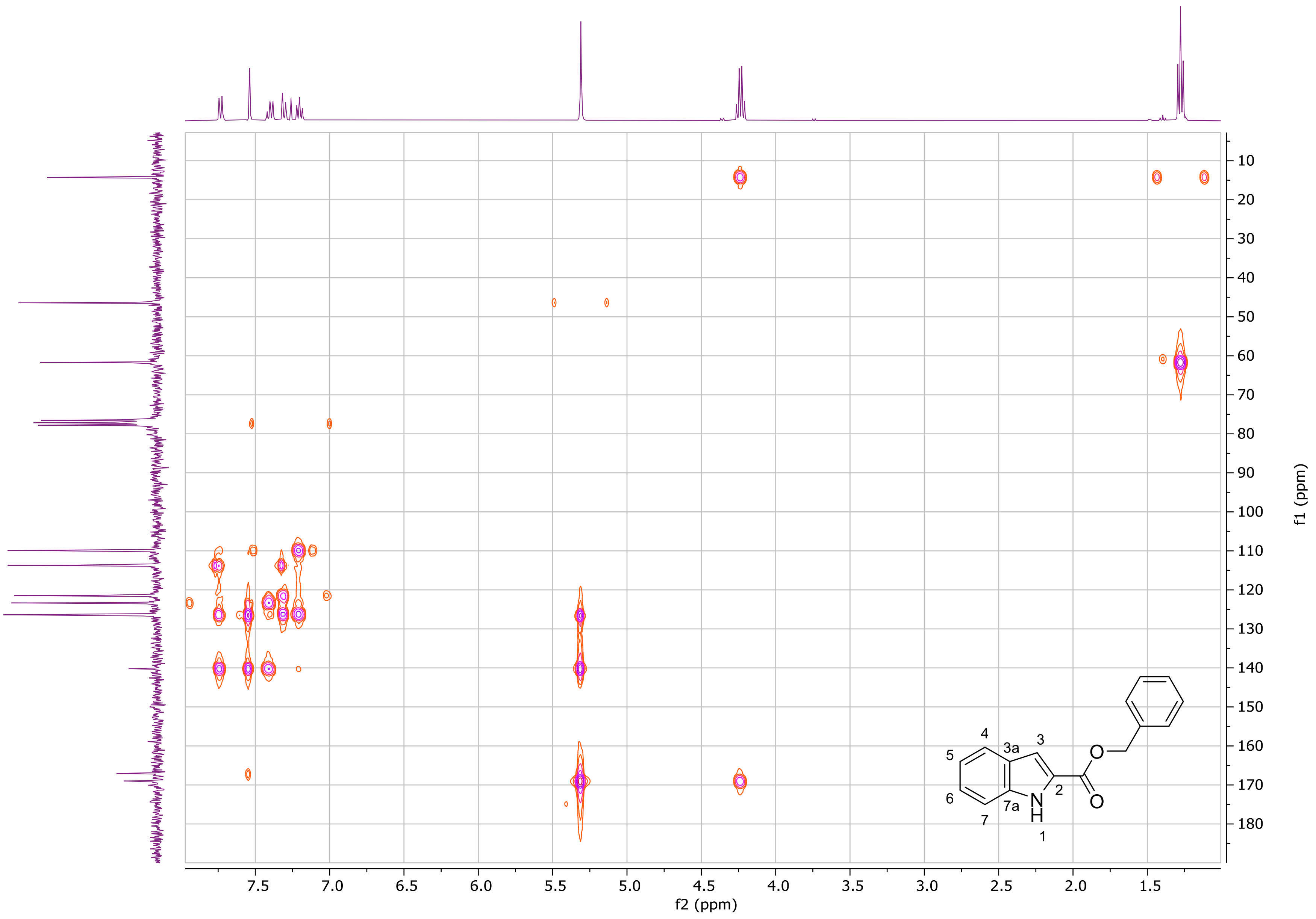

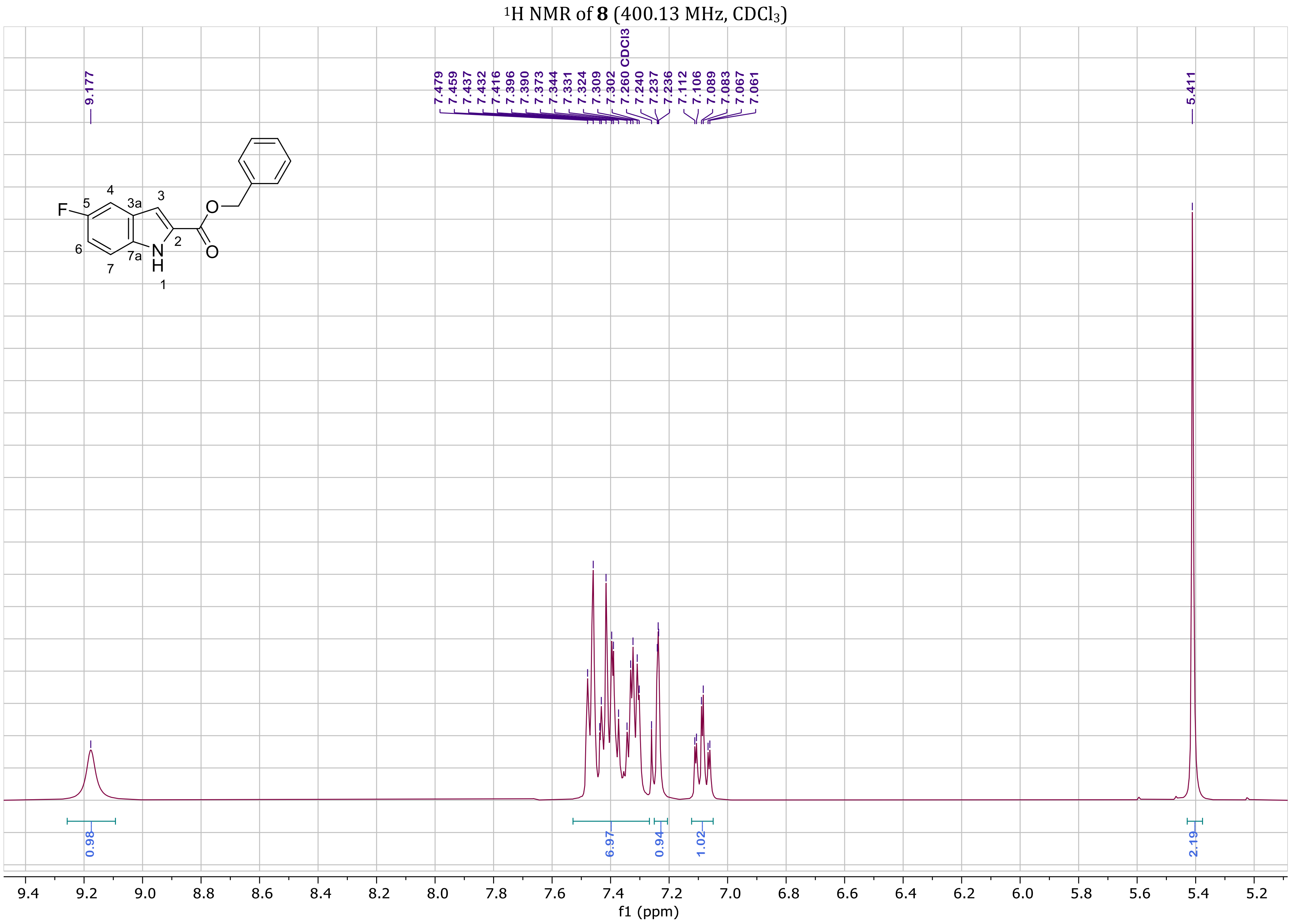

¹H NMR of 8 (400.13 MHz, CDCl3)
9.177
7.479
7.459
7.437
7.432
7.416
7.396
7.390
7.373
7.344
7.331
7.324
7.309
7.302
7.260 CDCl3
7.240
7.237
7.236
7.112
7.106
7.089
7.083
7.067
7.061
5.411
F
5
4
3a
3
O
O
2
N
H
1
7a
6
7
0.98
6.97
0.94
1.02
2.19
9.4
9.2
9.0
8.8
8.6
8.4
8.2
8.0
7.8
7.6
7.4
7.2
7.0
6.8
6.6
6.4
6.2
6.0
5.8
5.6
5.4
5.2
f1 (ppm)

^{13}C NMR of **8** (50.32 MHz, $CDCl_3$)

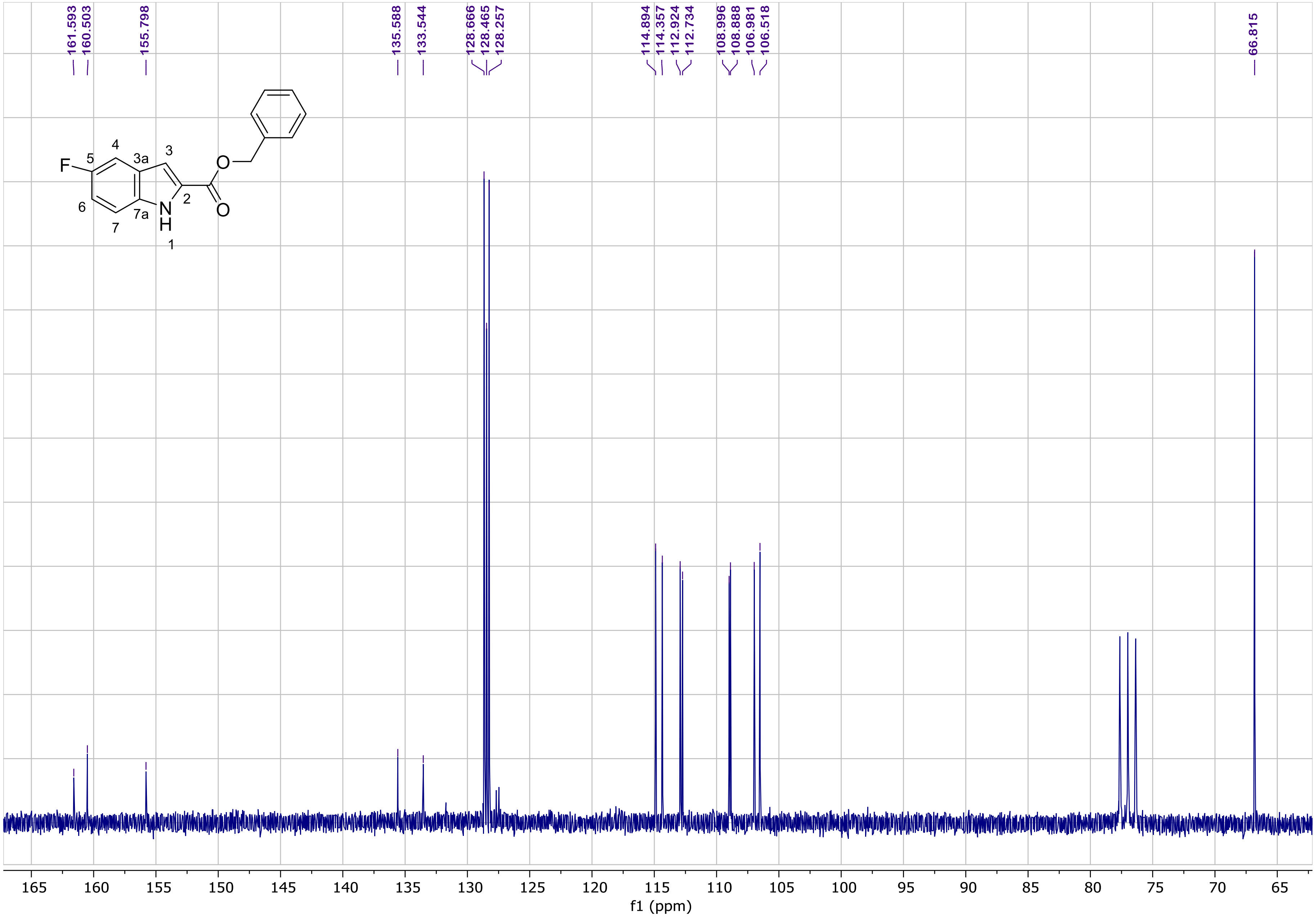

DEPT NMR of **8** (50.32 MHz, $CDCl_3$)

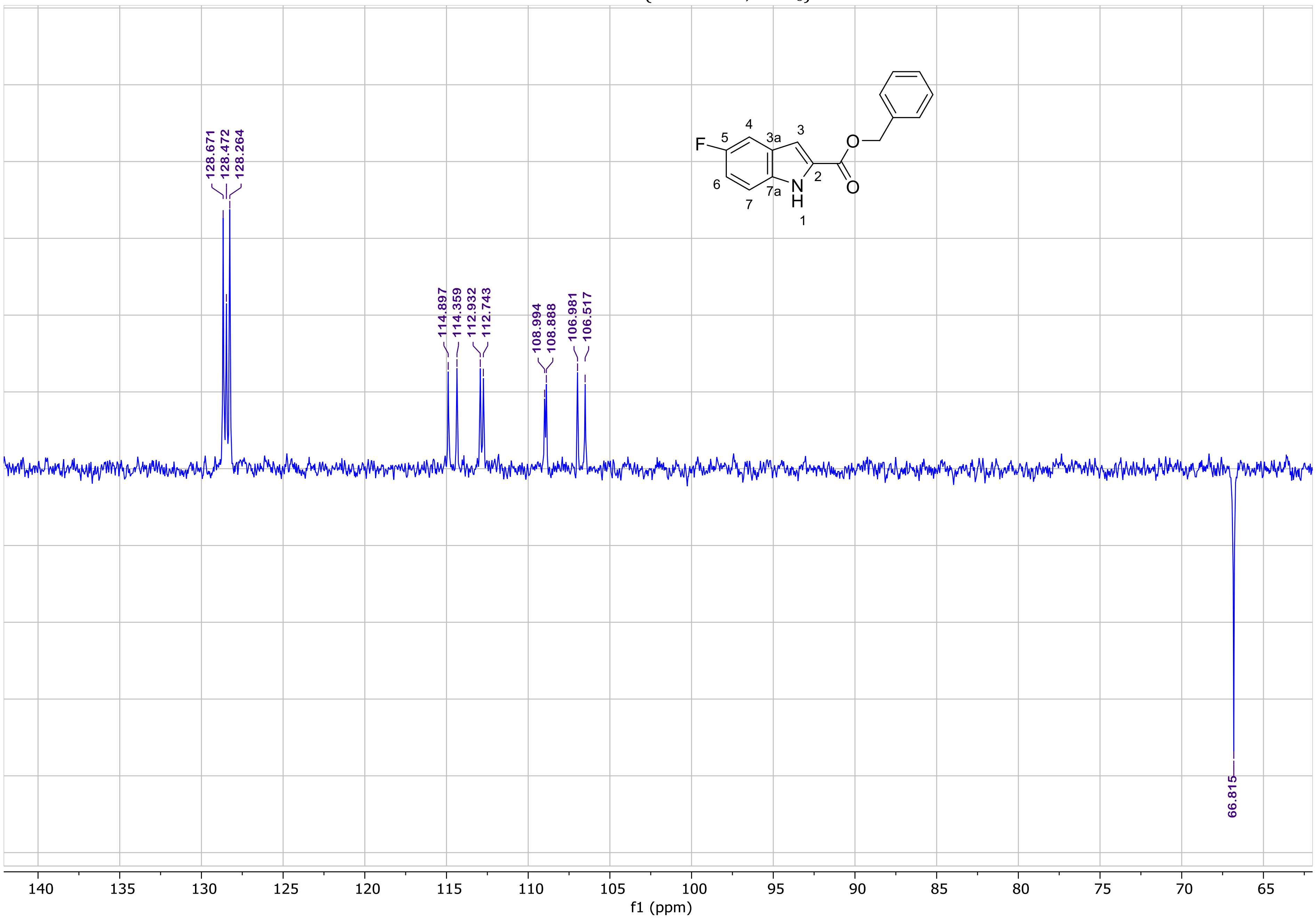

COSY NMR of **8** (400.13 MHz, $CDCl_3$)

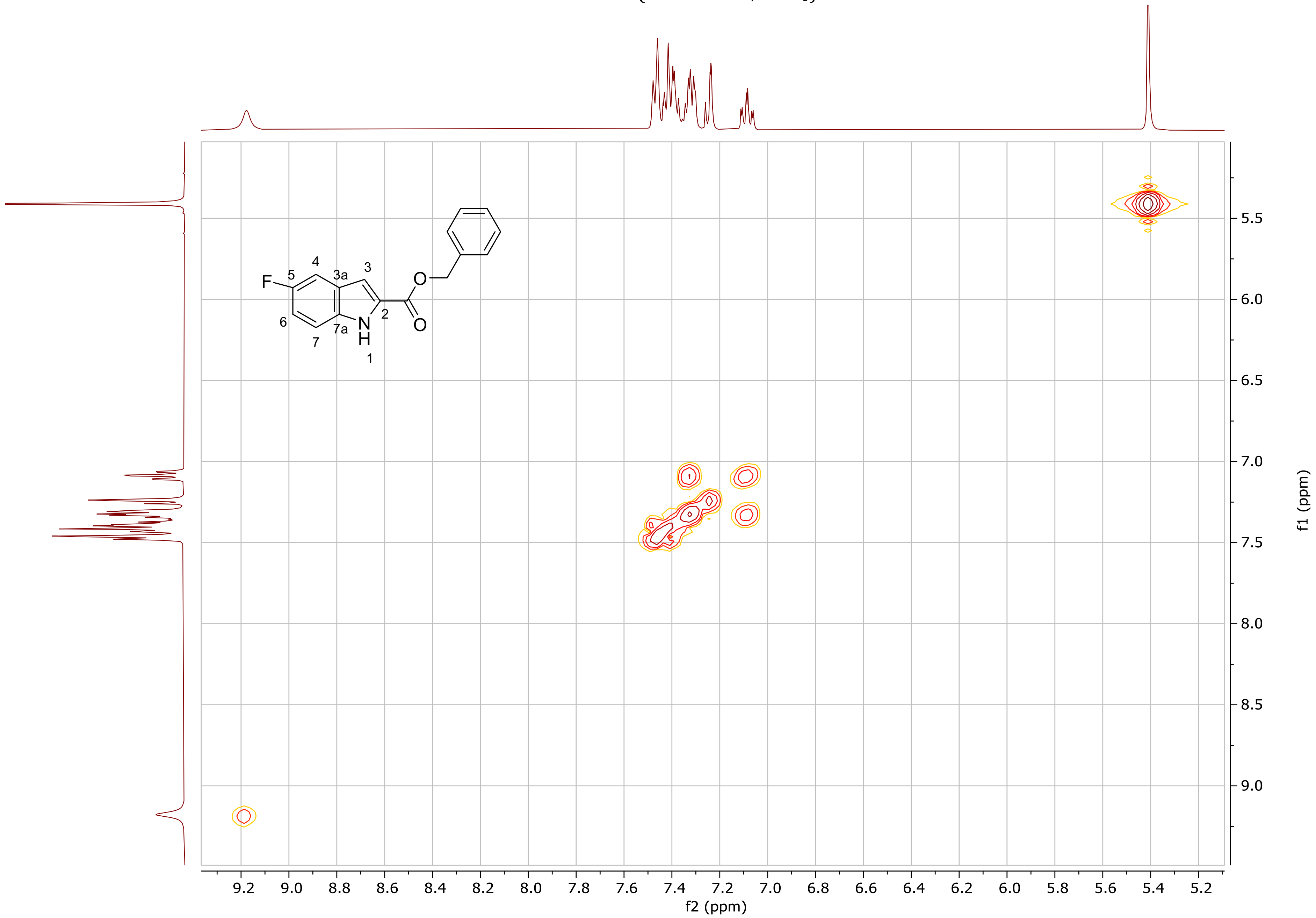

HSQC NMR of **8** (400.13 MHz, $CDCl_3$)

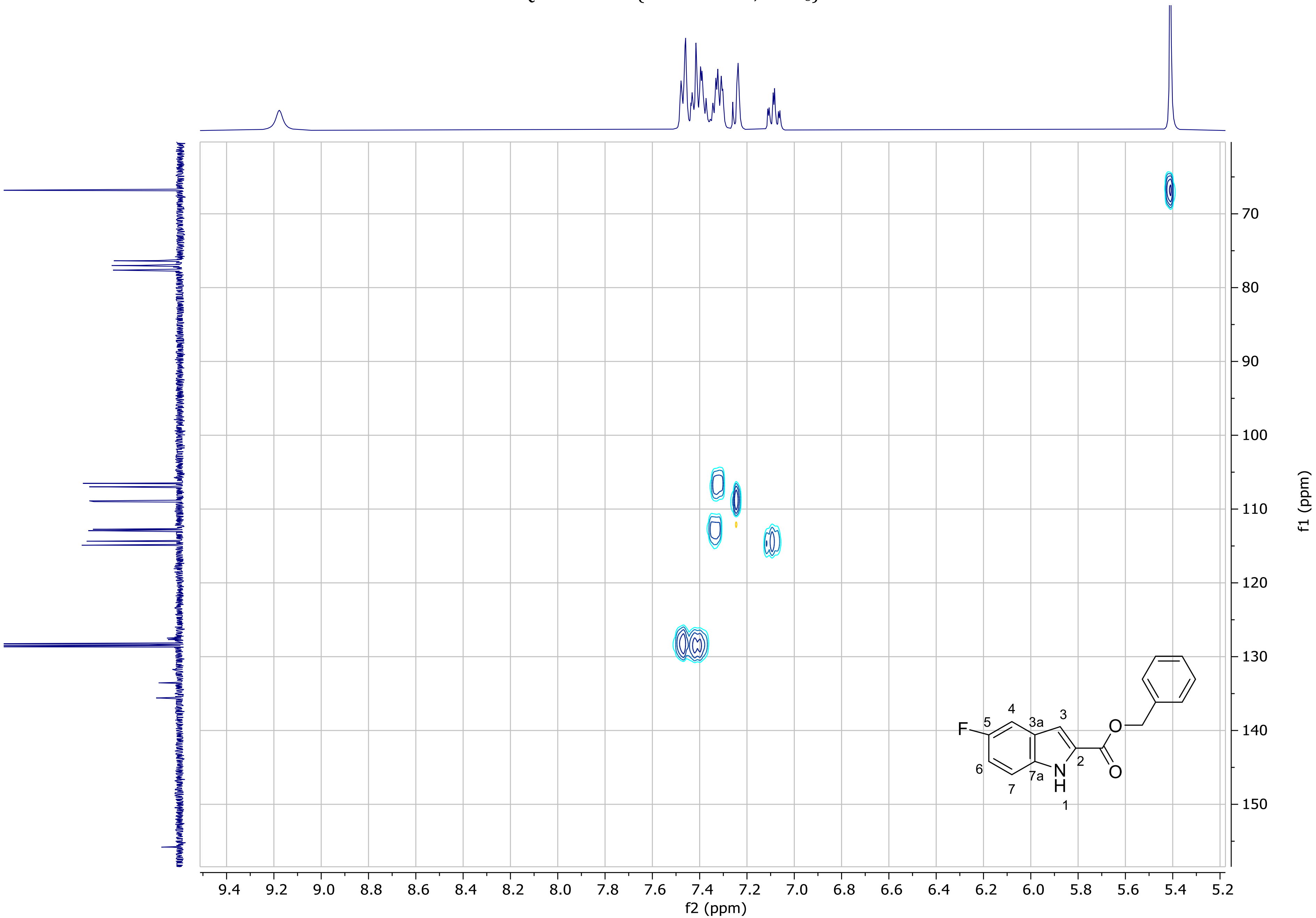

HMBC NMR of of **8** (400.13 MHz, $CDCl_3$)

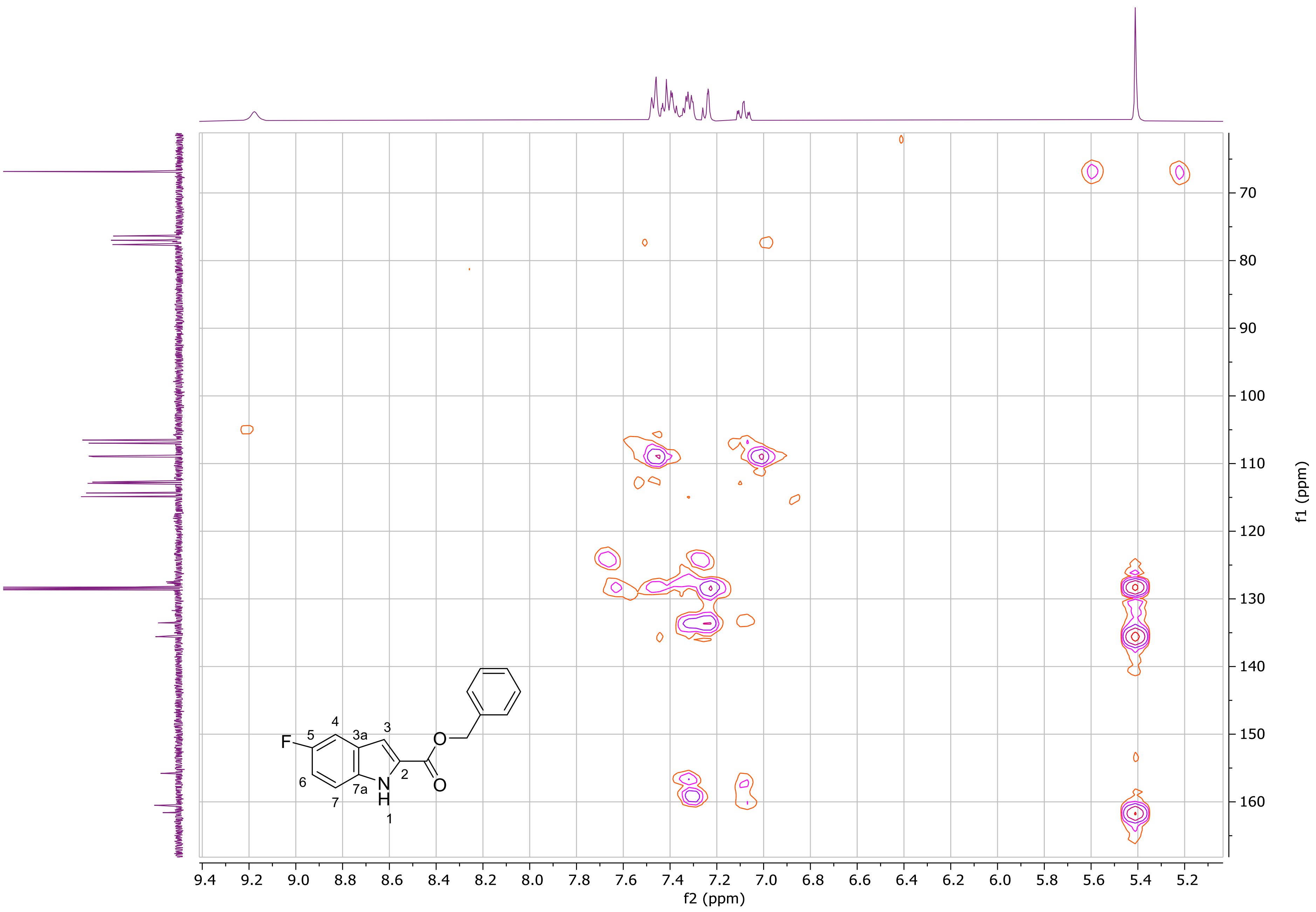

^{1}H NMR of **14** (400.13 MHz, $CDCl_3$)

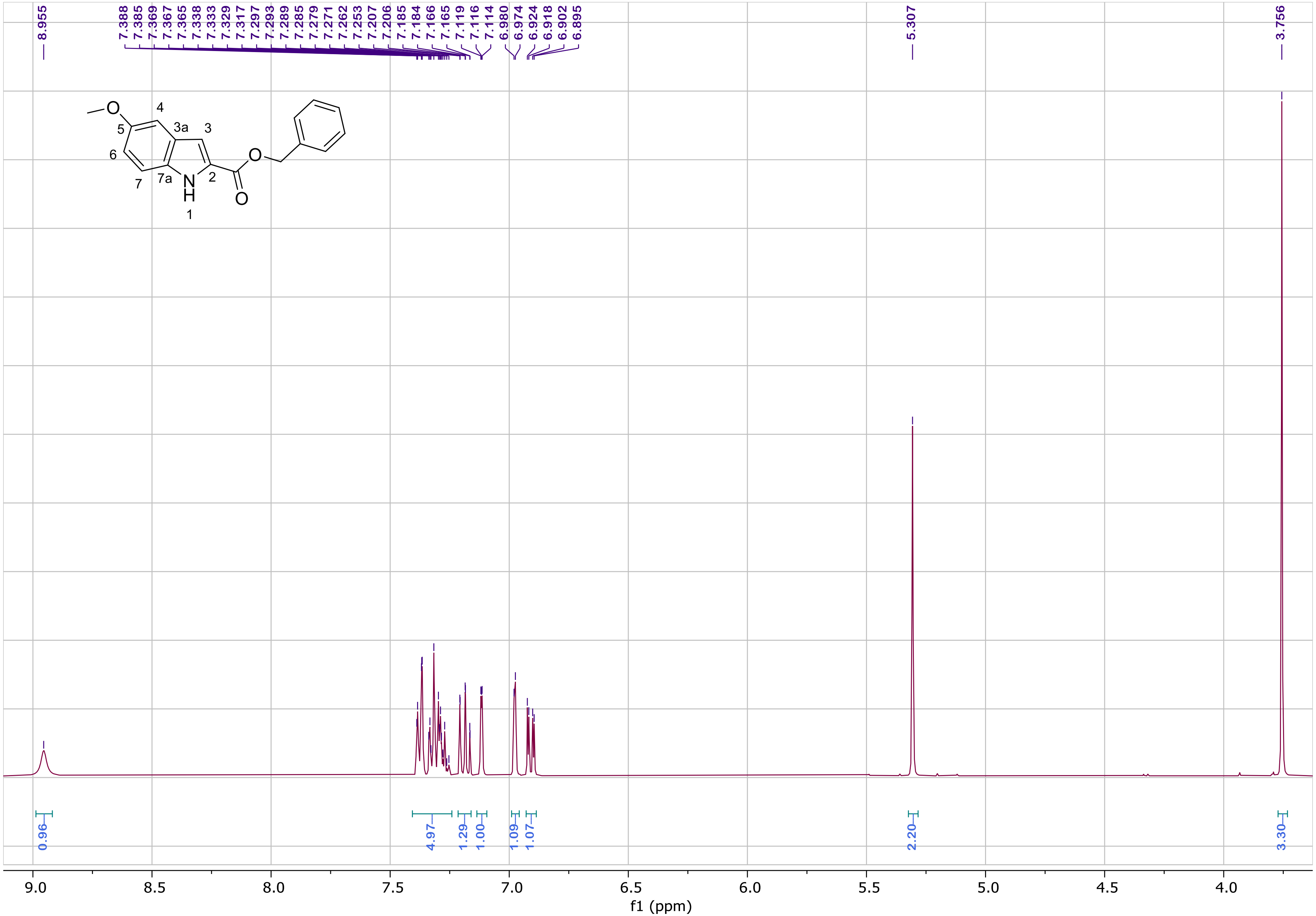

^{13}C NMR of **14** (50.32 MHz, $CDCl_3$)

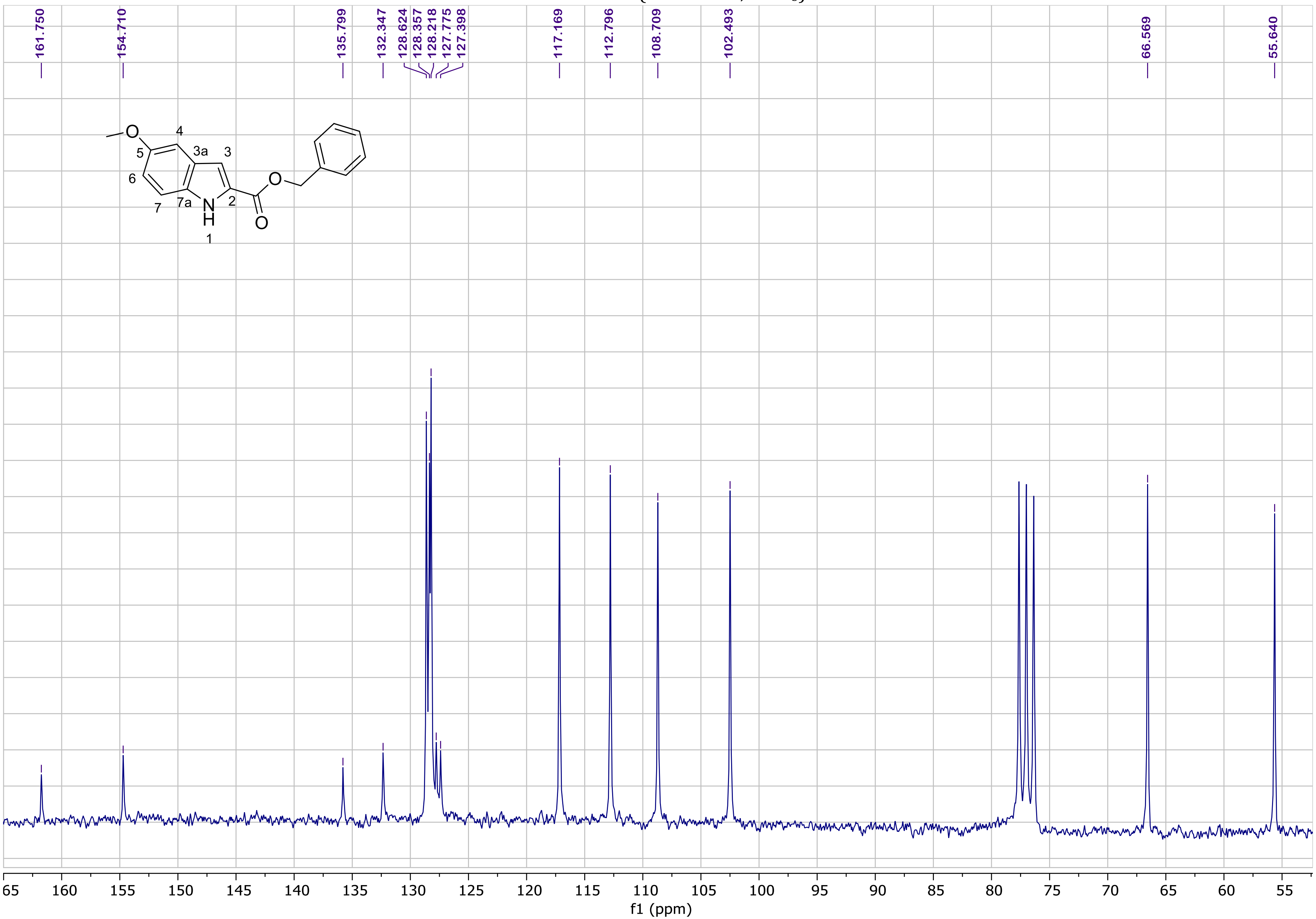

COSY NMR of **14** (400.13 MHz, $CDCl_3$)

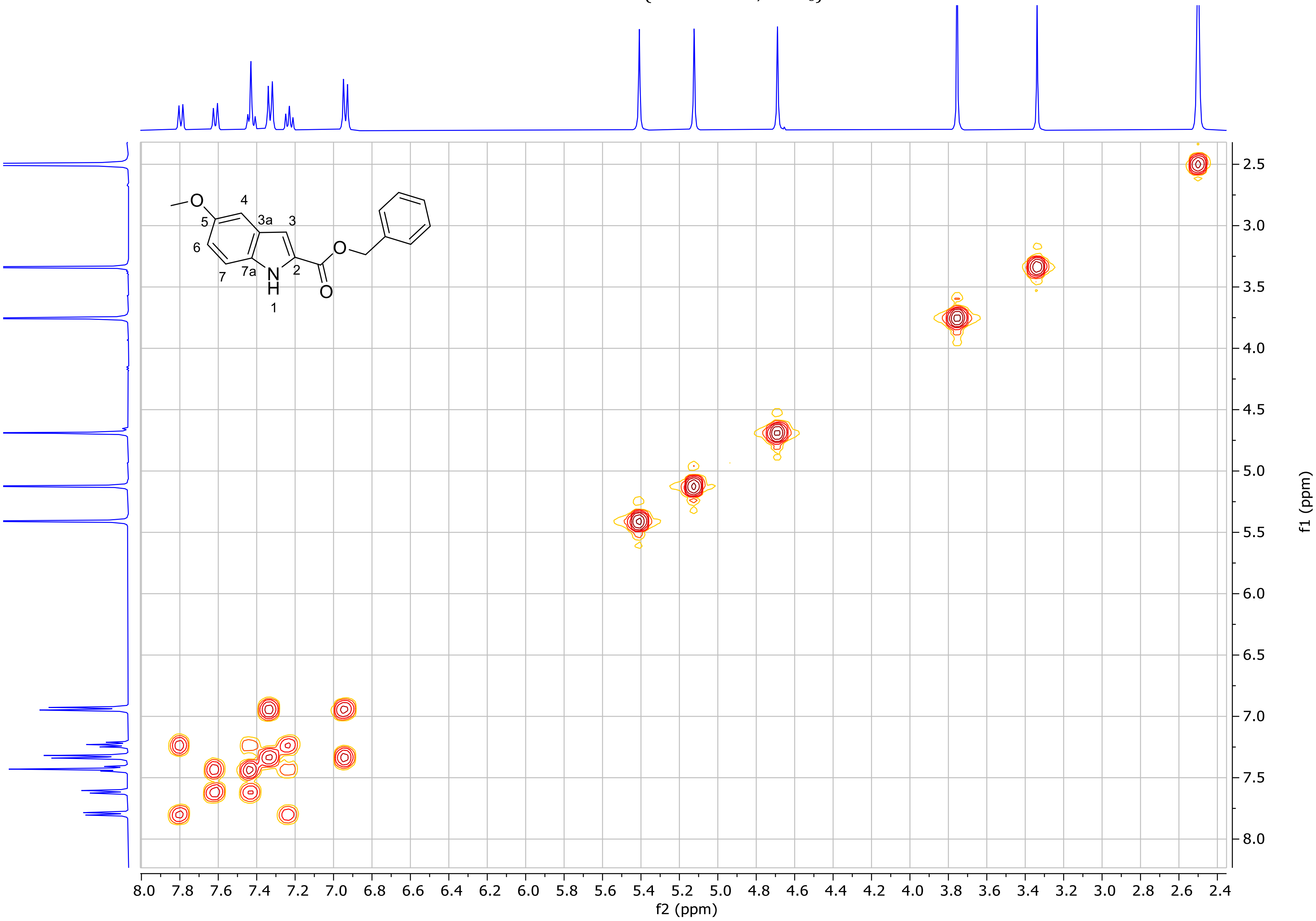

HSQC NMR of **14** (400.13 MHz, $CDCl_3$)

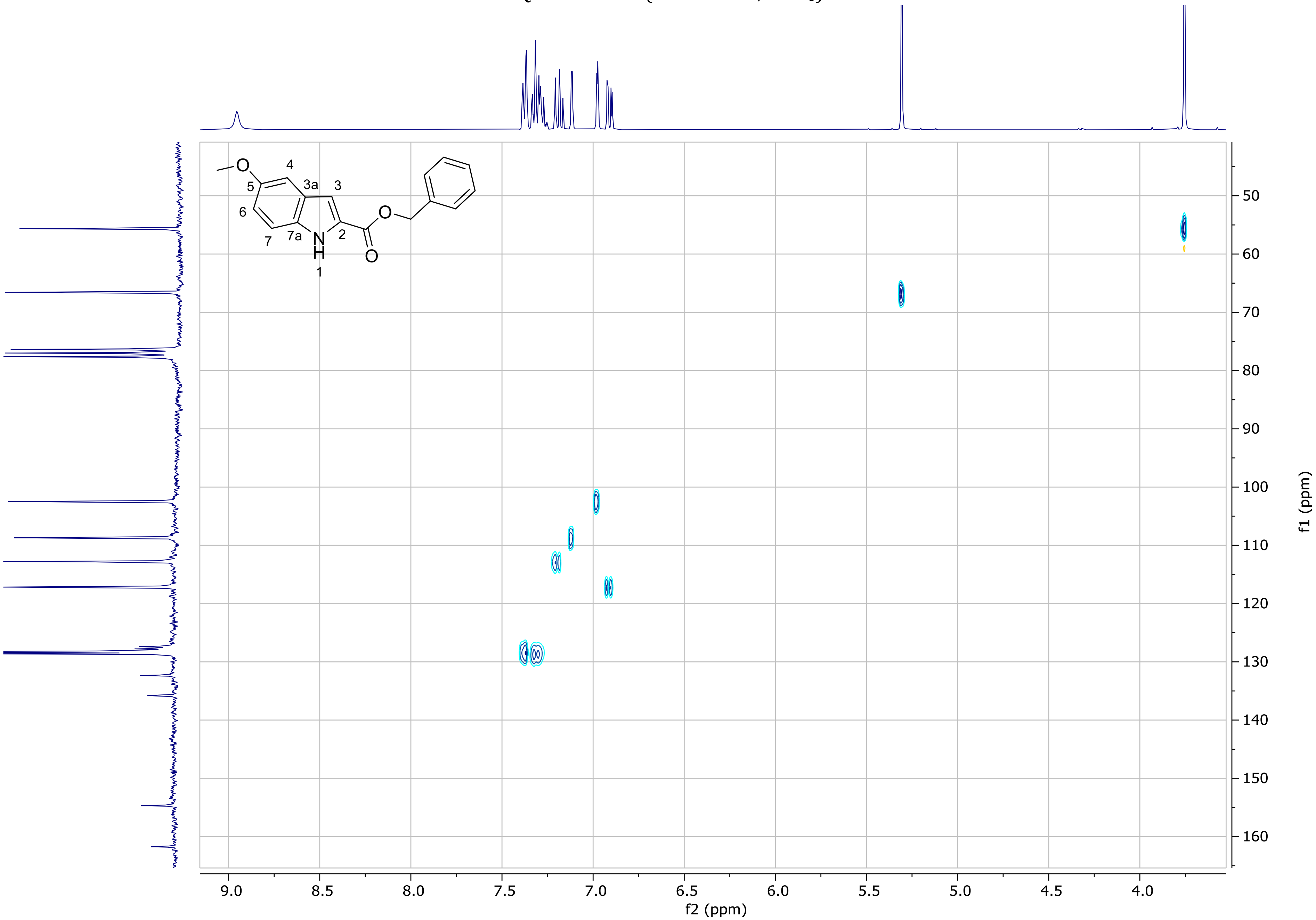

HMBC NMR of **14** (400.13 MHz, $CDCl_3$)

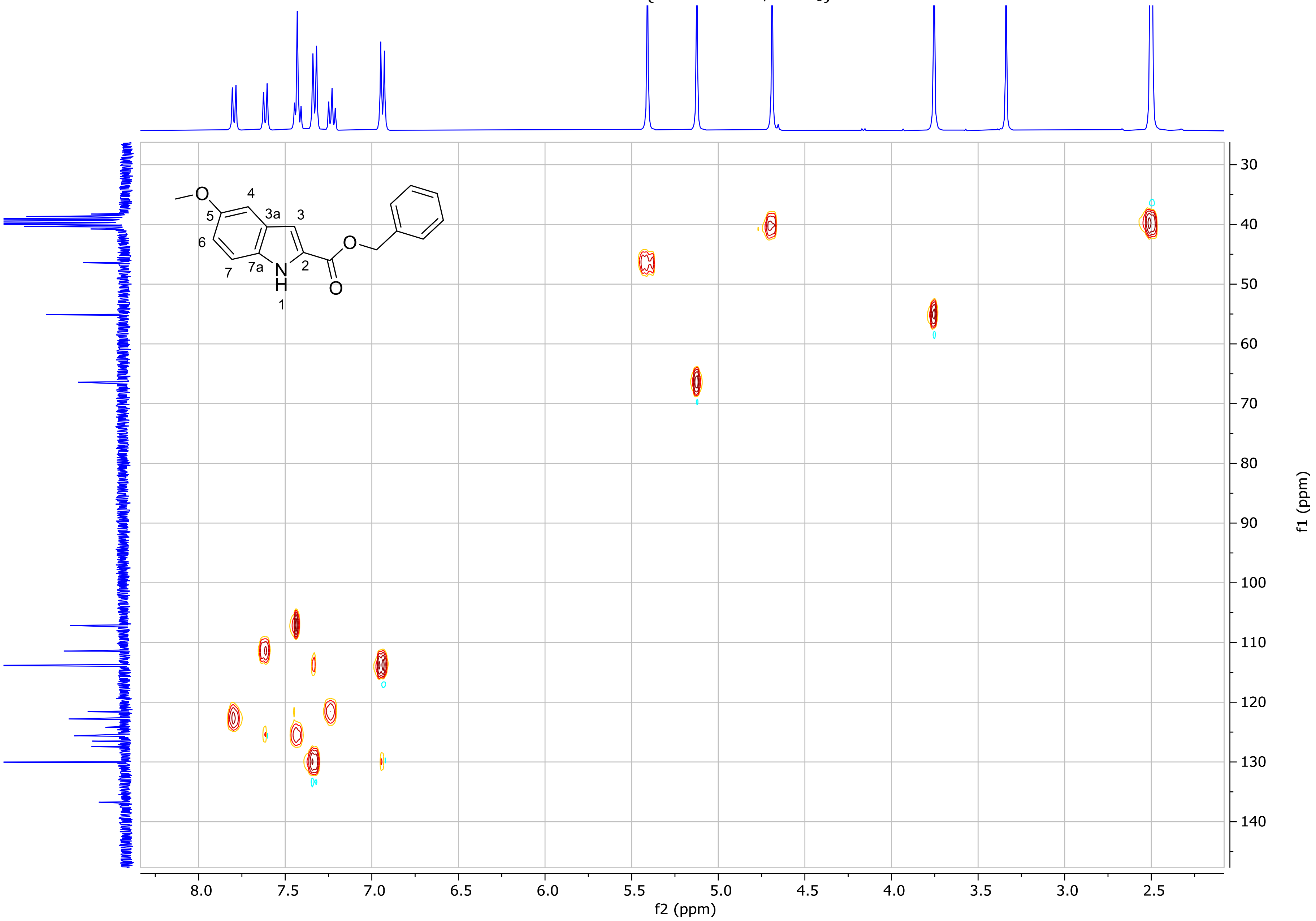

^{1}H NMR of **20** (400.13 MHz, $CDCl_3$)

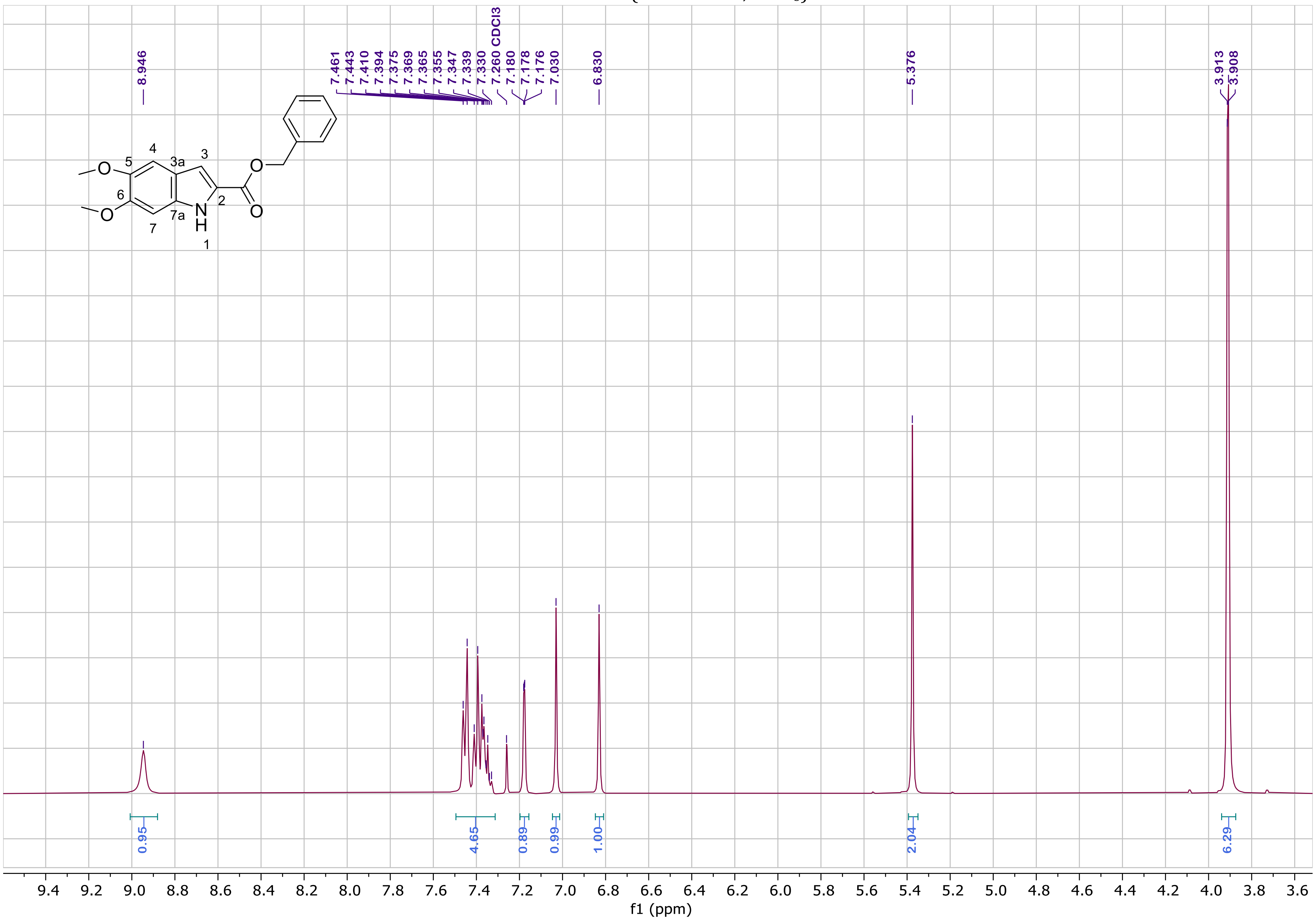

^{13}C NMR of **20** (50.32 MHz, CDCl$_3$)

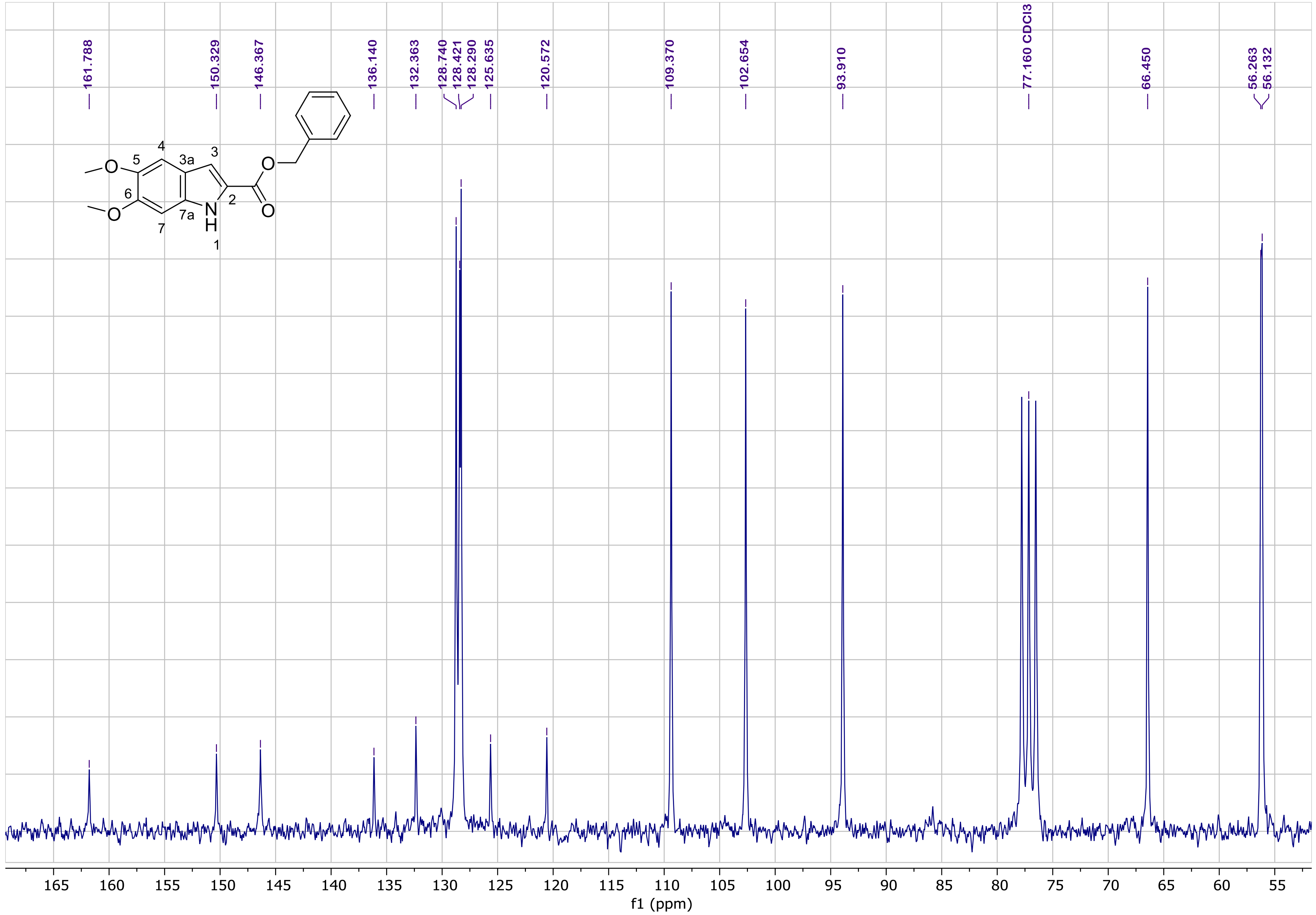

DEPT NMR of **20** (50.32 MHz, $CDCl_3$)

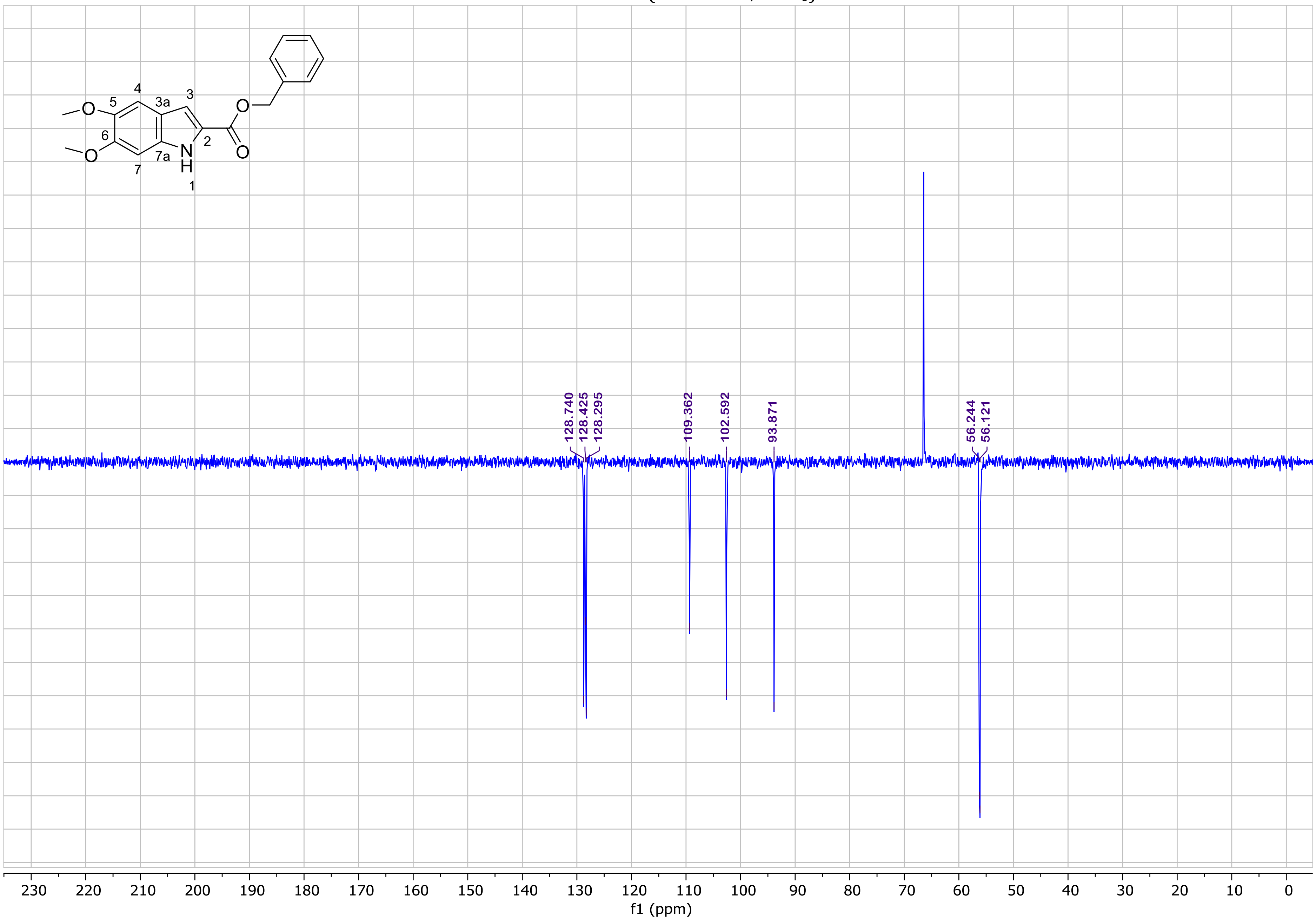

COSY NMR of **20** (400.13 MHz, $CDCl_3$)

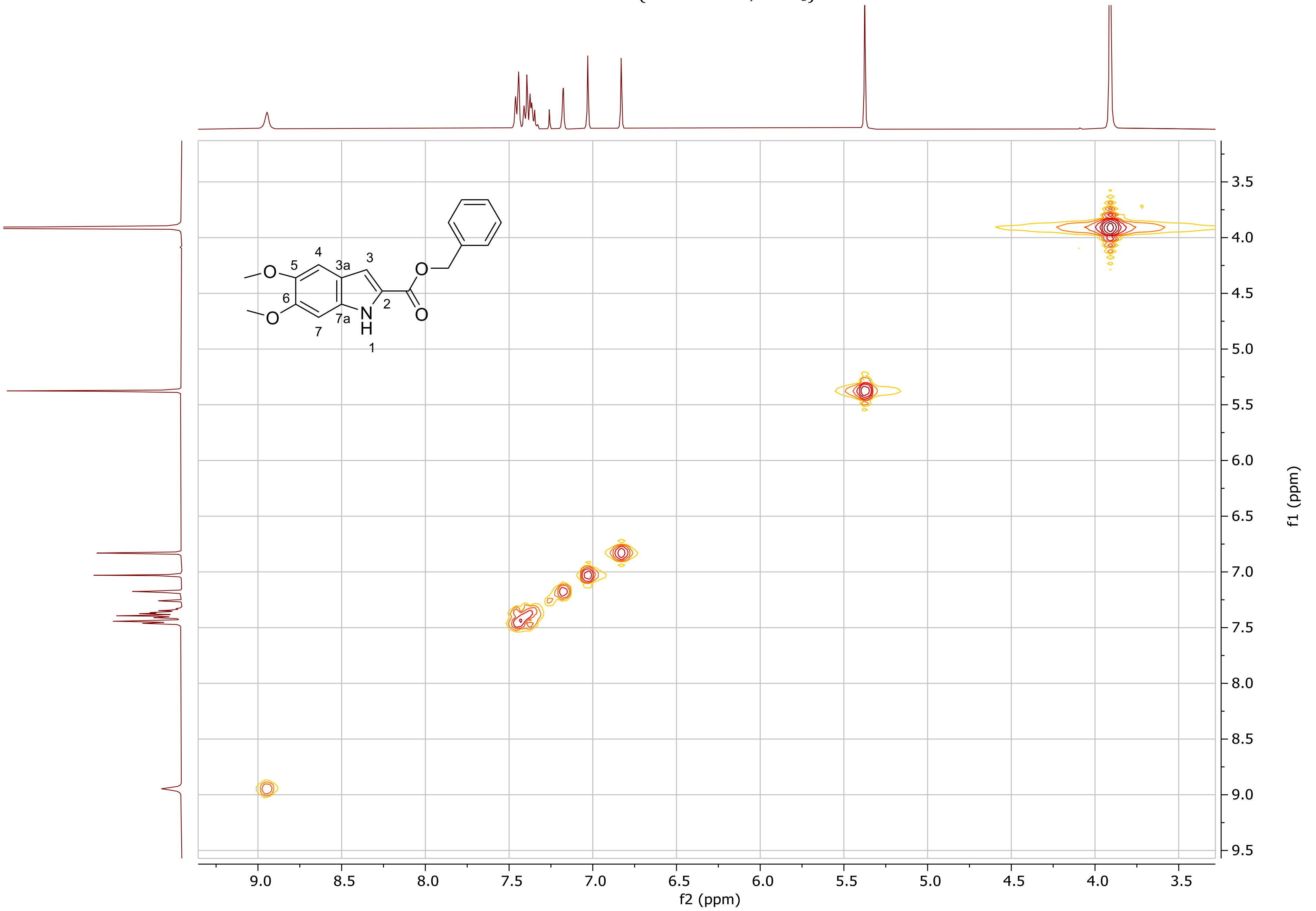

HSQC NMR of **20** (400.13 MHz, $CDCl_3$)

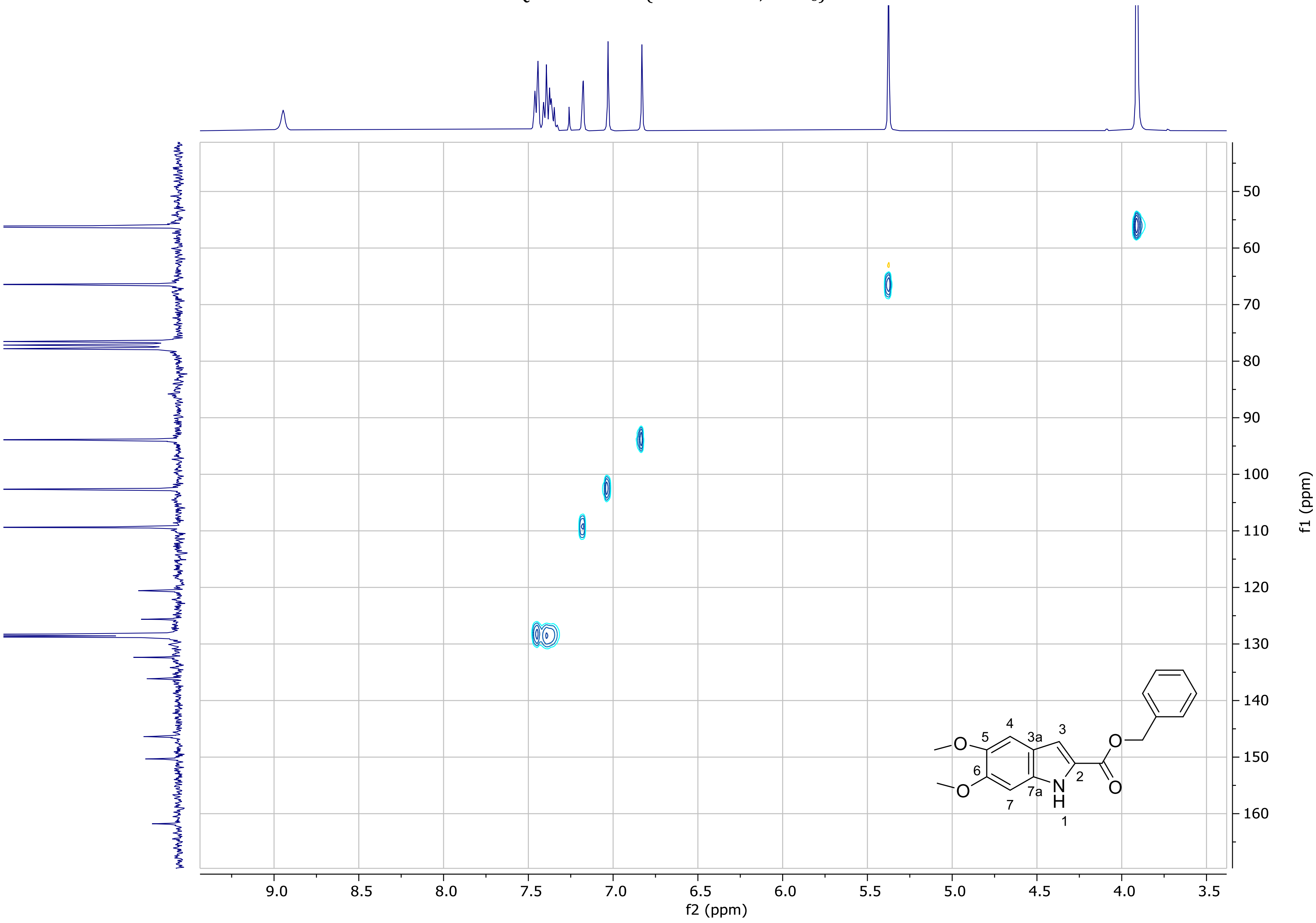

HMBC NMR of **20** (400.13 MHz, $CDCl_3$)

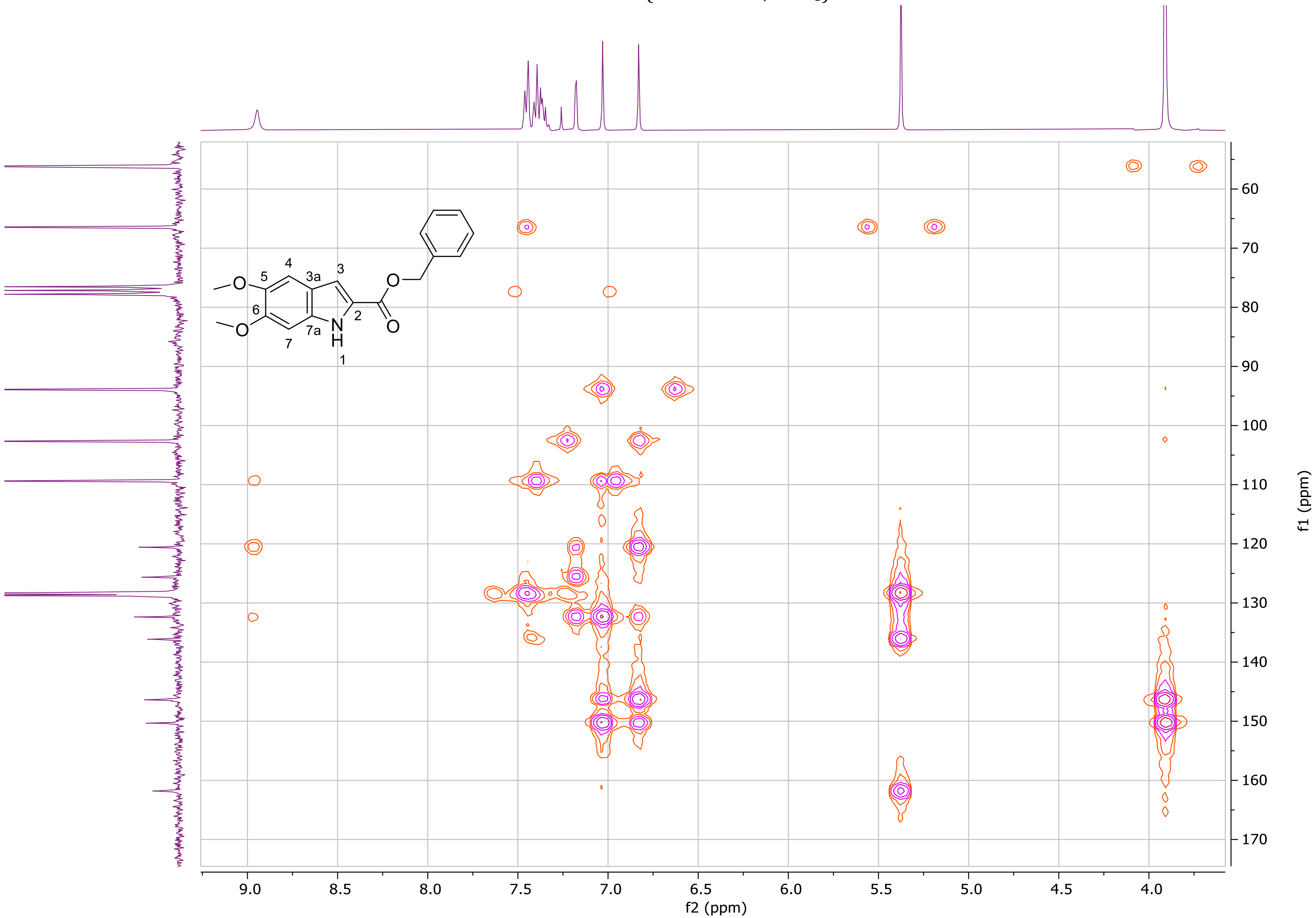

^{1}H NMR of **26** (400.13 MHz, $CDCl_3$)

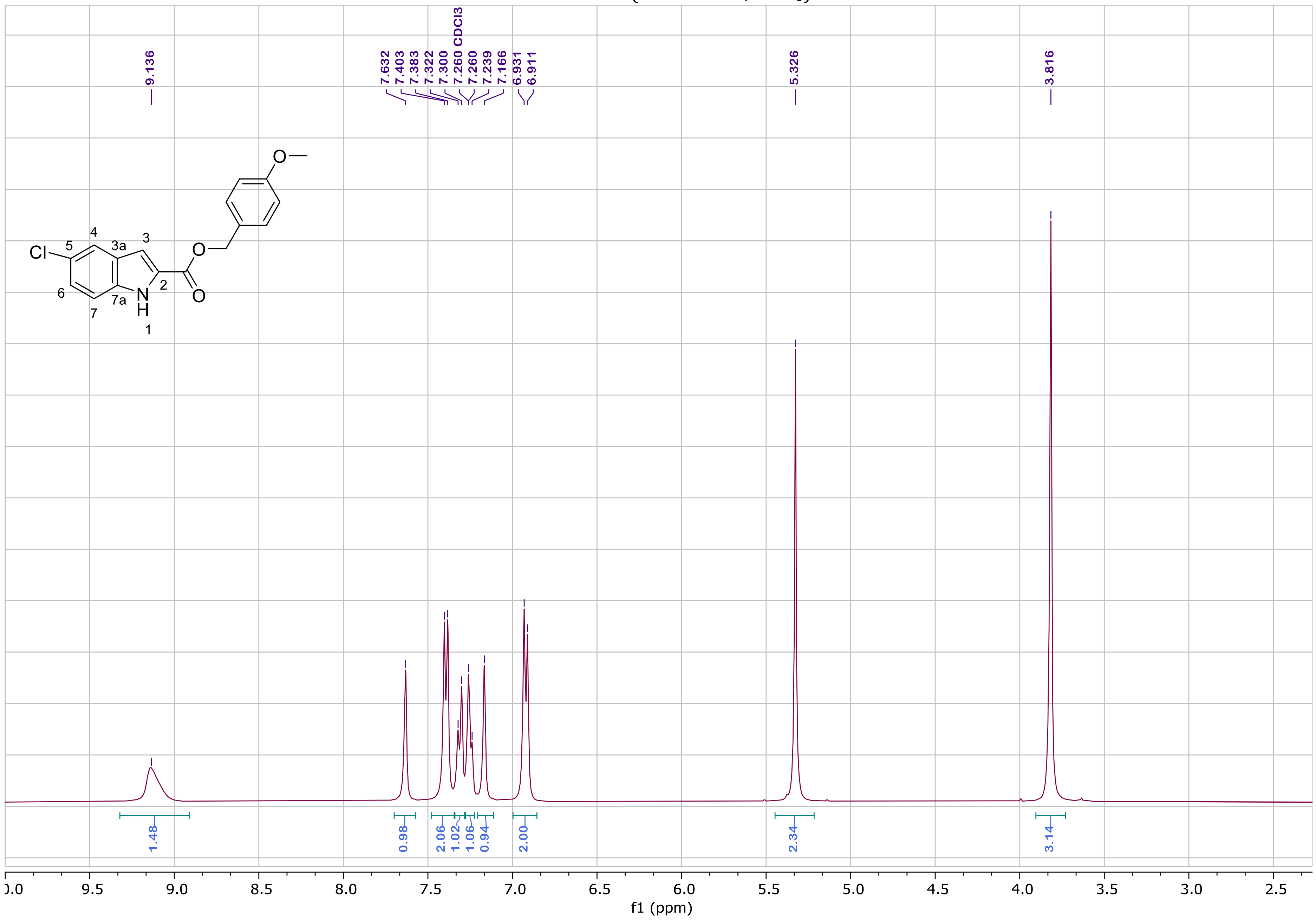

^{13}C NMR of **26** (50.32 MHz, $CDCl_3$)

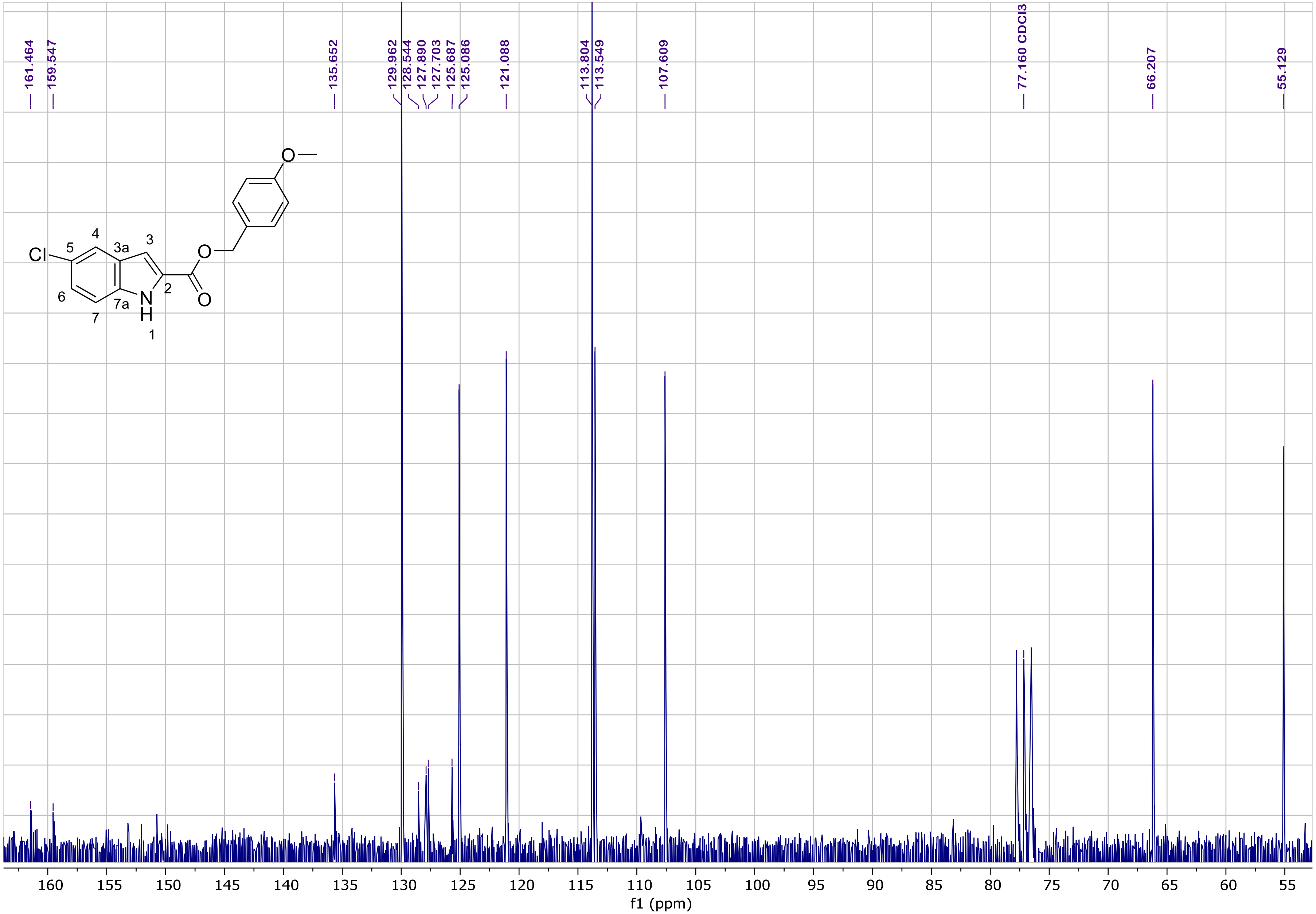

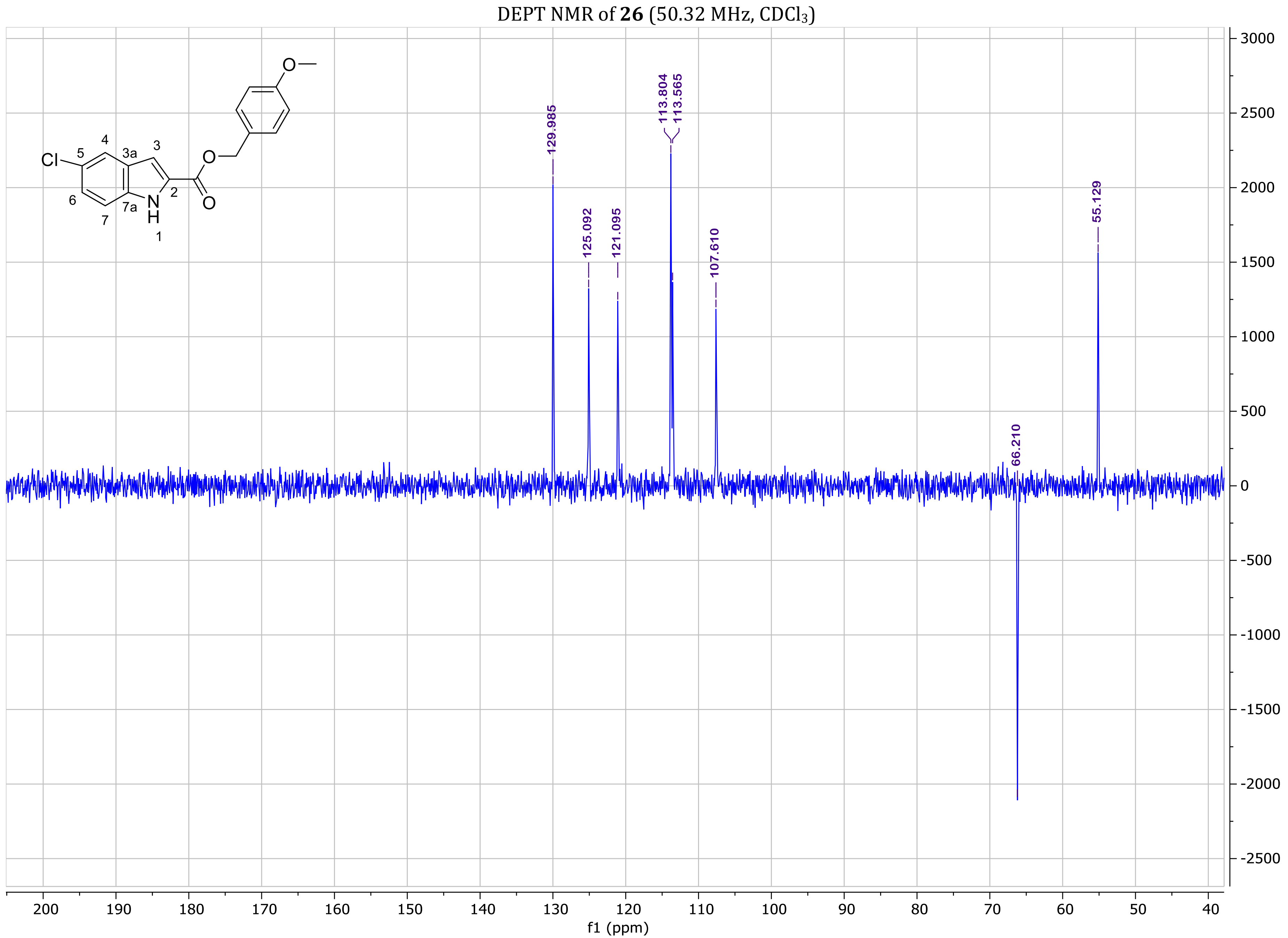
DEPT NMR of 26 (50.32 MHz, CDCl3)
Cl
5
4
3a
3
2
6
7
7a
N
H
1
O
O
O
129.985
125.092
121.095
113.804
113.565
107.610
66.210
55.129
f1 (ppm)

COSY NMR of **26** (400.13 MHz, $CDCl_3$)

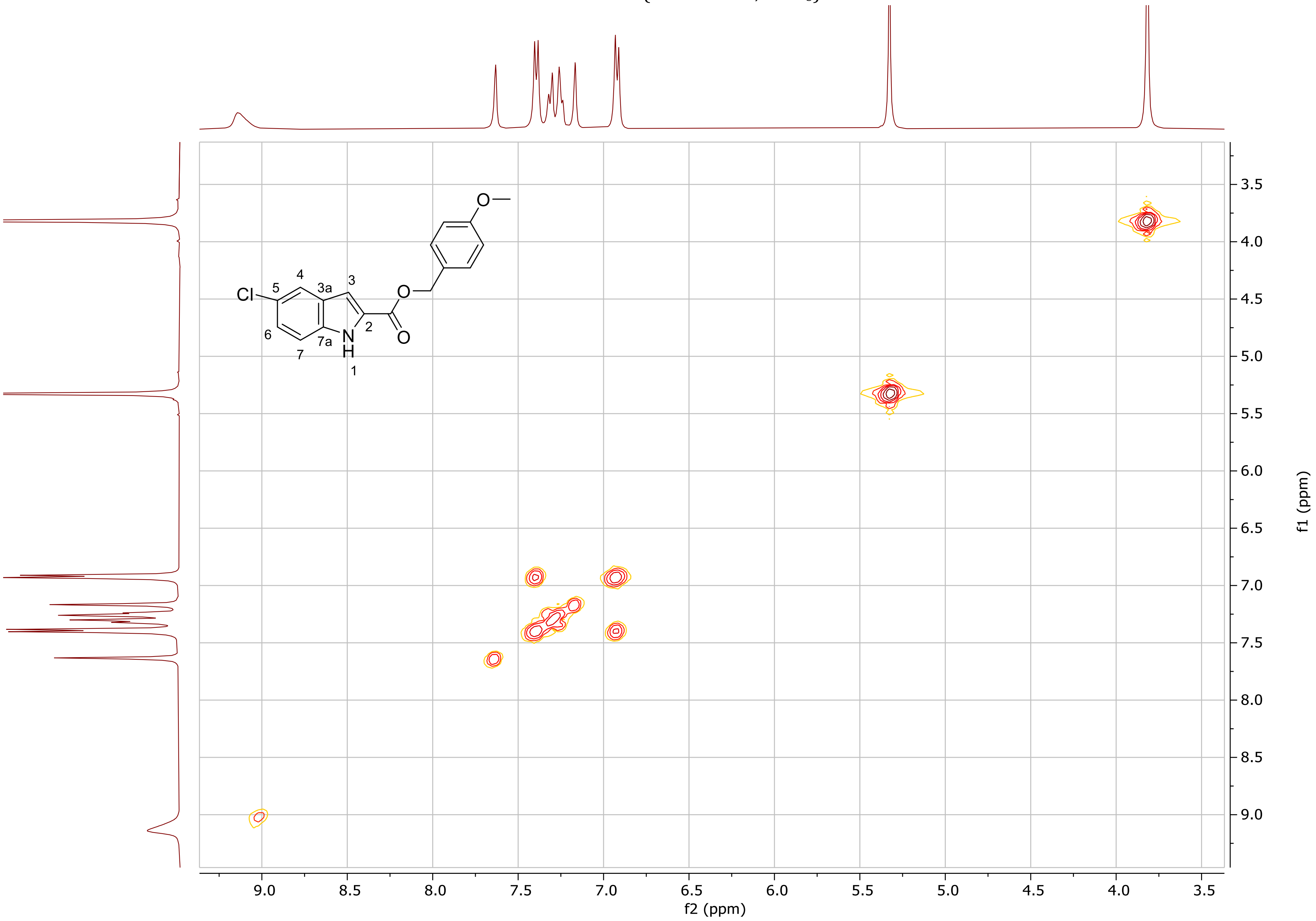

HSQC NMR of **26** (400.13 MHz, $CDCl_3$)

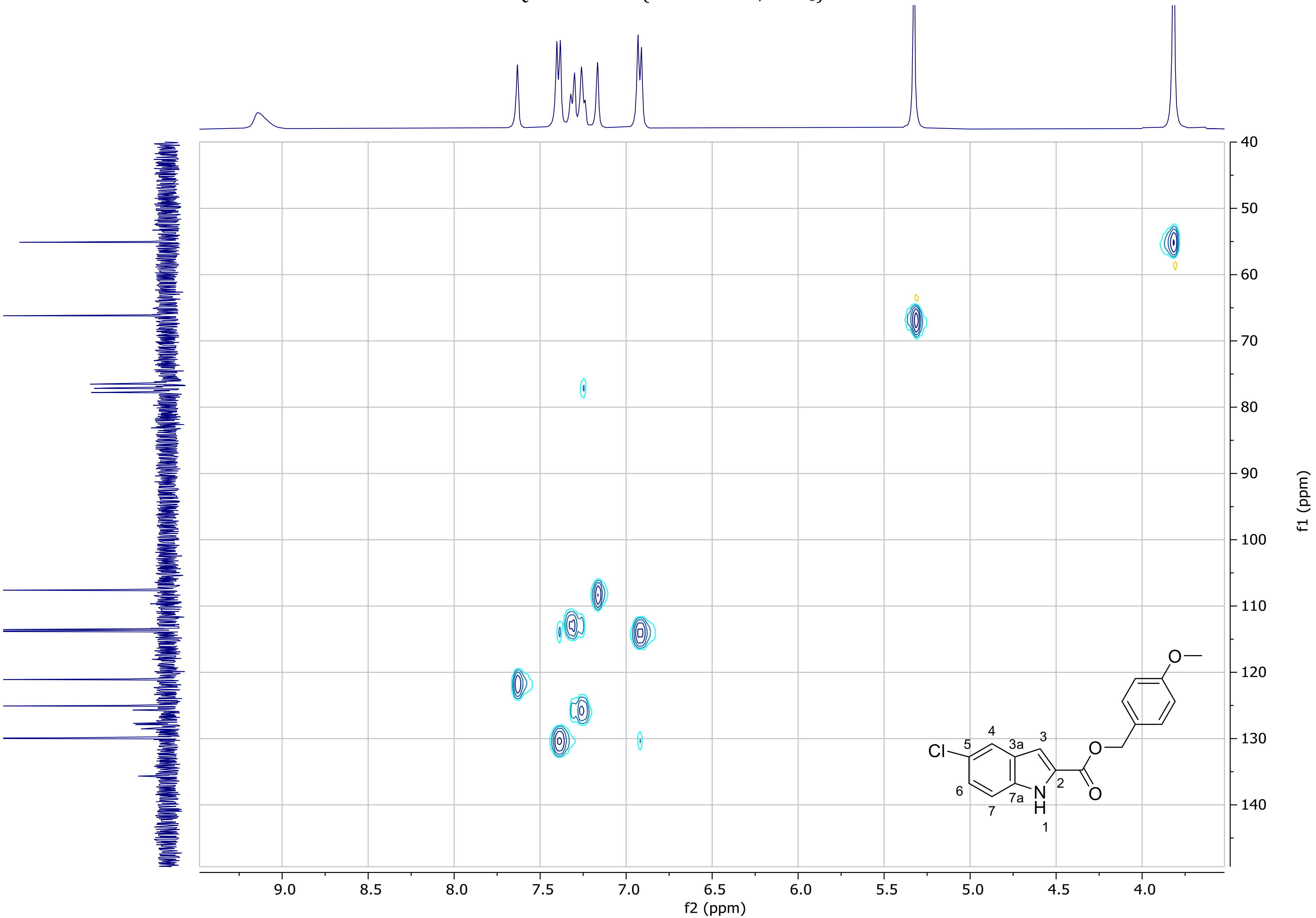

HMBC NMR of **26** (400.13 MHz, $CDCl_3$)

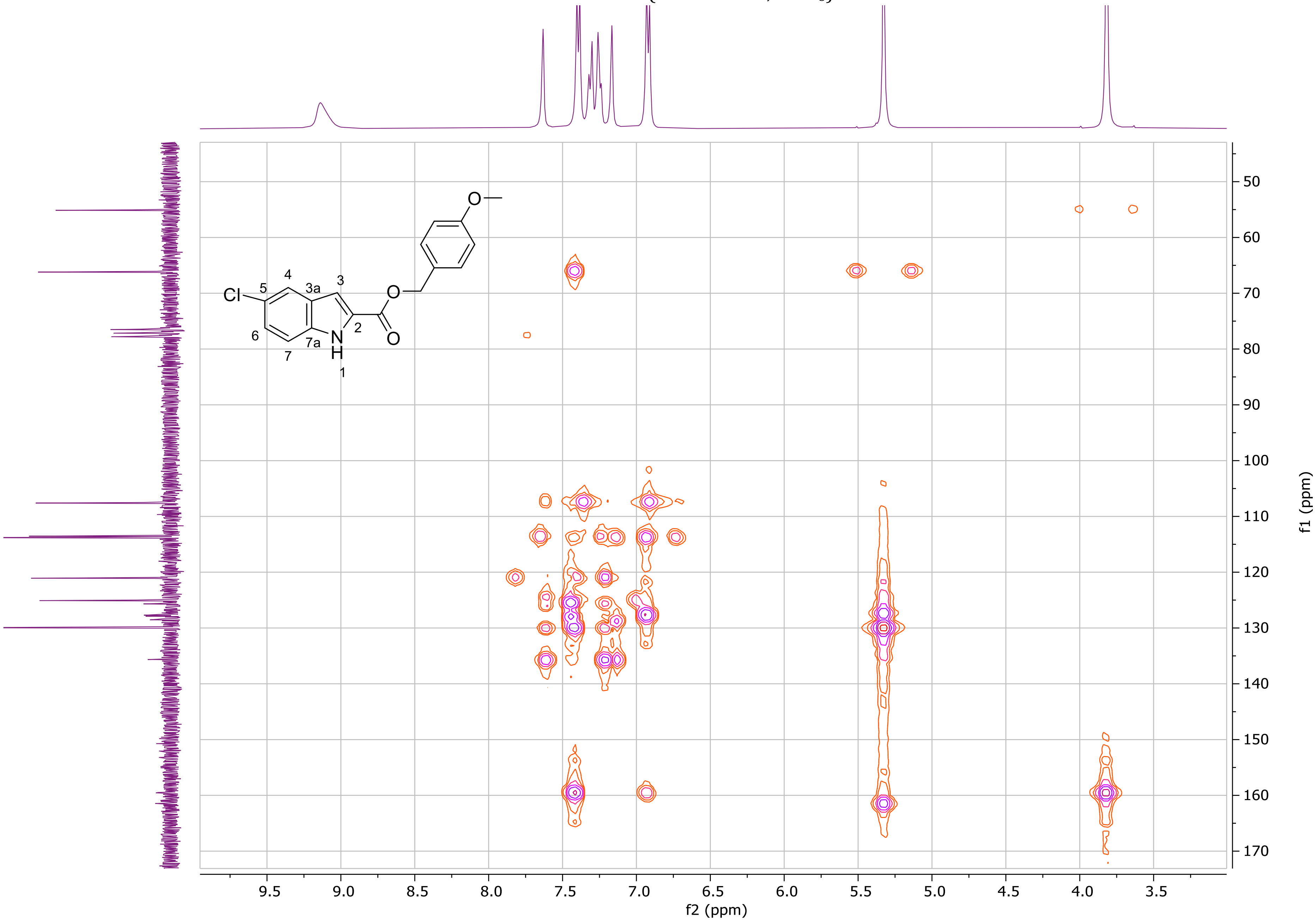

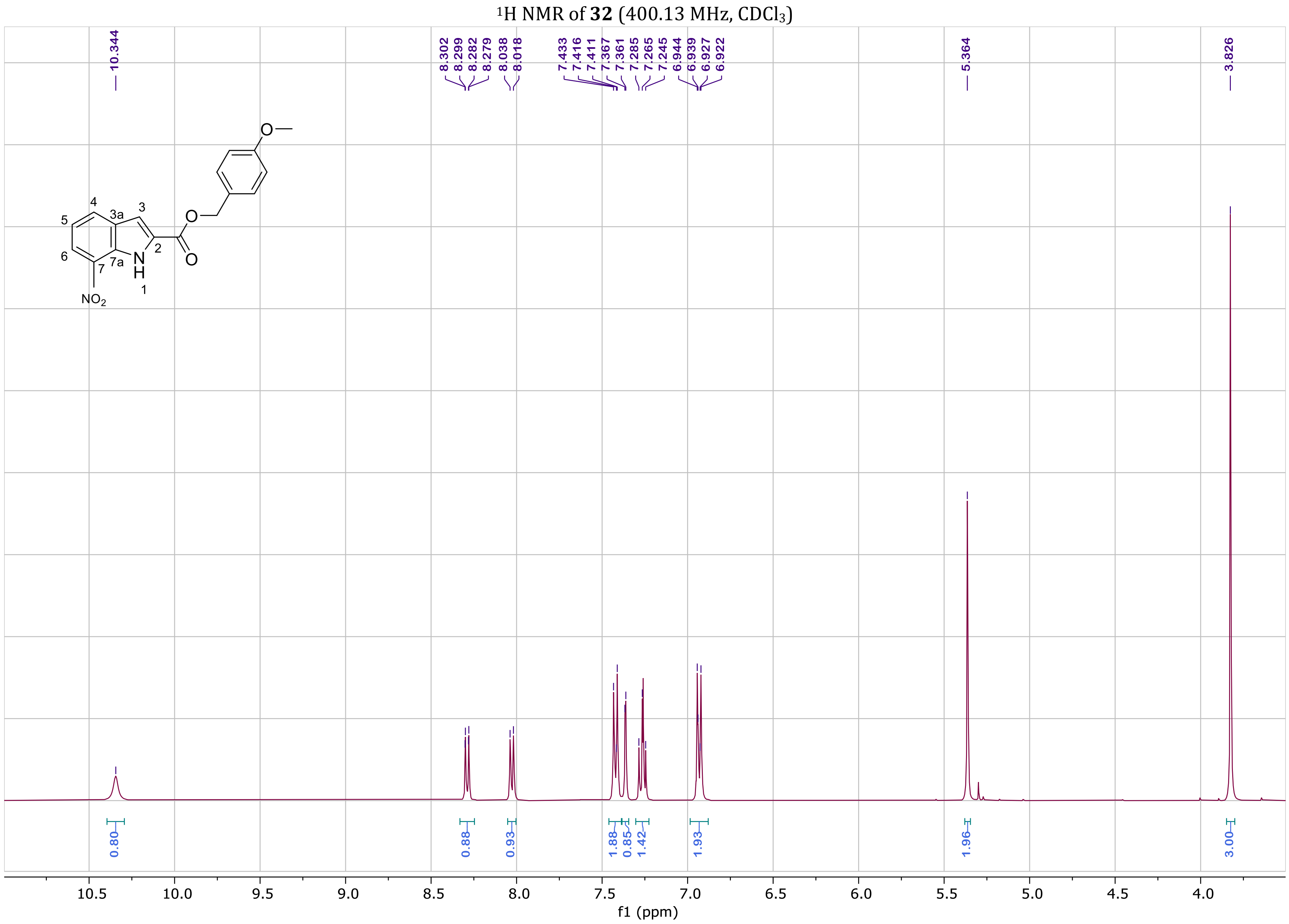
1H NMR of 32 (400.13 MHz, CDCl3)
10.344
8.302
8.299
8.282
8.279
8.038
8.018
7.433
7.416
7.411
7.367
7.361
7.285
7.265
7.245
6.944
6.939
6.927
6.922
5.364
3.826
0.80
0.88
0.93
1.88
0.85
1.42
1.93
1.96
3.00
10.5
10.0
9.5
9.0
8.5
8.0
7.5
7.0
6.5
6.0
5.5
5.0
4.5
4.0
f1 (ppm)
4
3
3a
5
2
6
7
7a
N
H
1
NO2
O
O
O

^{13}C NMR of **32** (50.32 MHz, $CDCl_3$)

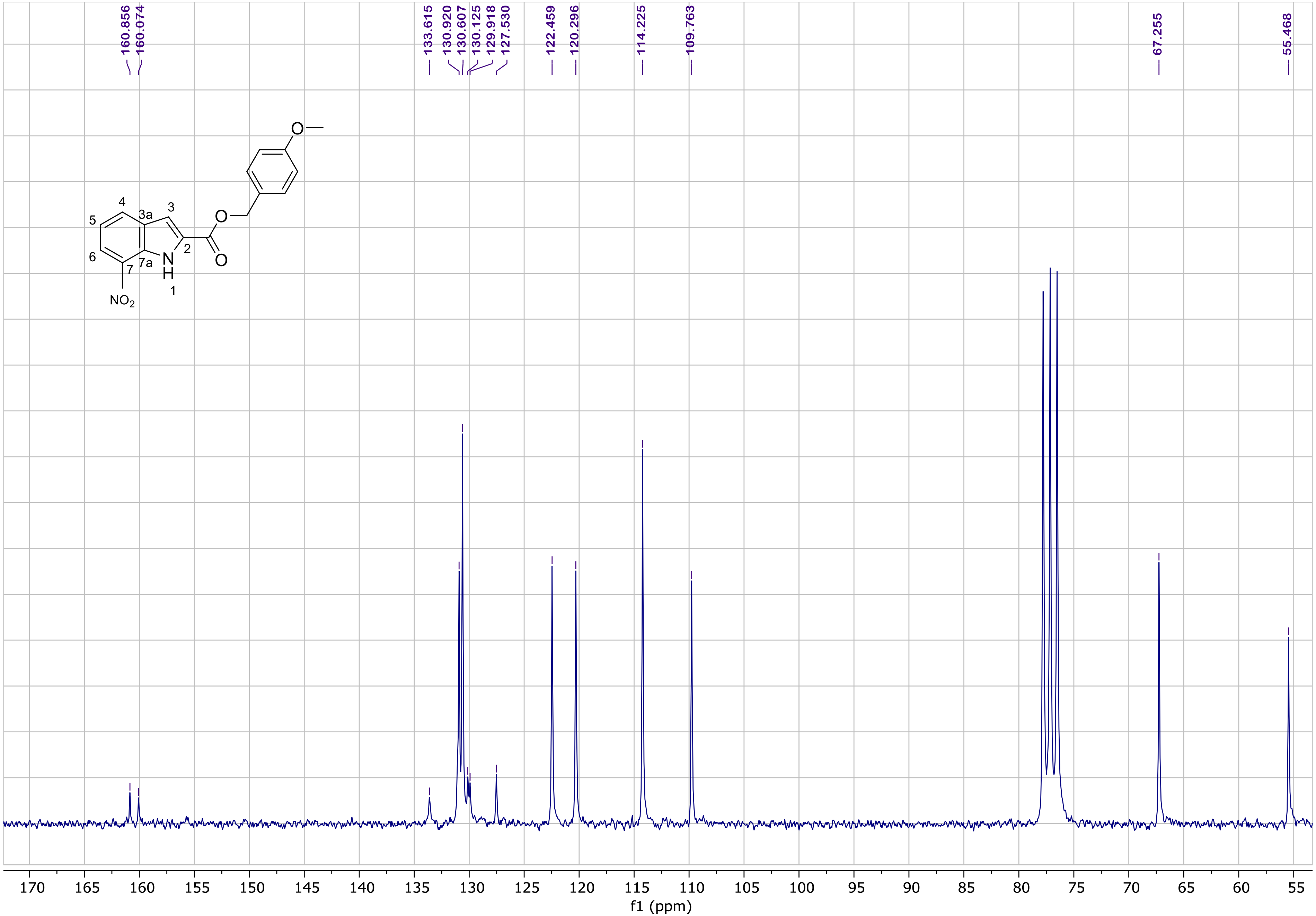

DEPT NMR of **32** (50.32 MHz, $CDCl_3$)

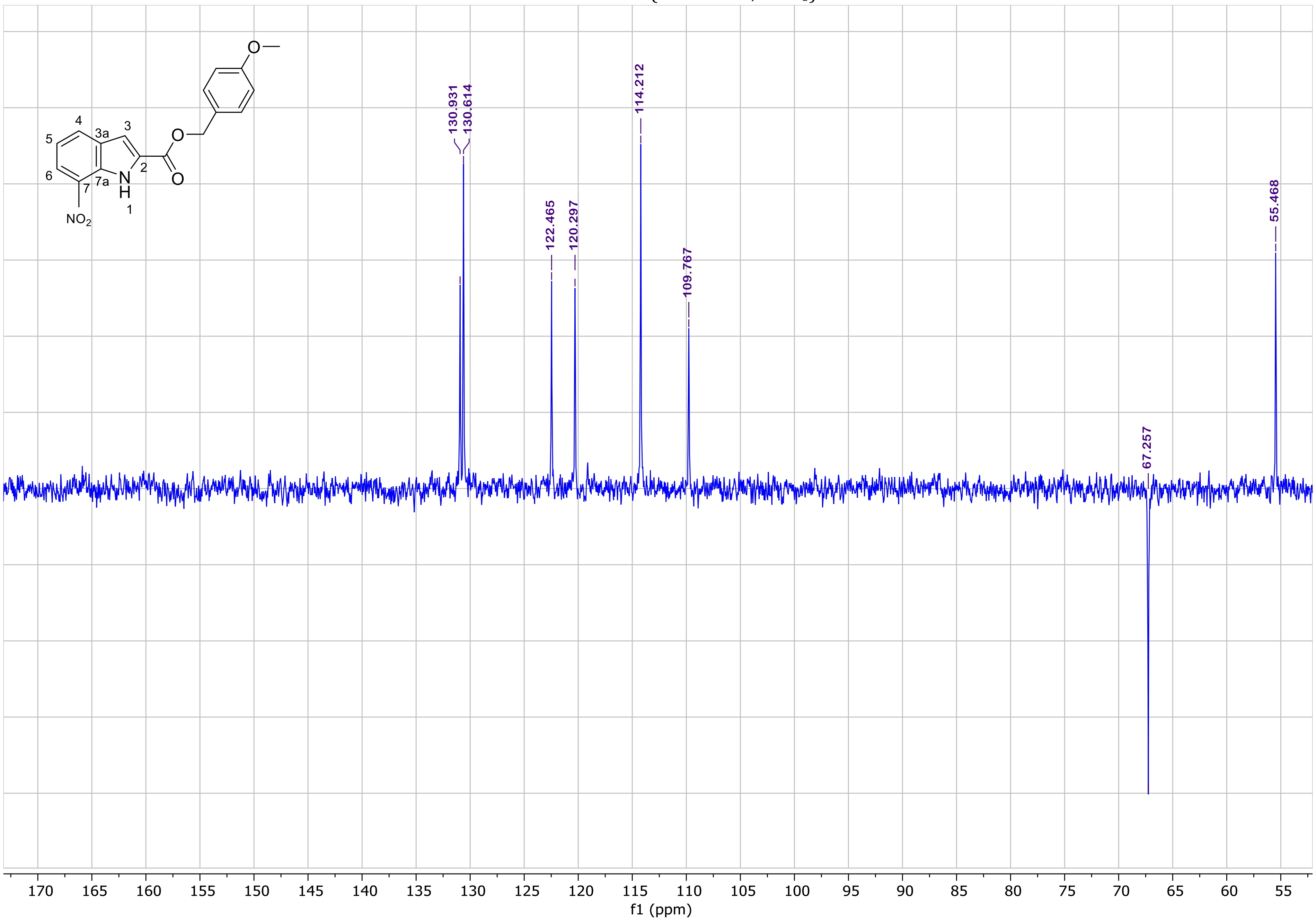

COSY NMR of **32** (400.13 MHz, $CDCl_3$)

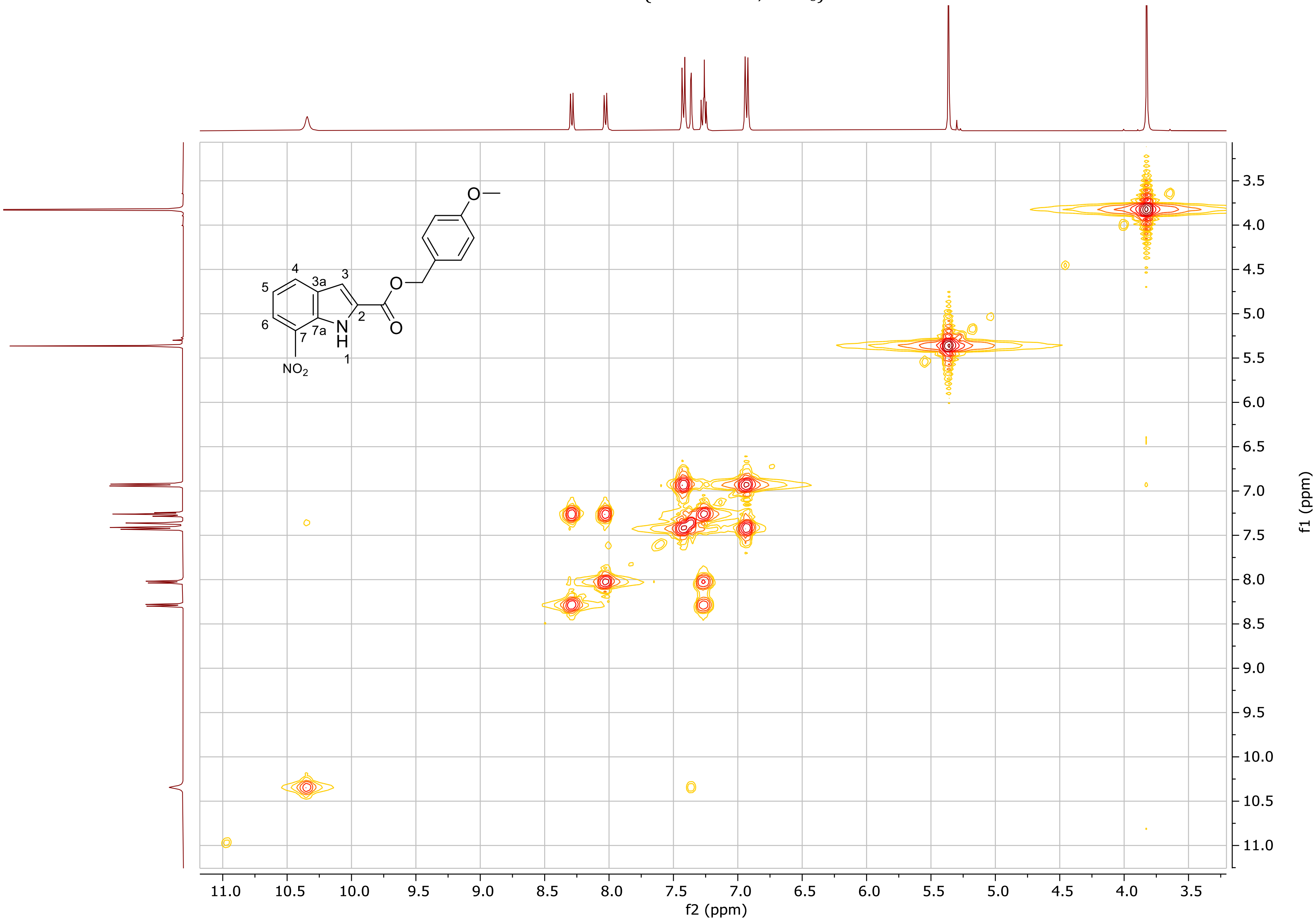

HSQC NMR of **32** (400.13 MHz, $CDCl_3$)

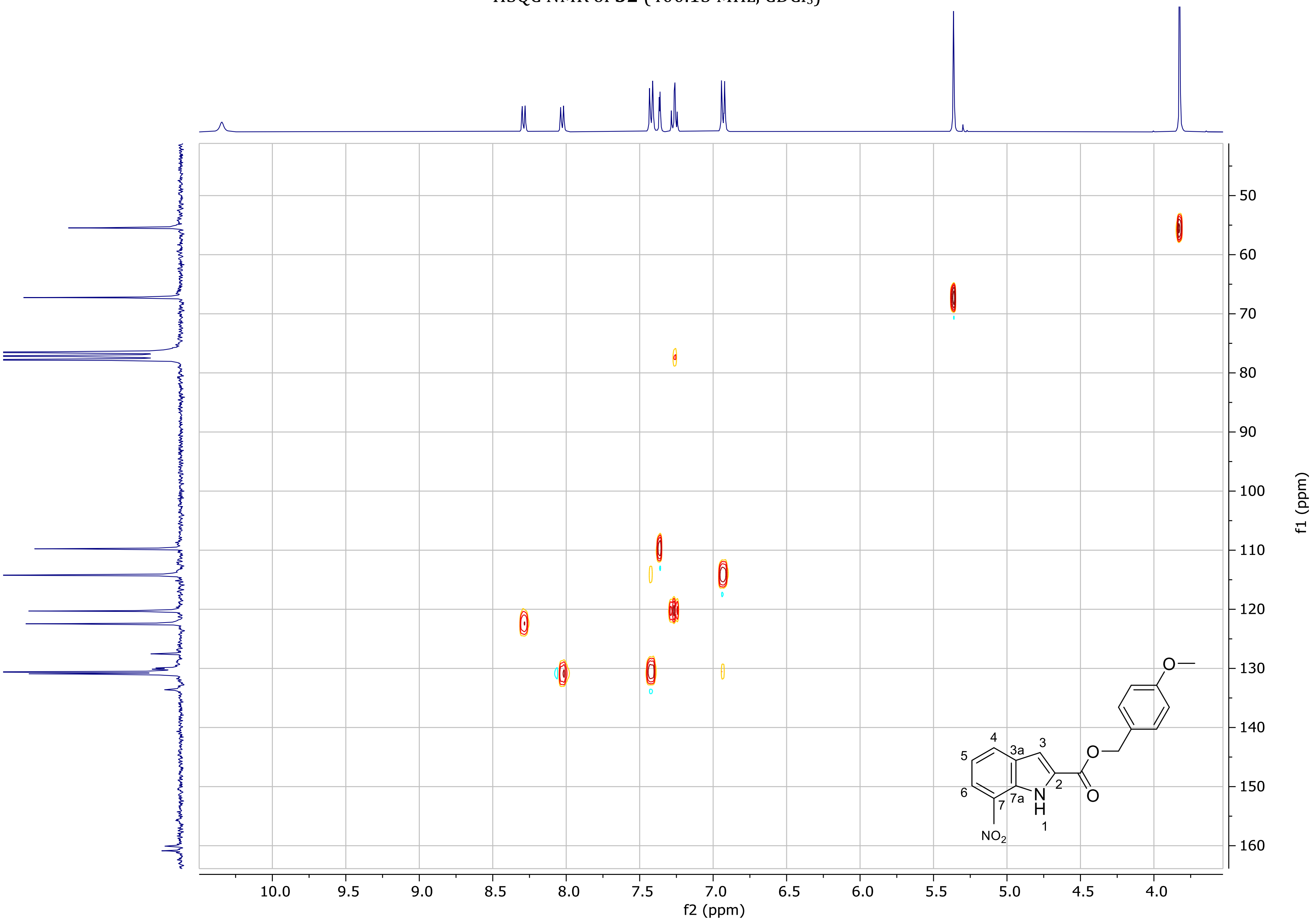

HMBC NMR of **32** (400.13 MHz, $CDCl_3$)

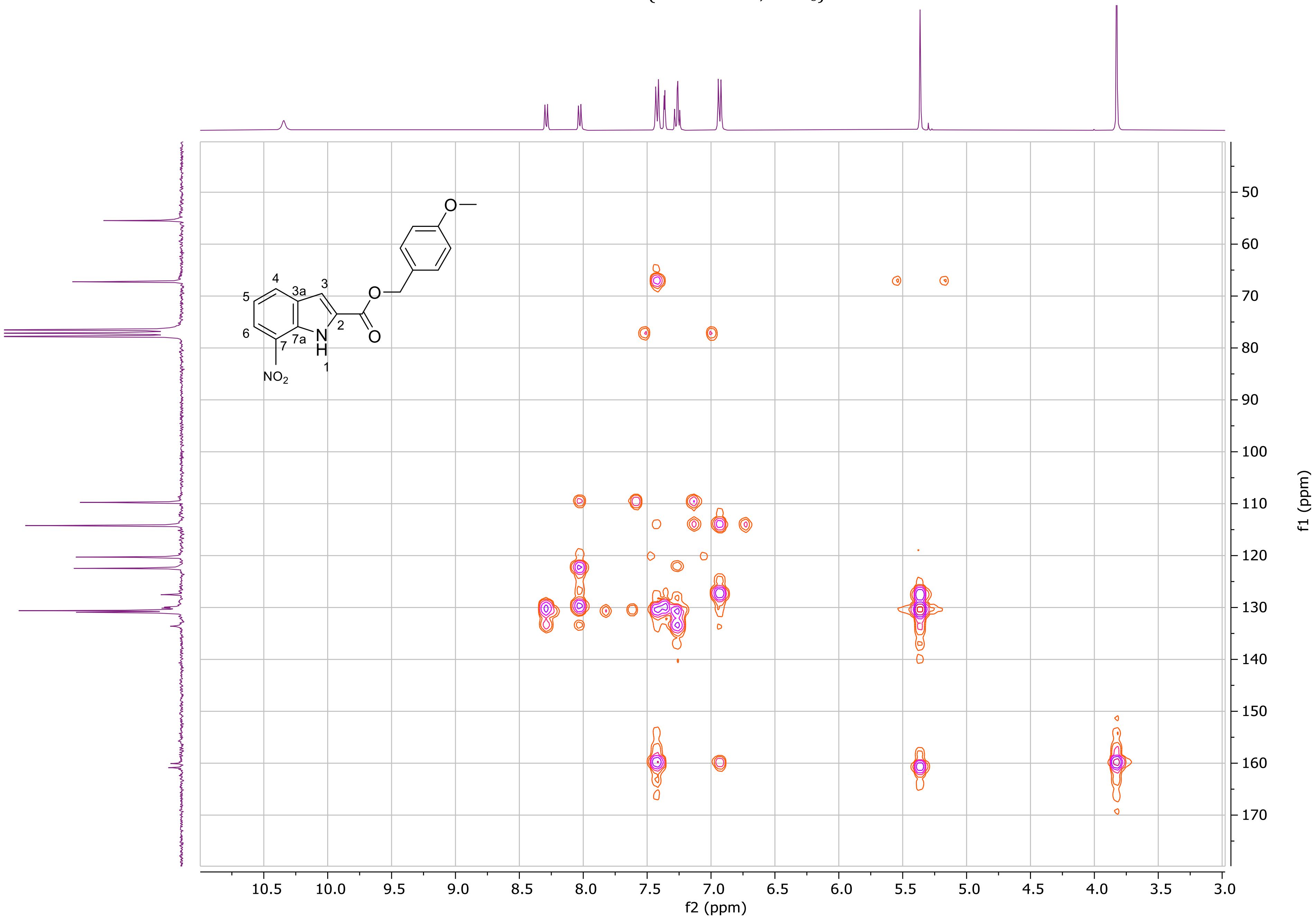

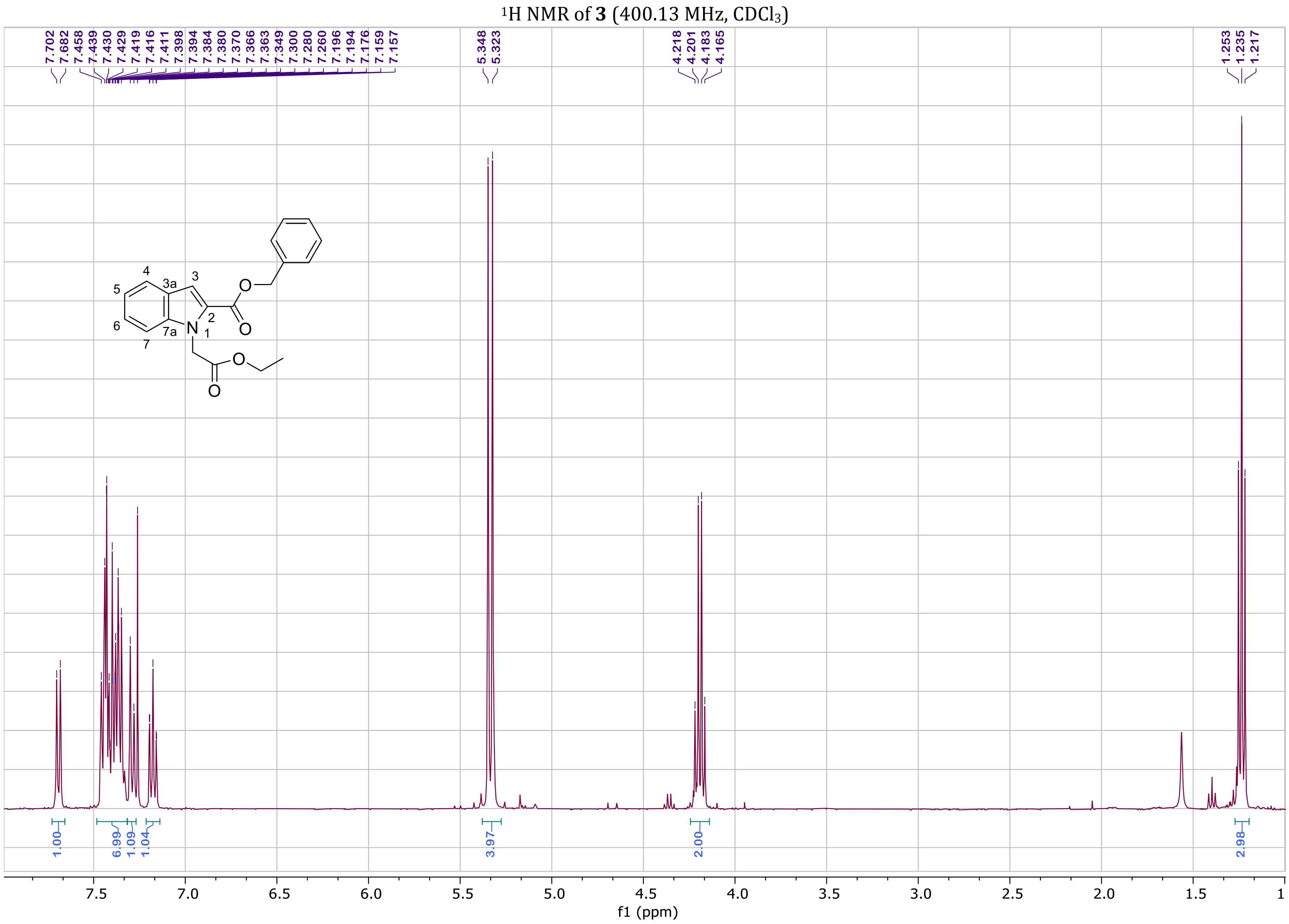

¹H NMR of 3 (400.13 MHz, CDCl₃)
7.702
7.682
7.458
7.439
7.430
7.429
7.419
7.416
7.411
7.398
7.394
7.384
7.380
7.370
7.366
7.363
7.349
7.300
7.280
7.260
7.196
7.194
7.176
7.159
7.157
5.348
5.323
4.218
4.201
4.183
4.165
1.253
1.235
1.217
4
3
3a
5
O
2
6
7a
N
1
O
7
O
O
1.00
6.99
1.09
1.04
3.97
2.00
2.98
7.5
7.0
6.5
6.0
5.5
5.0
4.5
4.0
3.5
3.0
2.5
2.0
1.5
1
f1 (ppm)

^{13}C NMR of **3** (50.32 MHz, $CDCl_3$)

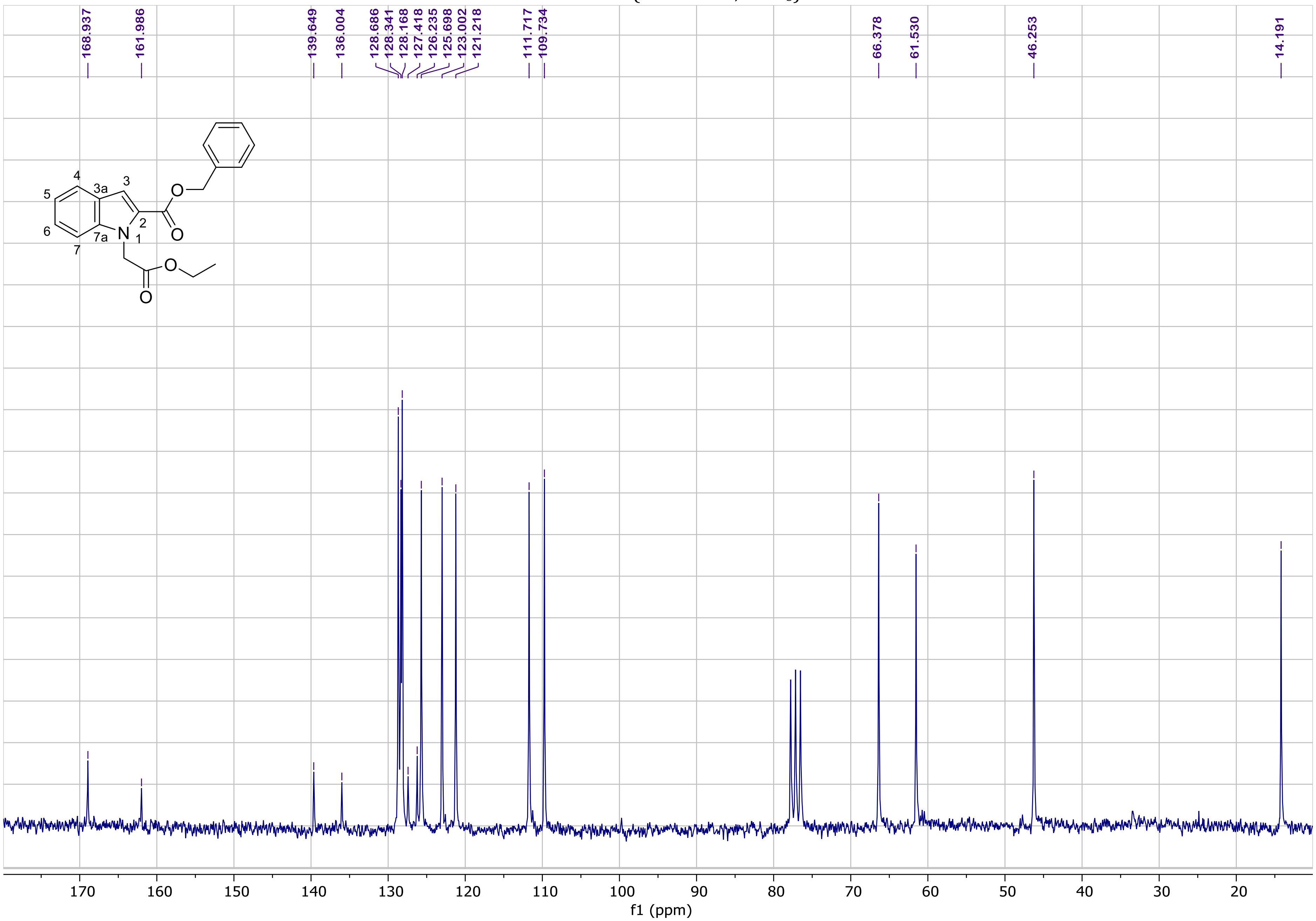

COSY NMR of **3** (400.13 MHz, $CDCl_3$)

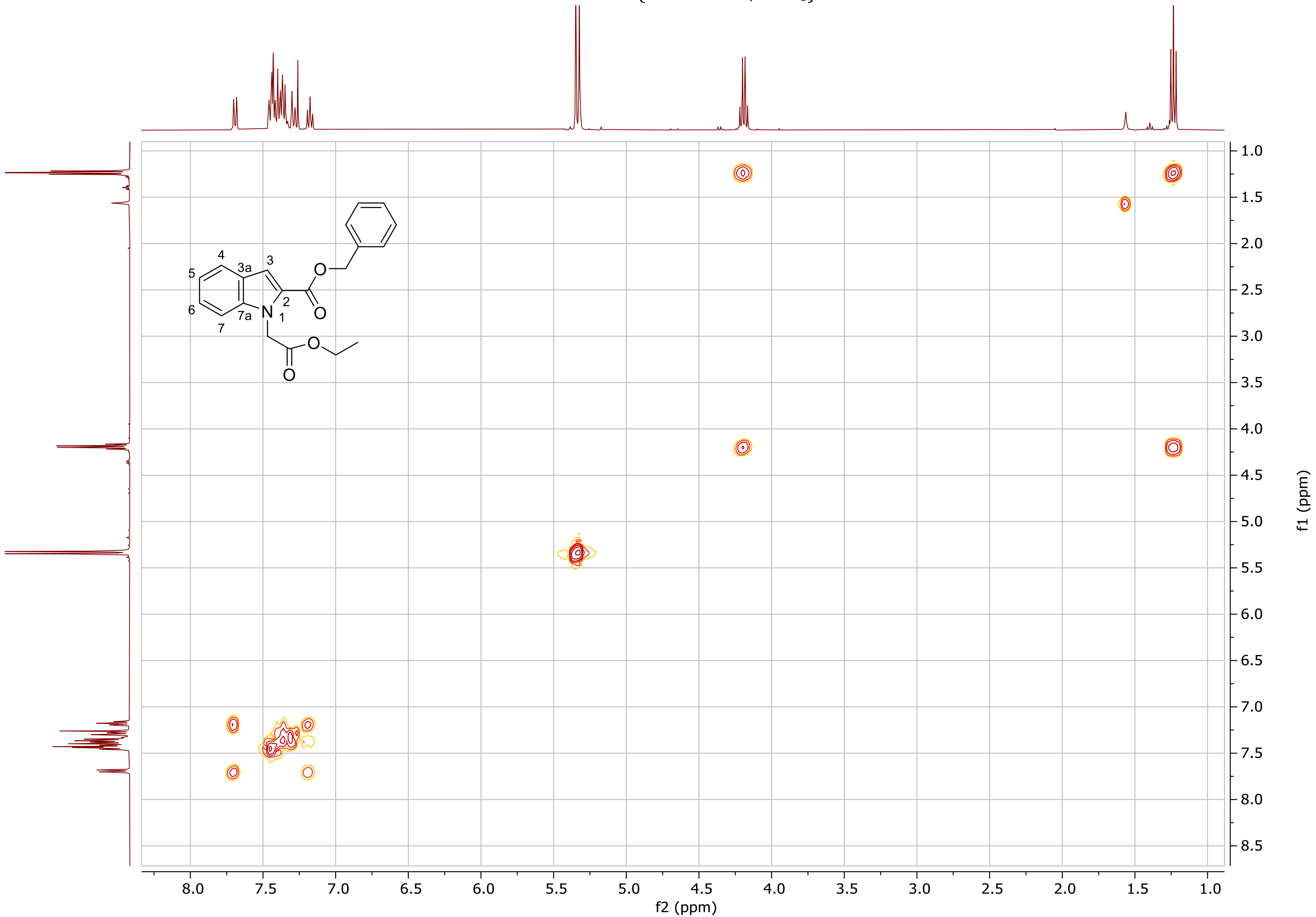

HSQC NMR of **3** (400.13 MHz, $CDCl_3$)

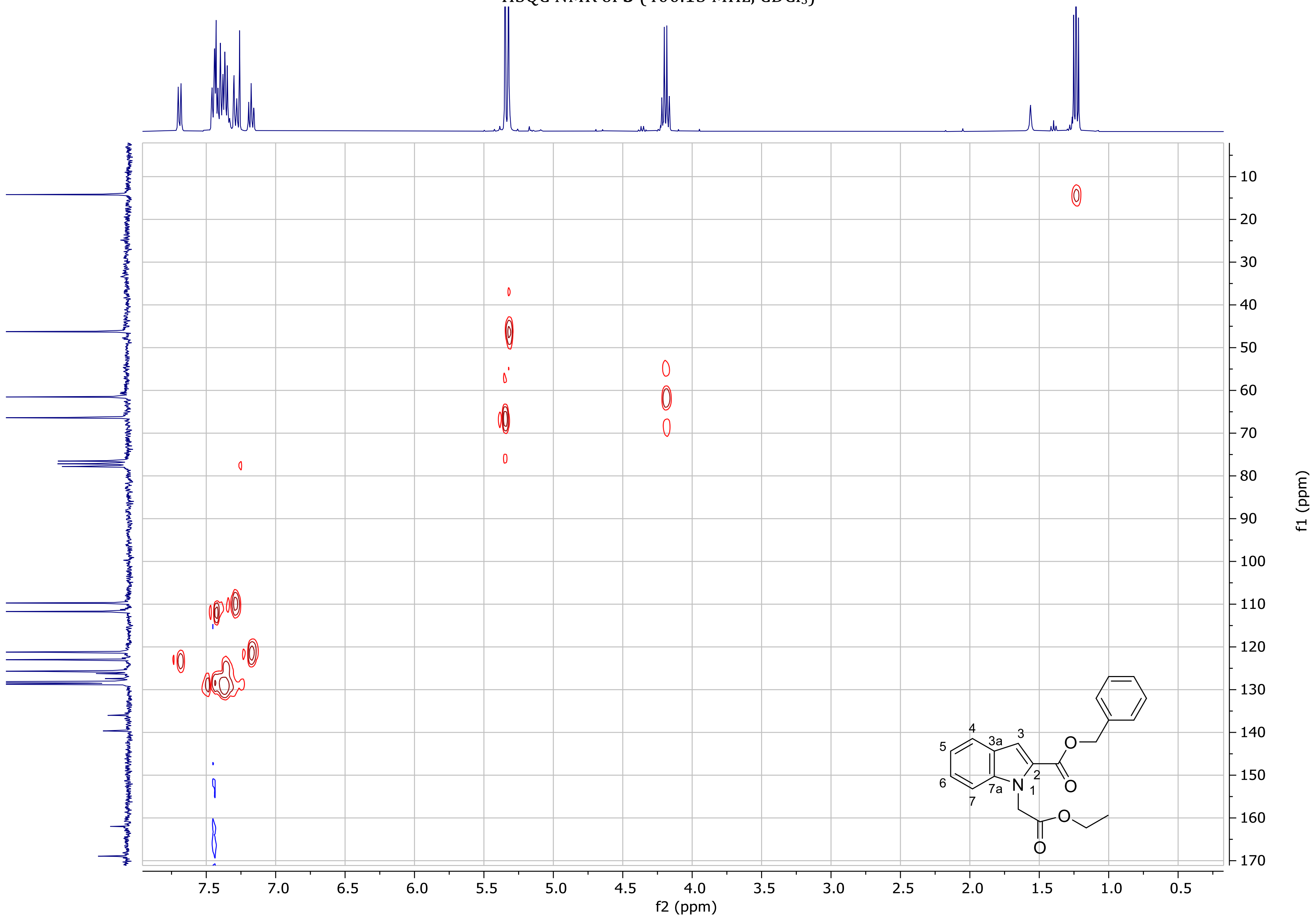

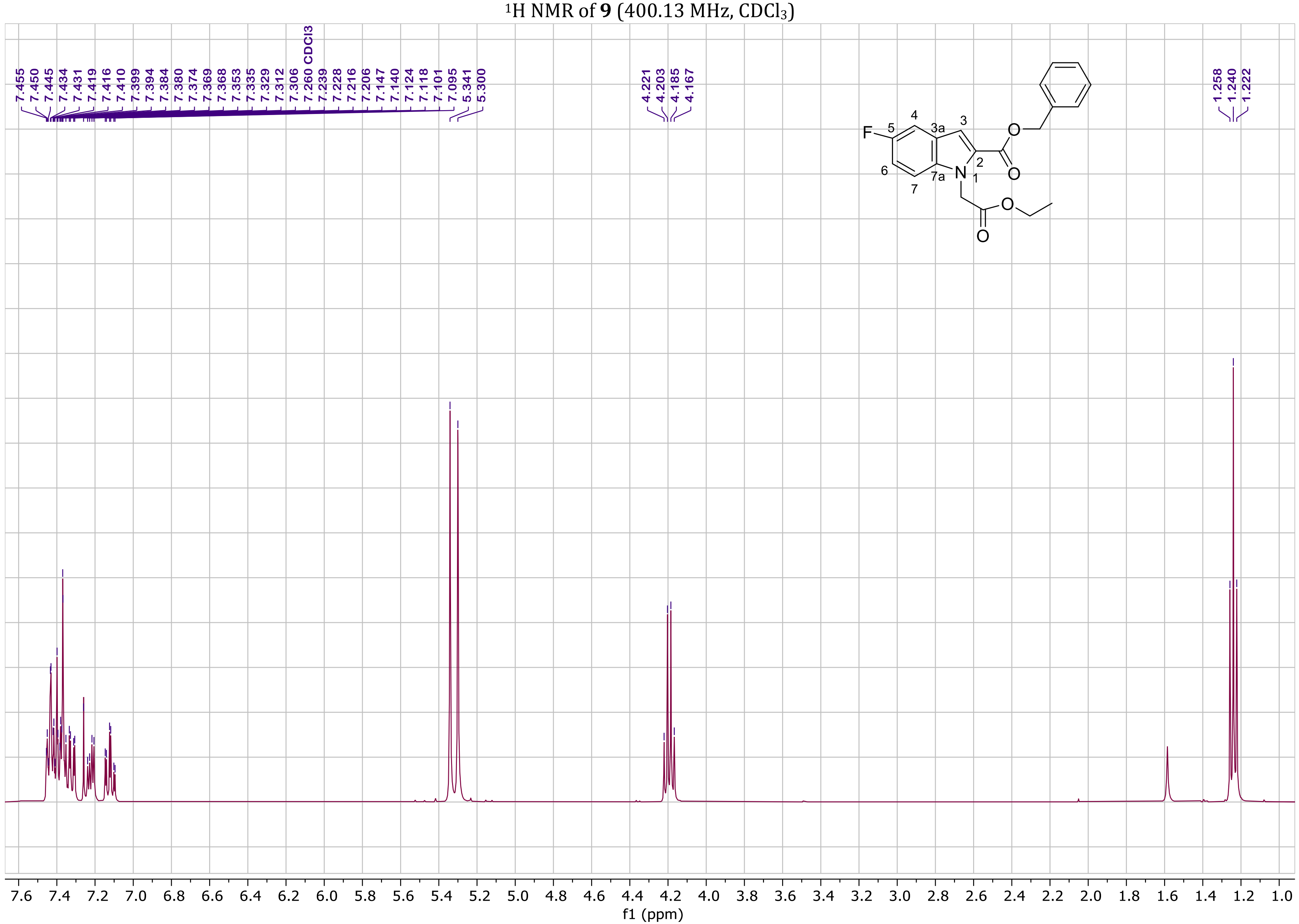
^{1}H NMR of **9** (400.13 MHz, CDCl$_3$)
7.455 7.450 7.445 7.434 7.431 7.419 7.416 7.410 7.399 7.394 7.384 7.380 7.374 7.369 7.368 7.353 7.335 7.329 7.312 7.306 7.260 CDCl3 7.239 7.228 7.216 7.206 7.147 7.140 7.124 7.118 7.101 7.095 5.341 5.300
4.221 4.203 4.185 4.167
1.258 1.240 1.222
f1 (ppm)

^{13}C NMR of **9** (50.32 MHz, CDCl$_3$)

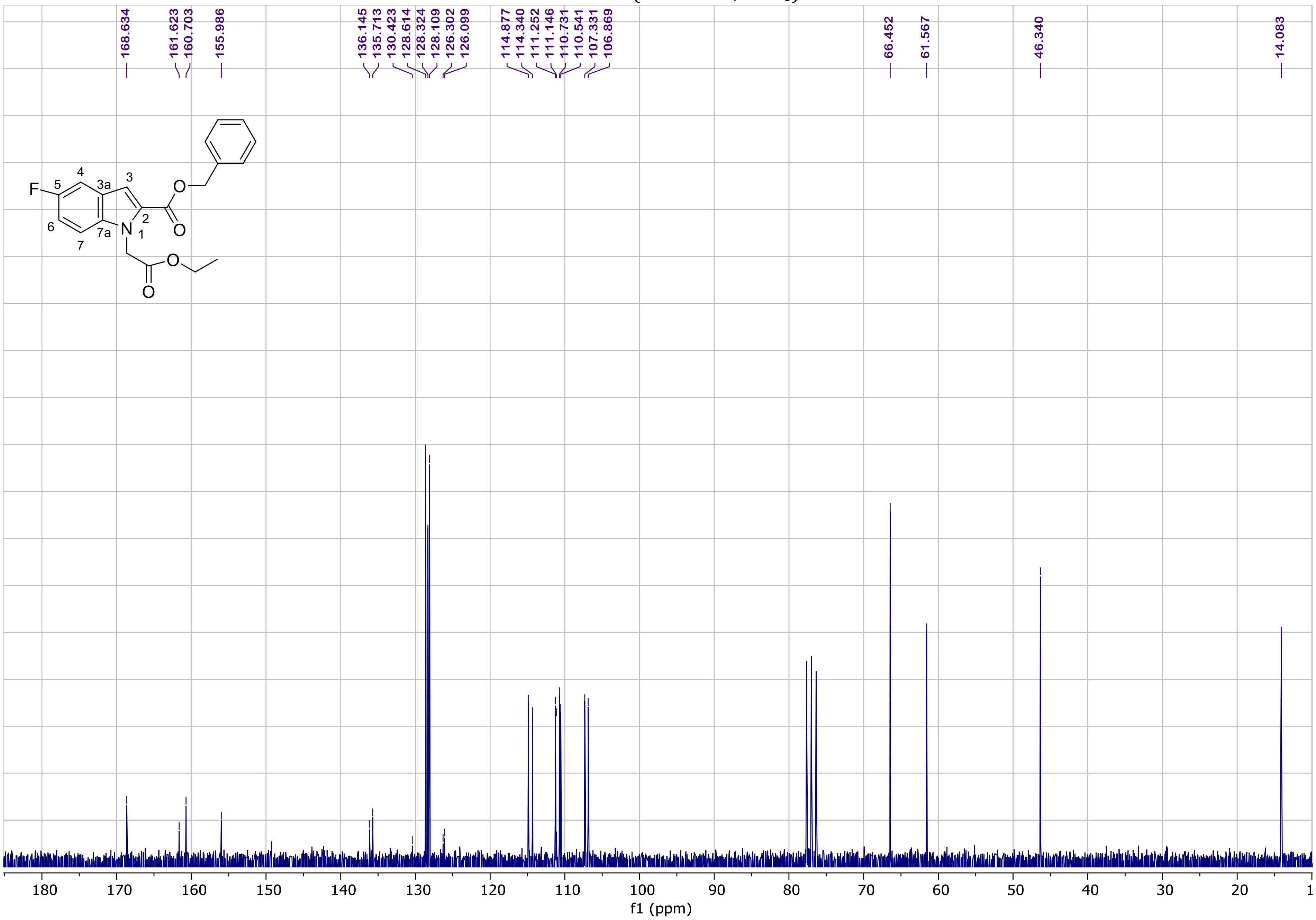

DEPT NMR of **9** (50.32 MHz, $CDCl_3$)

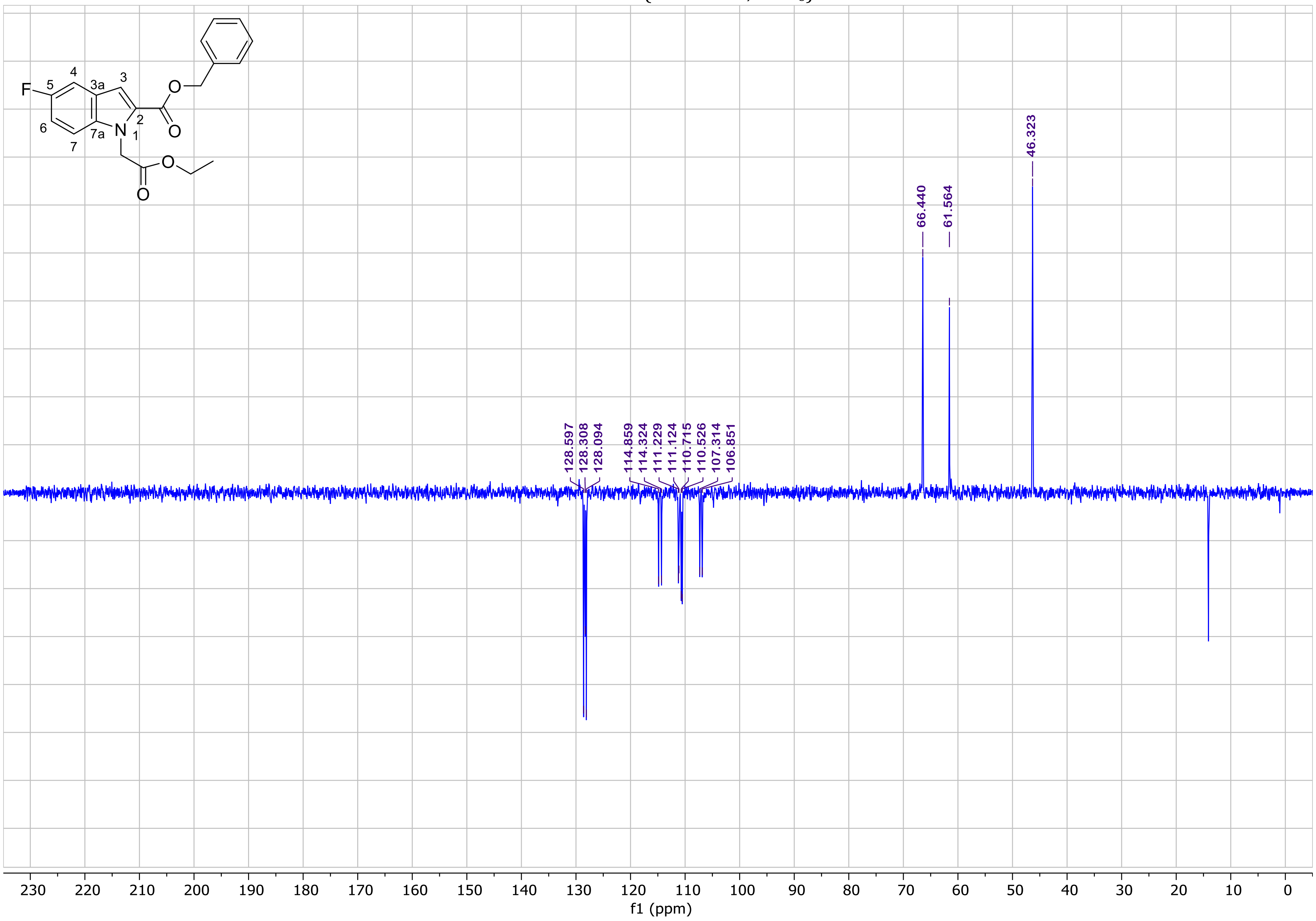

COSY NMR of **9** (400.13 MHz, $CDCl_3$)

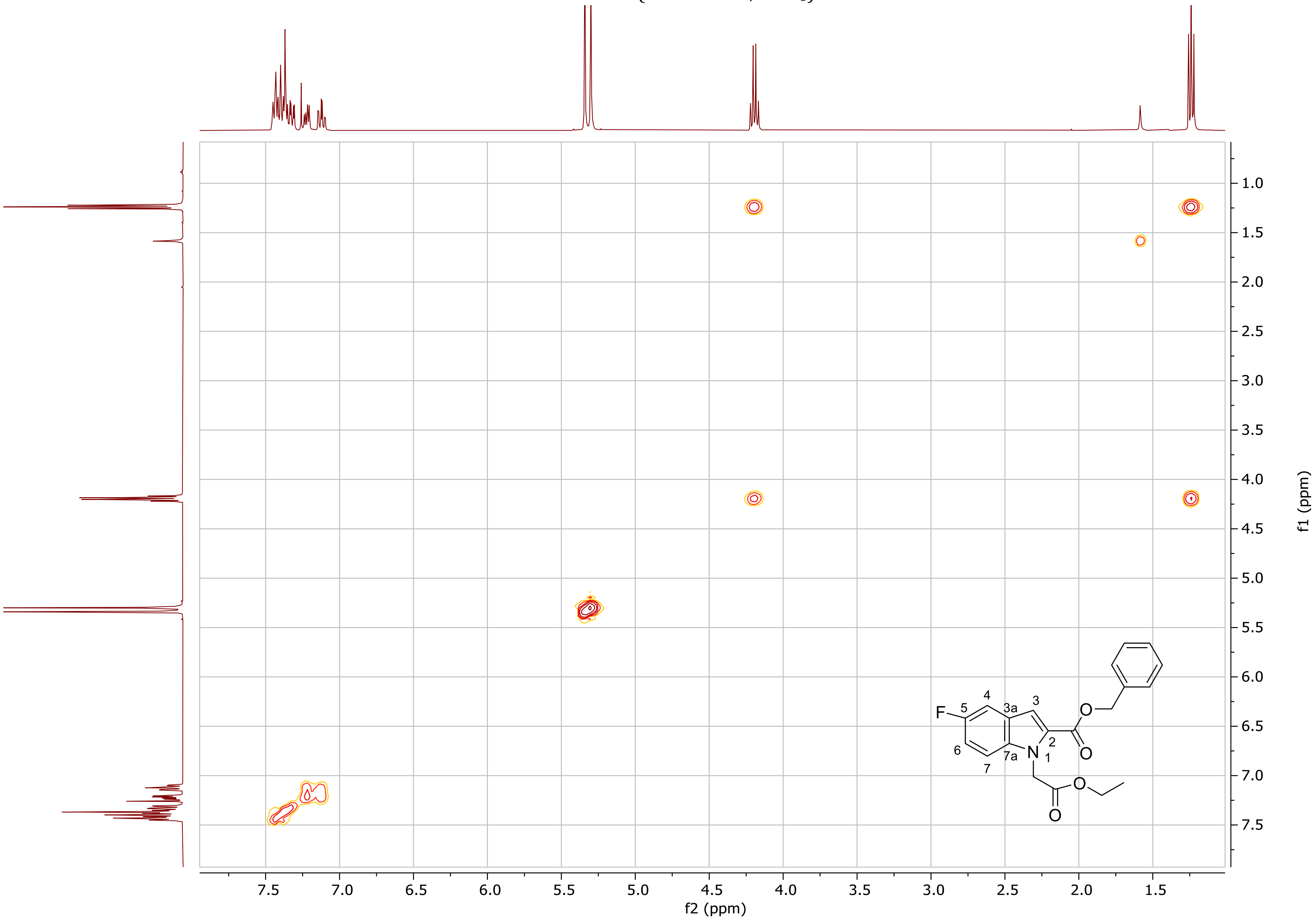

HSQC NMR of **9** (400.13 MHz, $CDCl_3$)

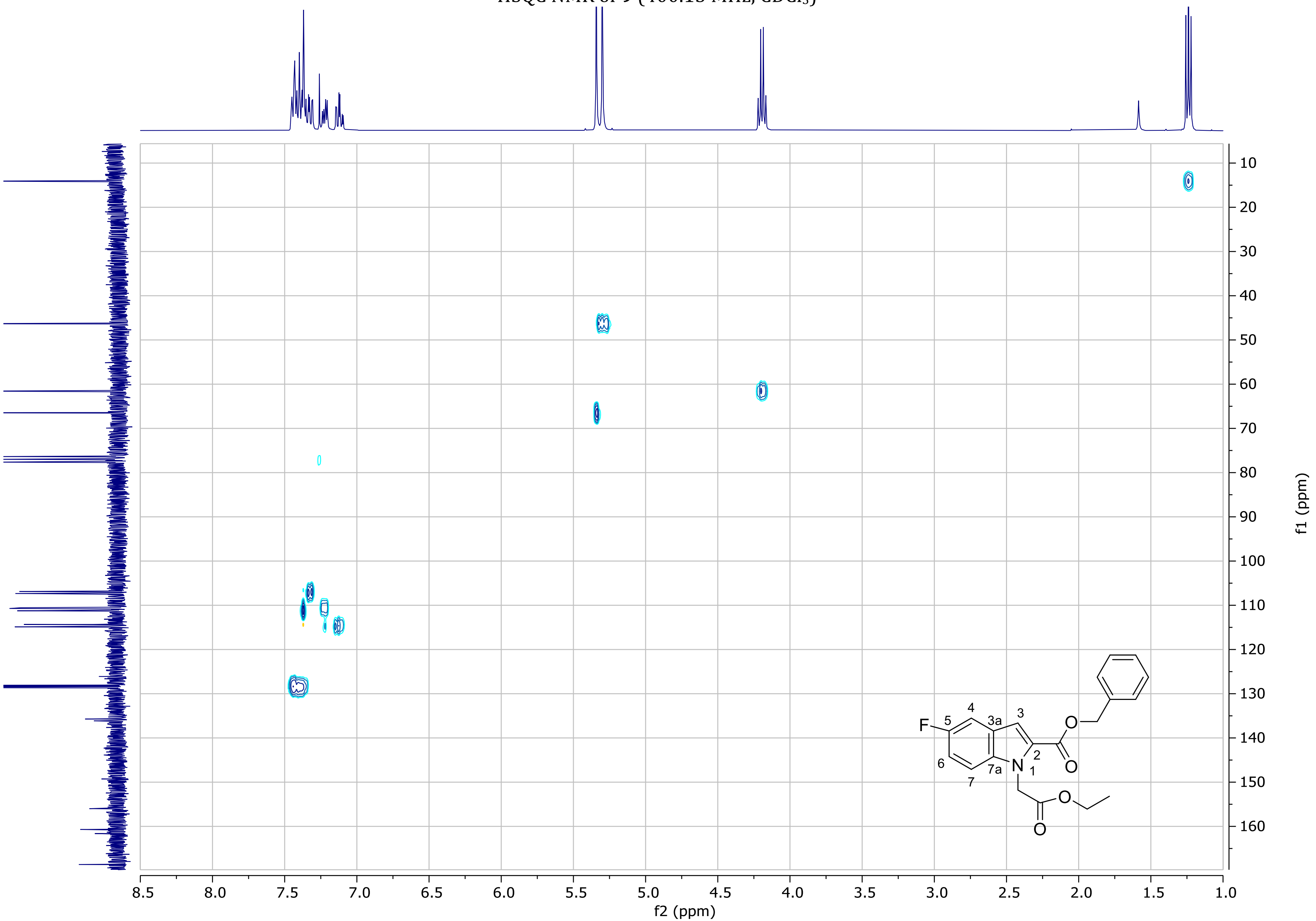

HMBC NMR of **9** (400.13 MHz, $CDCl_3$)

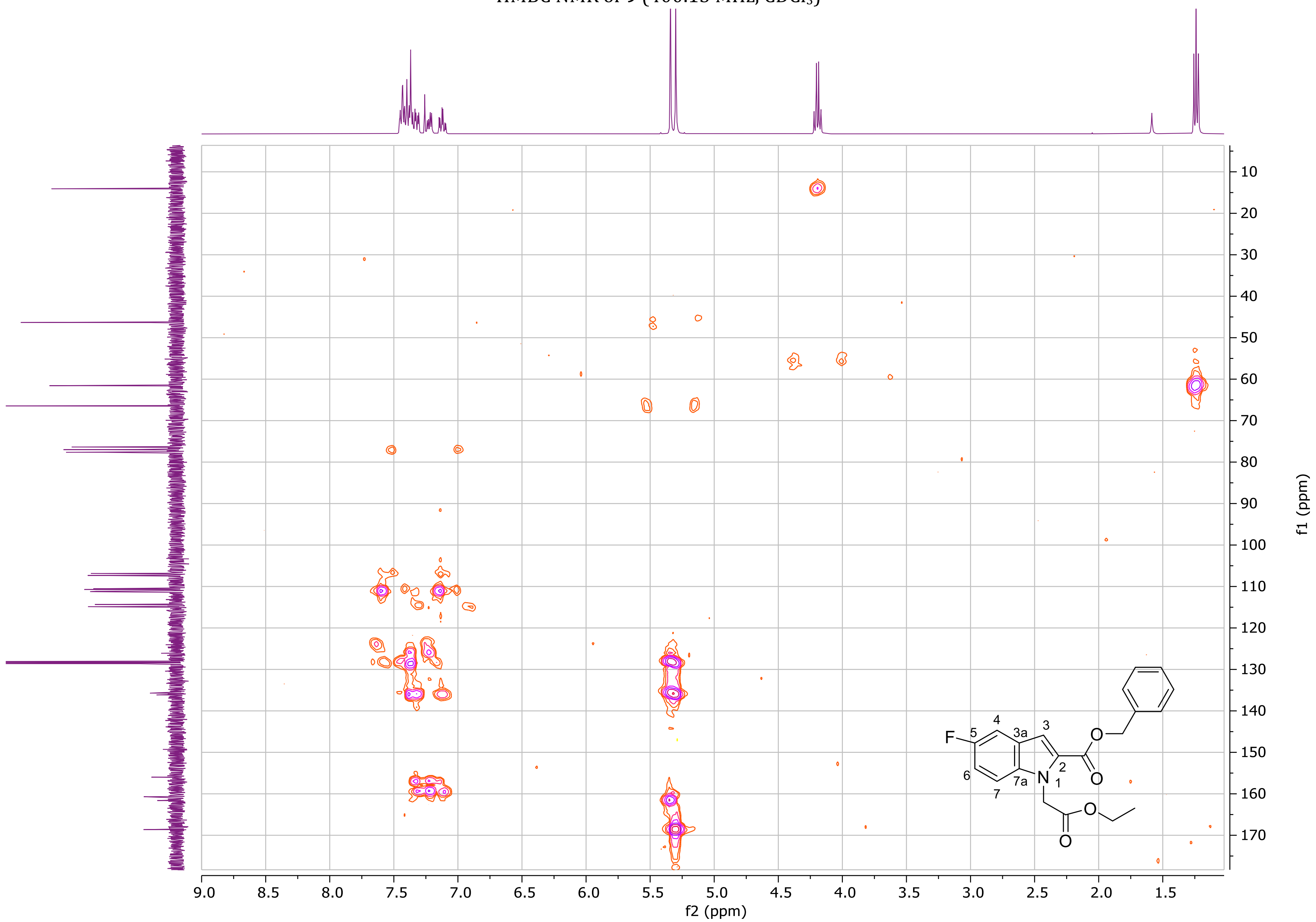

^{1}H NMR of **15** (400.13 MHz, $CDCl_3$)

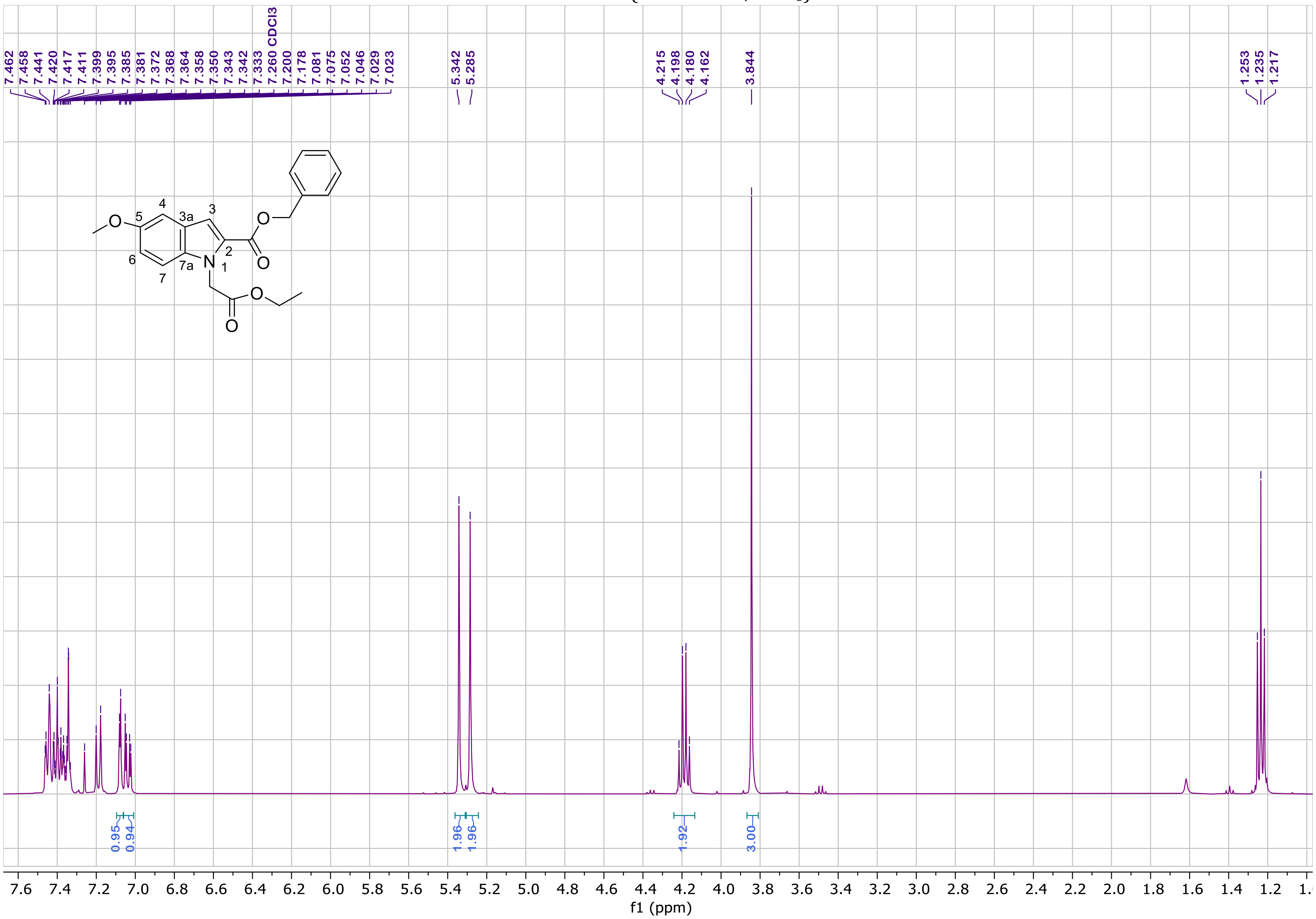

^{13}C NMR of **15** (50.32 MHz, $CDCl_3$)

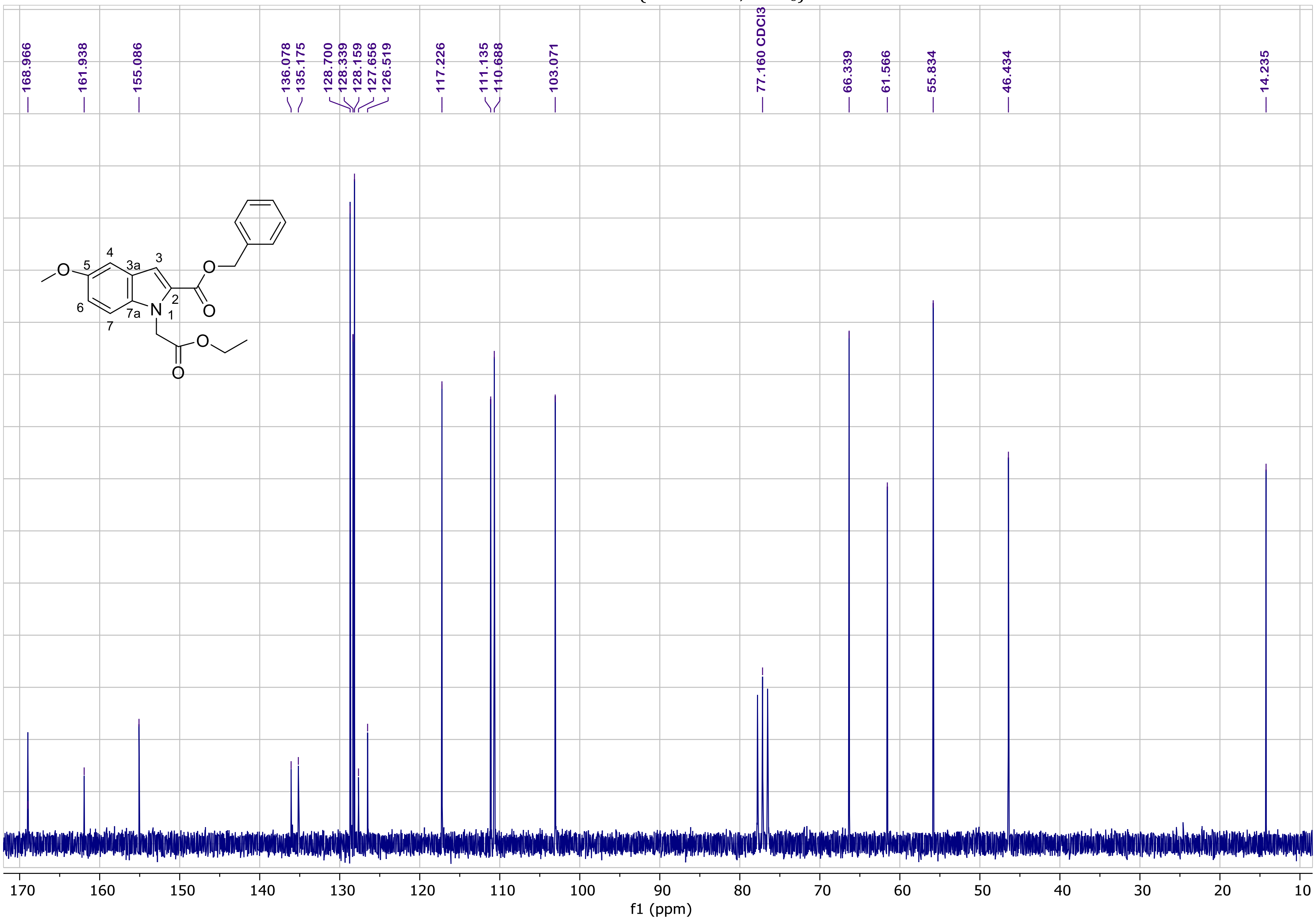

COSY NMR of **15** (400.13 MHz, $CDCl_3$)

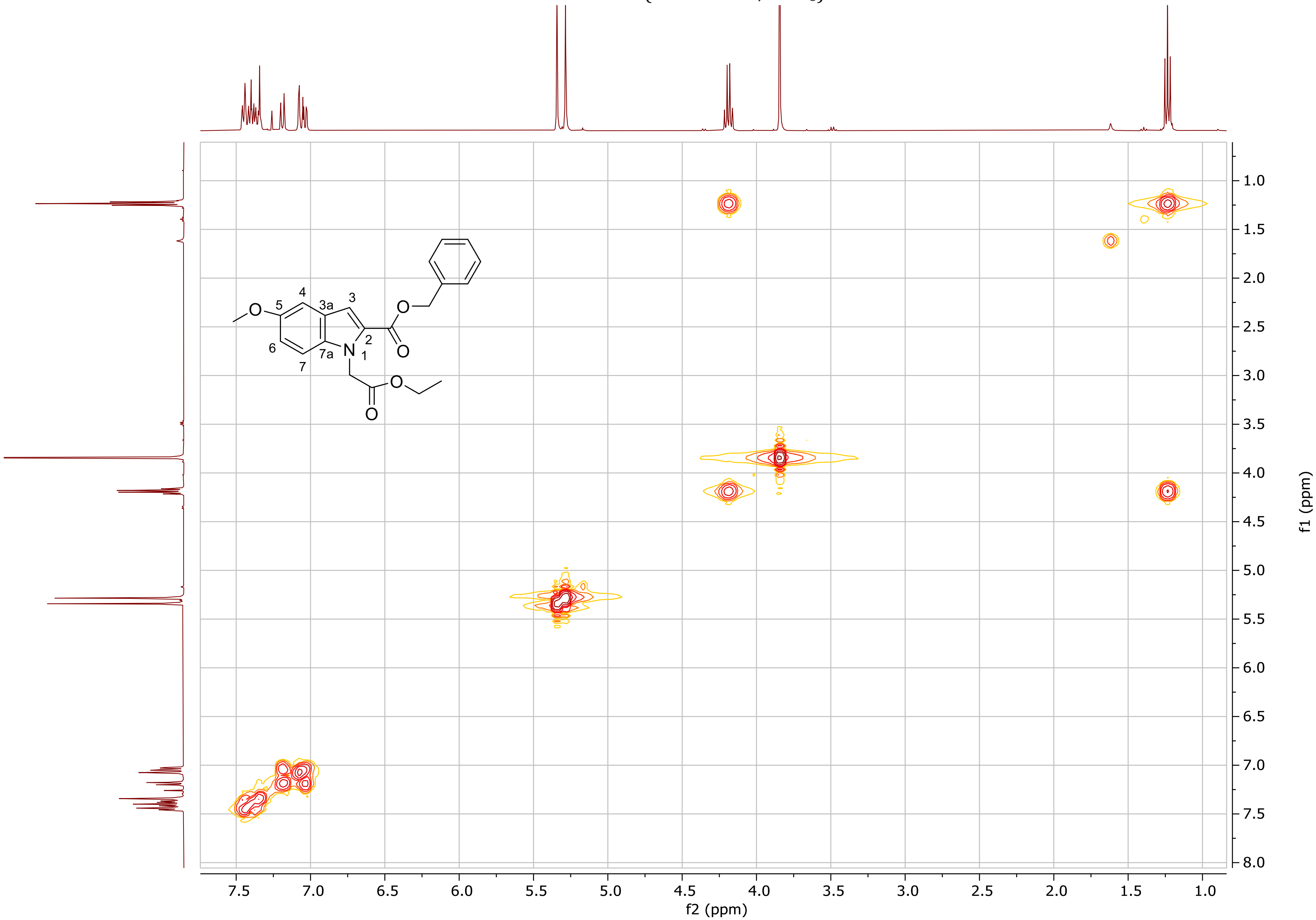

HSQC NMR of **15** (400.13 MHz, $CDCl_3$)

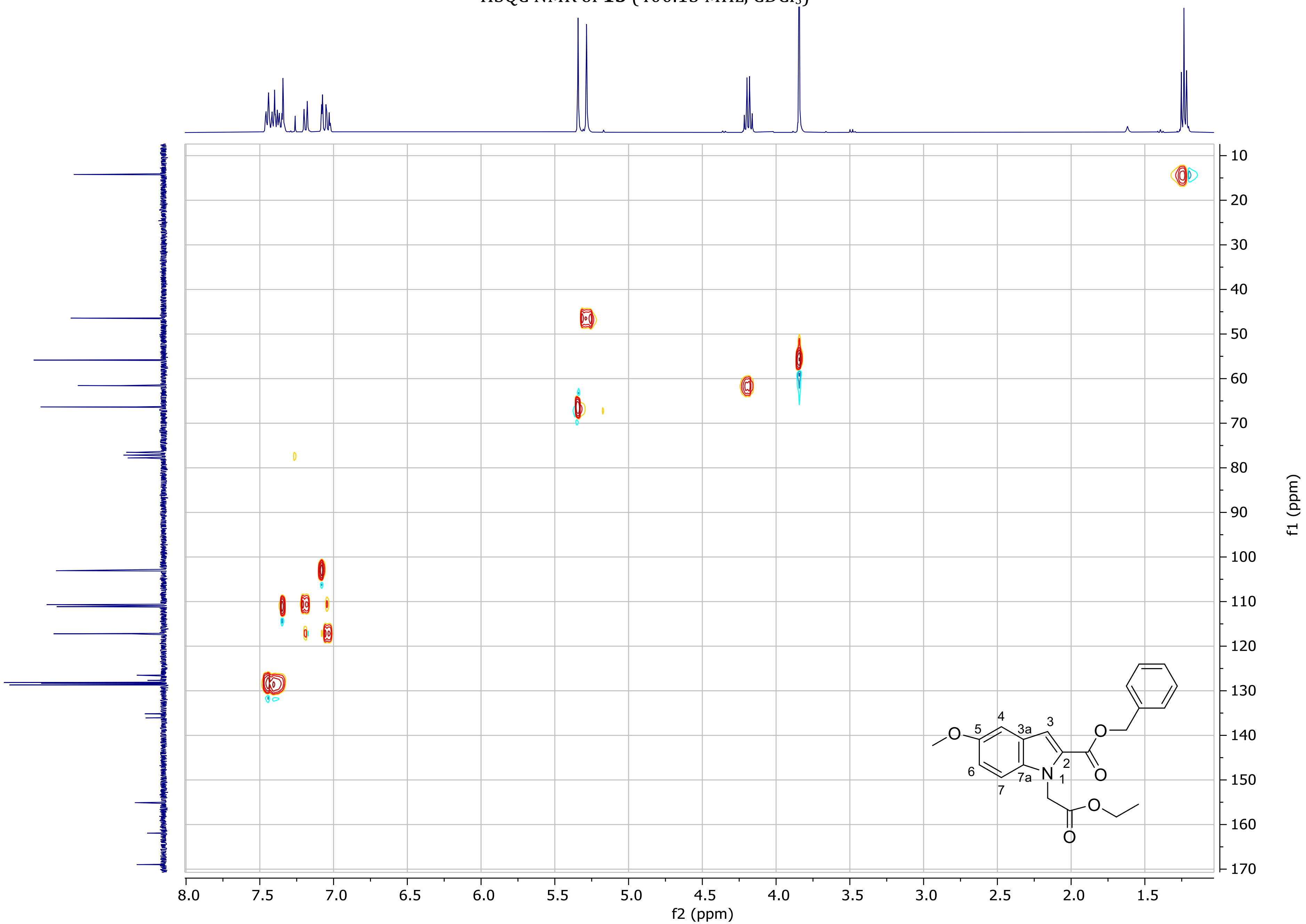

HMBC NMR of **15** (400.13 MHz, $CDCl_3$)

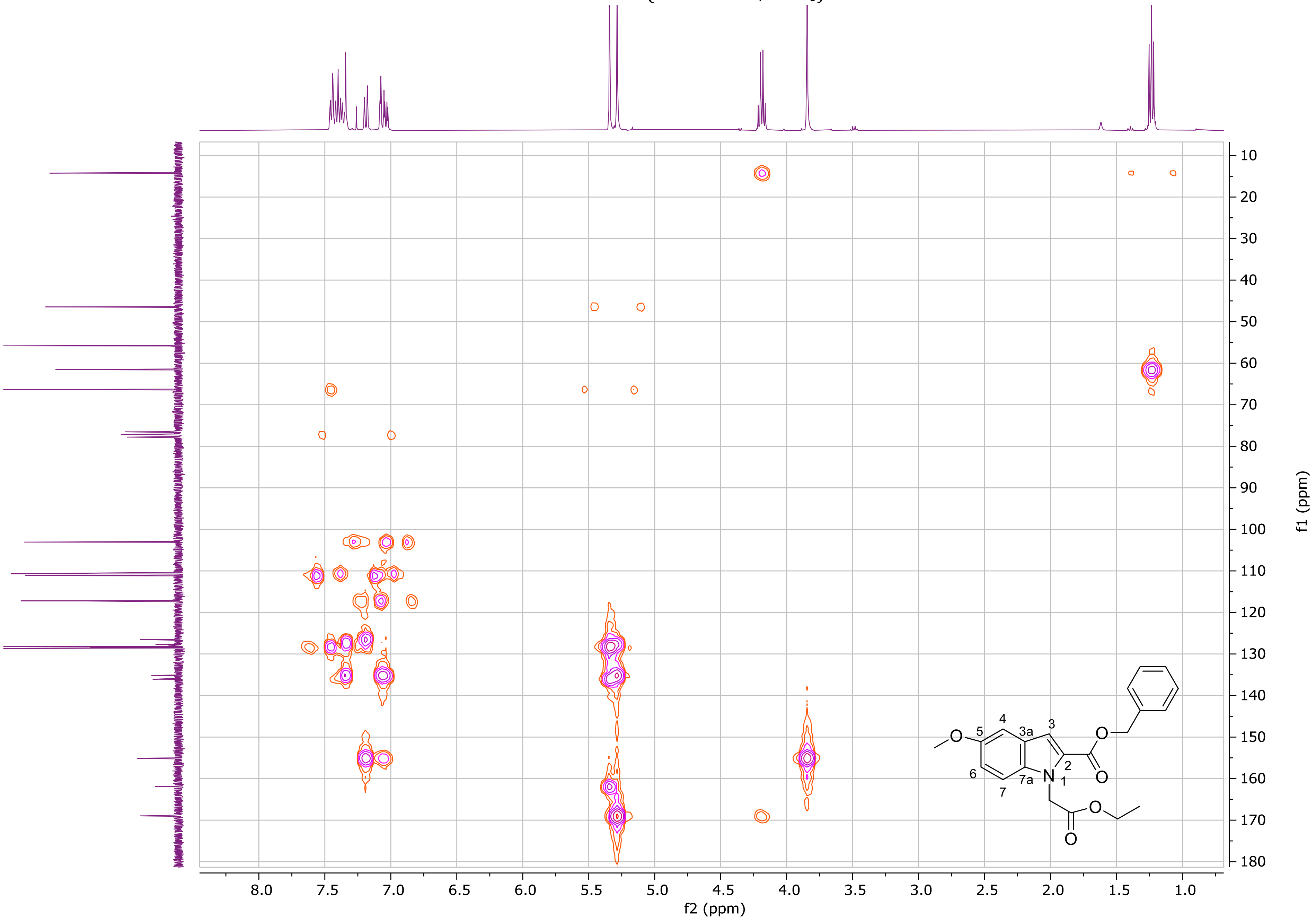

^{1}H NMR of **21** (400.13 MHz, $CDCl_3$)

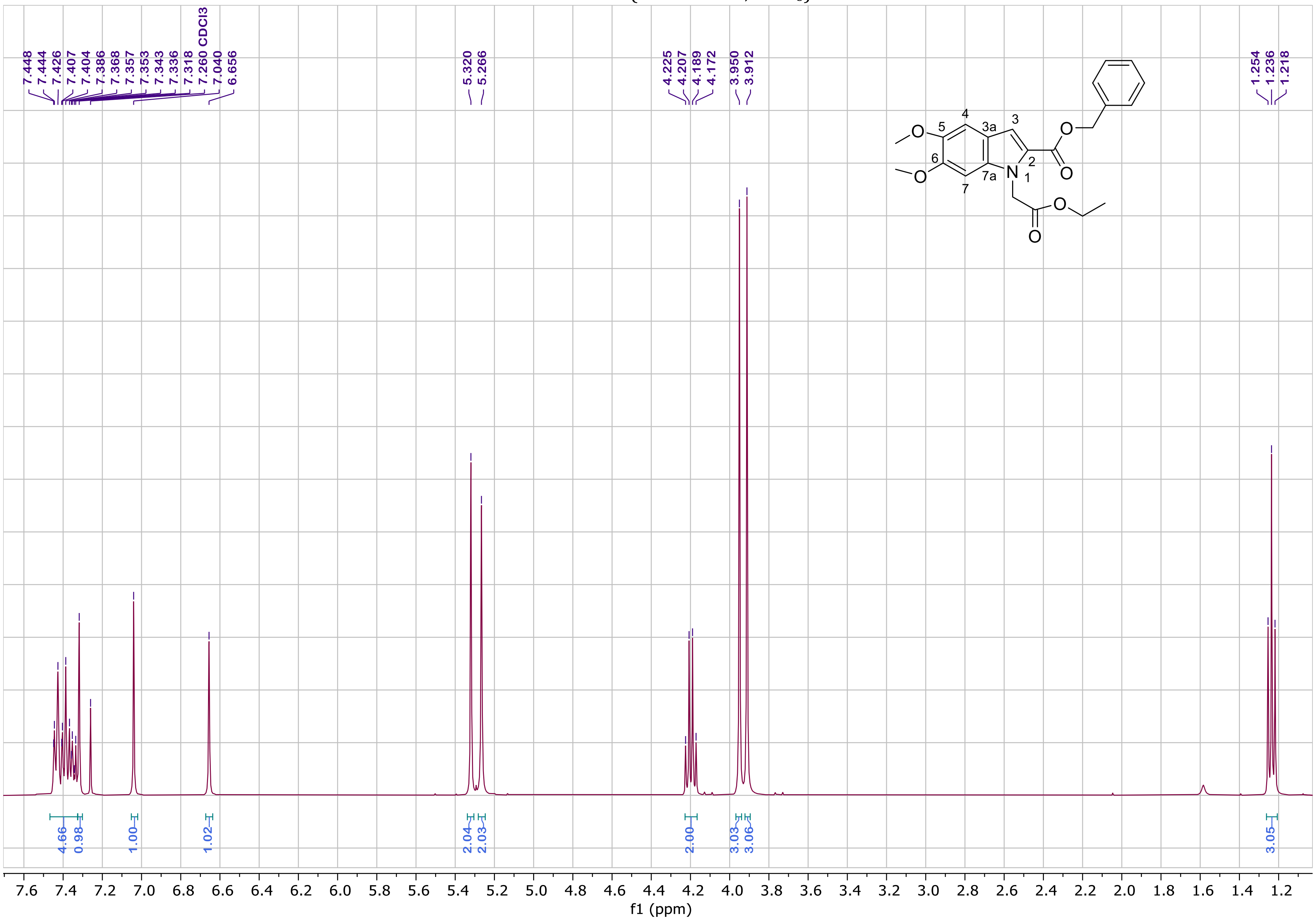

^{13}C NMR of **21** (50.32 MHz, $CDCl_3$)

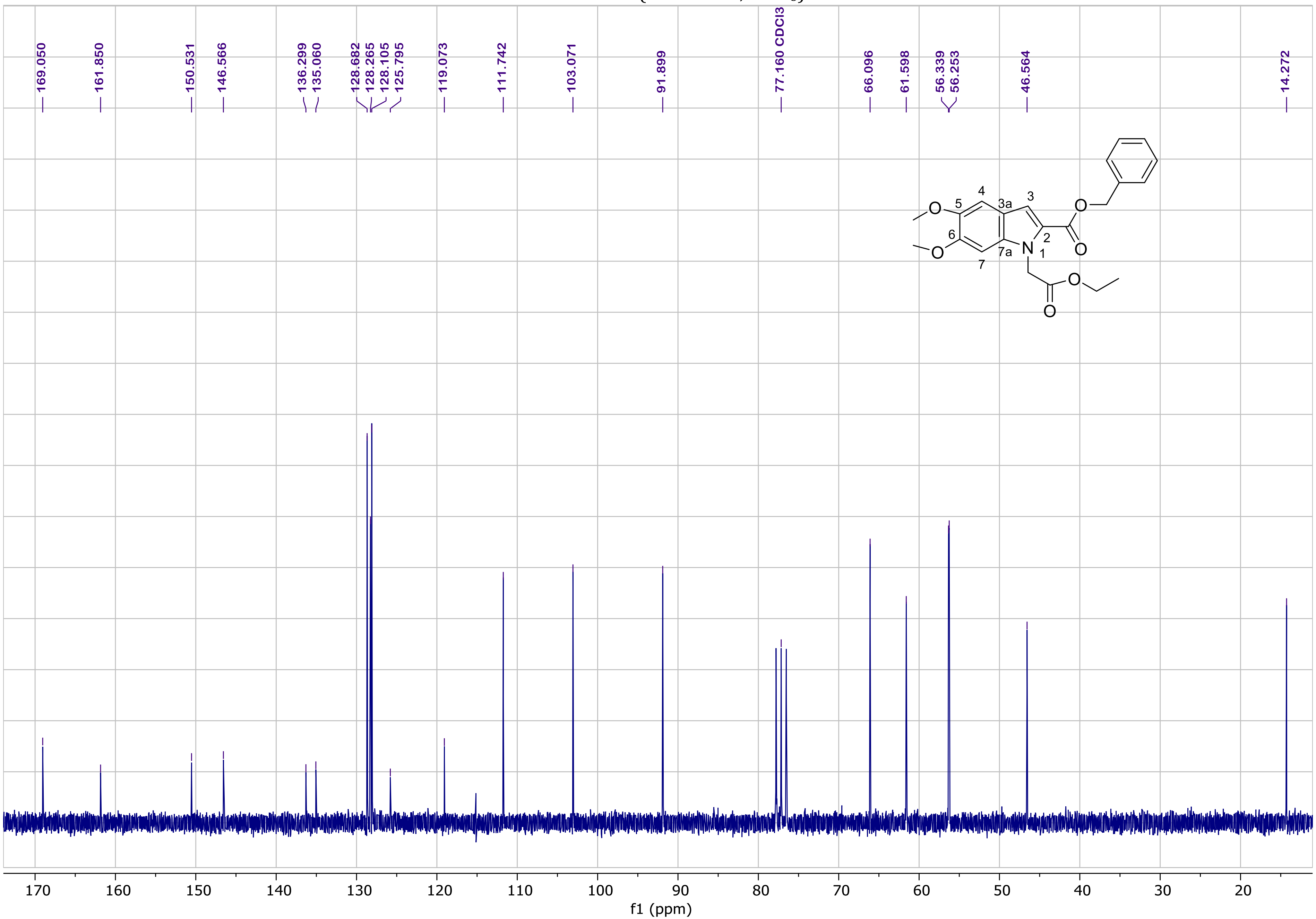

DEPT NMR of **21** (50.32 MHz, $CDCl_3$)

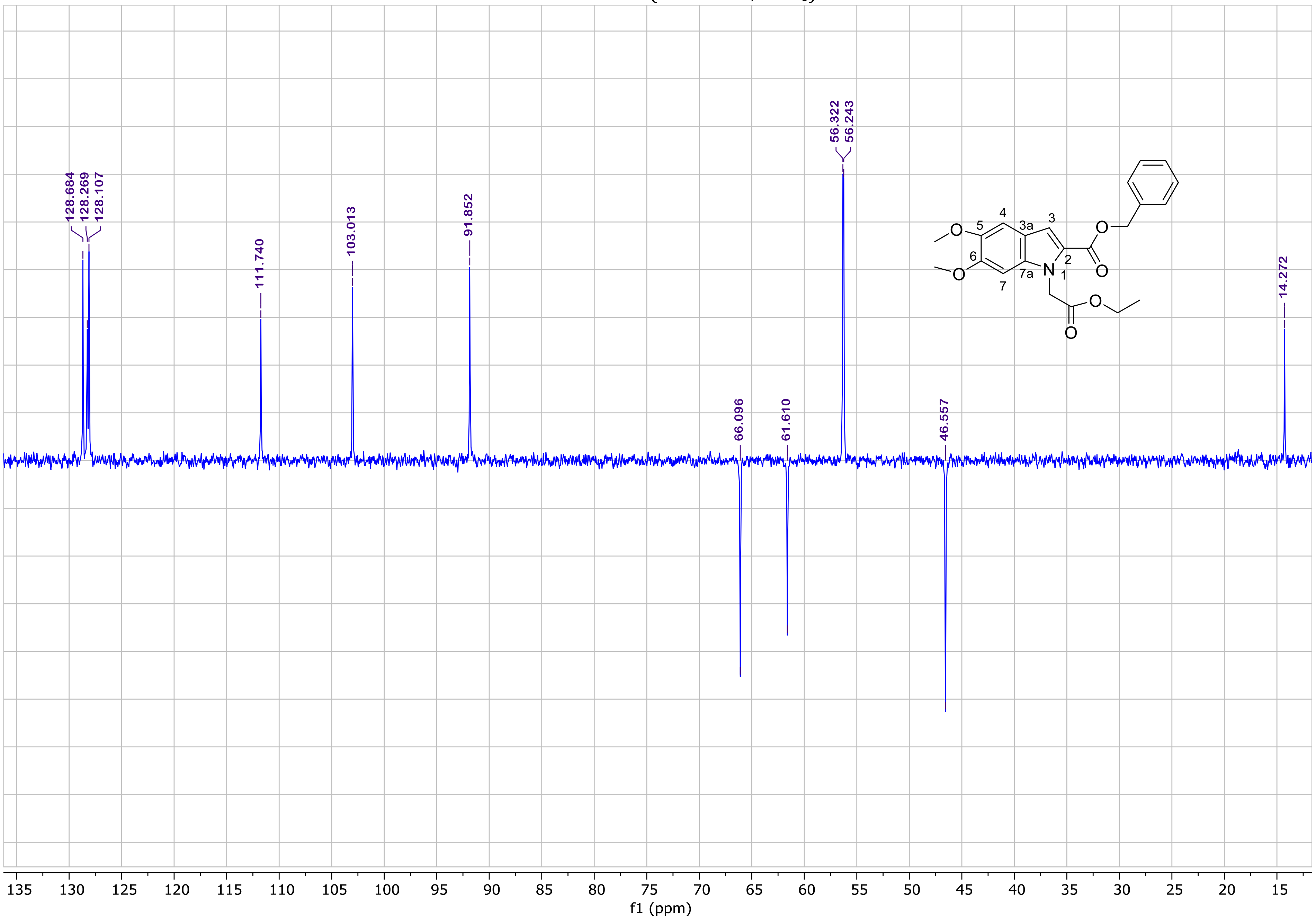

COSY NMR of **21** (400.13 MHz, $CDCl_3$)

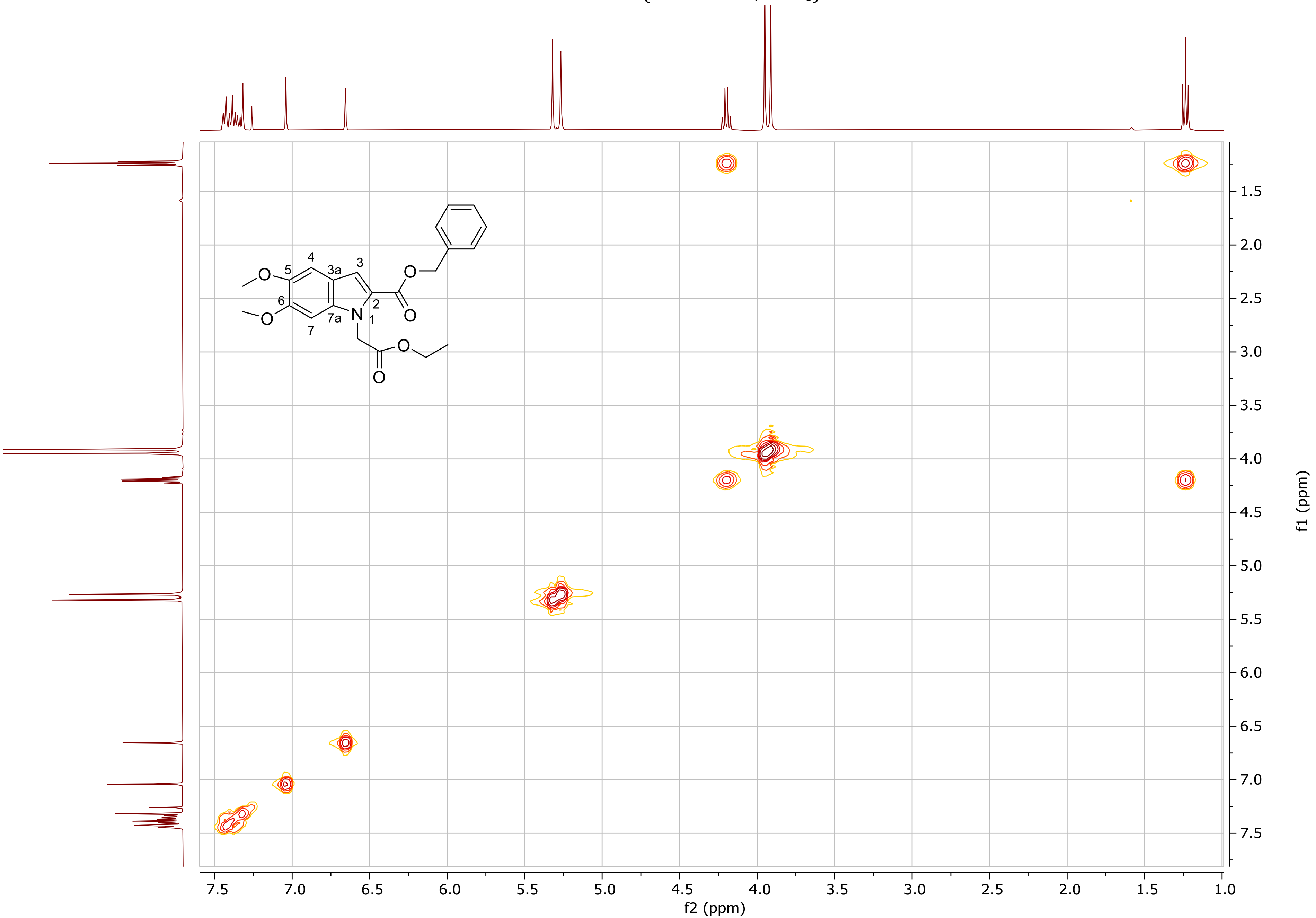

HSQC NMR of **21** (400.13 MHz, $CDCl_3$)

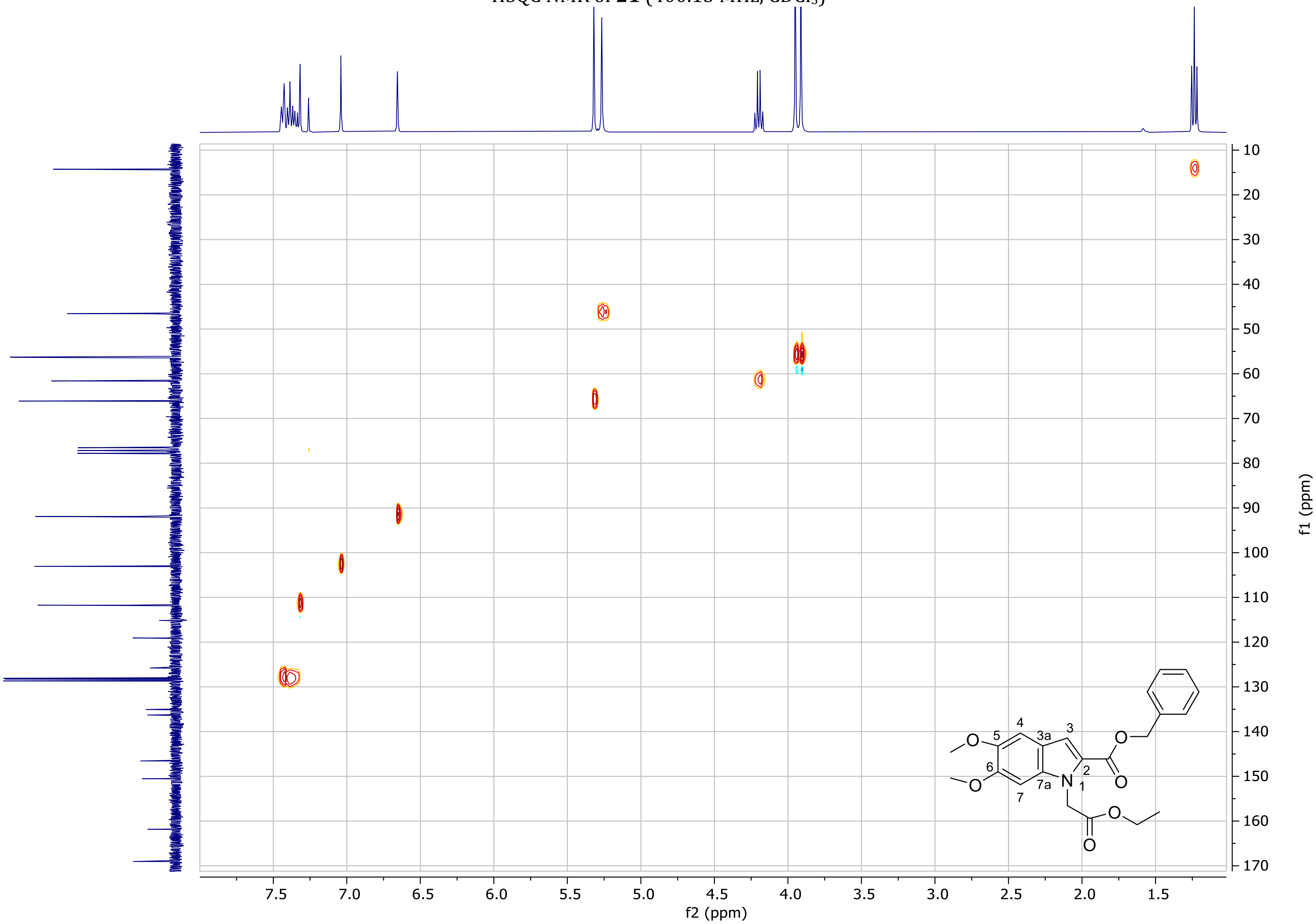

HMBC NMR of **21** (400.13 MHz, $CDCl_3$)

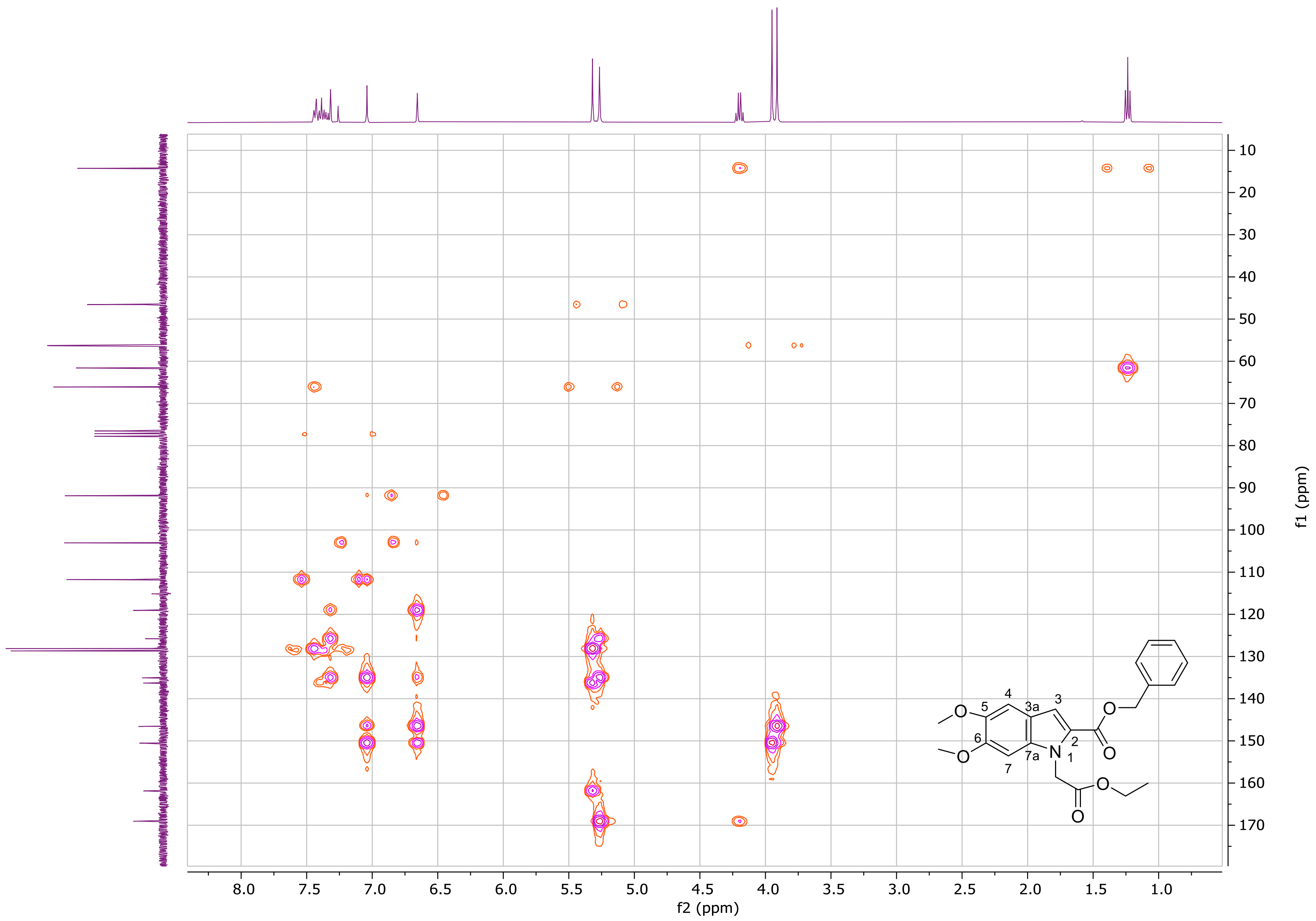

¹H NMR of **27** (400.13 MHz, $CDCl_3$)

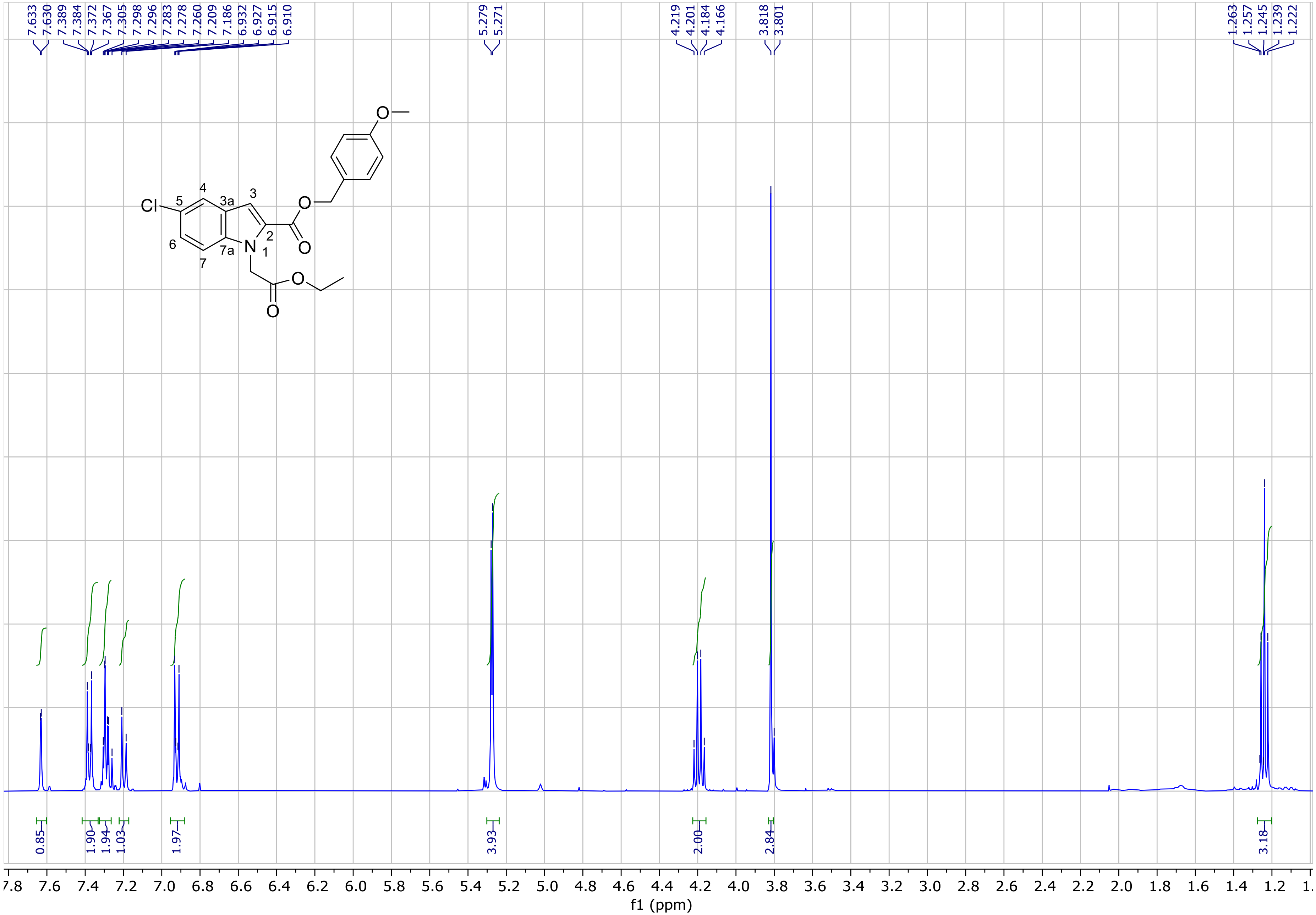

^{13}C NMR of **27** (50.32 MHz, $CDCl_3$)

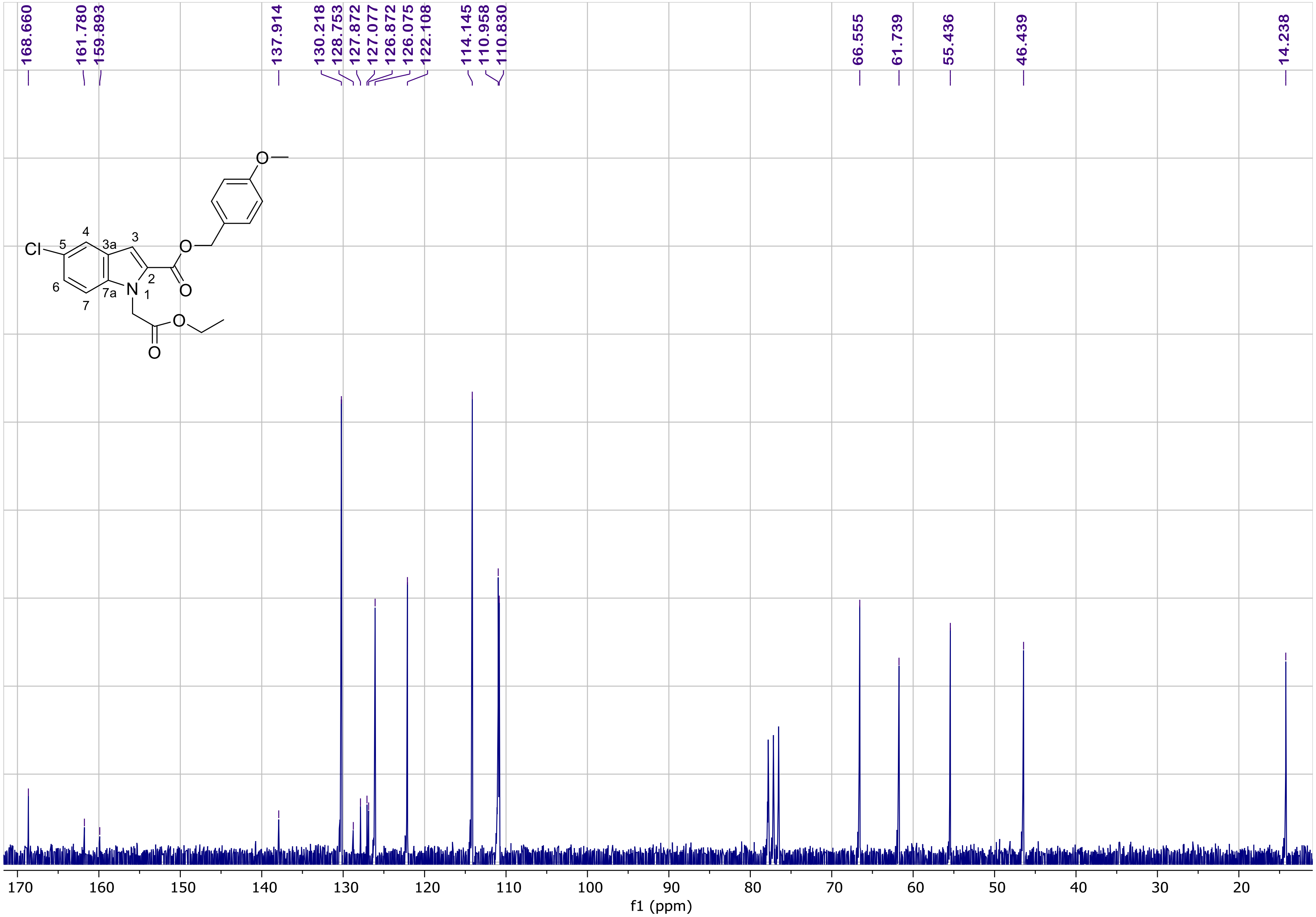

COSY NMR of **27** (400.13 MHz, $CDCl_3$)

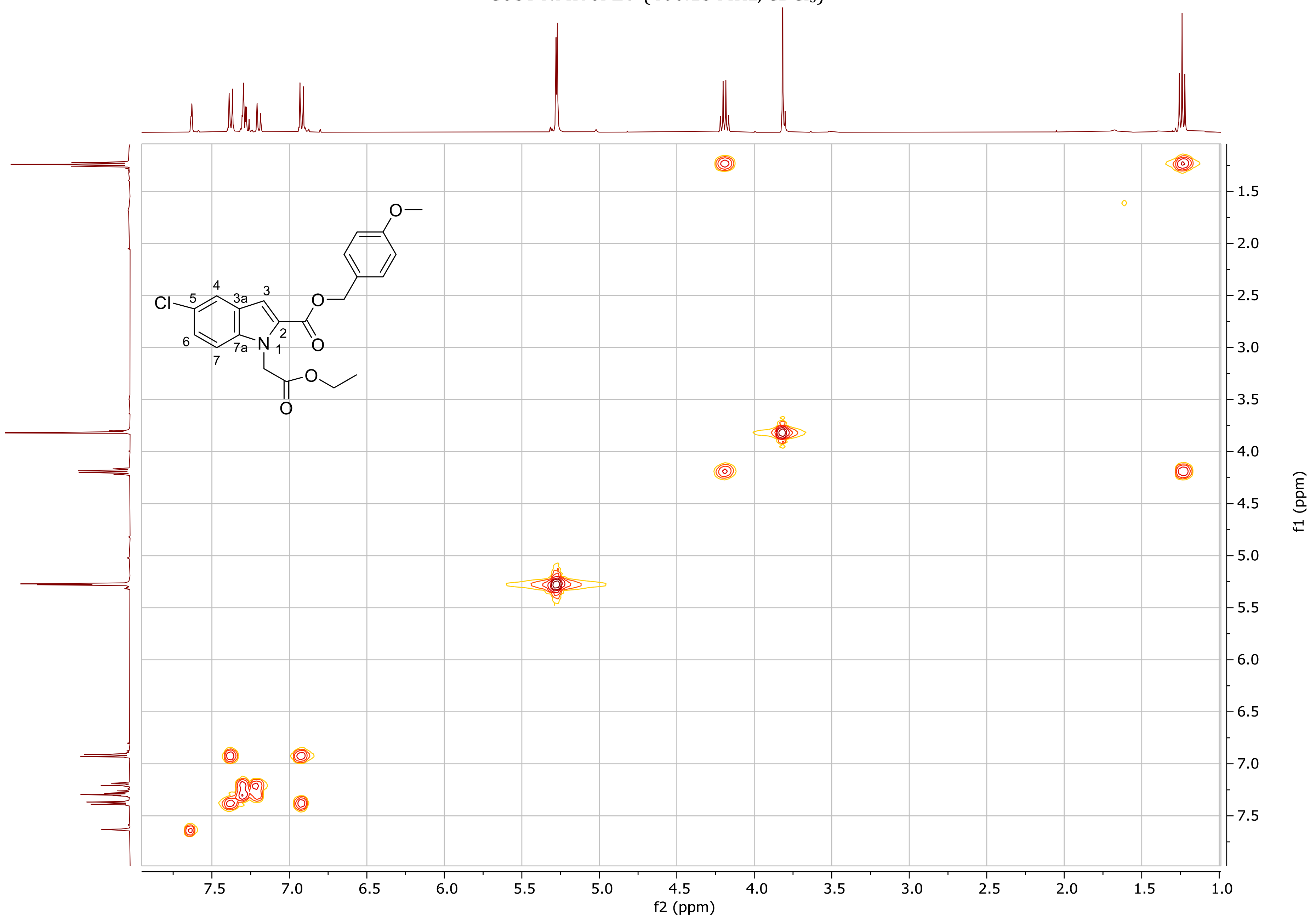

HSQC NMR of **27** (400.13 MHz, $CDCl_3$)

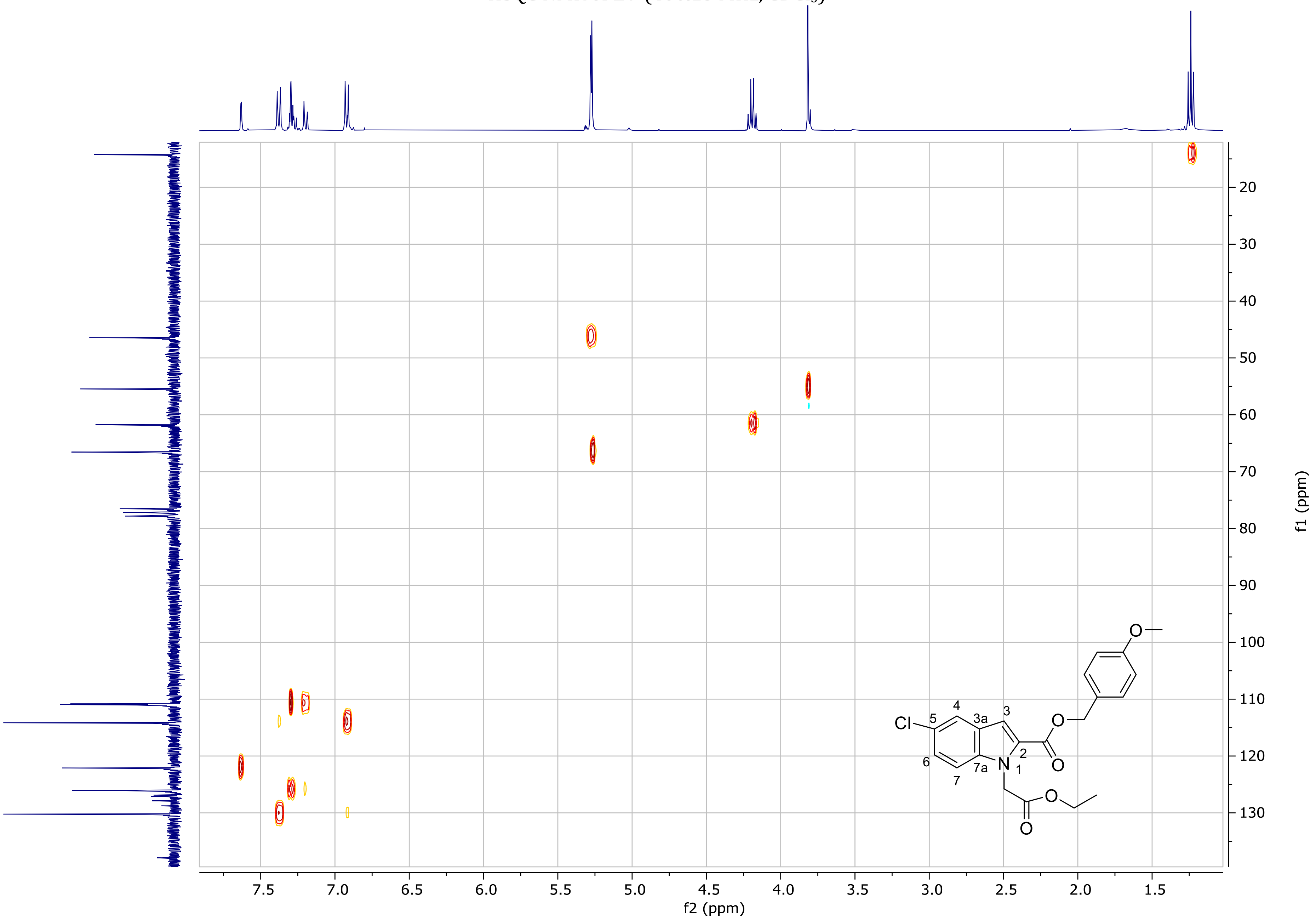

HMBC NMR of **27** (400.13 MHz, $CDCl_3$)

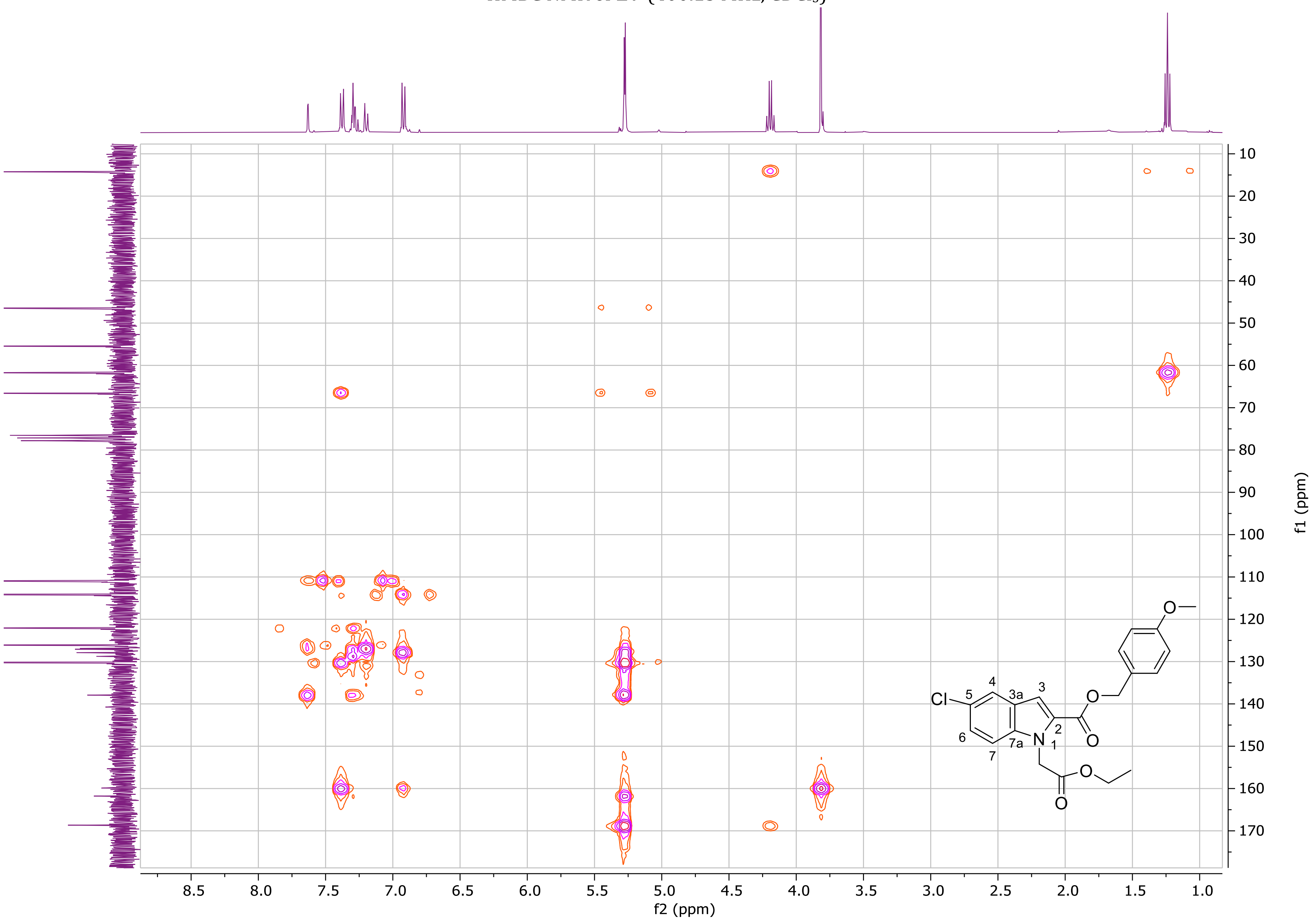

^1H NMR of **33** (400.13 MHz, $CDCl_3$)

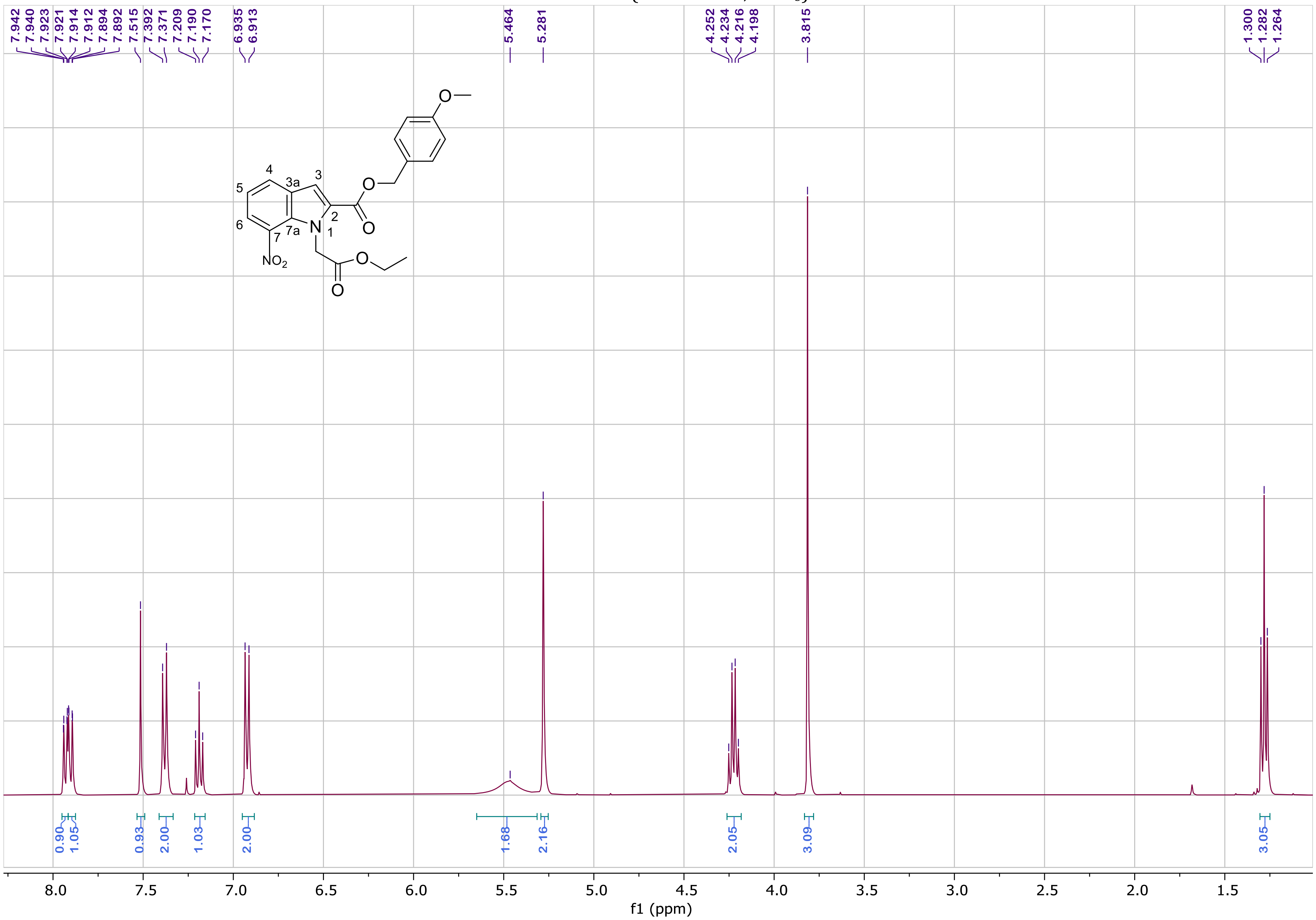

^{13}C NMR of **33** (50.32 MHz, $CDCl_3$)

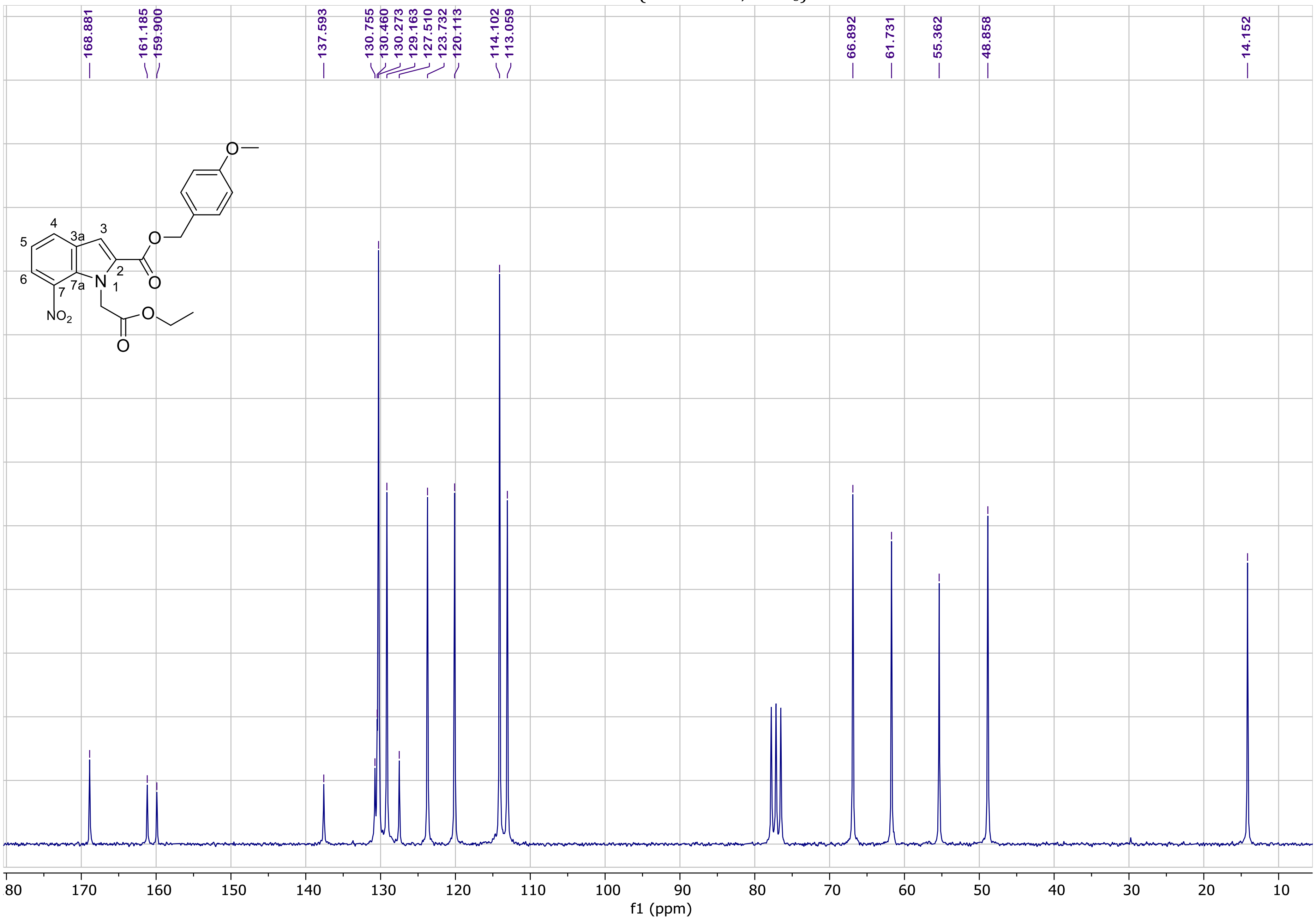

DEPT NMR of **33** (50.32 MHz, $CDCl_3$)

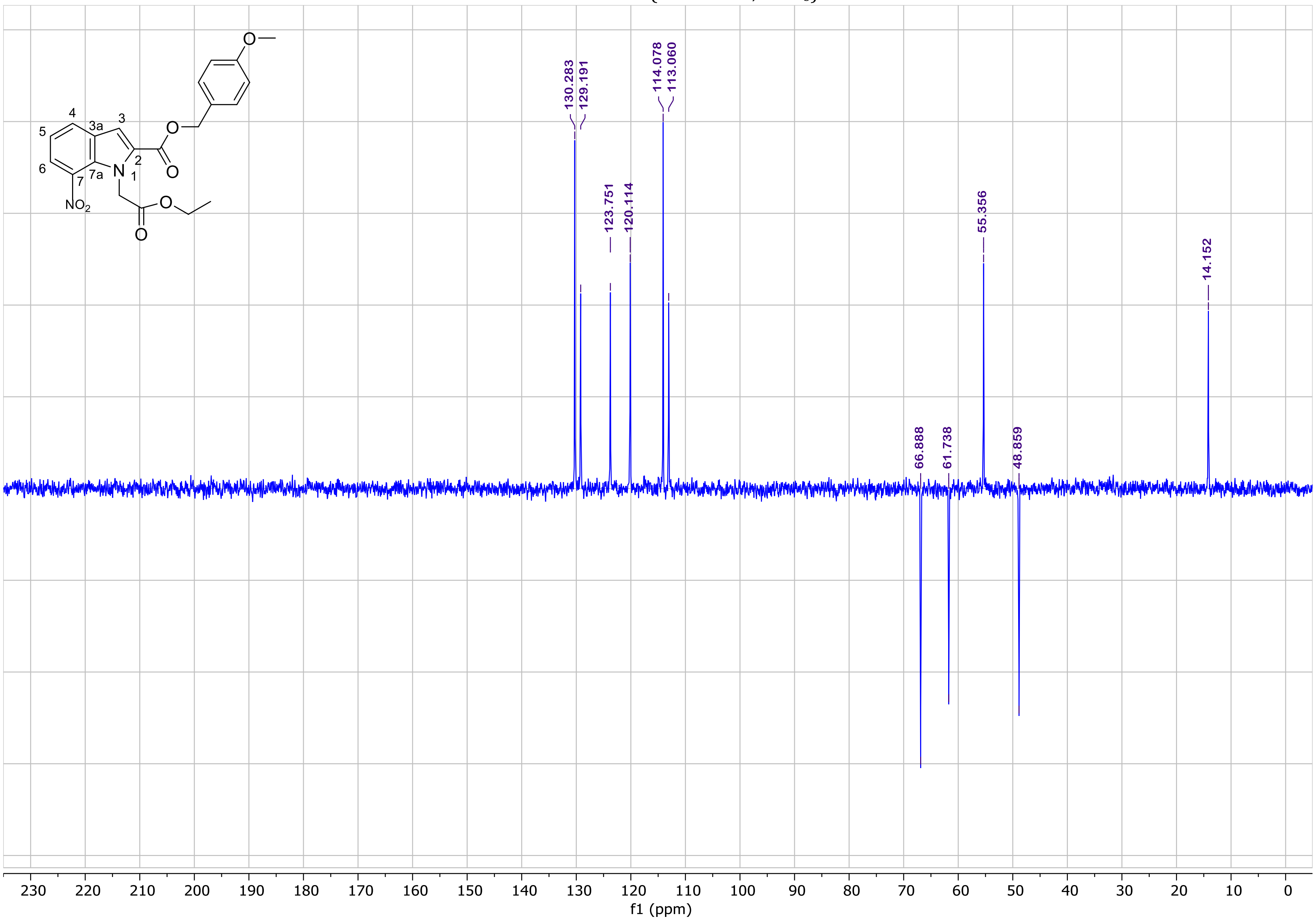

COSY NMR of **33** (400.13 MHz, $CDCl_3$)

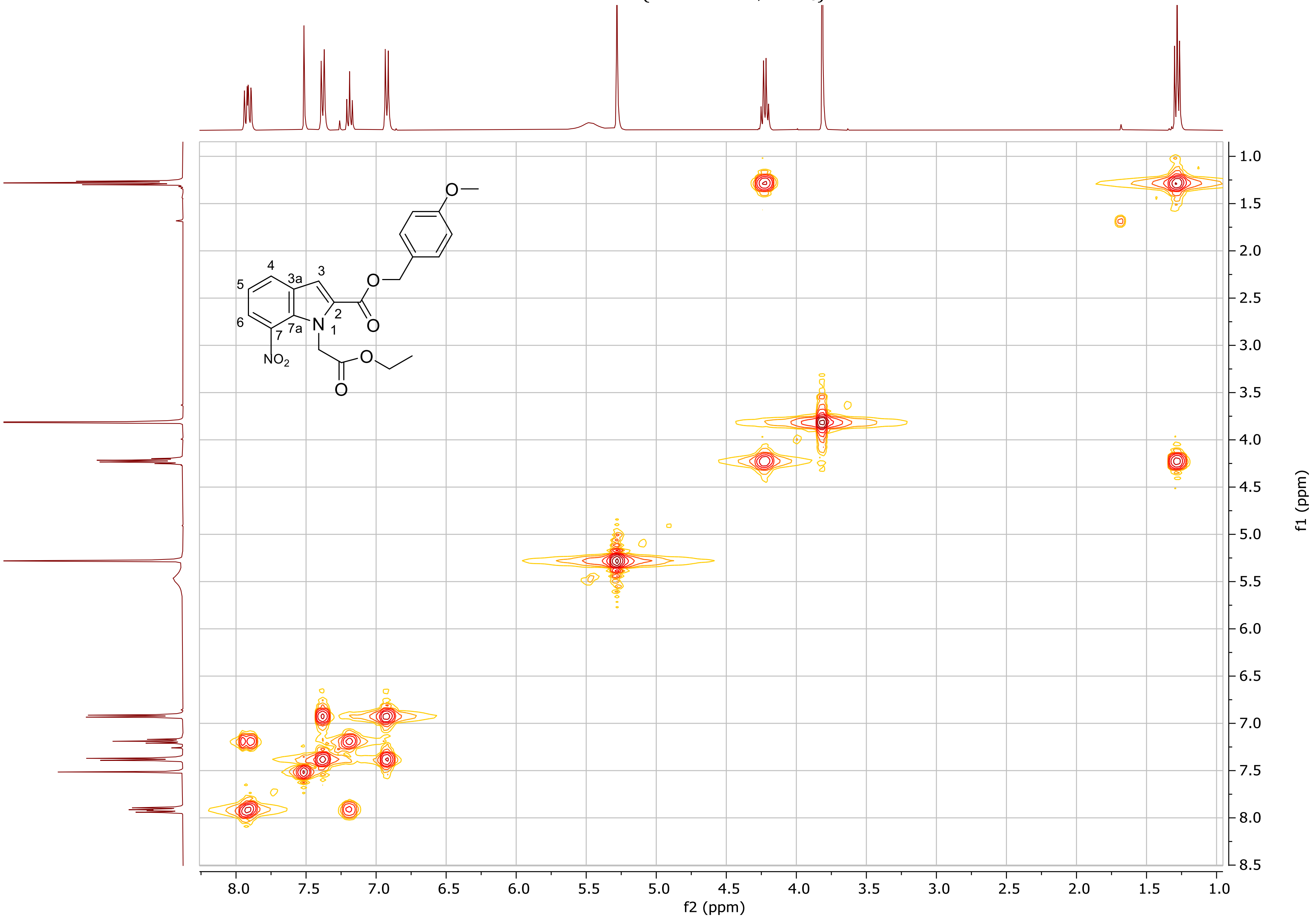

HSQC NMR of **33** (400.13 MHz, $CDCl_3$)

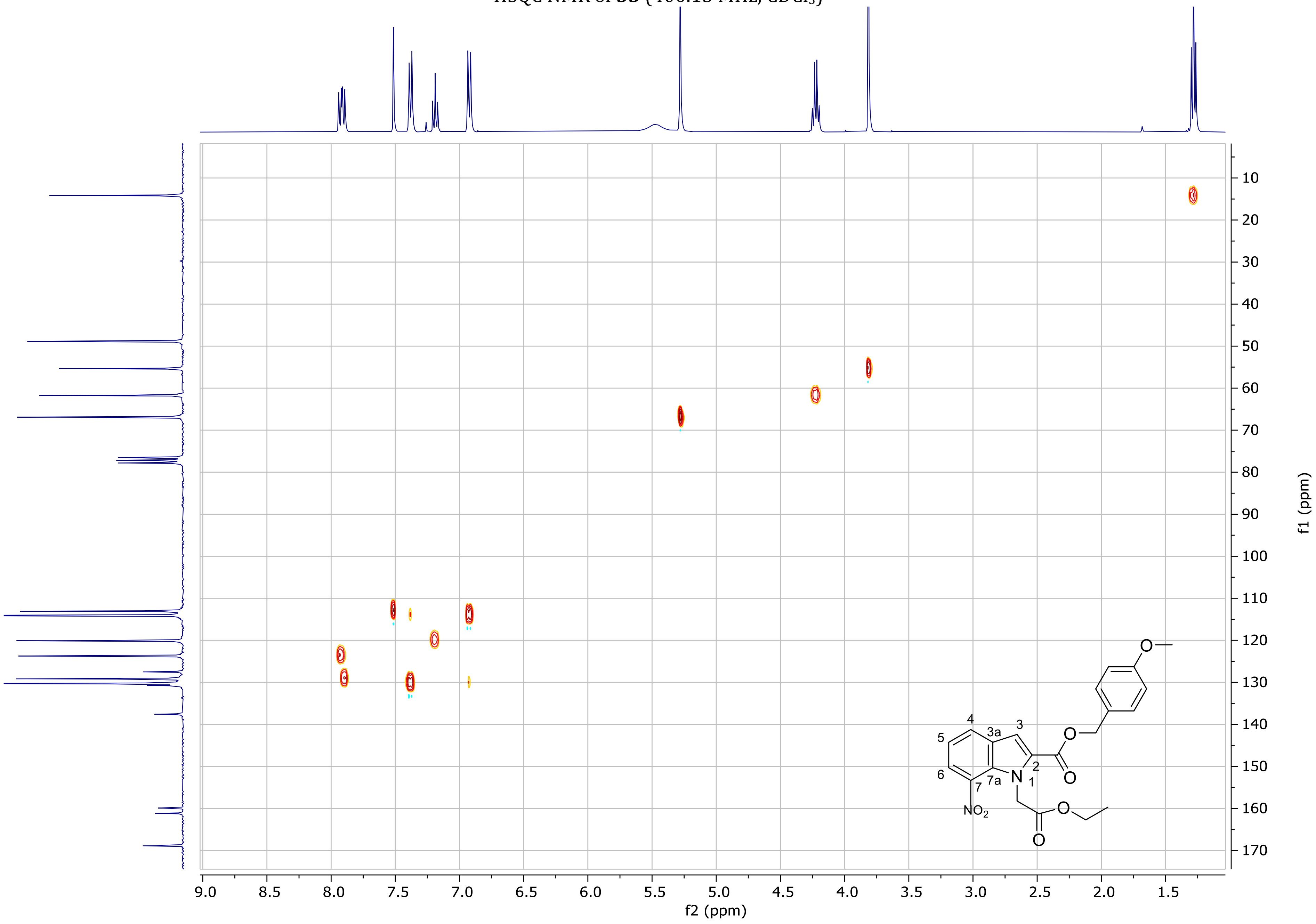

HMBC NMR of **33** (400.13 MHz, $CDCl_3$)

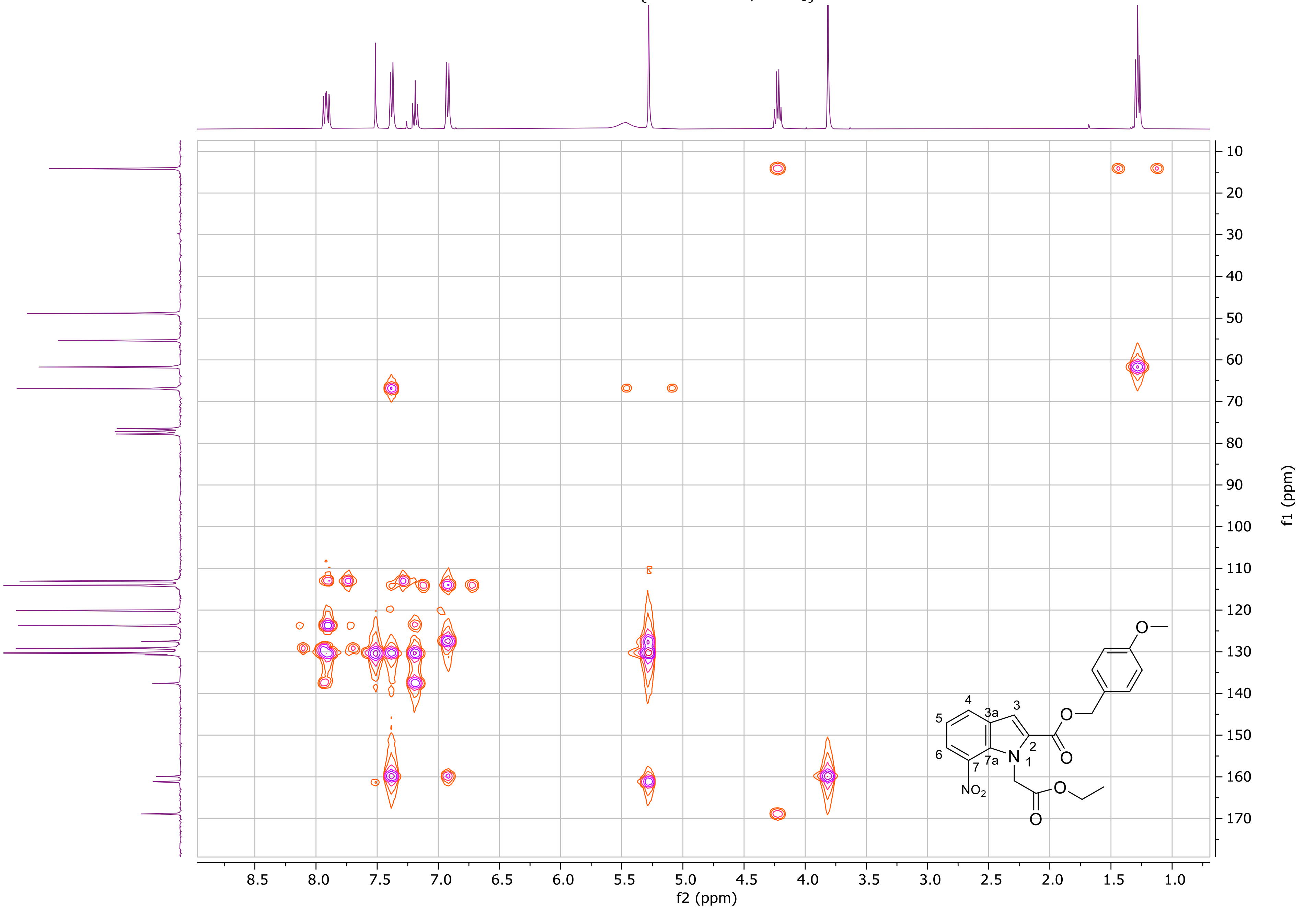

^{1}H NMR of **4** (400.13 MHz, $CDCl_3$)

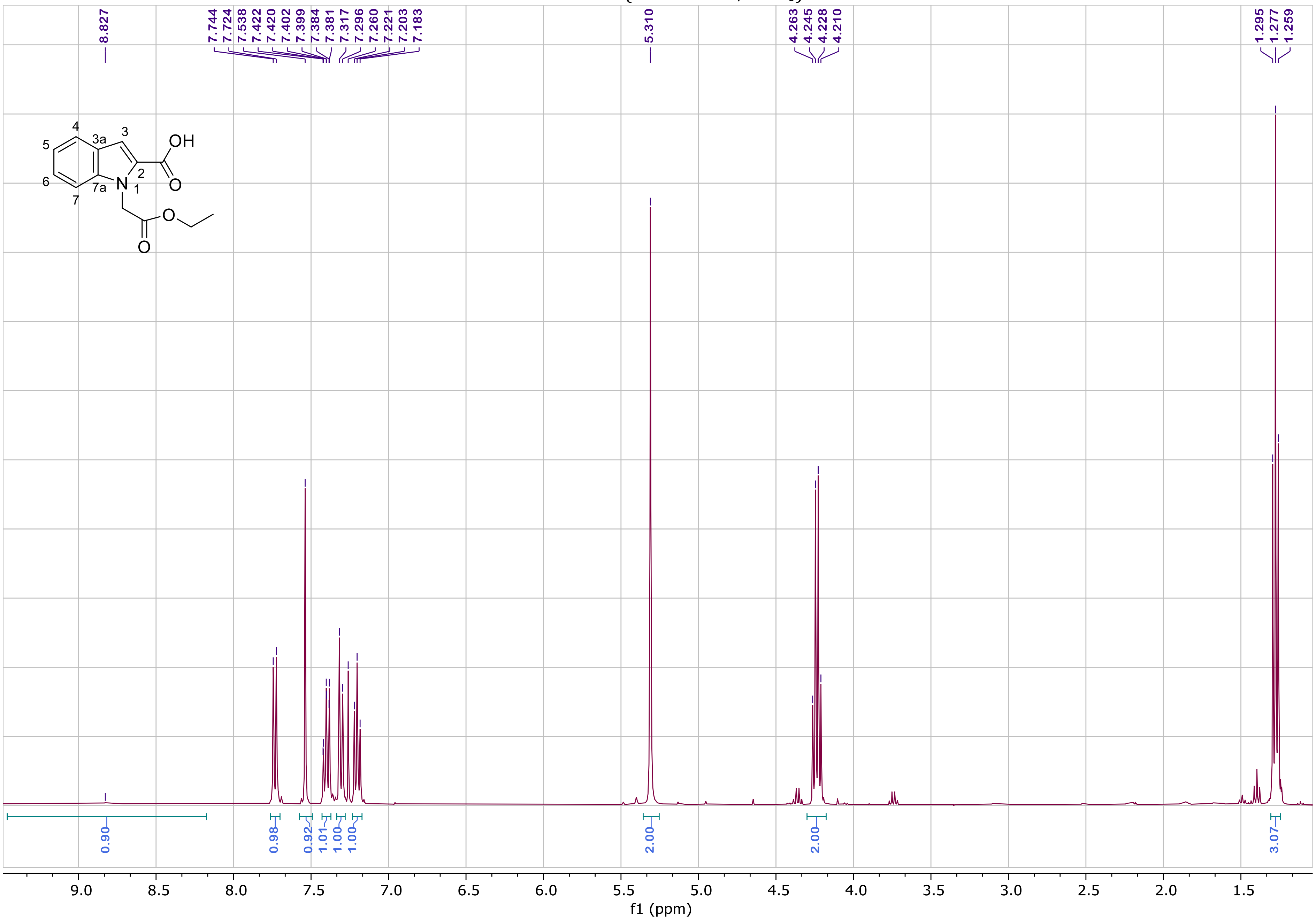

^{13}C NMR of **4** (50.32 MHz, CDCl$_3$)

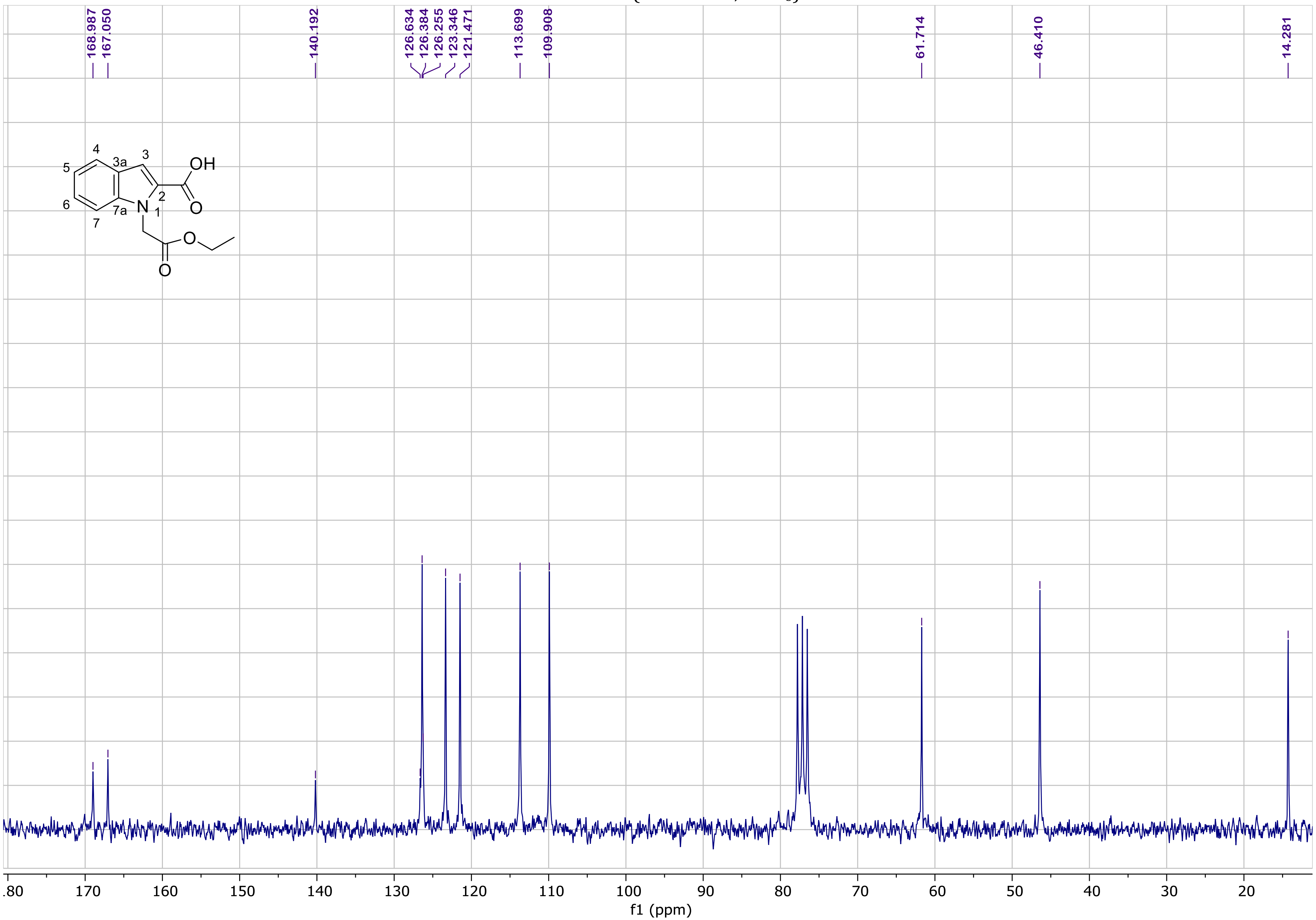

DEPT NMR of **4** (50.32 MHz, $CDCl_3$)

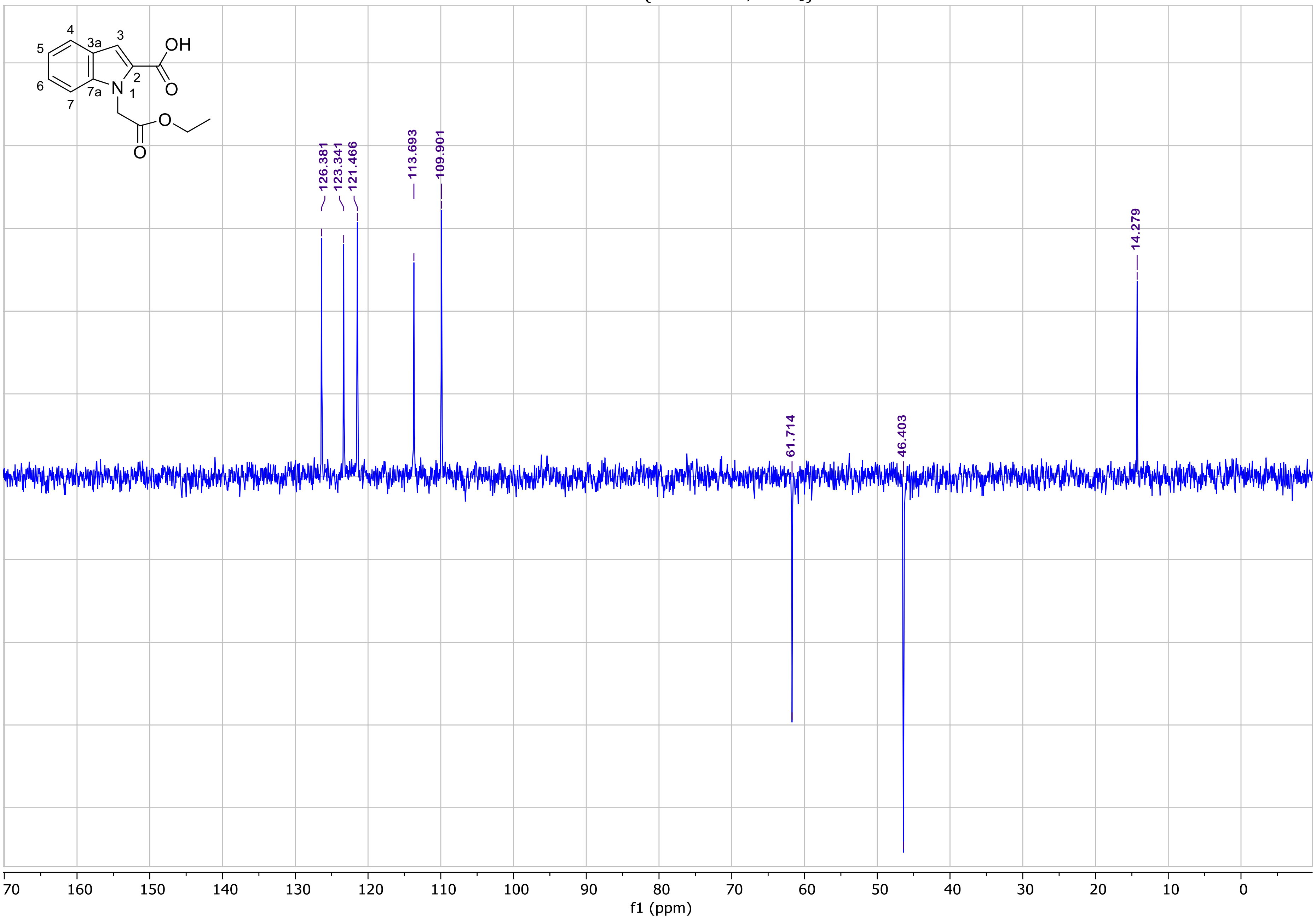

COSY NMR of **4** (400.13 MHz, $CDCl_3$)

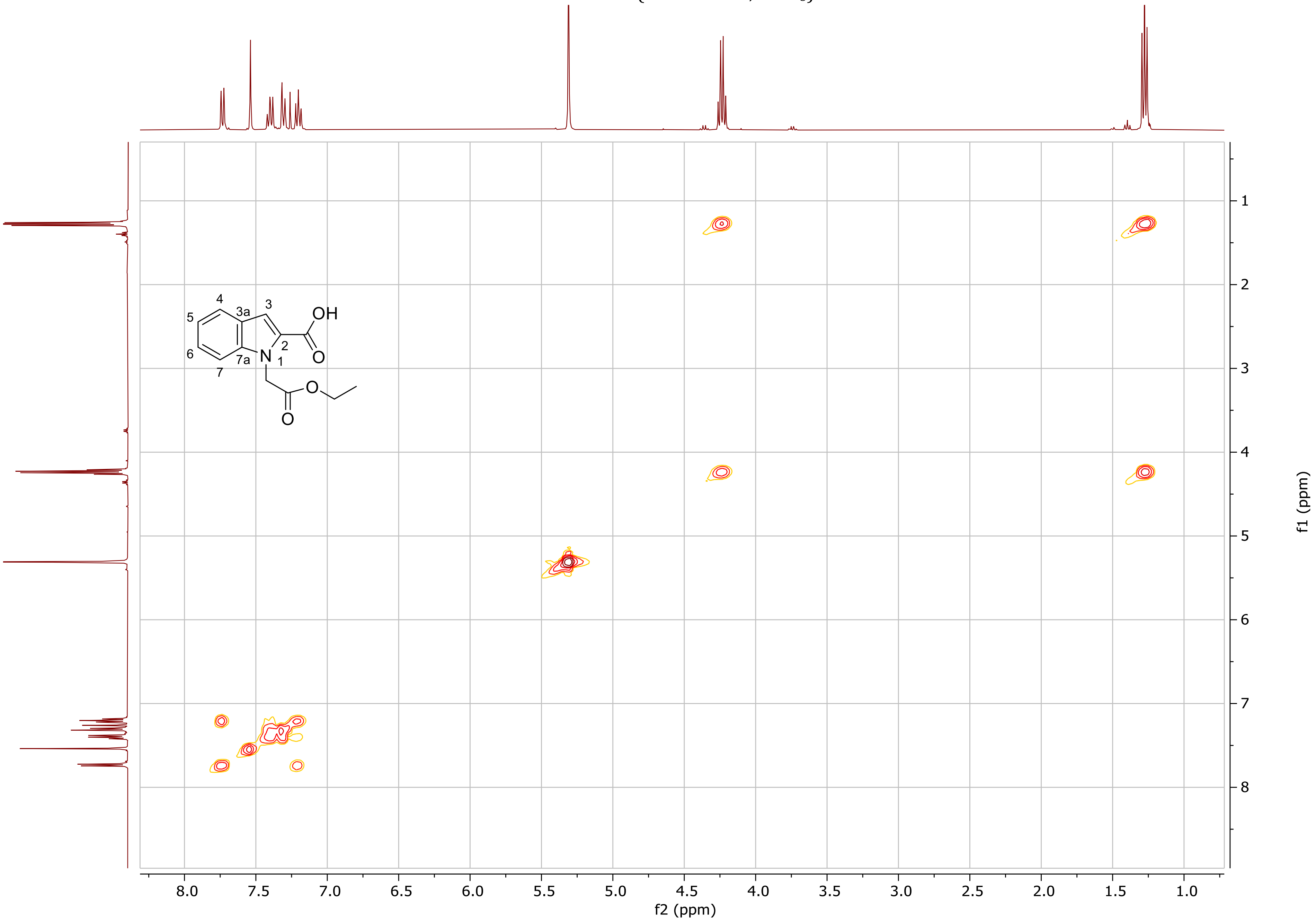

HSQC NMR of **4** (400.13 MHz, $CDCl_3$)

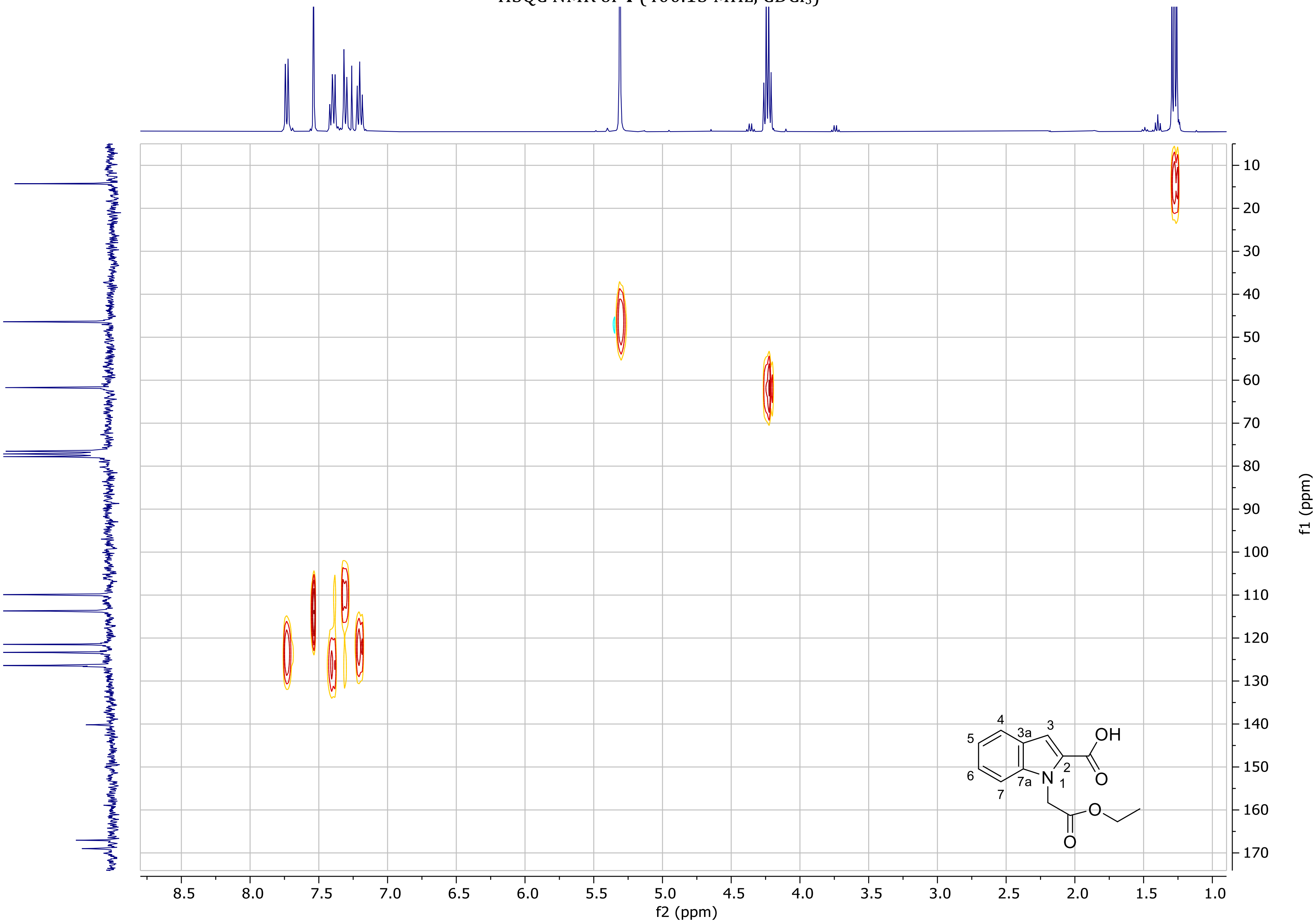

HMBC NMR of **4** (400.13 MHz, $CDCl_3$)

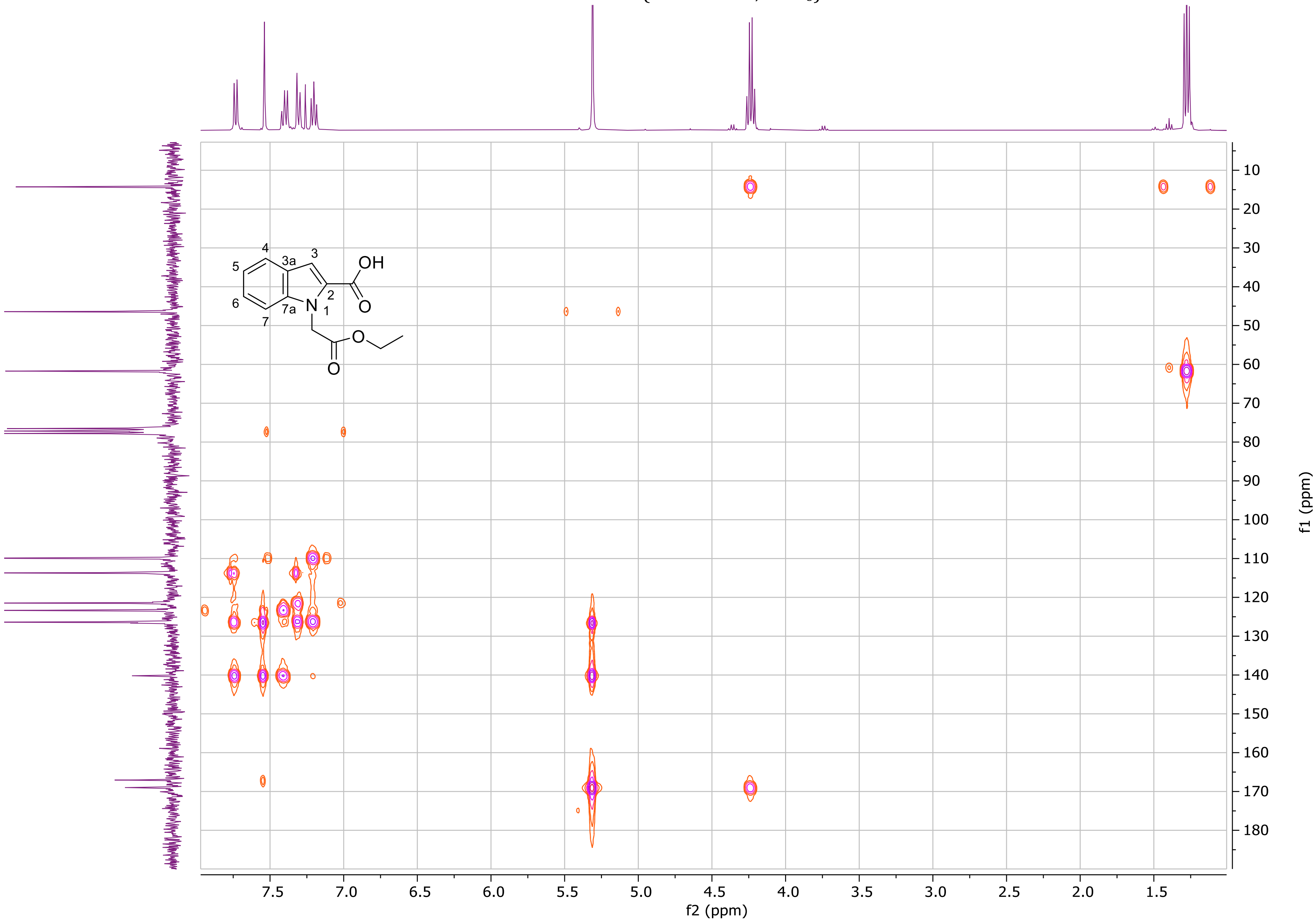

^{1}H NMR of **10** (400.13 MHz, DMSO-d_6)

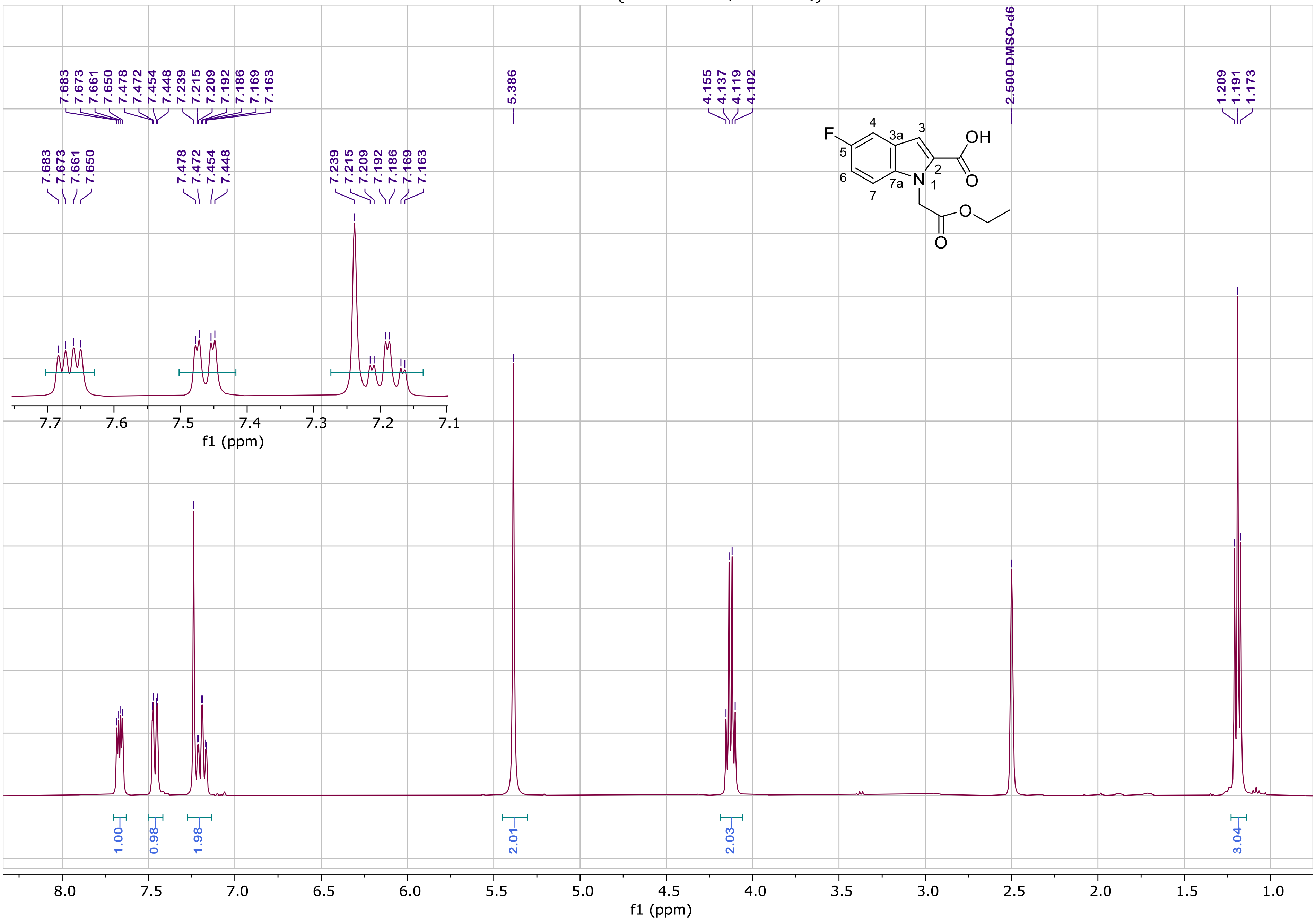

^{13}C NMR of **10** (50.32 MHz, DMSO-d_6)

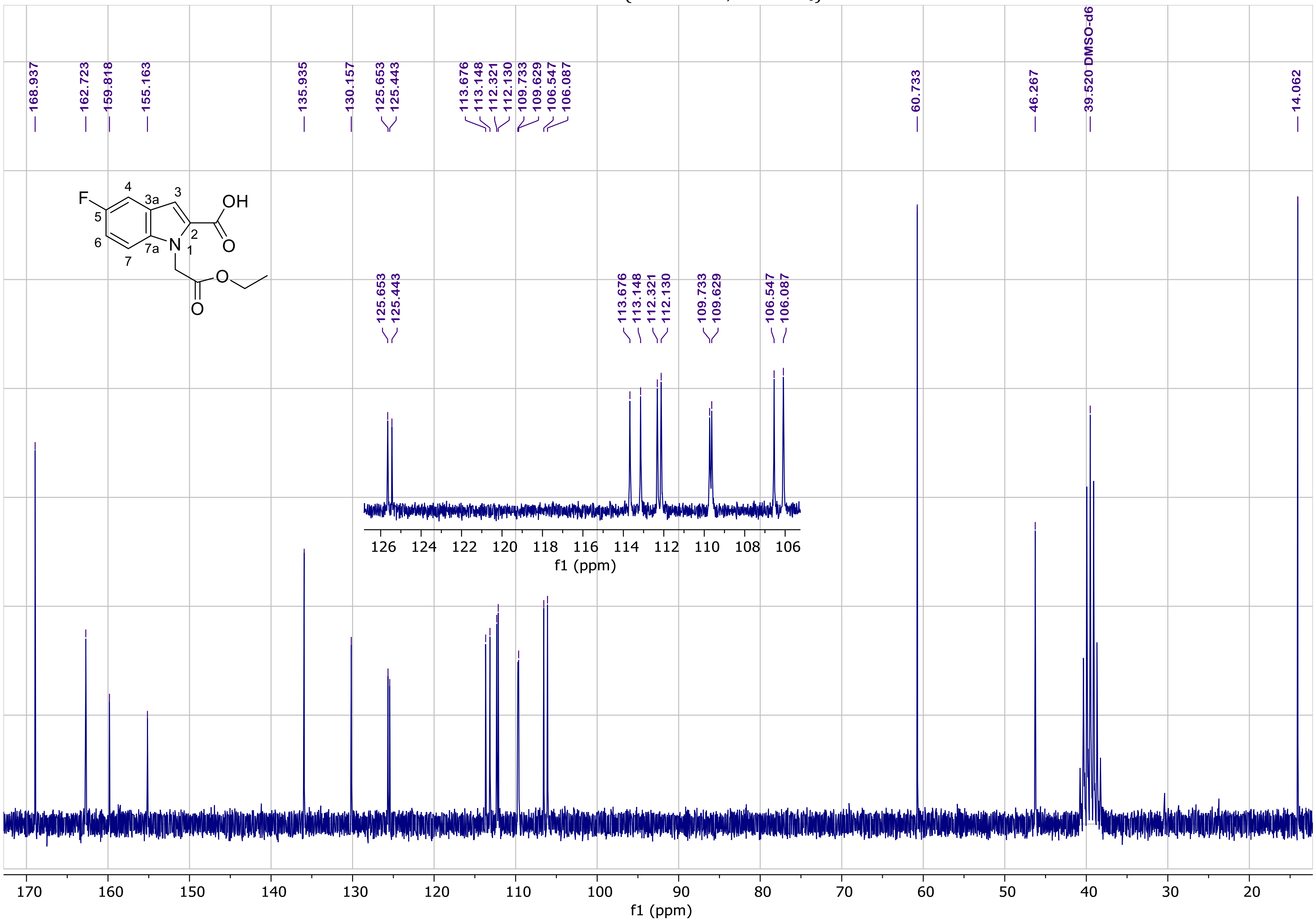

DEPT NMR of **10** (50.32 MHz, DMSO-d_6)

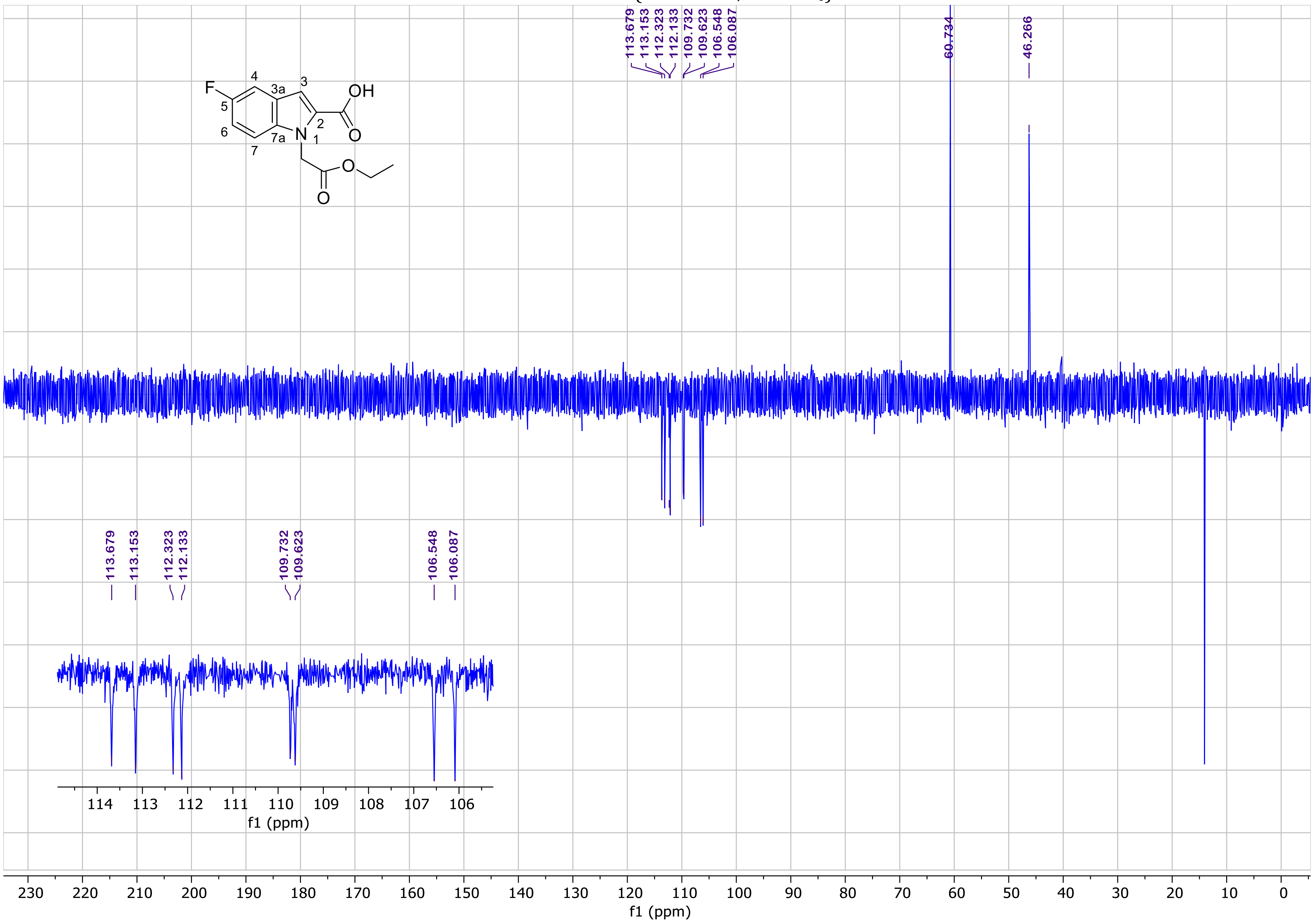

COSY NMR of **10** (400.13 MHz, DMSO-d_6)

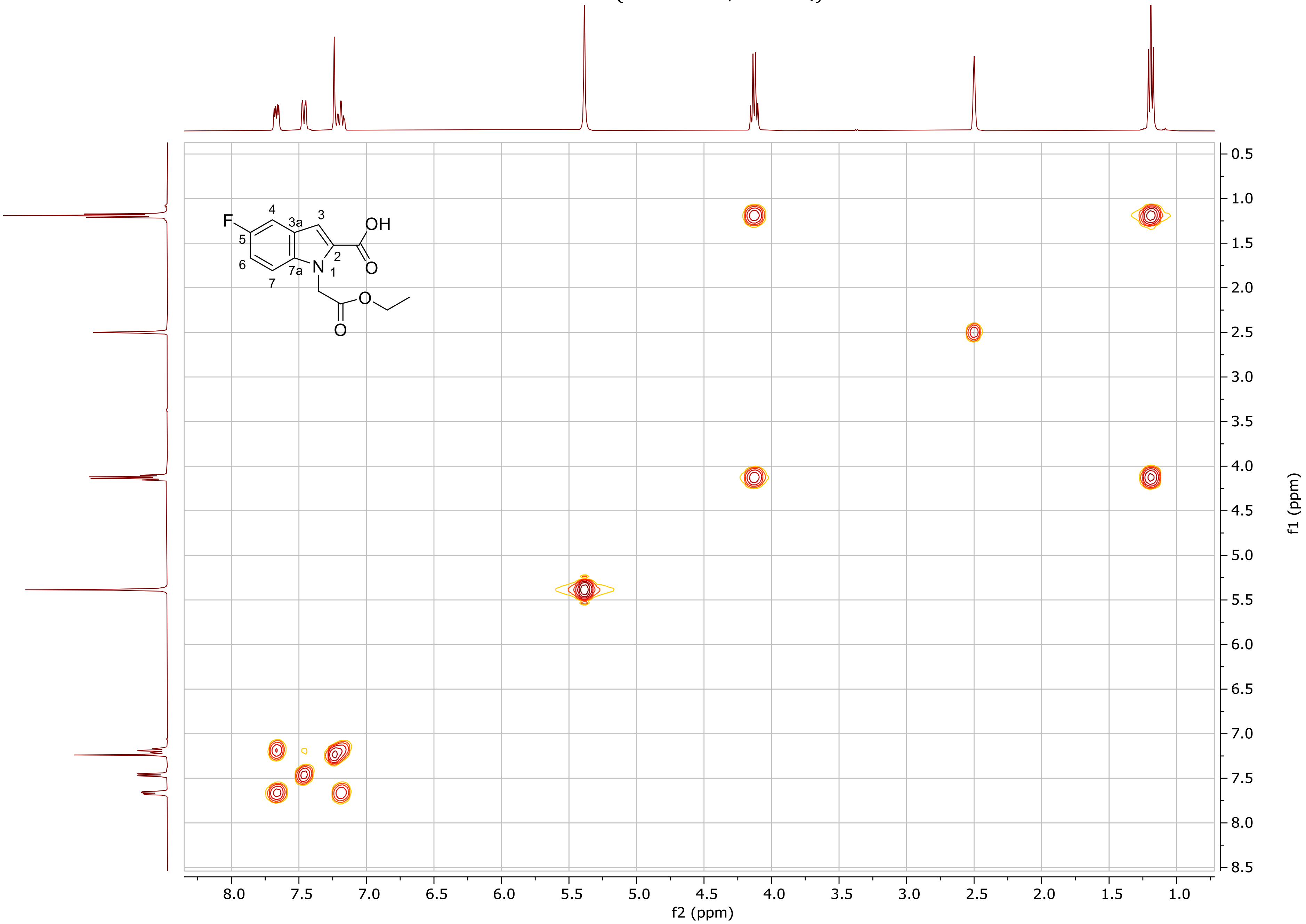

HSQC NMR of **10** (400.13 MHz, DMSO-d_6)

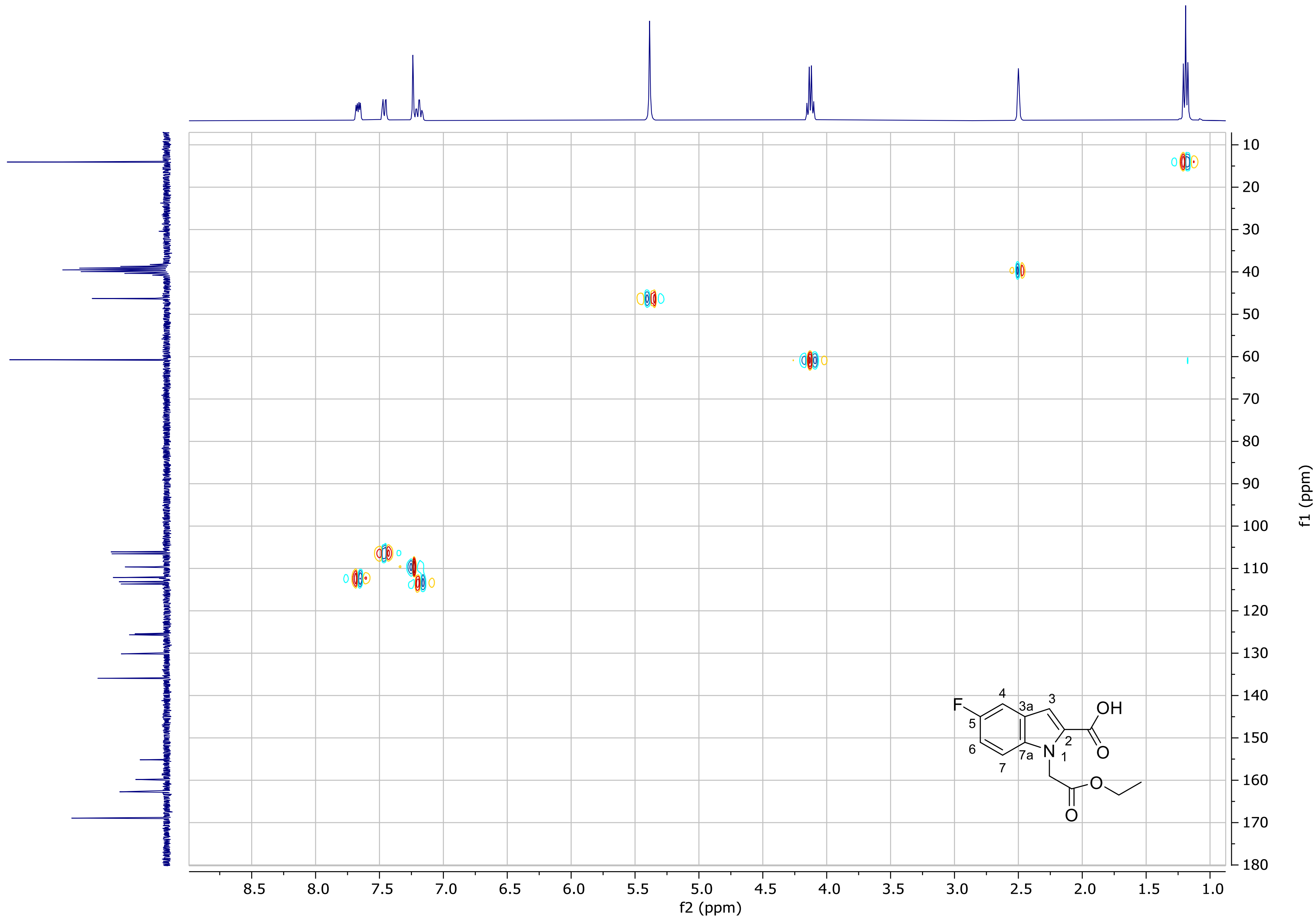

HMBC NMR of **10** (400.13 MHz, DMSO-d_6)

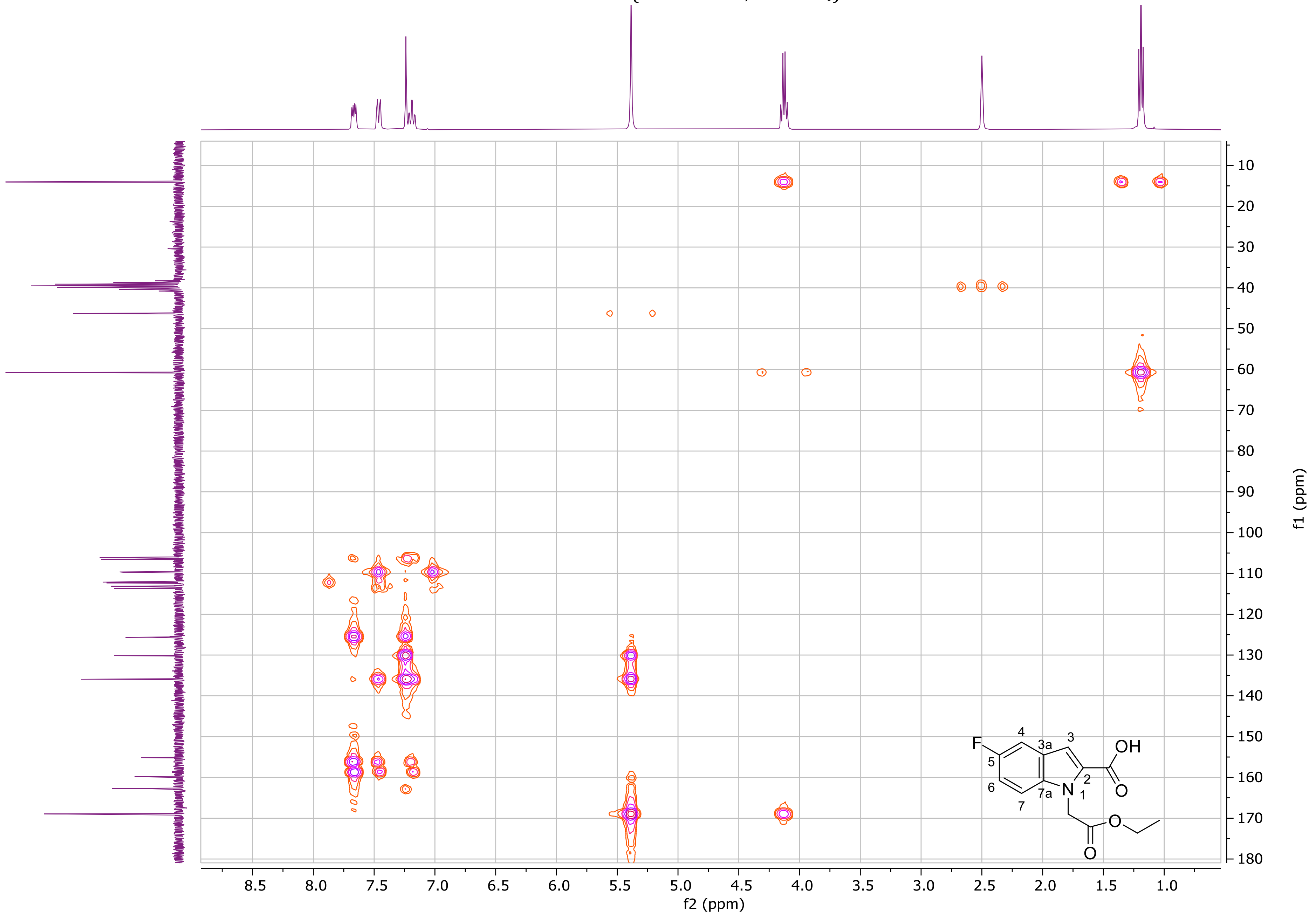

^{1}H NMR of **16** (400.13 MHz, DMSO-d_6)

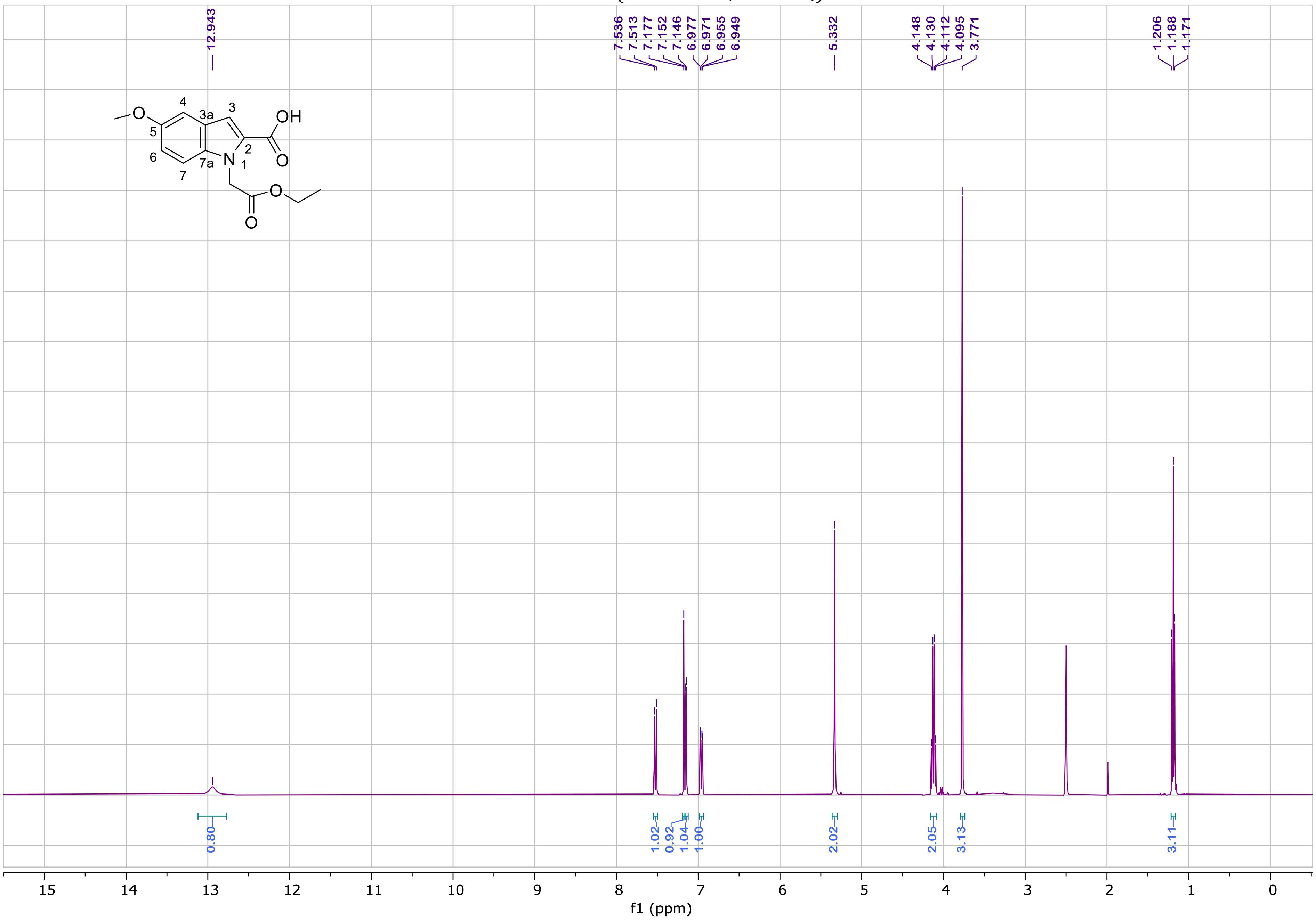

^{13}C NMR of **16** (50.32 MHz, DMSO-d_6)

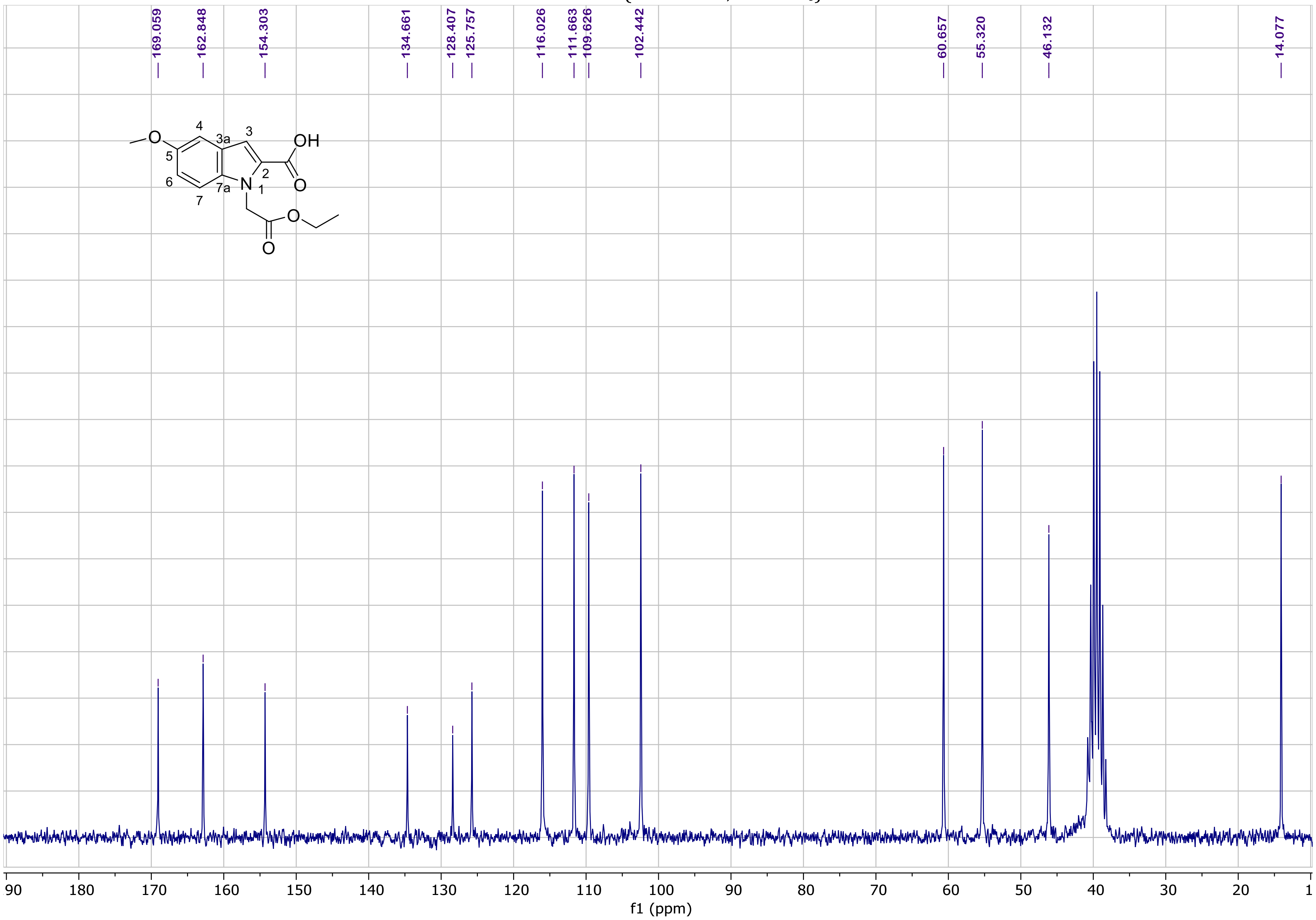

DEPT NMR of **16** (50.32 MHz, DMSO-d_6)

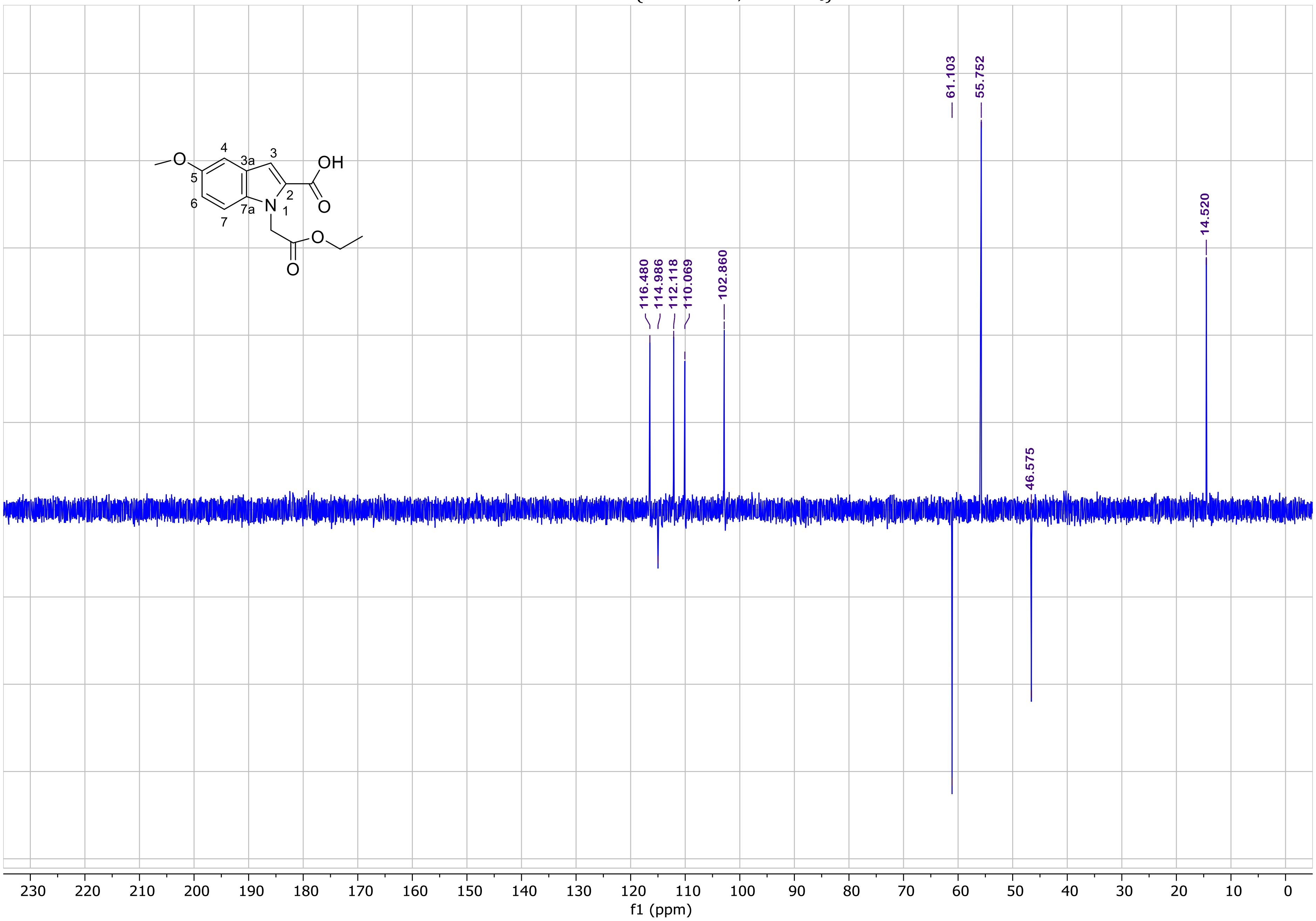

COSY NMR of **16** (400.13 MHz, DMSO-d_6)

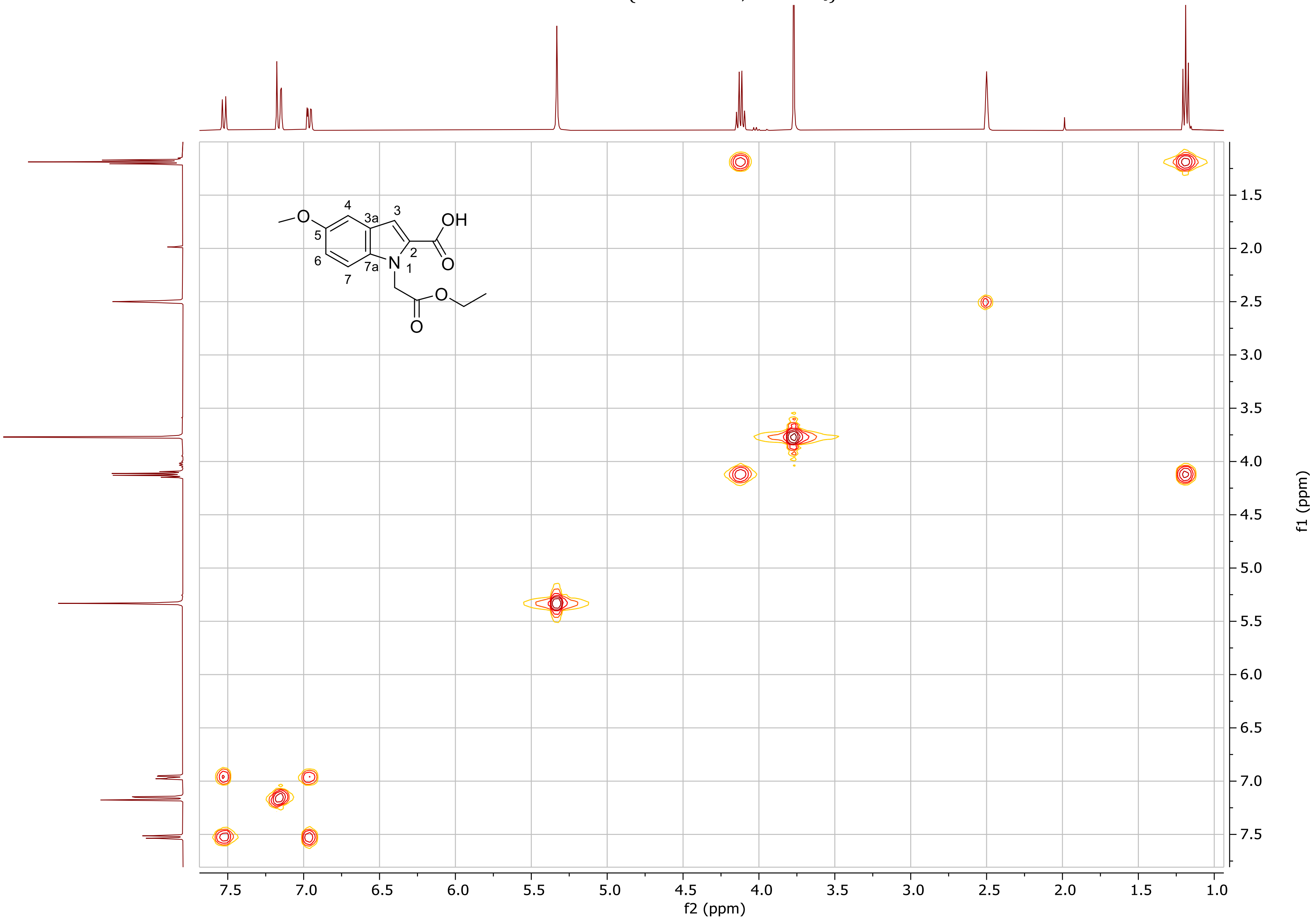

HSQC NMR of **16** (400.13 MHz, DMSO-d_6)

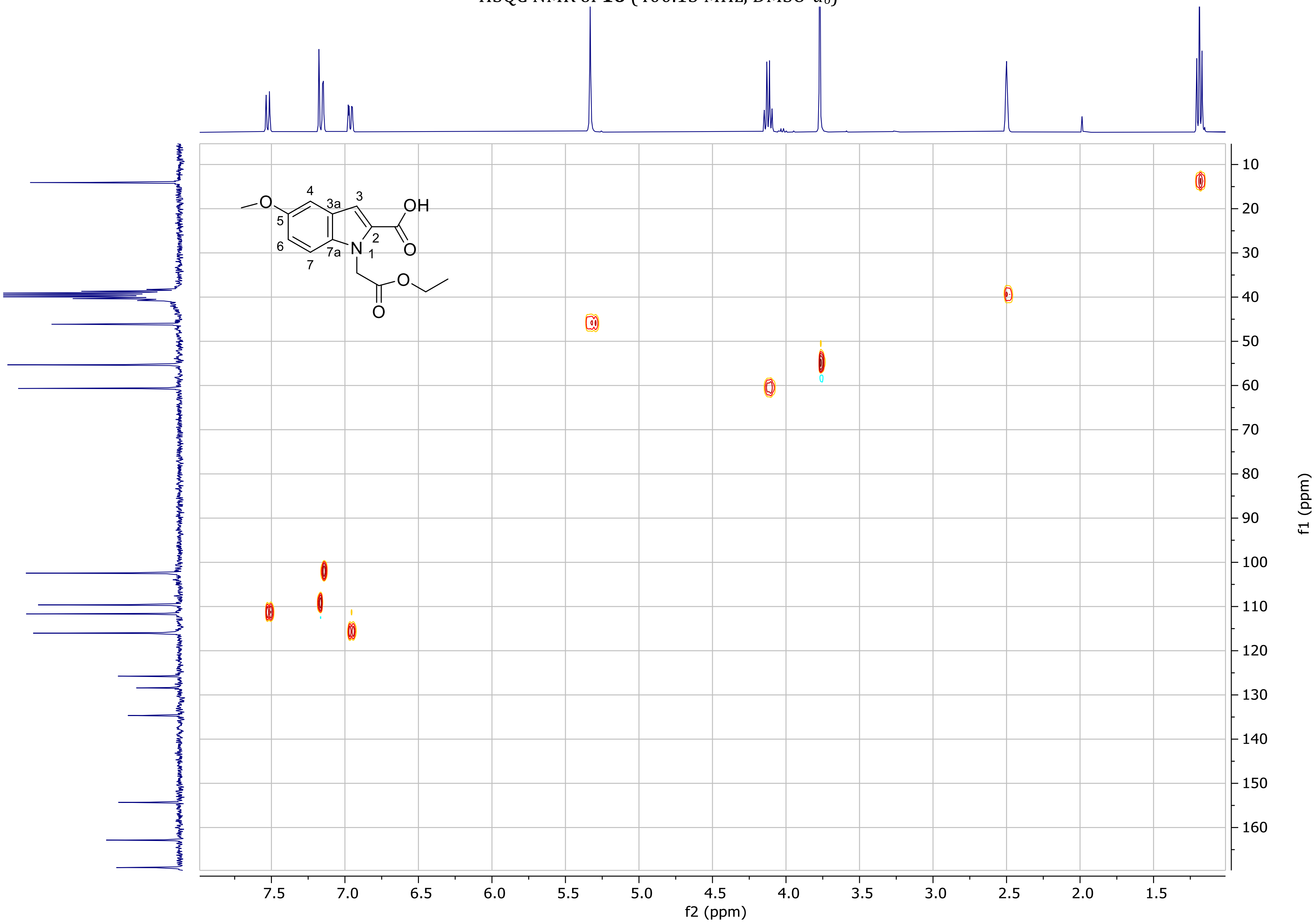

HMBC NMR of **16** (400.13 MHz, DMSO-d_6)

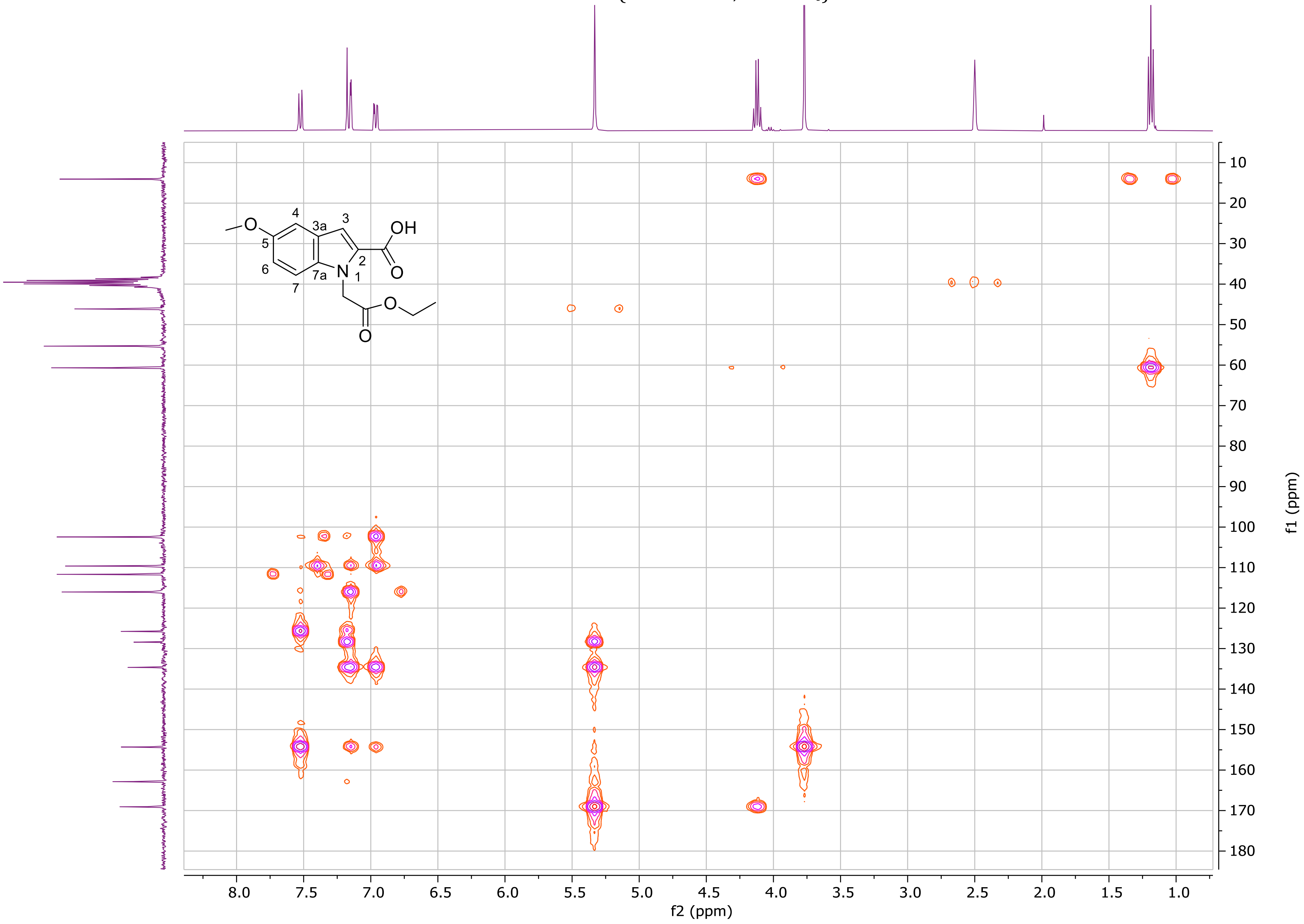

^{1}H NMR of **22** (400.13 MHz, $CDCl_3$)

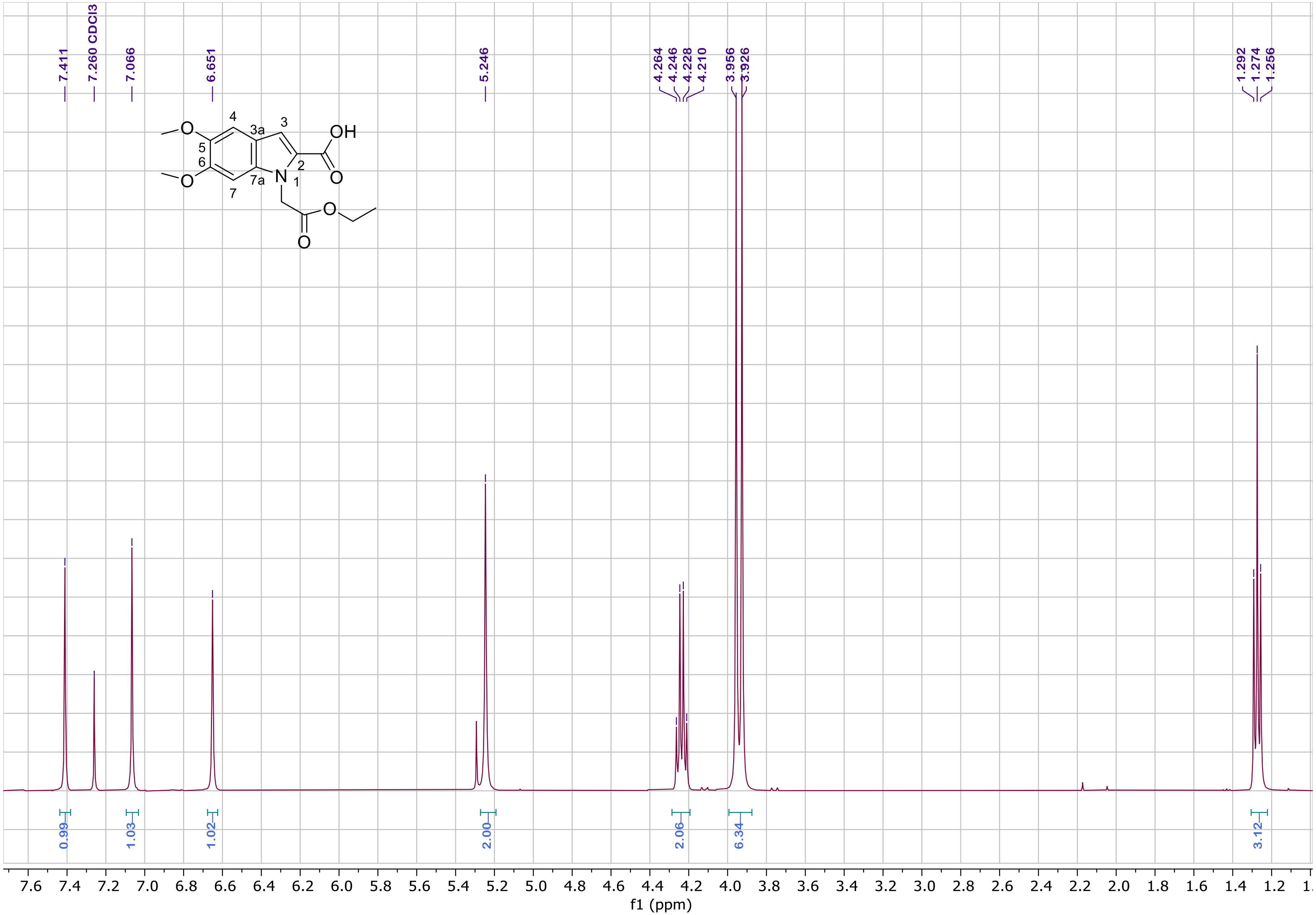

^{13}C NMR of **22** (50.32 MHz, $CDCl_3$)

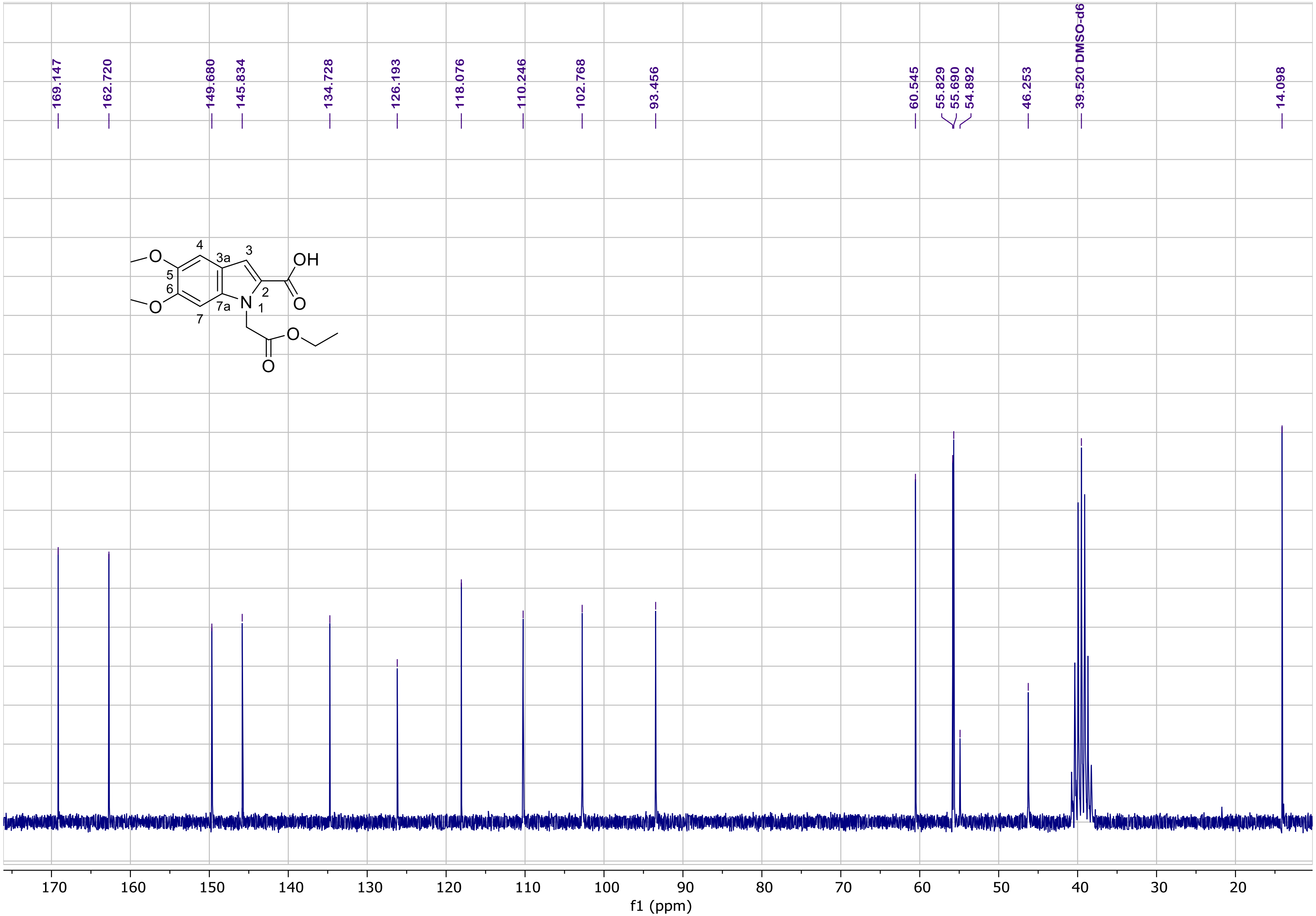

DEPT NMR of **22** (50.32 MHz, $CDCl_3$)

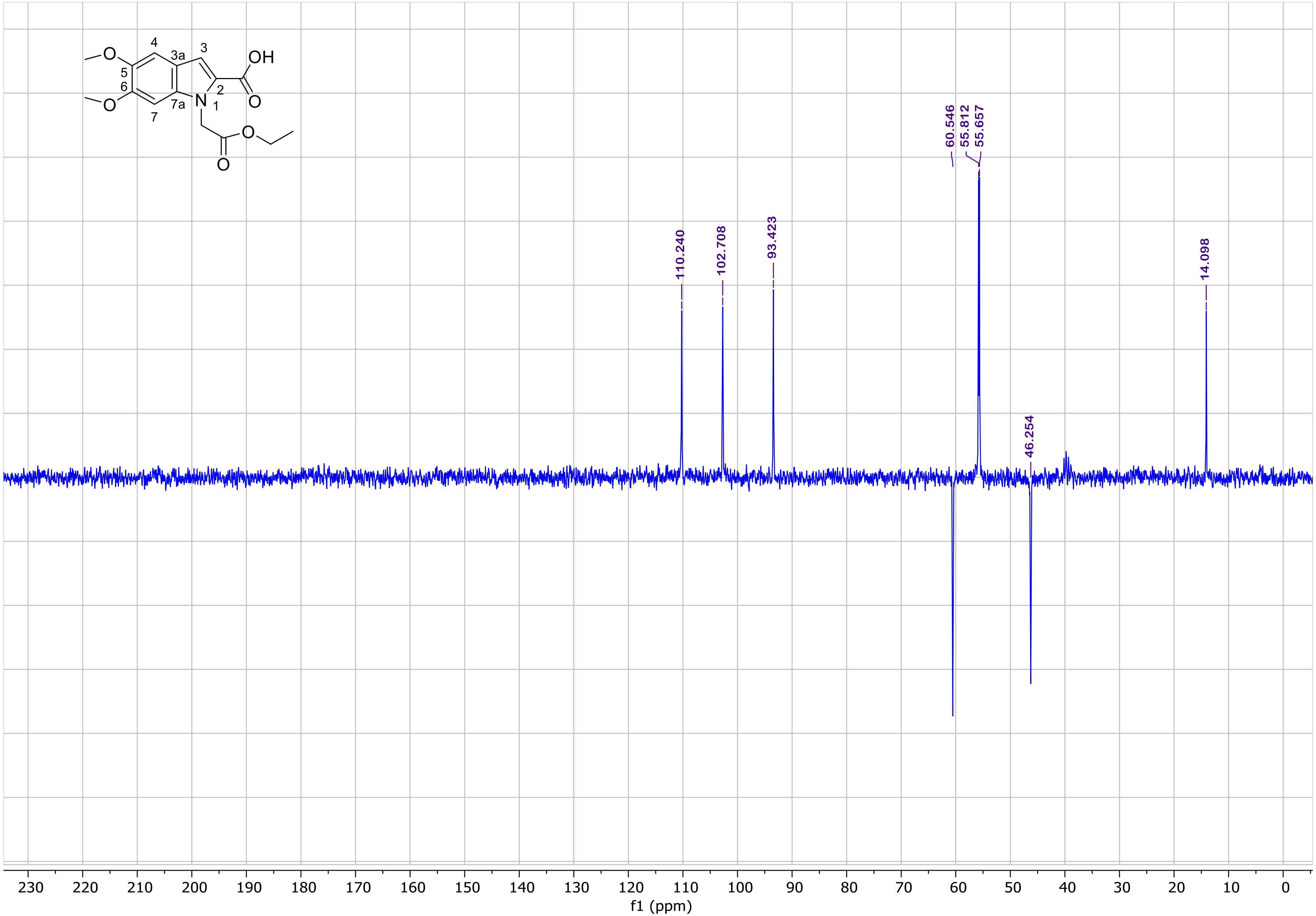

COSY NMR of **22** (400.13 MHz, $CDCl_3$)

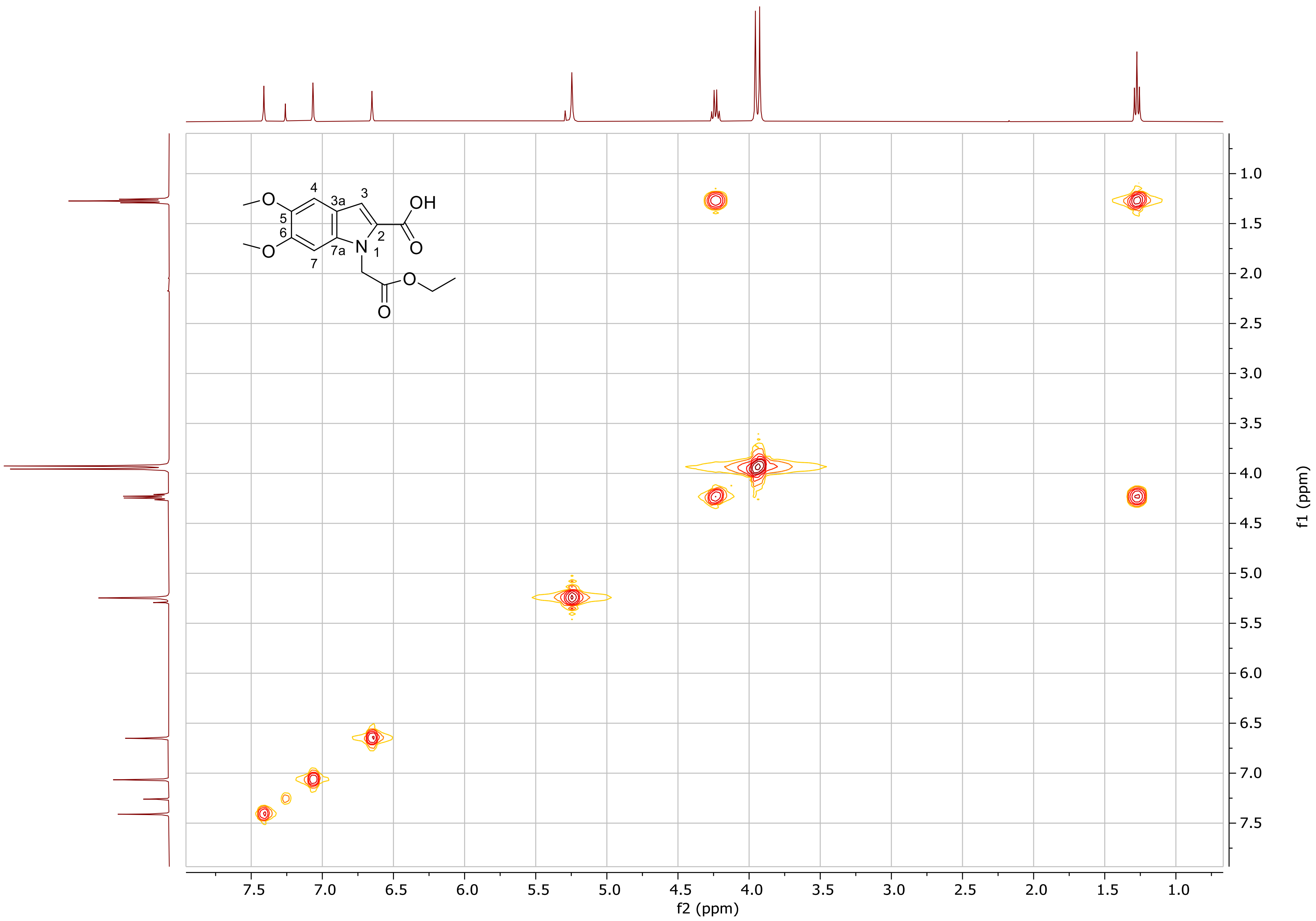

HSQC NMR of **22** (400.13 MHz, CDCl3)

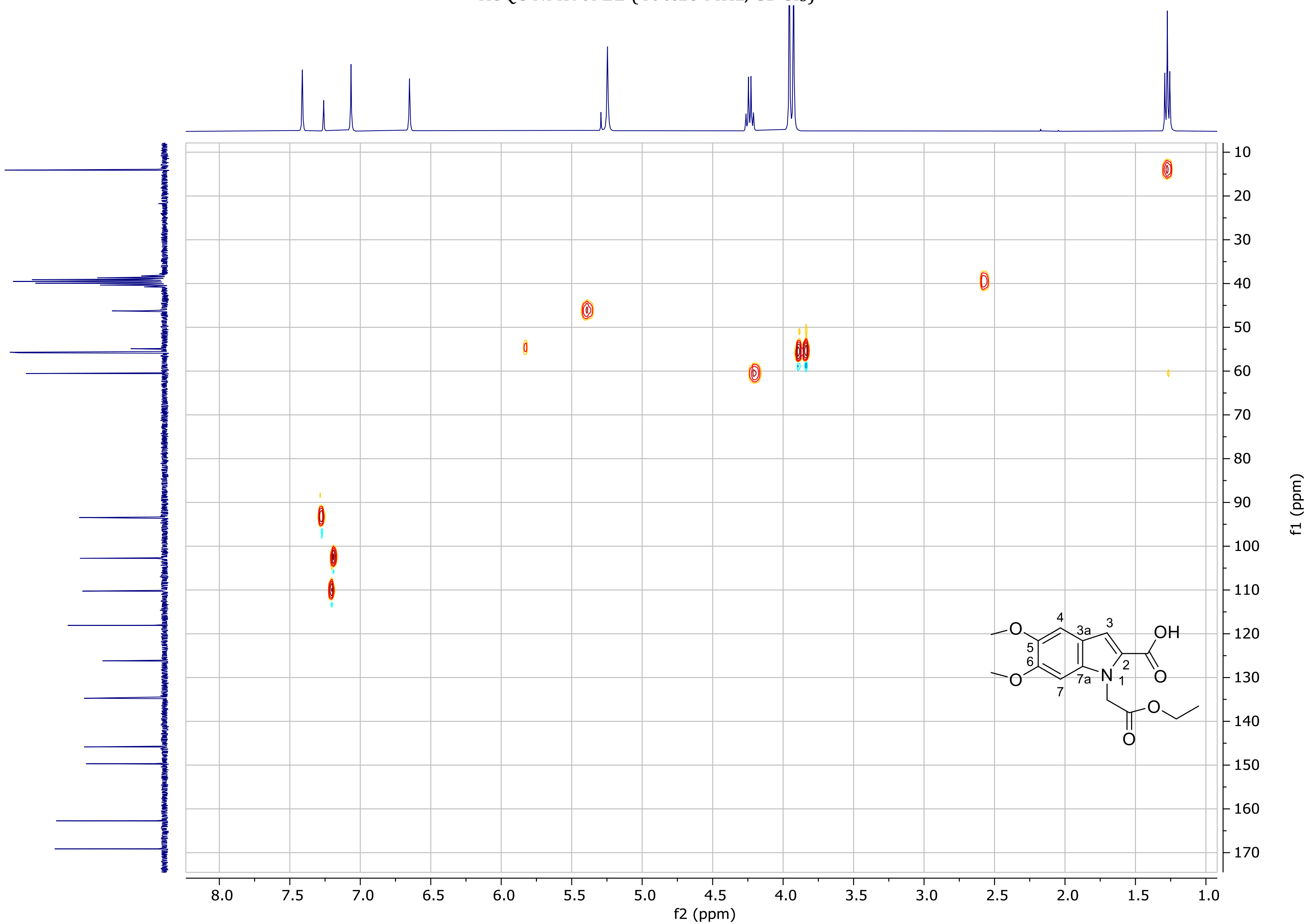

HMBC NMR of **22** (400.13 MHz, $CDCl_3$)

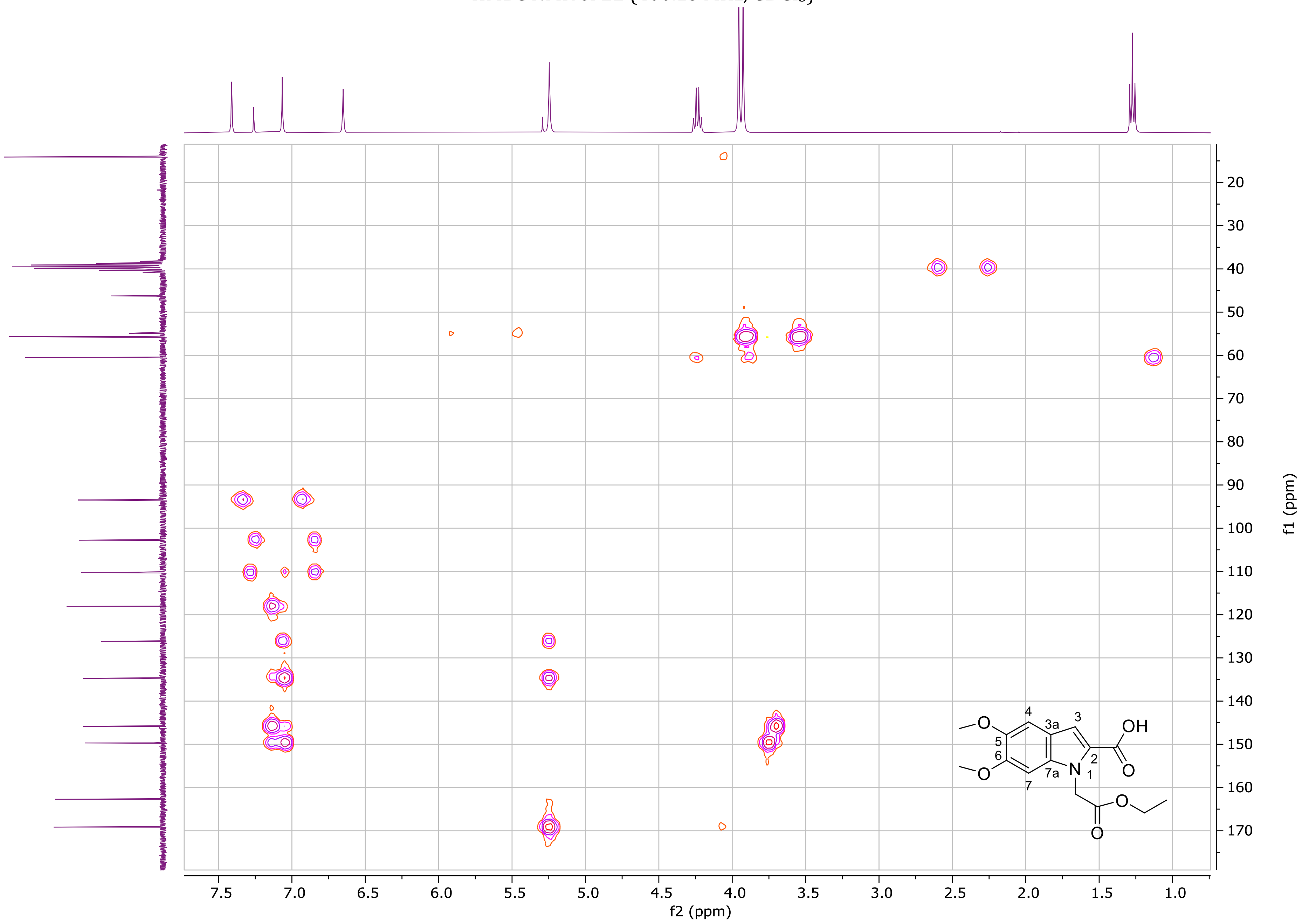

^{1}H NMR of **28** (400.13 MHz, DMSO-d_6)

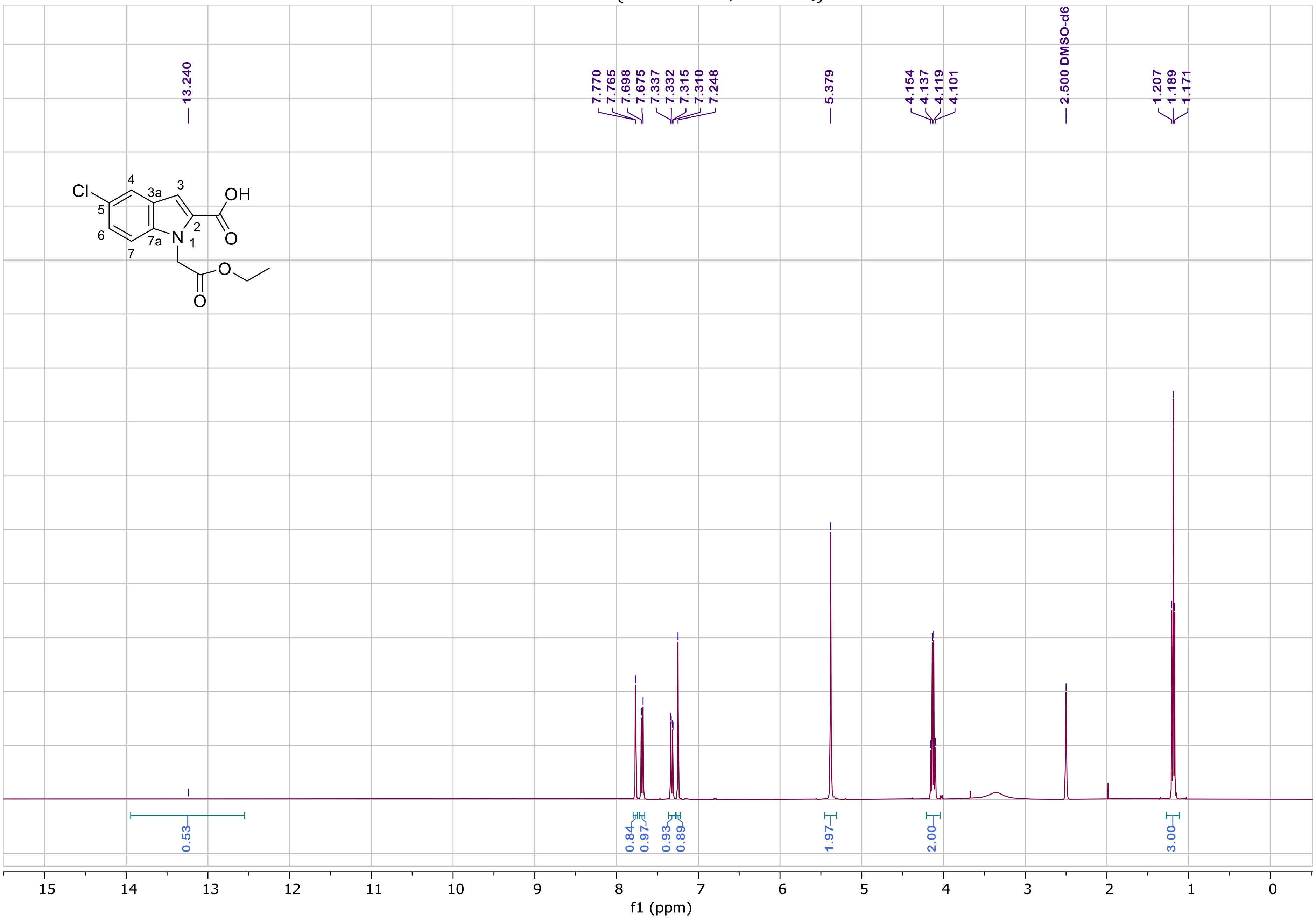

^{13}C NMR of **28** (50.32 MHz, DMSO-d_6)

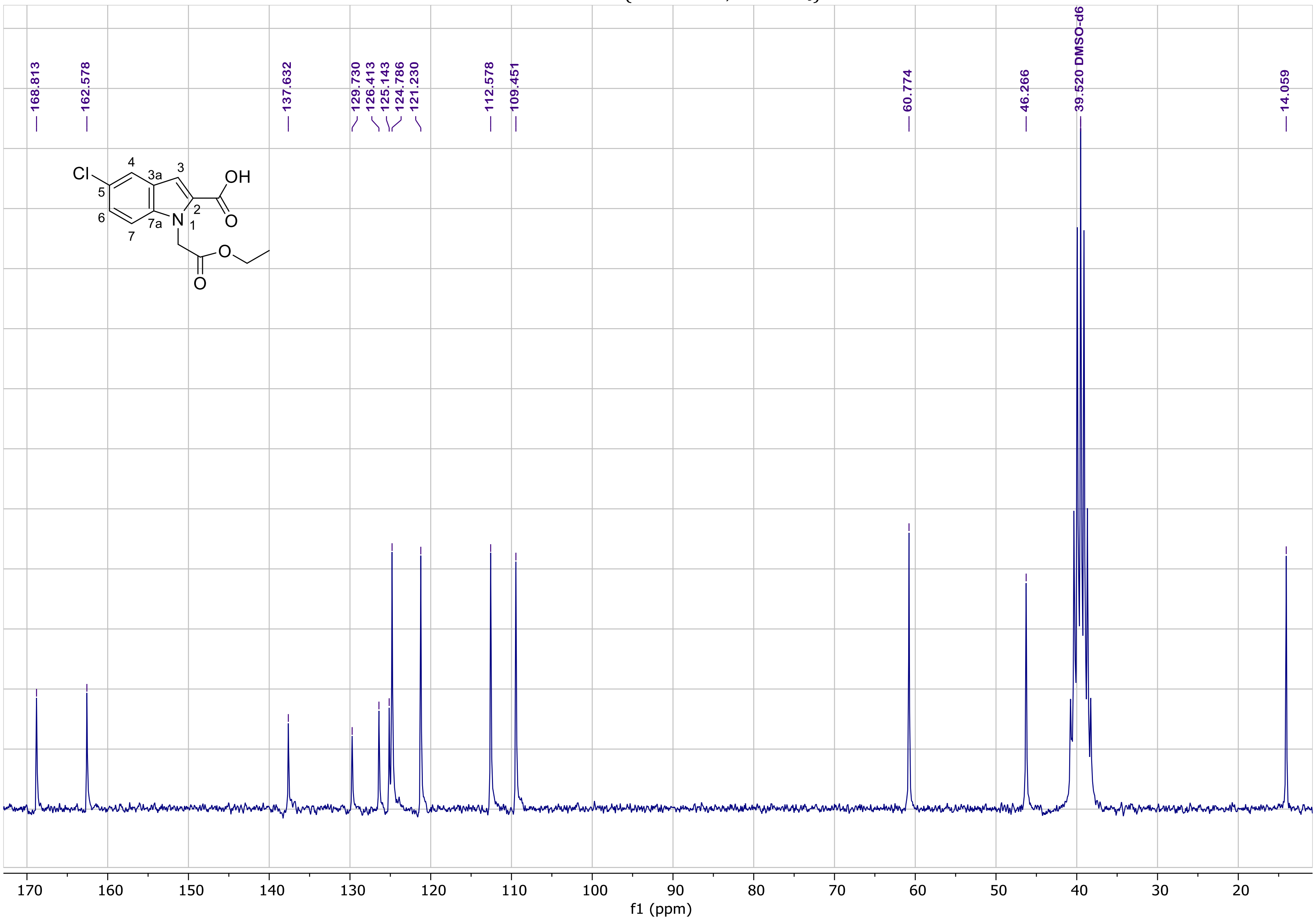

COSY NMR of **28** (400.13 MHz, DMSO-d_6)

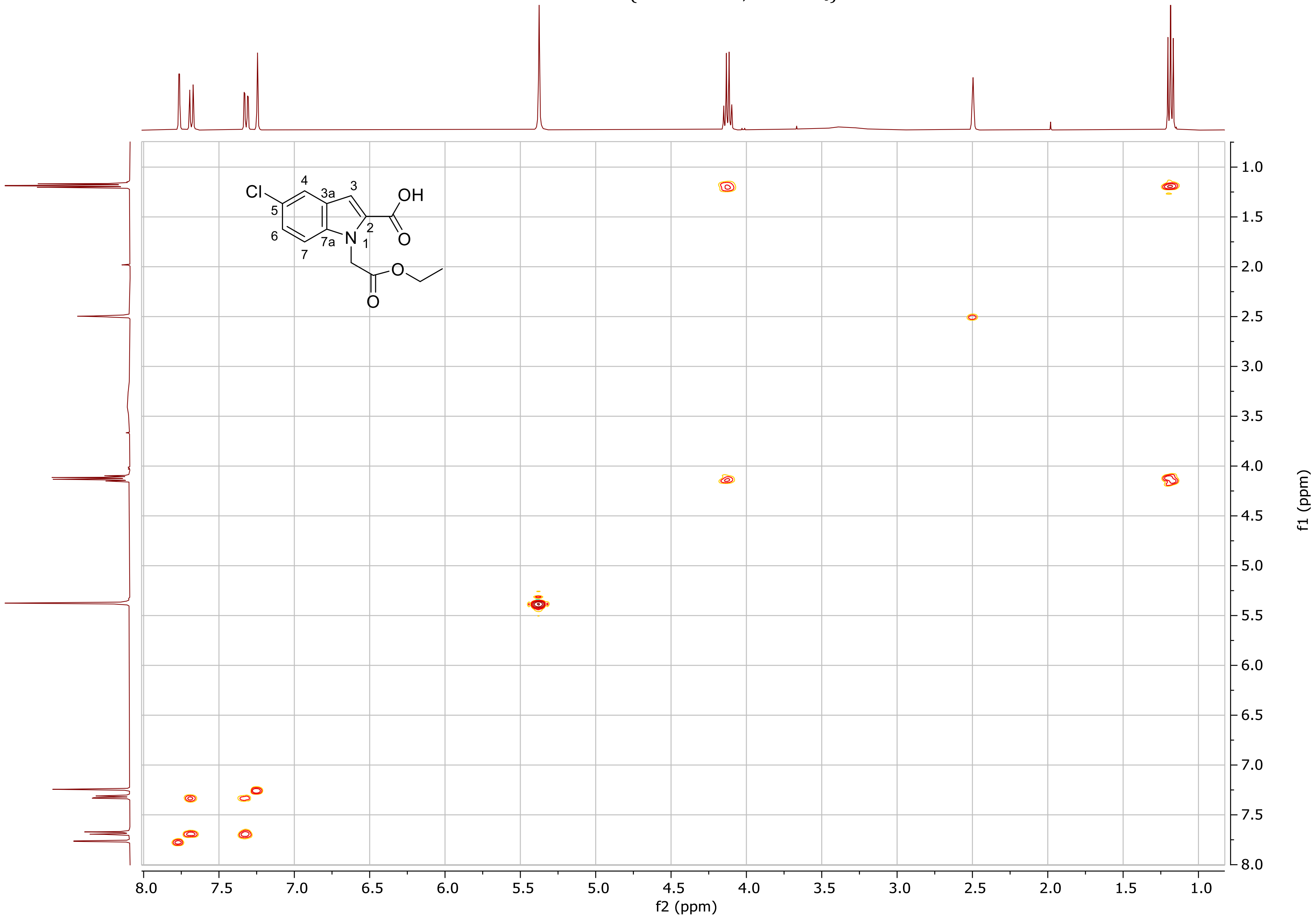

HSQC NMR of **28** (400.13 MHz, DMSO-d_6)

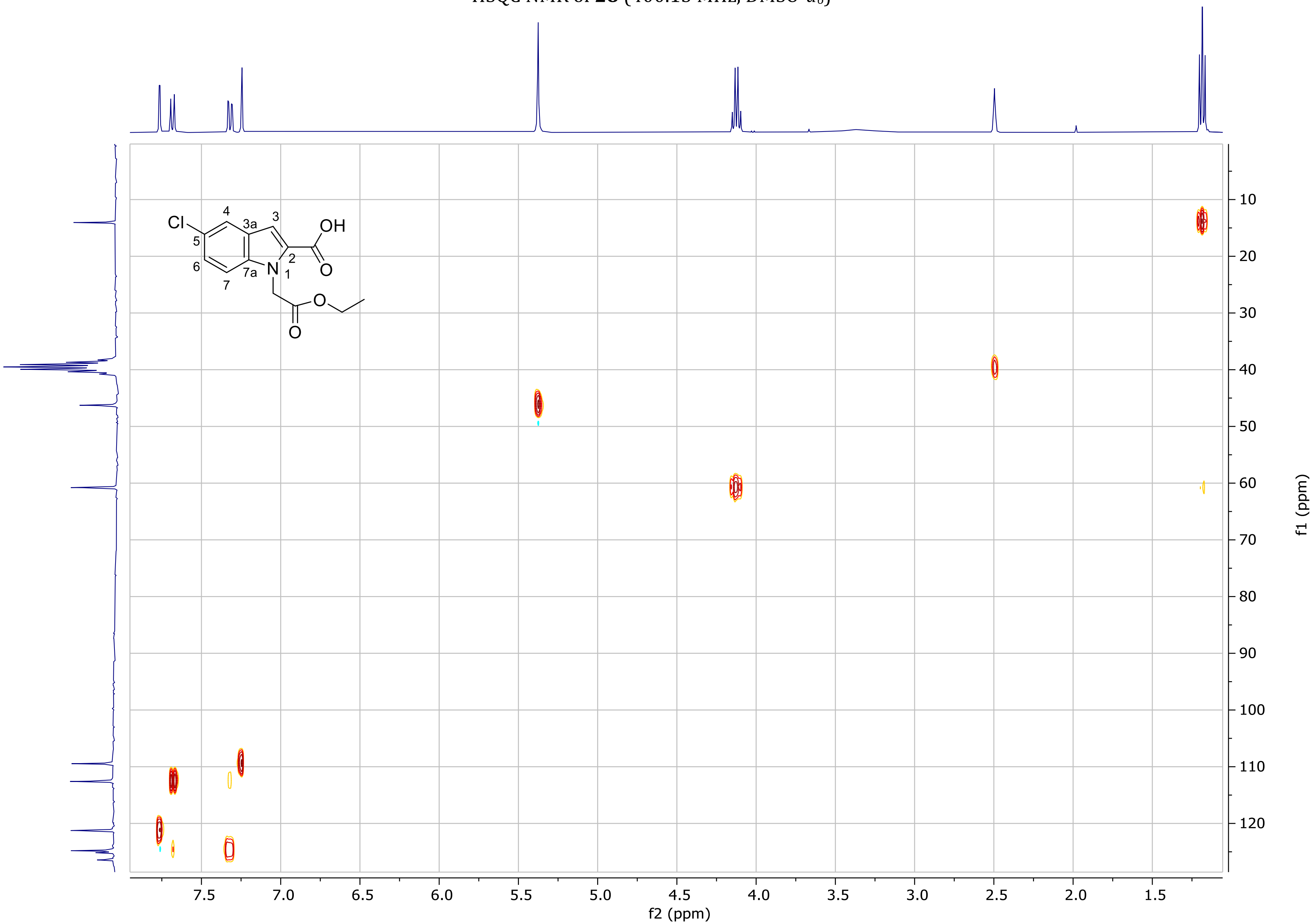

HMBC NMR of **28** (400.13 MHz, DMSO-d_6)

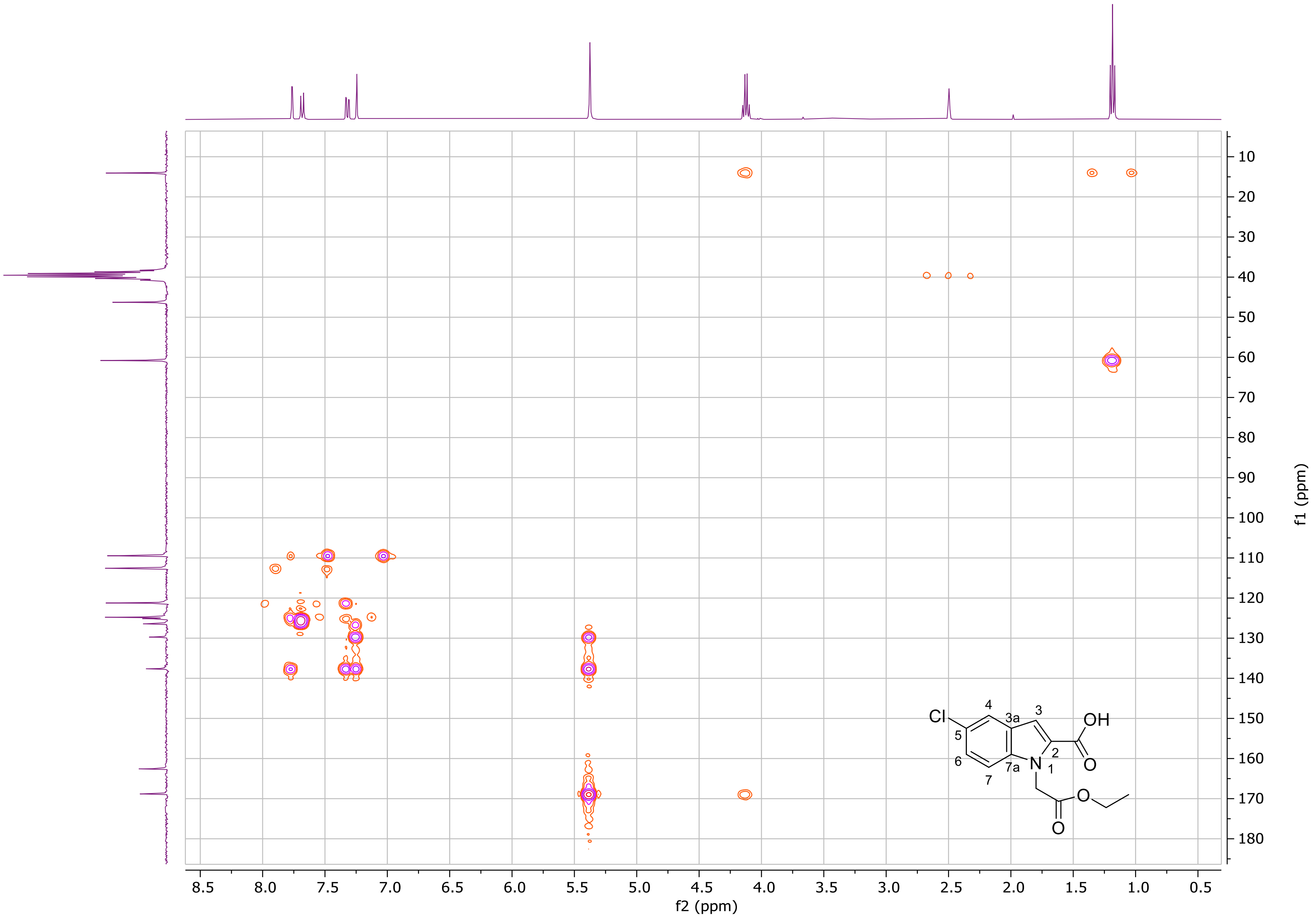

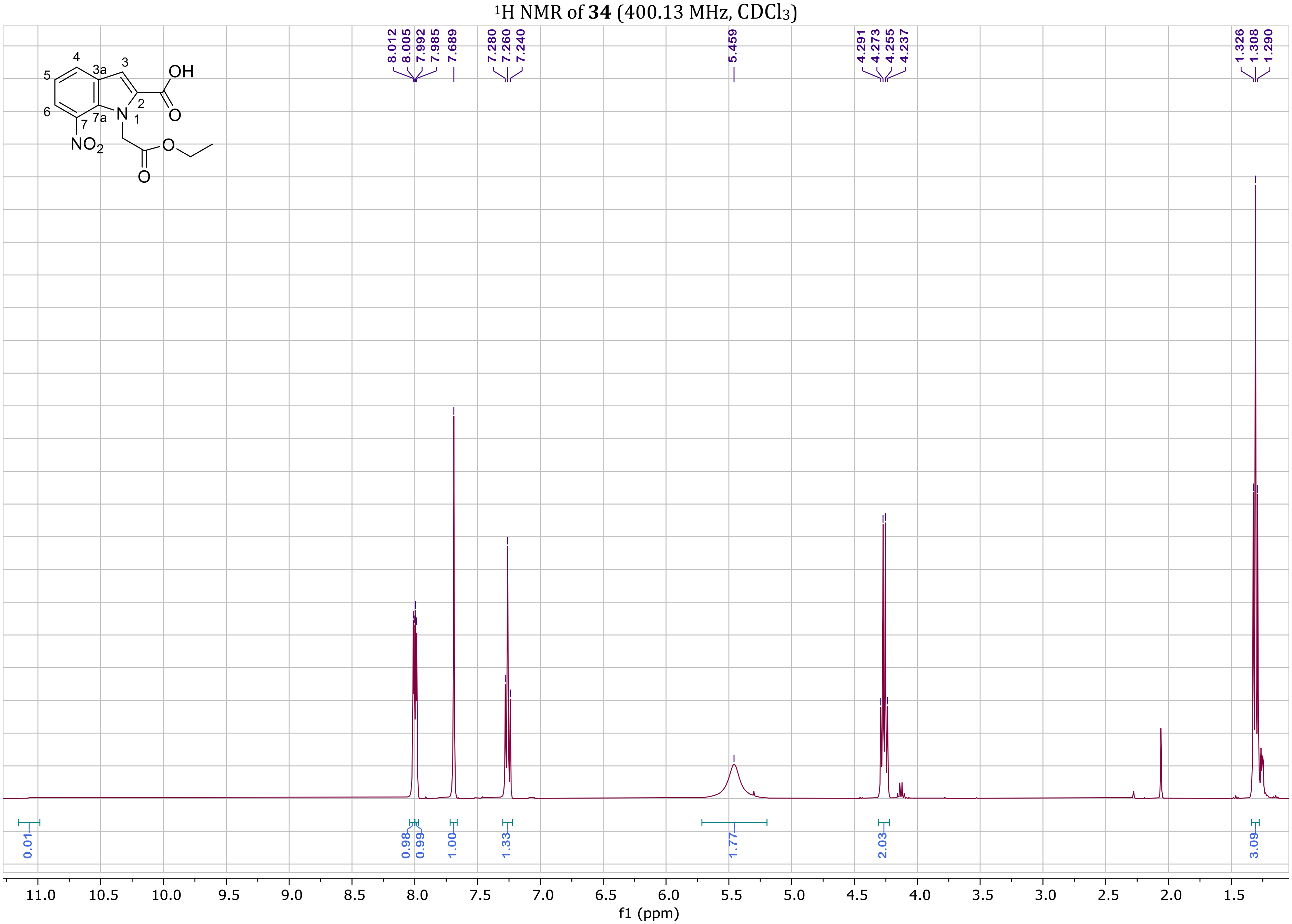
1H NMR of 34 (400.13 MHz, CDCl3)
8.012
8.005
7.992
7.985
7.689
7.280
7.260
7.240
5.459
4.291
4.273
4.255
4.237
1.326
1.308
1.290
0.01
0.98
0.99
1.00
1.33
1.77
2.03
3.09
f1 (ppm)
OH
O
NO2
O
O
N
1
2
3
3a
4
5
6
7
7a

^{13}C NMR of **34** (50.32 MHz, $CDCl_3$)

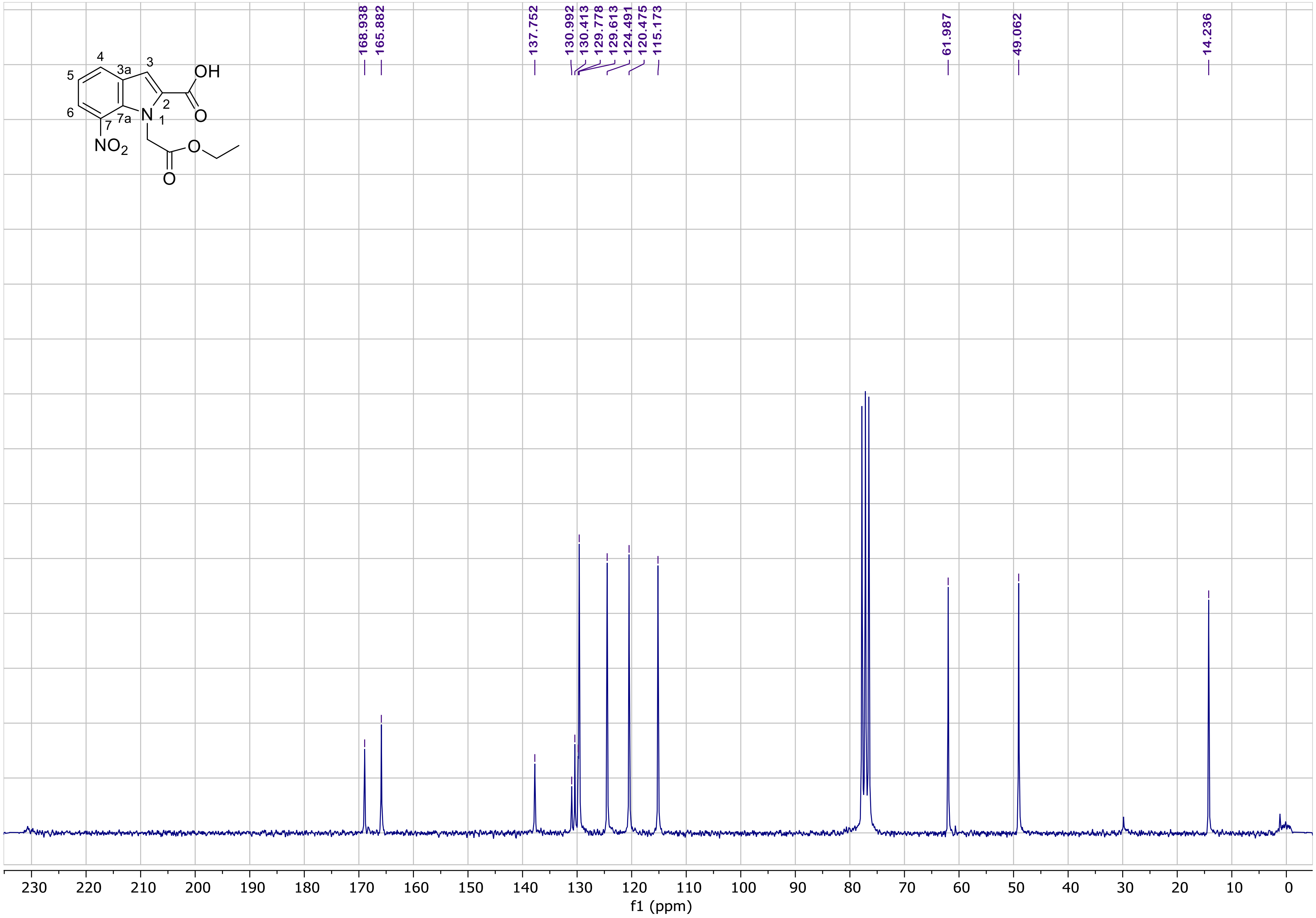

DEPT NMR of **34** (50.32 MHz, $CDCl_3$)

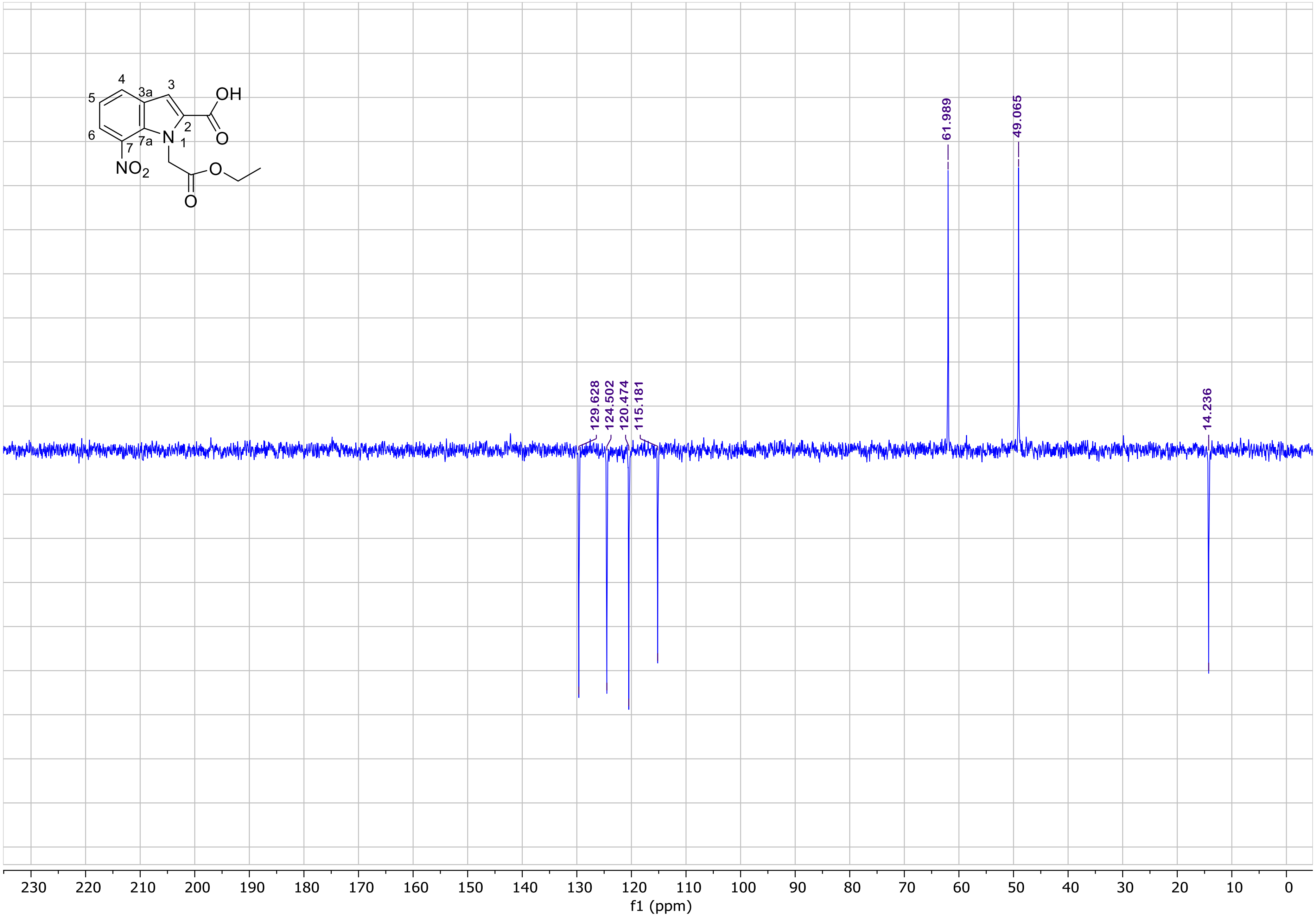

COSY NMR of **34** (400.13 MHz, $CDCl_3$)

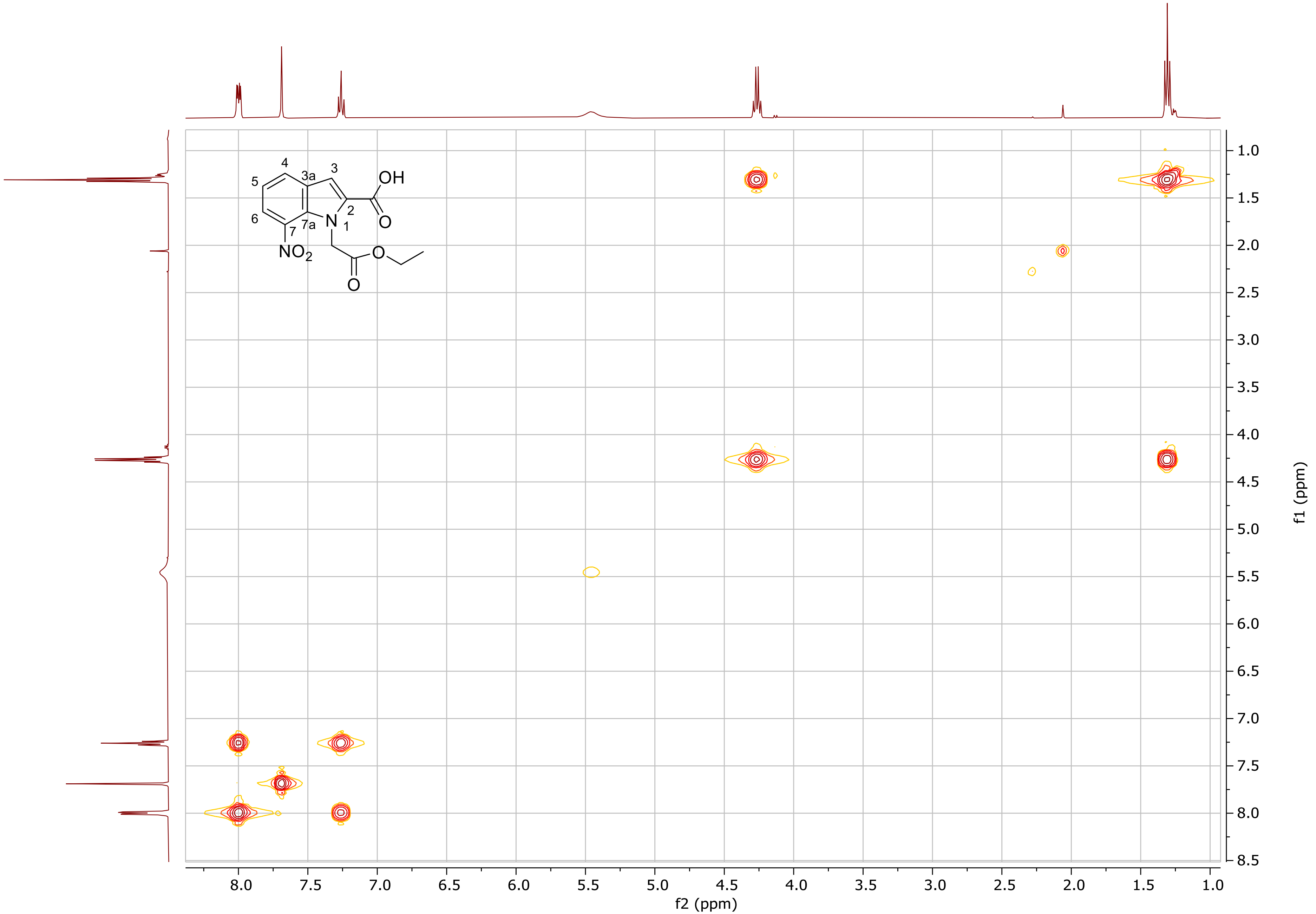

HSQC NMR of **34** (400.13 MHz, $CDCl_3$)

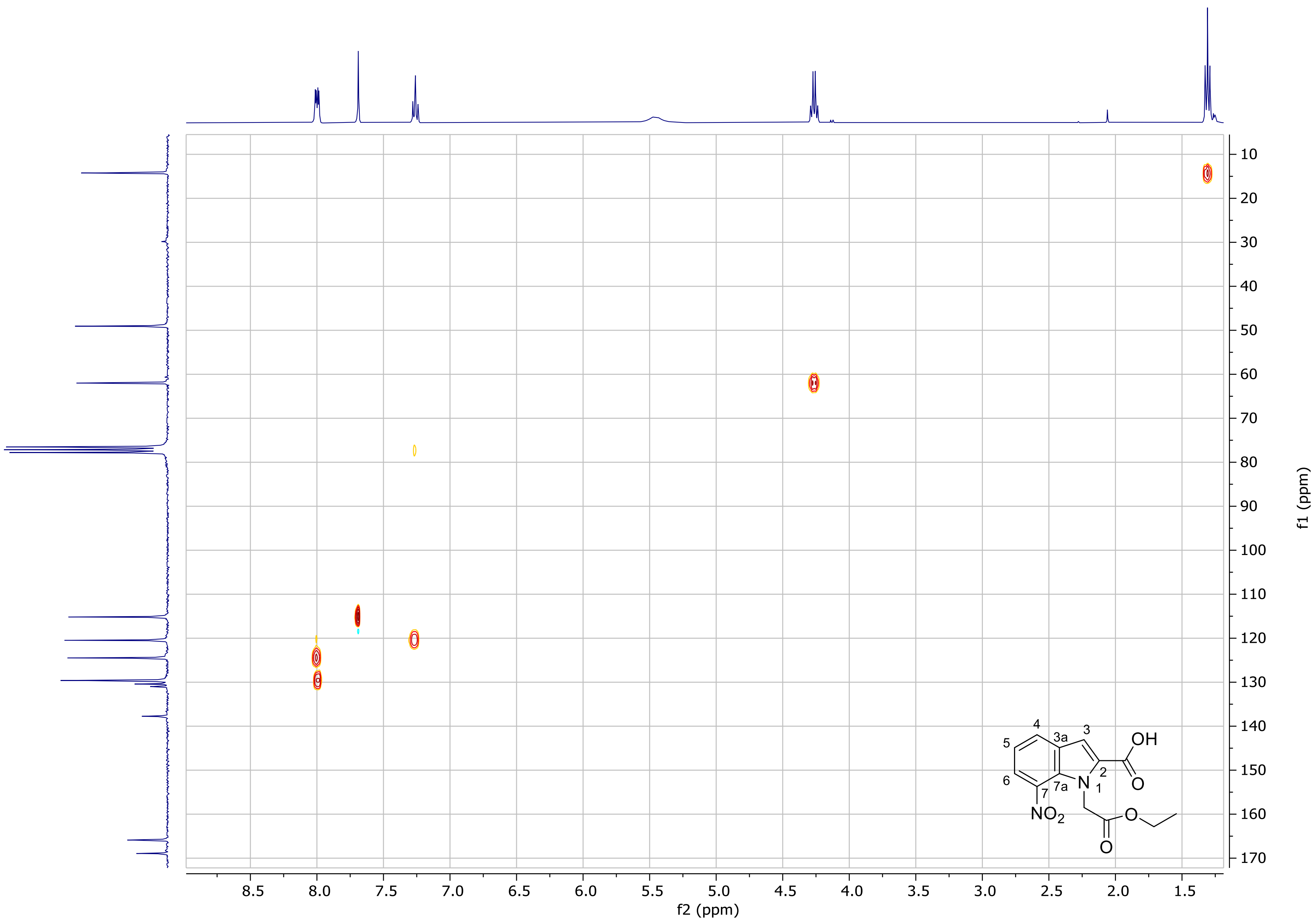

HMBC NMR of **34** (400.13 MHz, $CDCl_3$)

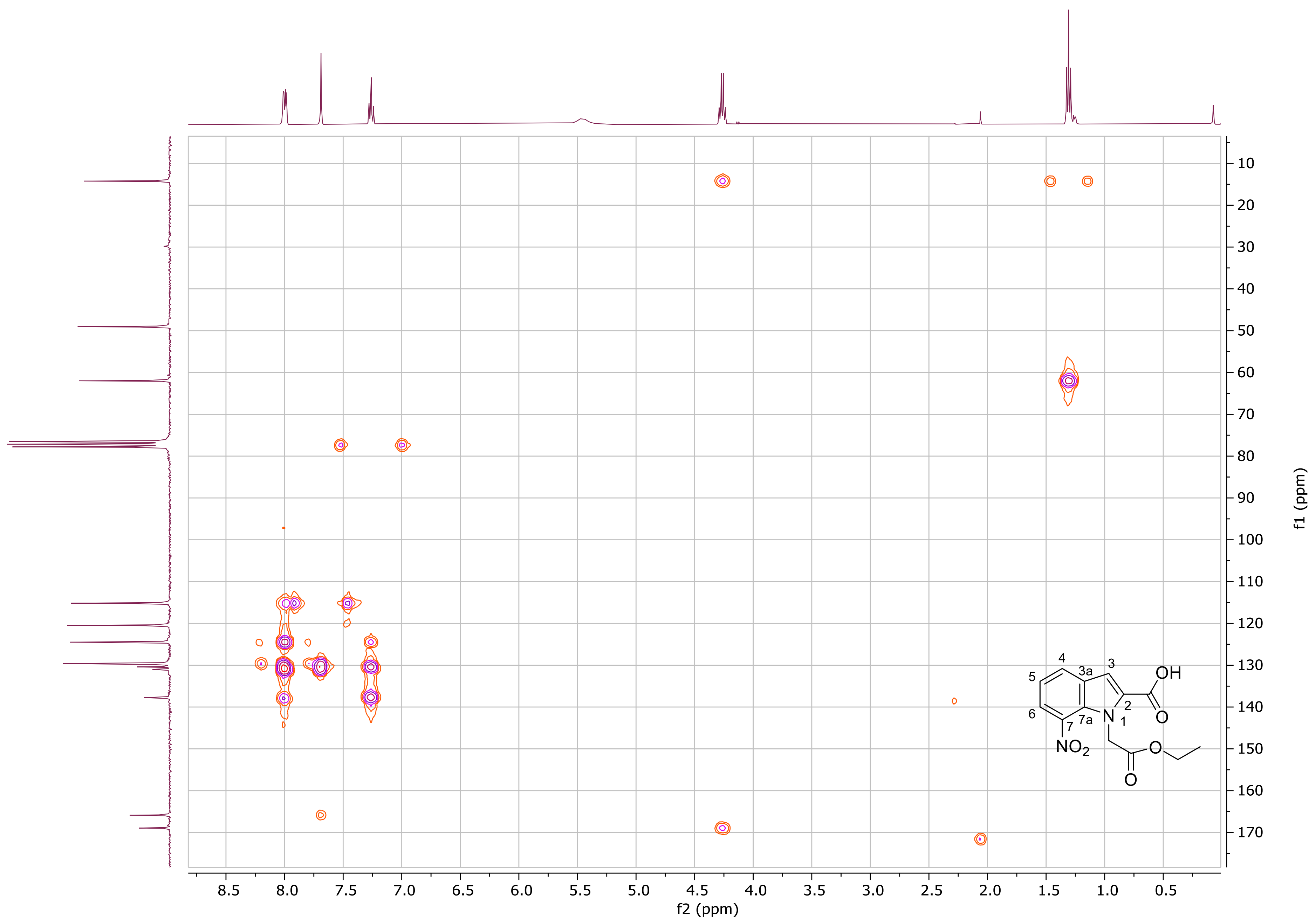

^{1}H NMR of **5** (600.11 MHz, DMSO-d_6)

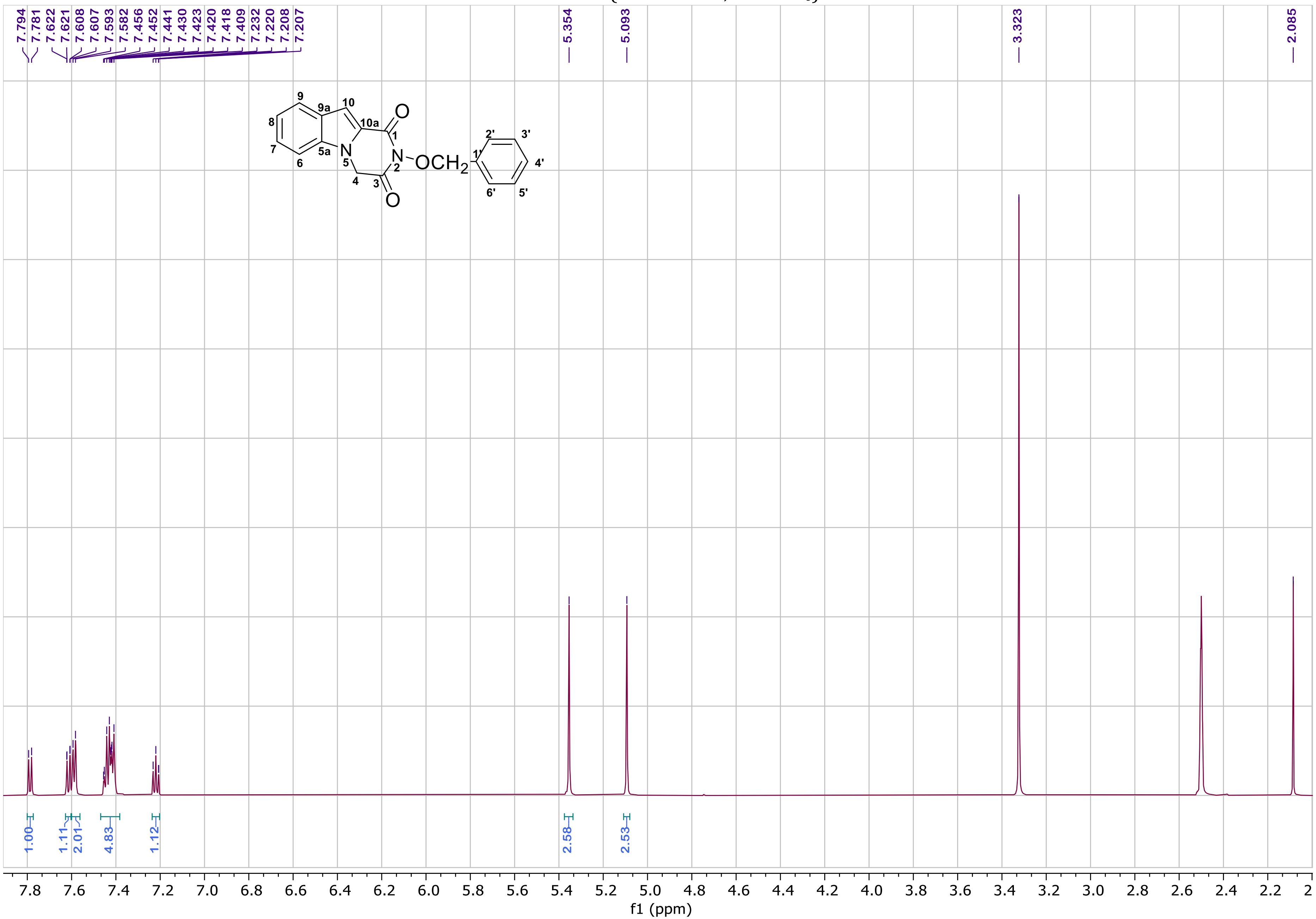

¹³C NMR of **5** (50.32 MHz, DMSO-d_6)

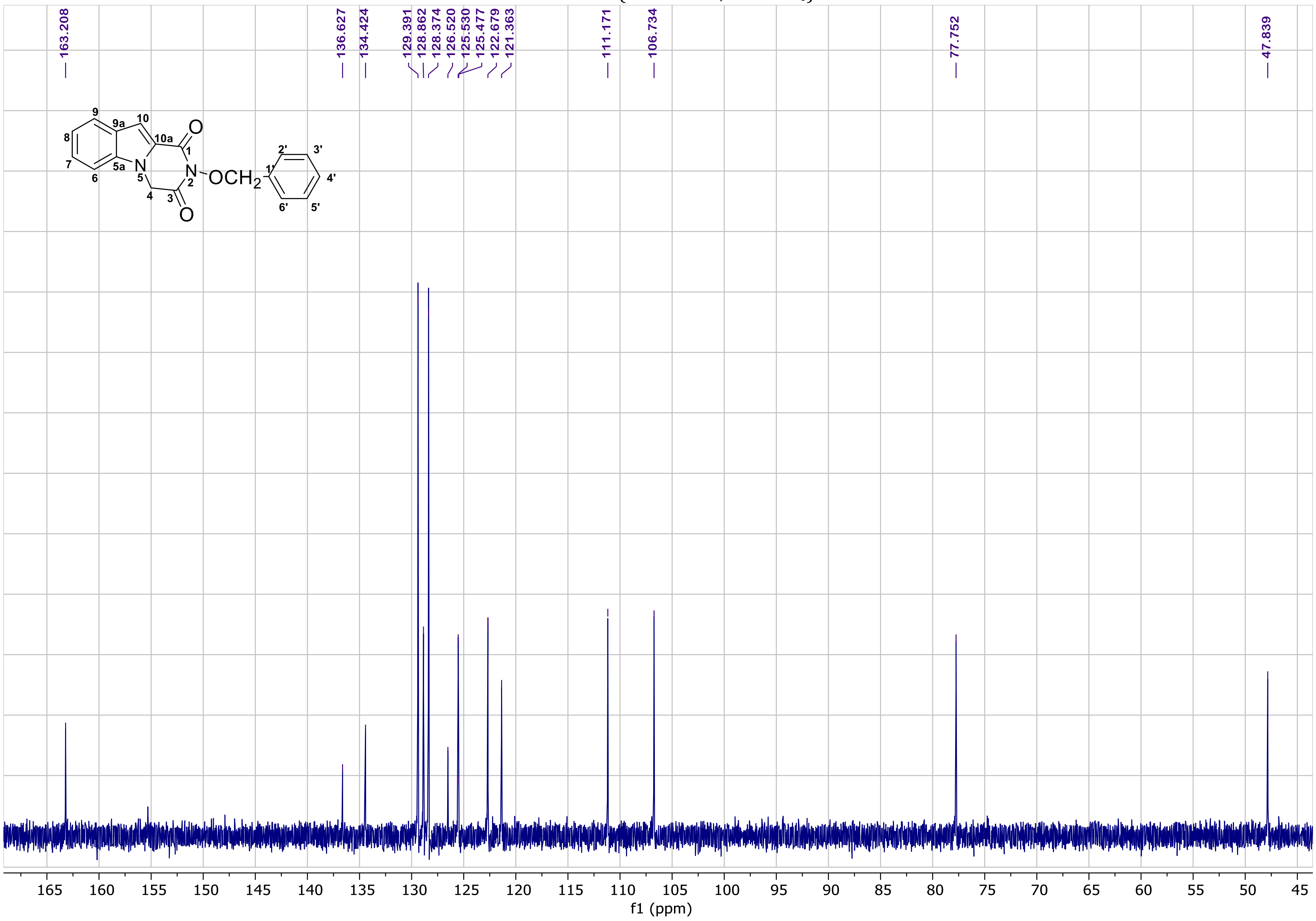

COSY NMR of **5** (600.11 MHz, DMSO-d_6)

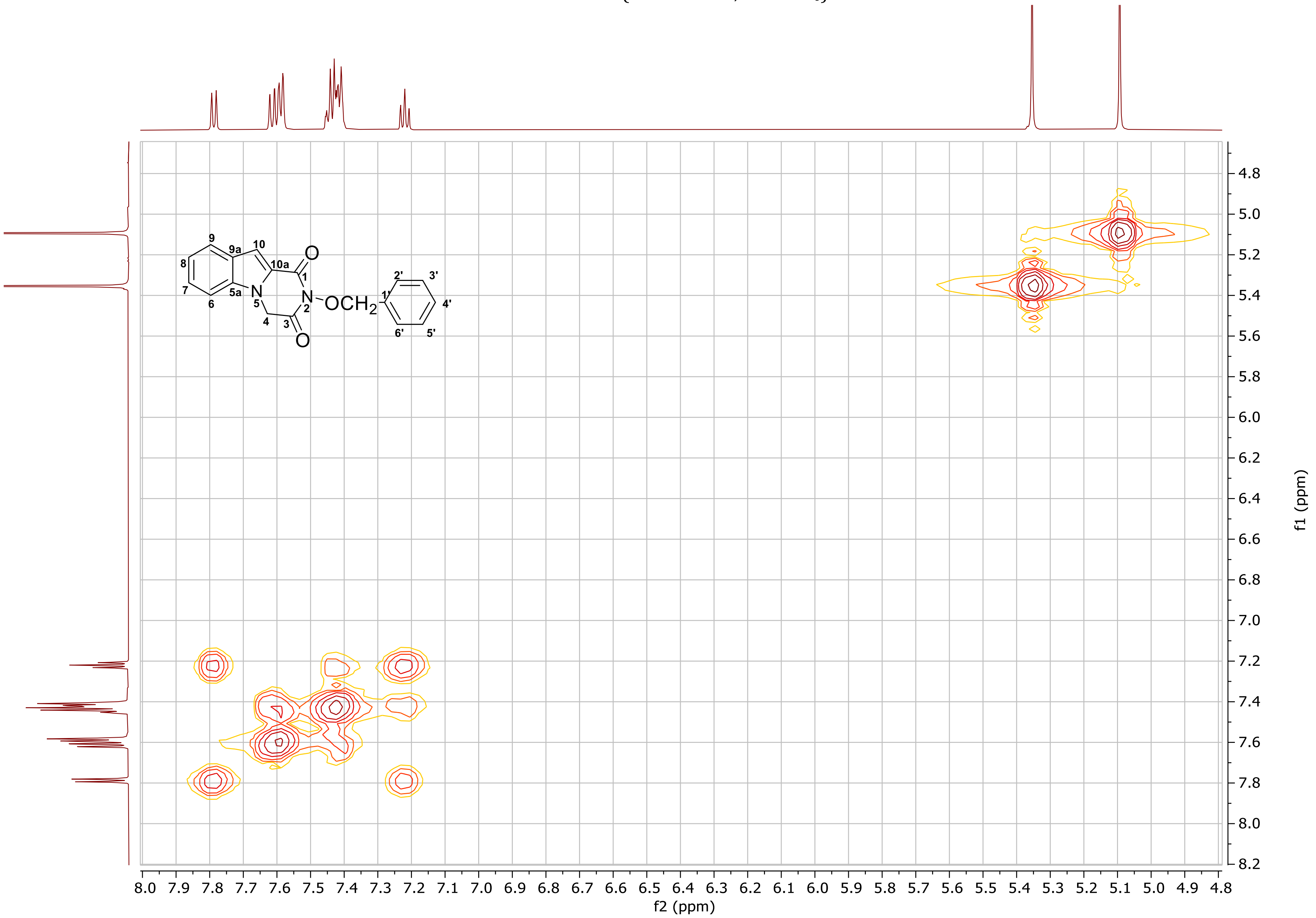

HSQC-DEPT NMR of **5** (600.11 MHz, DMSO-d_6)

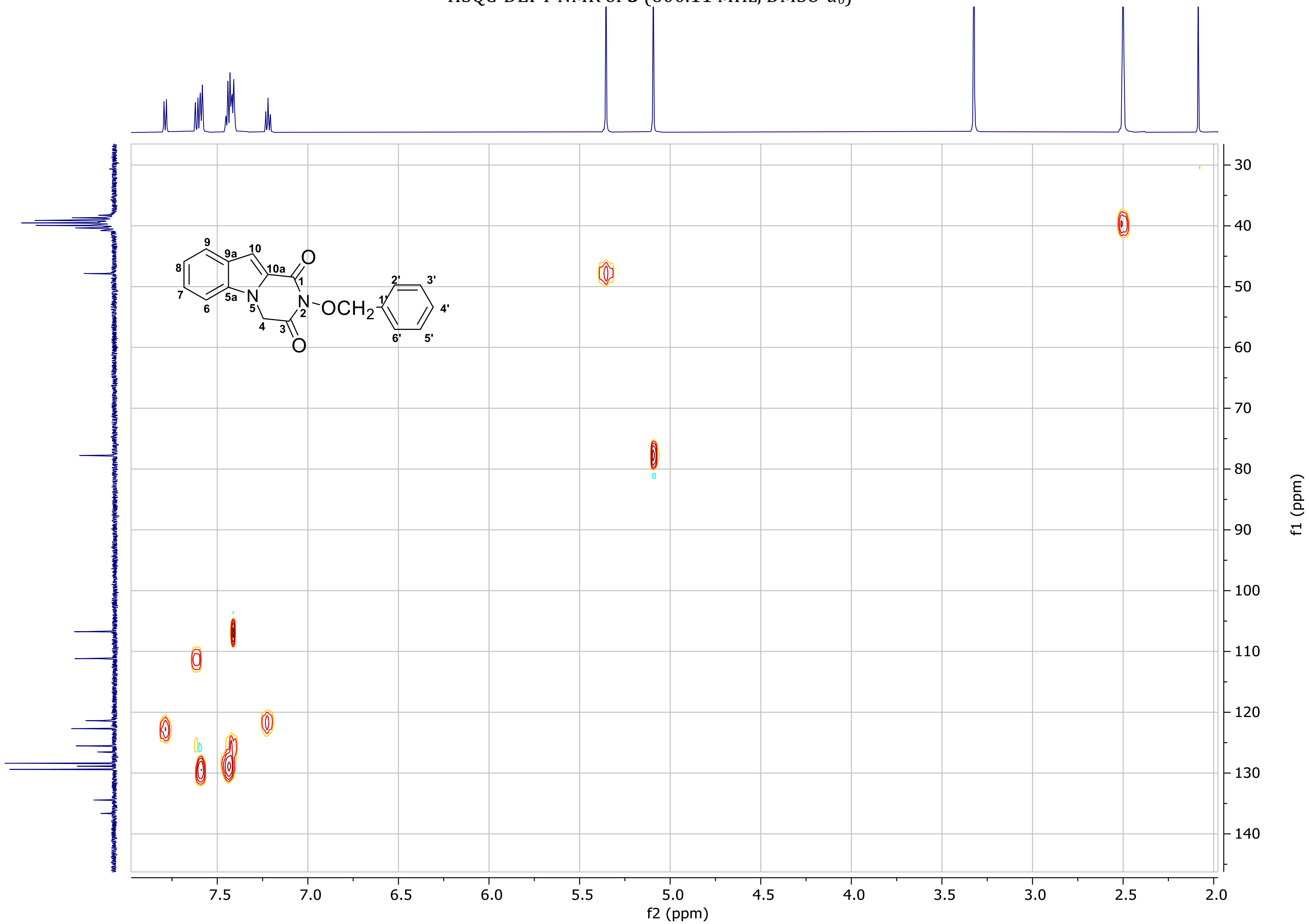

HMBC NMR of **5** (600.11 MHz, DMSO-d_6)

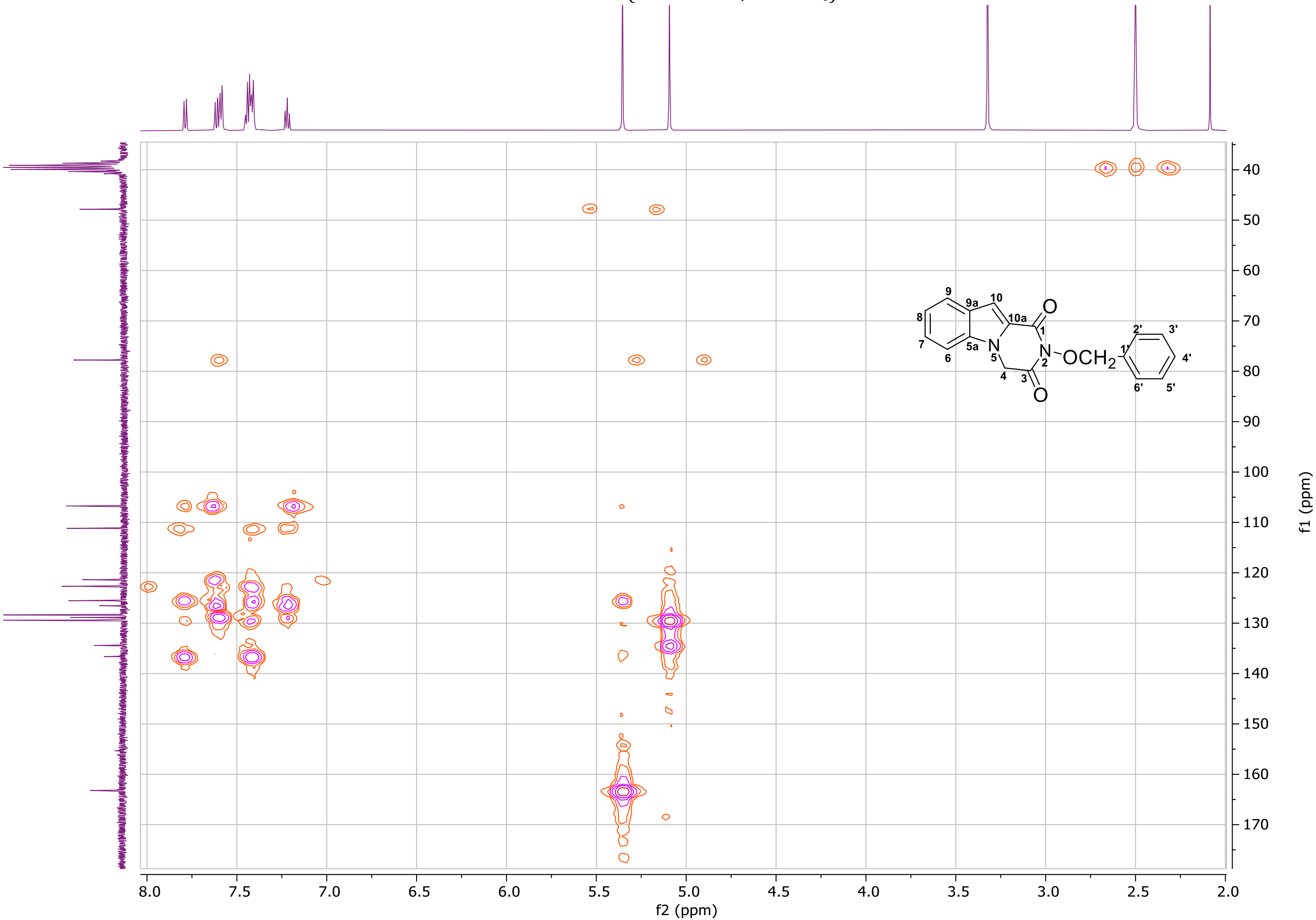

^{1}H NMR of **11** (400.13 MHz, DMSO-d_6)

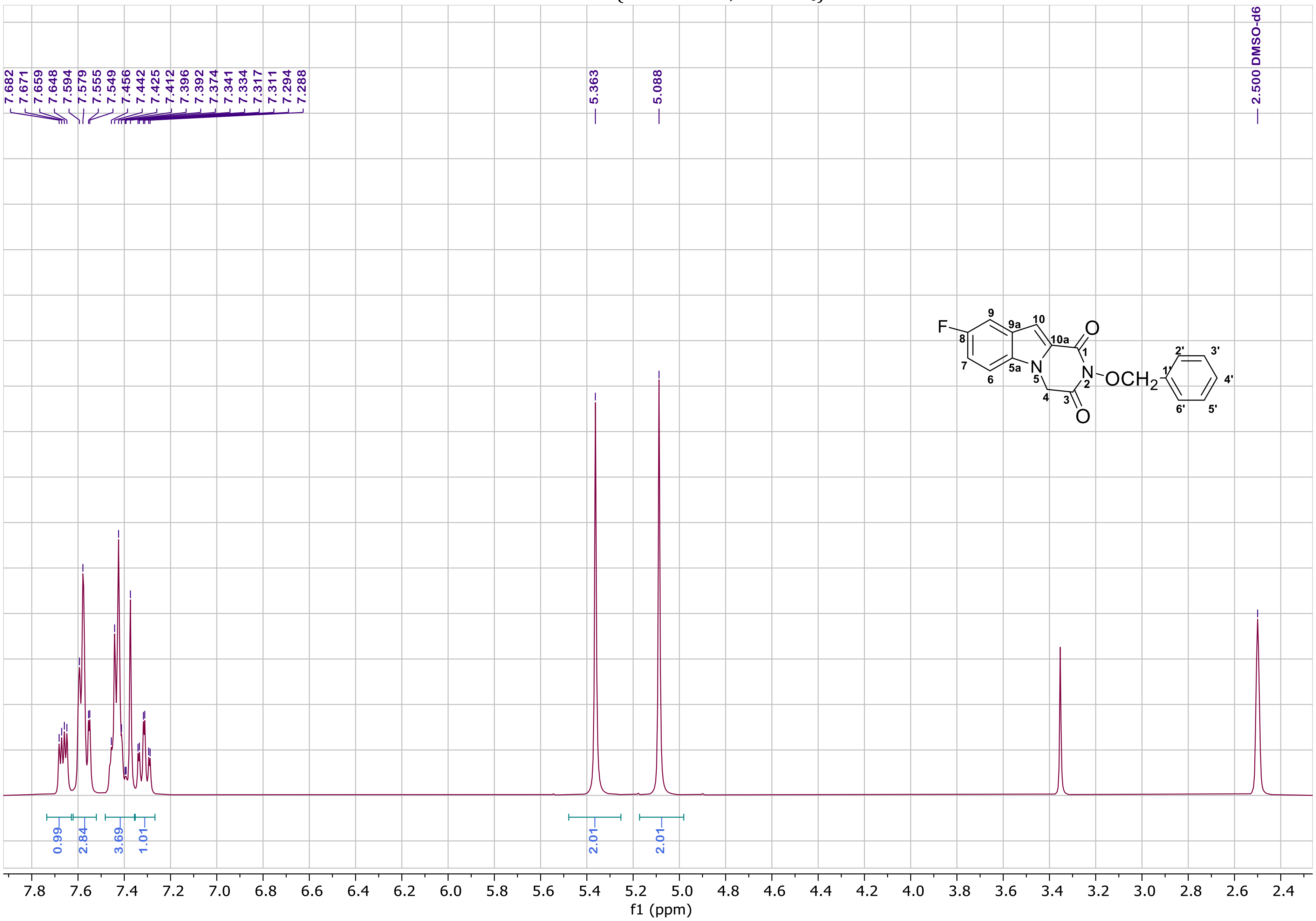

13C NMR of **11** (50.32 MHz, DMSO-d_6)

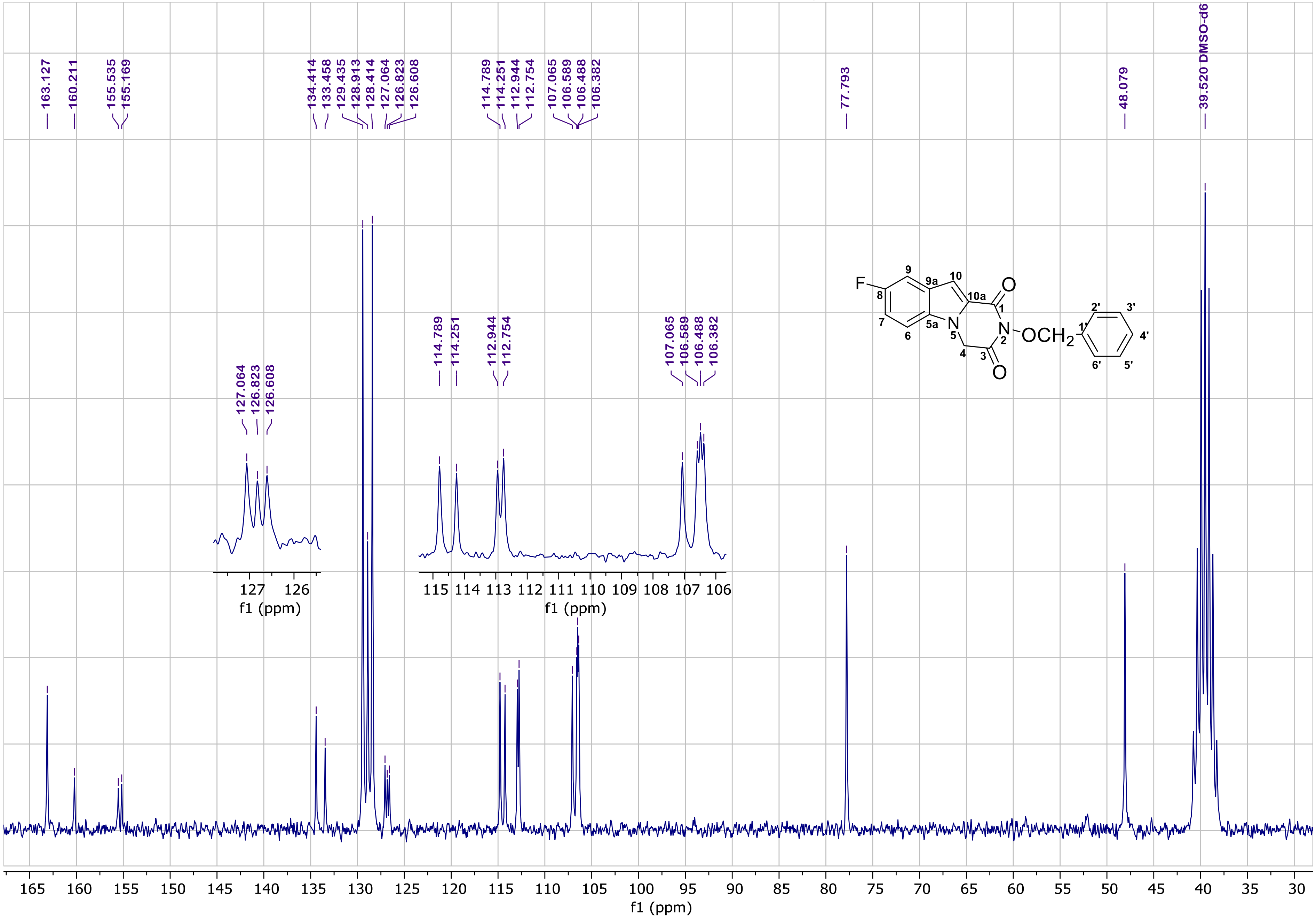

DEPT NMR of **11** (50.32 MHz, DMSO-d_6)

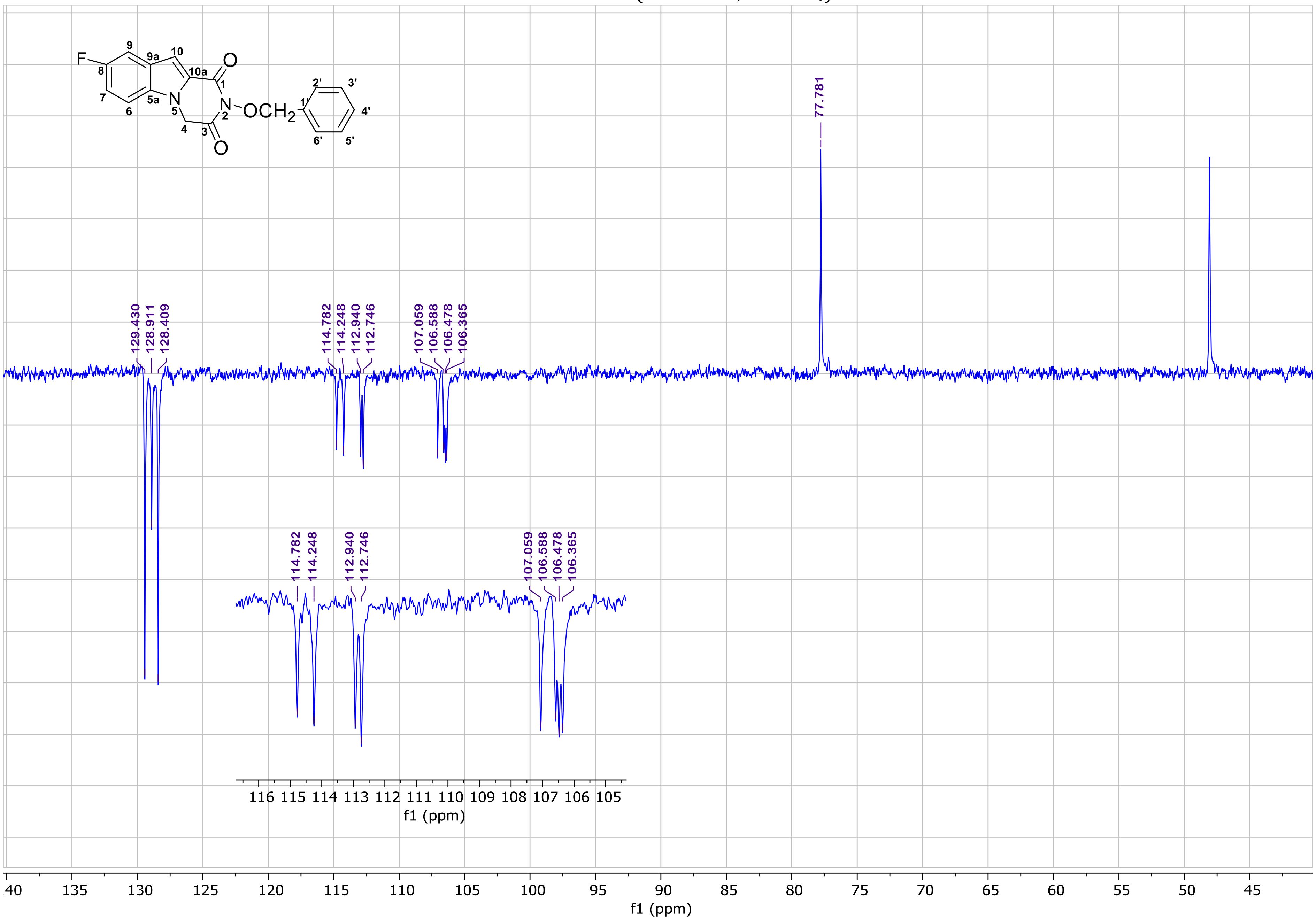

COSY NMR of **11** (400.13 MHz, DMSO-d_6)

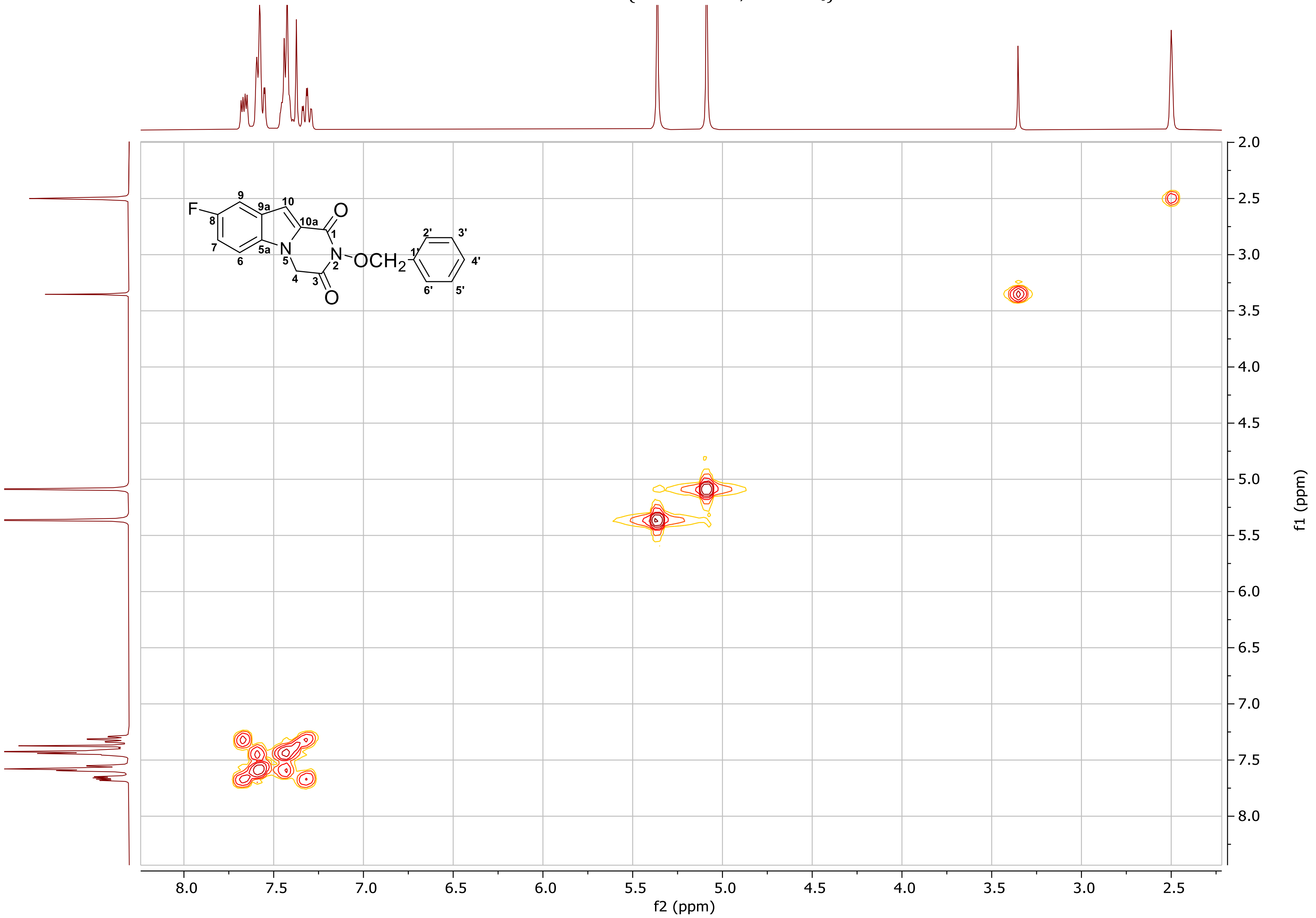

HSQC NMR of **11** (400.13 MHz, DMSO-d_6)

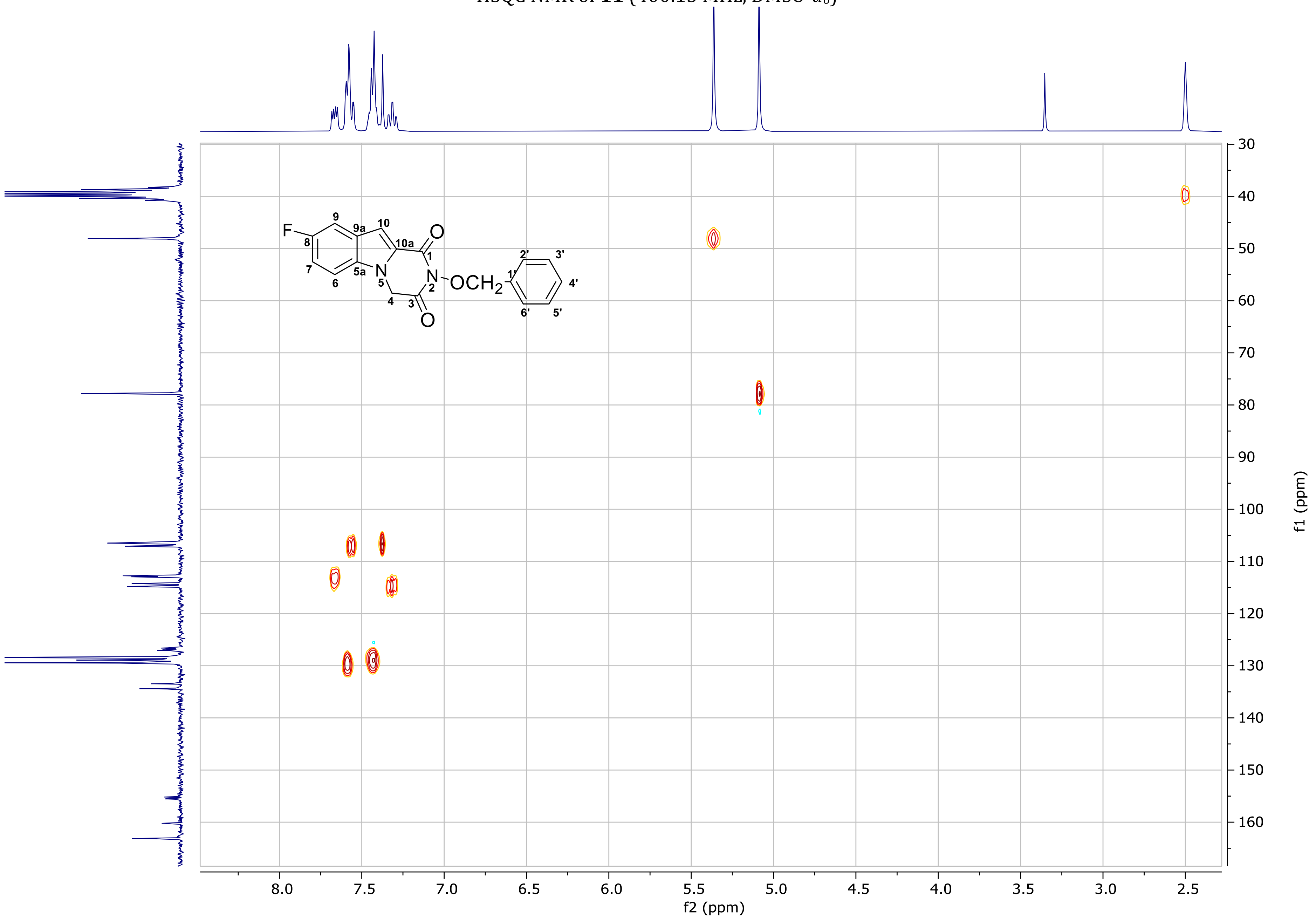

HMBC NMR of **11** (400.13 MHz, DMSO-d_6)

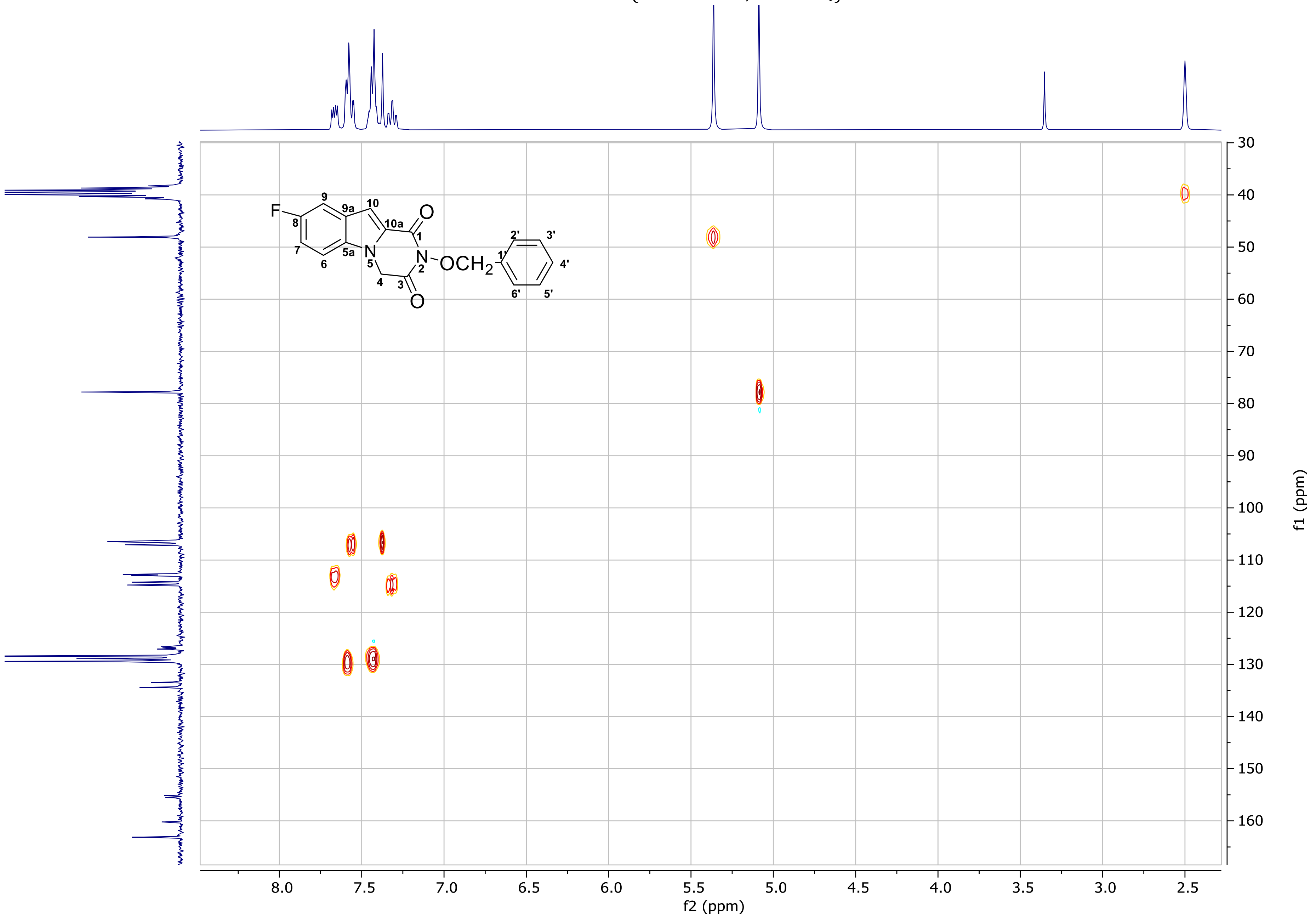

^{1}H NMR of **17** (400.13 MHz, DMSO-d_6)

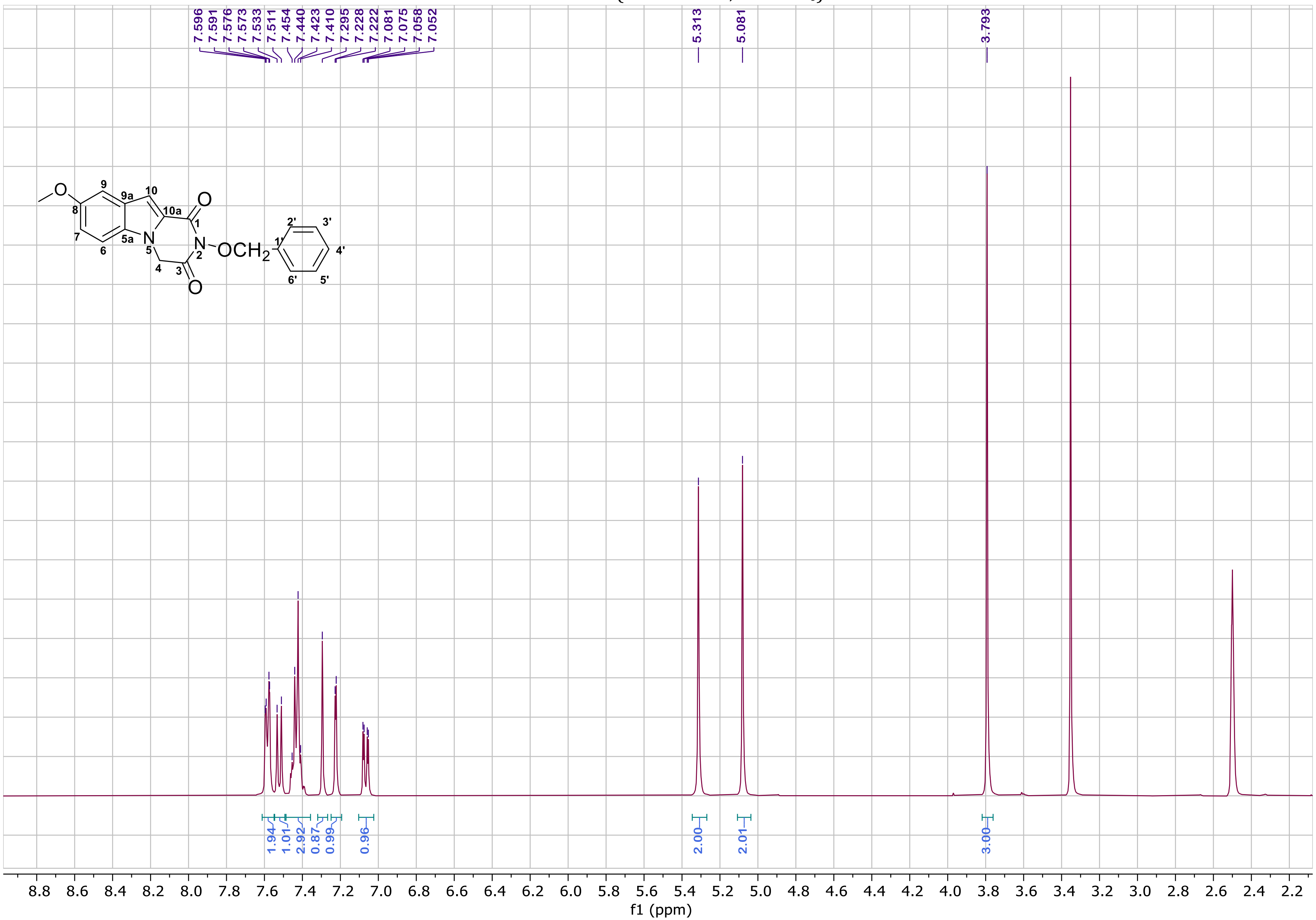

^{13}C NMR of **17** (50.32 MHz, DMSO-d_6)

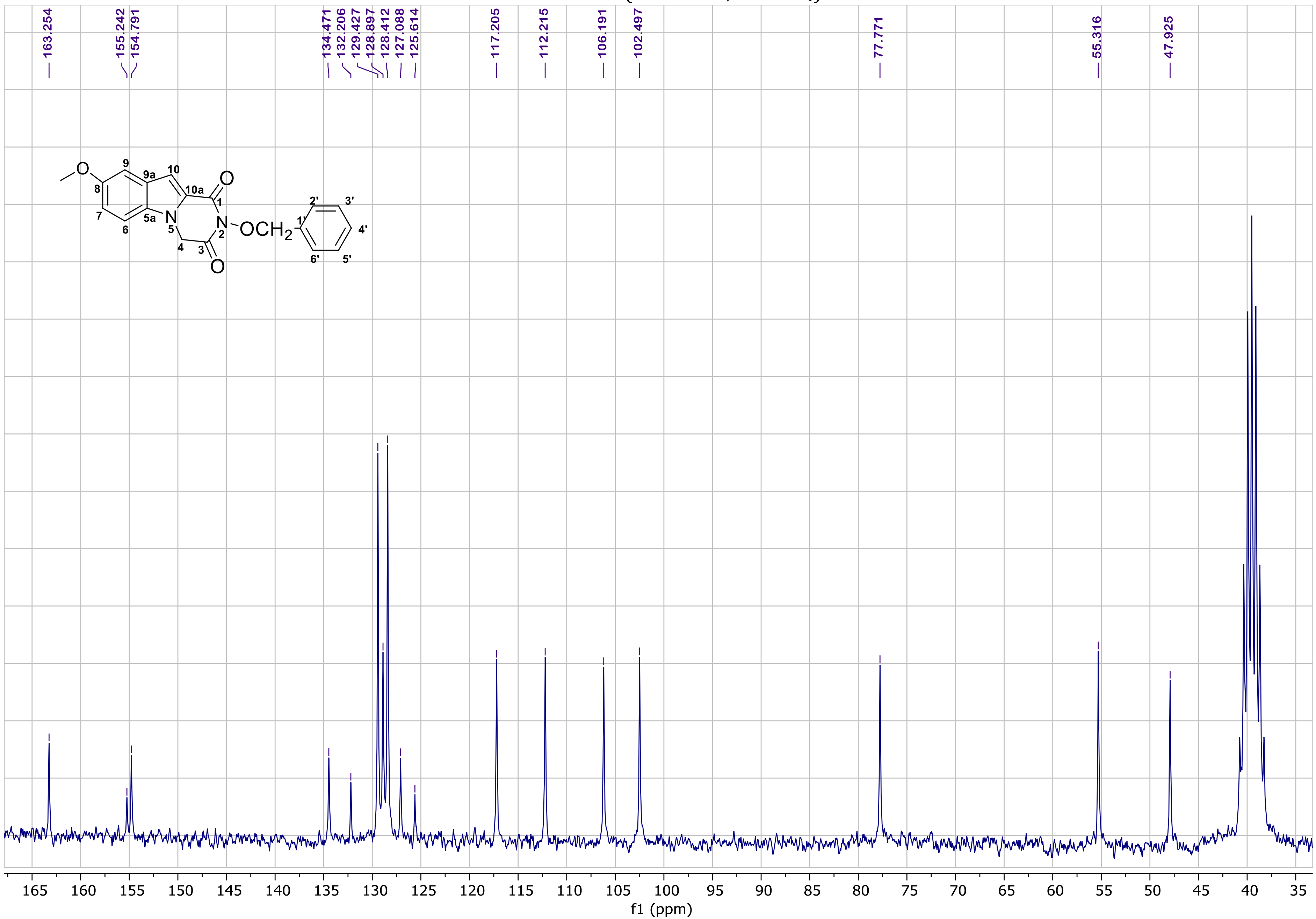

DEPT NMR of **17** (50.32 MHz, DMSO-d_6)

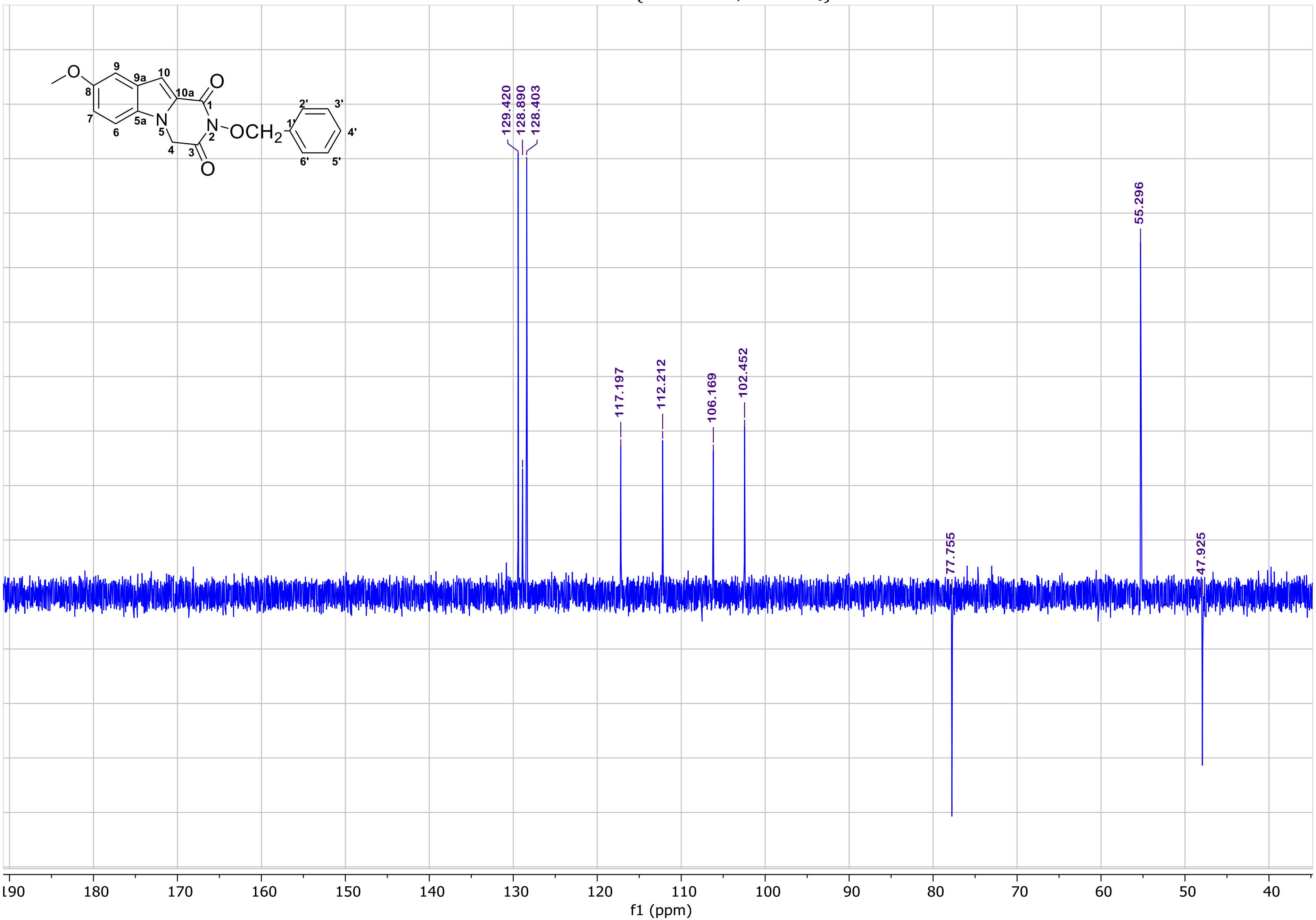

COSY NMR of **17** (400.13 MHz, DMSO-d_6)

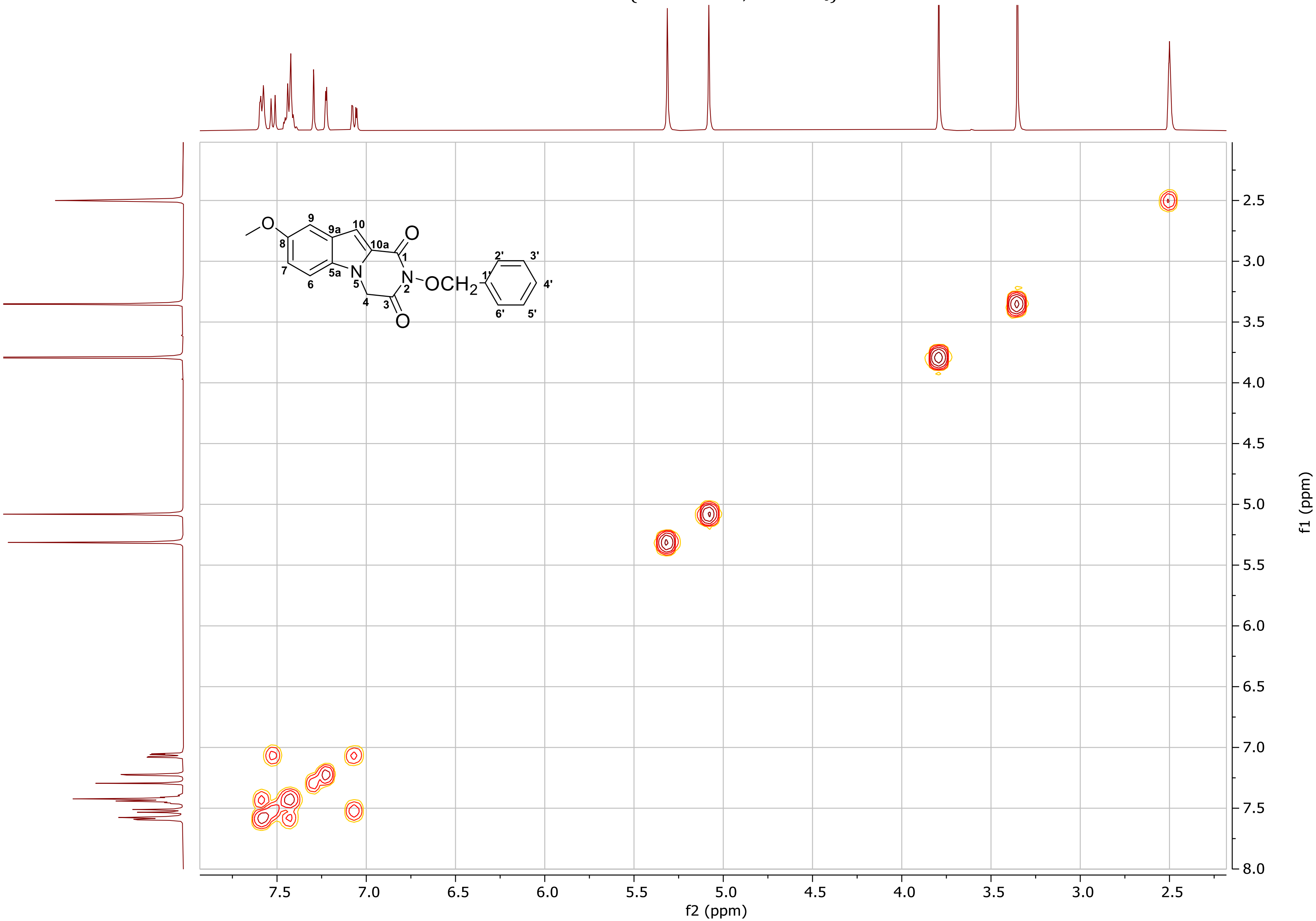

HSQC NMR of **17** (400.13 MHz, DMSO-d_6)

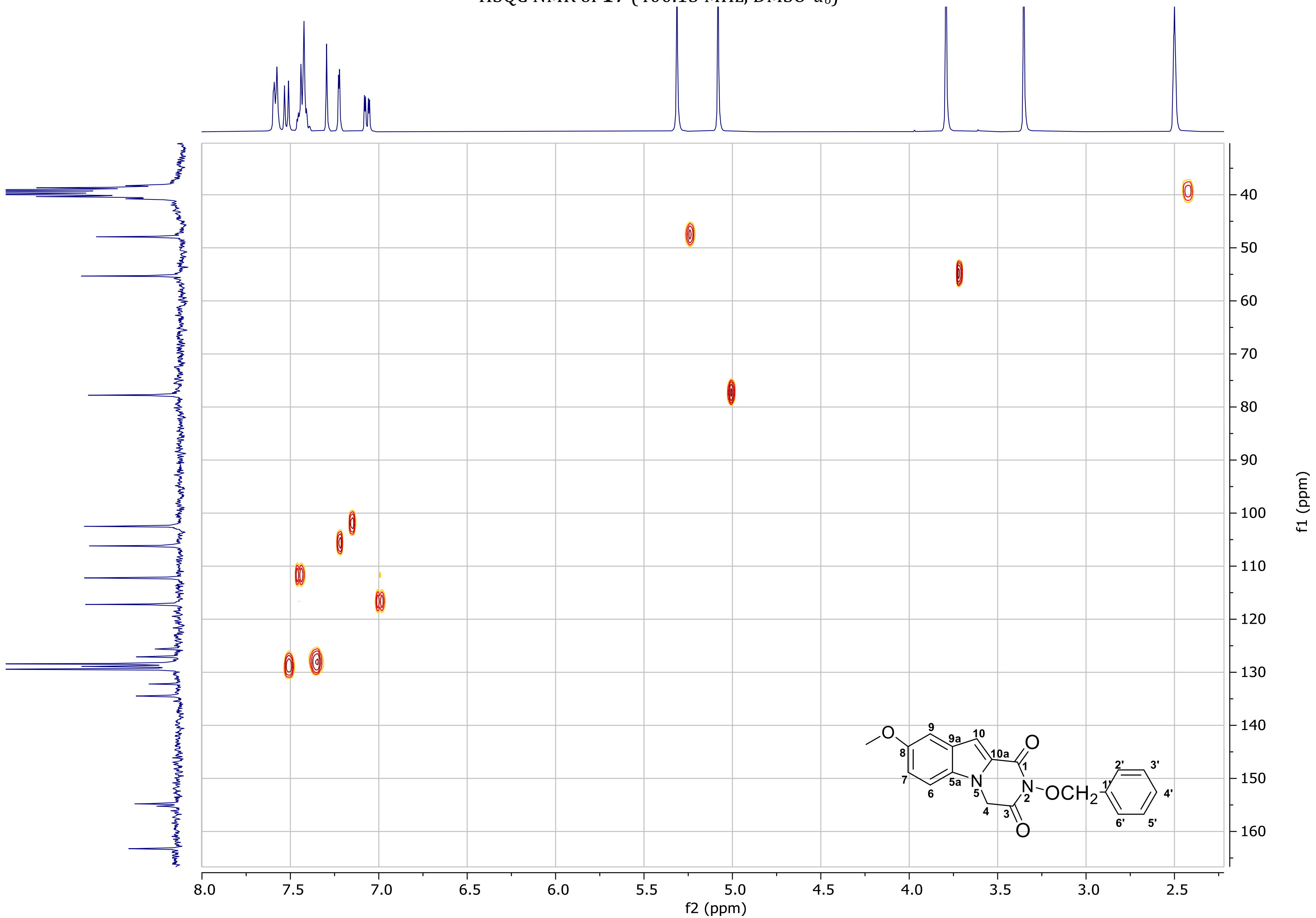

HMBC NMR of **17** (400.13 MHz, DMSO-d_6)

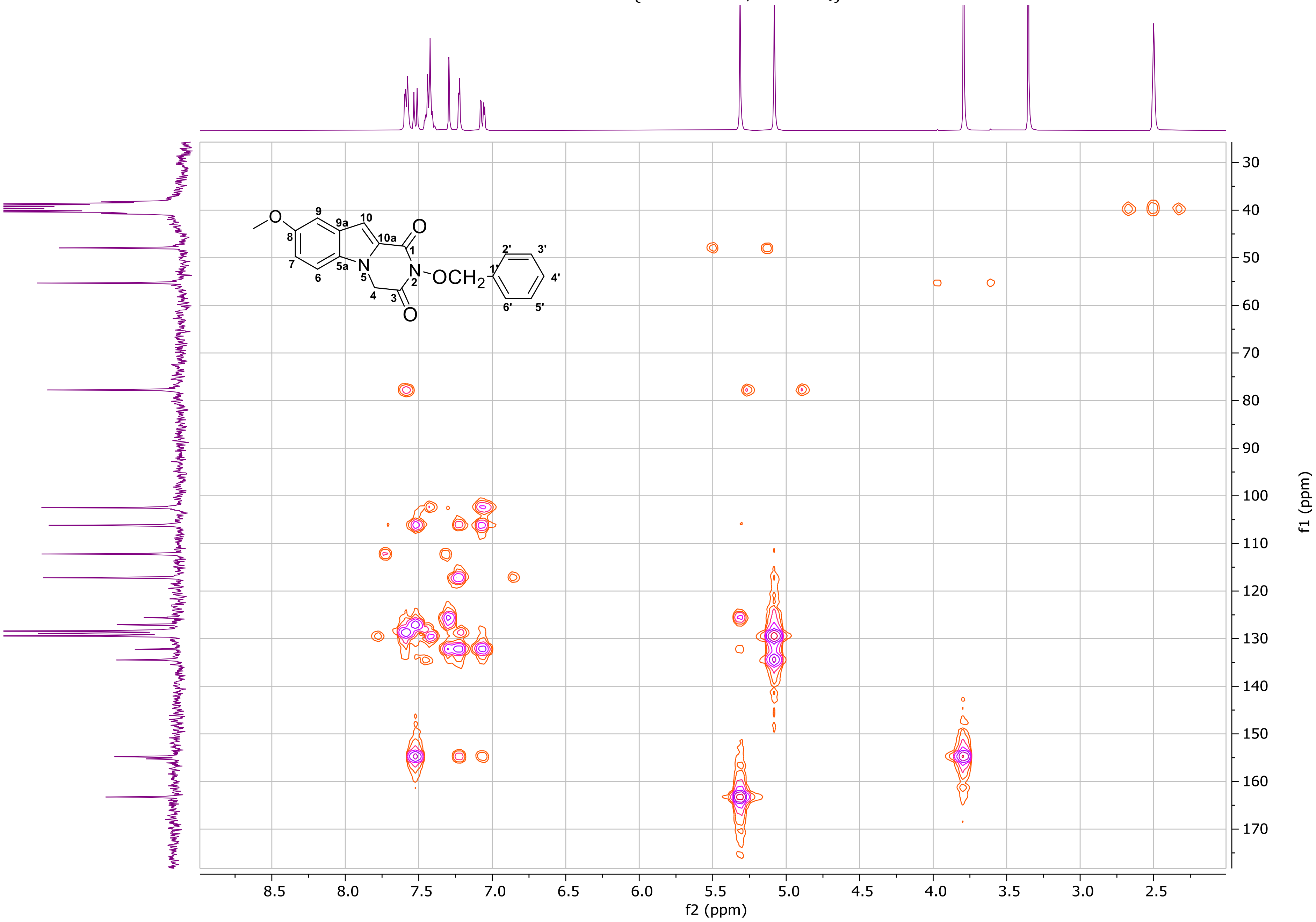

^{1}H NMR of **23** (400.13 MHz, DMSO-d_6)

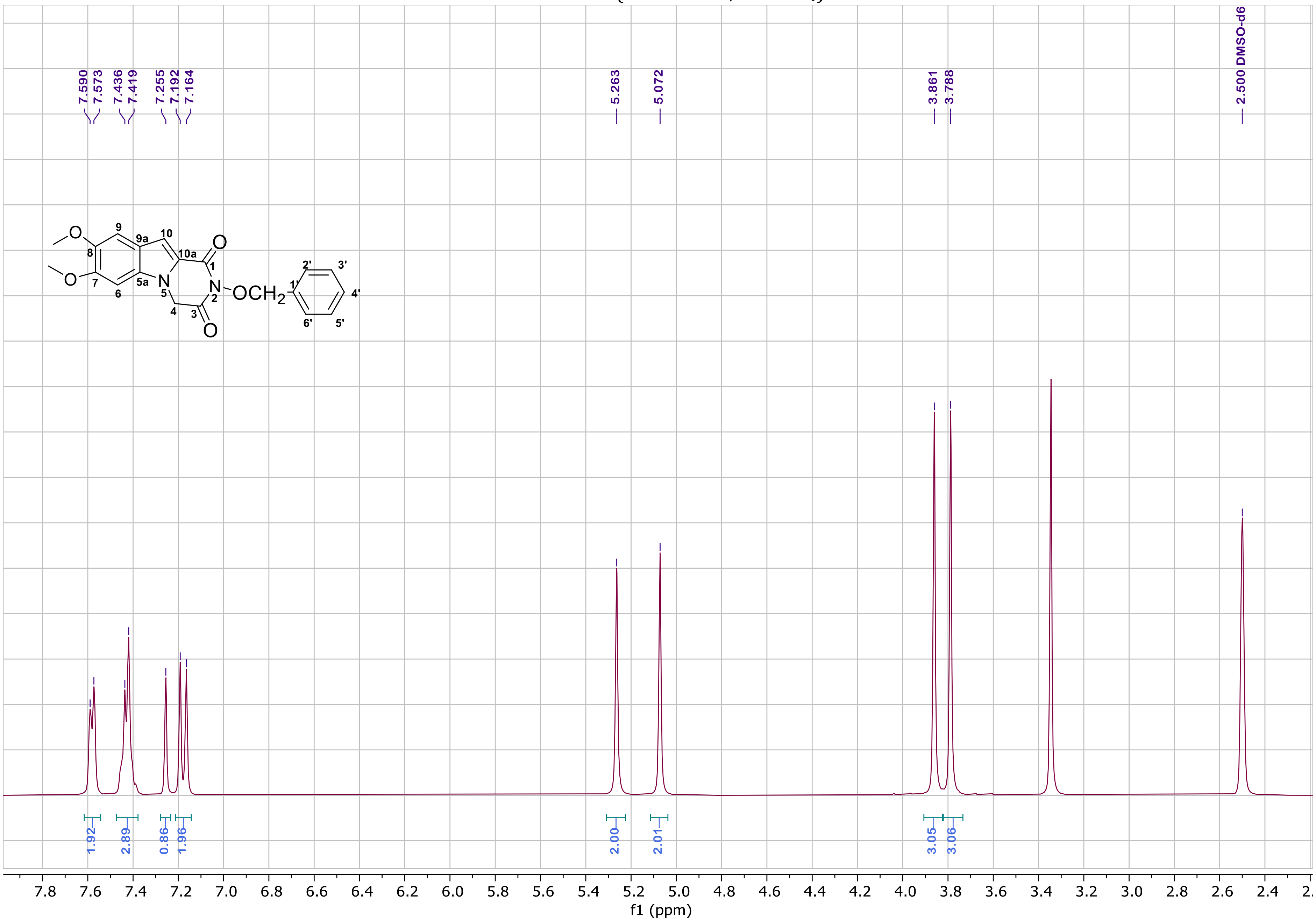

^{13}C NMR of **23** (50.32 MHz, DMSO-d_6)

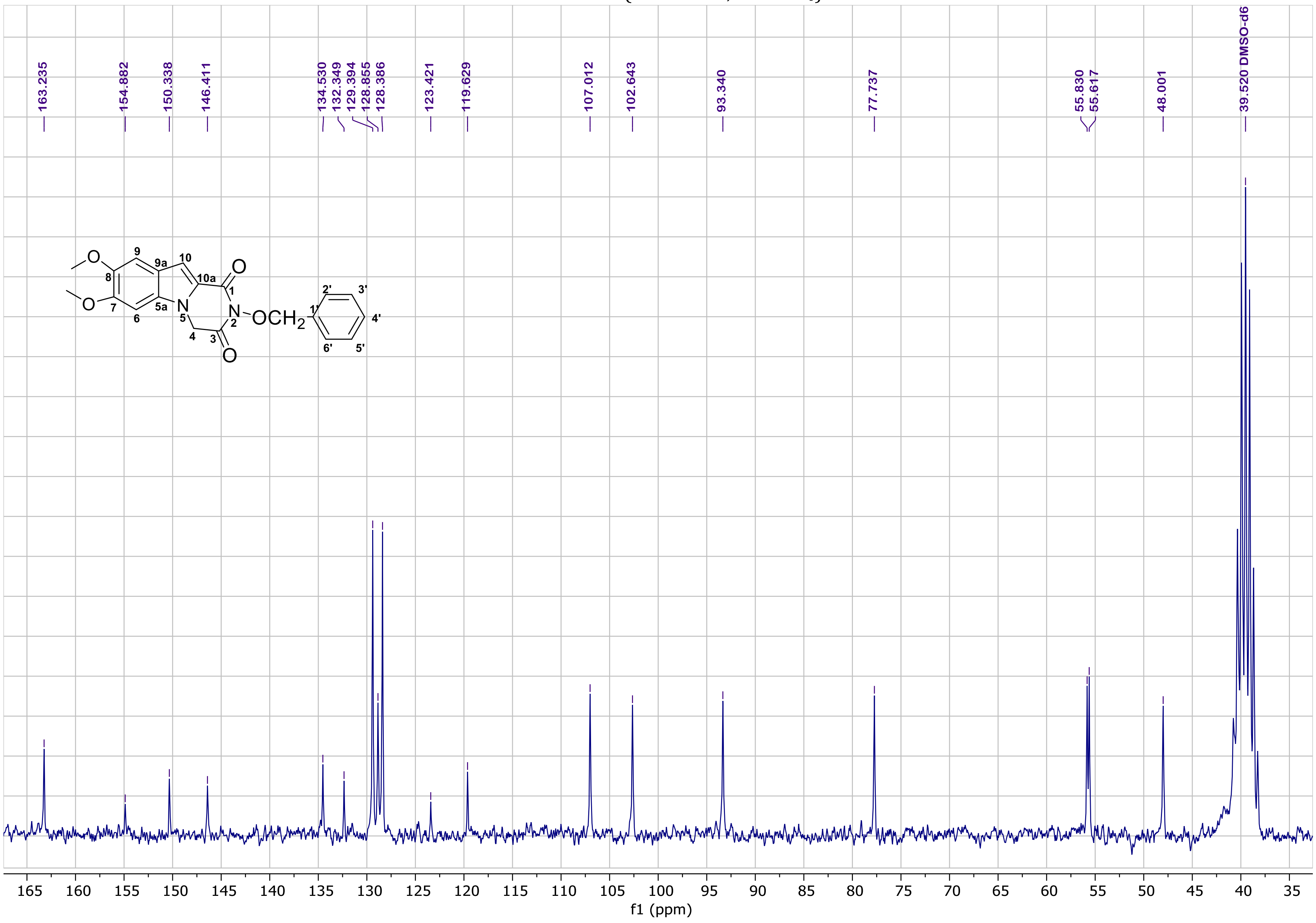

DEPT NMR of **23** (50.32 MHz, DMSO-d_6)

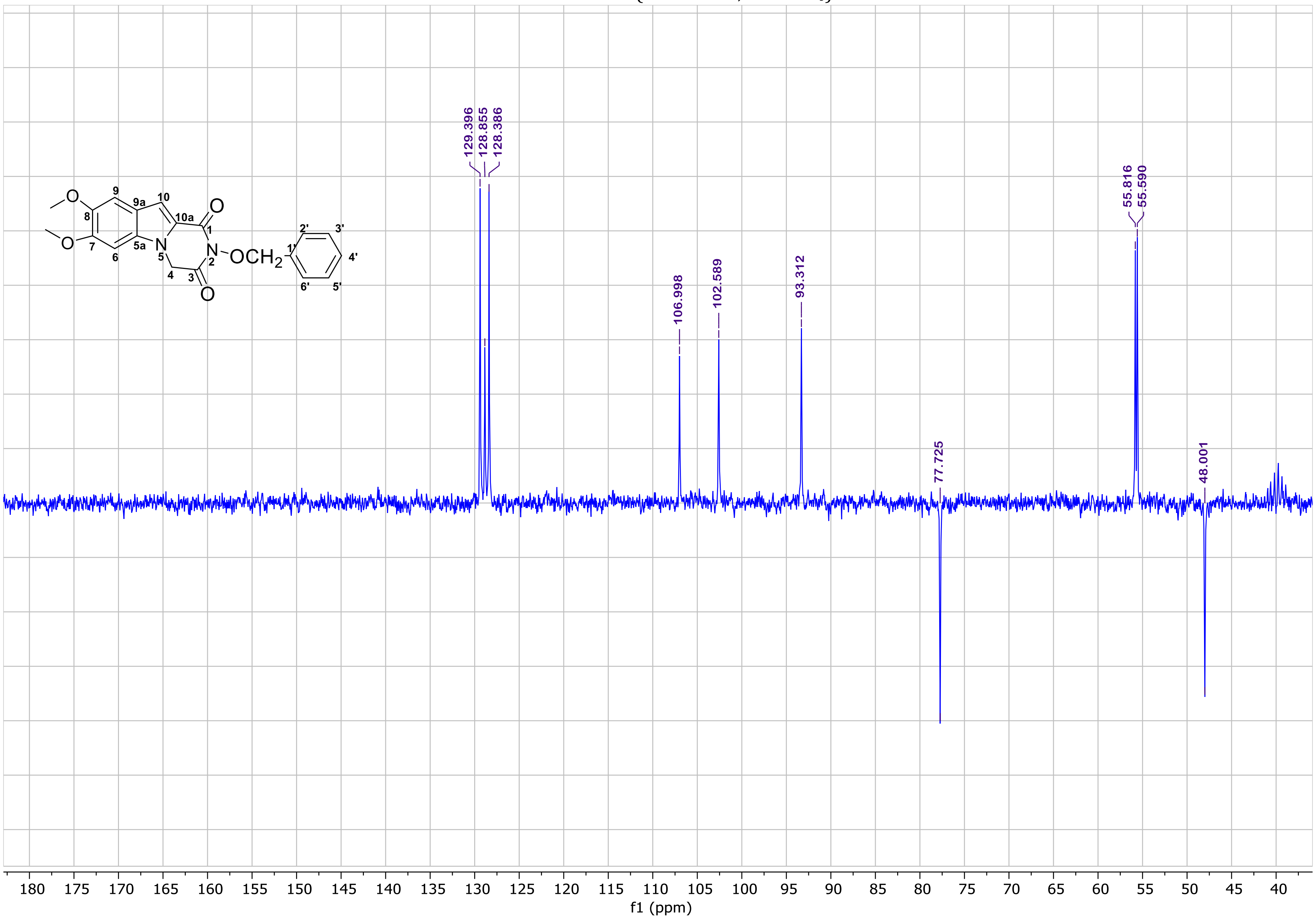

COSY NMR of **23** (400.13 MHz, DMSO-d_6)

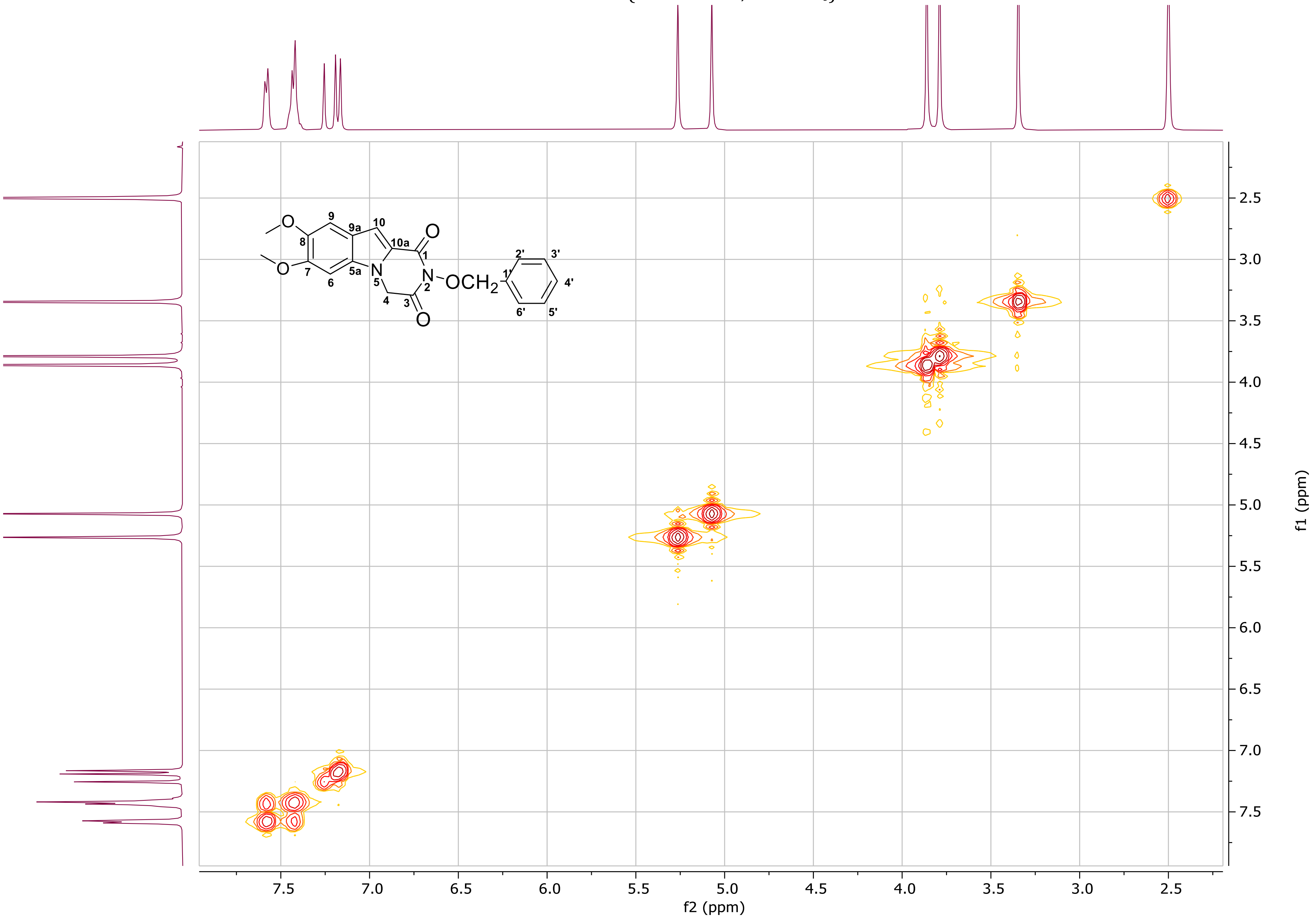

HSQC NMR of **23** (400.13 MHz, DMSO-d_6)

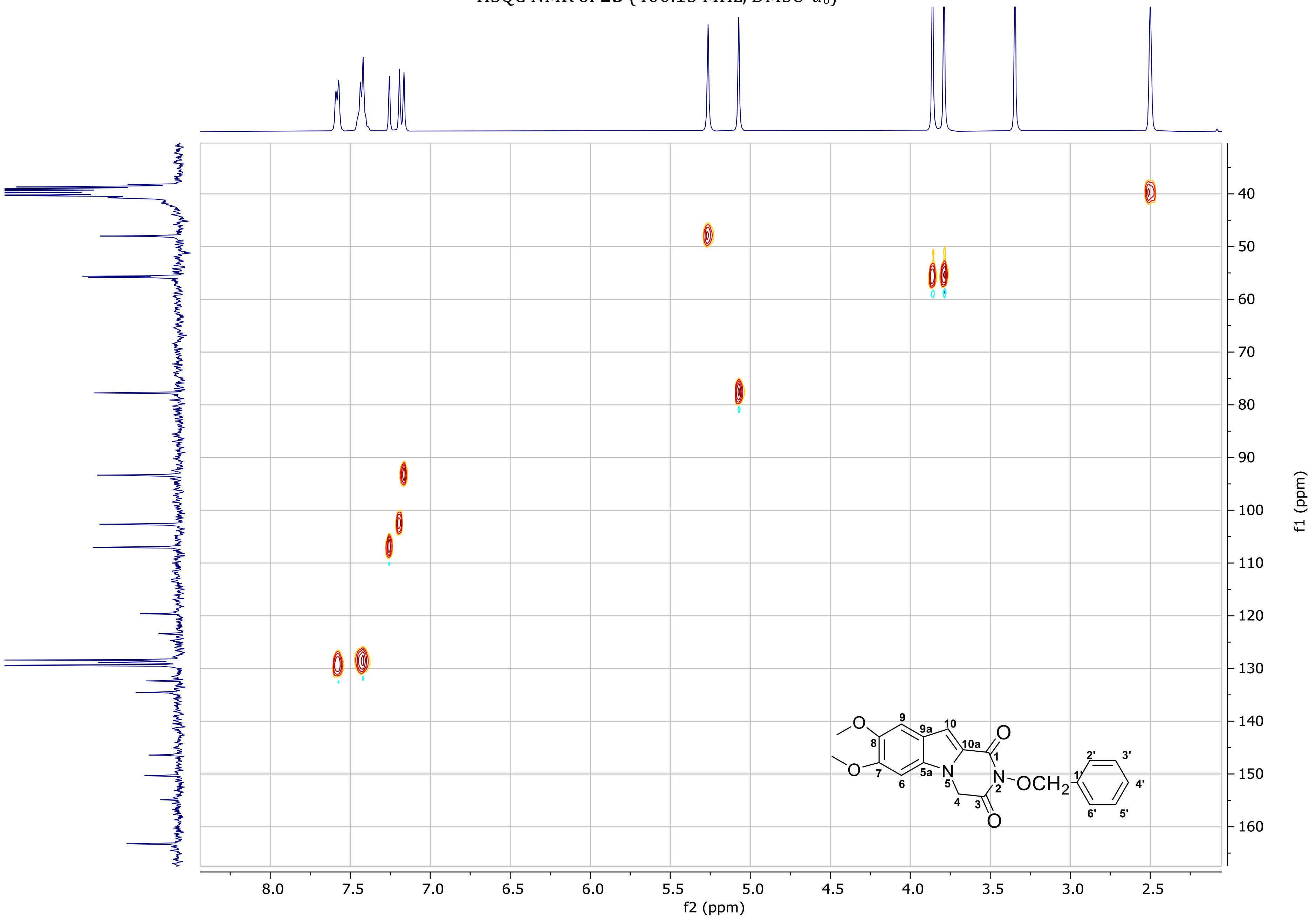

HMBC NMR of **23** (400.13 MHz, DMSO-d_6)

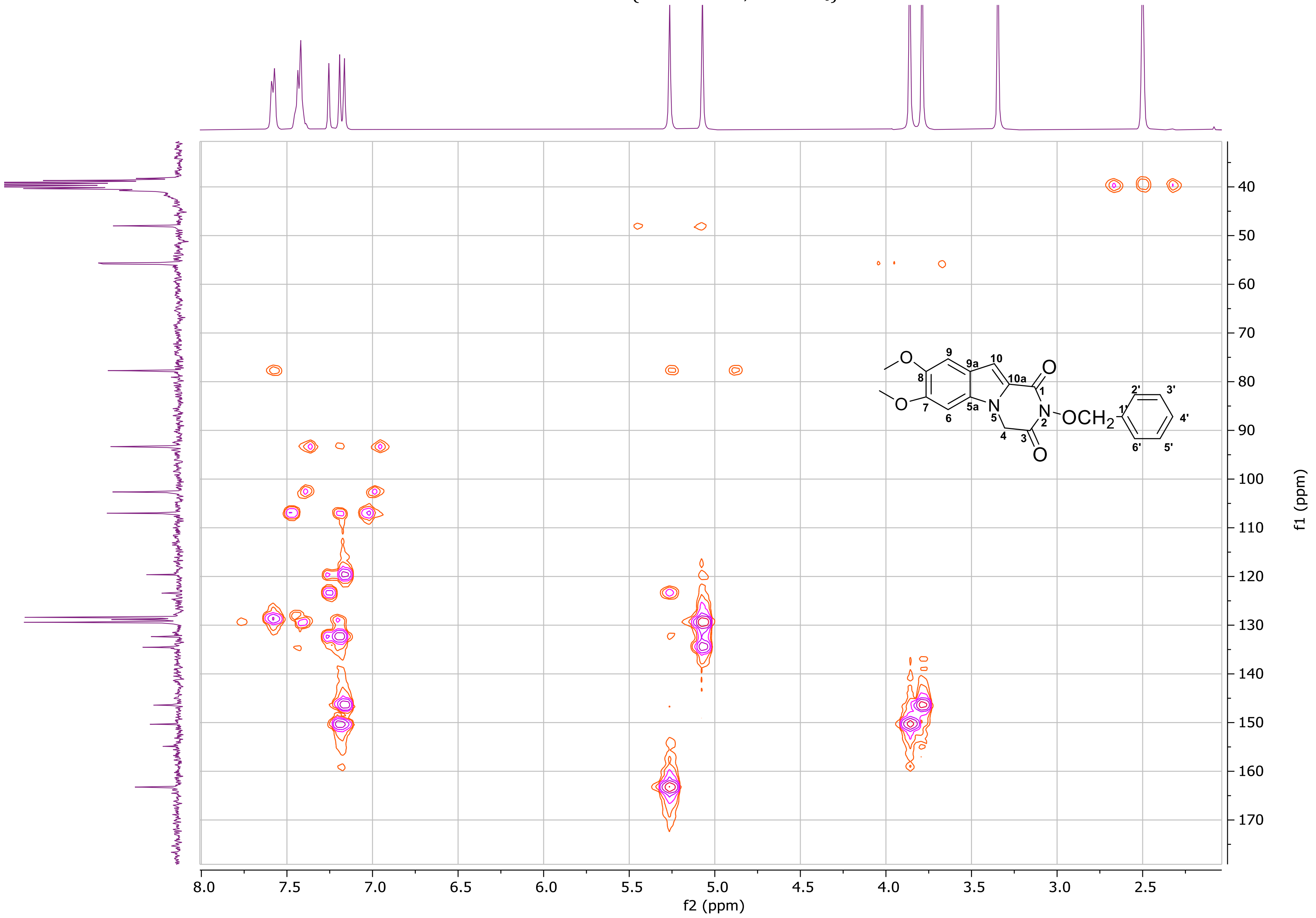

^{1}H NMR of **23a** (400.13 MHz, $CDCl_3$)

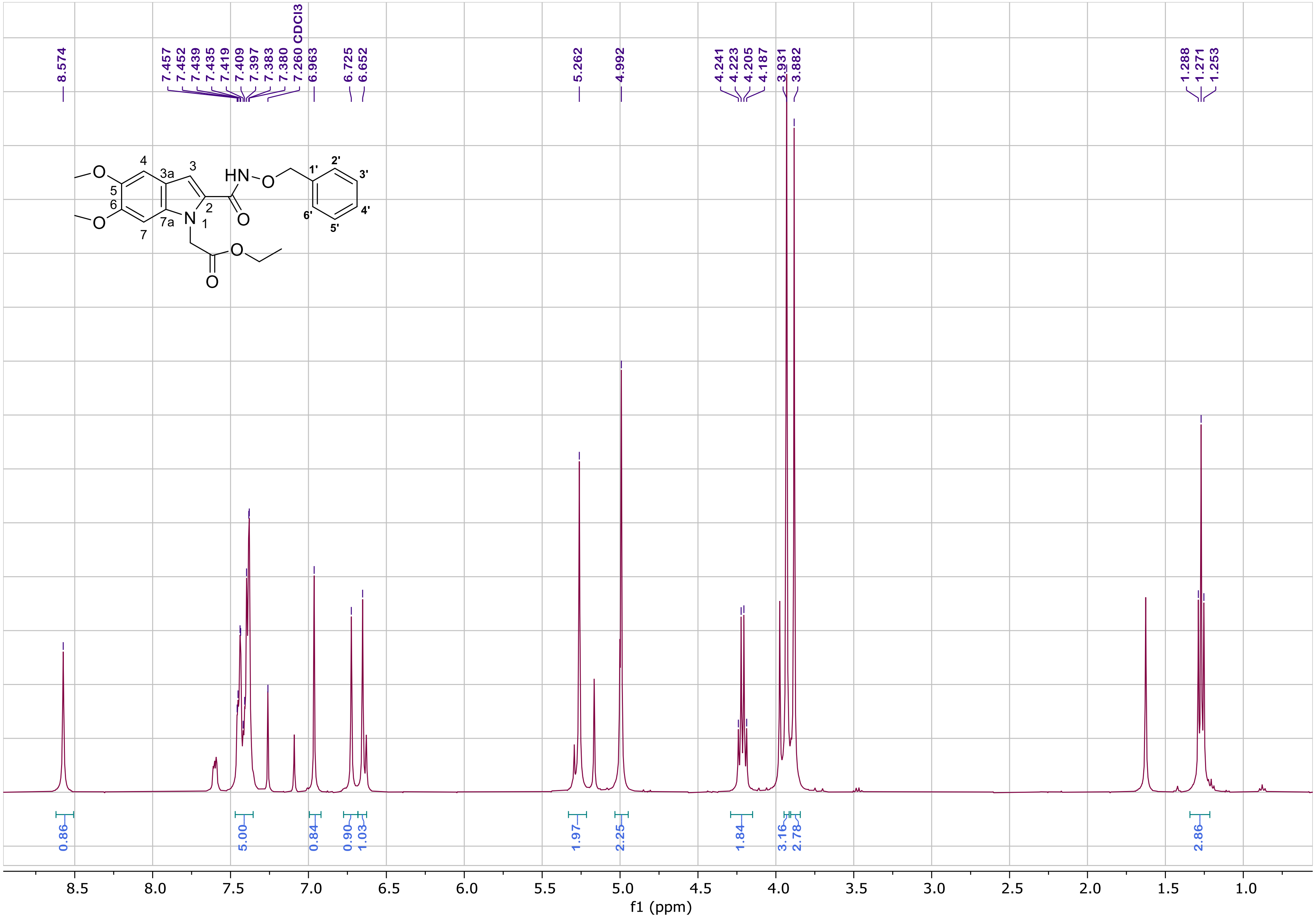

^{13}C NMR of **23a** (50.32 MHz, $CDCl_3$)

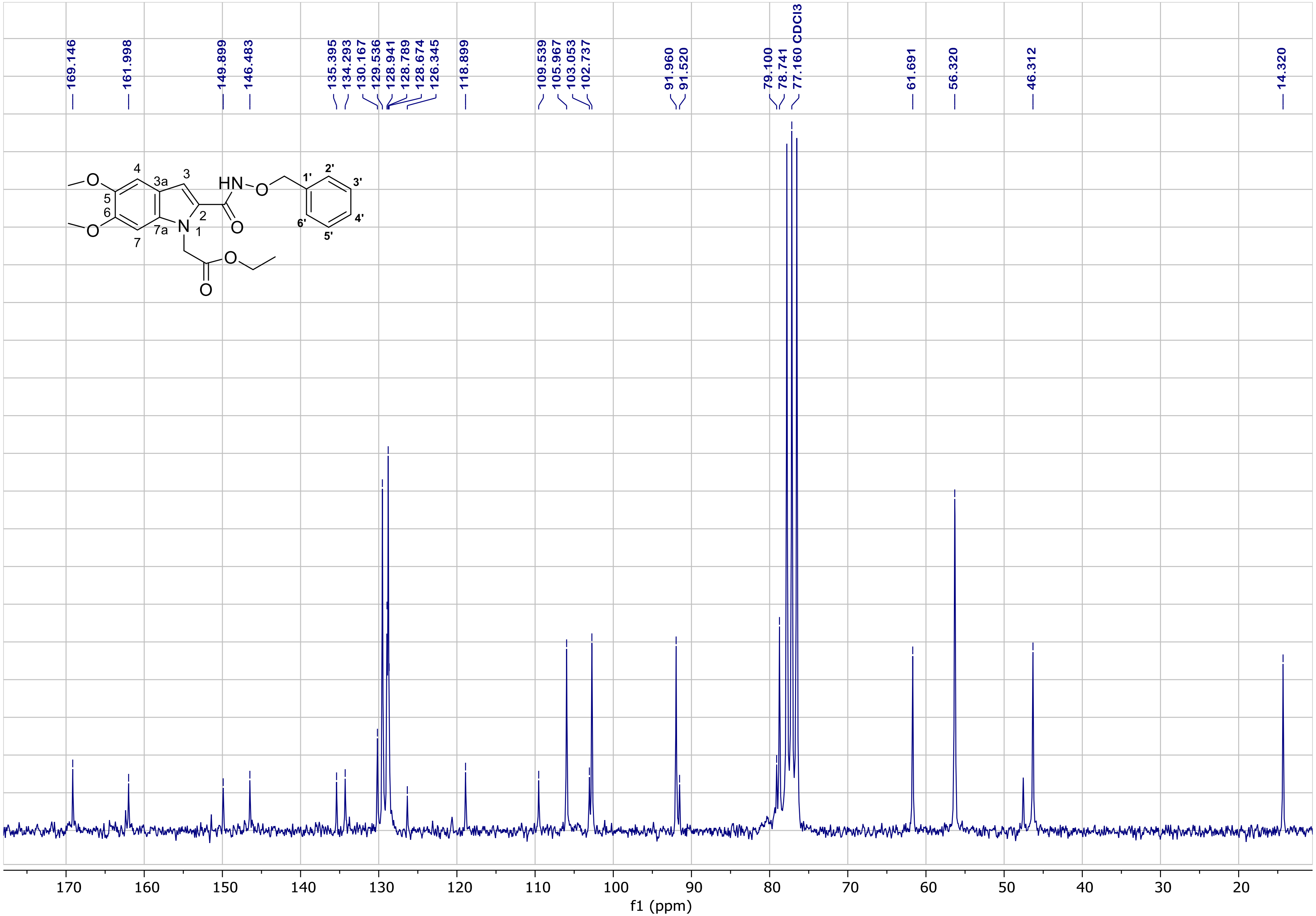

DEPT NMR of **23** (50.32 MHz, $CDCl_3$)

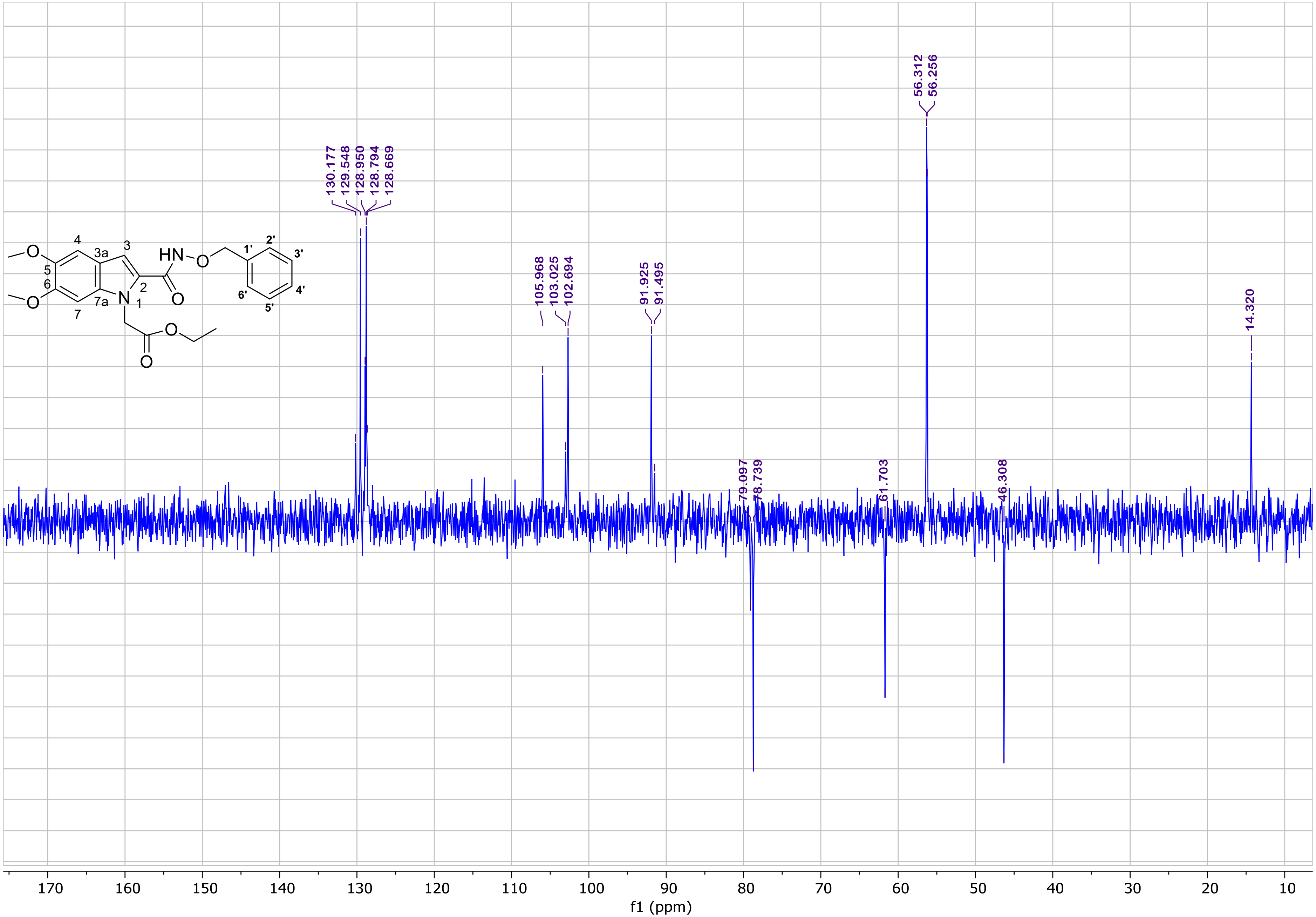

COSY NMR of **23** (400.13 MHz, $CDCl_3$)

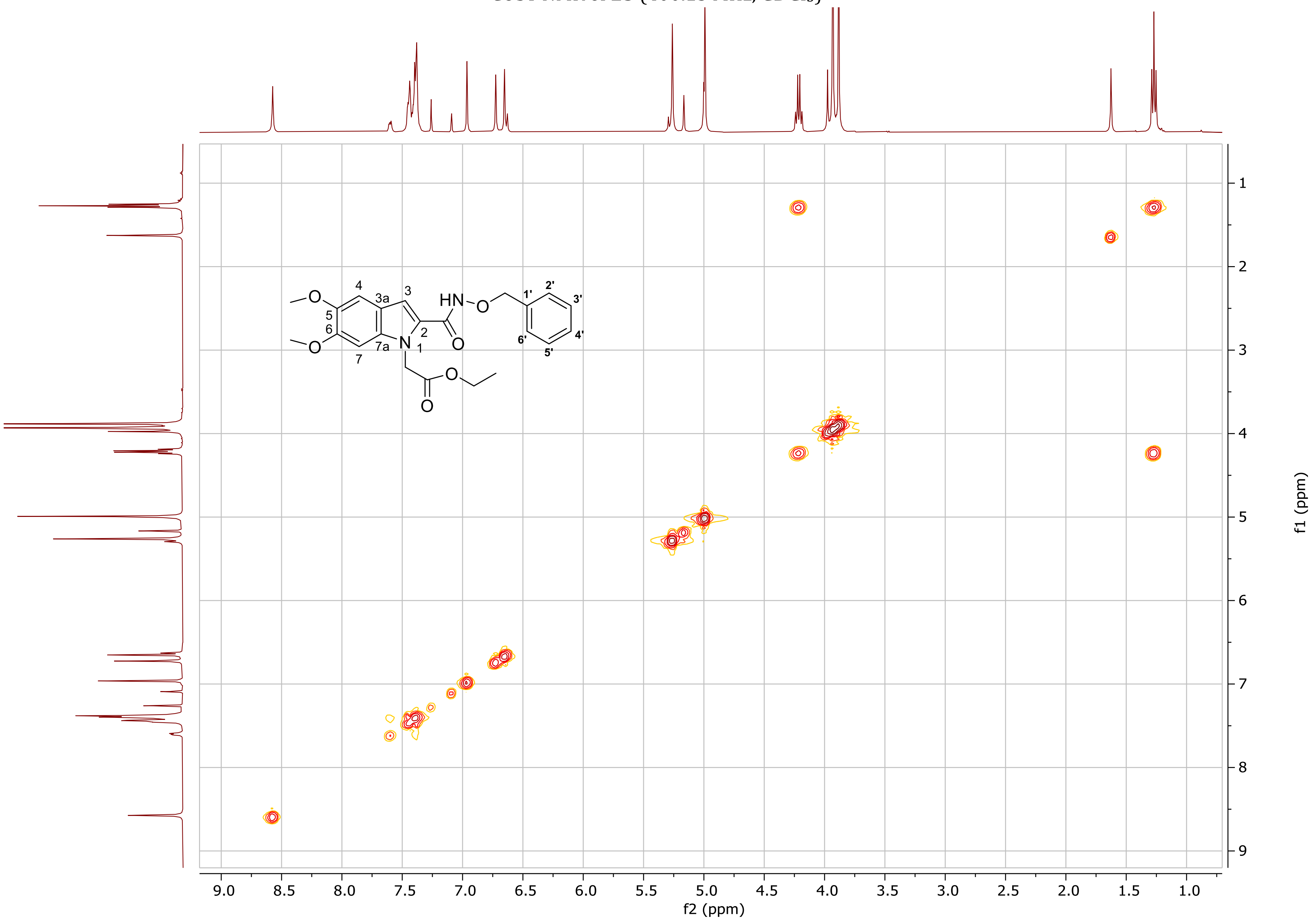

HSQC NMR of **23** (400.13 MHz, $CDCl_3$)

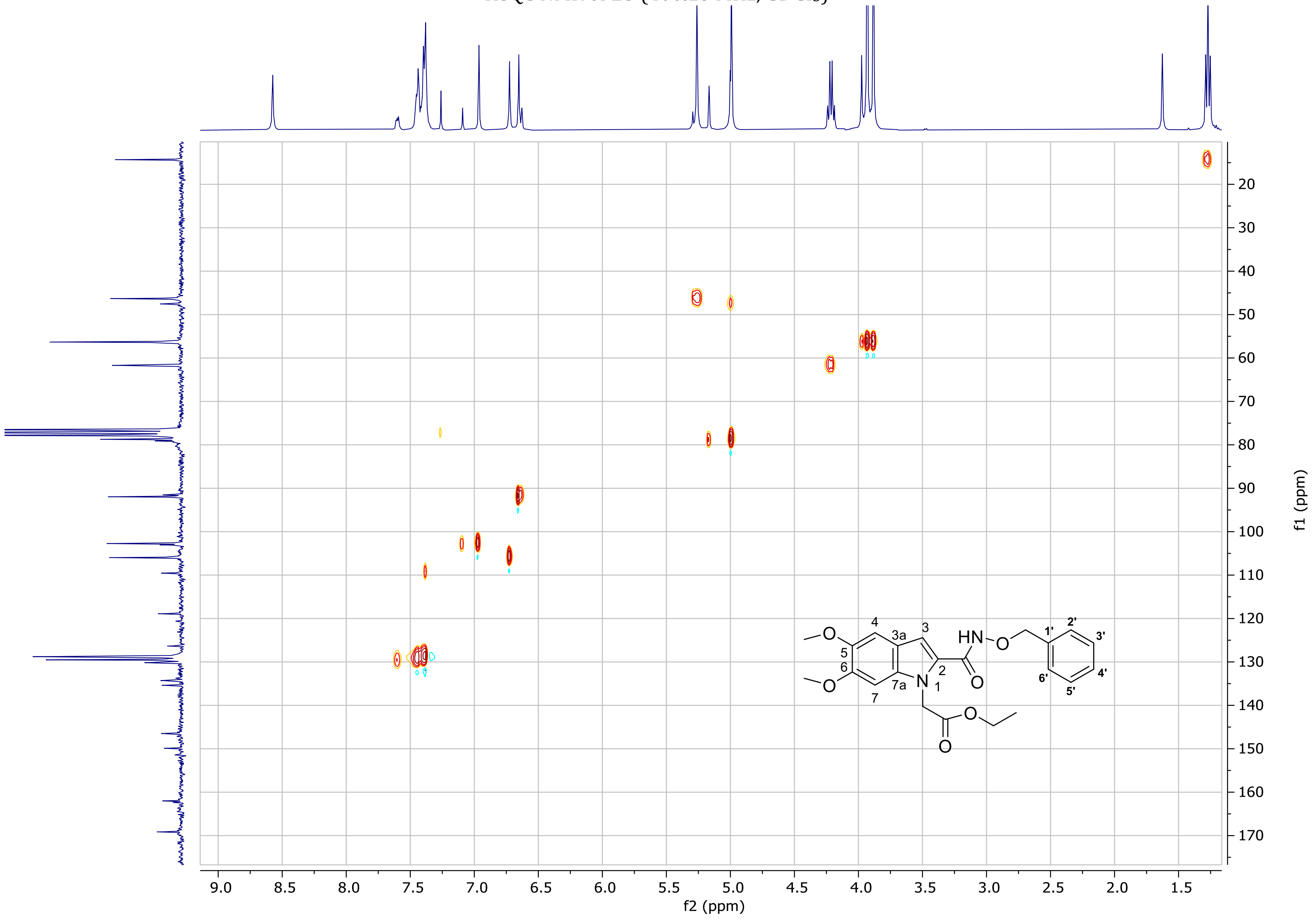

HMBC NMR of **23** (400.13 MHz, $CDCl_3$)

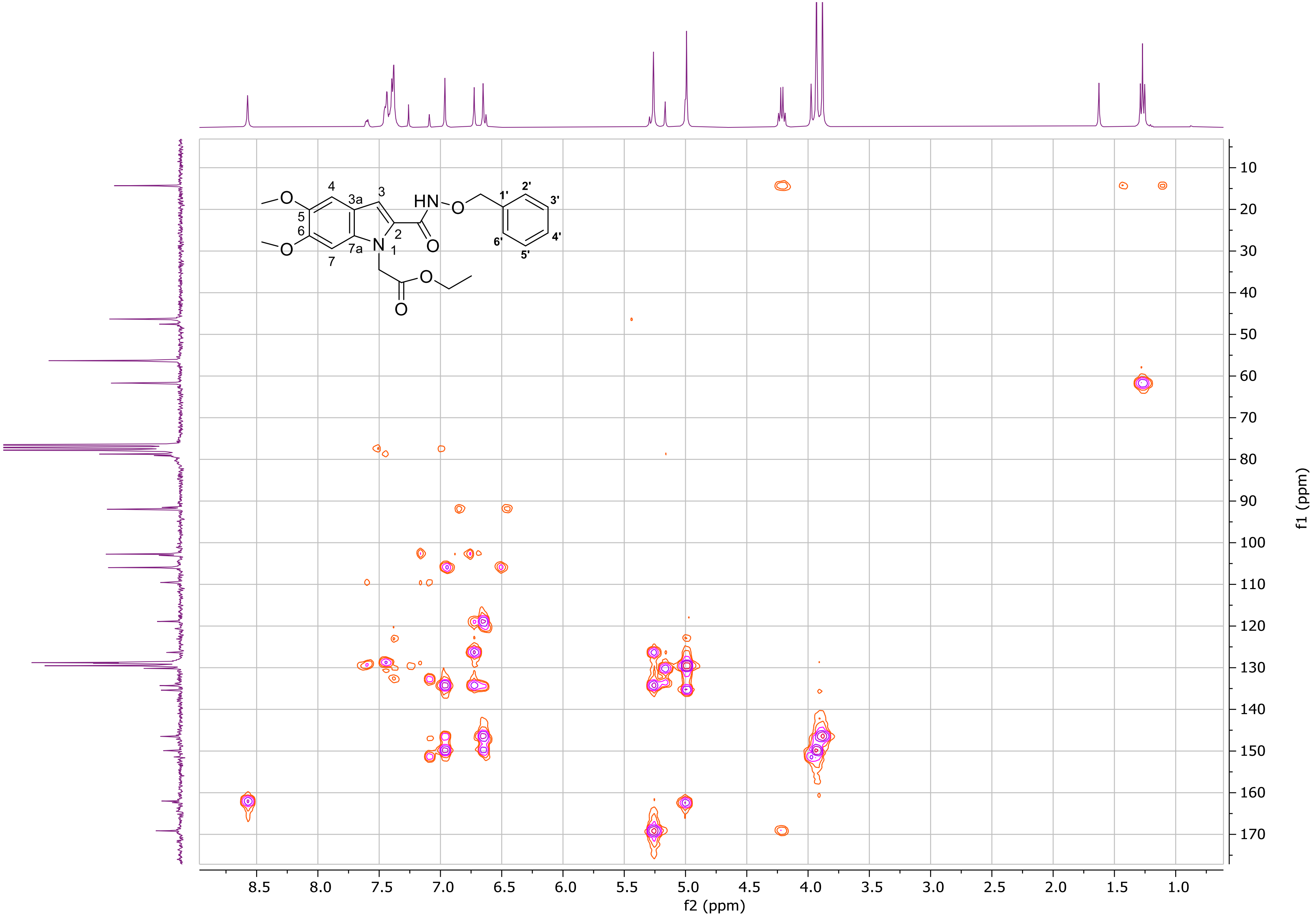

^{1}H NMR of **29** (400.13 MHz, DMSO-d_6)

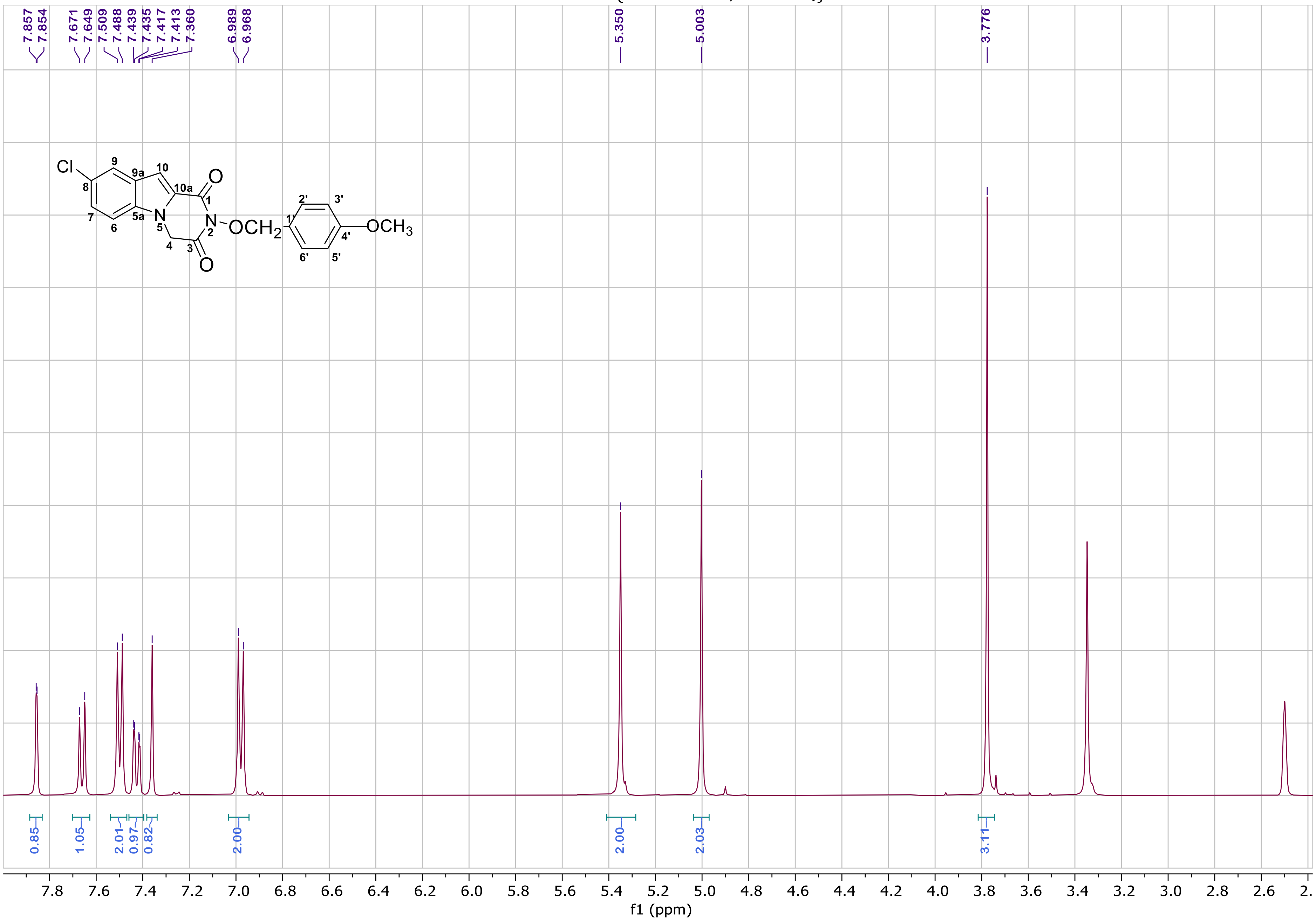

^{13}C NMR of **29** (50.32 MHz, DMSO-d_6)

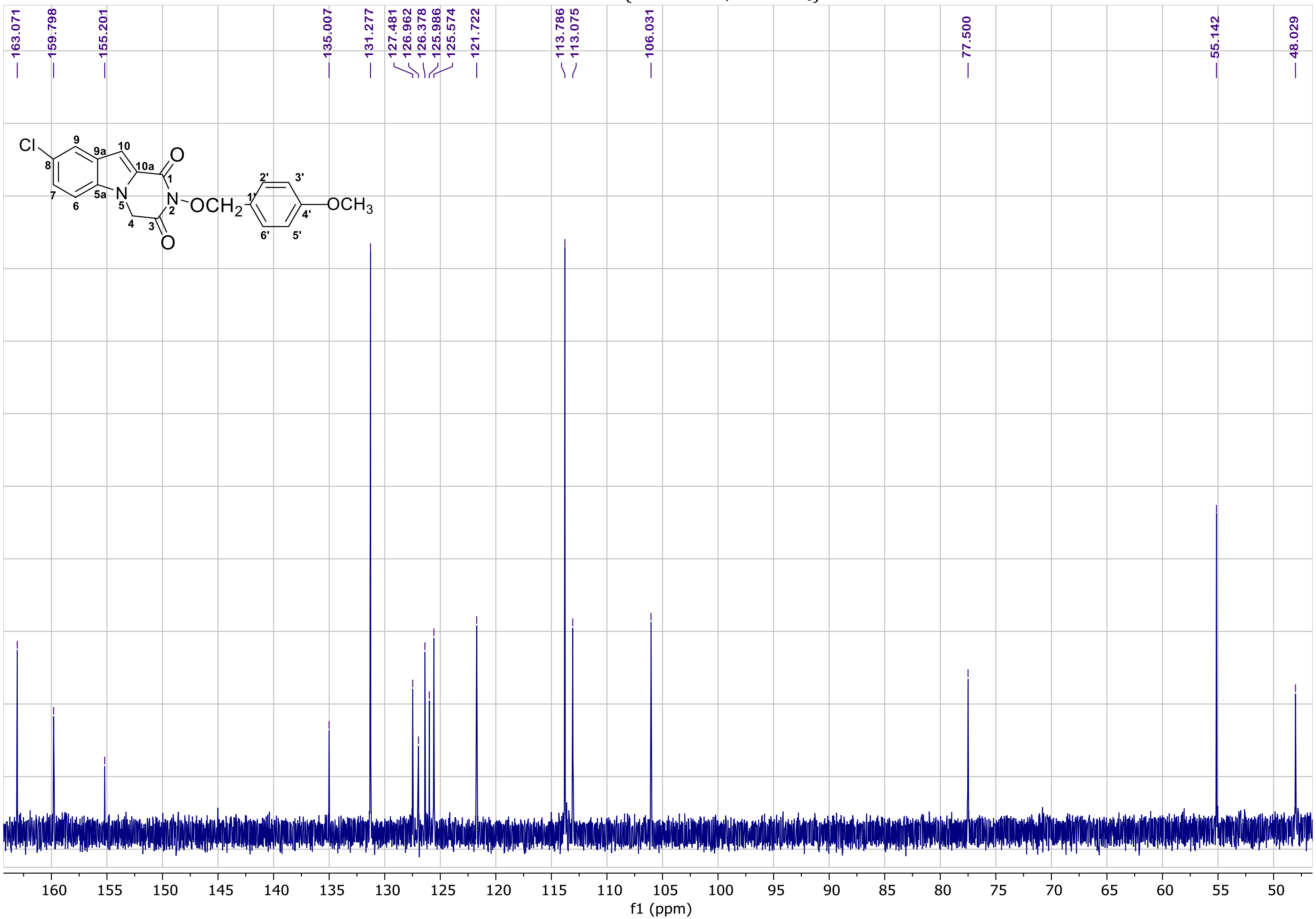

COSY NMR of **29** (400.13 MHz, DMSO-d_6)

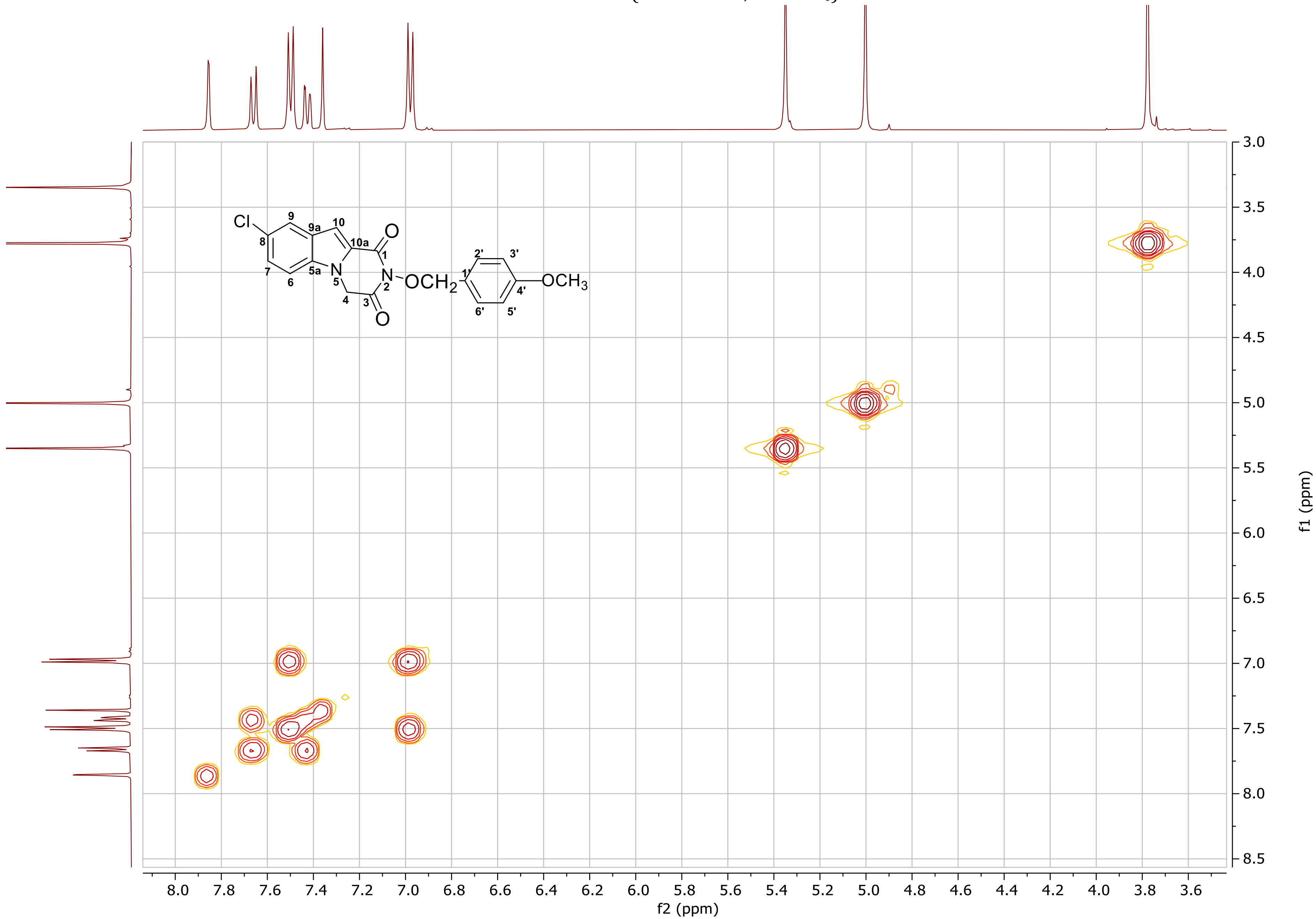

HSQC NMR of **29** (400.13 MHz, DMSO-d_6)

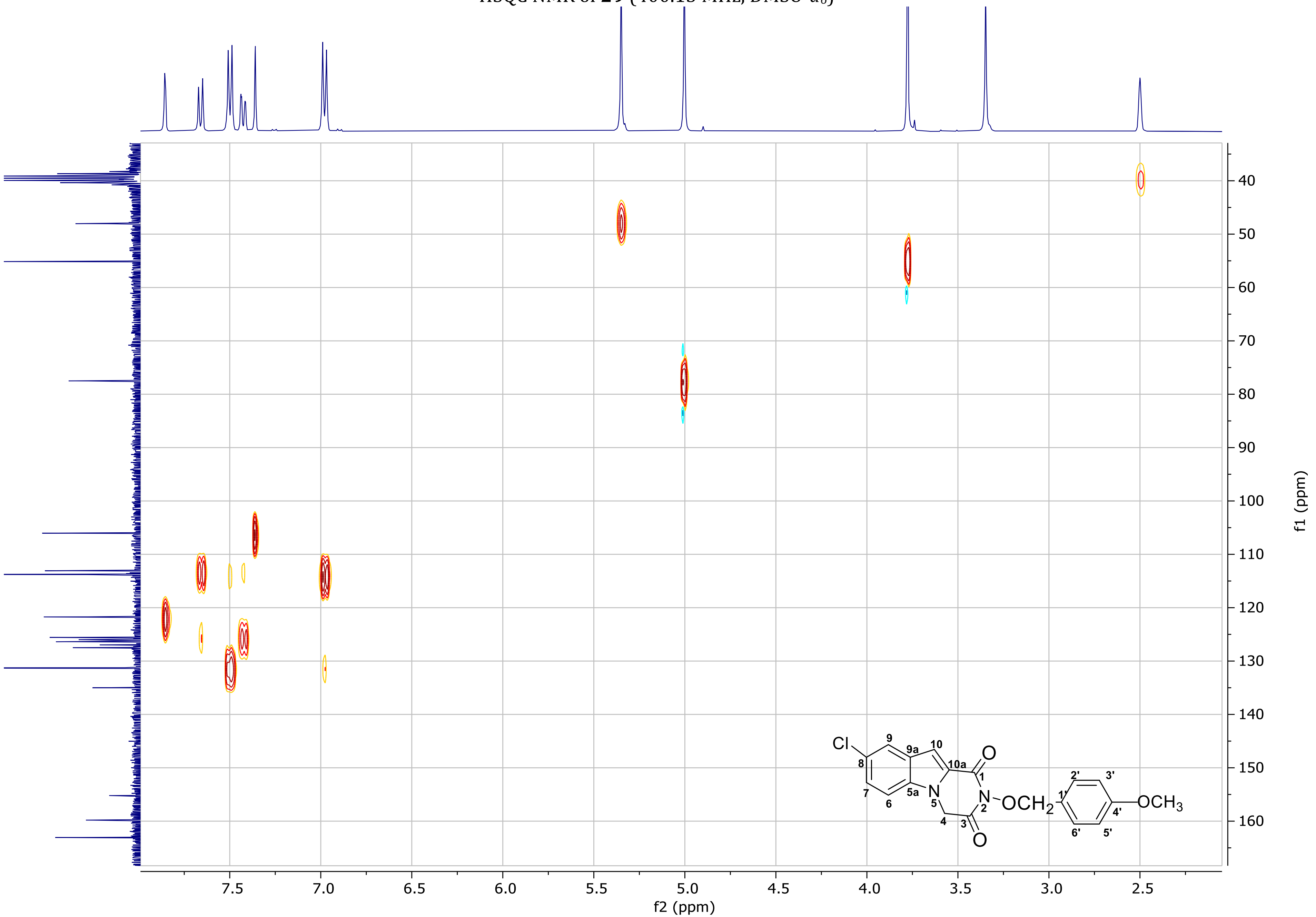

^{1}H NMR of **35** (400.13 MHz, $CDCl_3$)

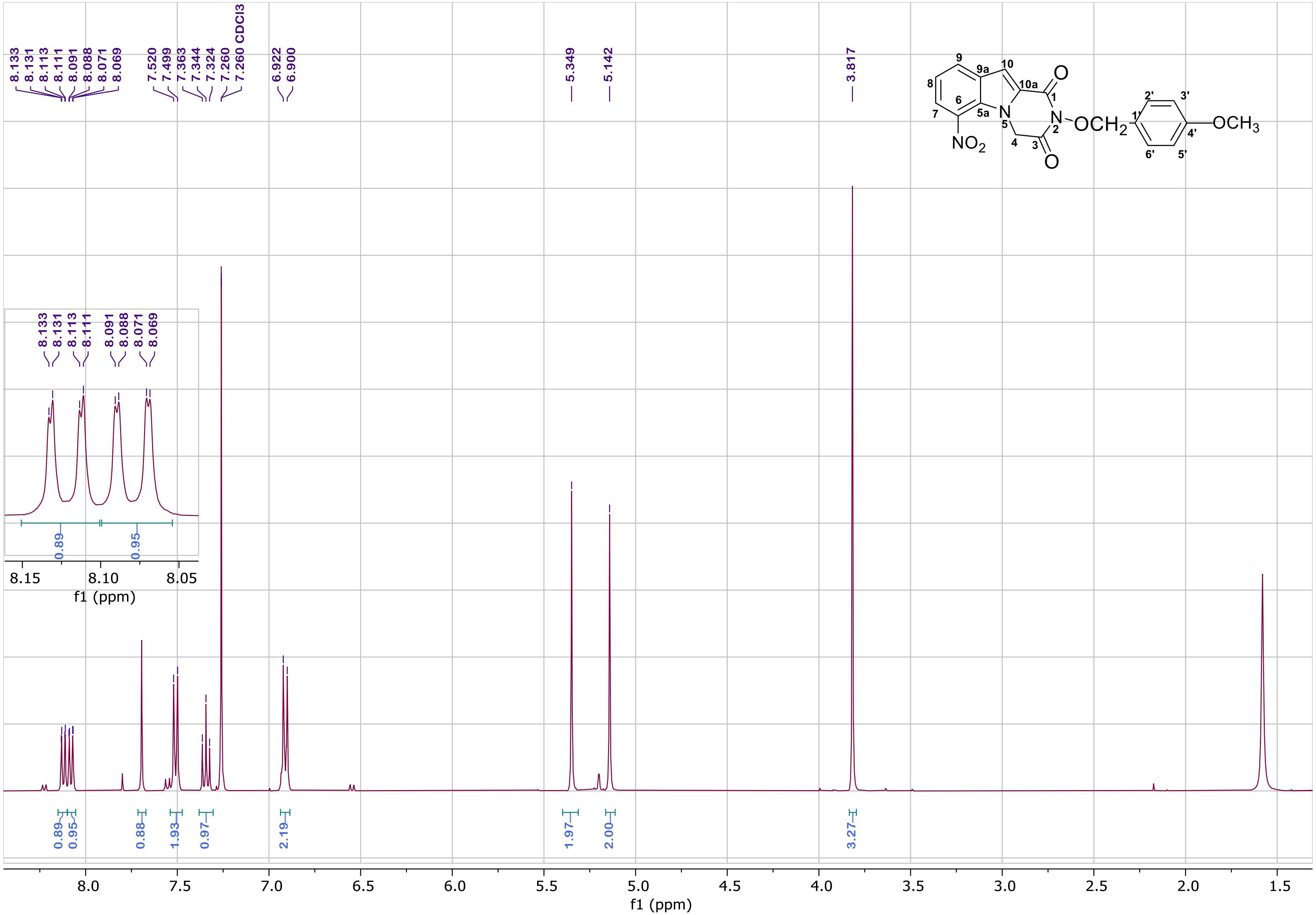

^{13}C NMR of **35** (50.32 MHz, $CDCl_3$)

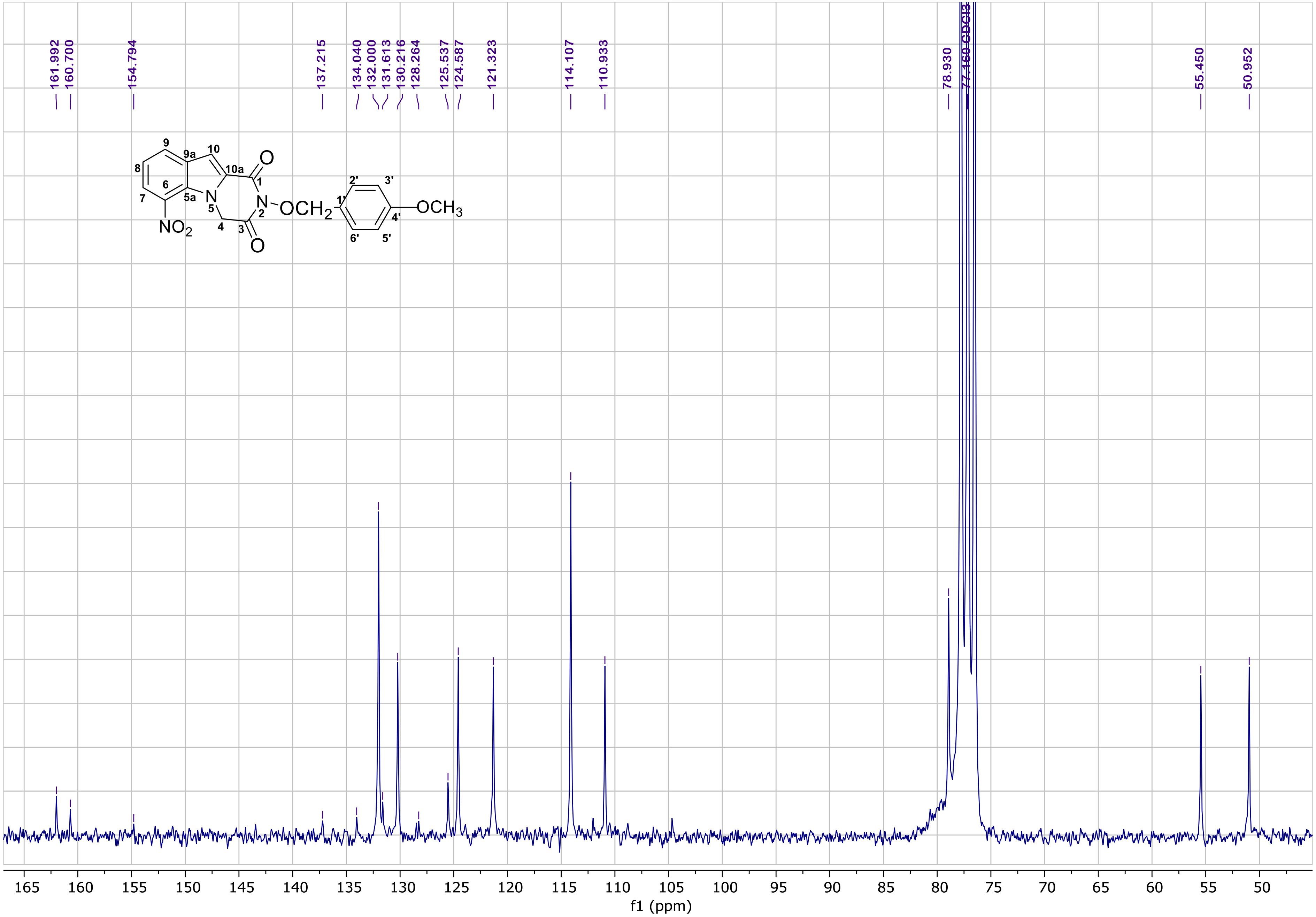

DEPT NMR of **35** (50.32 MHz, $CDCl_3$)

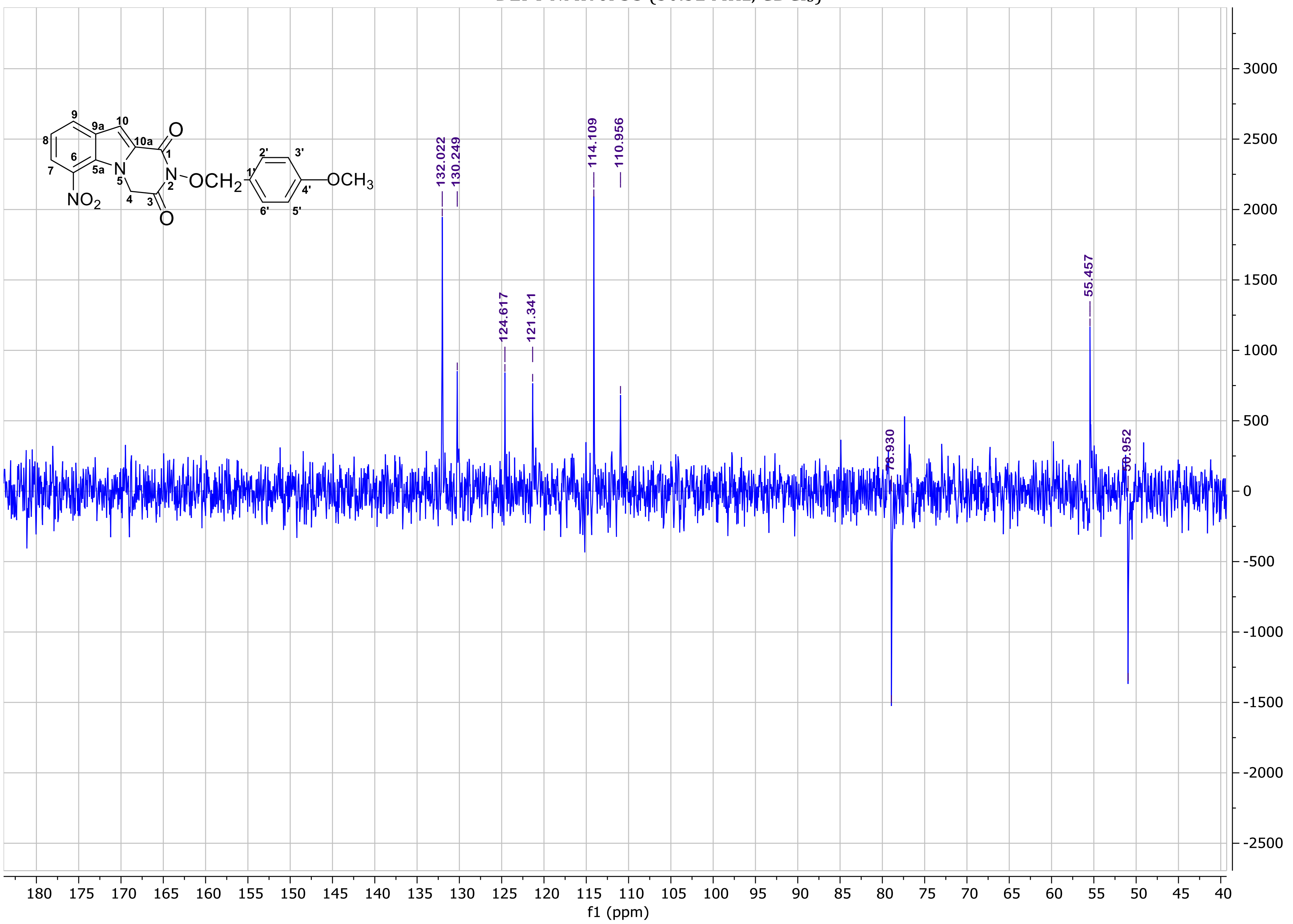

COSY NMR of **35** (400.13 MHz, $CDCl_3$)

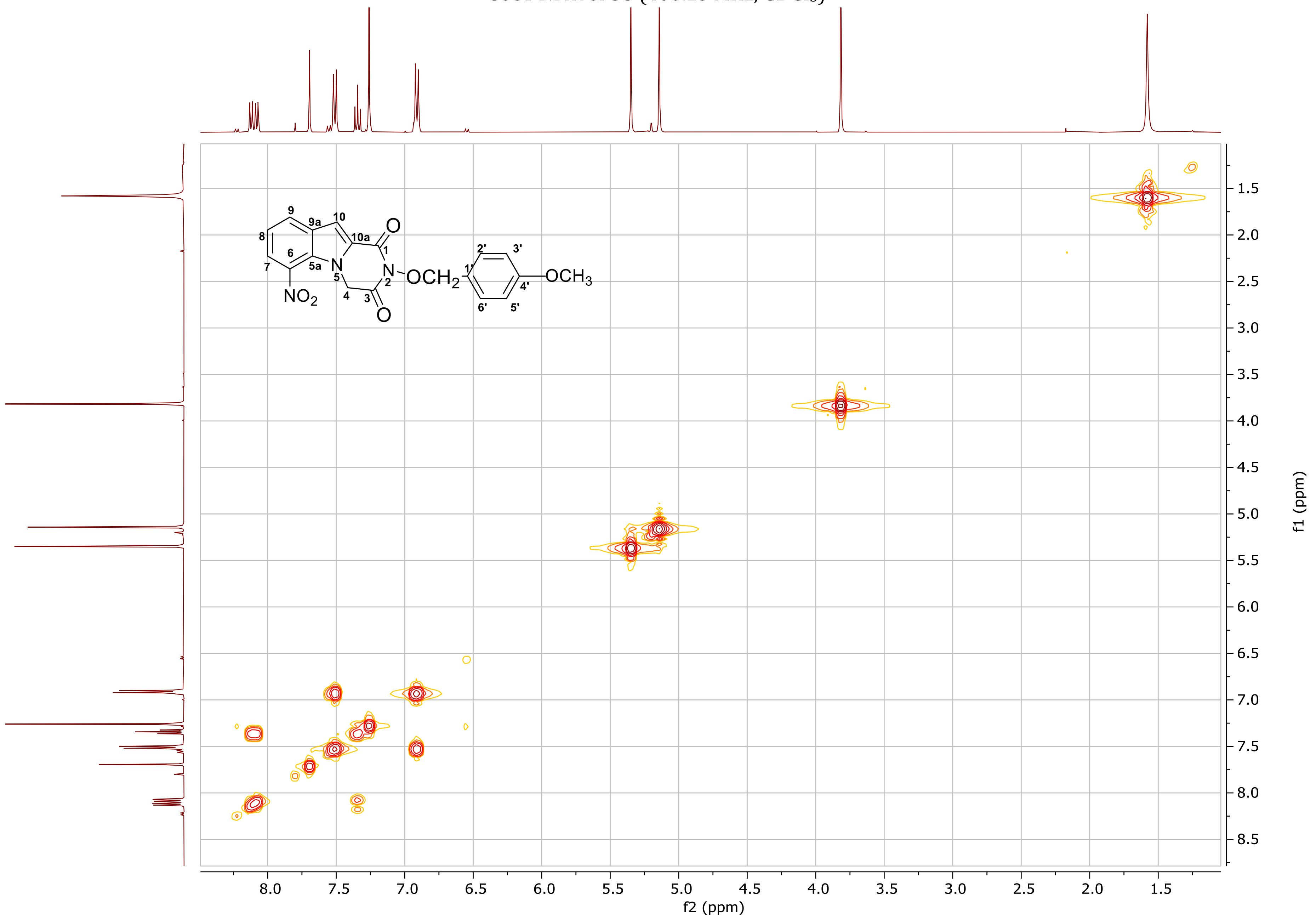

HSQC NMR of **35** (400.13 MHz, $CDCl_3$)

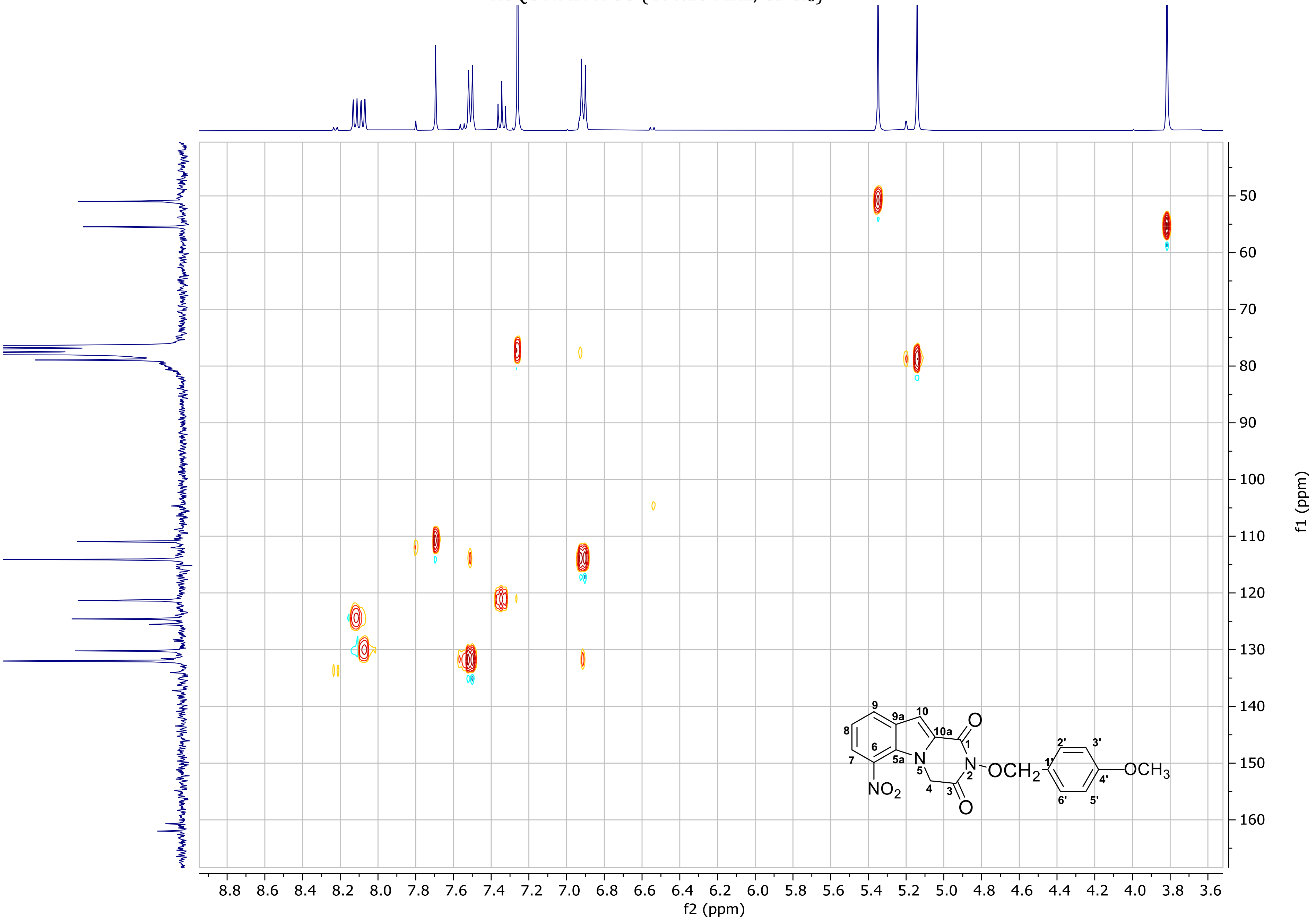

HMBC NMR of **35** (400.13 MHz, $CDCl_3$)

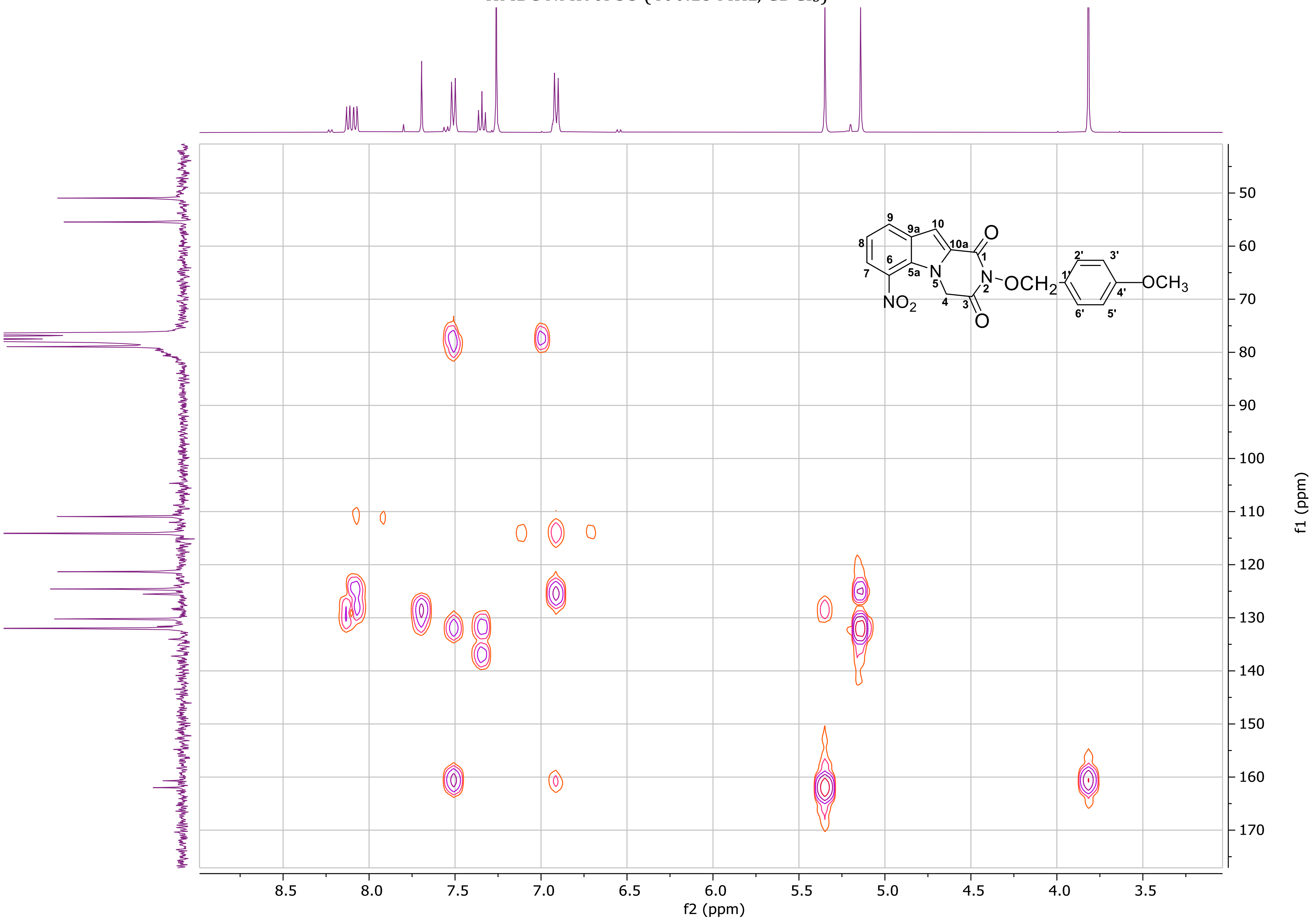

^{1}H NMR of **6** (400.13 MHz, DMSO-d_6)

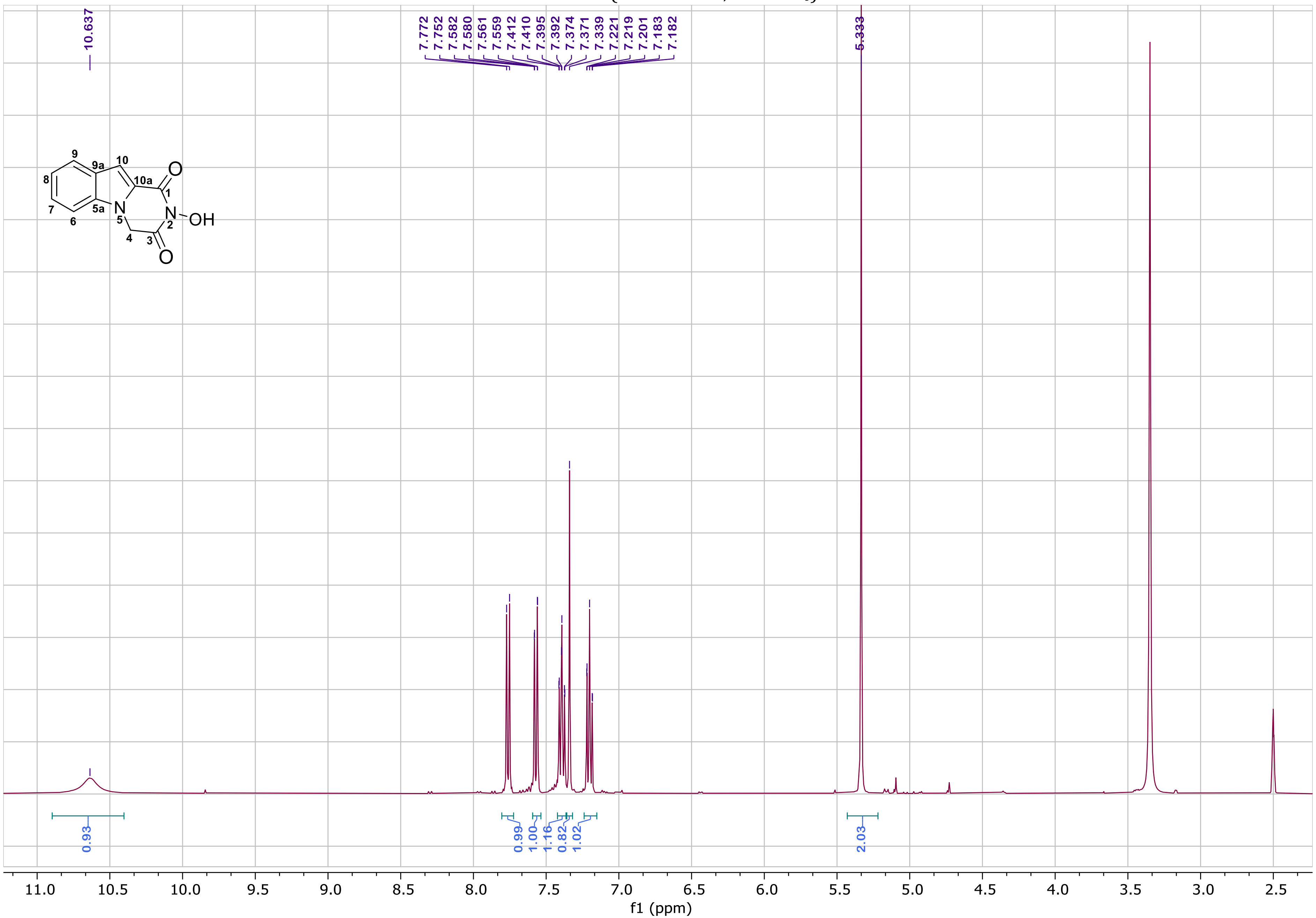

^{13}C NMR of **6** (50.32 MHz, DMSO-d_6)

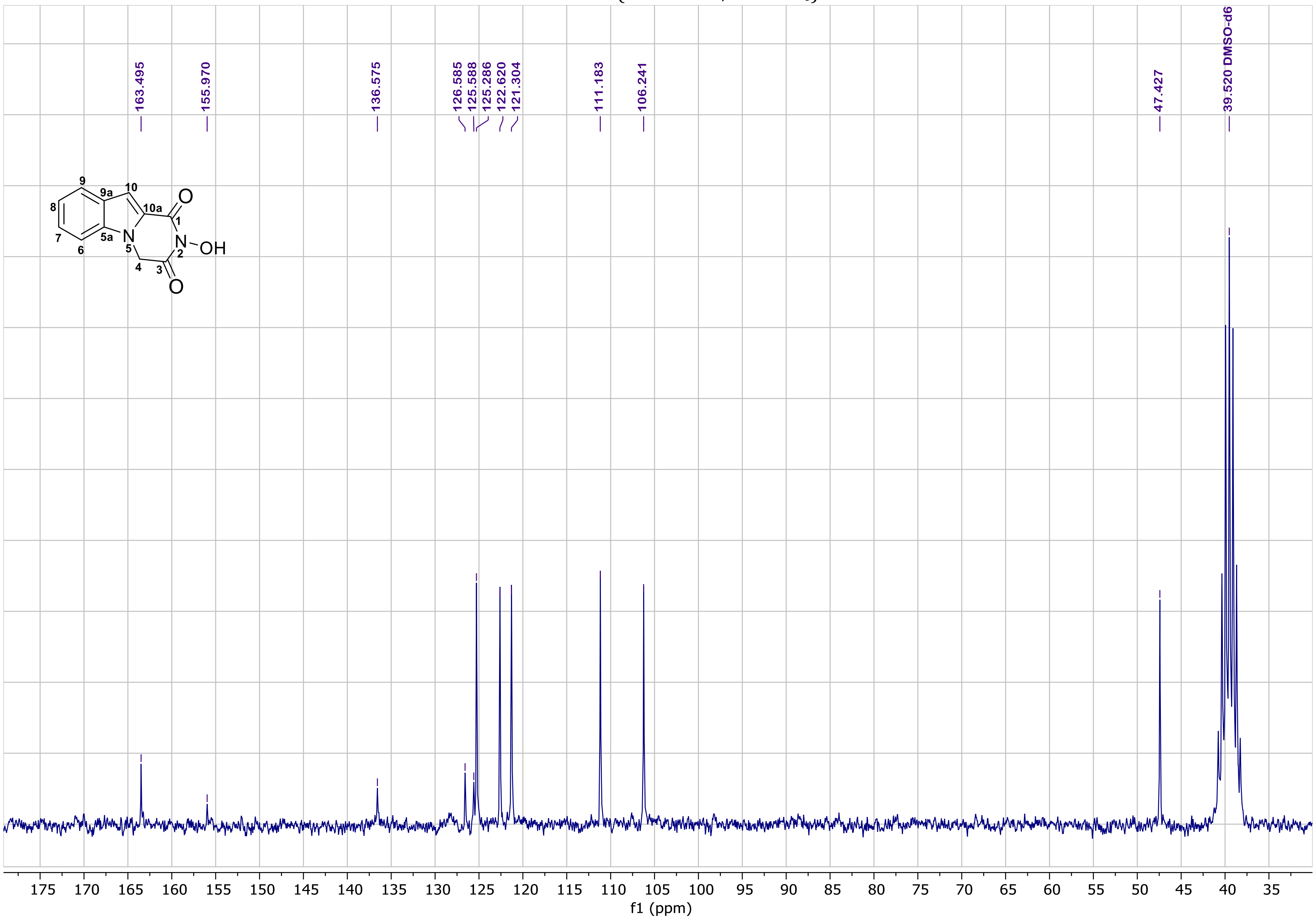

DEPT NMR of **6** (50.32 MHz, DMSO-d_6)

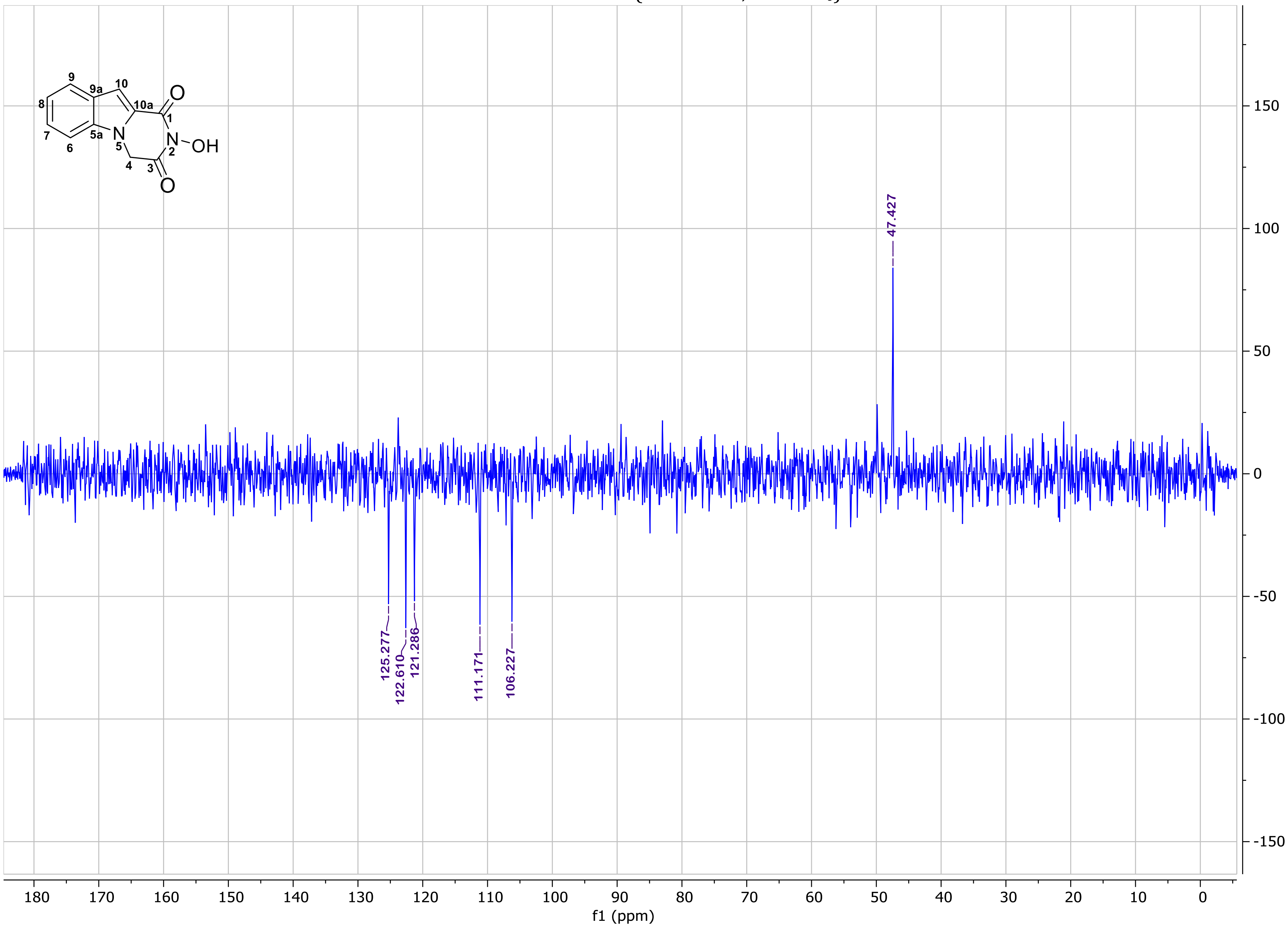

COSY NMR of **6** (400.13 MHz, DMSO-d_6)

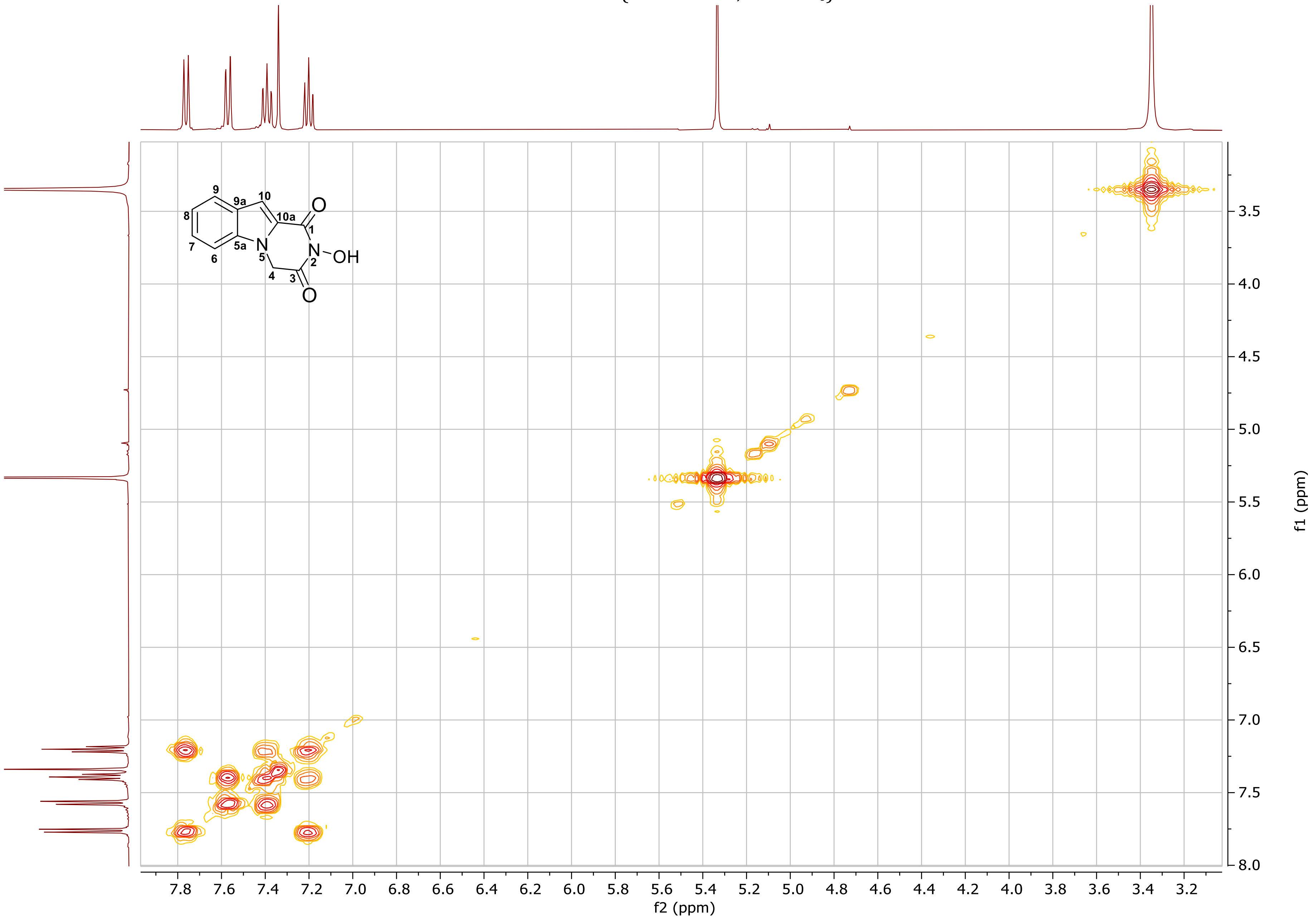

HSQC NMR of **6** (400.13 MHz, DMSO-d_6)

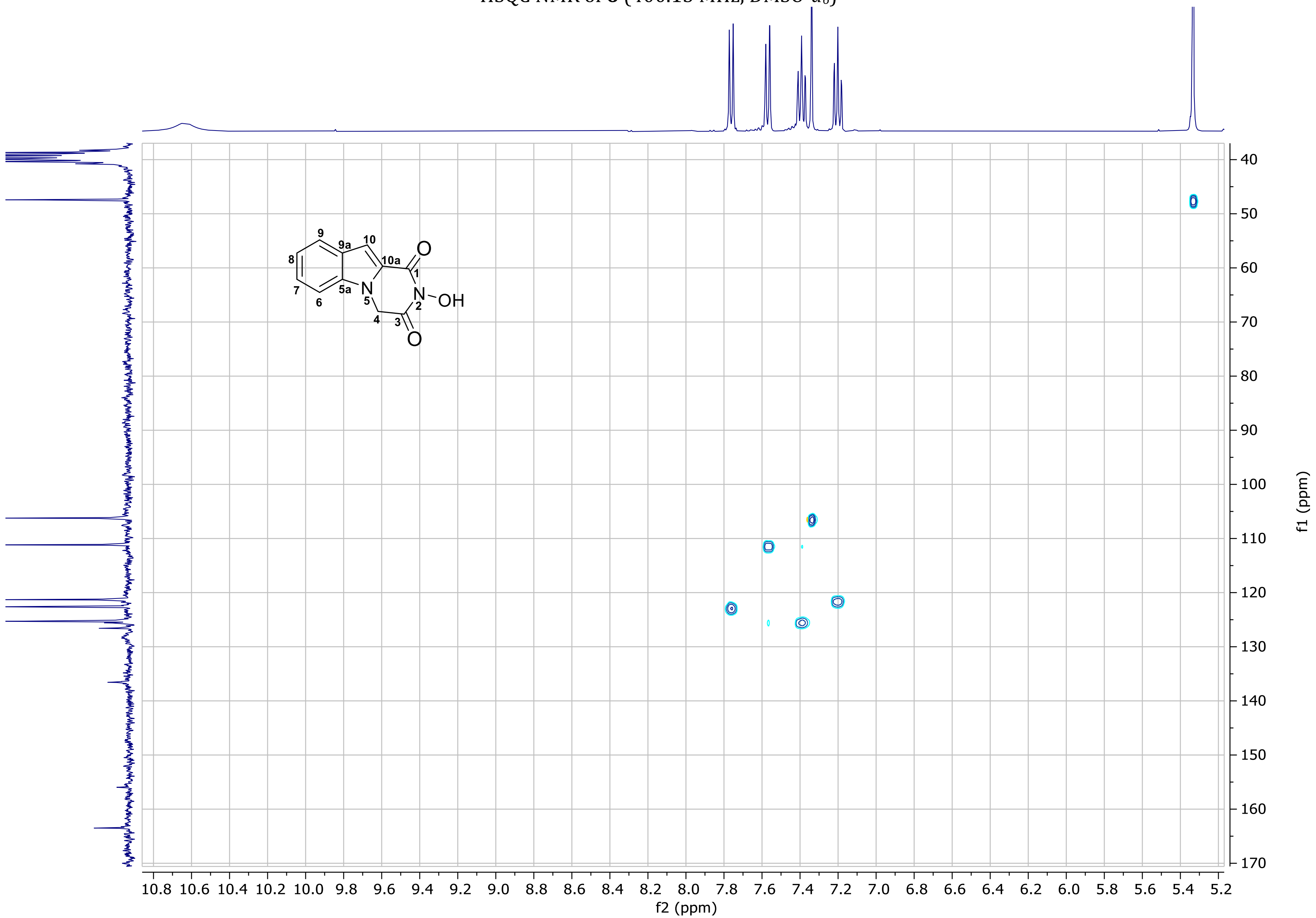

HMBC NMR of **6** (400.13 MHz, DMSO-d_6)

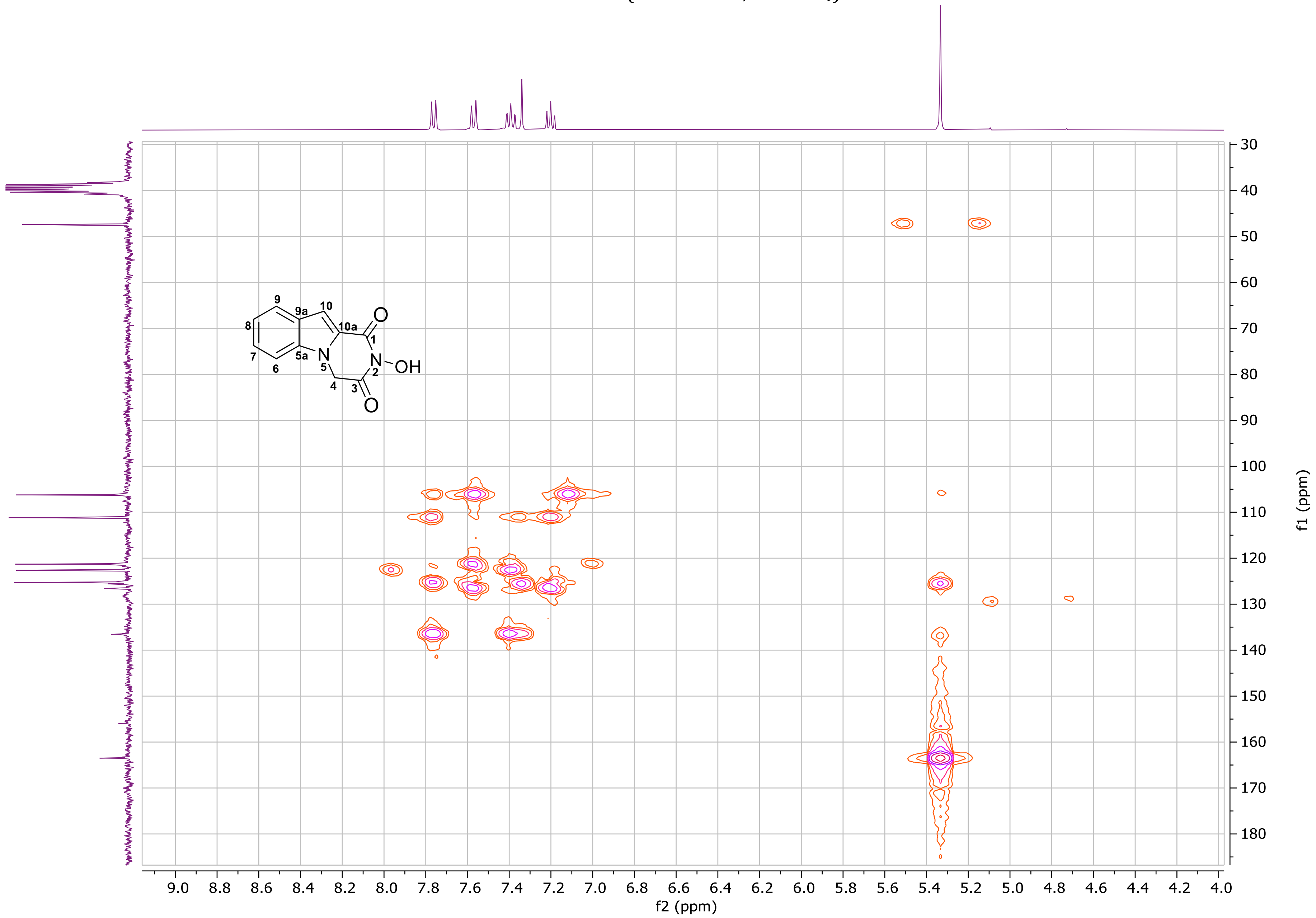

^{1}H NMR of **12** (600.11 MHz, DMSO-d_6)

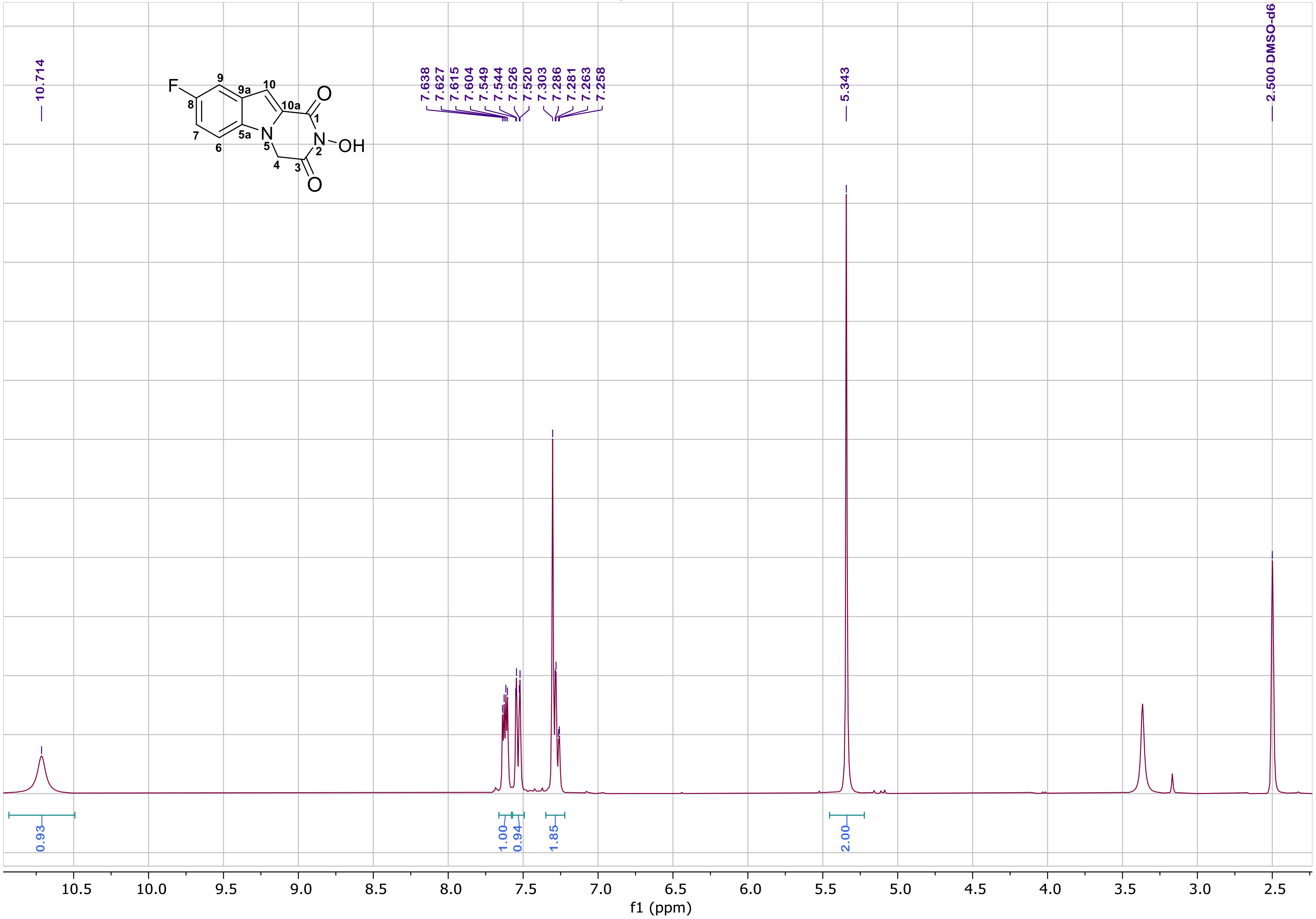

^{13}C NMR of **12** (50.32 MHz, DMSO-d_6)

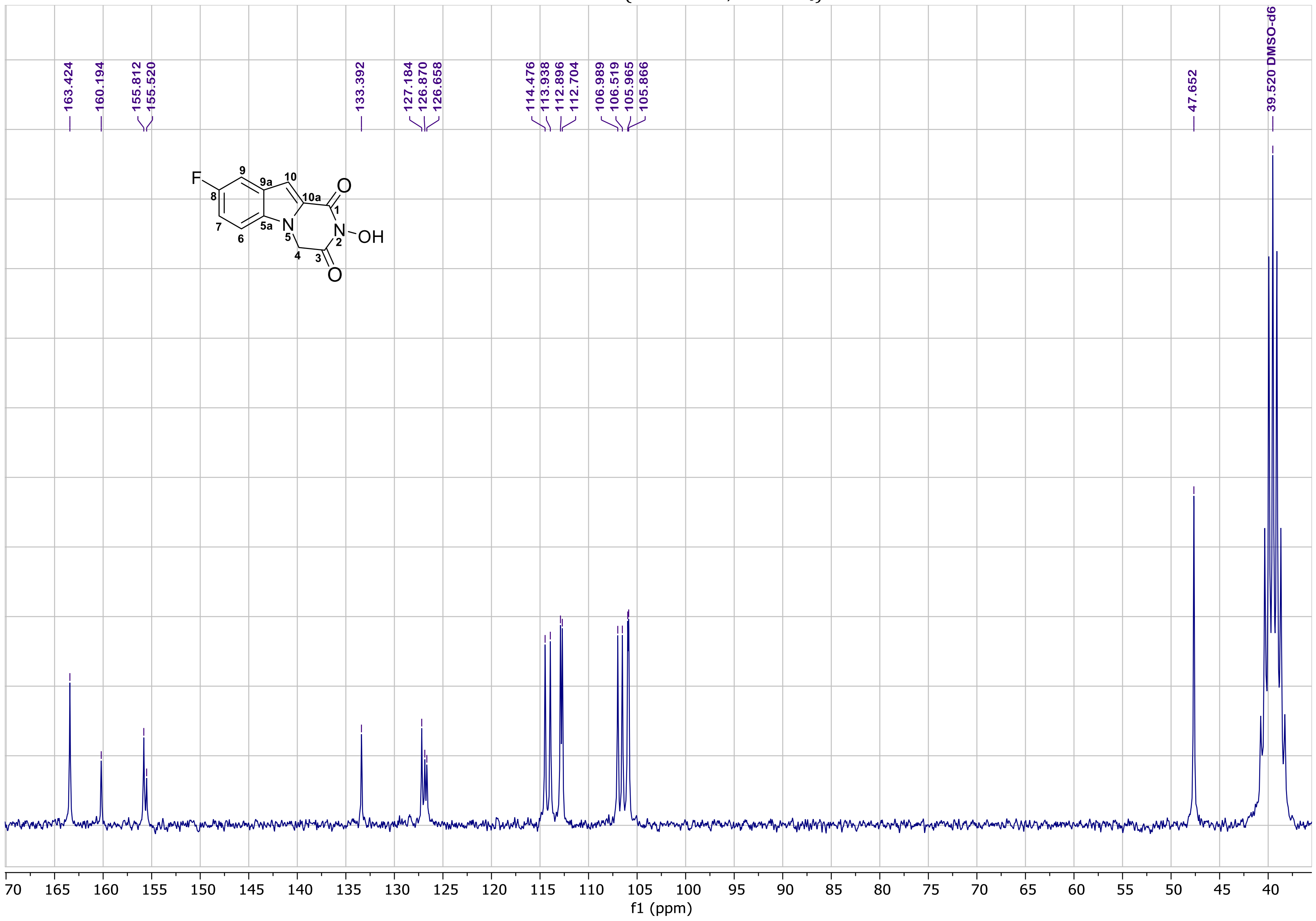

DEPT NMR of **12** (50.32 MHz, DMSO-d_6)

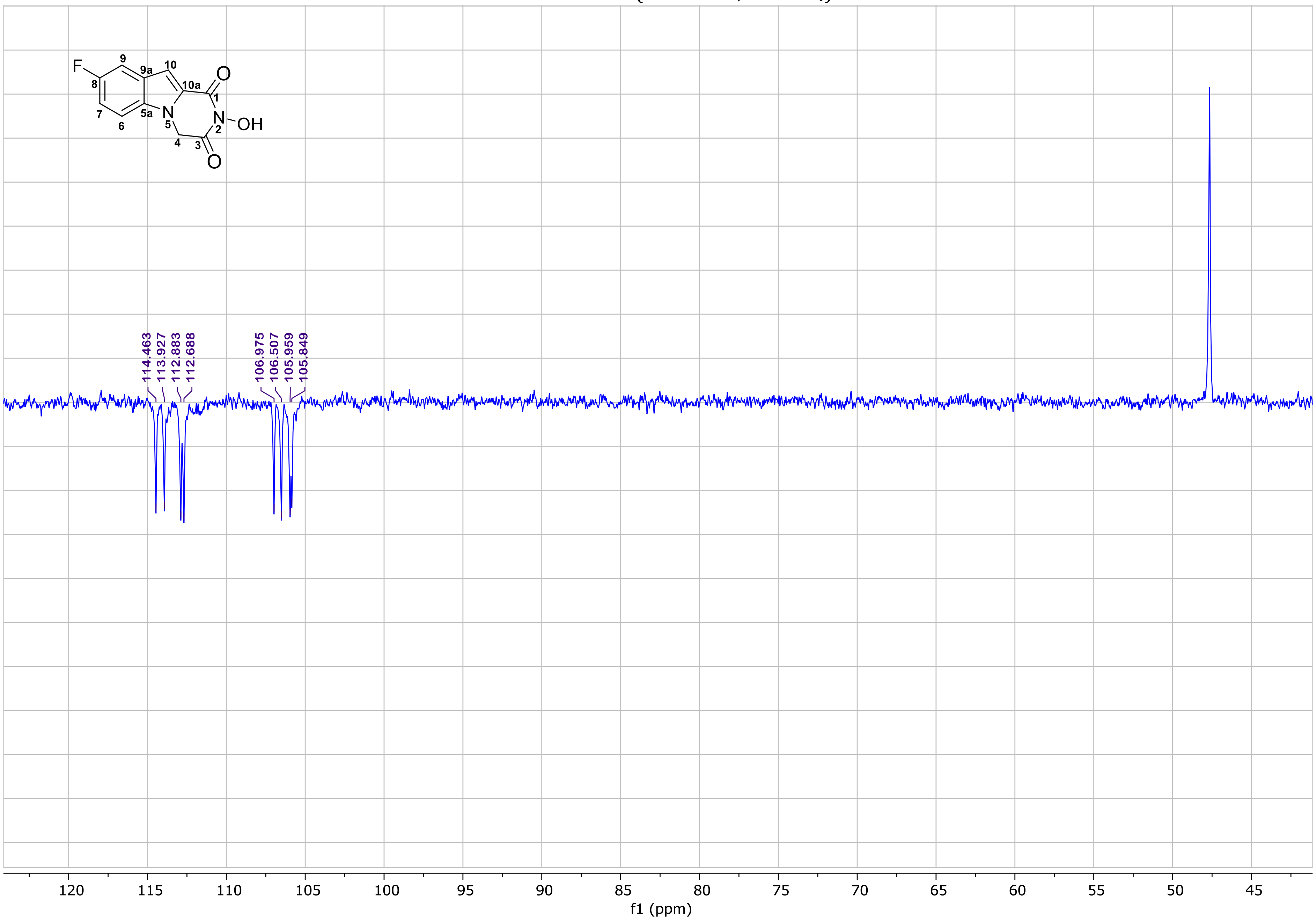

COSY NMR of **12** (600.11 MHz, DMSO-d_6)

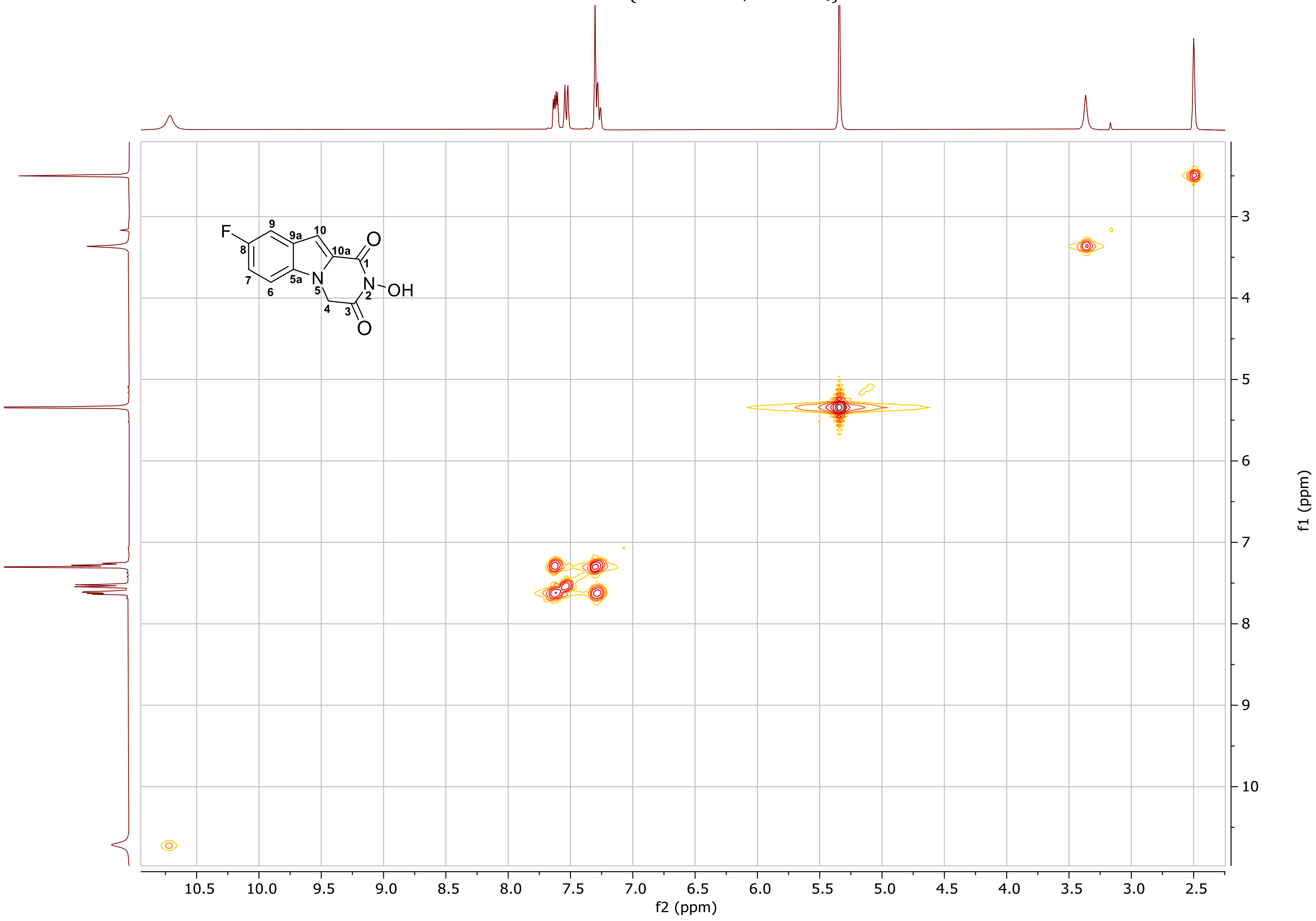

HSQC-DEPT NMR of **12** (600.11 MHz, DMSO-d_6)

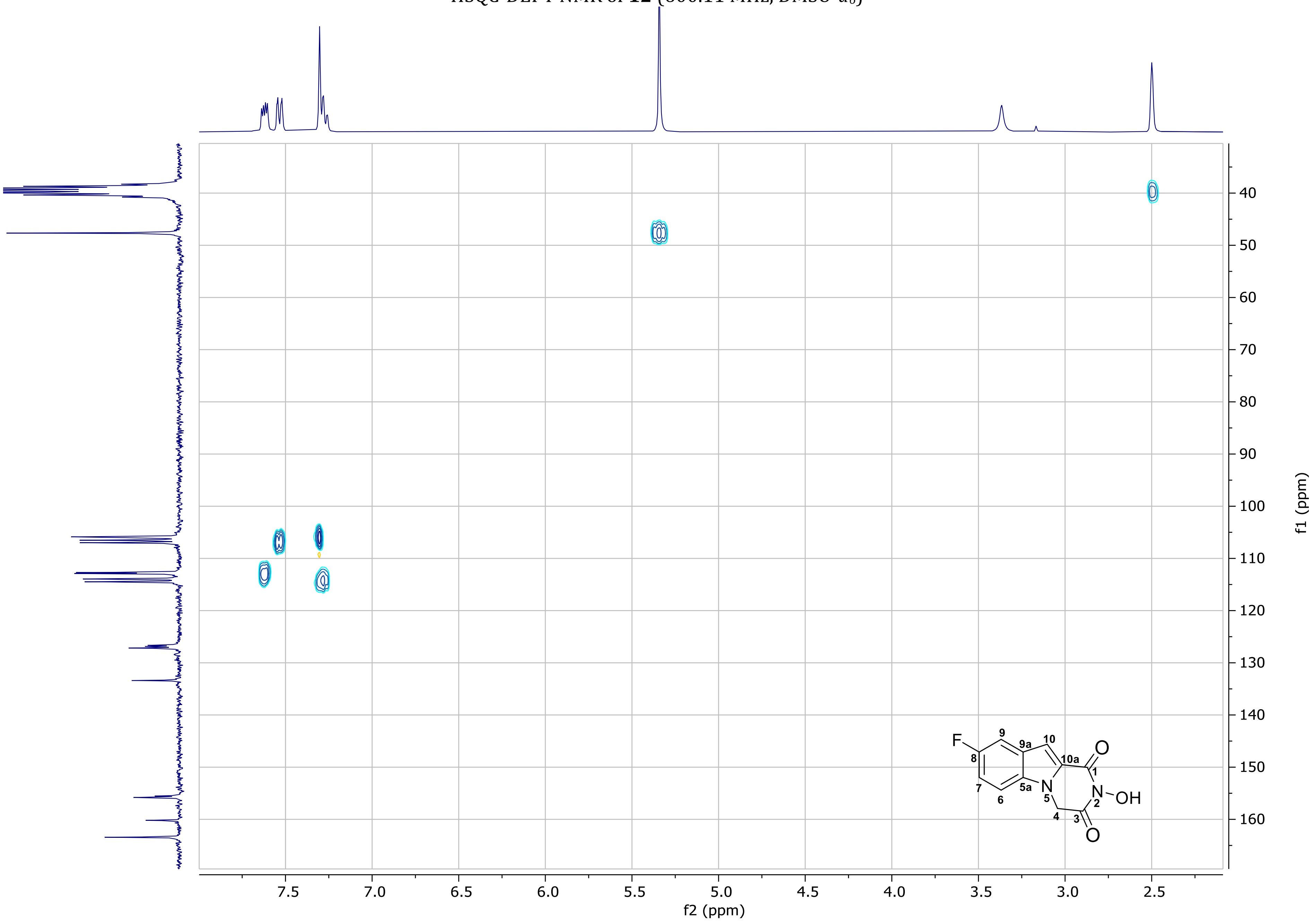

HMBC NMR of **12** (600.11 MHz, DMSO-d_6)

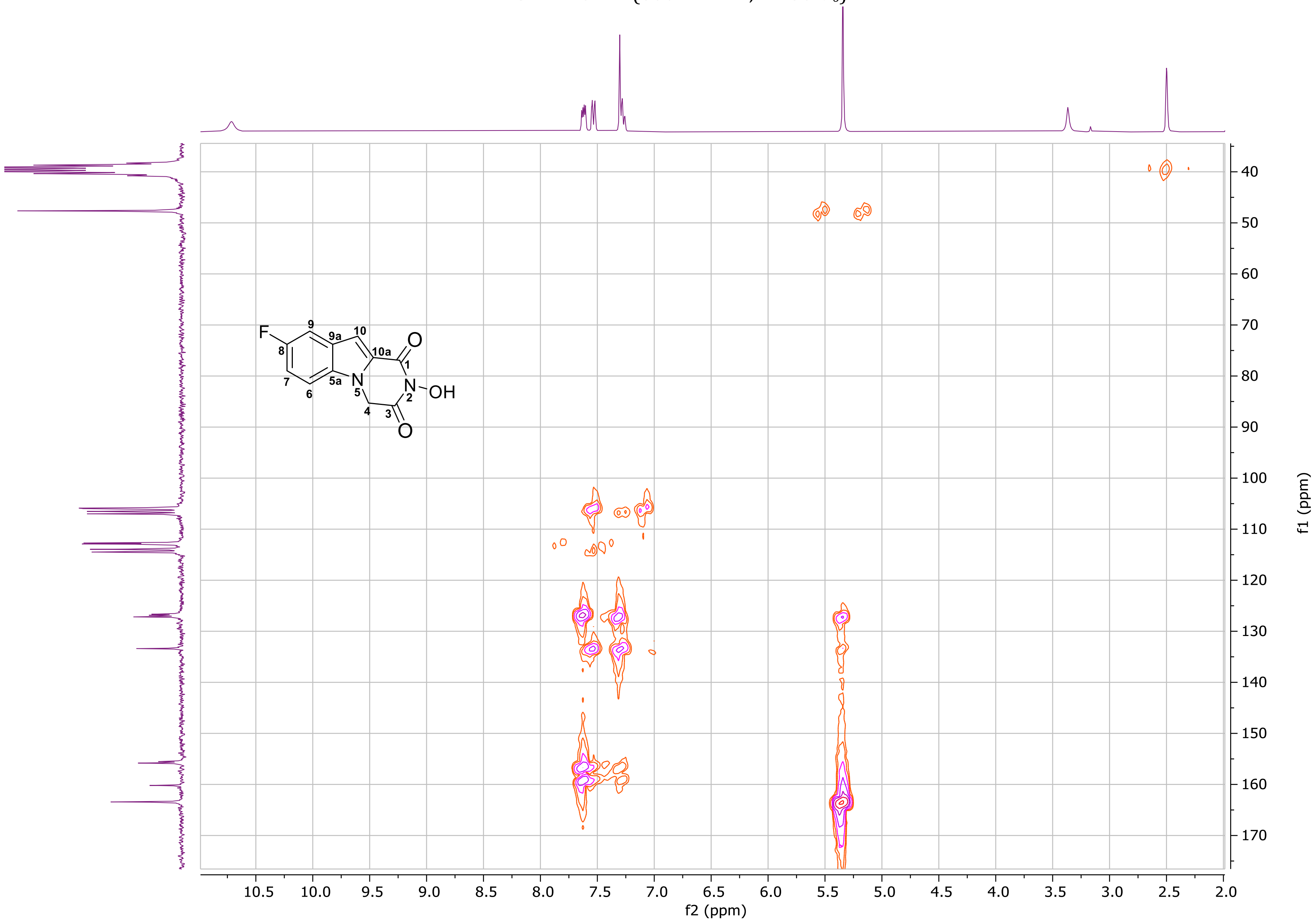

^{1}H NMR of **18** (400.13 MHz, DMSO-d_6)

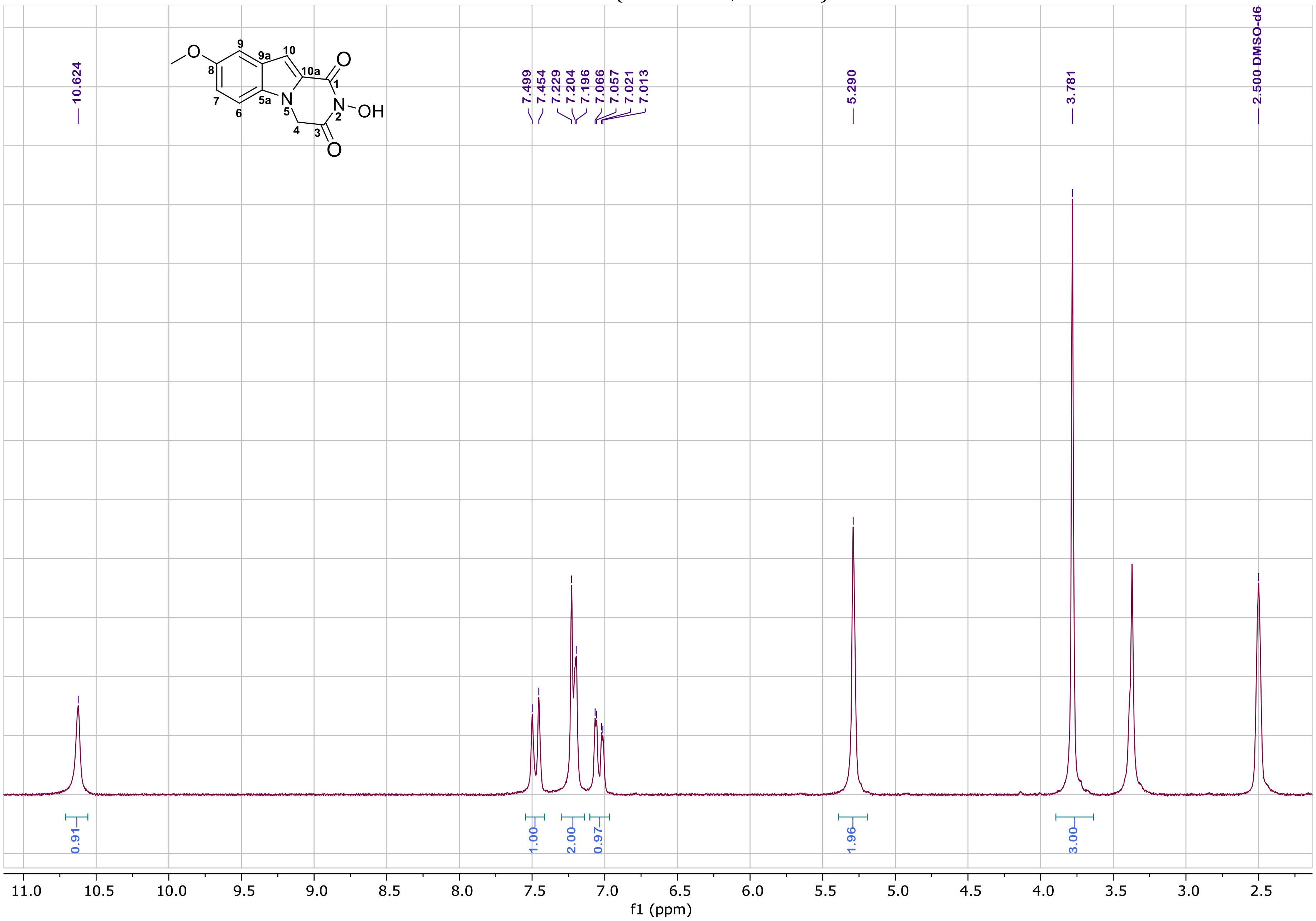

^{13}C NMR of **18** (50.32 MHz, DMSO-d_6)

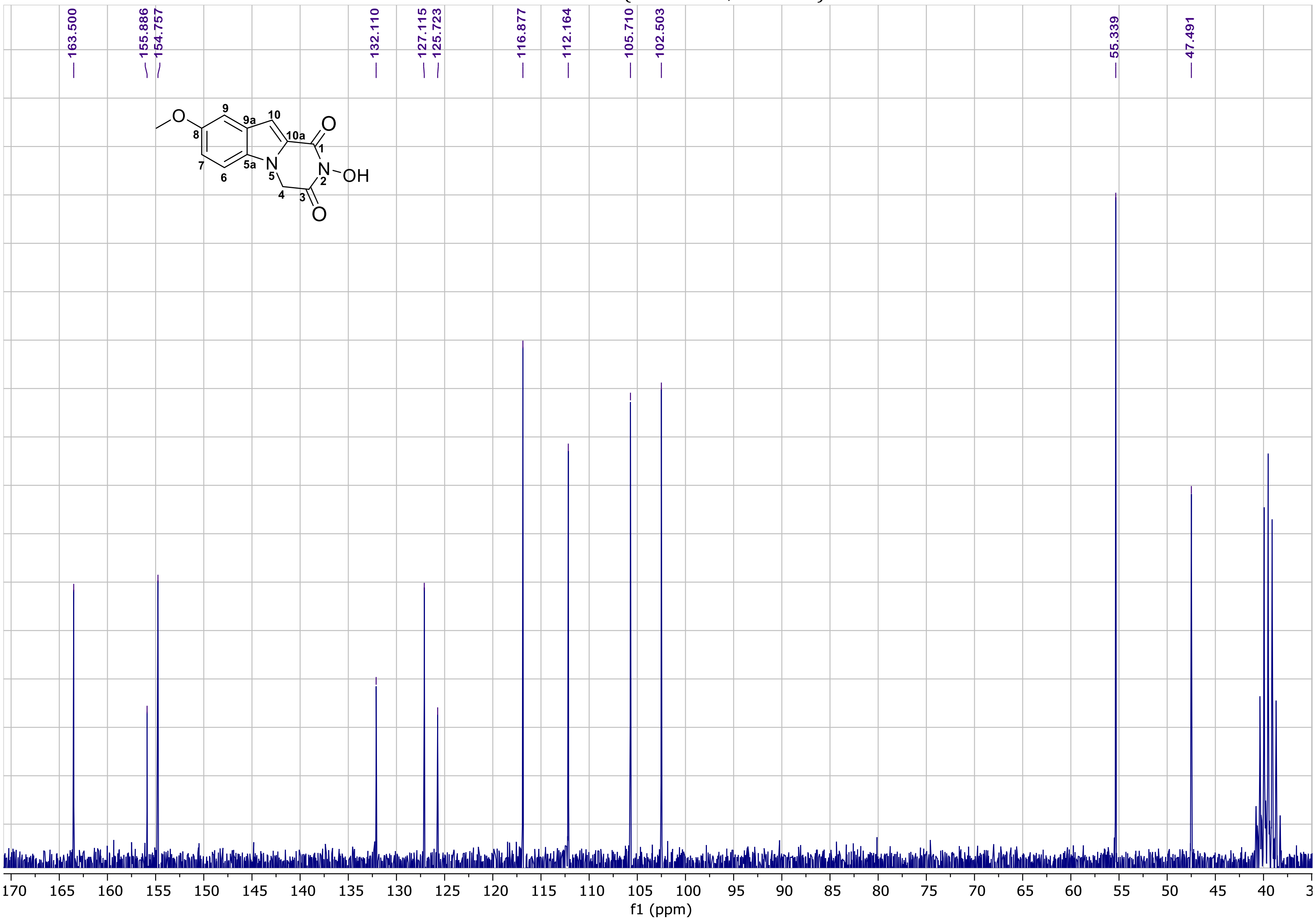

DEPT NMR of **18** (50.32 MHz, DMSO-d_6)

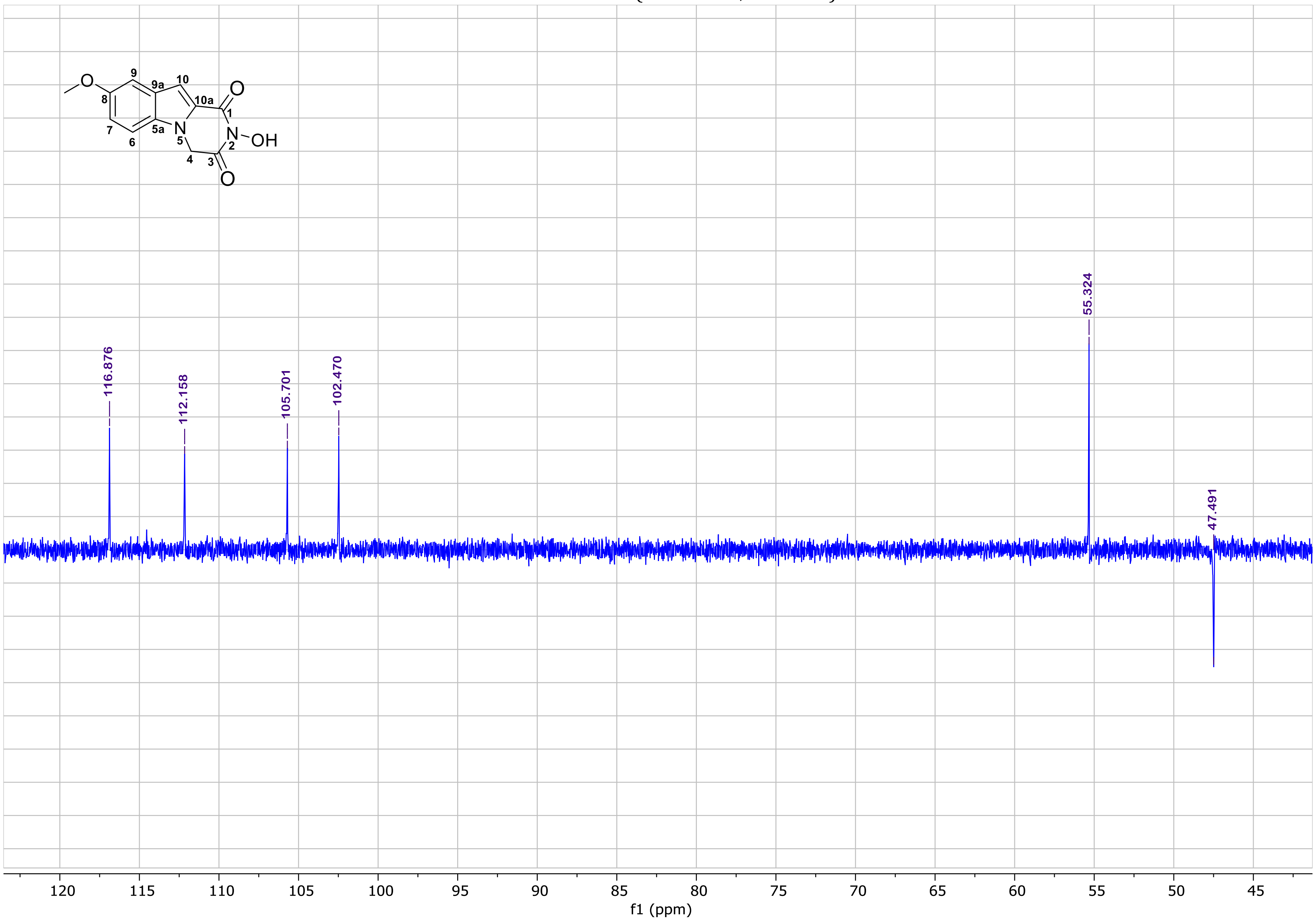

COSY NMR of **18** (400.13 MHz, DMSO-d_6)

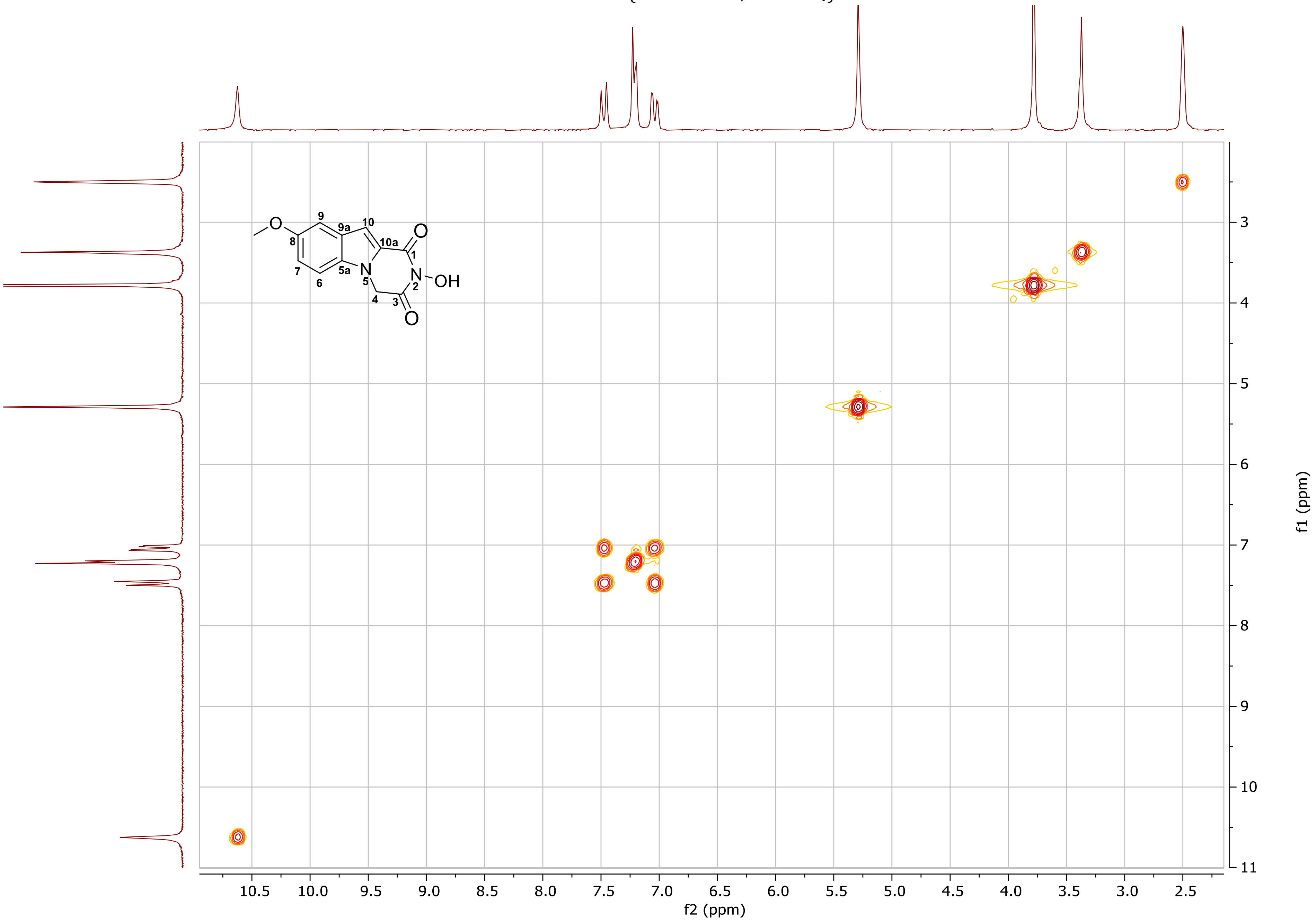

HSQC NMR of **18** (400.13 MHz, DMSO-d_6)

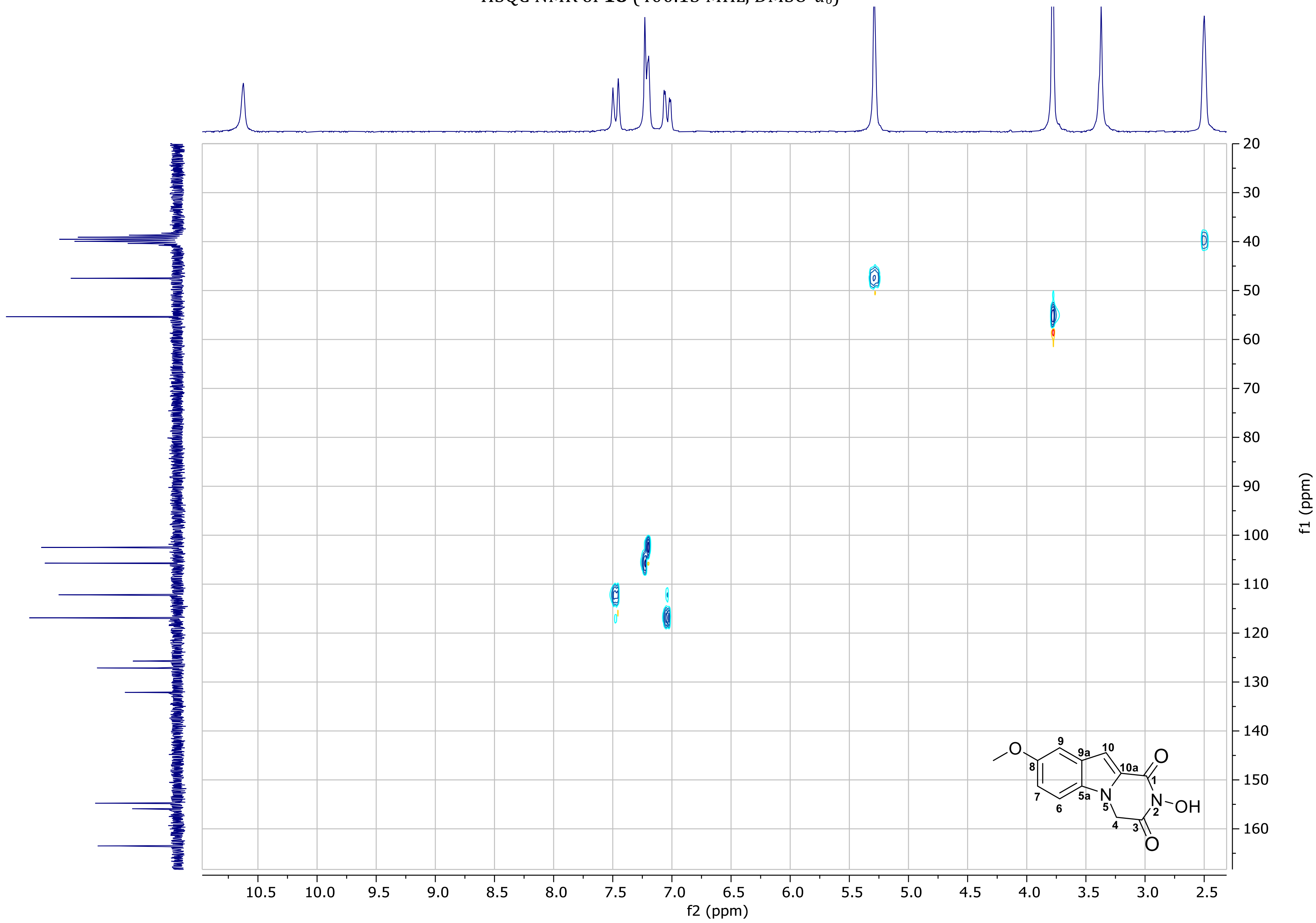

^1H NMR of **24** (400.13 MHz, DMSO-d_6)

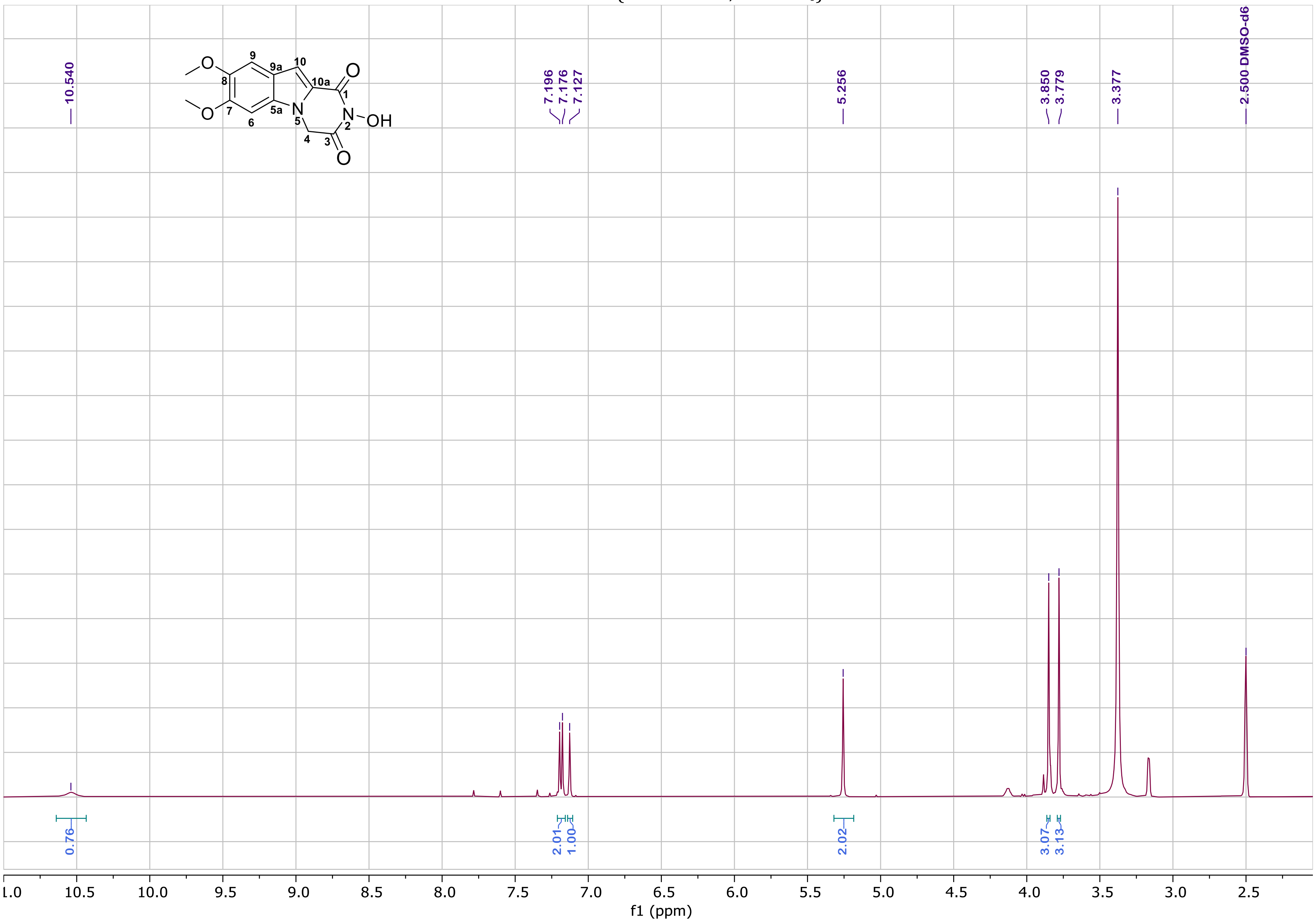

^{13}C NMR of **24** (50.32 MHz, DMSO-d_6)

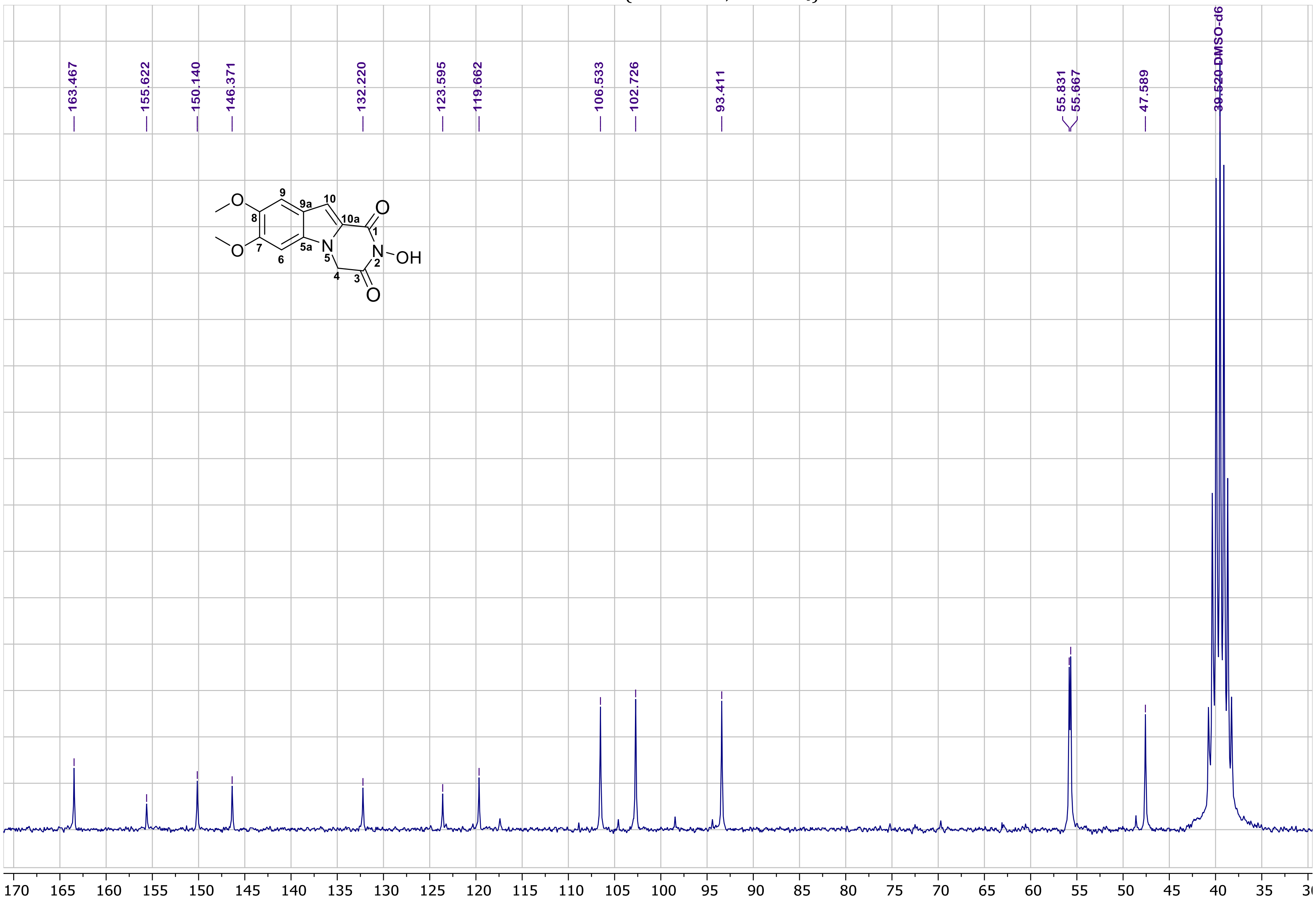

DEPT NMR of **24** (50.32 MHz, DMSO-d_6)

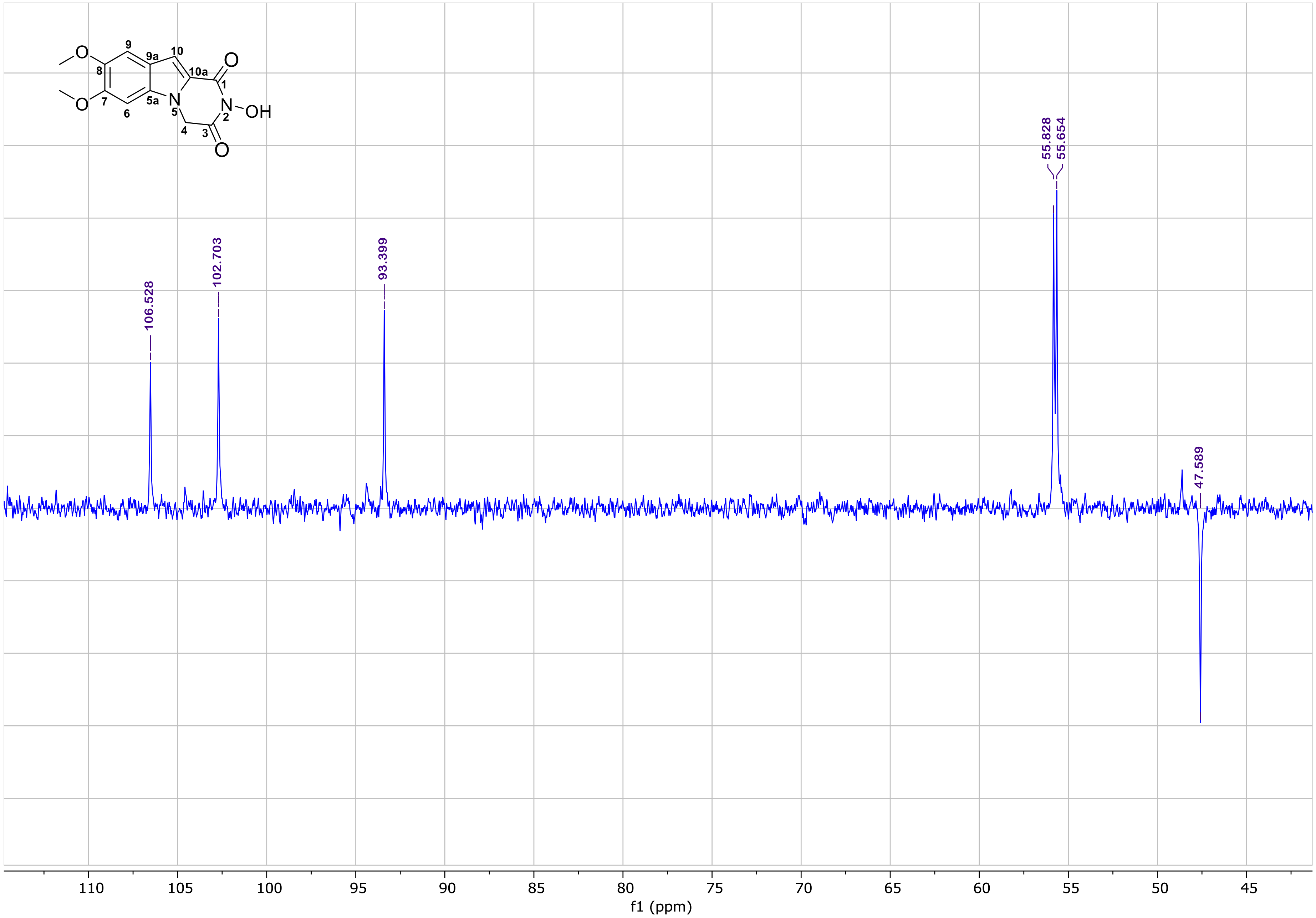

COSY NMR of **24** (400.13 MHz, DMSO-d_6)

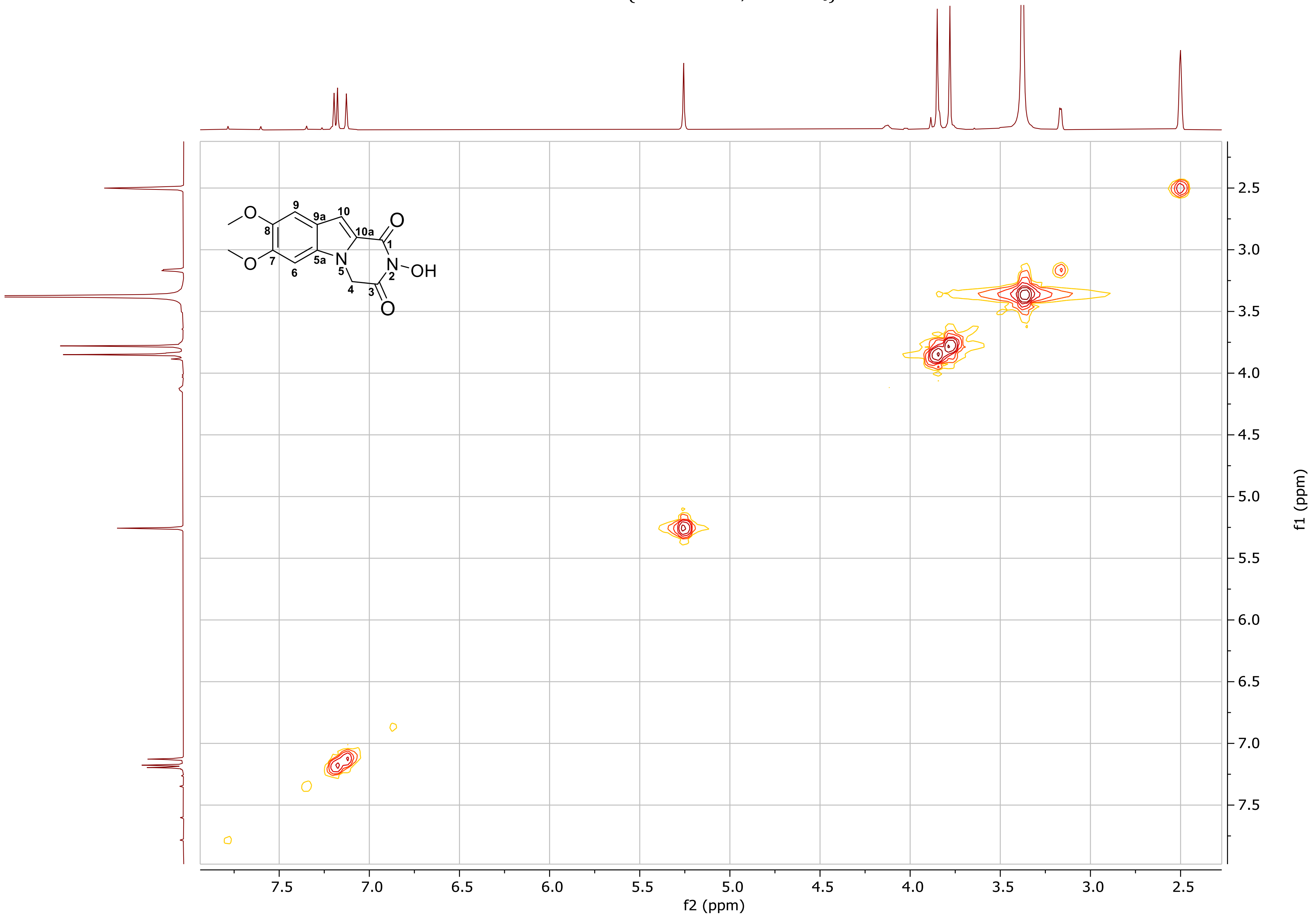

HSQC NMR of **24** (400.13 MHz, DMSO-d_6)

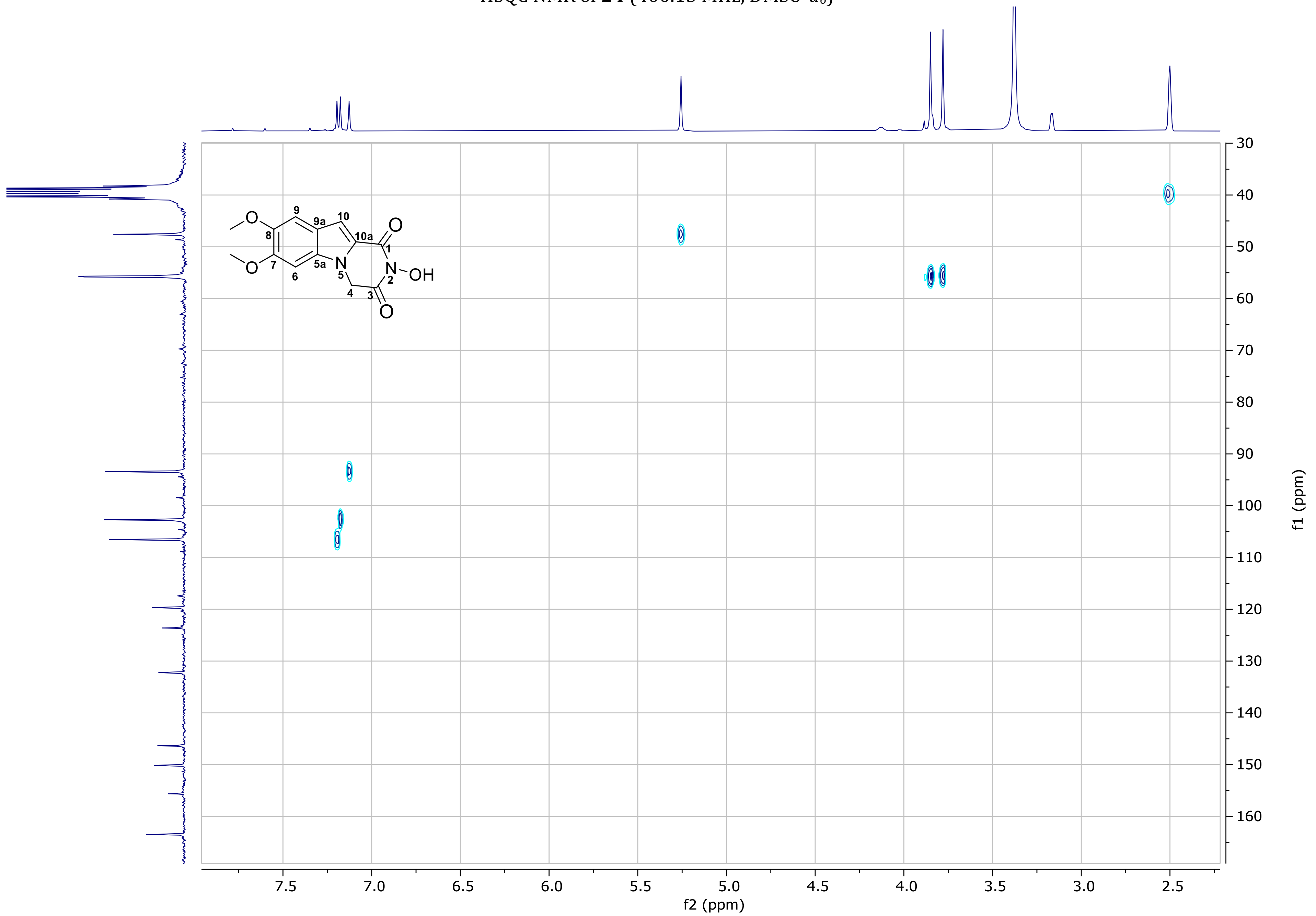

HMBC NMR of **24** (400.13 MHz, DMSO-d_6)

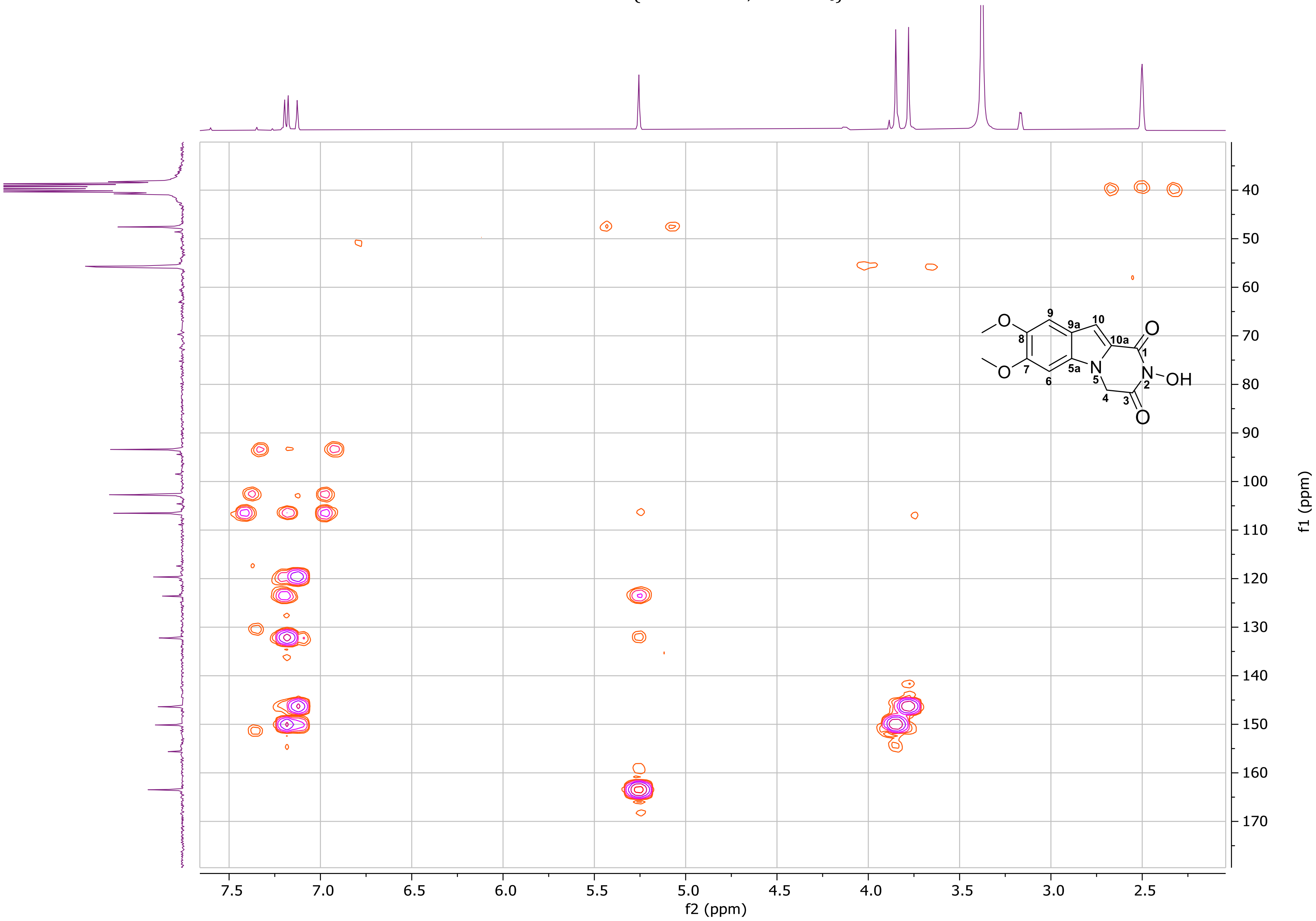

^{1}H NMR of **40** (400.13 MHz, DMSO-d_6)

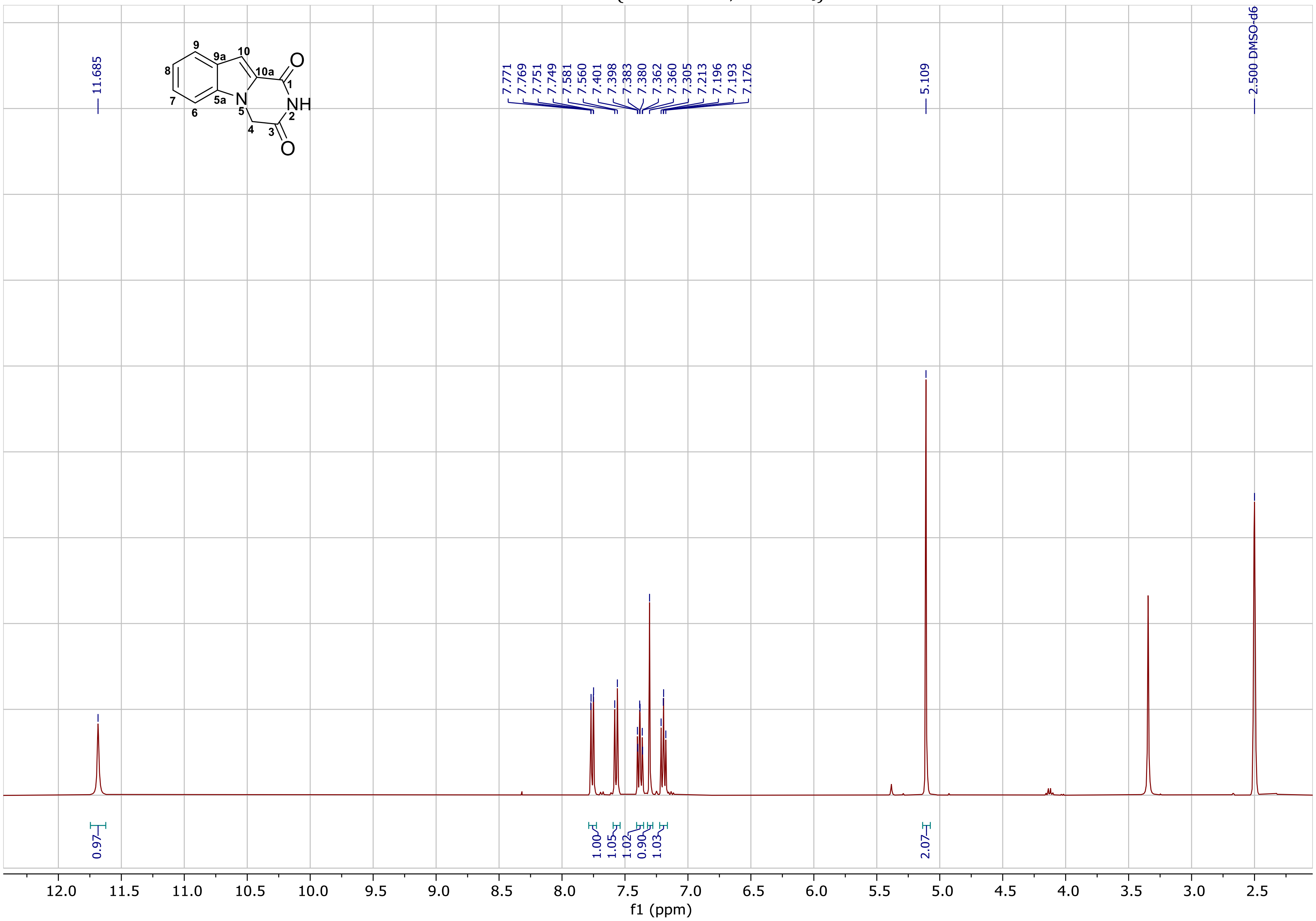

^{13}C NMR of **40** (50.32 MHz, DMSO-d_6)

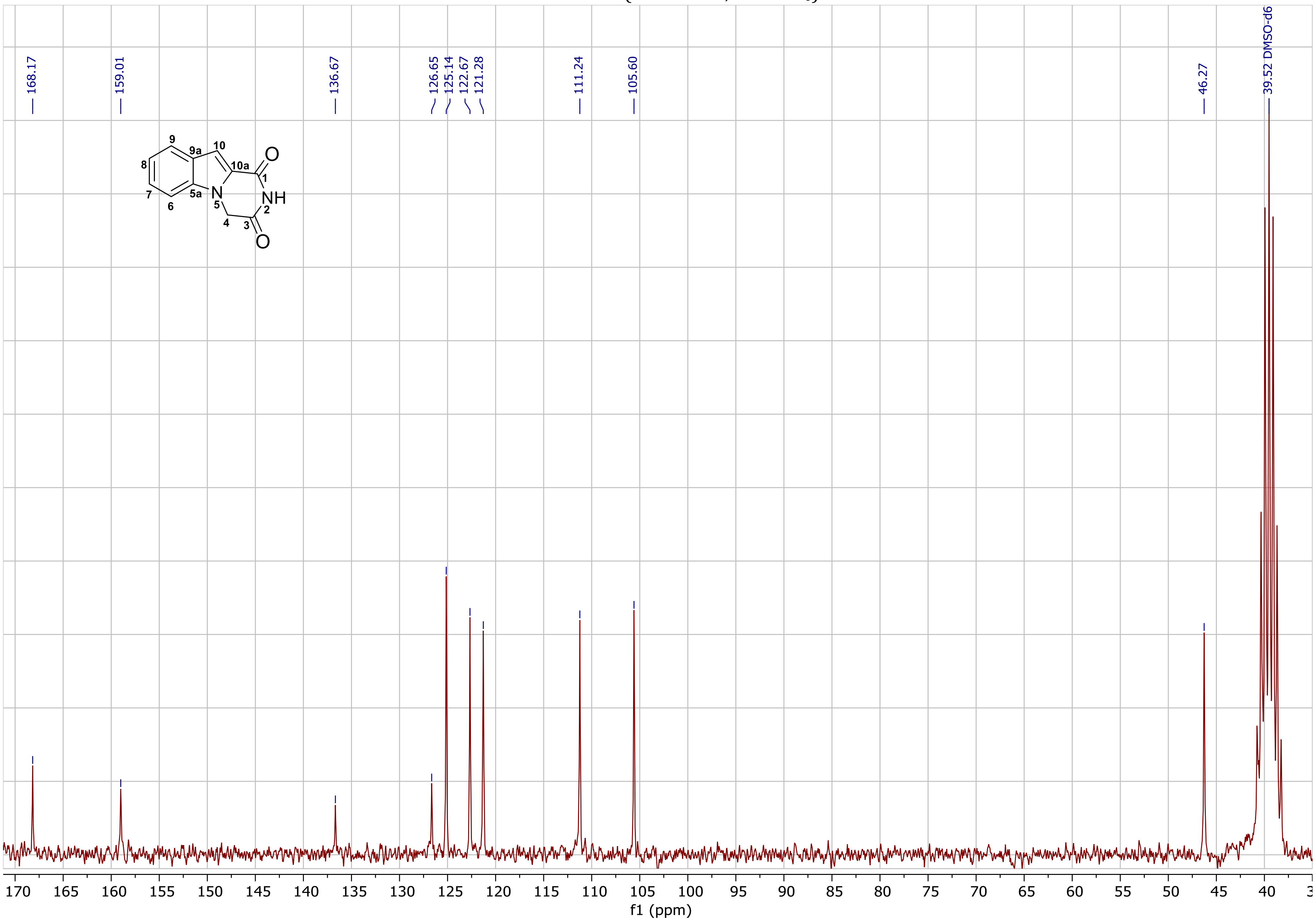

COSY NMR of **40** (400.13 MHz, DMSO-d_6)

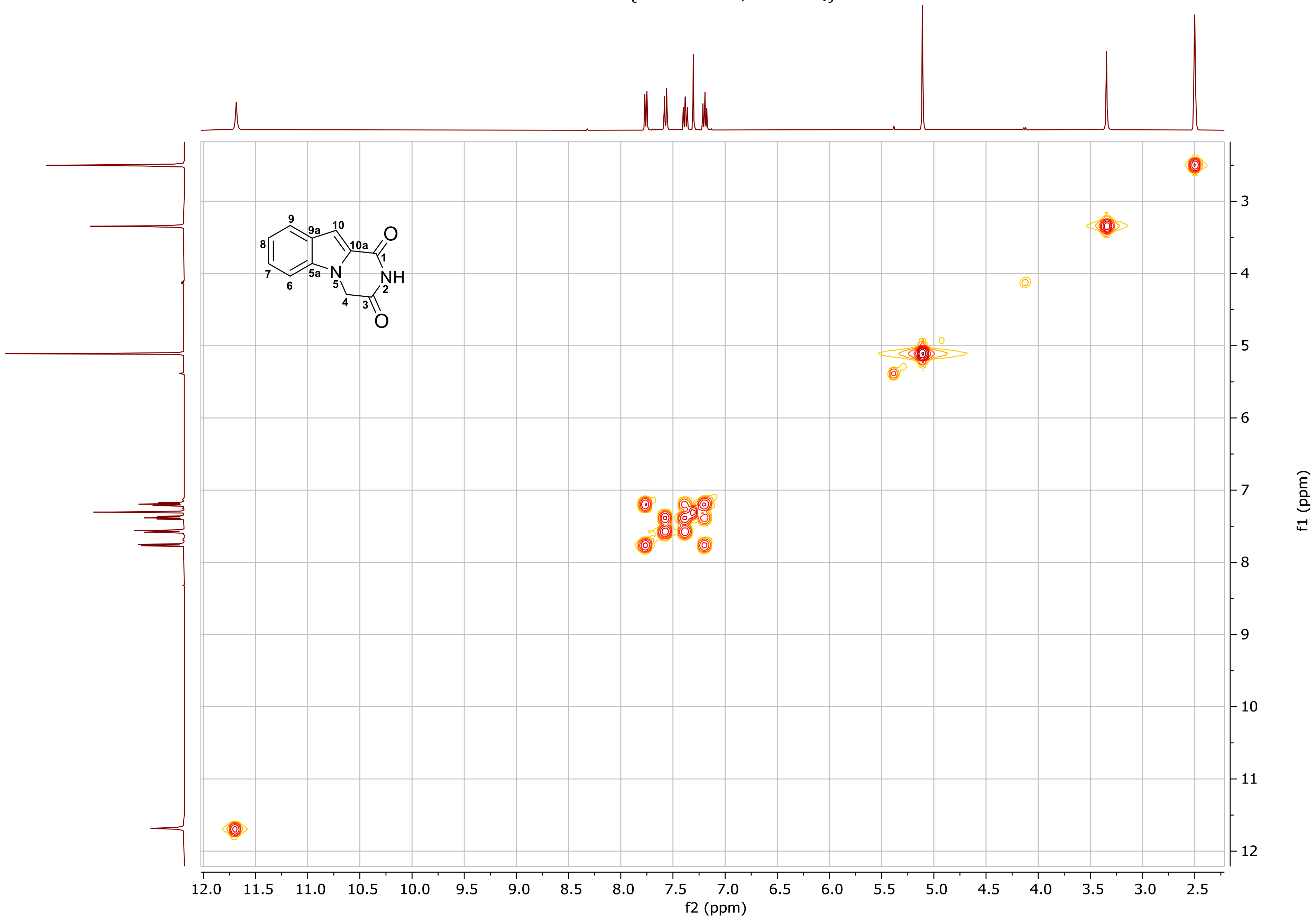

HSQC NMR of **40** (400.13 MHz, DMSO-d_6)

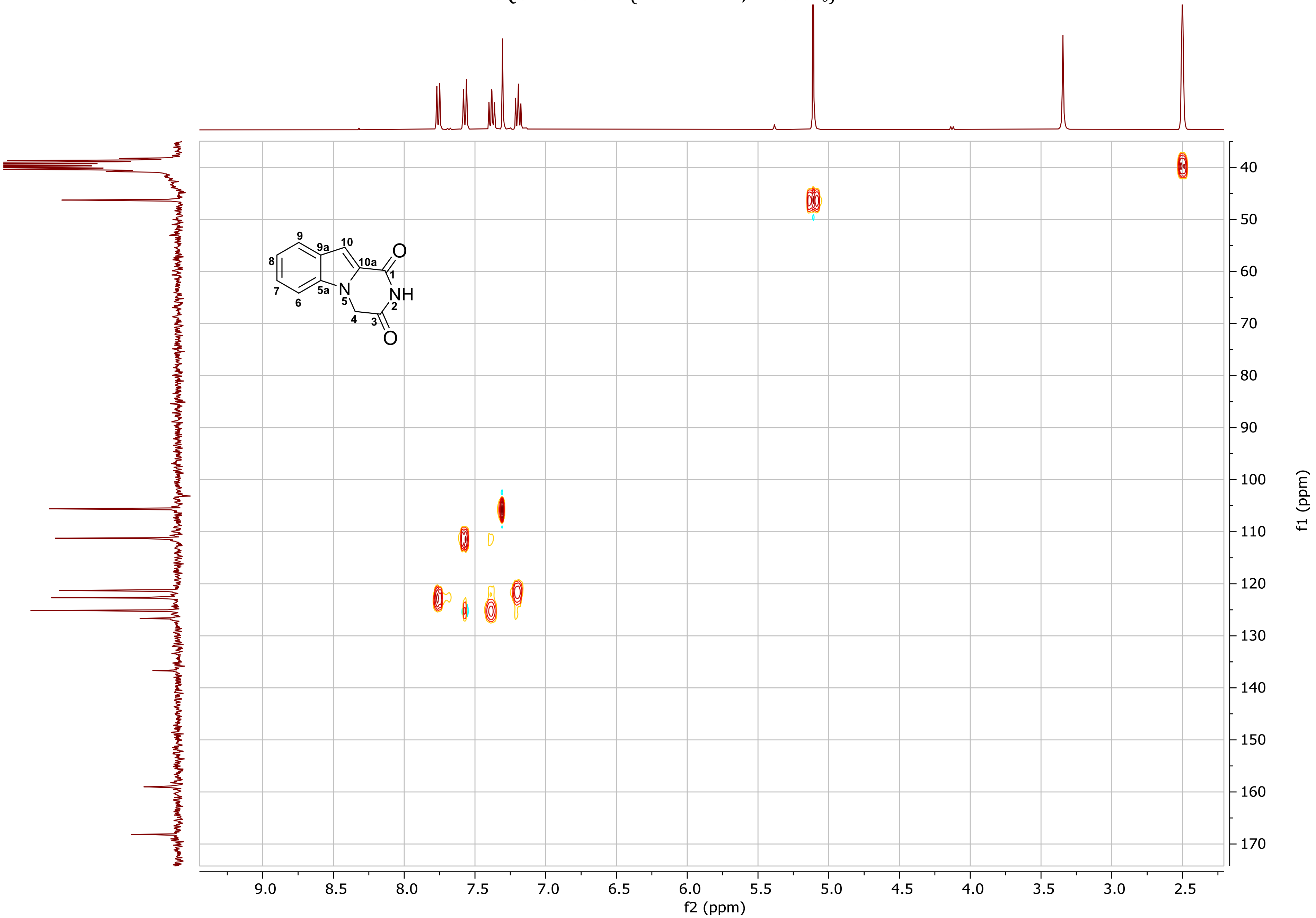

^{1}H NMR of **30** (400.13 MHz, DMSO-d_6)

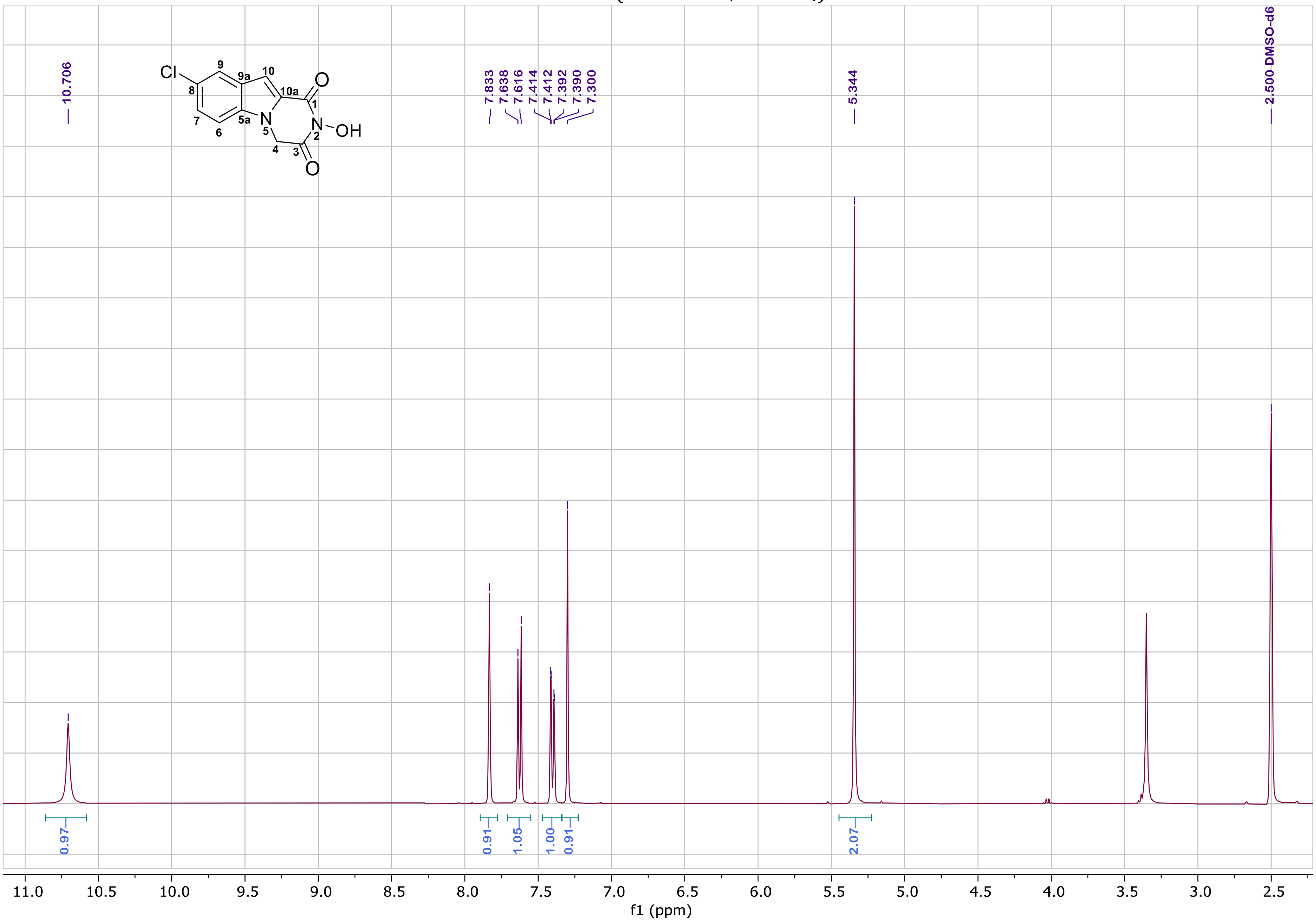

^{13}C NMR of **30** (50.32 MHz, DMSO-d_6)

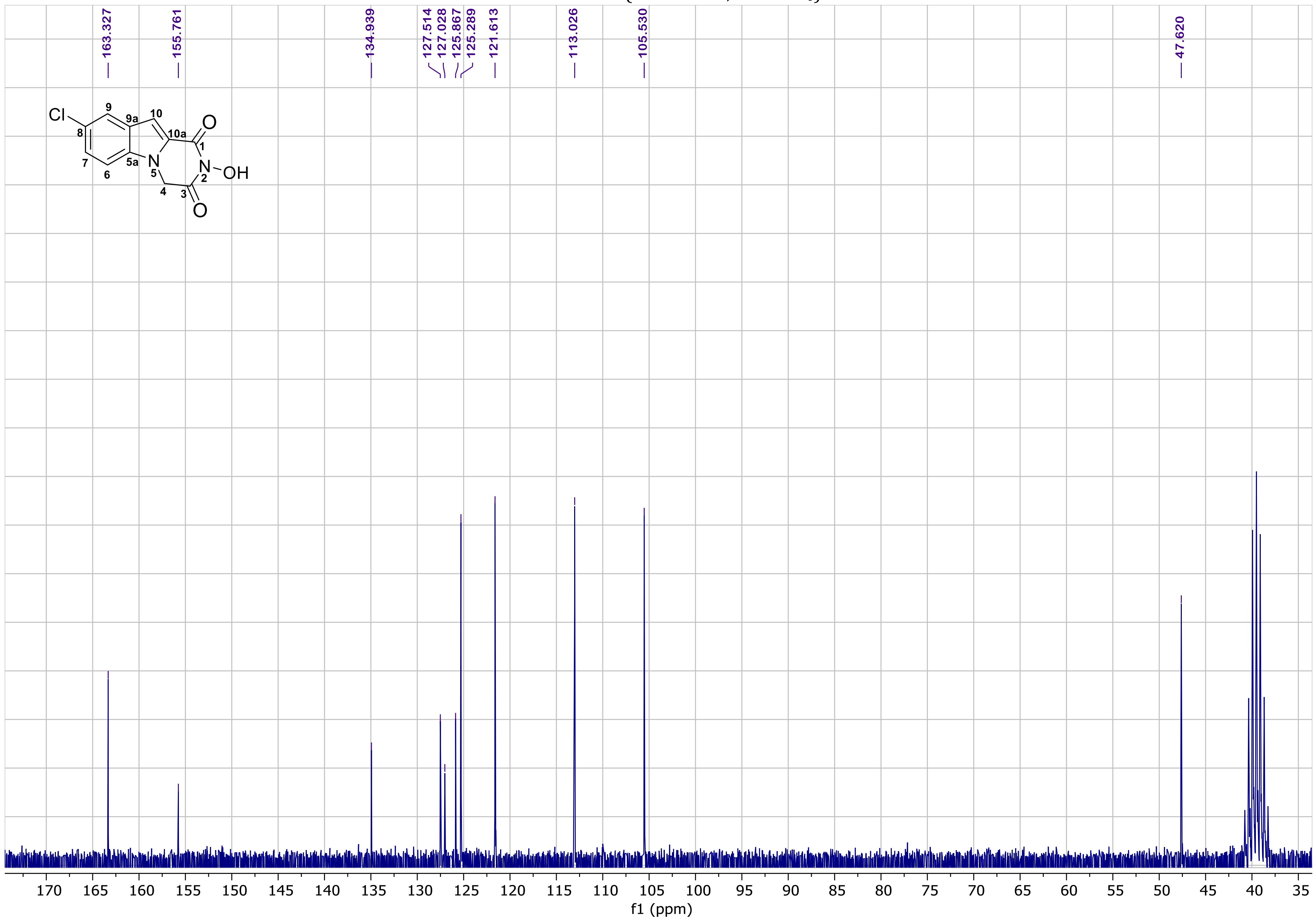

COSY NMR of **30** (400.13 MHz, DMSO-d_6)

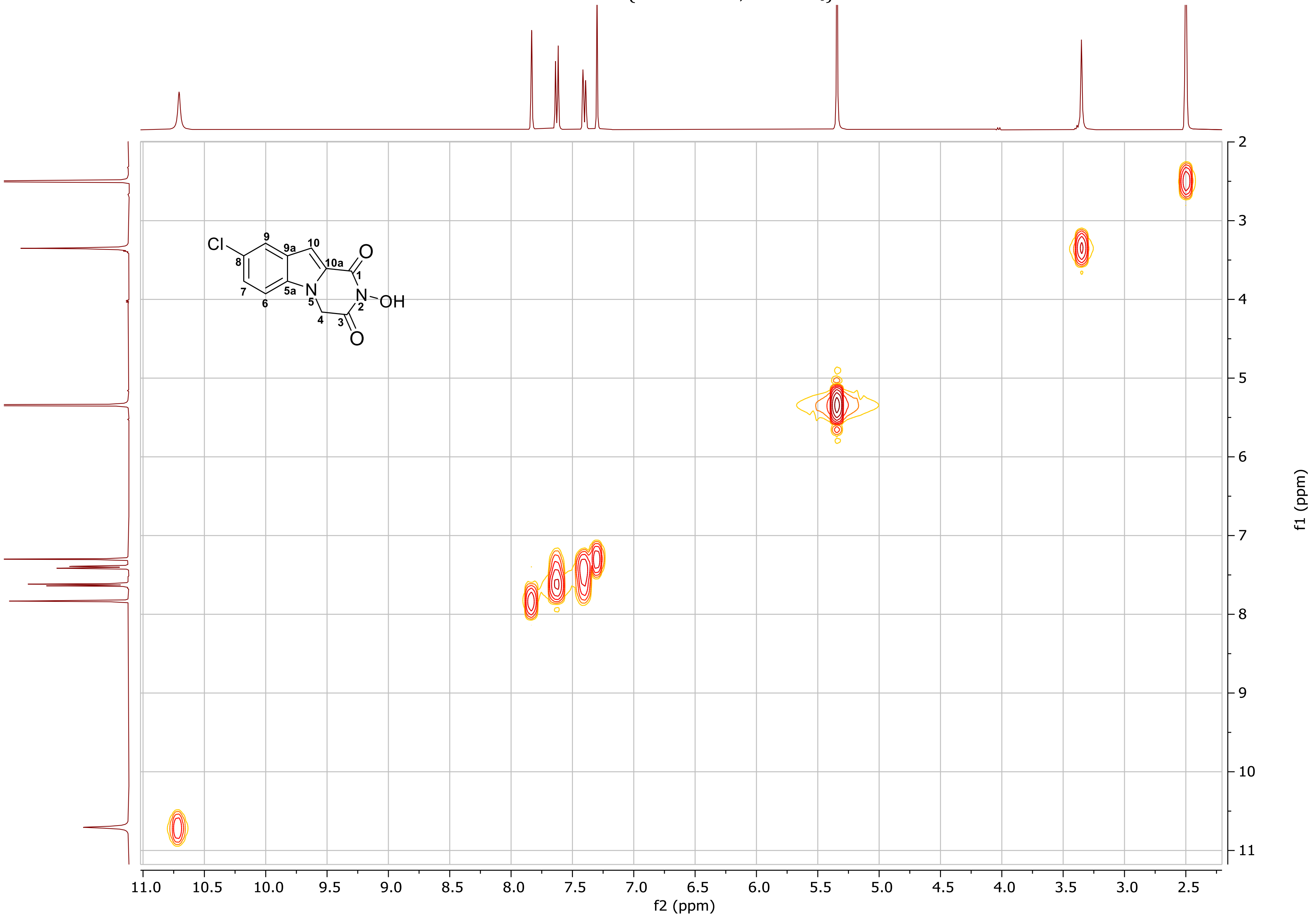

HSQC NMR of **30** (400.13 MHz, DMSO-d_6)

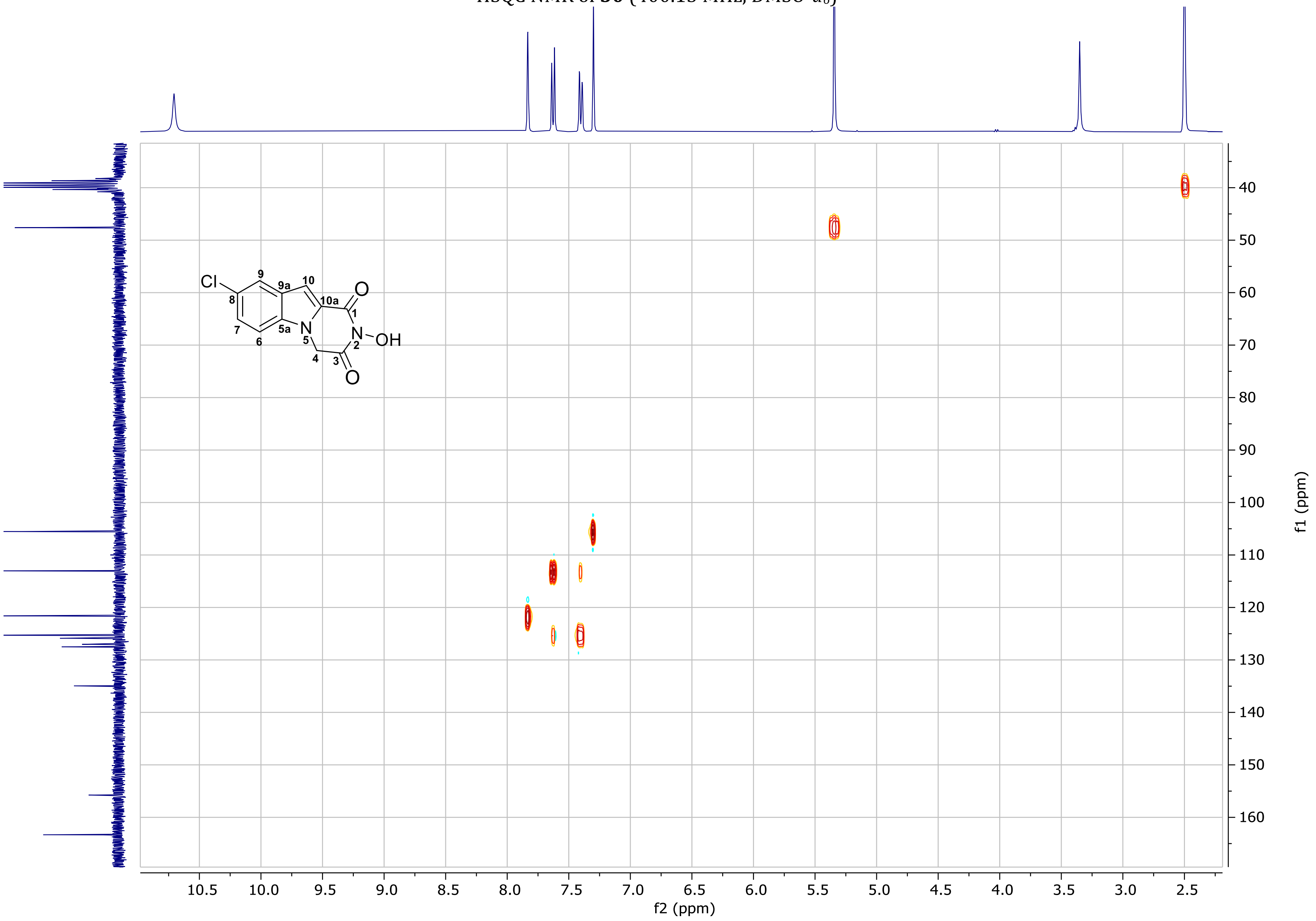

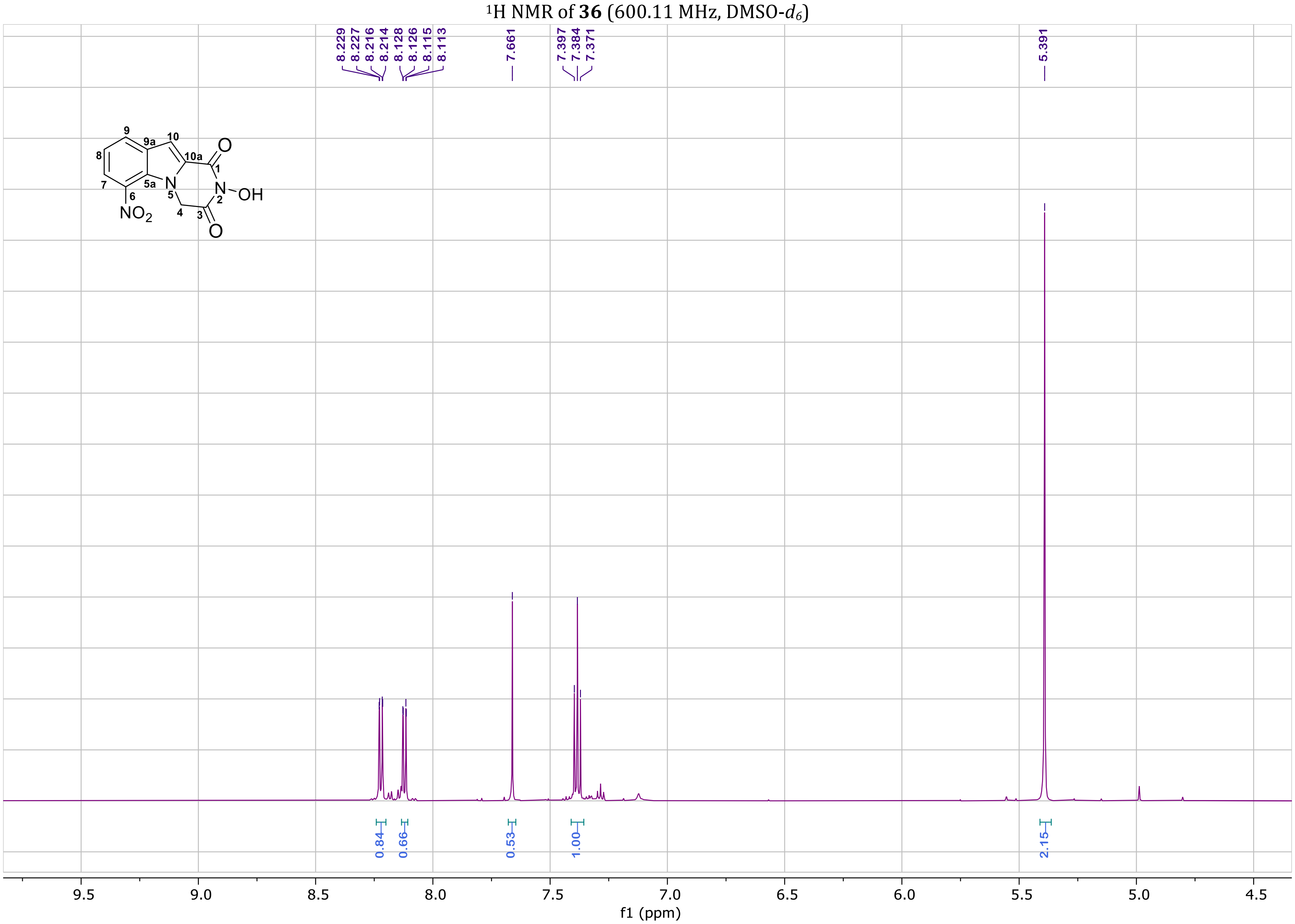

¹H NMR of **36** (600.11 MHz, DMSO-d_6)
8.229
8.227
8.216
8.214
8.128
8.126
8.115
8.113
7.661
7.397
7.384
7.371
5.391
9
9a
10
10a
8
7
6
5a
5
4
3
2
1
O
N
N-OH
NO2
O
0.84
0.66
0.53
1.00
2.15
9.5
9.0
8.5
8.0
7.5
7.0
6.5
6.0
5.5
5.0
4.5
f1 (ppm)

^{13}C NMR of **36** (50.32 MHz, DMSO-d_6)

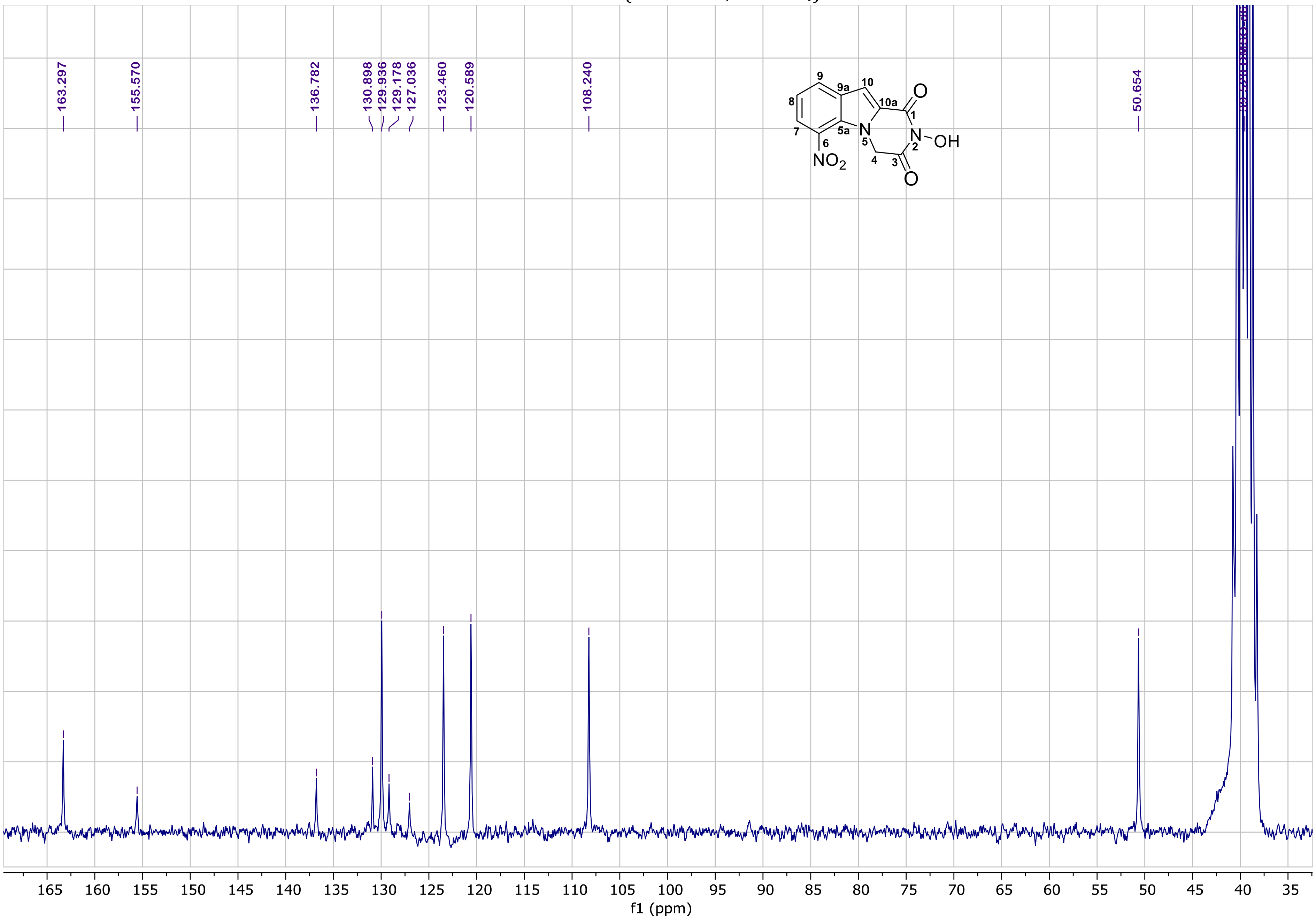

DEPT NMR of **36** (50.32 MHz, DMSO-d_6)

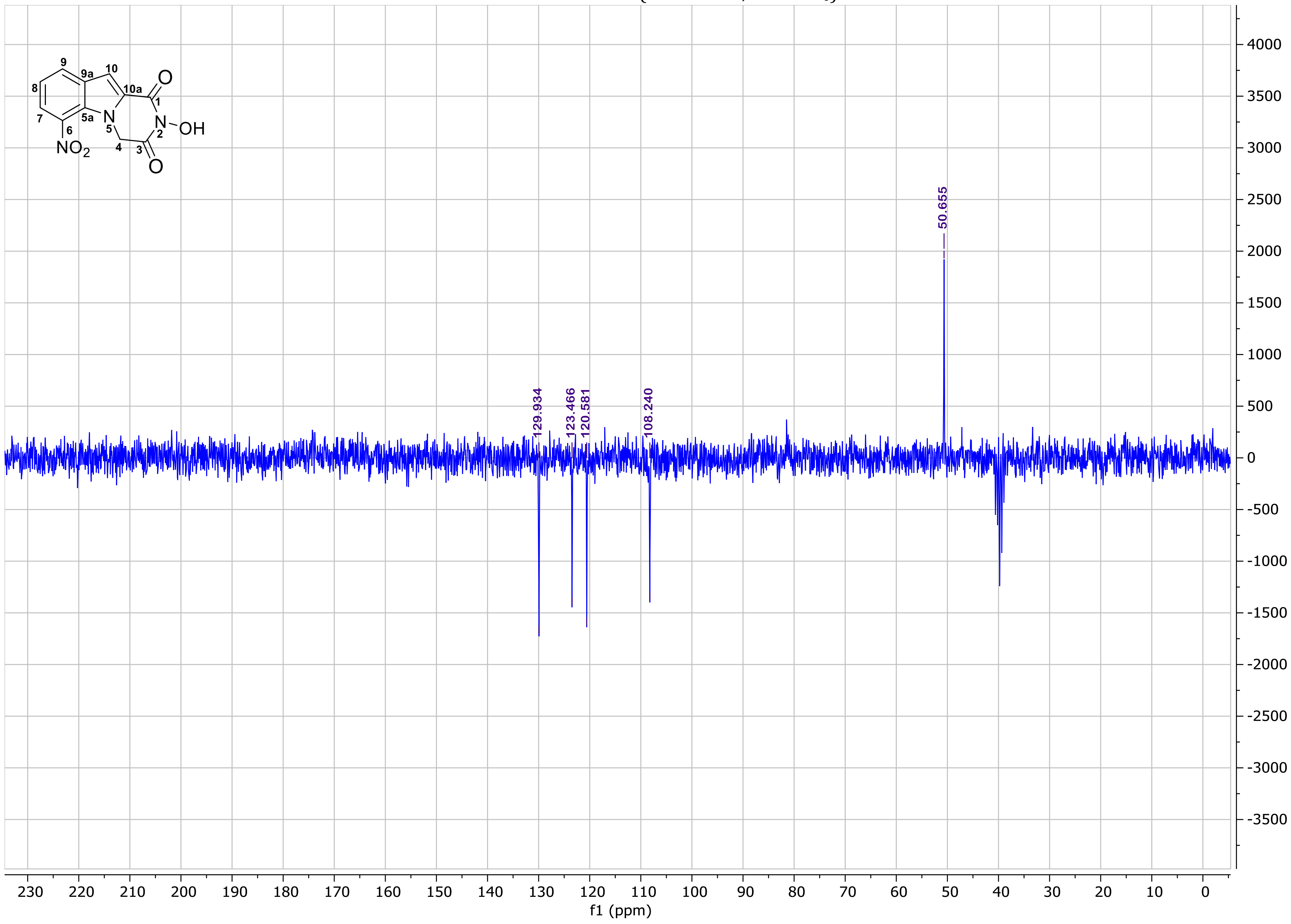

COSY NMR of **36** (600.11 MHz, DMSO-d_6)

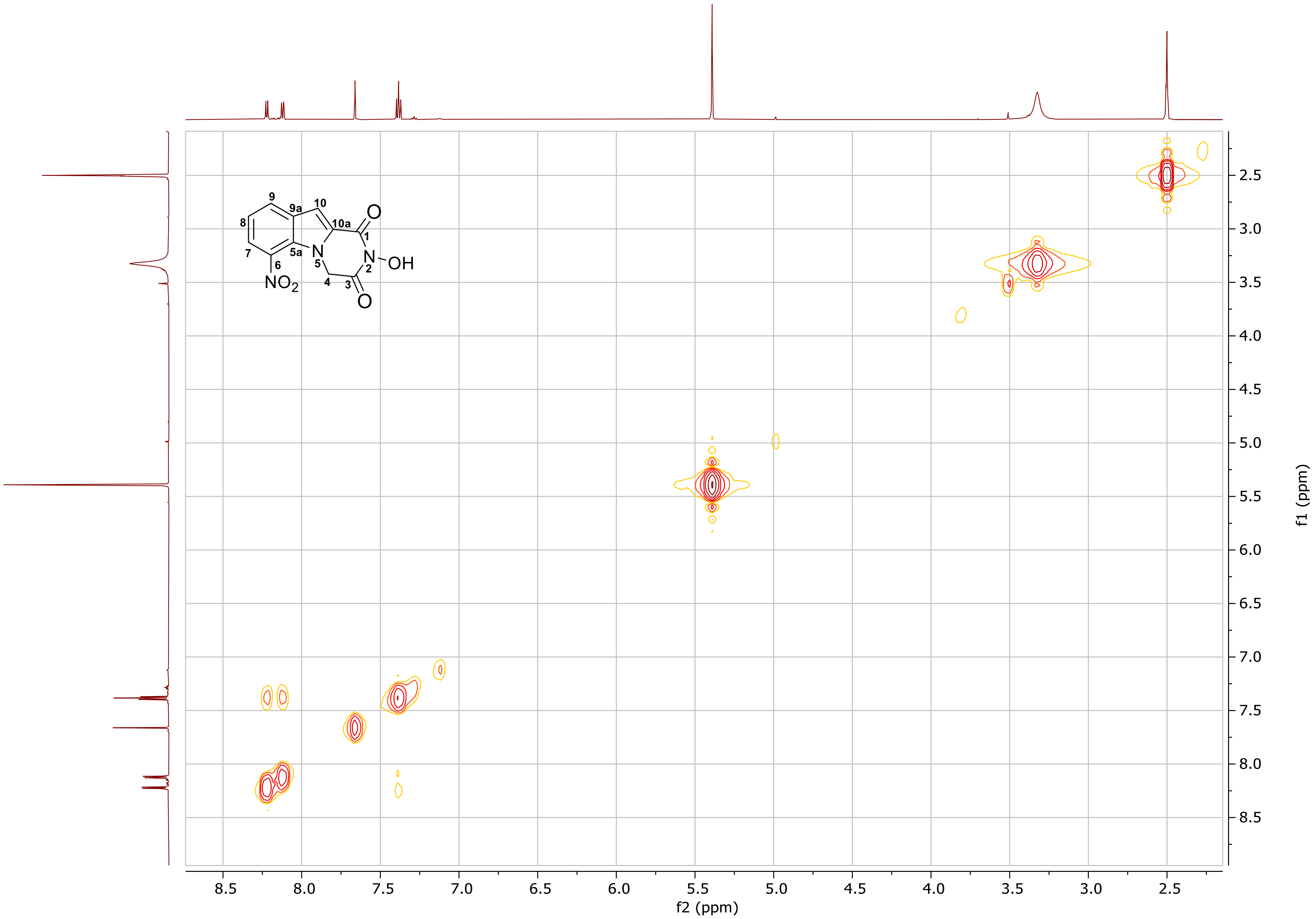

HSQC-DEPT NMR of **36** (600.11 MHz, DMSO-d_6)

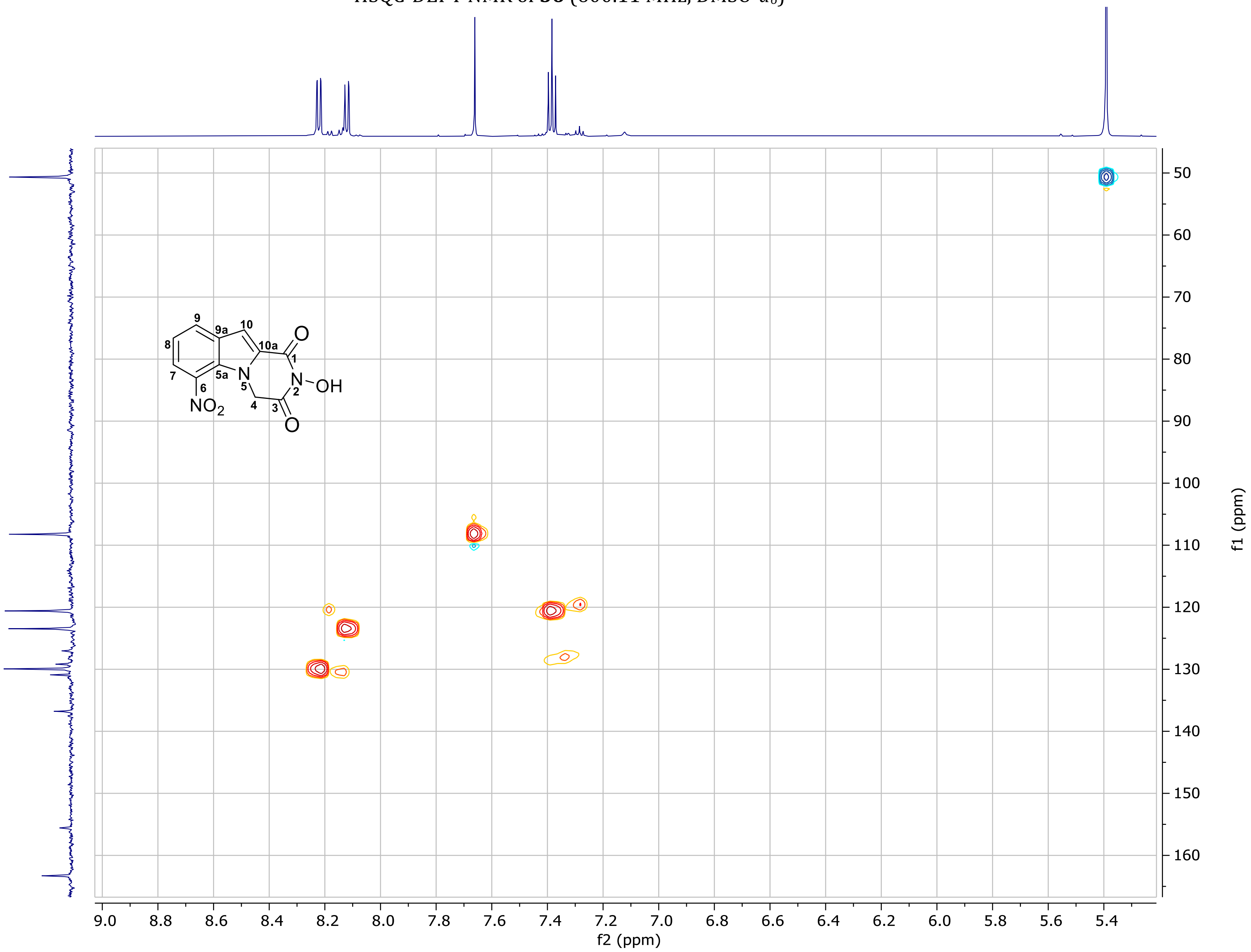

HMBC NMR of **36** (600.11 MHz, DMSO-d_6)

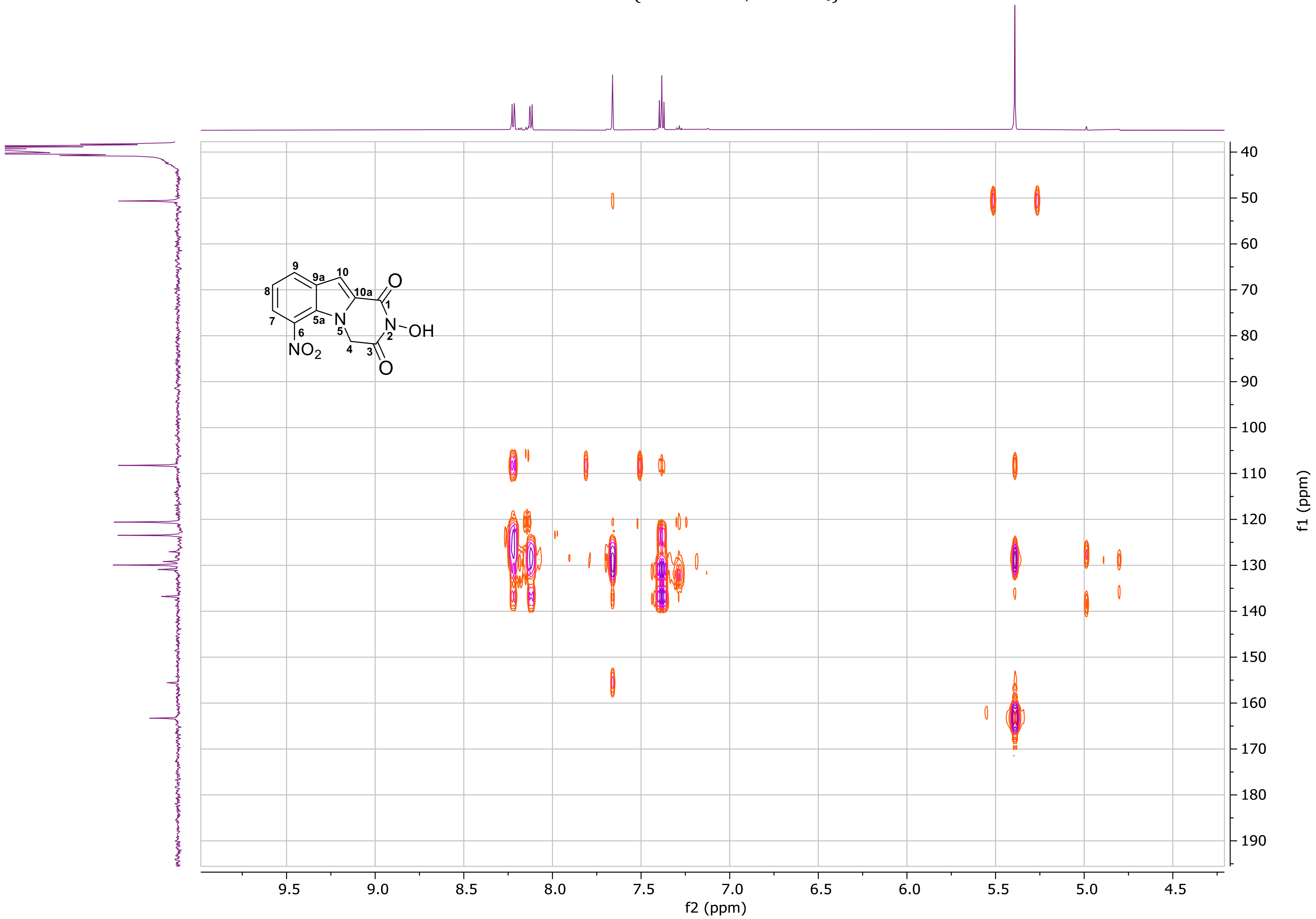

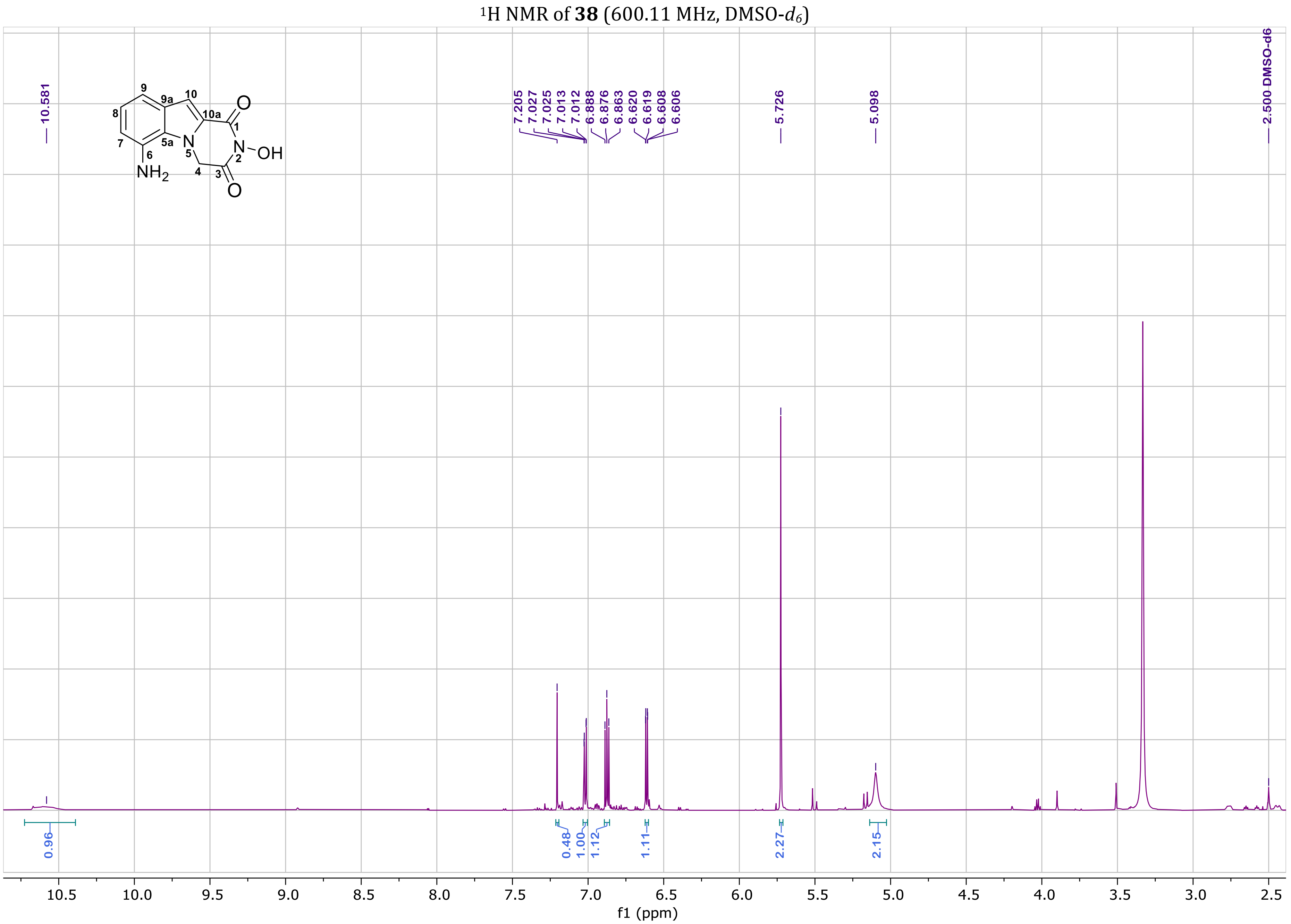

¹H NMR of 38 (600.11 MHz, DMSO-d6)
10.581
7.205
7.027
7.025
7.013
7.012
6.888
6.876
6.863
6.620
6.619
6.608
6.606
5.726
5.098
2.500 DMSO-d6
0.96
0.48
1.00
1.12
1.11
2.27
2.15
f1 (ppm)

^{13}C NMR of **38** (50.32 MHz, DMSO-d_6)

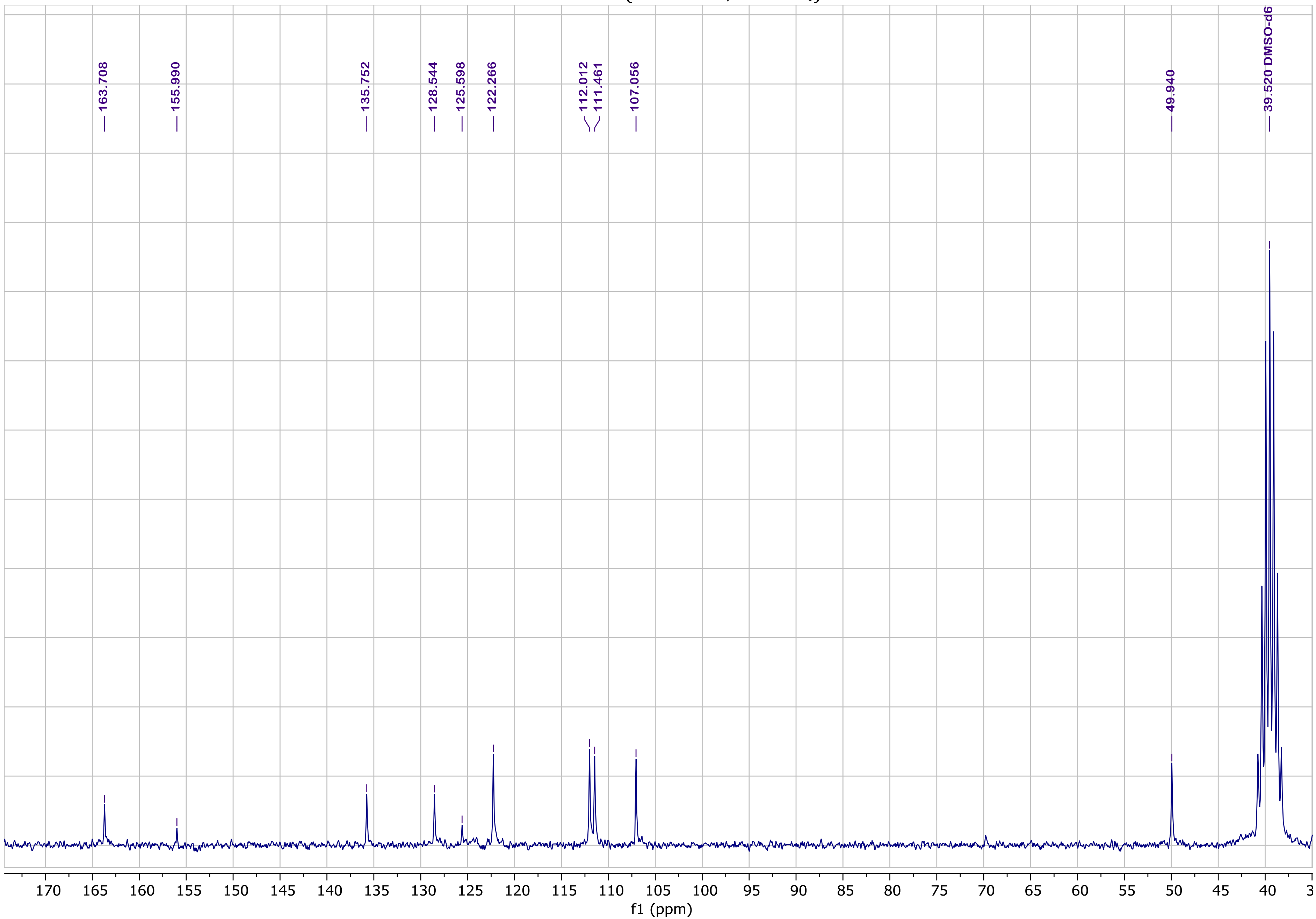

DEPT NMR of **38** (50.32 MHz, DMSO-d_6)

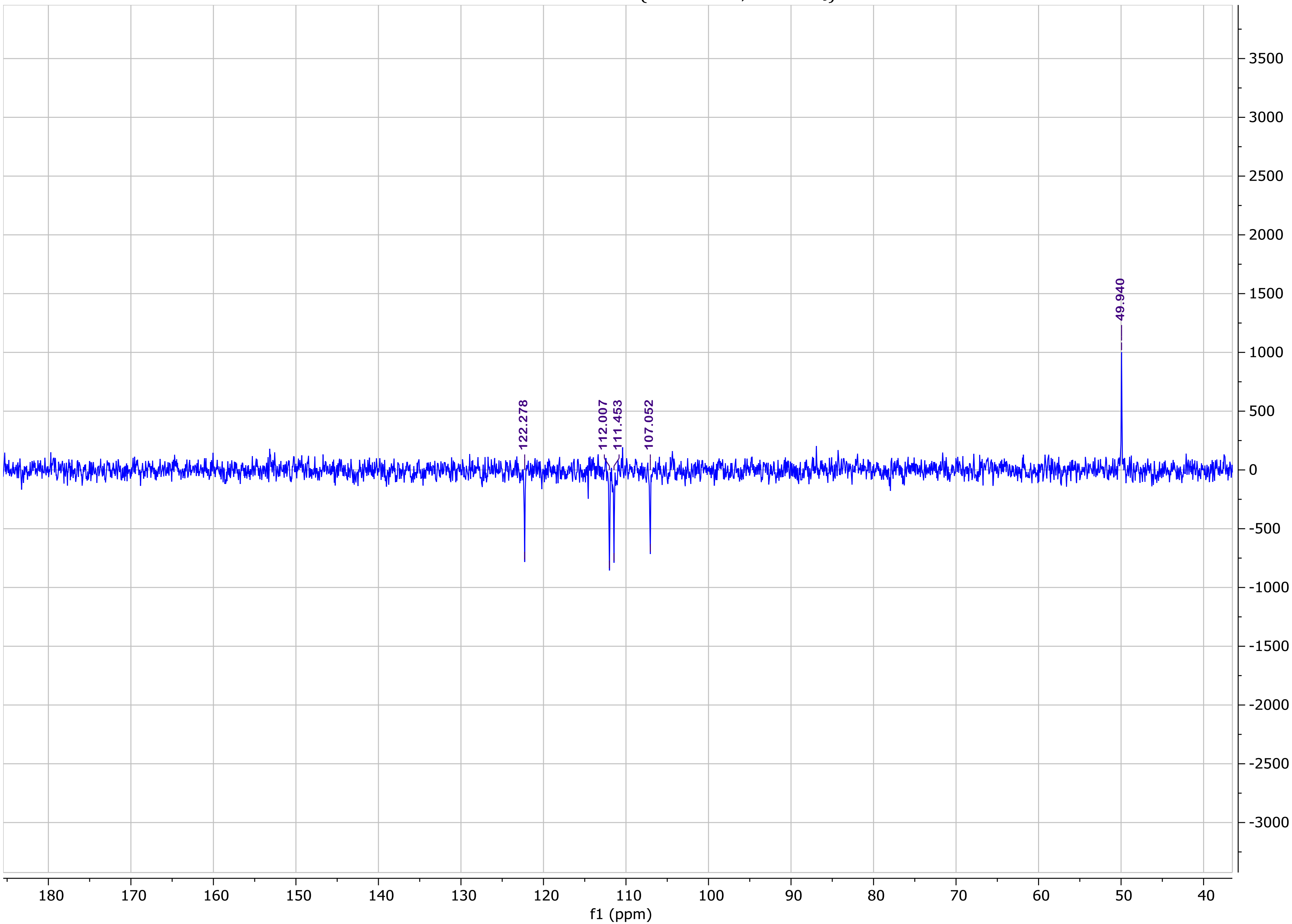

COSY NMR of **38** (400.13 MHz, DMSO-d_6)

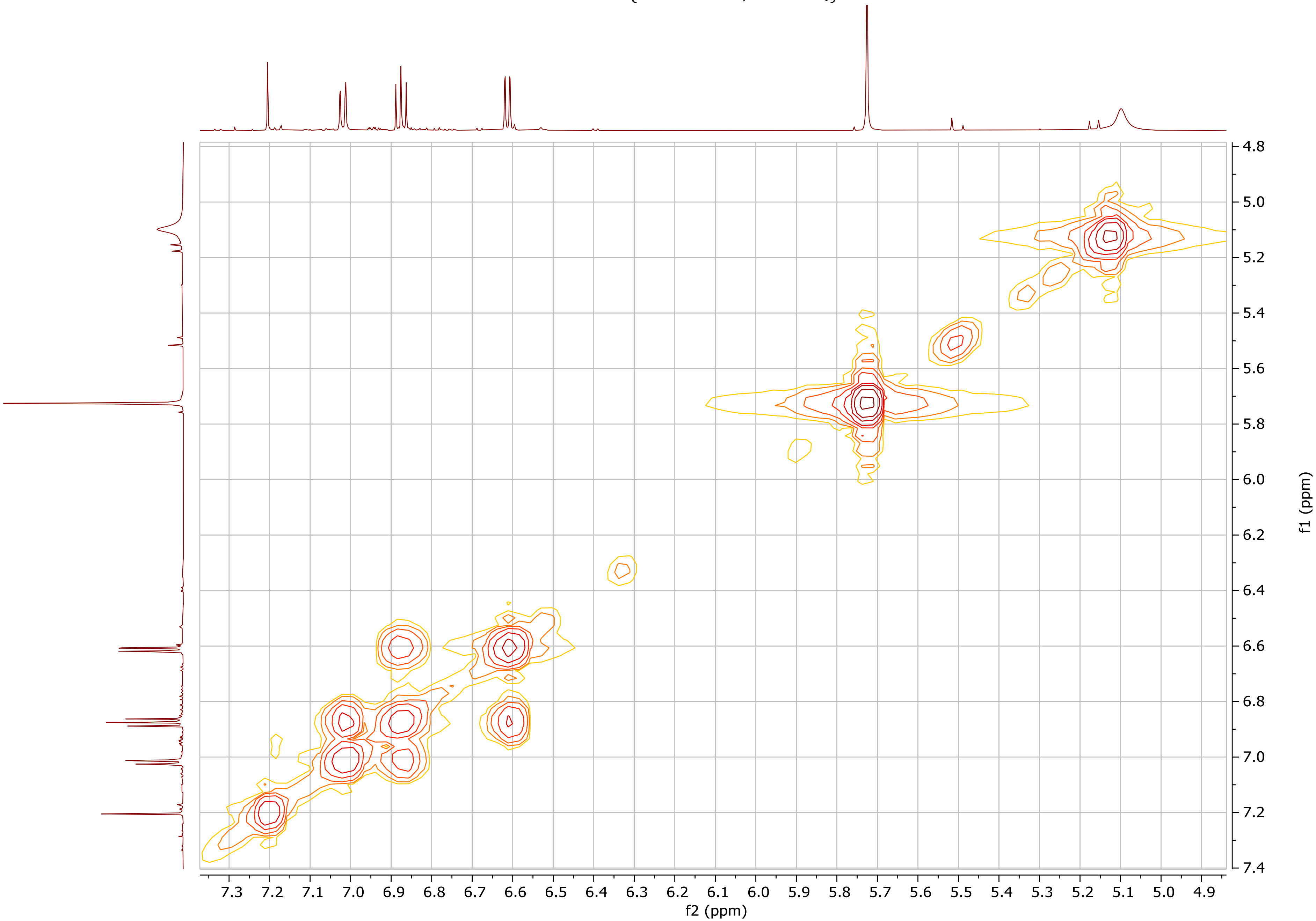

HSQC NMR of **38** (400.13 MHz, DMSO-d_6)

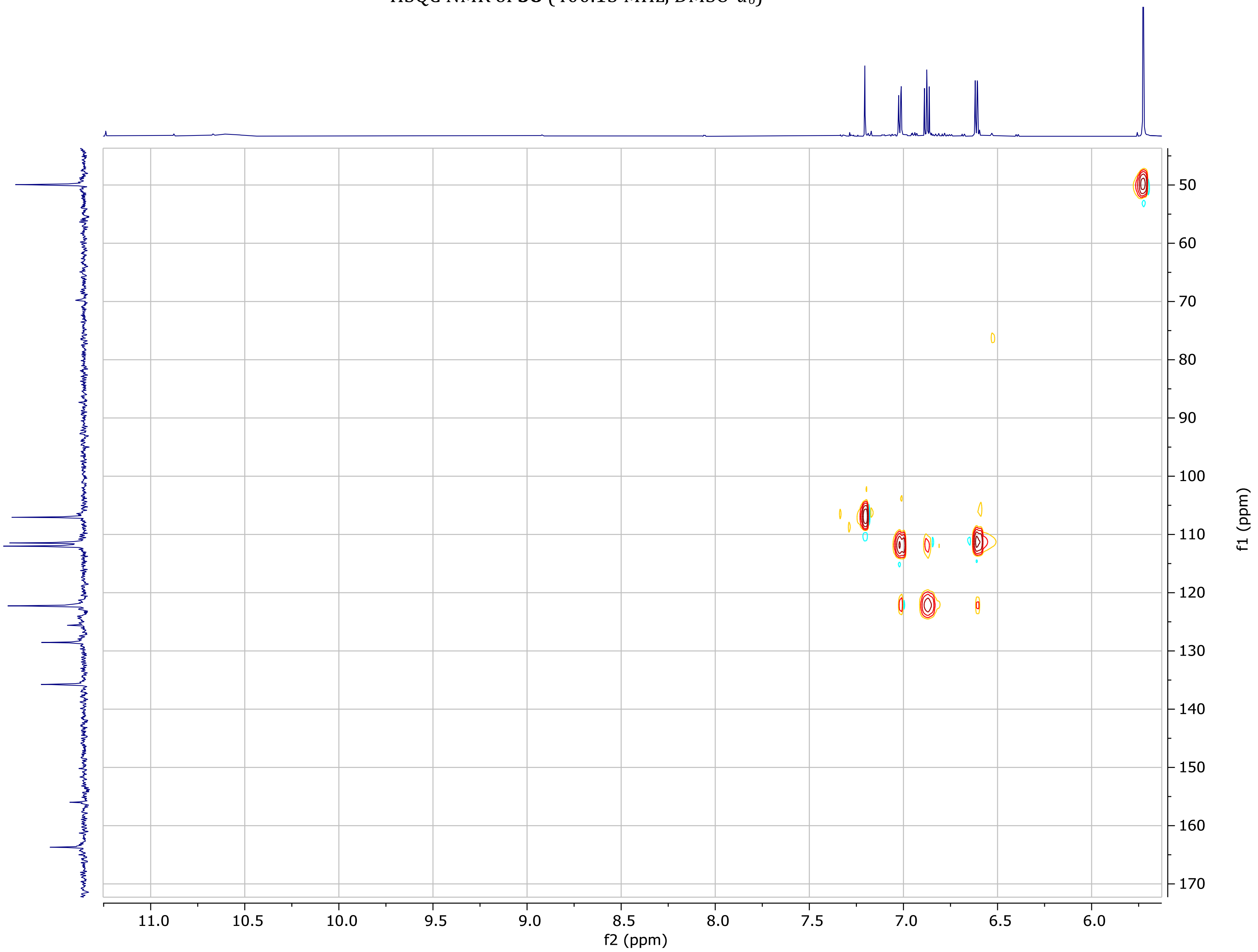

HMBC NMR of **38** (400.13 MHz, DMSO-d_6)

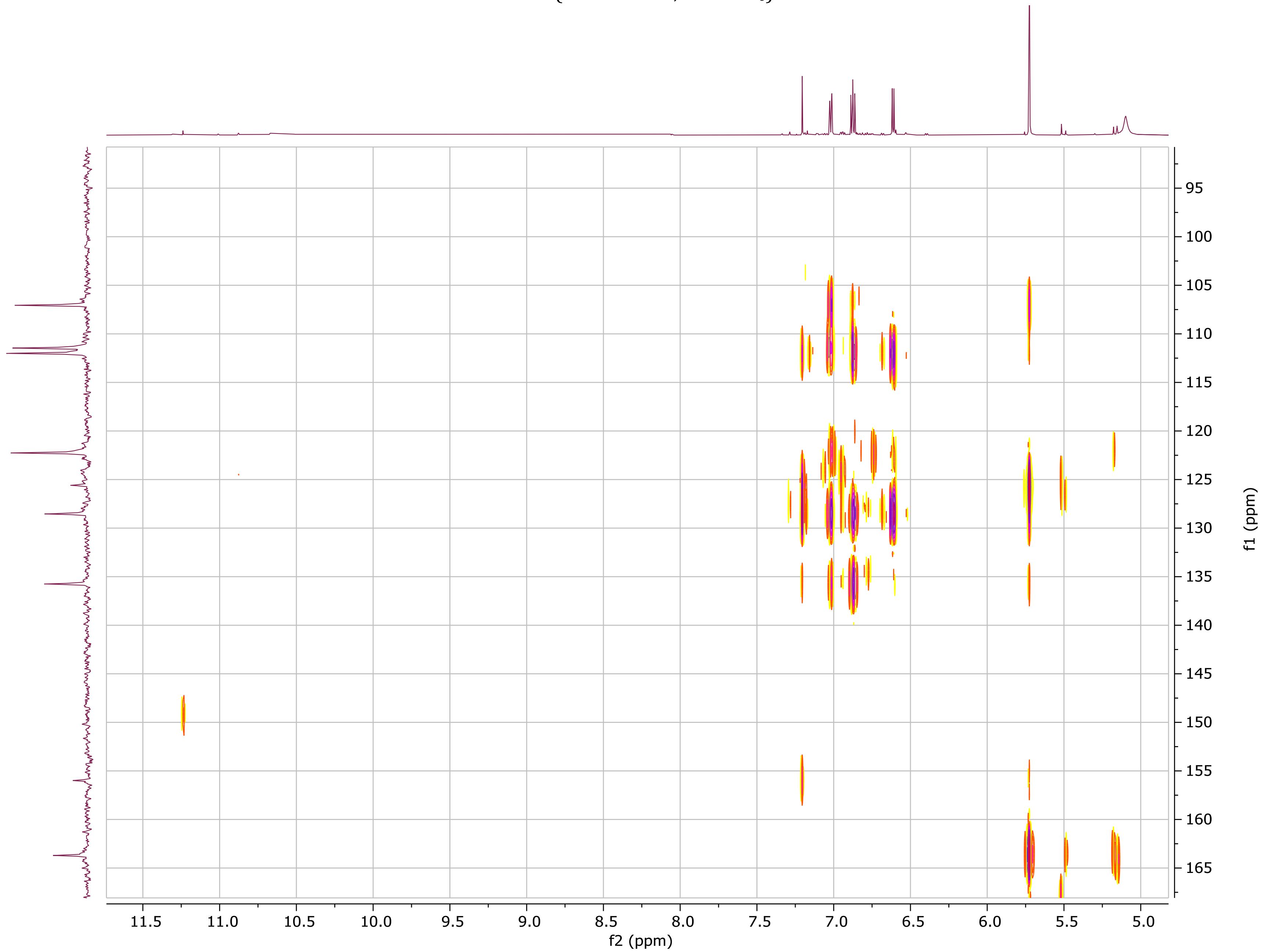

^{1}H NMR of **41** (400.13 MHz, $CDCl_3$)

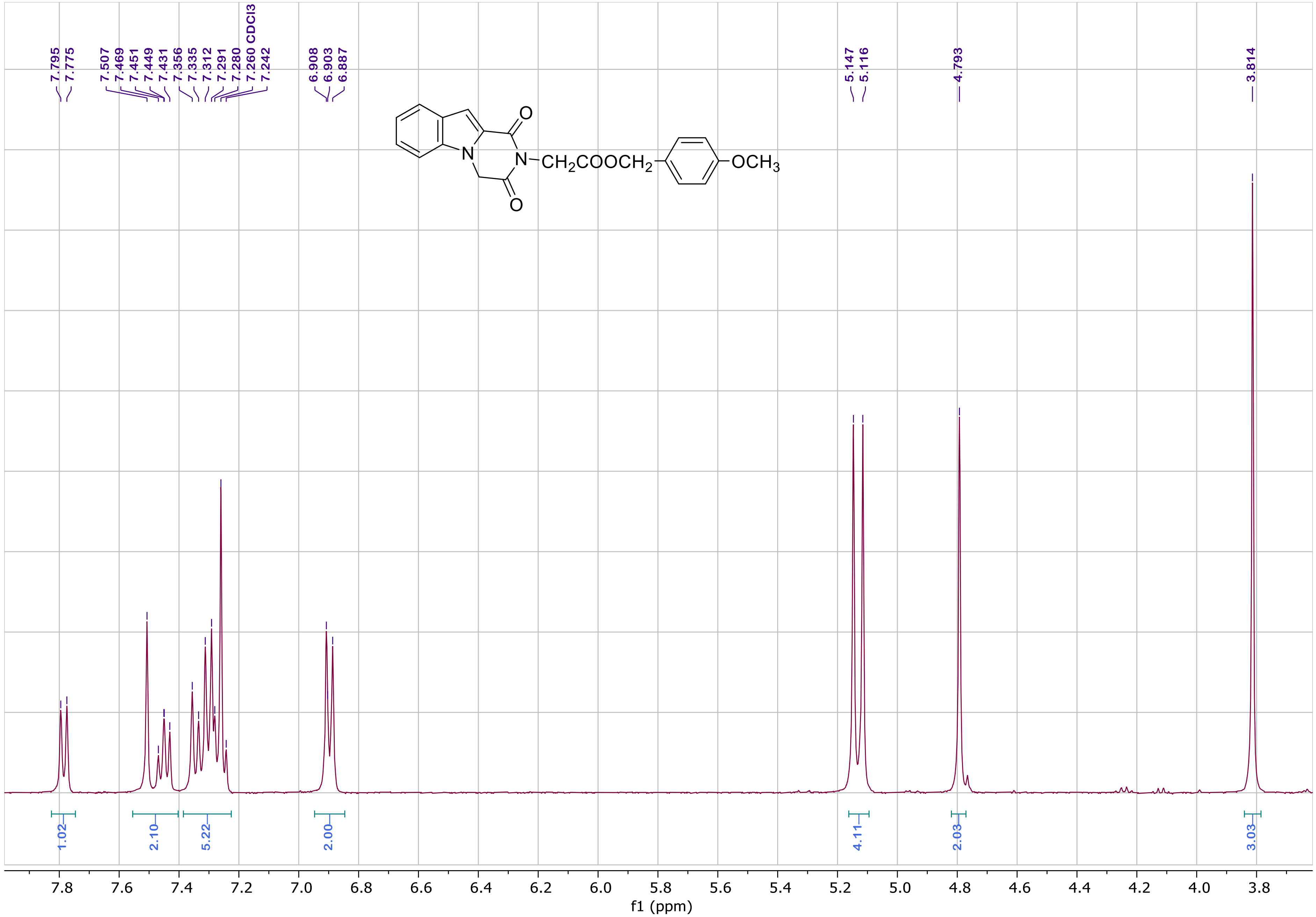

^{13}C NMR of **41** (50.32 MHz, $CDCl_3$)

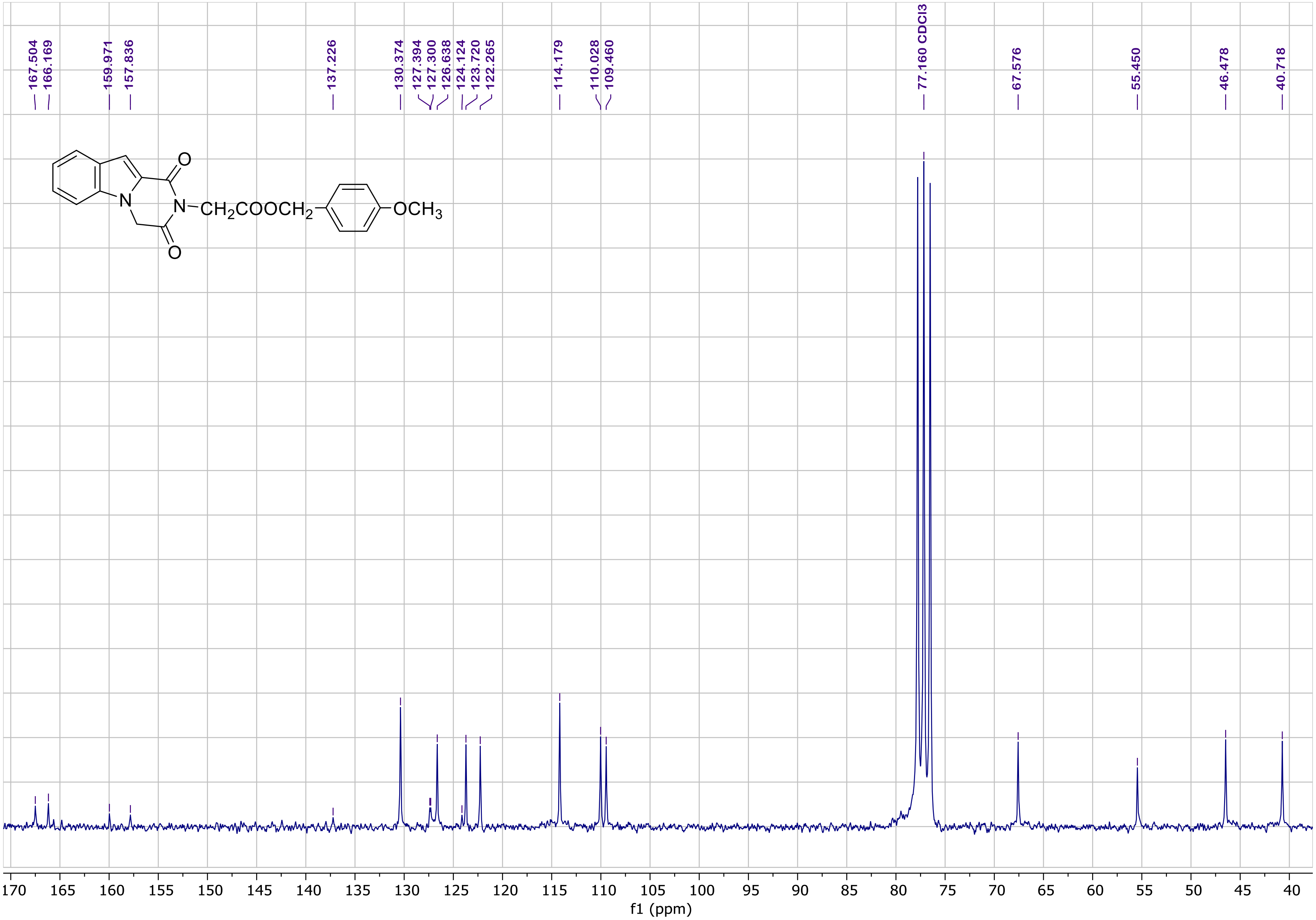

DEPT NMR of **41** (50.32 MHz, $CDCl_3$)

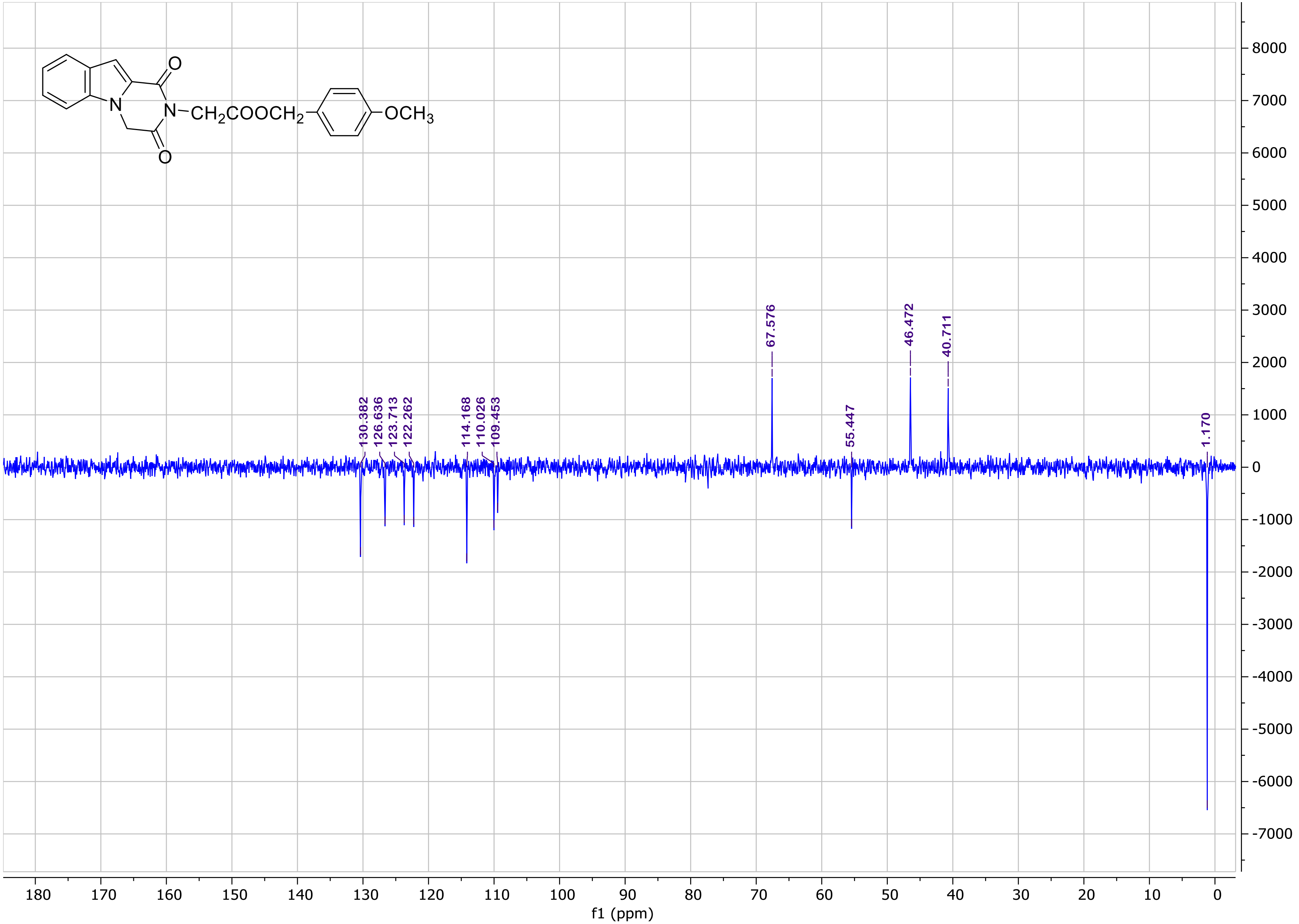

COSY NMR of **41** (400.13 MHz, CDCl3)

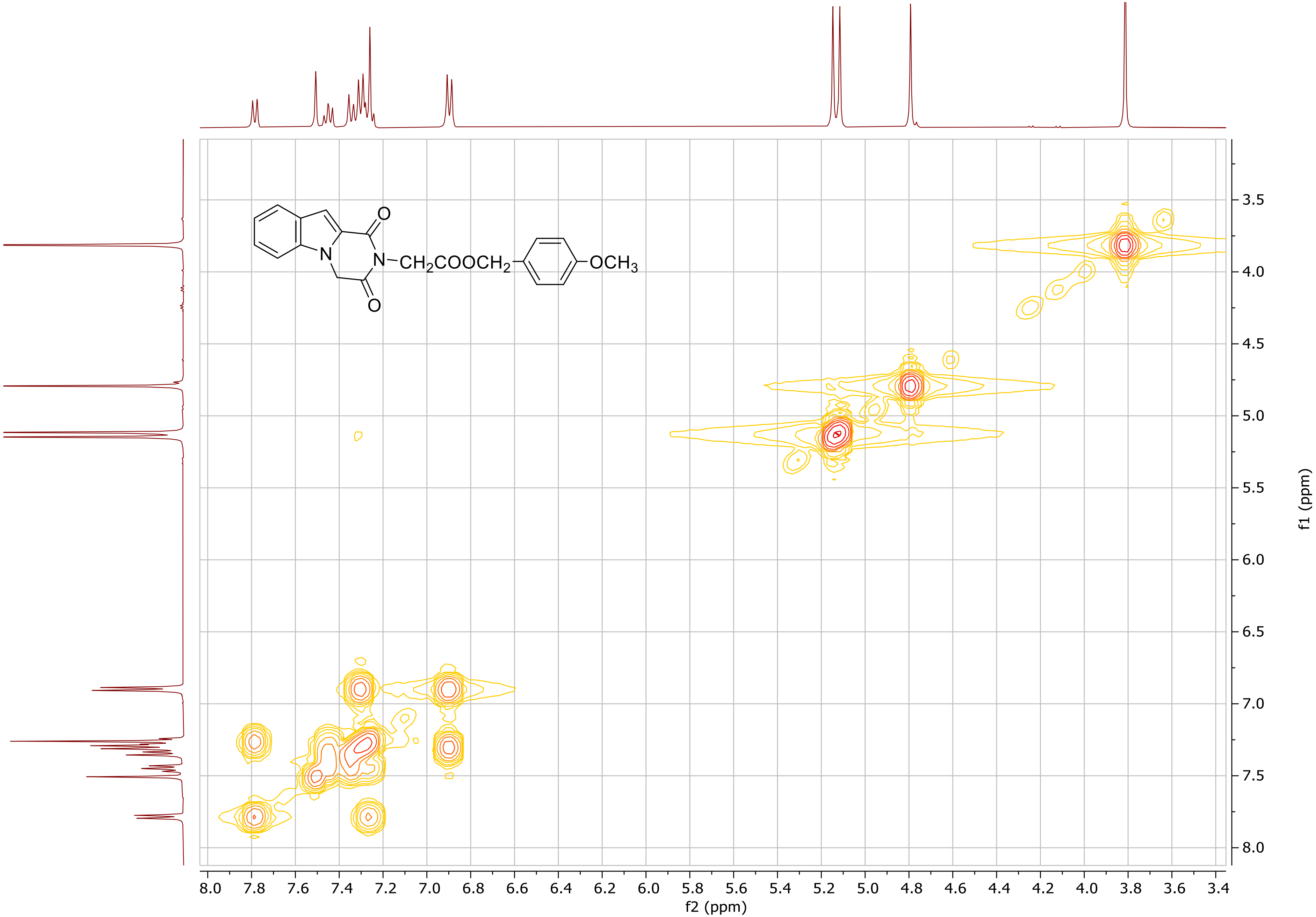

HSQC NMR of **41** (400.13 MHz, $CDCl_3$)

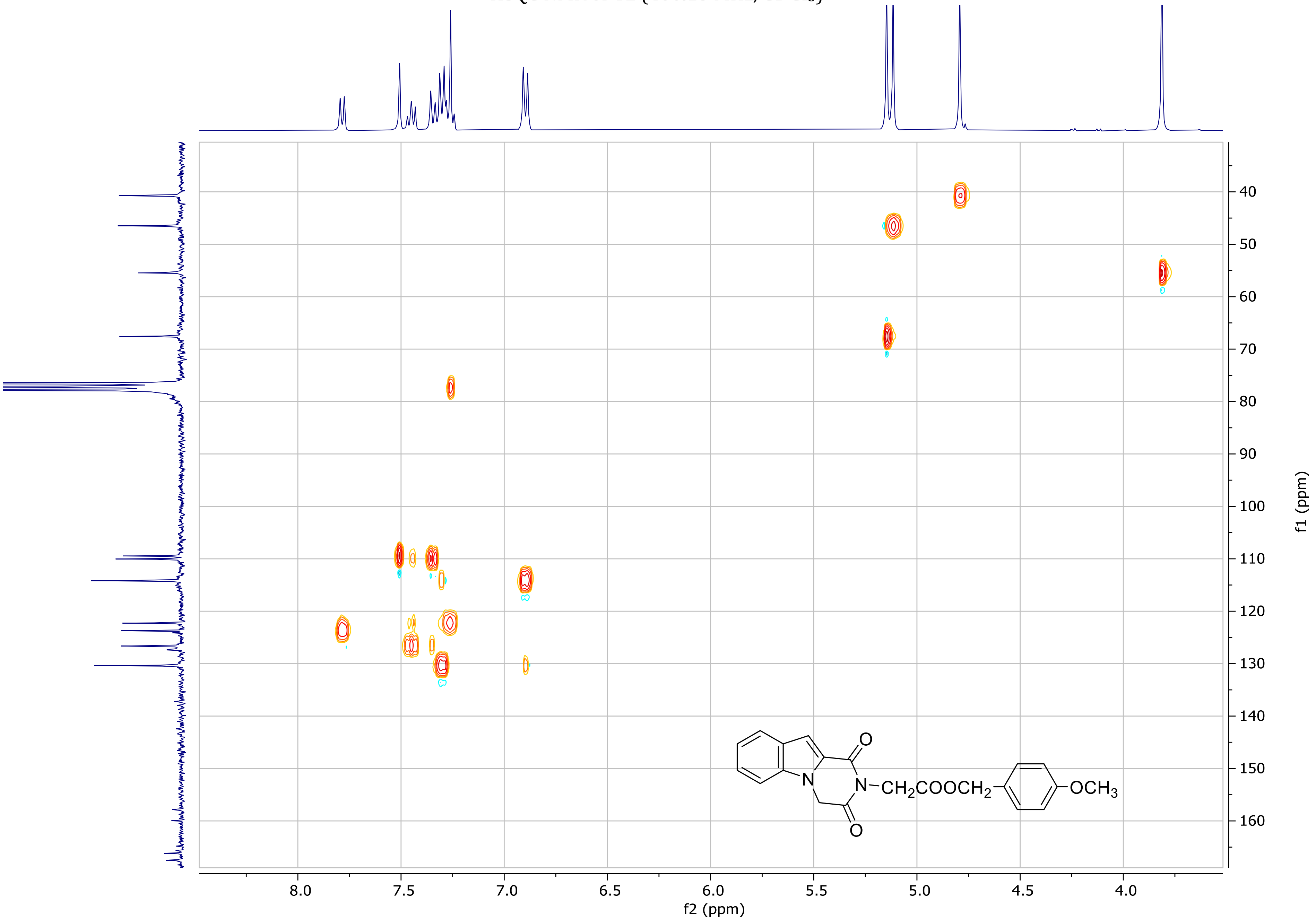

HMBC NMR of **41** (400.13 MHz, $CDCl_3$)

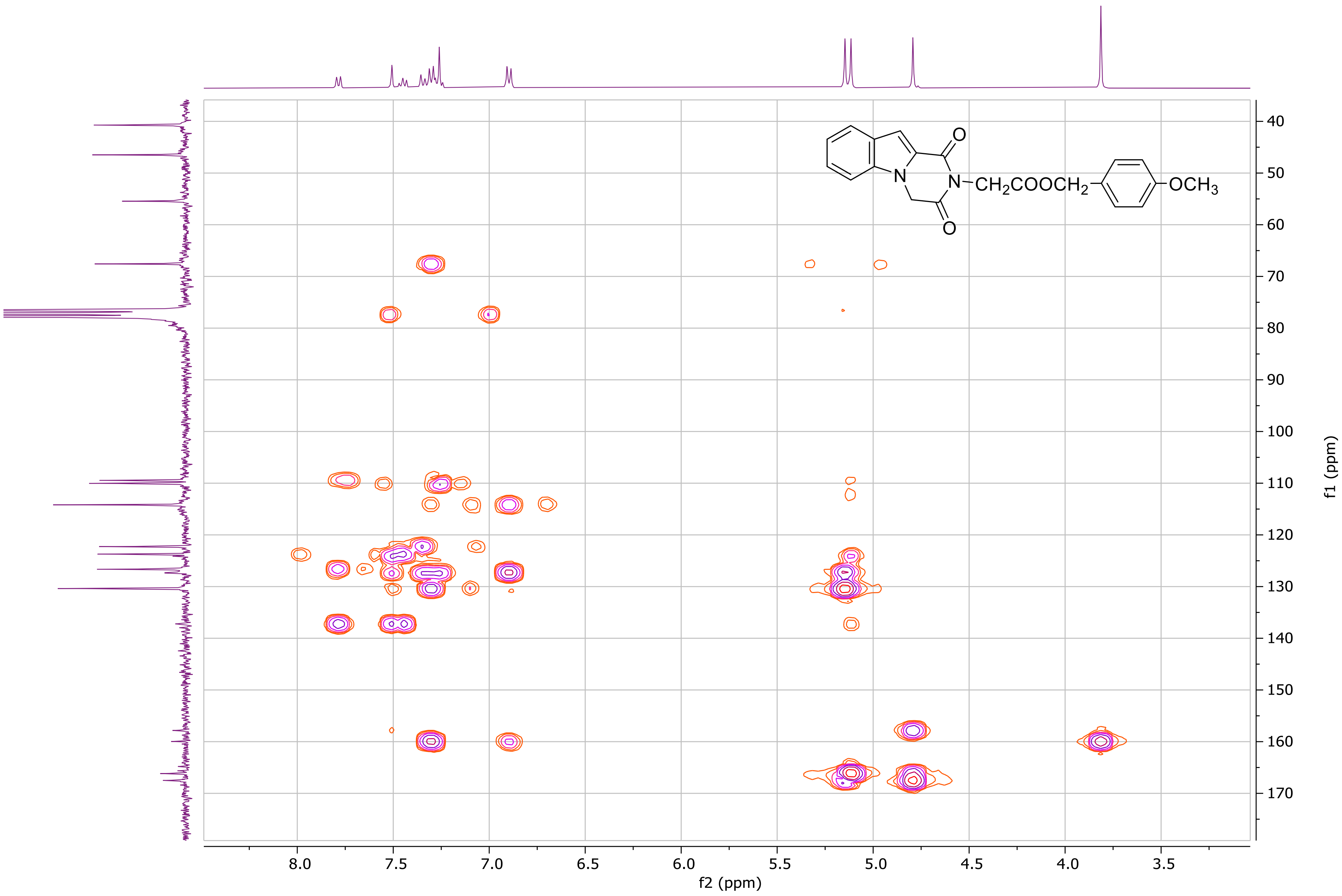

^{1}H NMR of **41a** (600.11 MHz, $CDCl_3$)

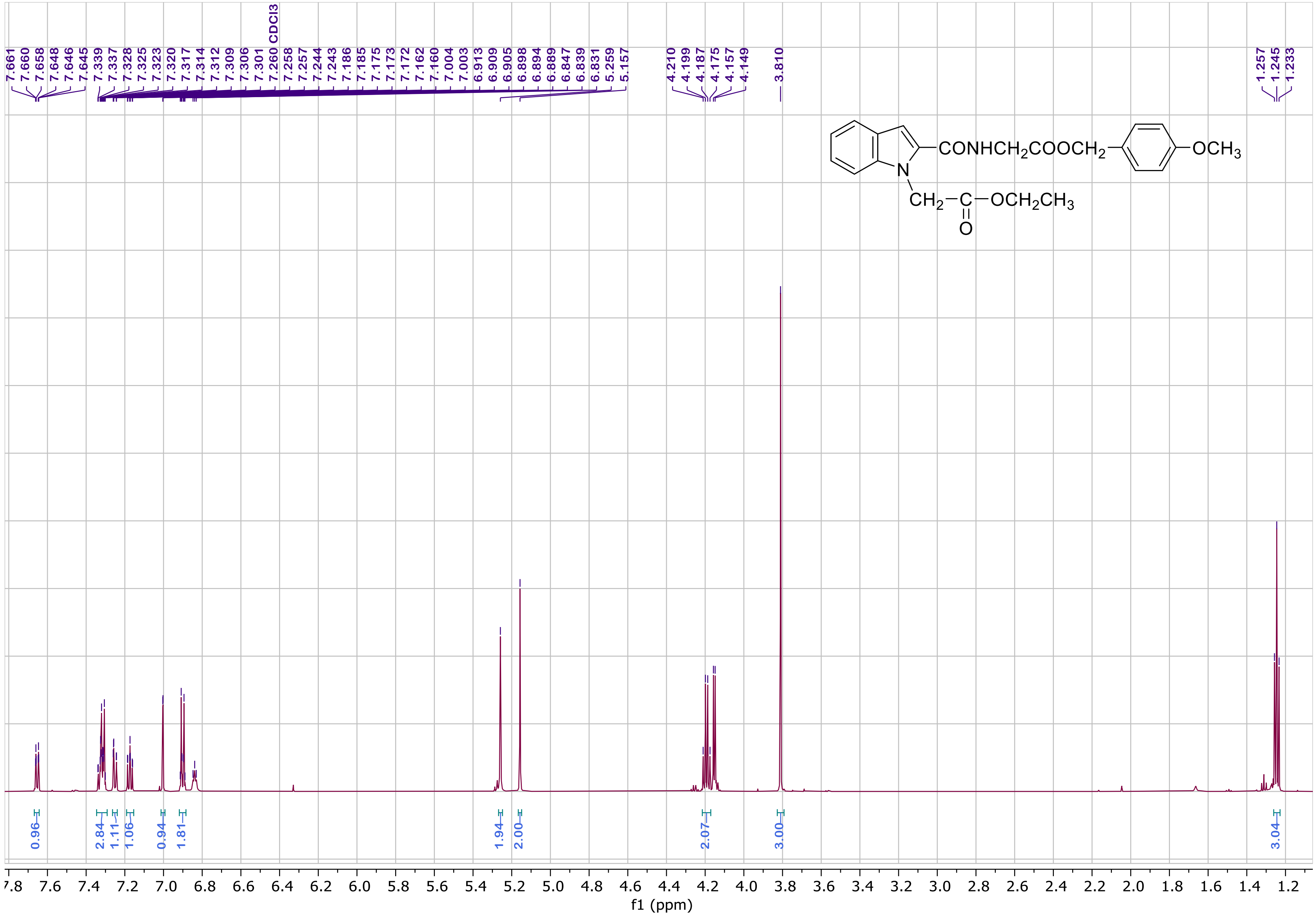

^{13}C NMR of **41a** (50.32 MHz, $CDCl_3$)

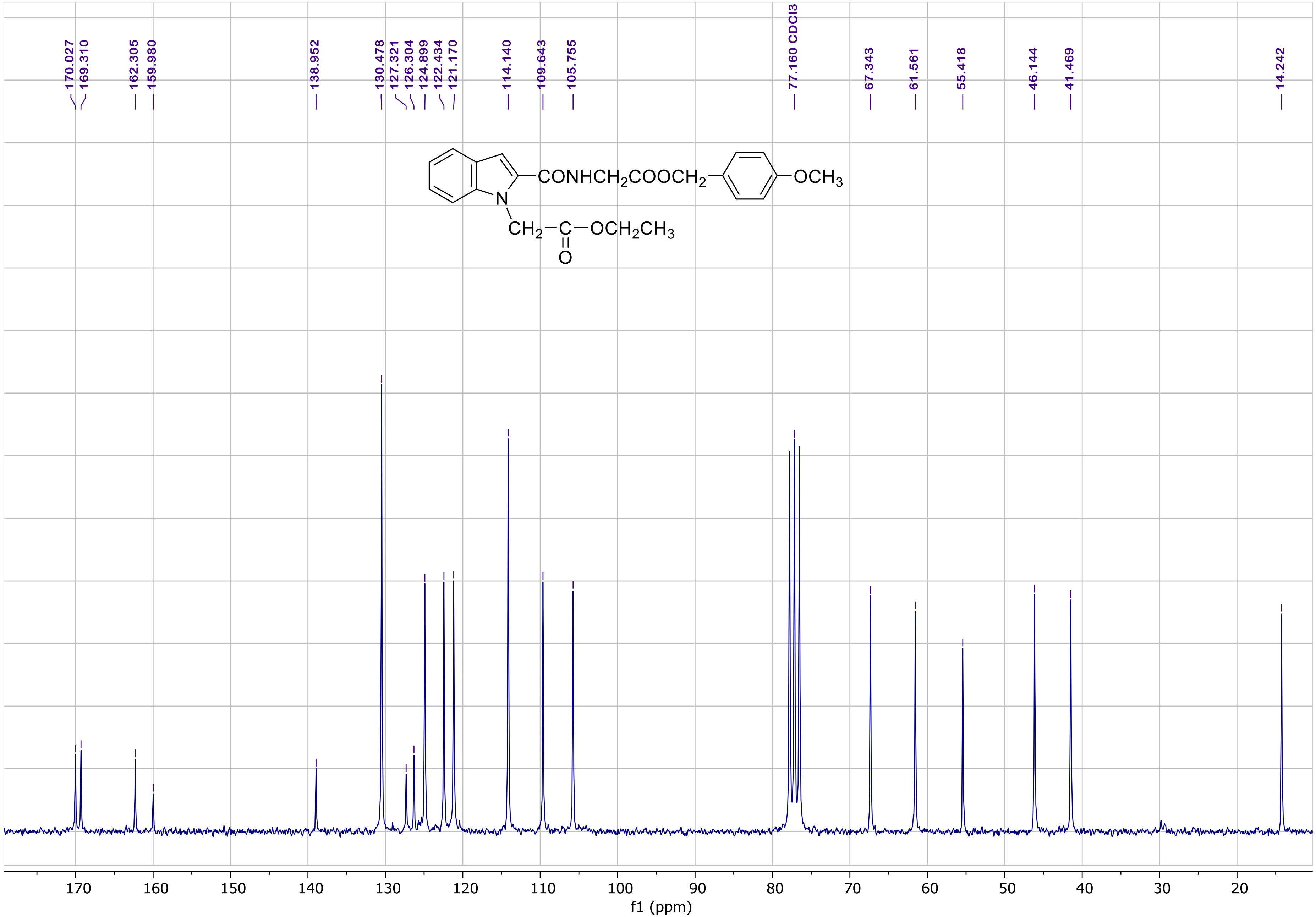

DEPT NMR of **41a** (50.32 MHz, $CDCl_3$)

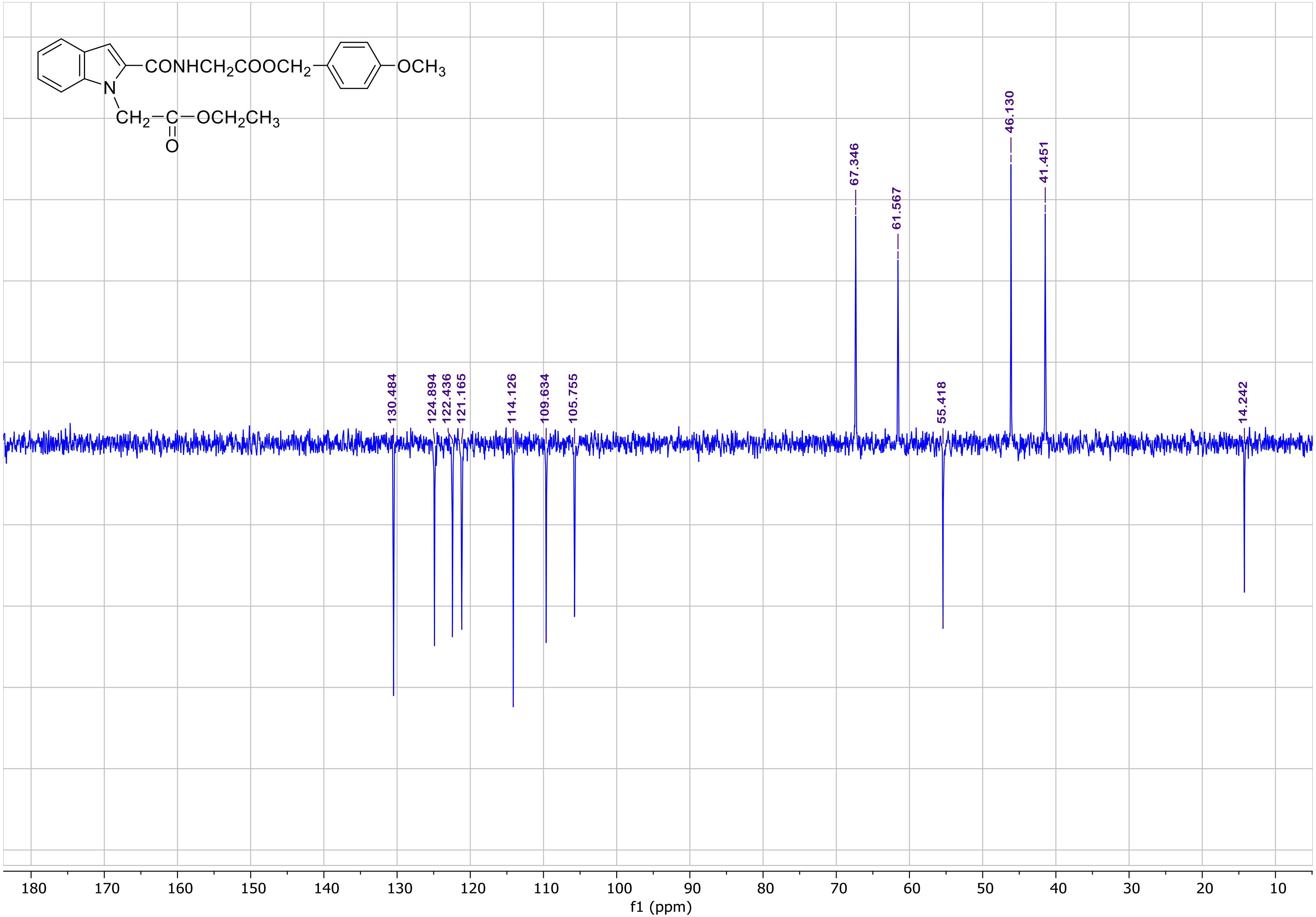

COSY NMR of **41a** (400.13 MHz, $CDCl_3$)

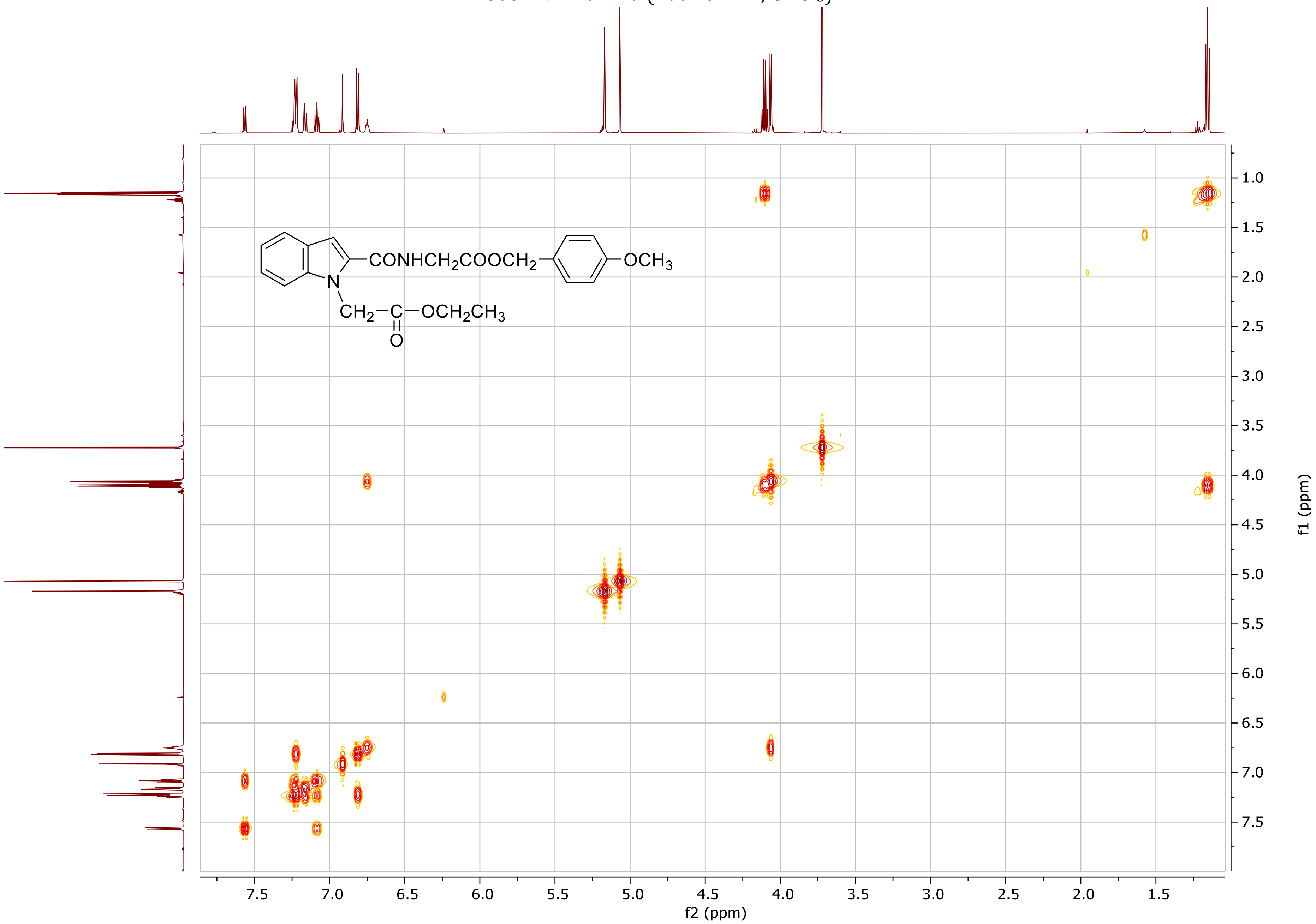

HSQC-DEPT NMR of **41a** (600.11 MHz, $CDCl_3$)

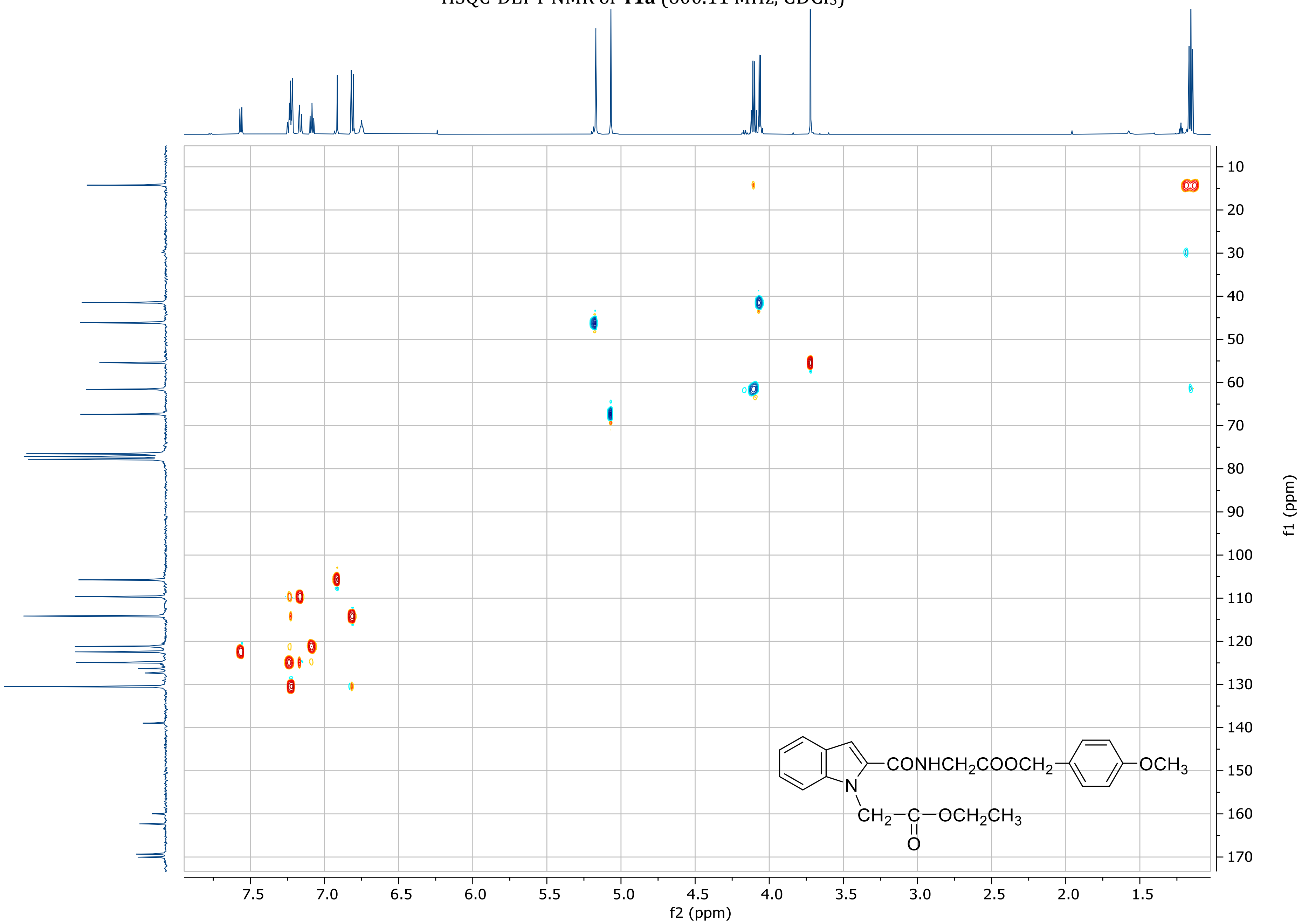

HMBC NMR of **41a** (400.13 MHz, $CDCl_3$)

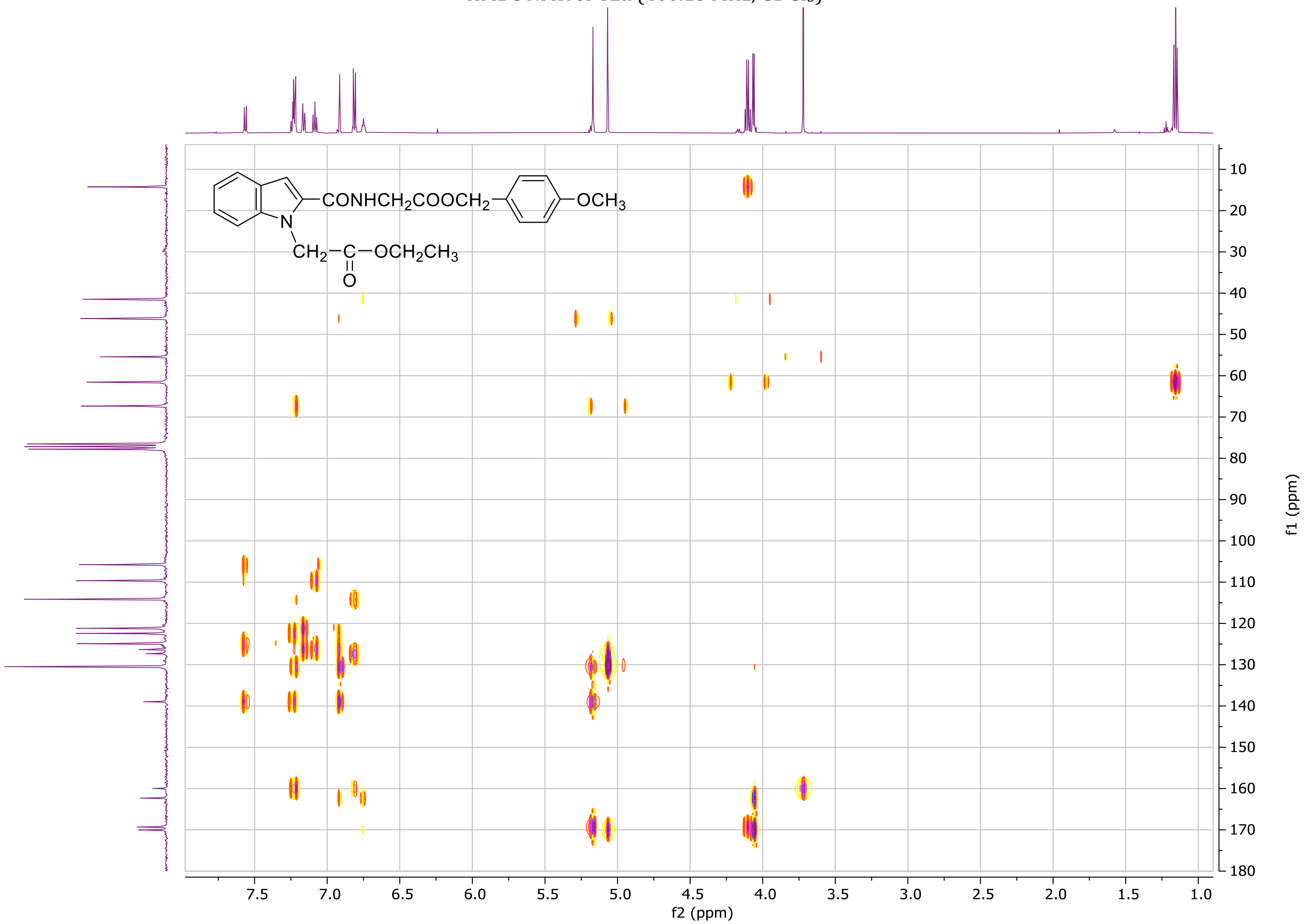

^{1}H NMR of **45** (400.13 MHz, $CDCl_3$)

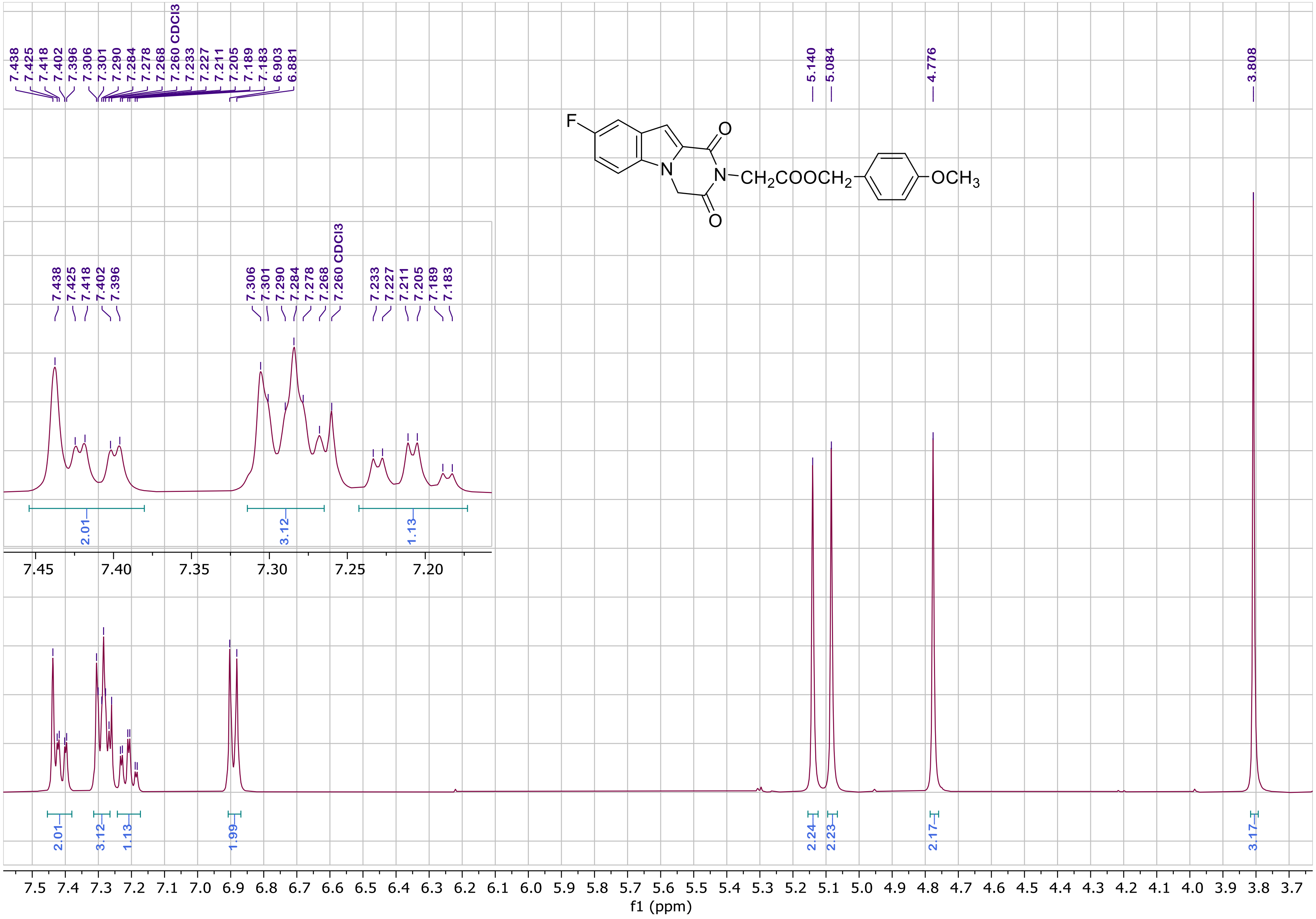

^{13}C NMR of **45** (50.32 MHz, $CDCl_3$)

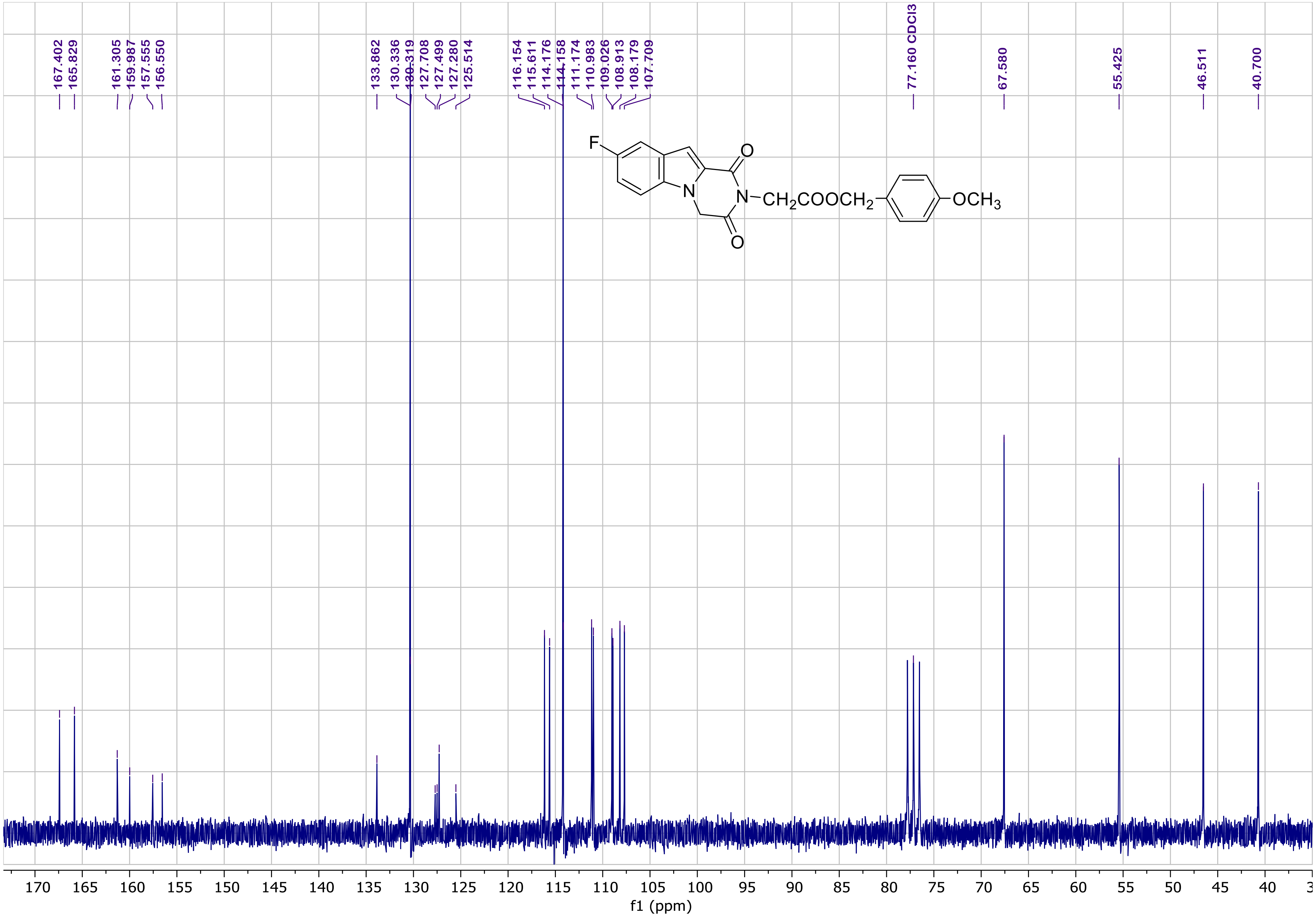

COSY NMR of **45** (400.13 MHz, $CDCl_3$)

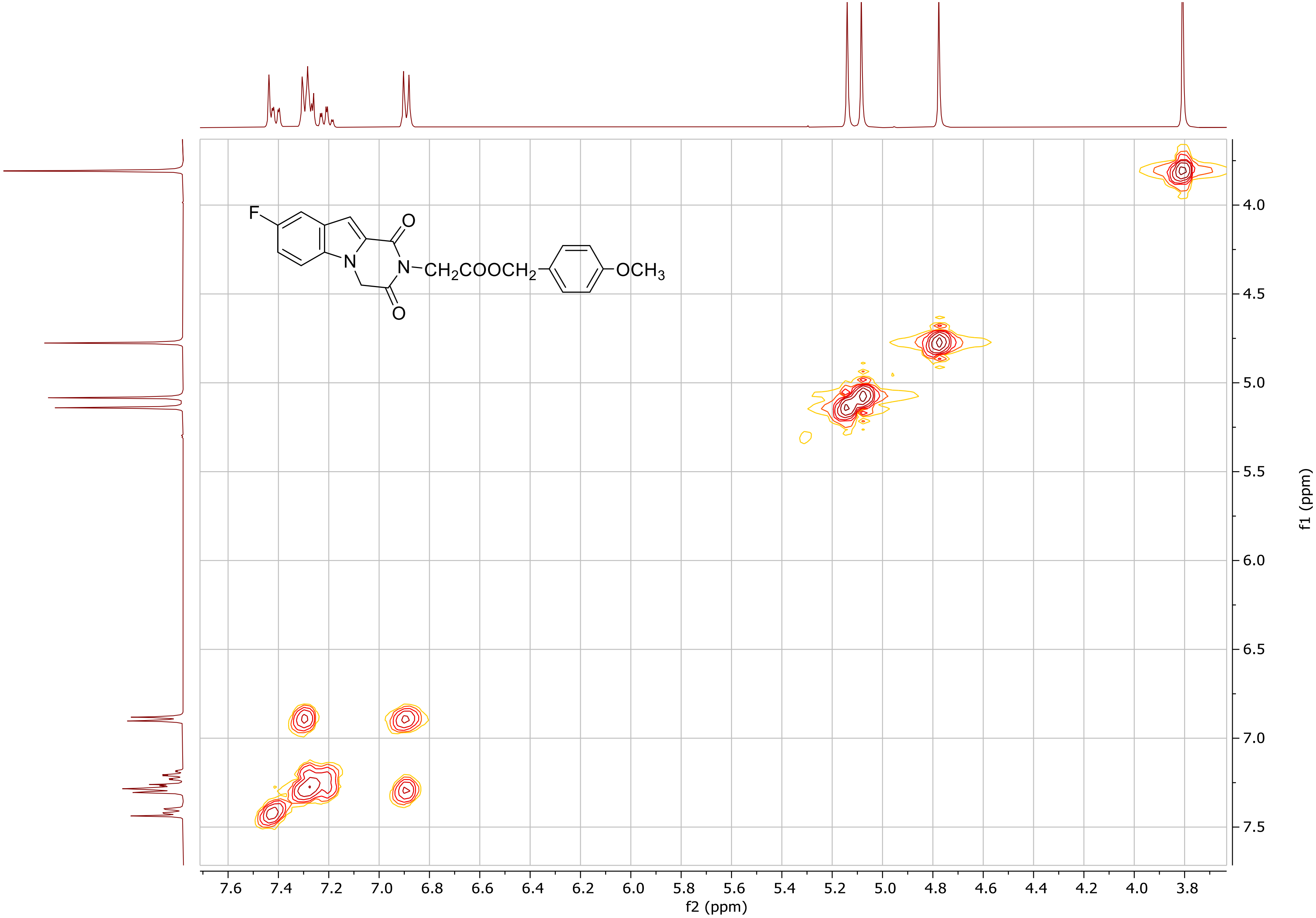

HSQC NMR of **45** (400.13 MHz, $CDCl_3$)

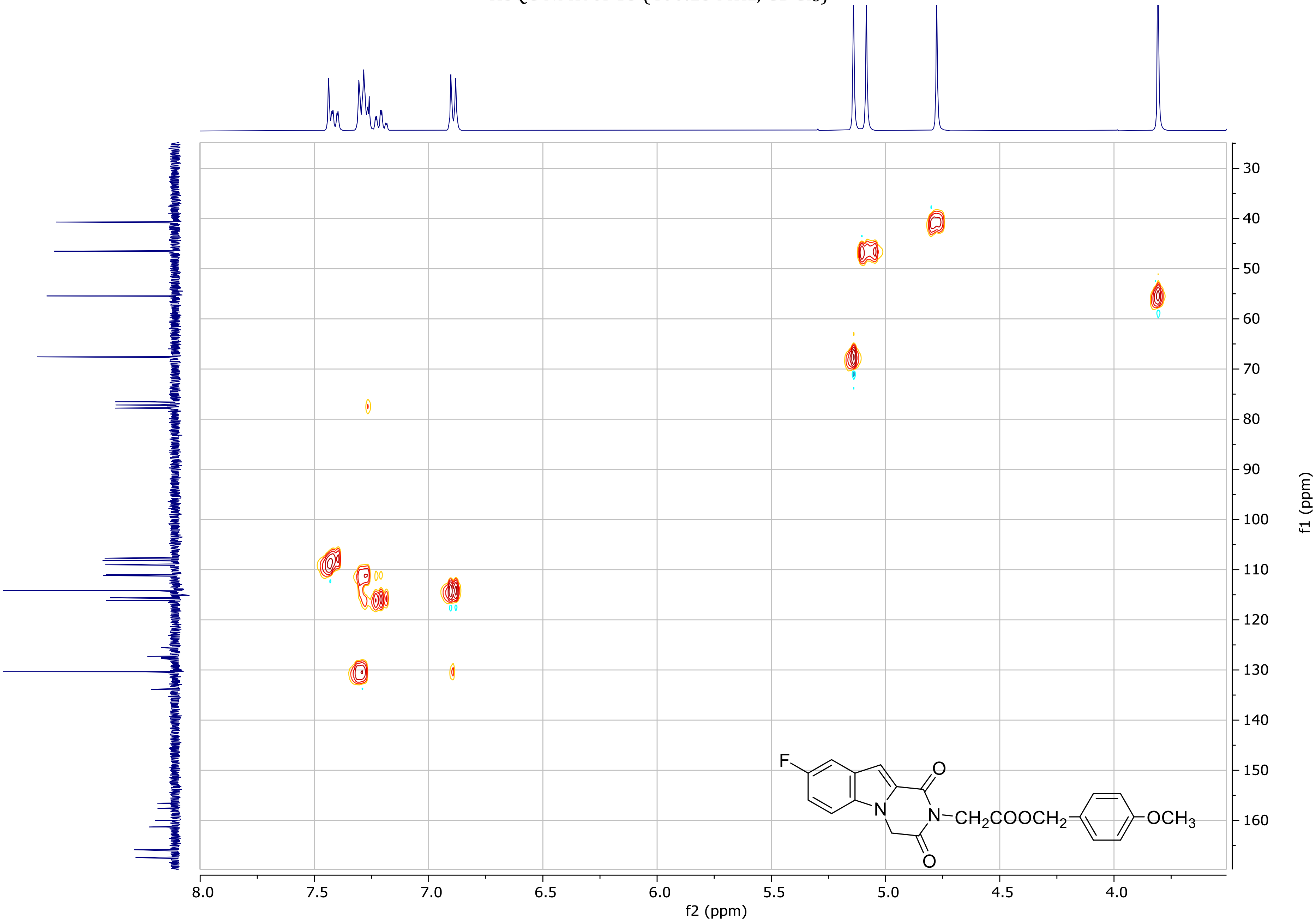

^{1}H NMR of **49** (400.13 MHz, $CDCl_3$)

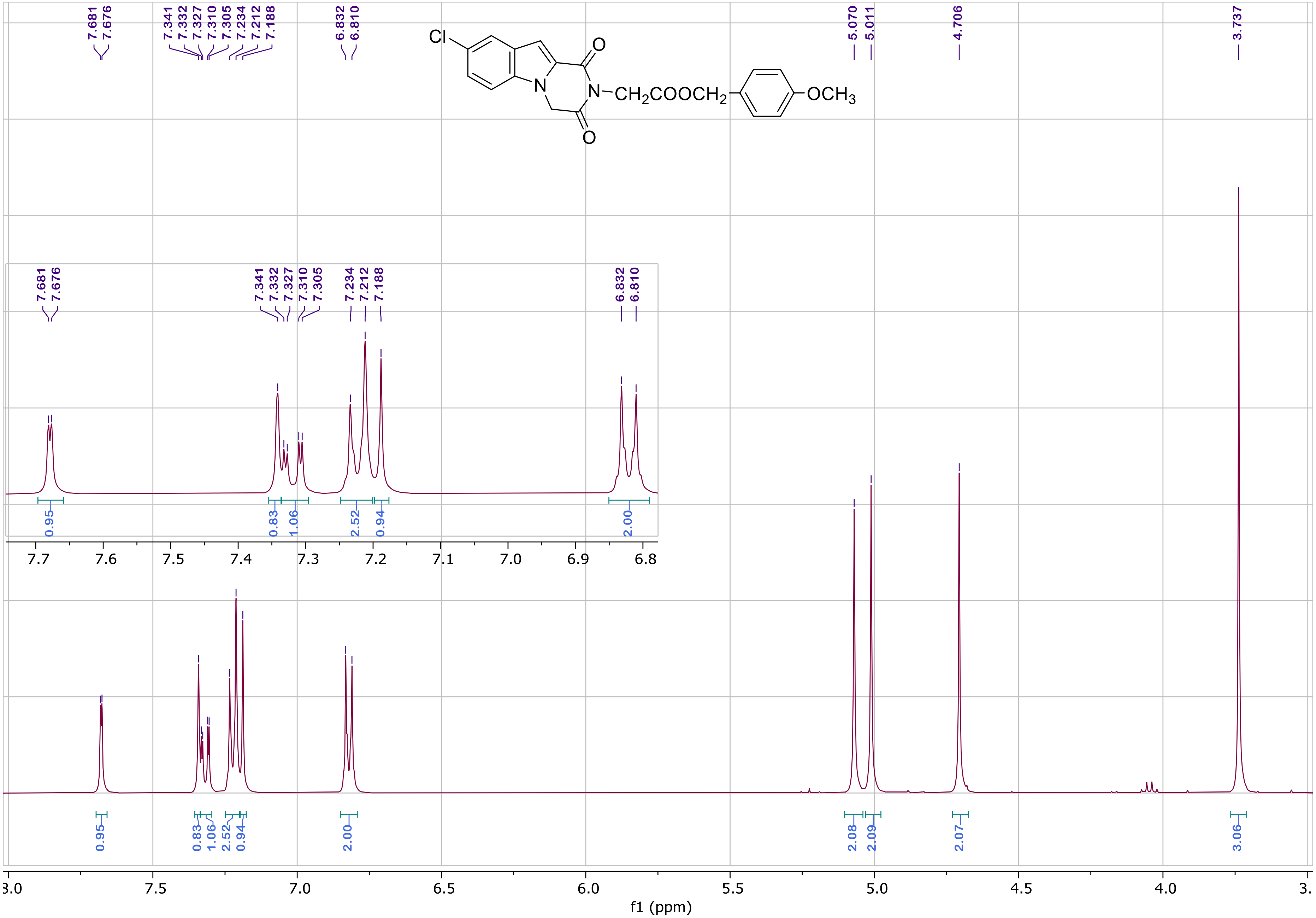

^{13}C NMR of **49** (50.32 MHz, $CDCl_3$)

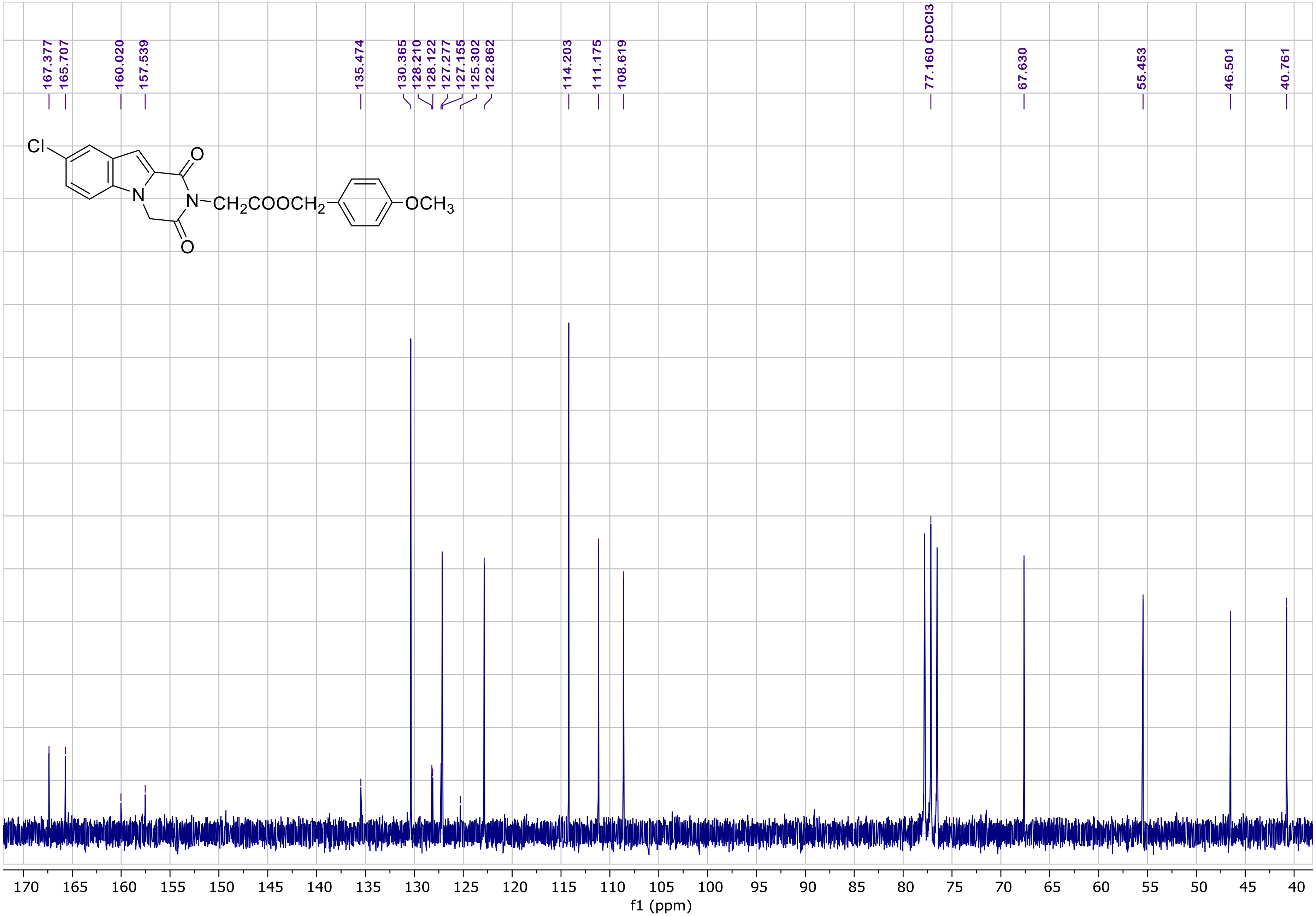

DEPT NMR of **49** (50.32 MHz, $CDCl_3$)

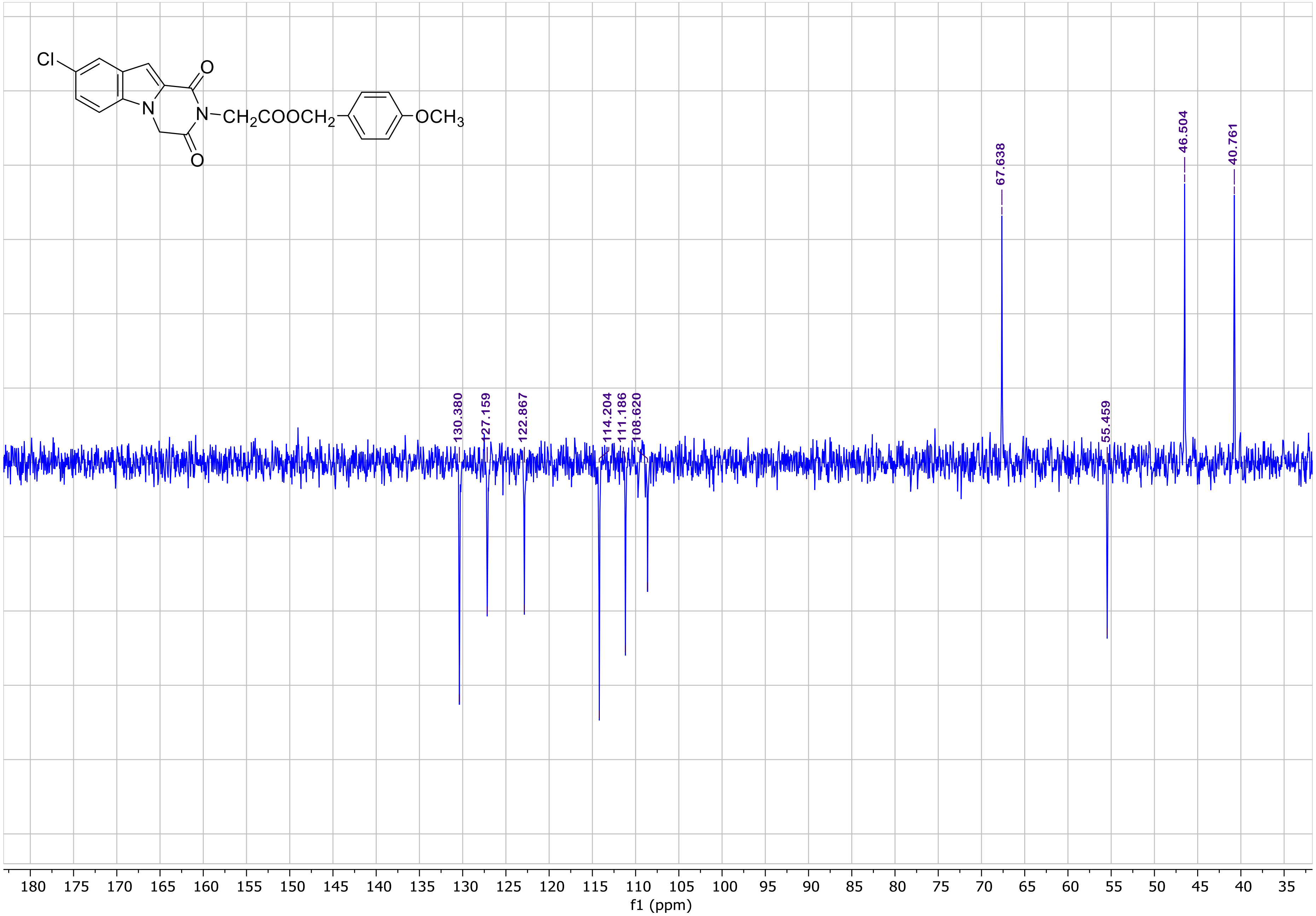

COSY NMR of **49** (400.13 MHz, CDCl3)

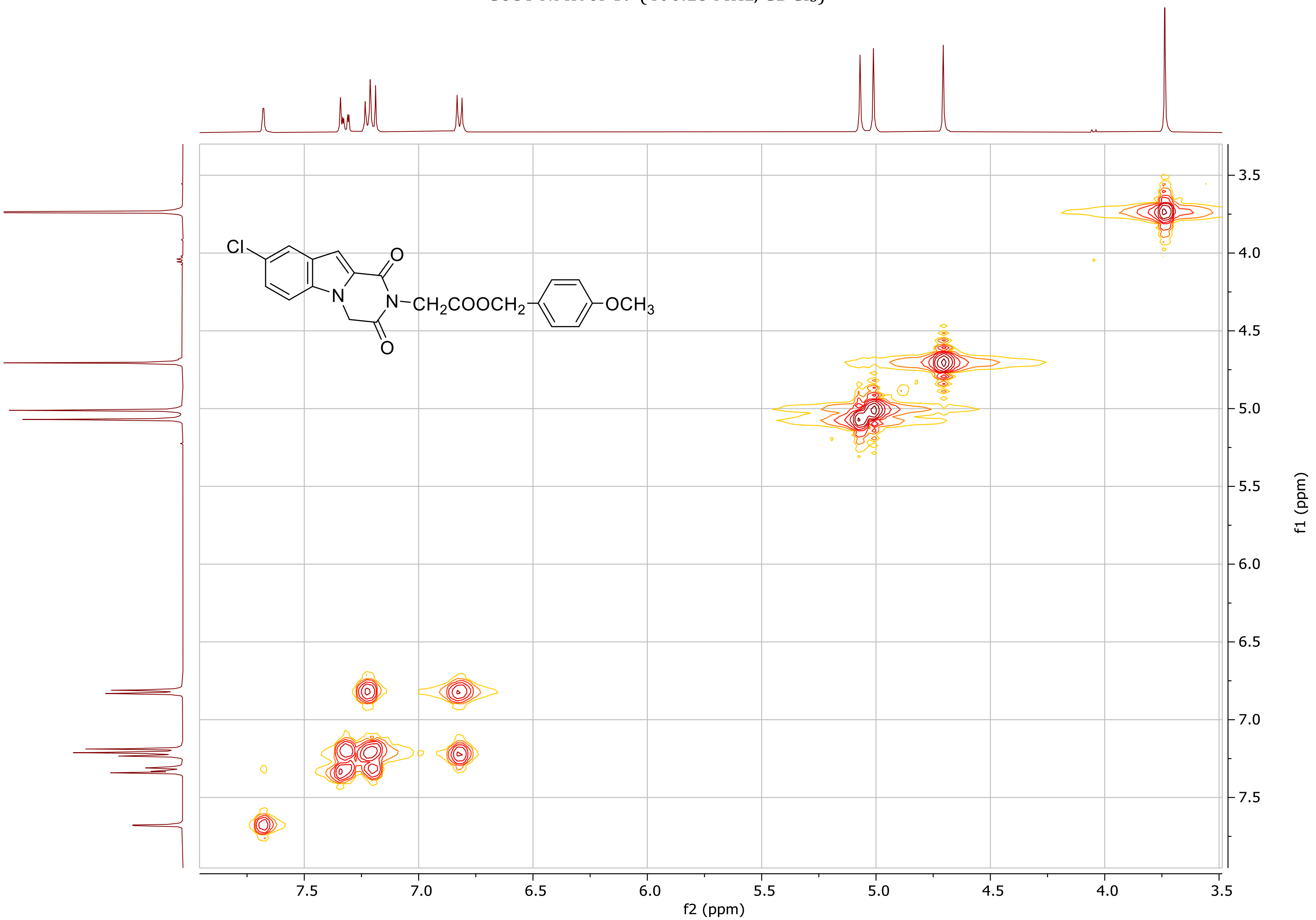

HSQC NMR of **49** (400.13 MHz, $CDCl_3$)

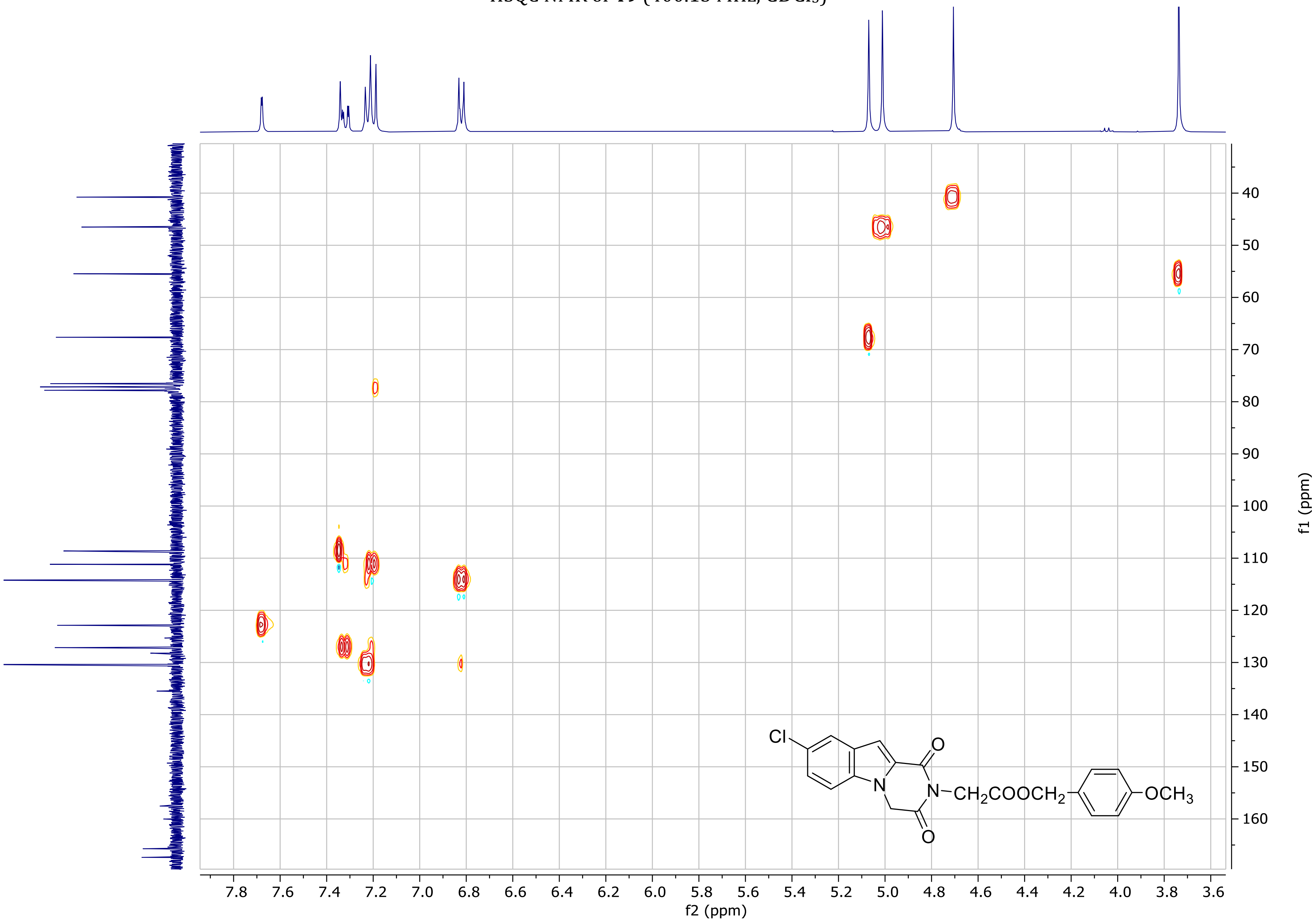

HMBC NMR of **49** (400.13 MHz, $CDCl_3$)

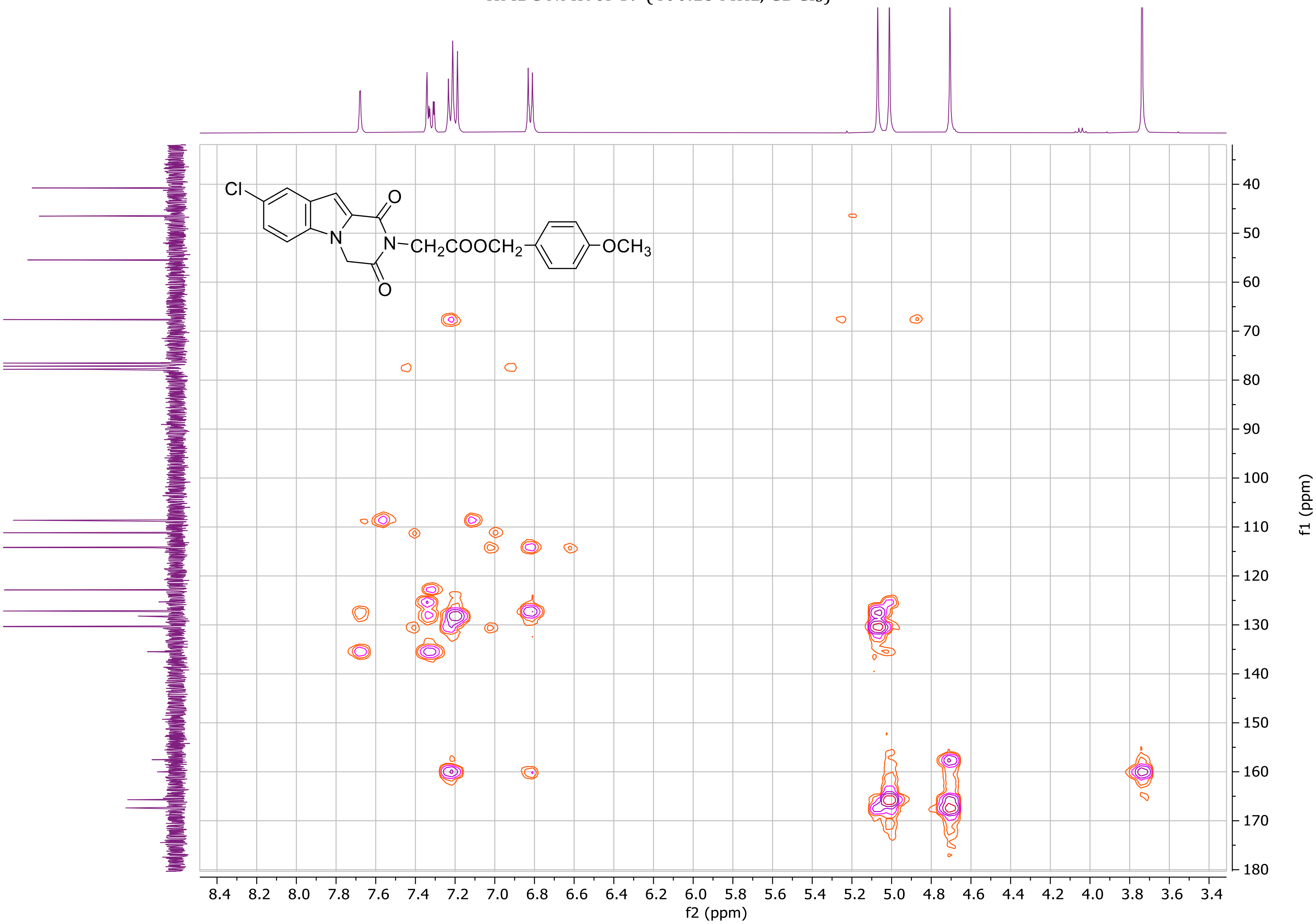

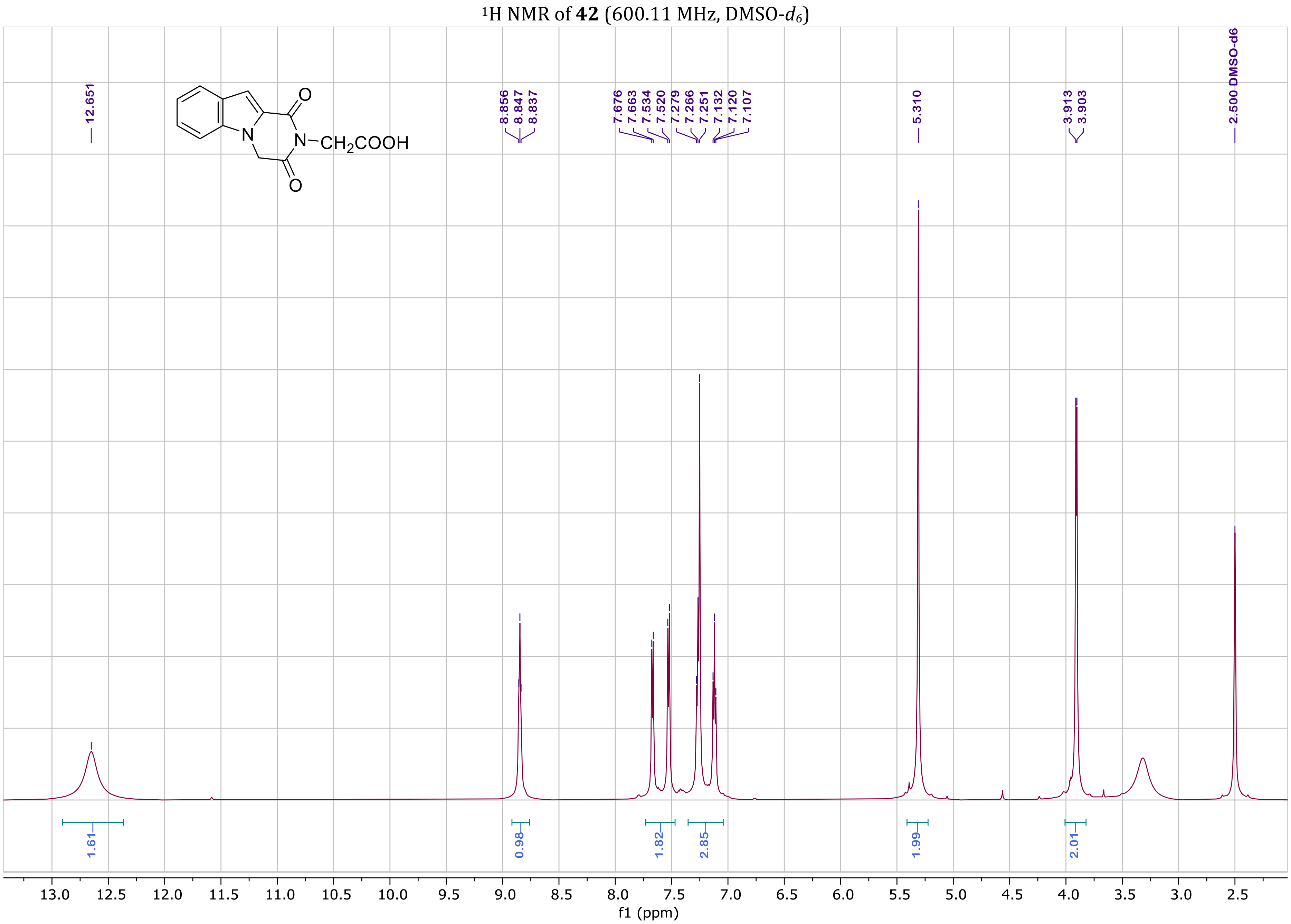

^{1}H NMR of **42** (600.11 MHz, DMSO-d_6)
N
N
O
O
CH$_2$COOH
12.651
8.856
8.847
8.837
7.676
7.663
7.534
7.520
7.279
7.266
7.251
7.132
7.120
7.107
5.310
3.913
3.903
2.500 DMSO-d6
1.61
0.98
1.82
2.85
1.99
2.01
13.0 12.5 12.0 11.5 11.0 10.5 10.0 9.5 9.0 8.5 8.0 7.5 7.0 6.5 6.0 5.5 5.0 4.5 4.0 3.5 3.0 2.5
f1 (ppm)

^{13}C NMR of **42** (50.32 MHz, DMSO-d_6)

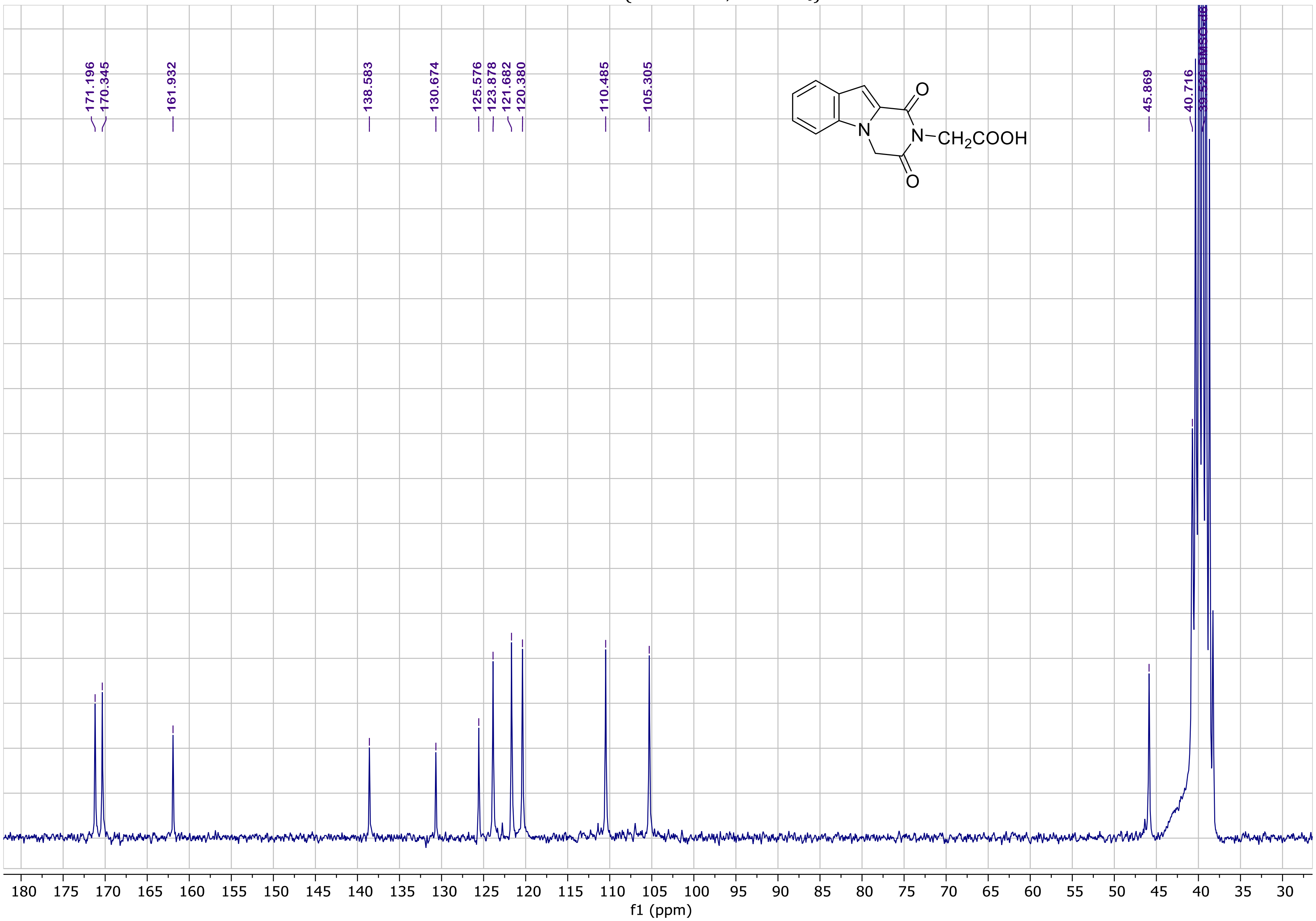

DEPT NMR of **42** (50.32 MHz, DMSO-d_6)

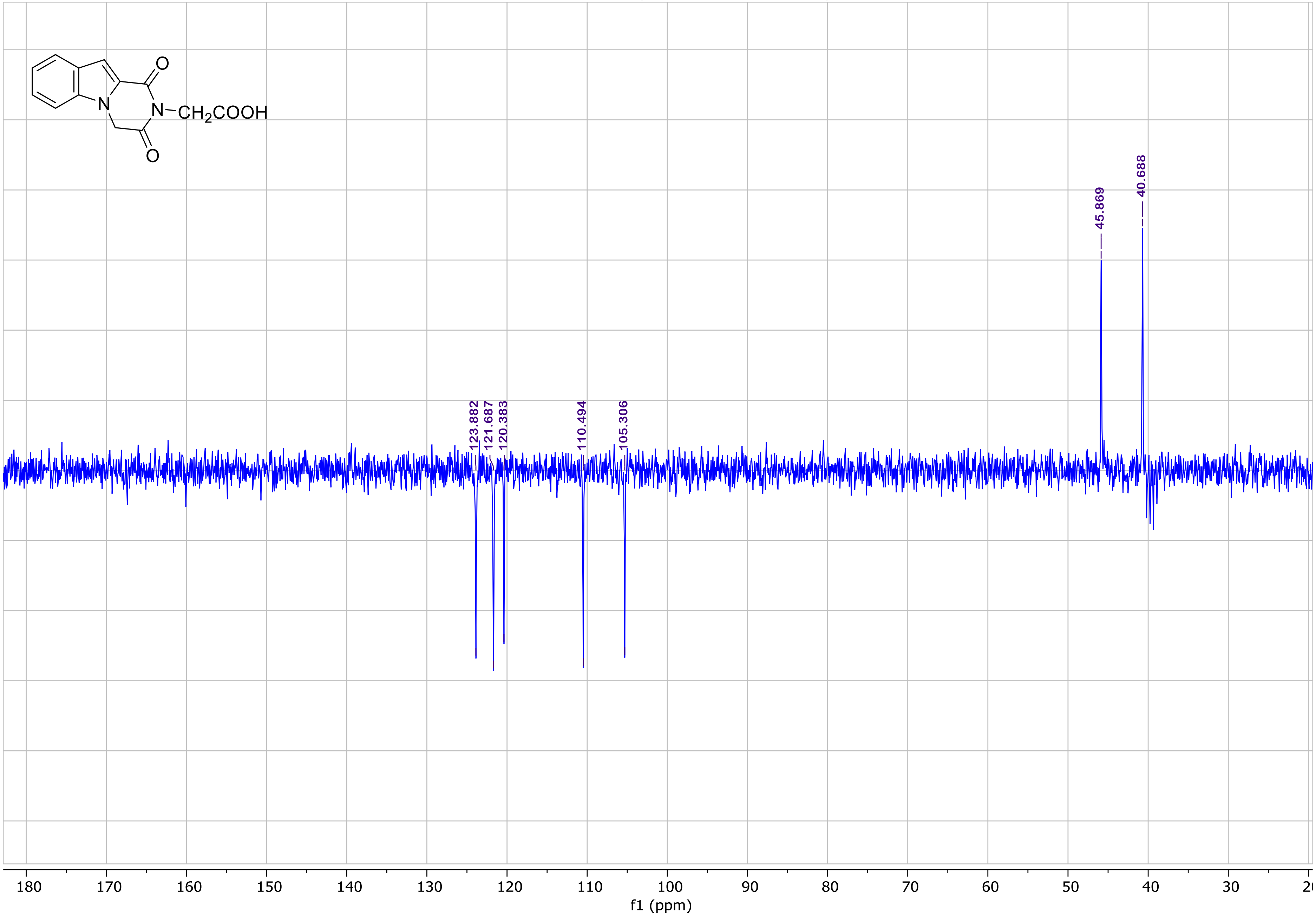

COSY NMR of **42** (600.11 MHz, DMSO-d_6)

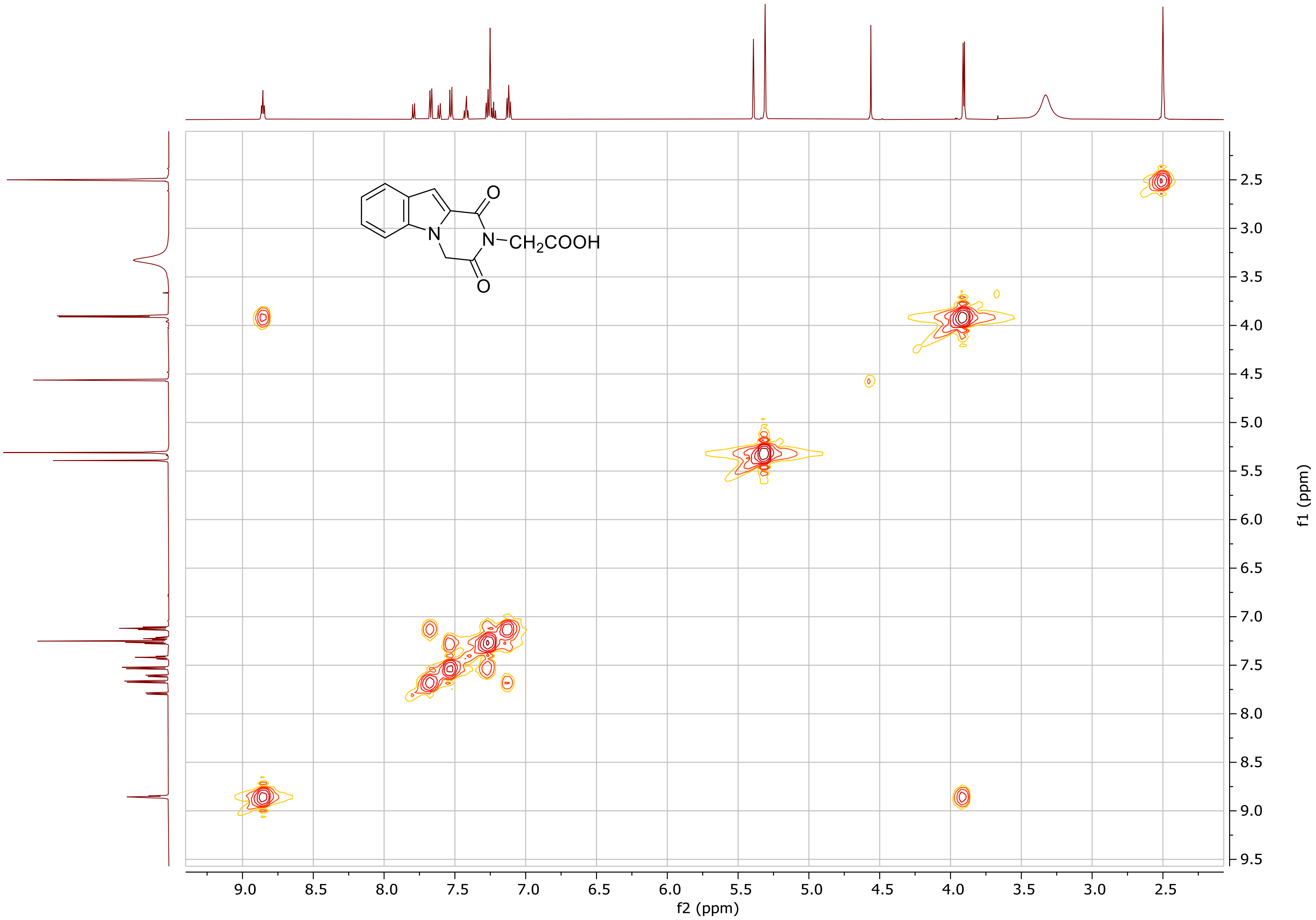

HSQC-DEPT NMR of **42** (600.11 MHz, DMSO-d_6)

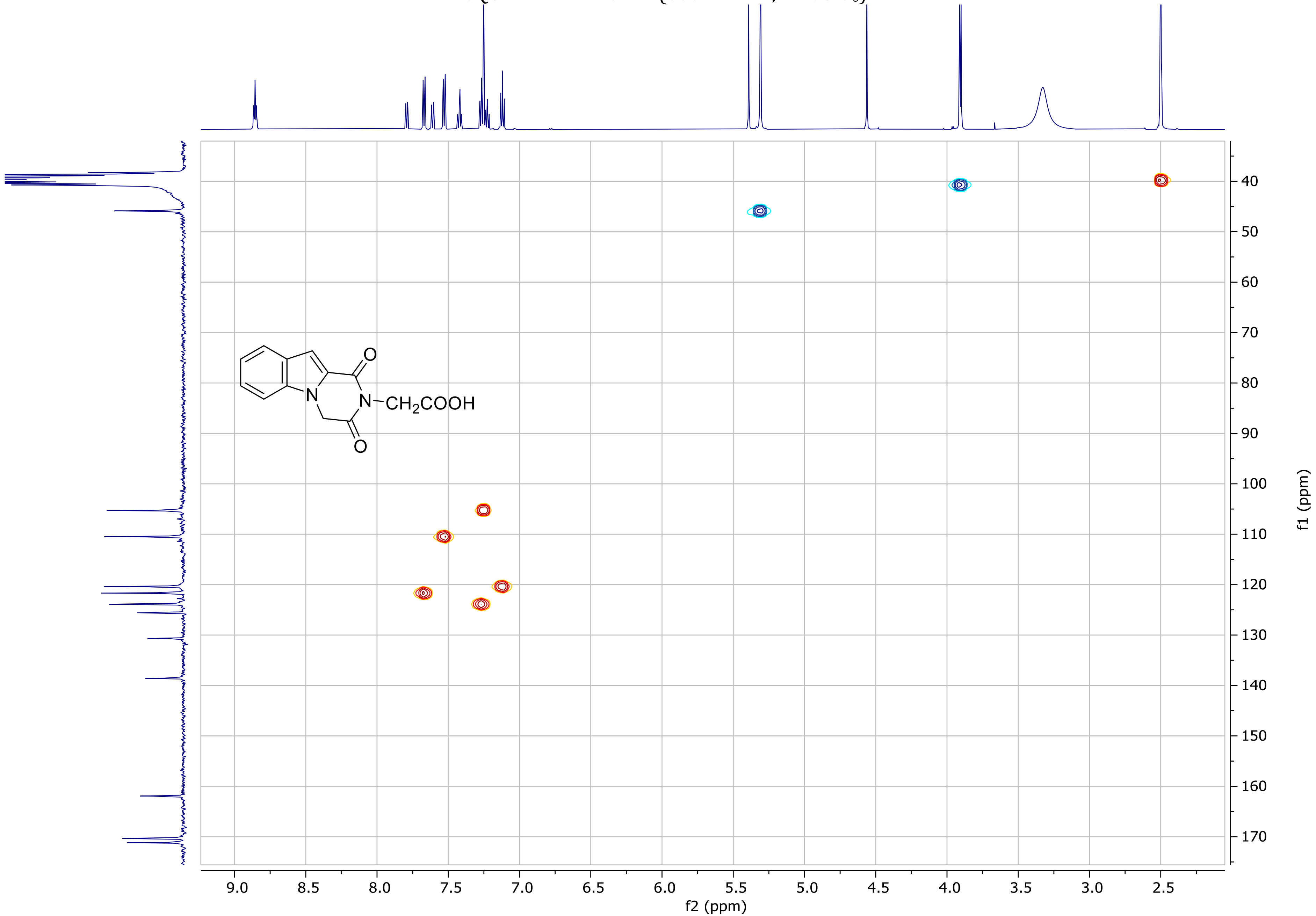

HMBC NMR of **42** (600.11 MHz, DMSO-d_6)

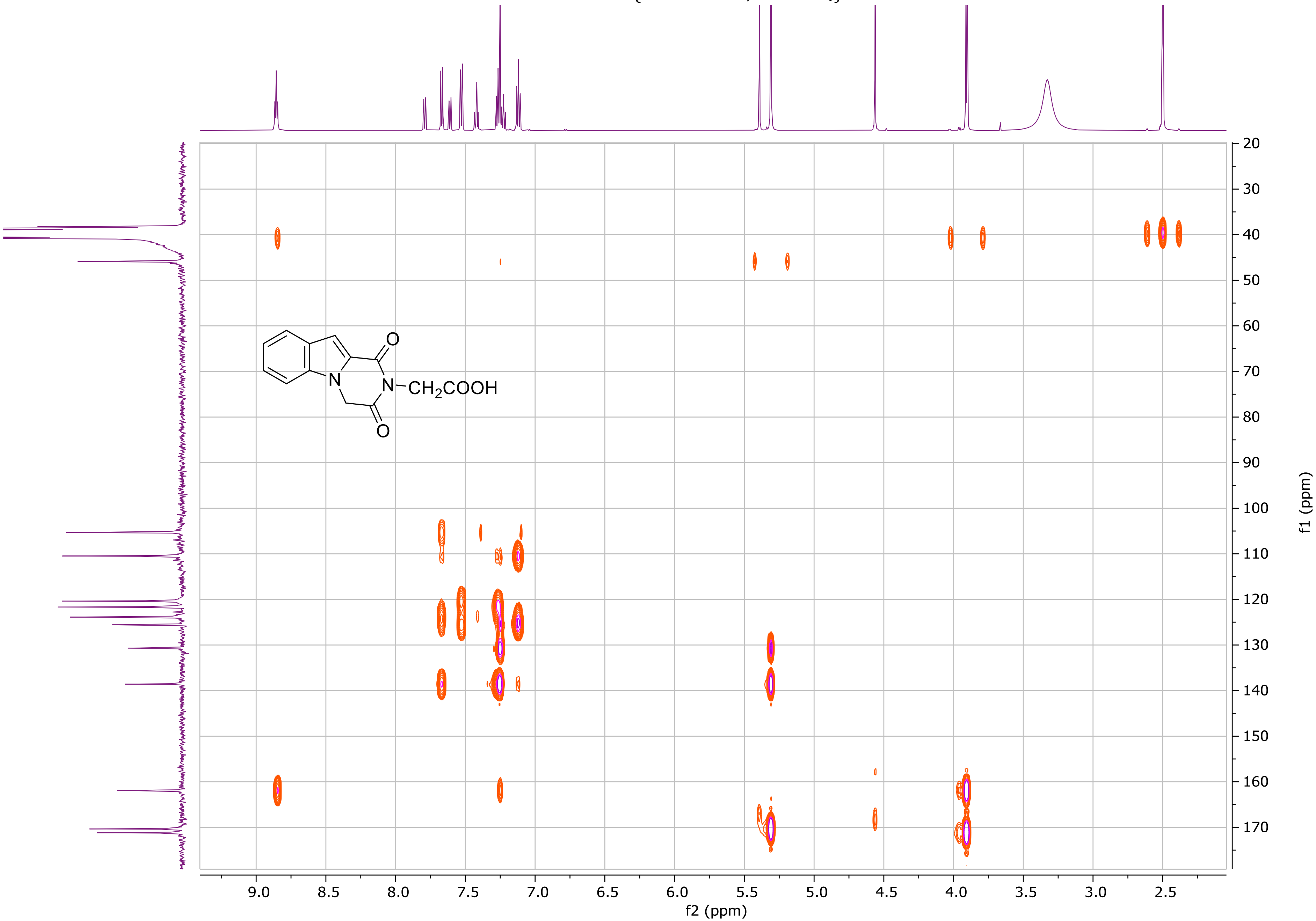

^{1}H NMR of **46** (400.13 MHz, DMSO-d_6)

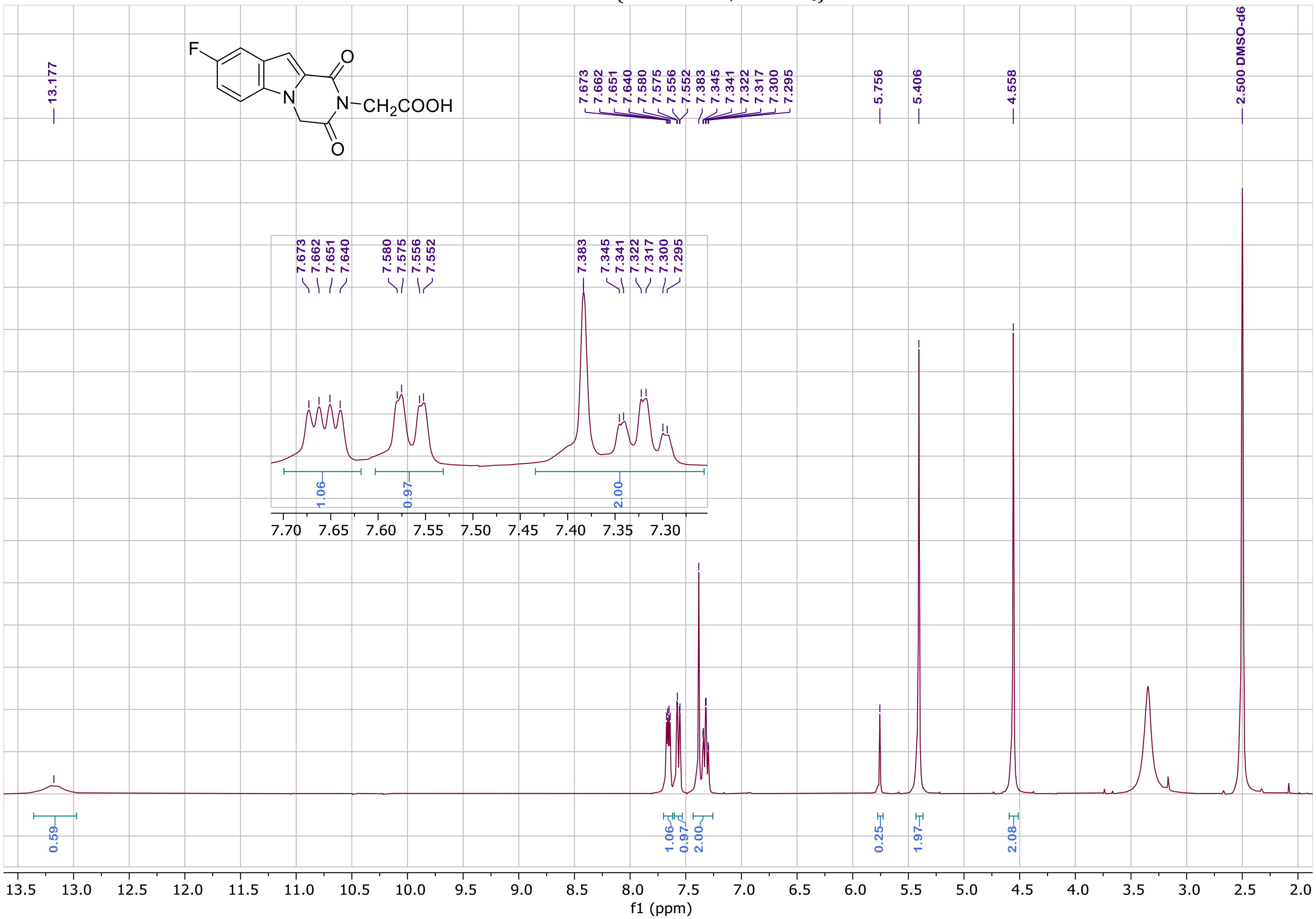

^{13}C NMR of **46** (50.32 MHz, DMSO-d_6)

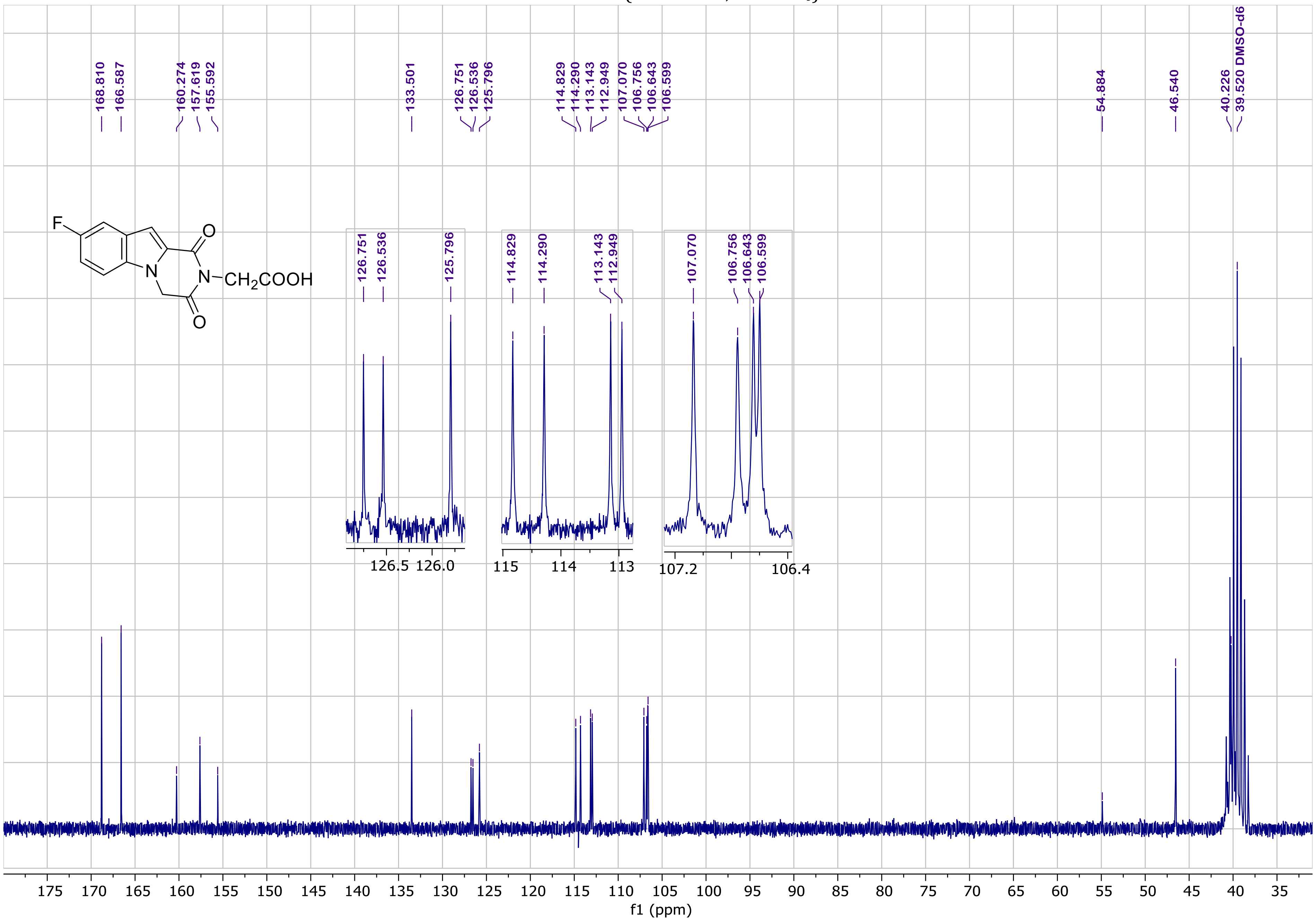

DEPT NMR of **46** (50.32 MHz, $CDCl_3$)

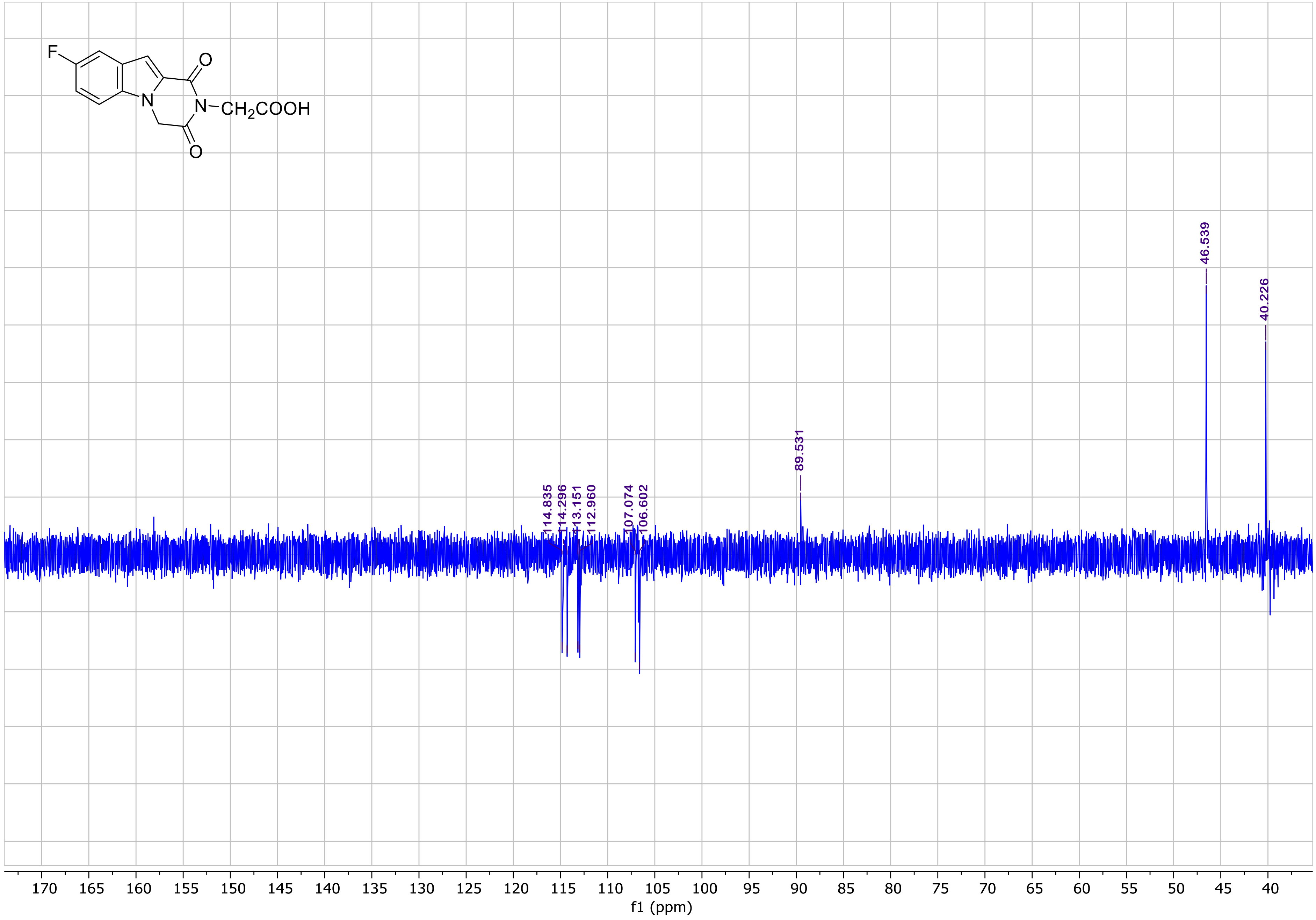

COSY NMR of **46** (400.13 MHz, DMSO-d_6)

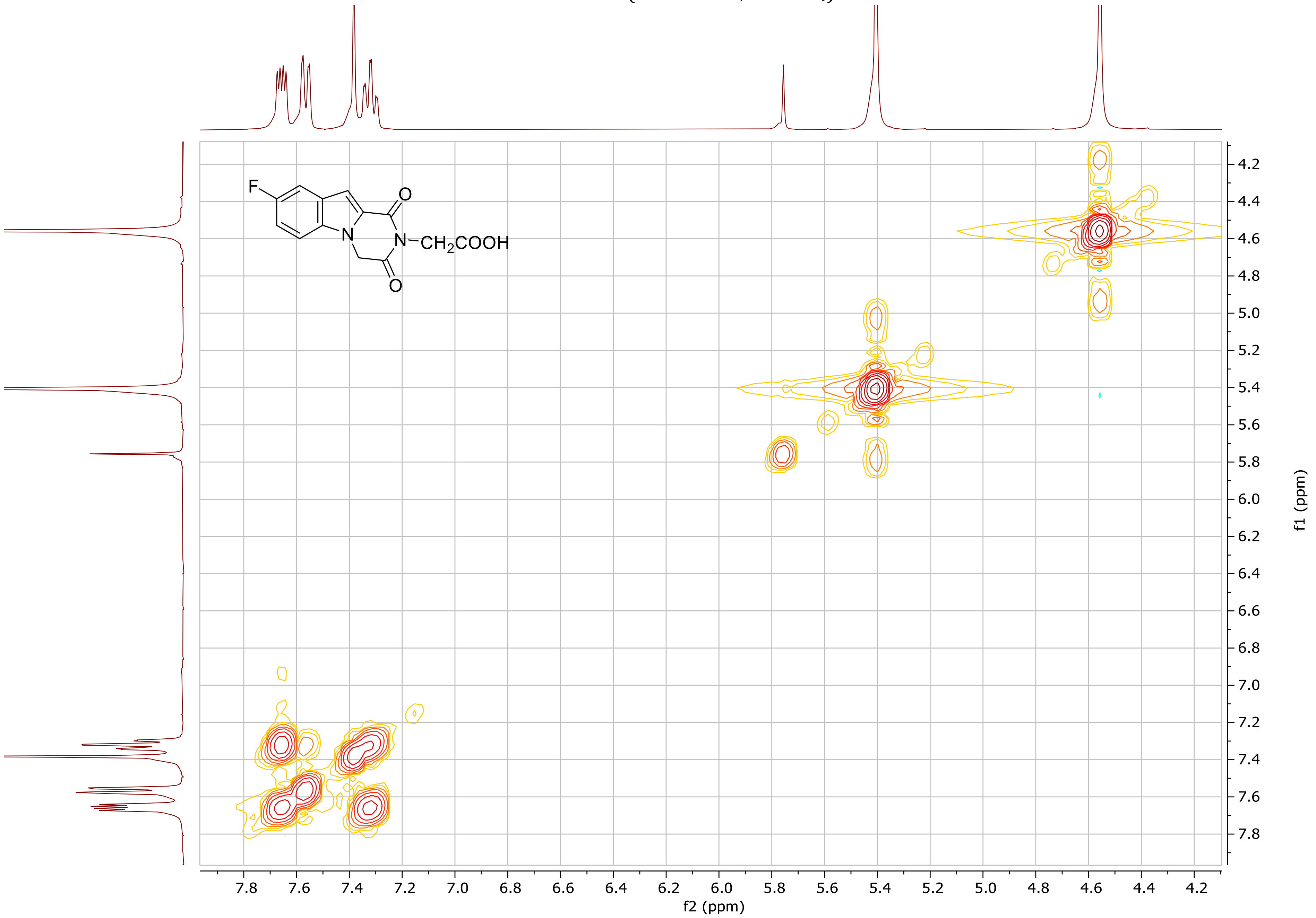

HSQC NMR of **46** (400.13 MHz, DMSO-d_6)

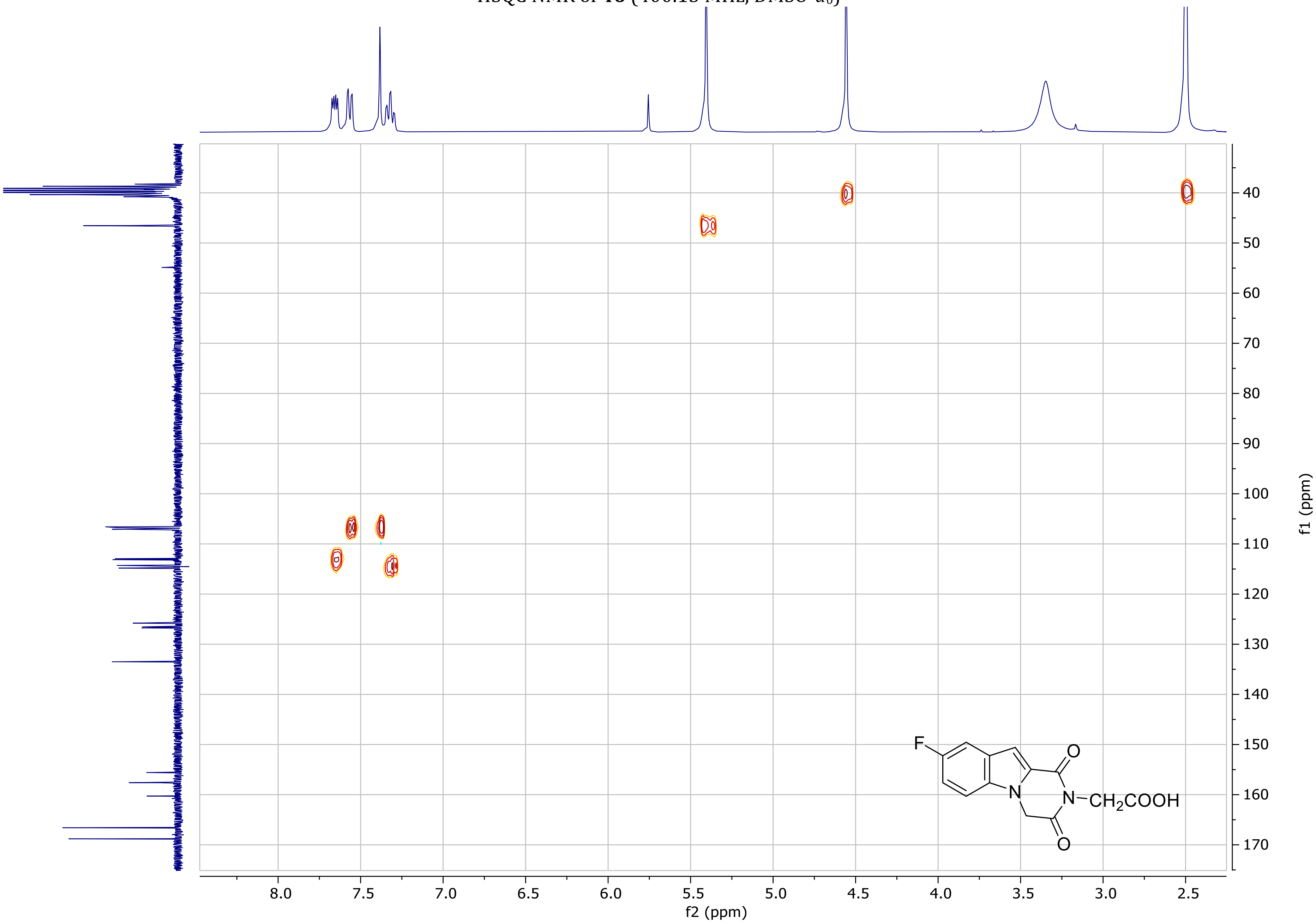

HMBC NMR of **46** (400.13 MHz, DMSO-d_6)

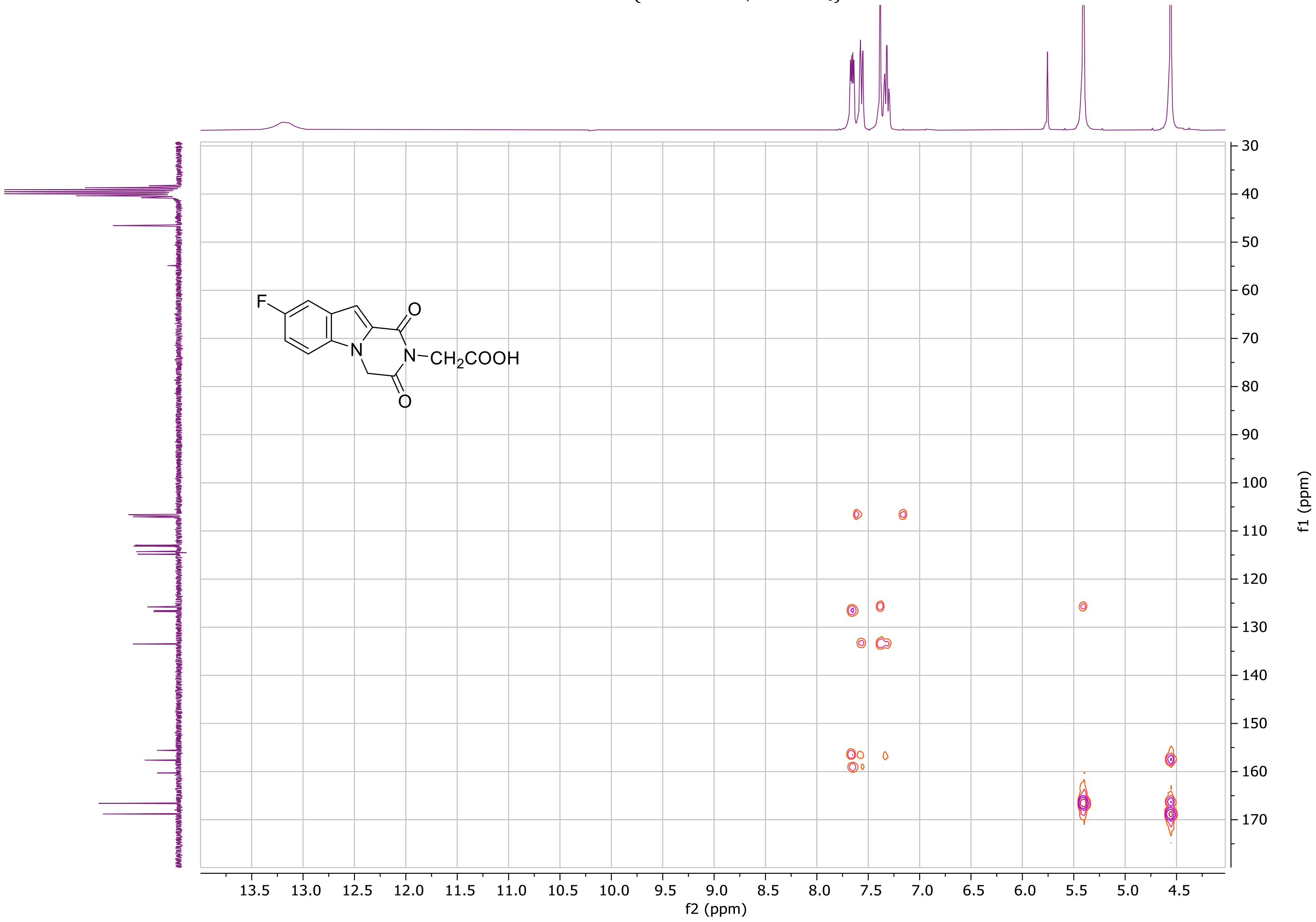

^{1}H NMR of **50** (400.13 MHz, DMSO-d_6)

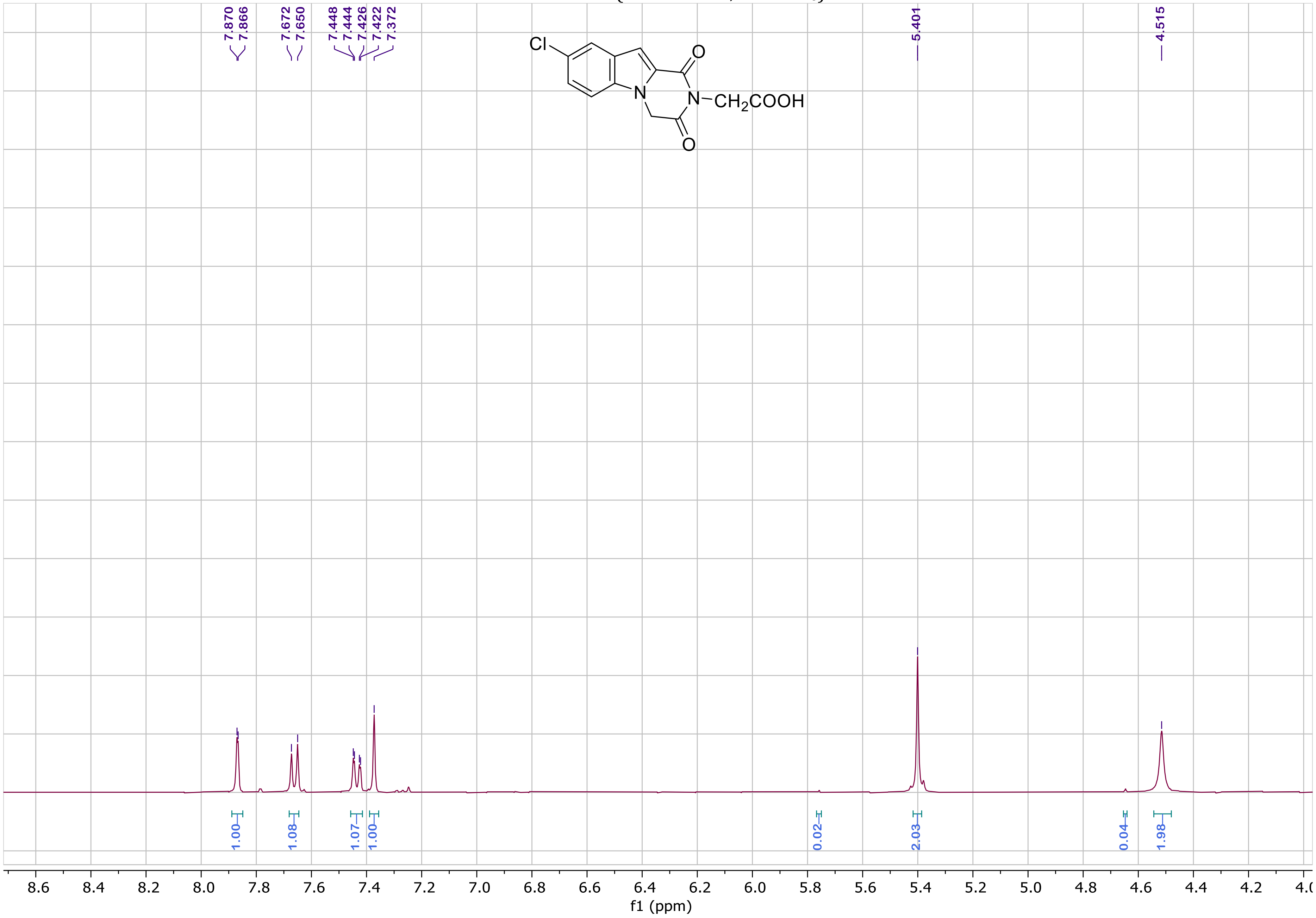

^{13}C NMR of **50** (50.32 MHz, DMSO-d_6)

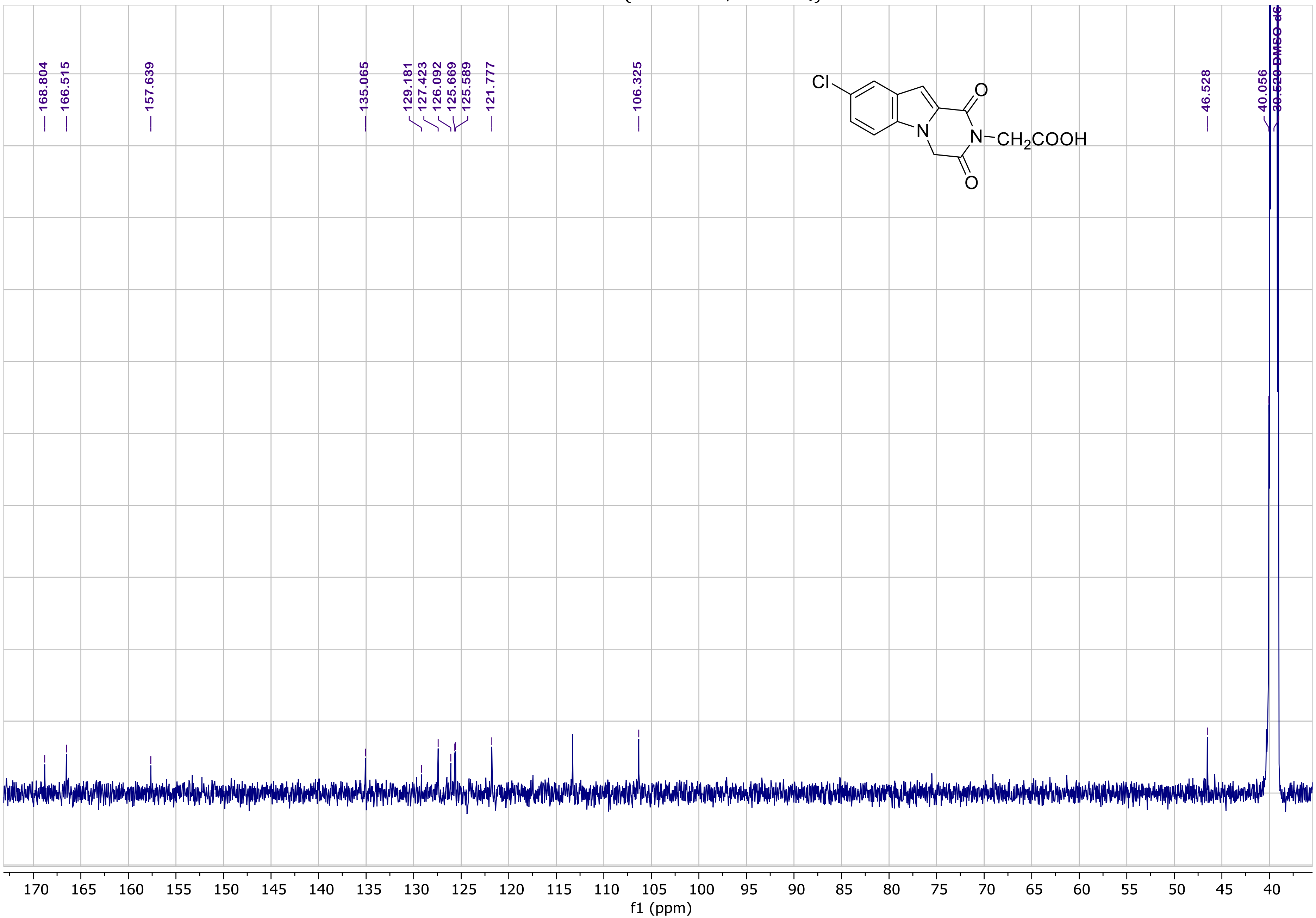

DEPT NMR of **50** (50.32 MHz, DMSO-d_6)

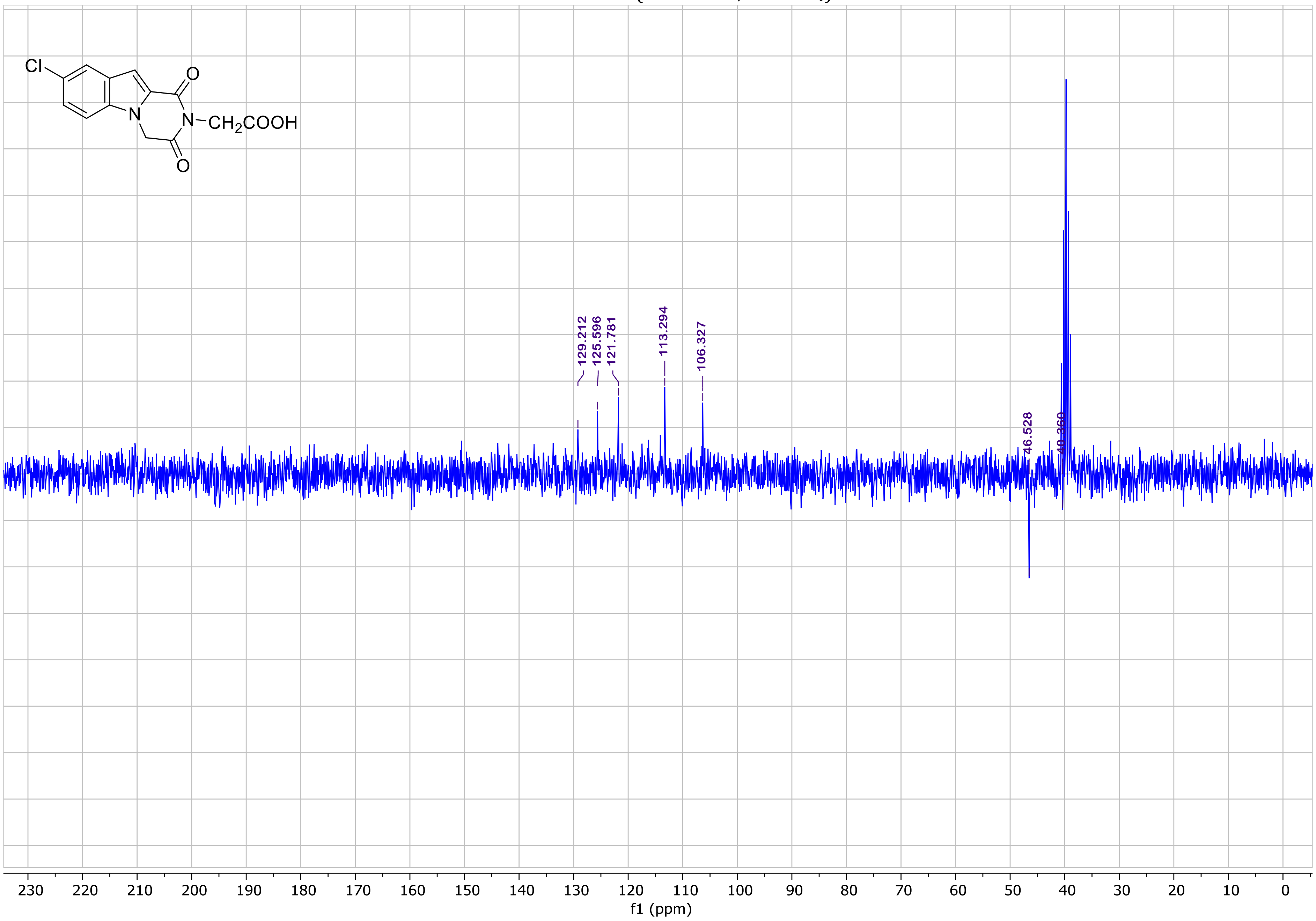

COSY NMR of **50** (400.13 MHz, DMSO-d_6)

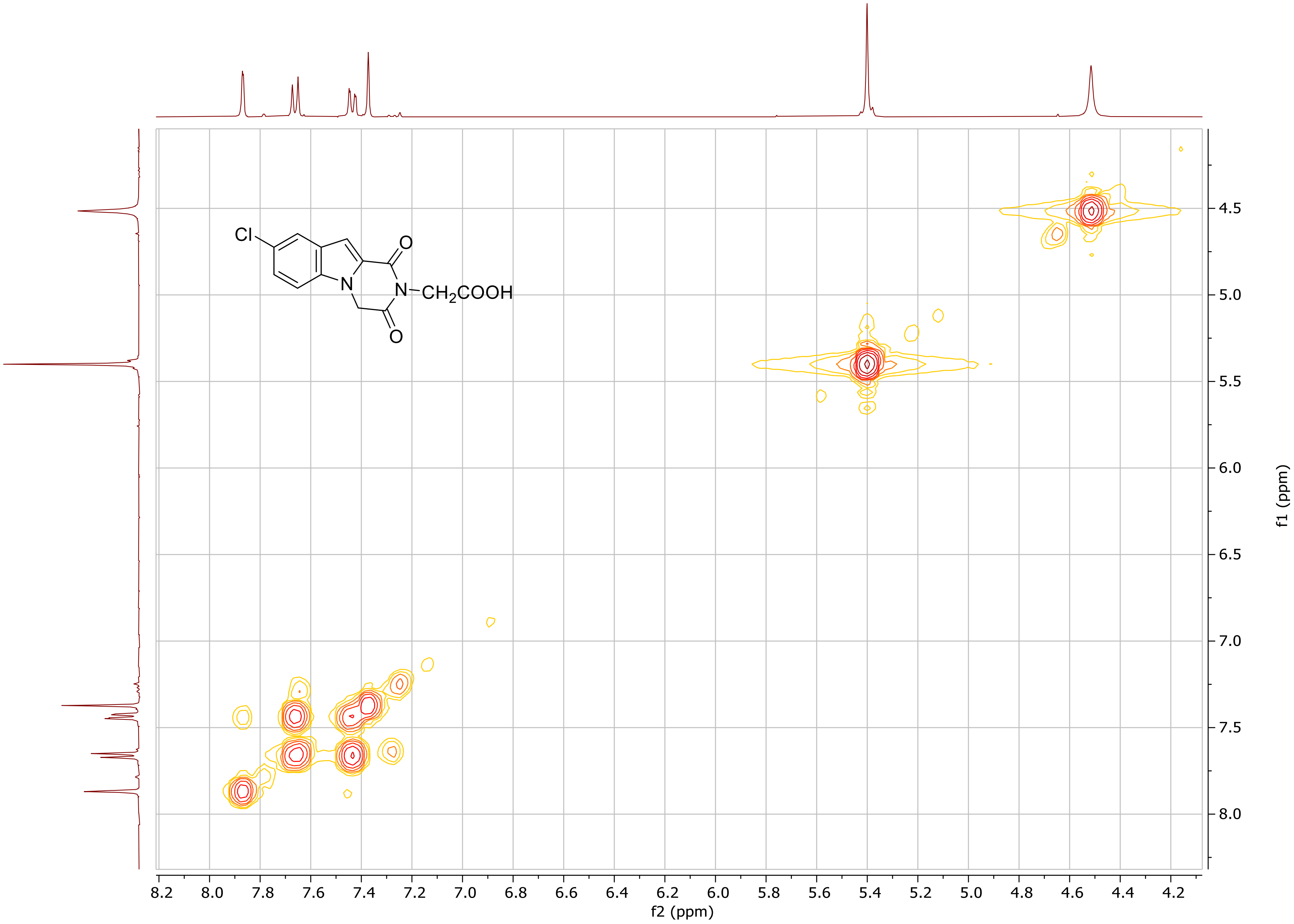

HSQC NMR of **50** (400.13 MHz, DMSO-d_6)

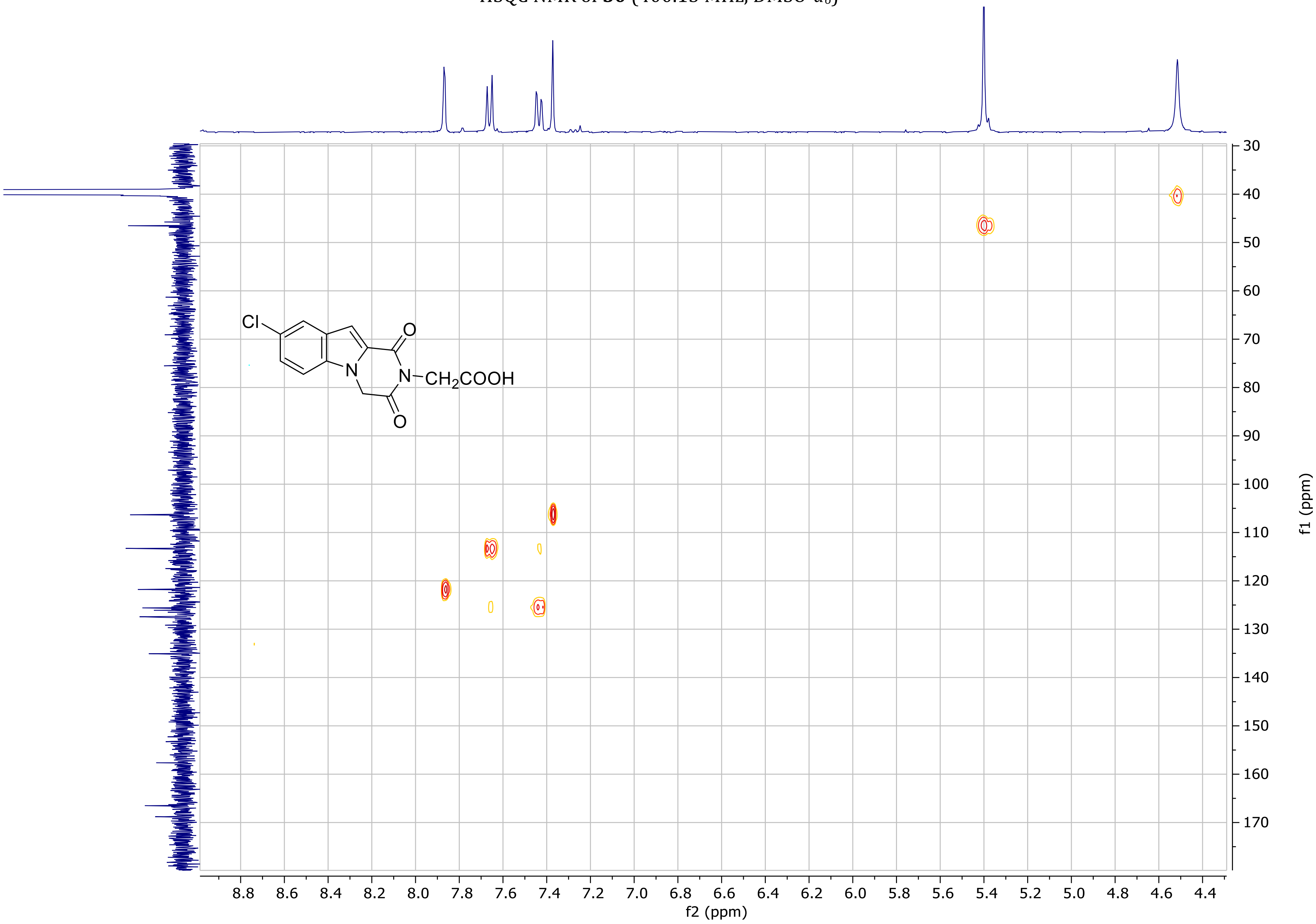

HMBC NMR of **50** (400.13 MHz, DMSO-d_6)

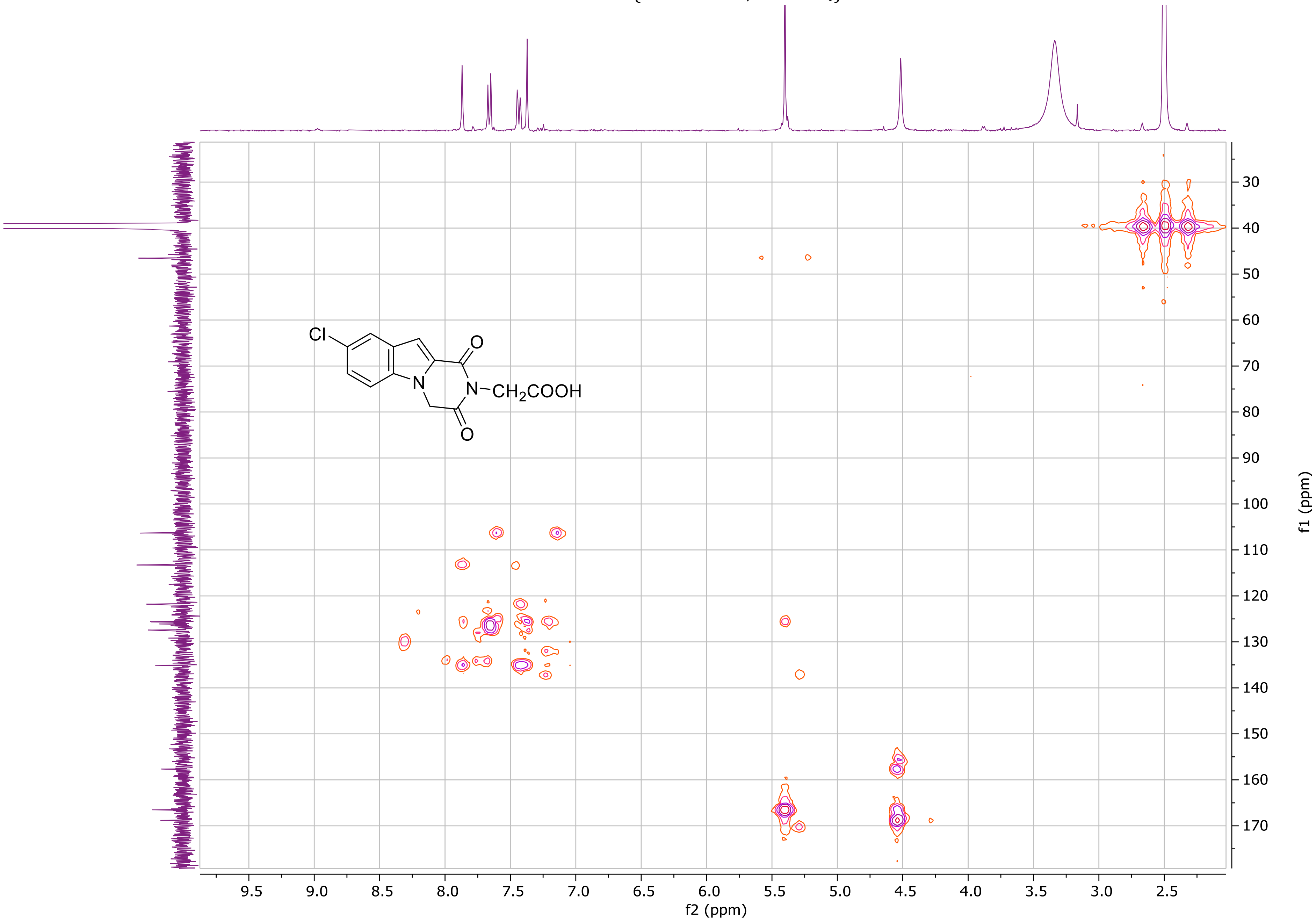

^{1}H NMR of **43** (600.11 MHz, DMSO-d_6)

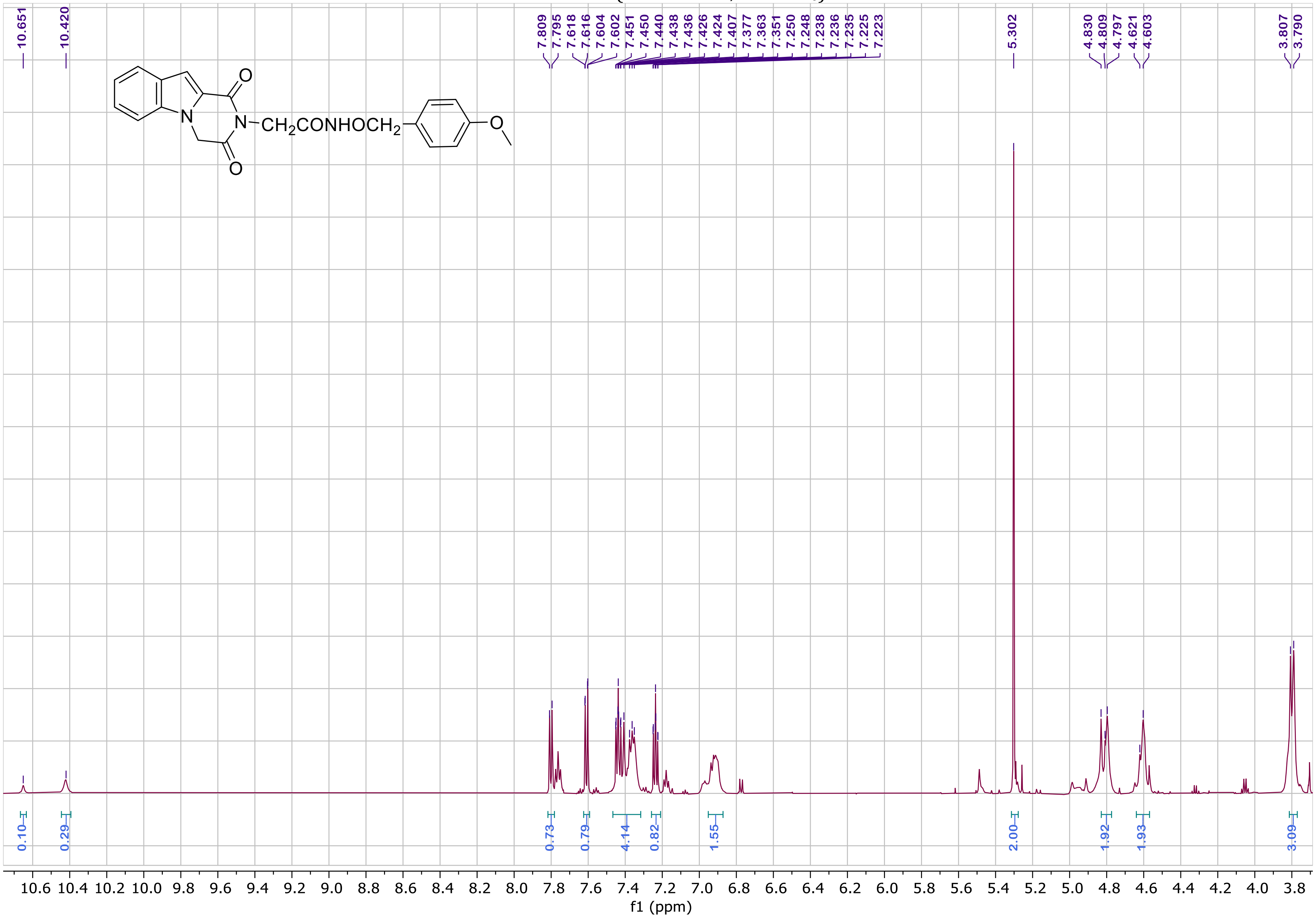

^{13}C NMR of **43** (50.32 MHz, DMSO-d_6)

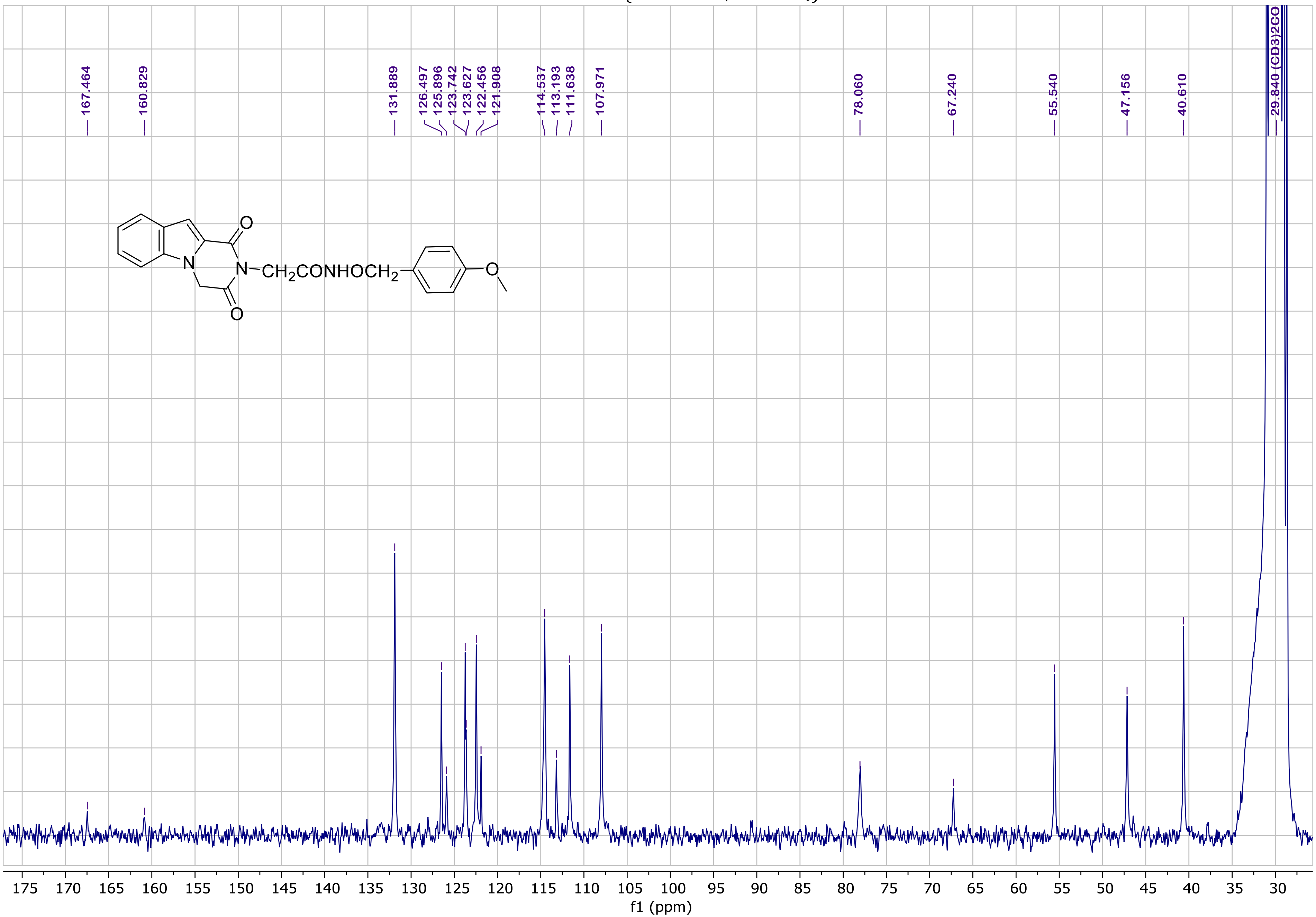

DEPT NMR of **43** (50.32 MHz, DMSO-d_6)

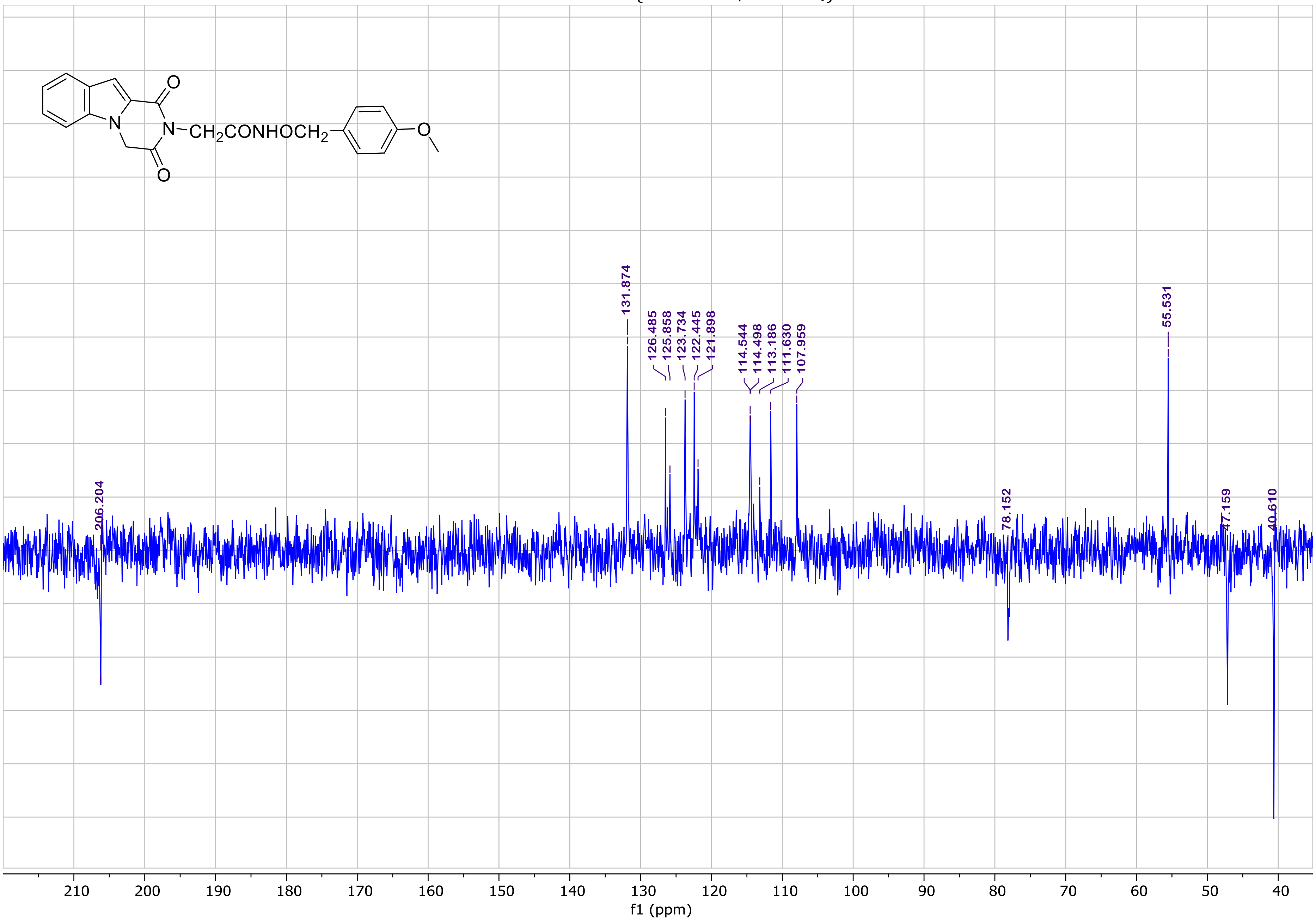

COSY NMR of **43** (600.11 MHz, DMSO-d_6)

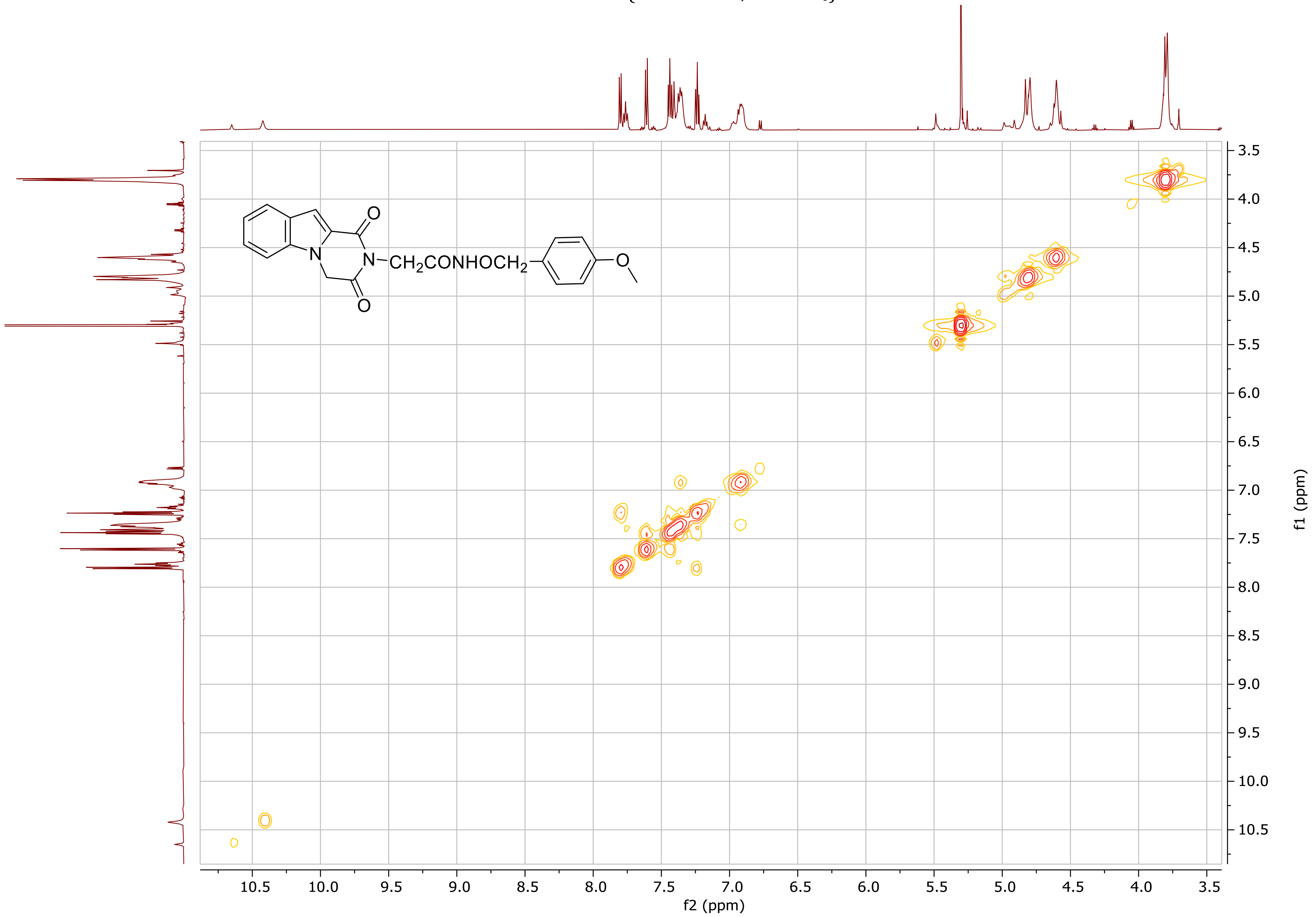

HSQC-DEPT NMR of **43** (600.11 MHz, DMSO-d_6)

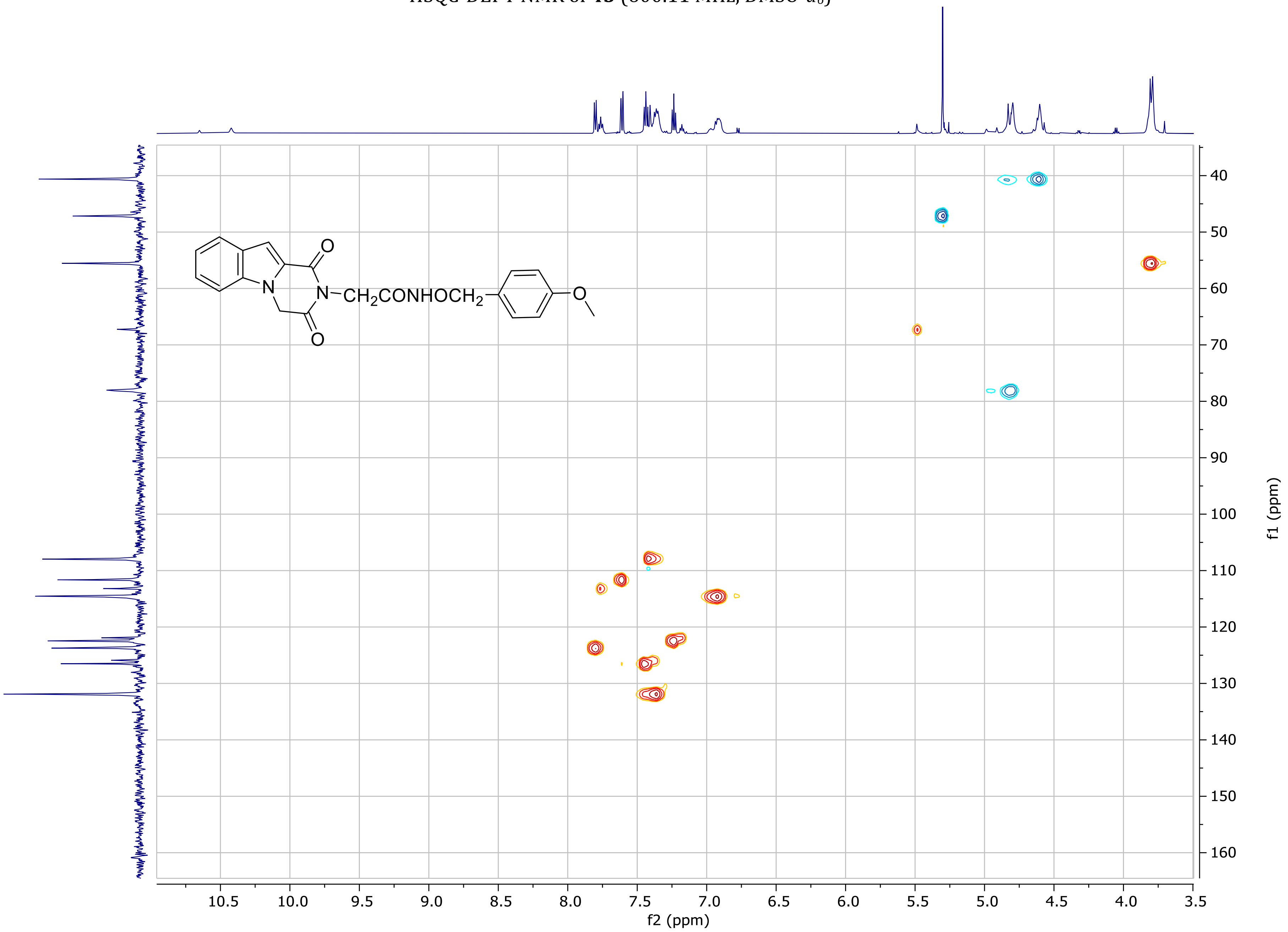

HMBC NMR of **43** (600.11 MHz, DMSO-d_6)

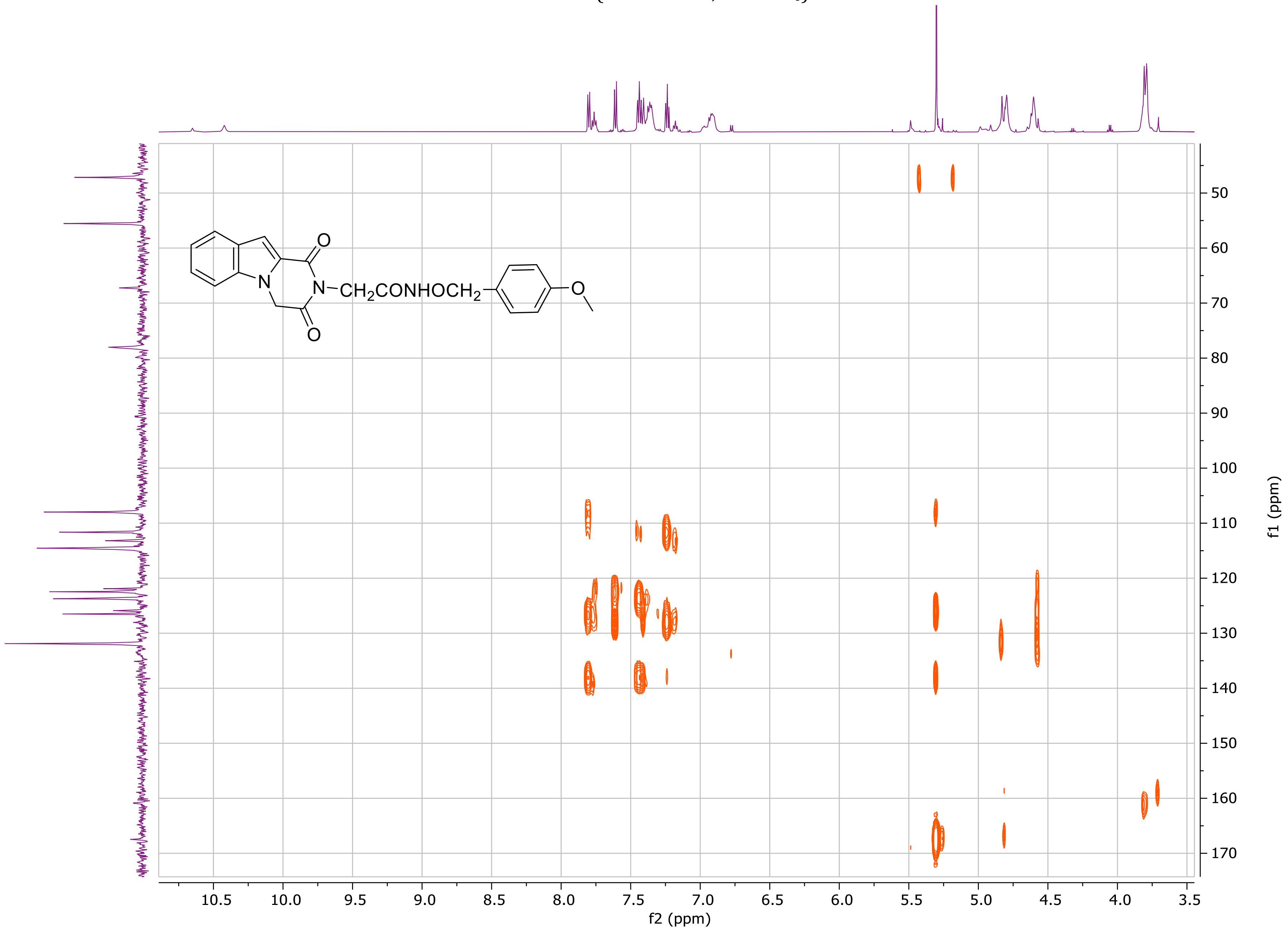

^{1}H NMR of **16** (600.11 MHz, DMSO-d_6)

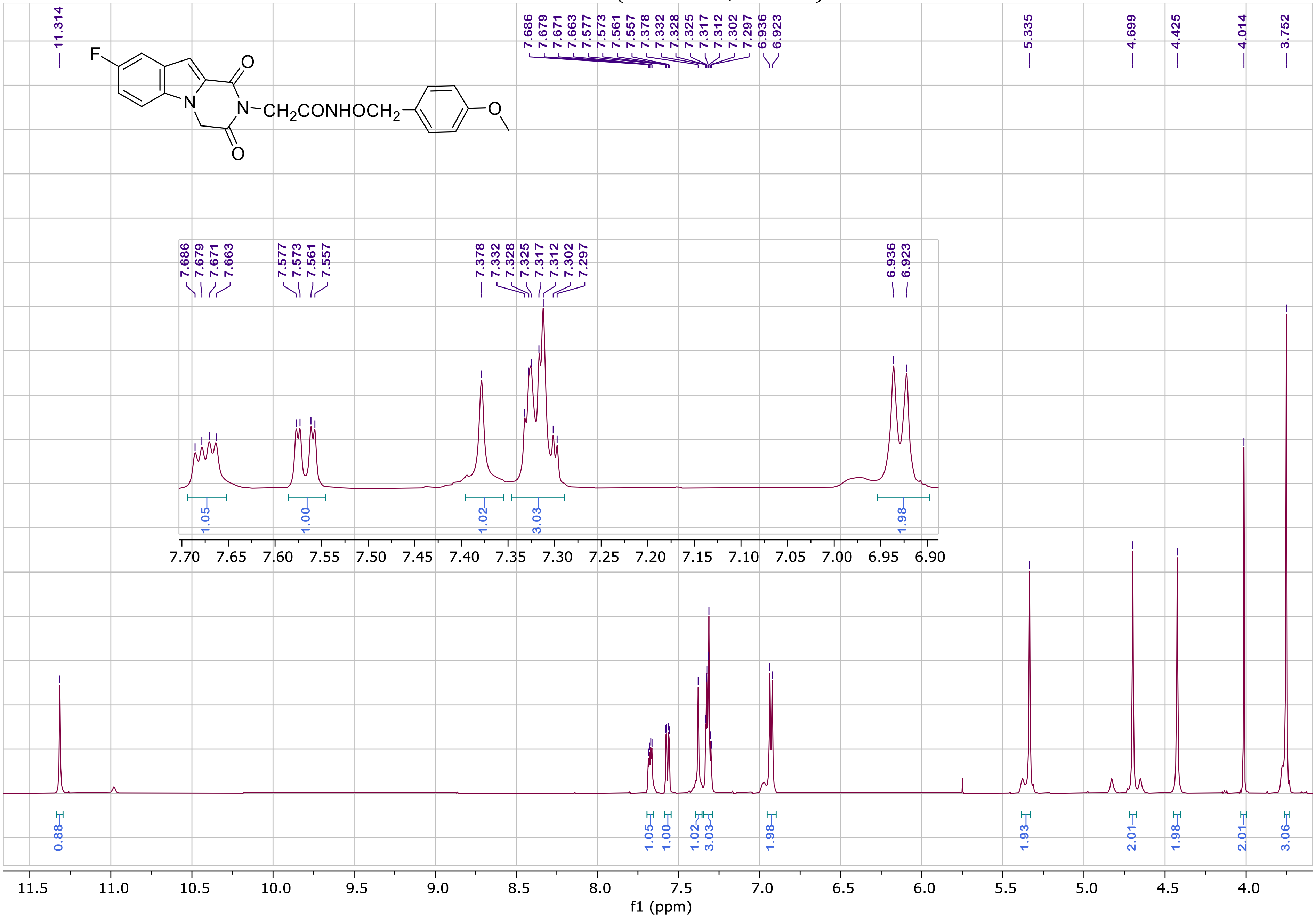

^{13}C NMR of **16** (50.32 MHz, DMSO-d_6)

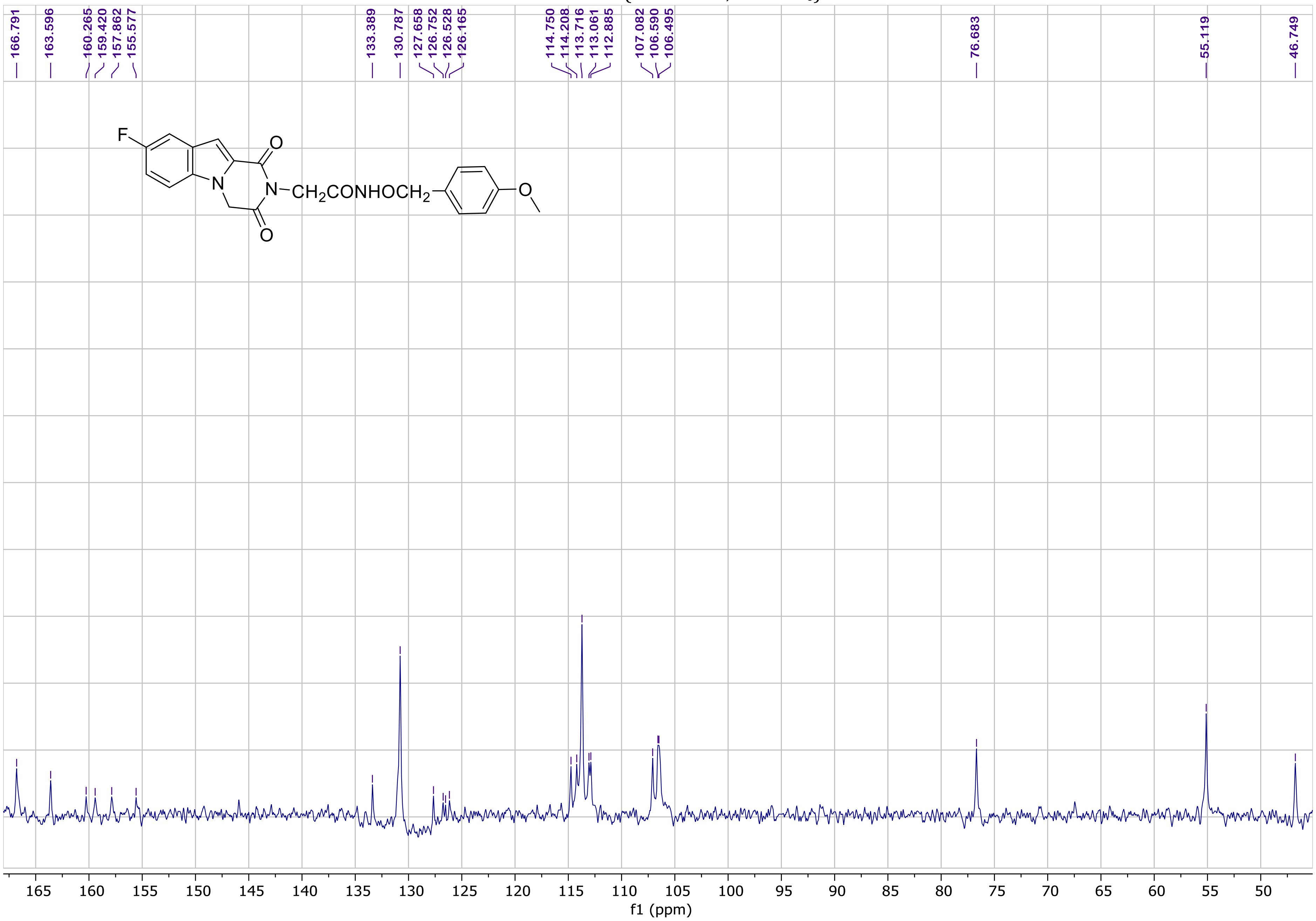

DEPT NMR of **16** (50.32 MHz, DMSO-d_6)

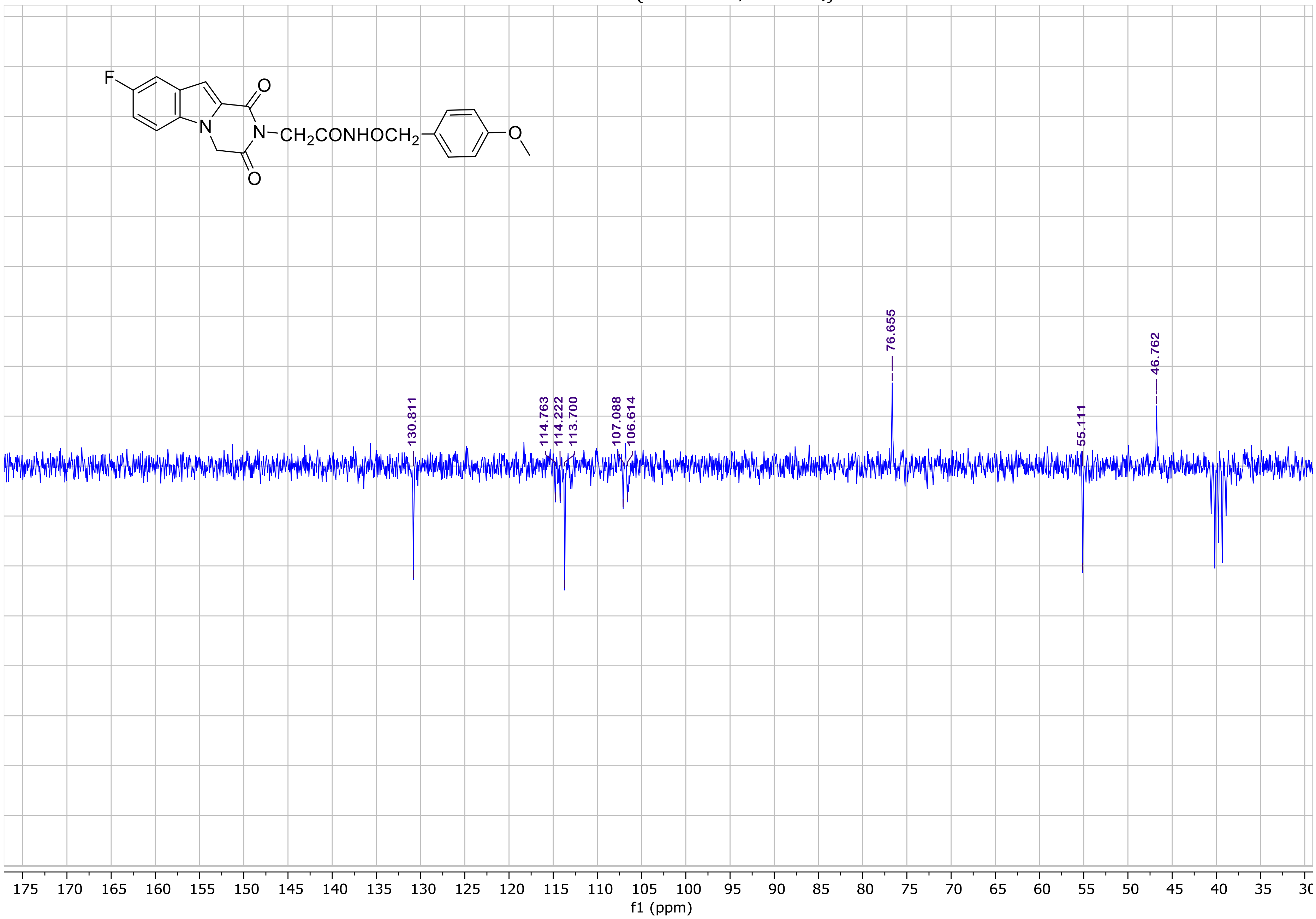

COSY NMR of **16** (600.11 MHz, DMSO-d_6)

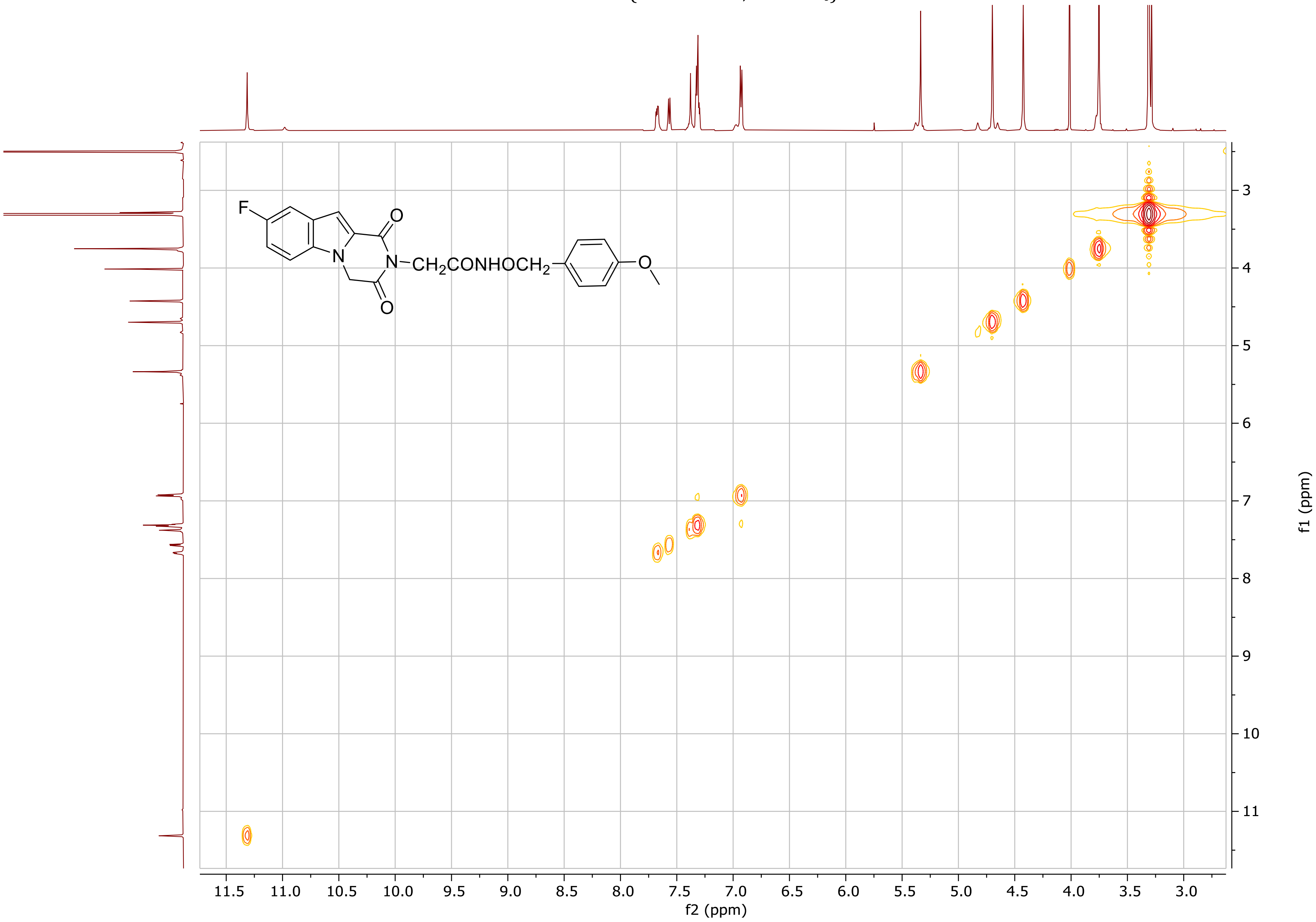

HSQC-DEPT NMR of **16** (600.11 MHz, DMSO-d_6)

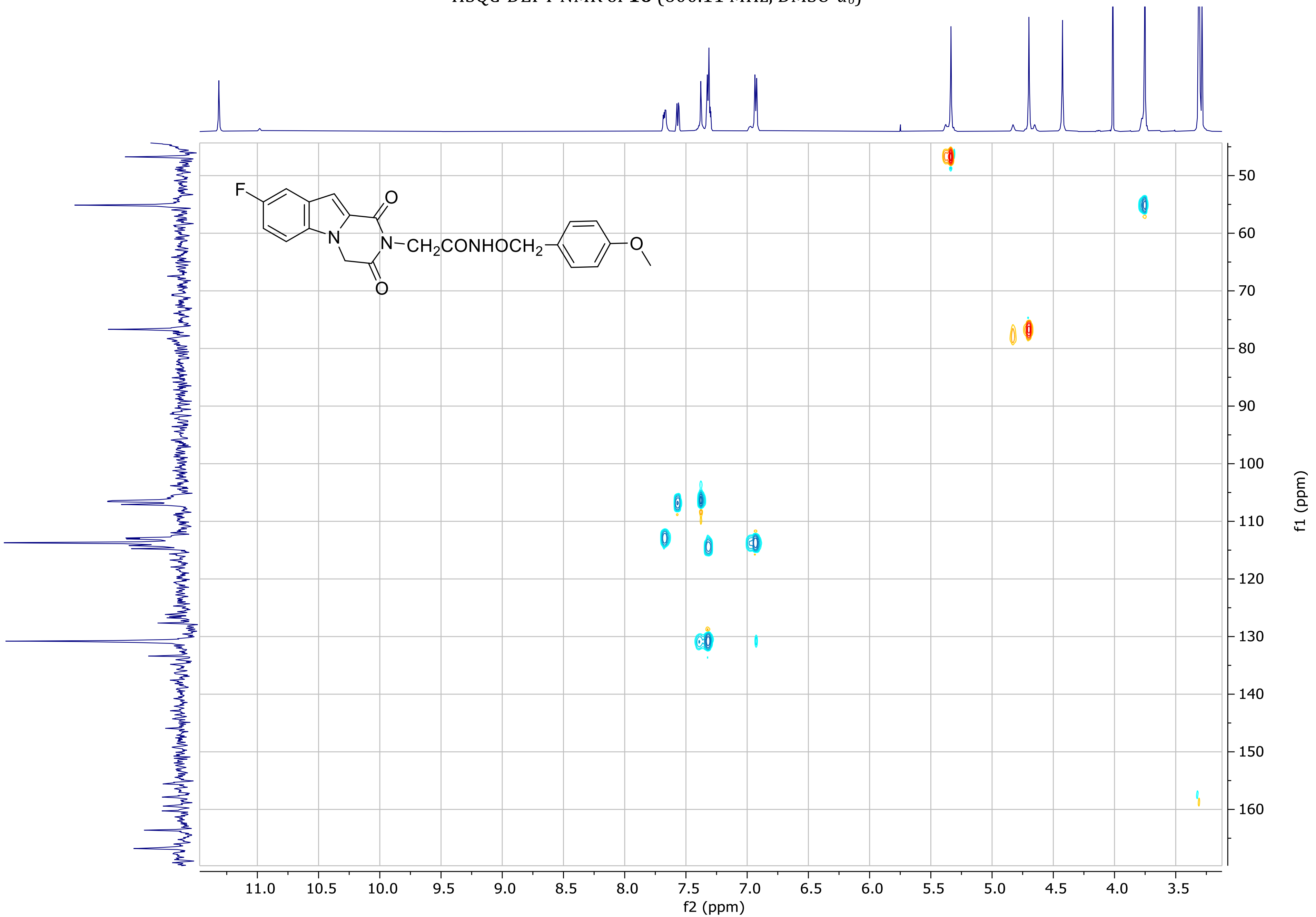

HMBC NMR of **16** (600.11 MHz, DMSO-d_6)

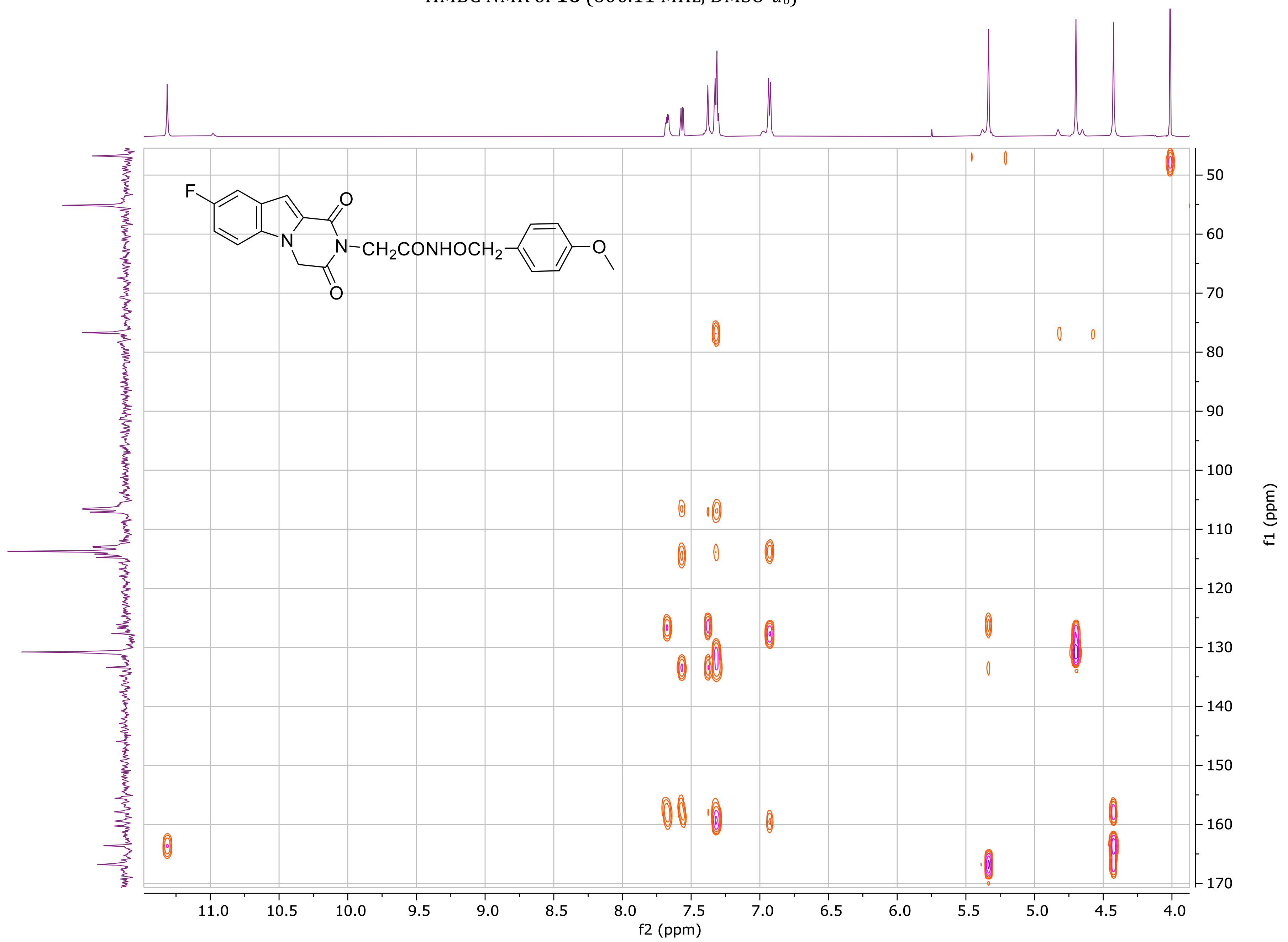

^{1}H NMR of **51** (400.13 MHz, DMSO-d_6)

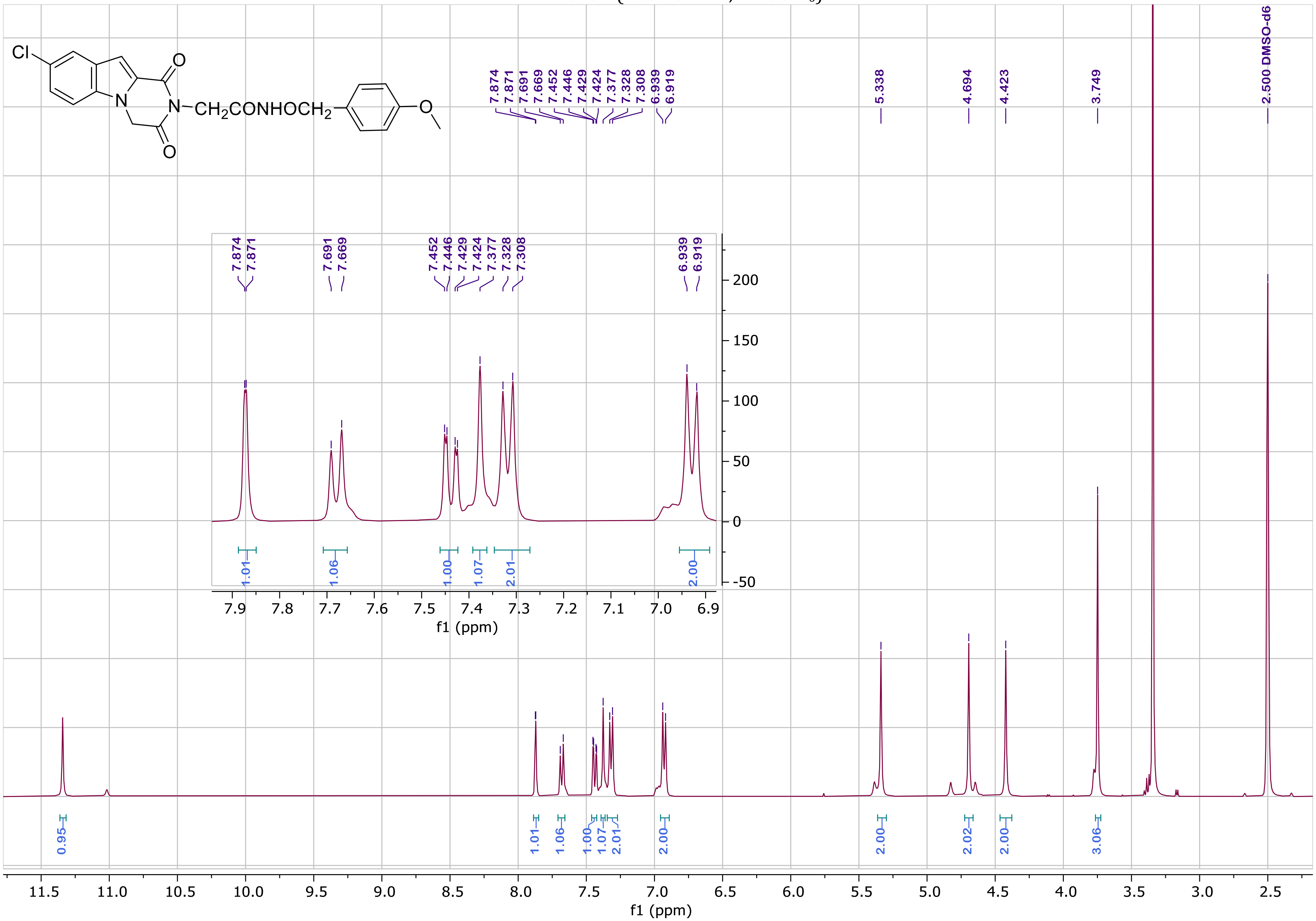

^{13}C NMR of **51** (50.32 MHz, DMSO-d_6)

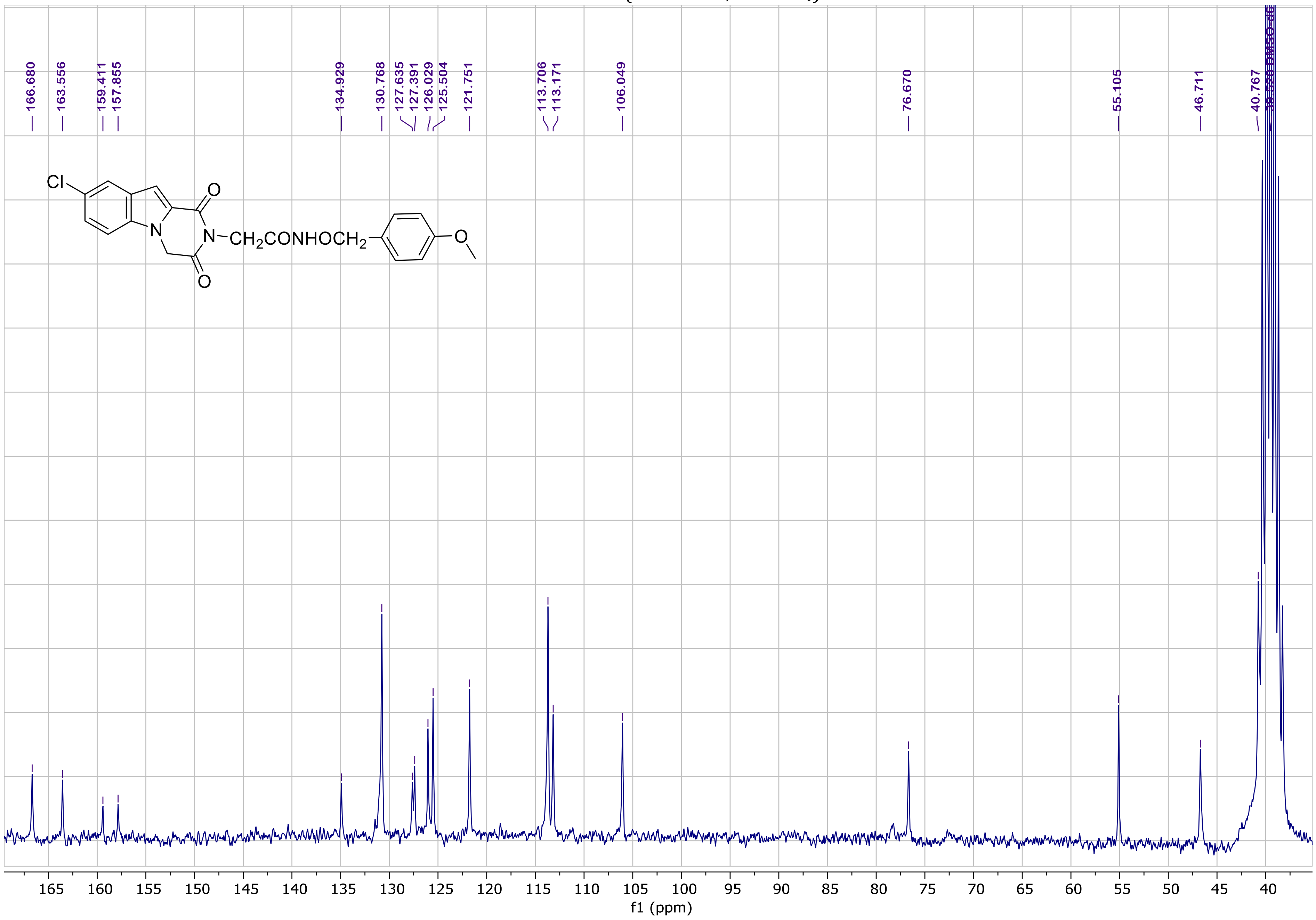

COSY NMR of **51** (400.13 MHz, DMSO-d_6)

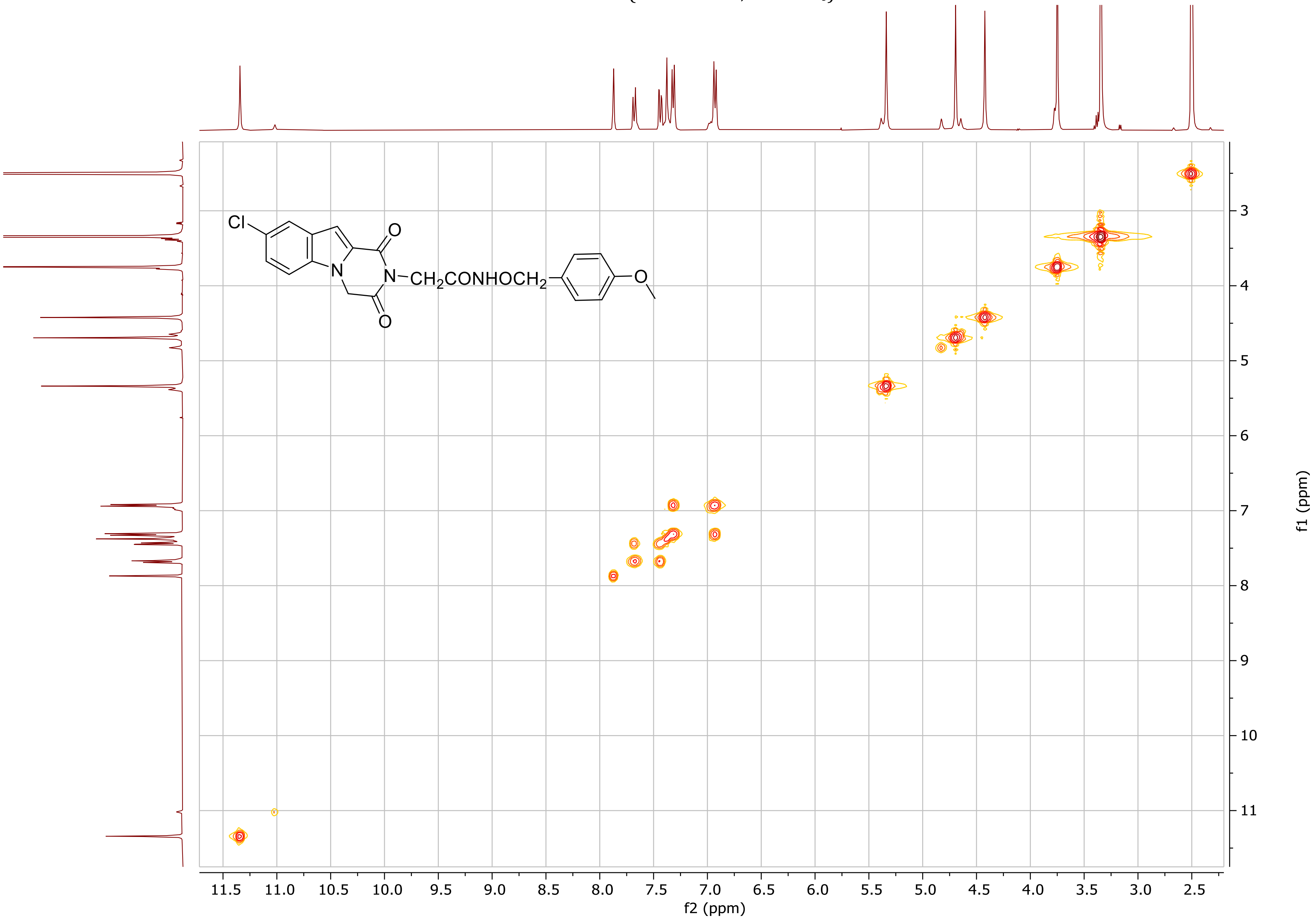

HSQC NMR of **51** (400.13 MHz, DMSO-d_6)

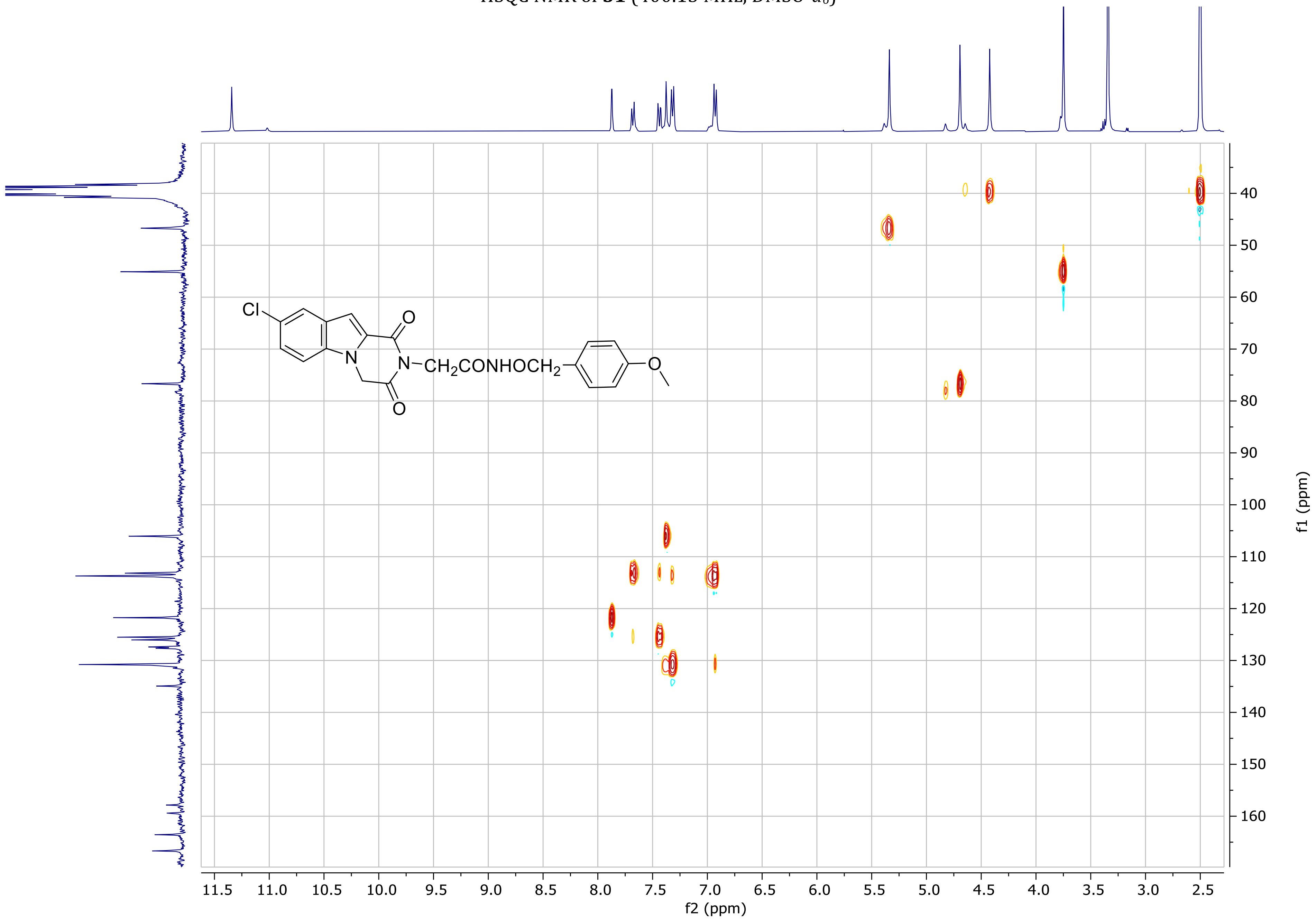

HMBC NMR of **51** (400.13 MHz, DMSO-d_6)

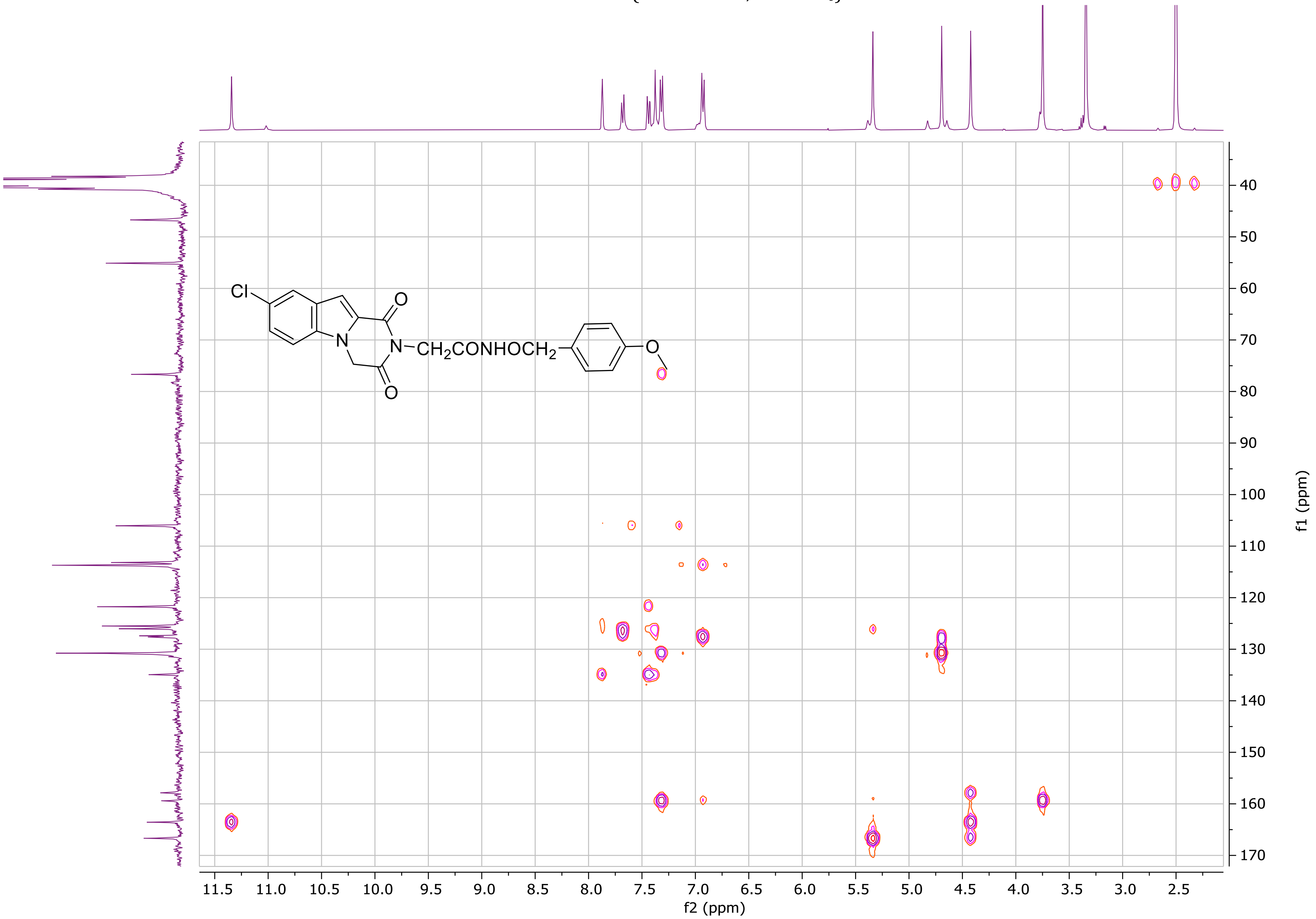

^{1}H NMR of **44** (600.11 MHz, DMSO-d_6)

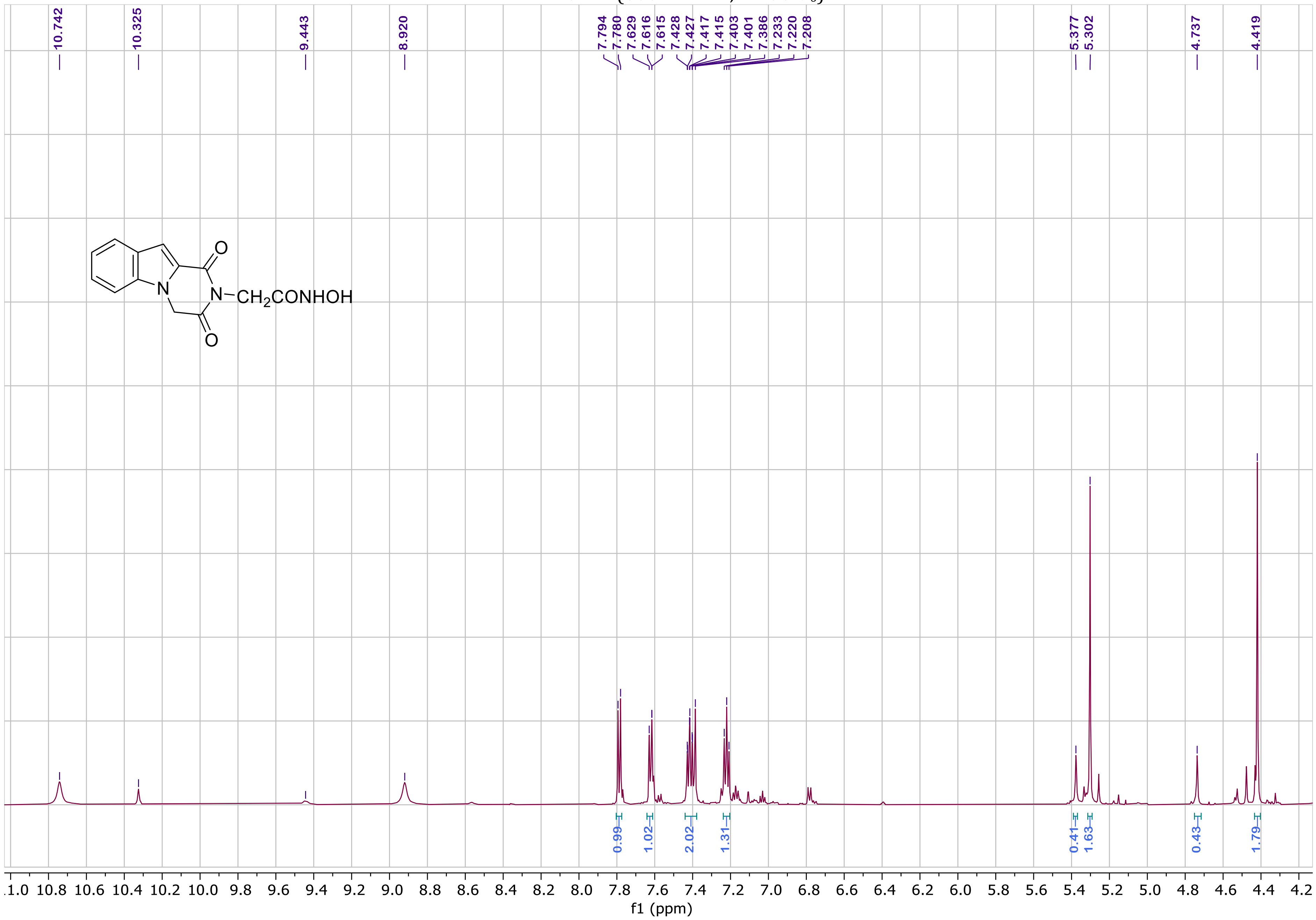

^{13}C NMR of **44** (50.32 MHz, DMSO-d_6)

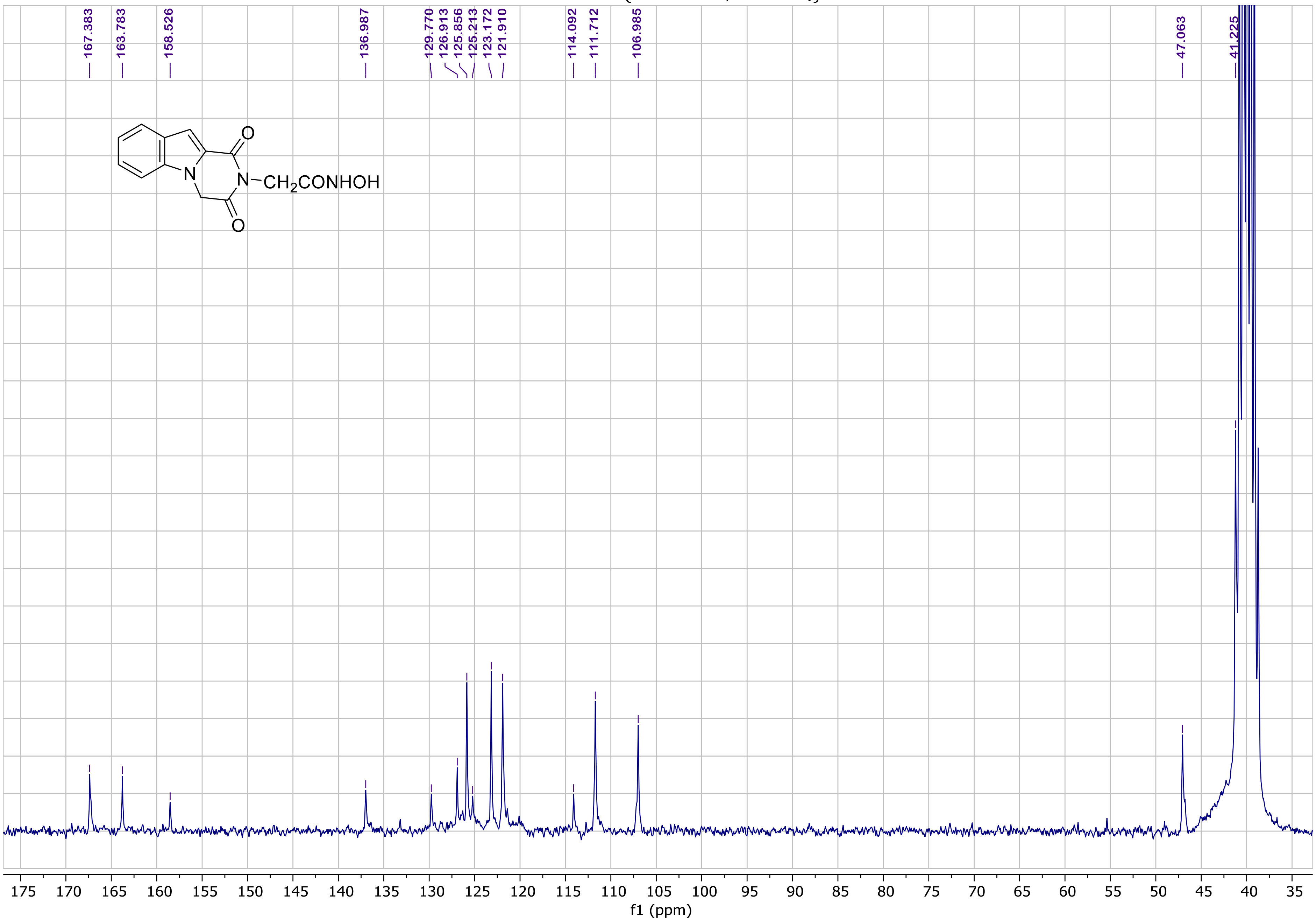

DEPT NMR of **44** (50.32 MHz, DMSO-d_6)

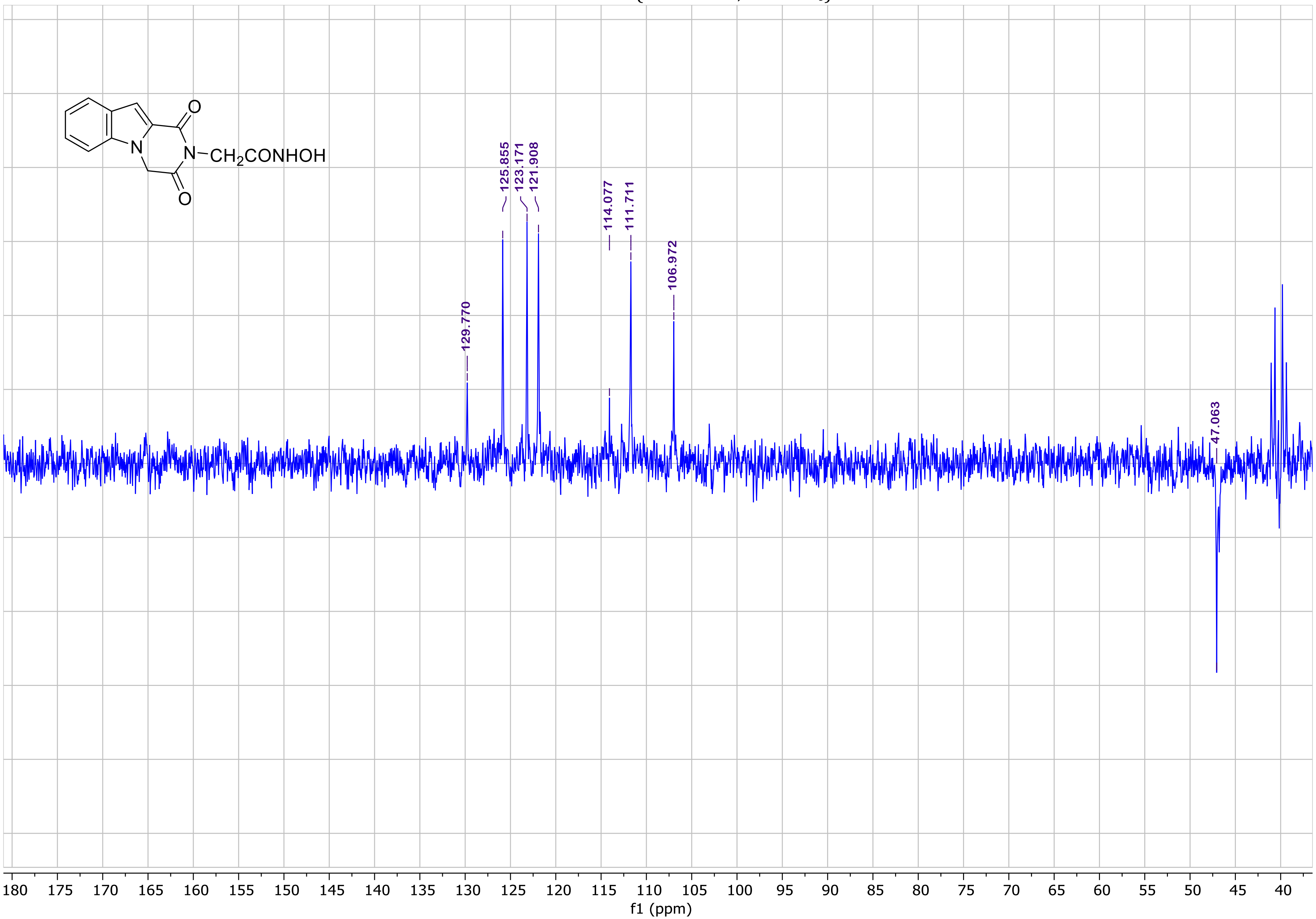

COSY NMR of **44** (600.11 MHz, DMSO-d_6)

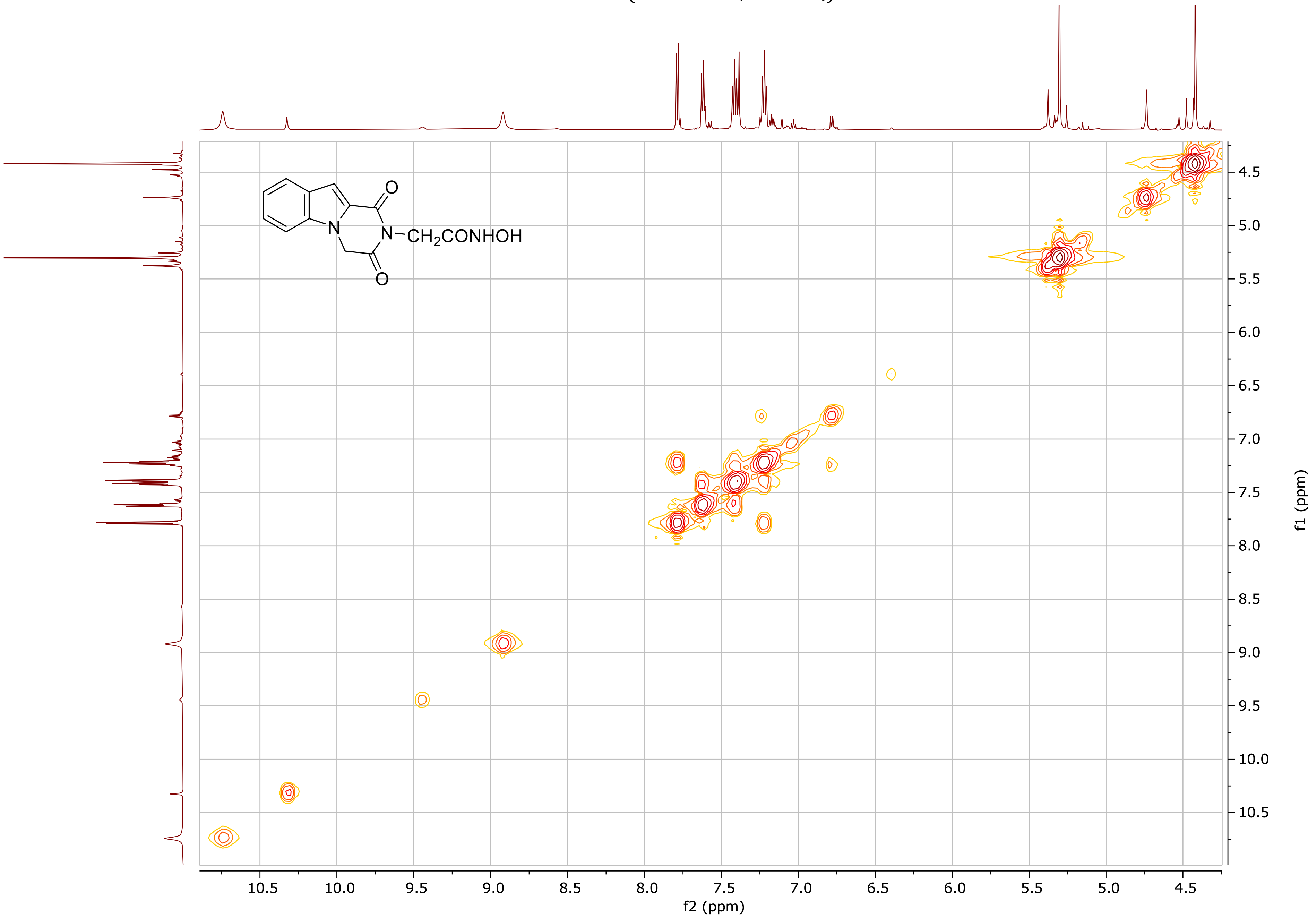

HSQC-DEPT NMR of **44** (600.11 MHz, DMSO-d_6)

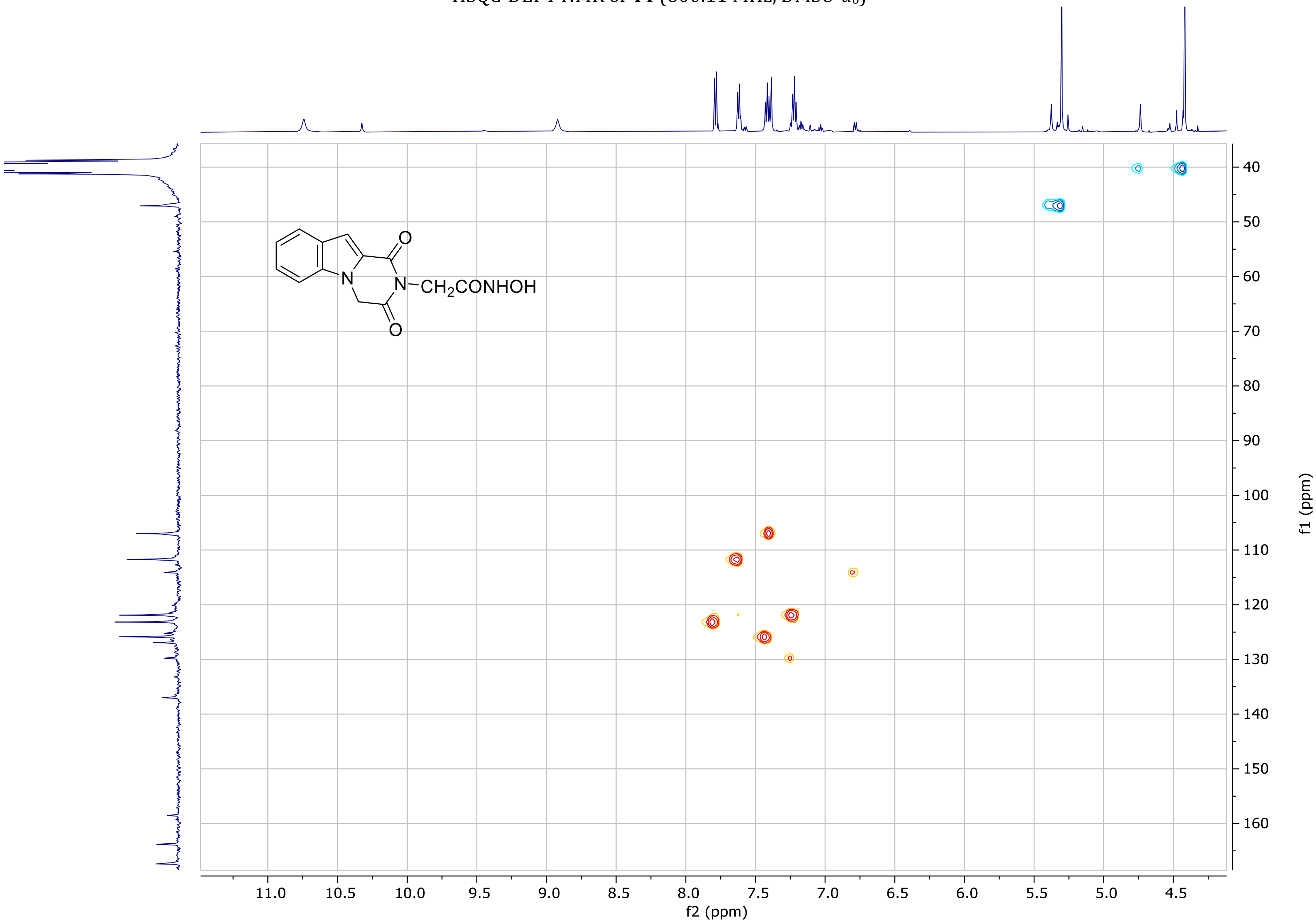

HMBC NMR of **44** (600.11 MHz, DMSO-d_6)

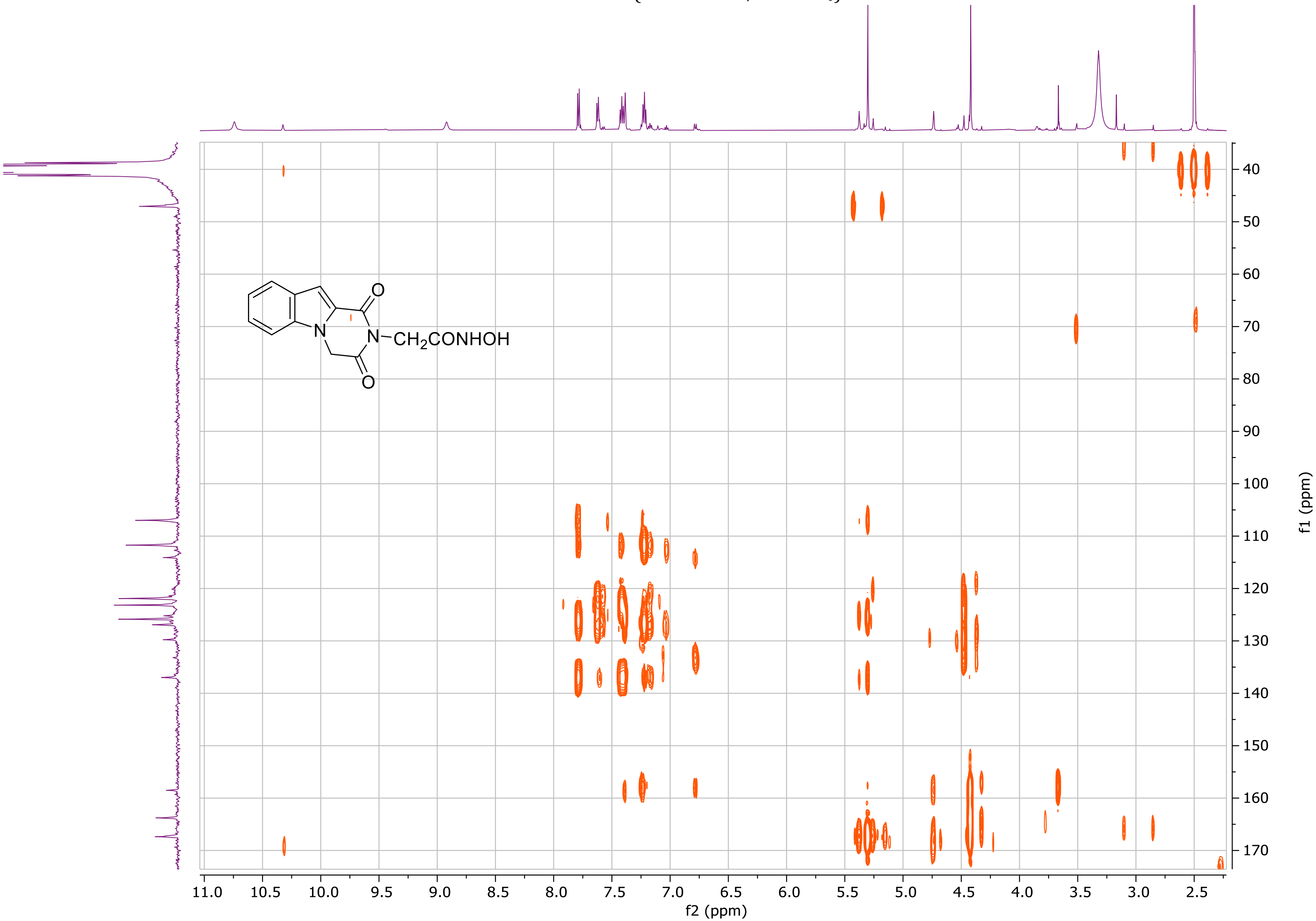

^{1}H NMR of **48** (600.11 MHz, DMSO-d_6)

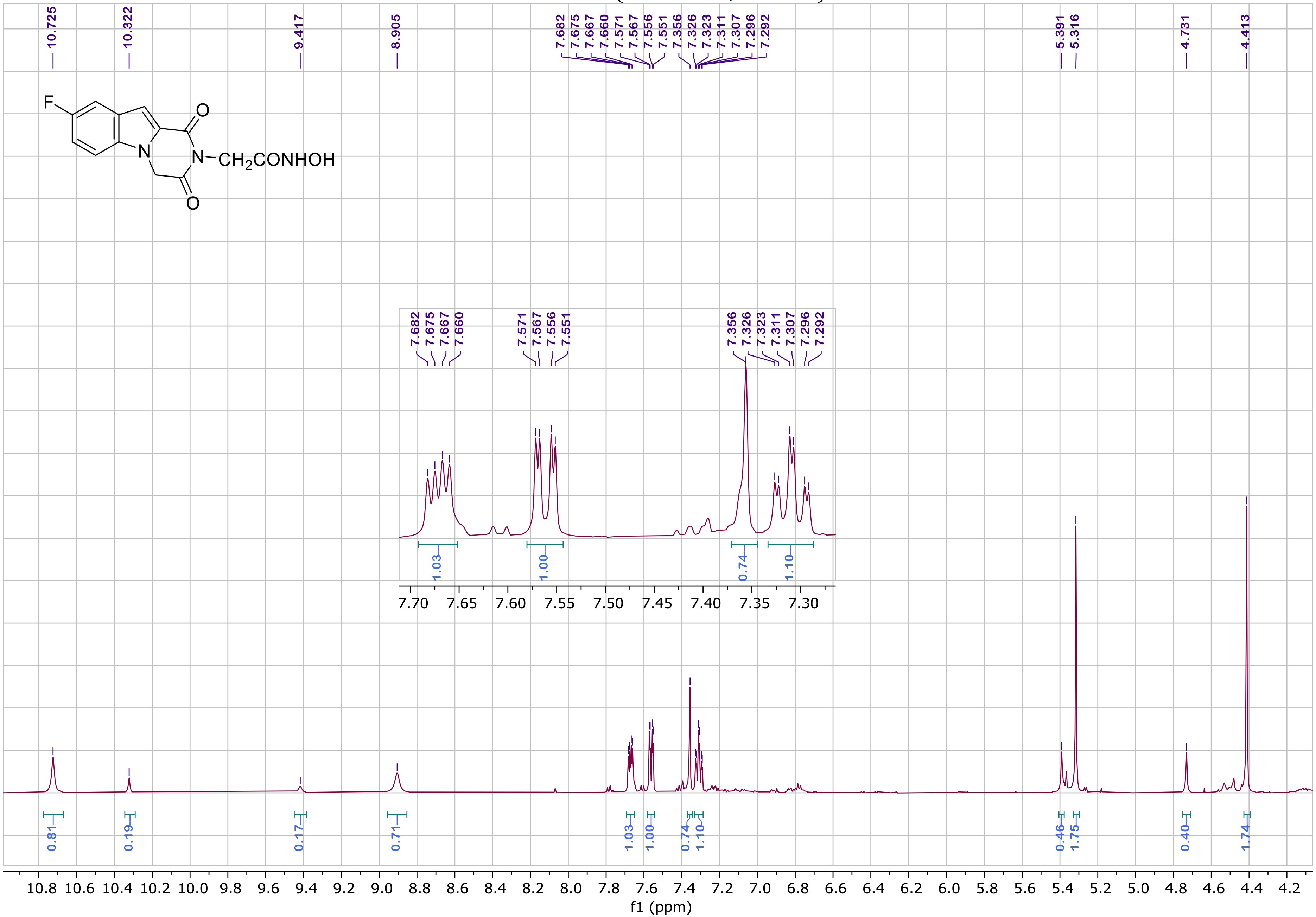

^{13}C NMR of **48** (50.32 MHz, DMSO-d_6)

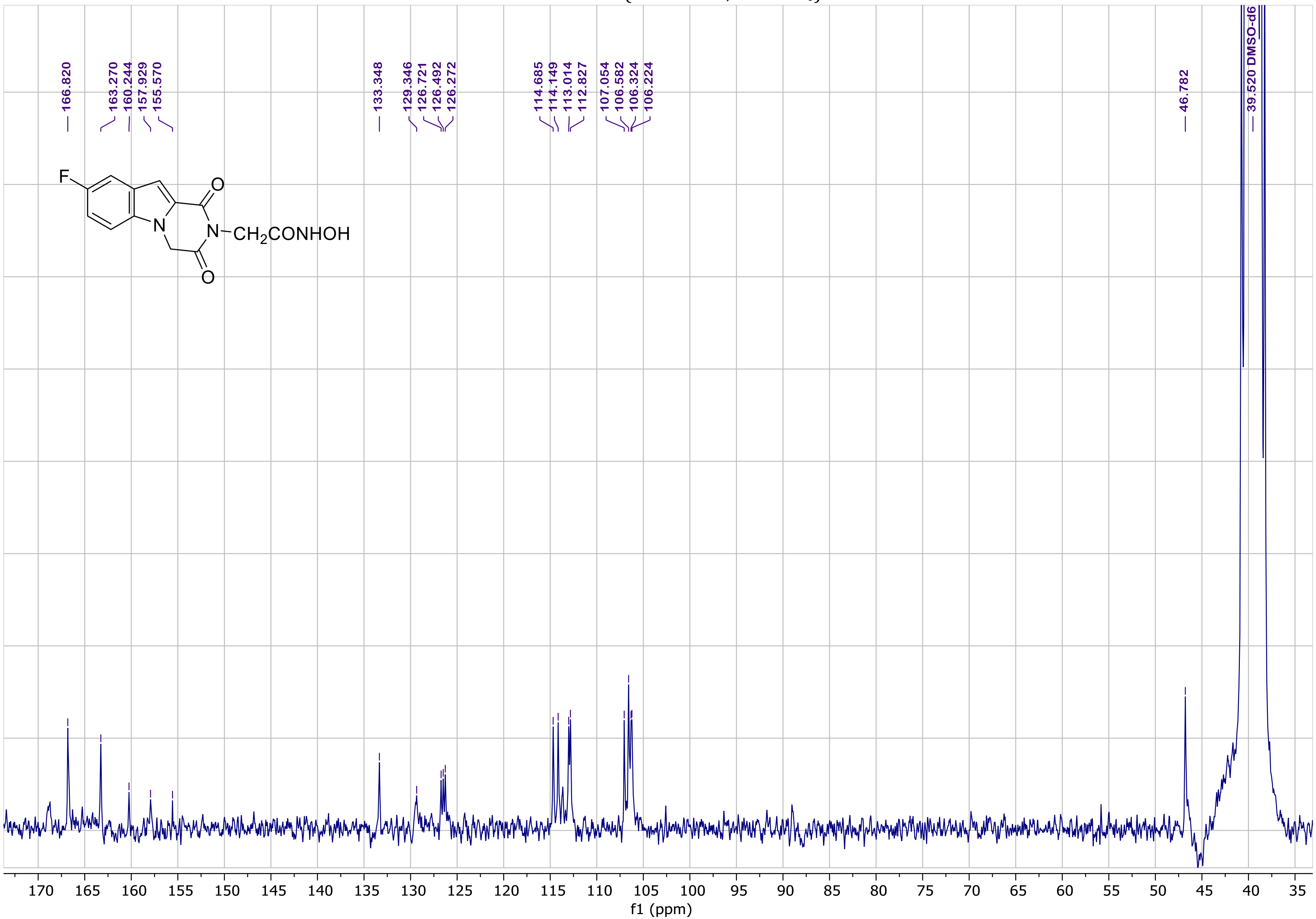

DEPT NMR of **48** (50.32 MHz, DMSO-d_6)

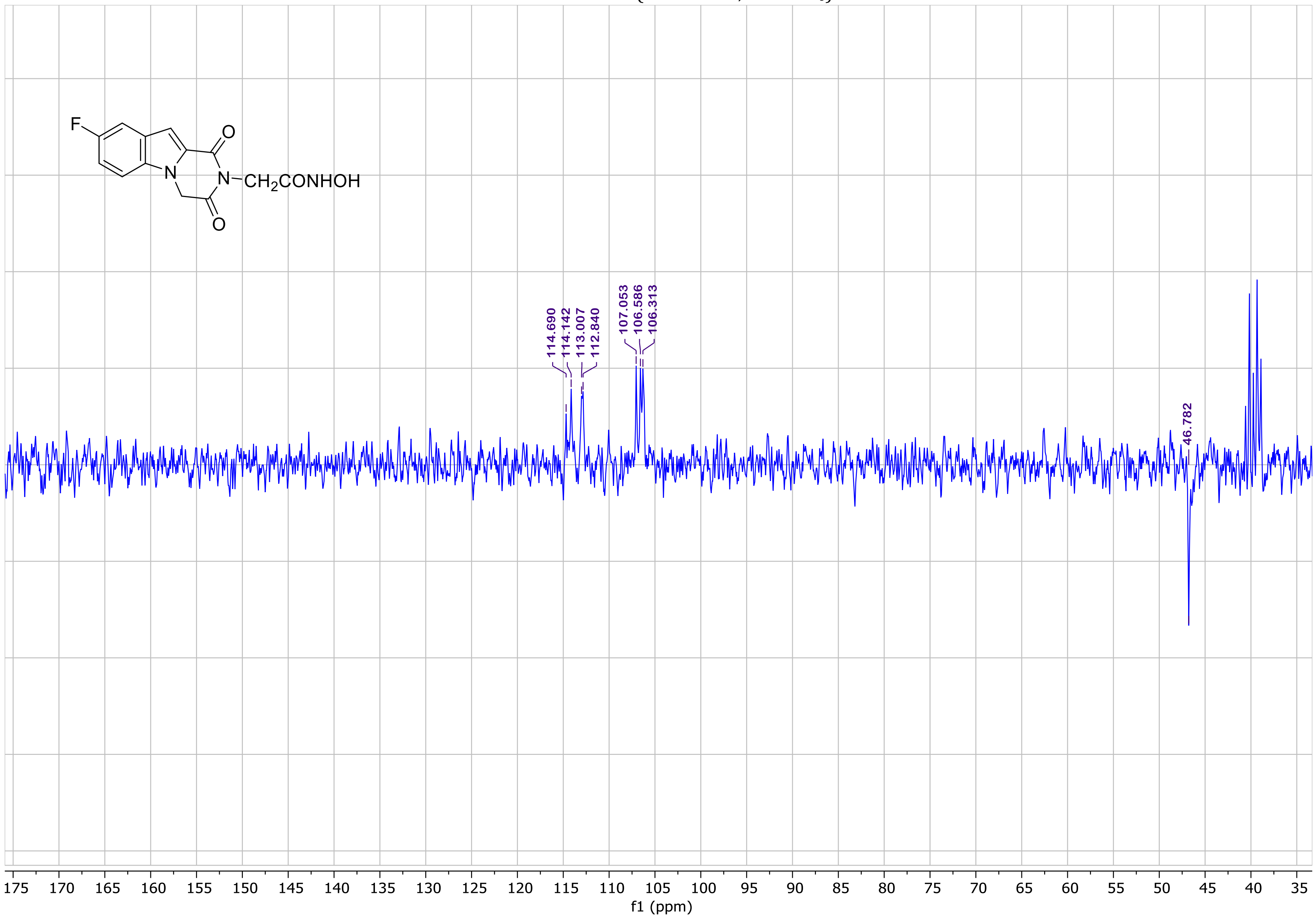

COSY NMR of **48** (600.11 MHz, DMSO-d_6)

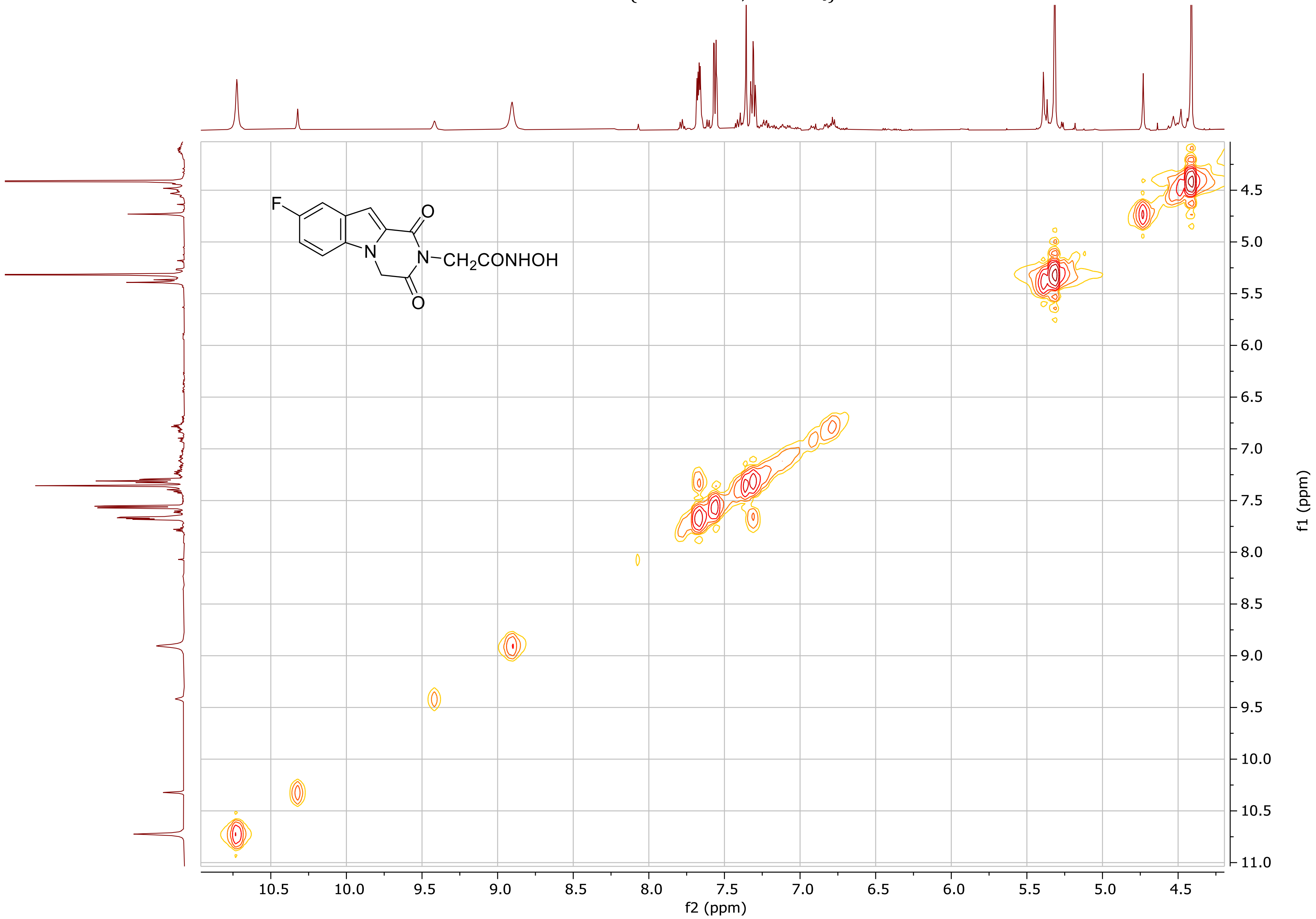

HSQC-DEPT NMR of **48** (600.11 MHz, DMSO-d_6)

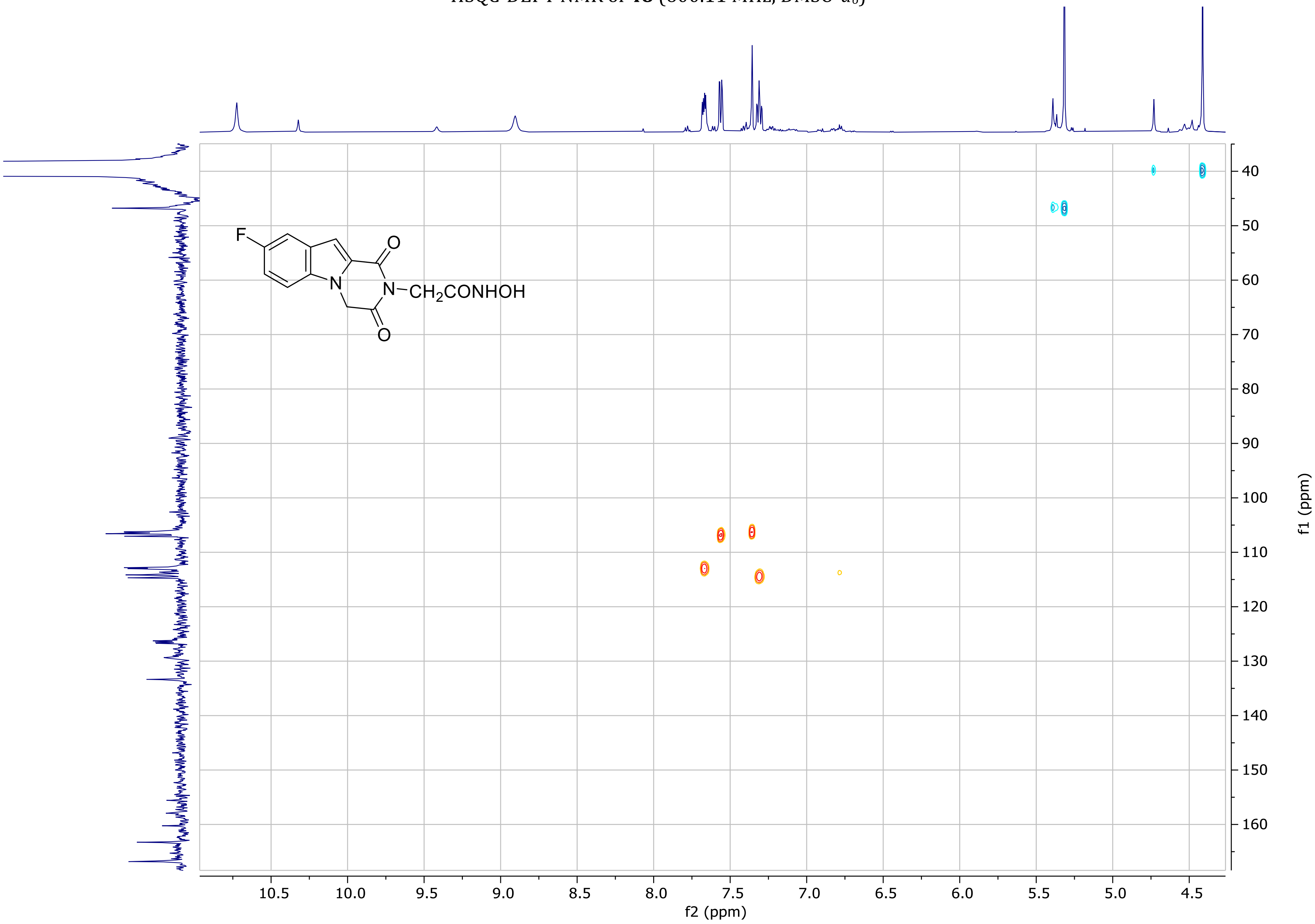

HMBC NMR of **48** (600.11 MHz, DMSO-d_6)

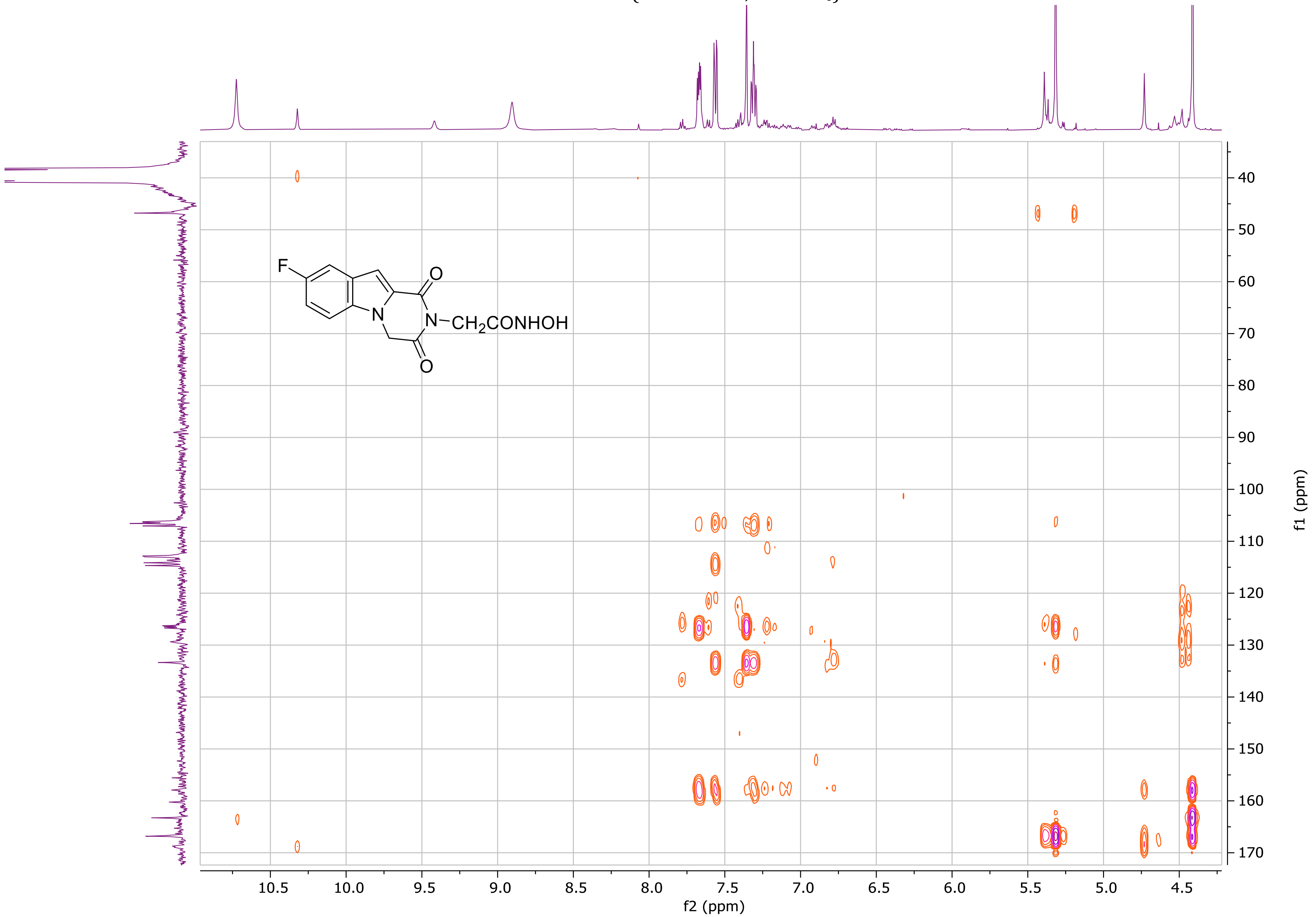

^{1}H NMR of **52** (600.11 MHz, DMSO-d_6)

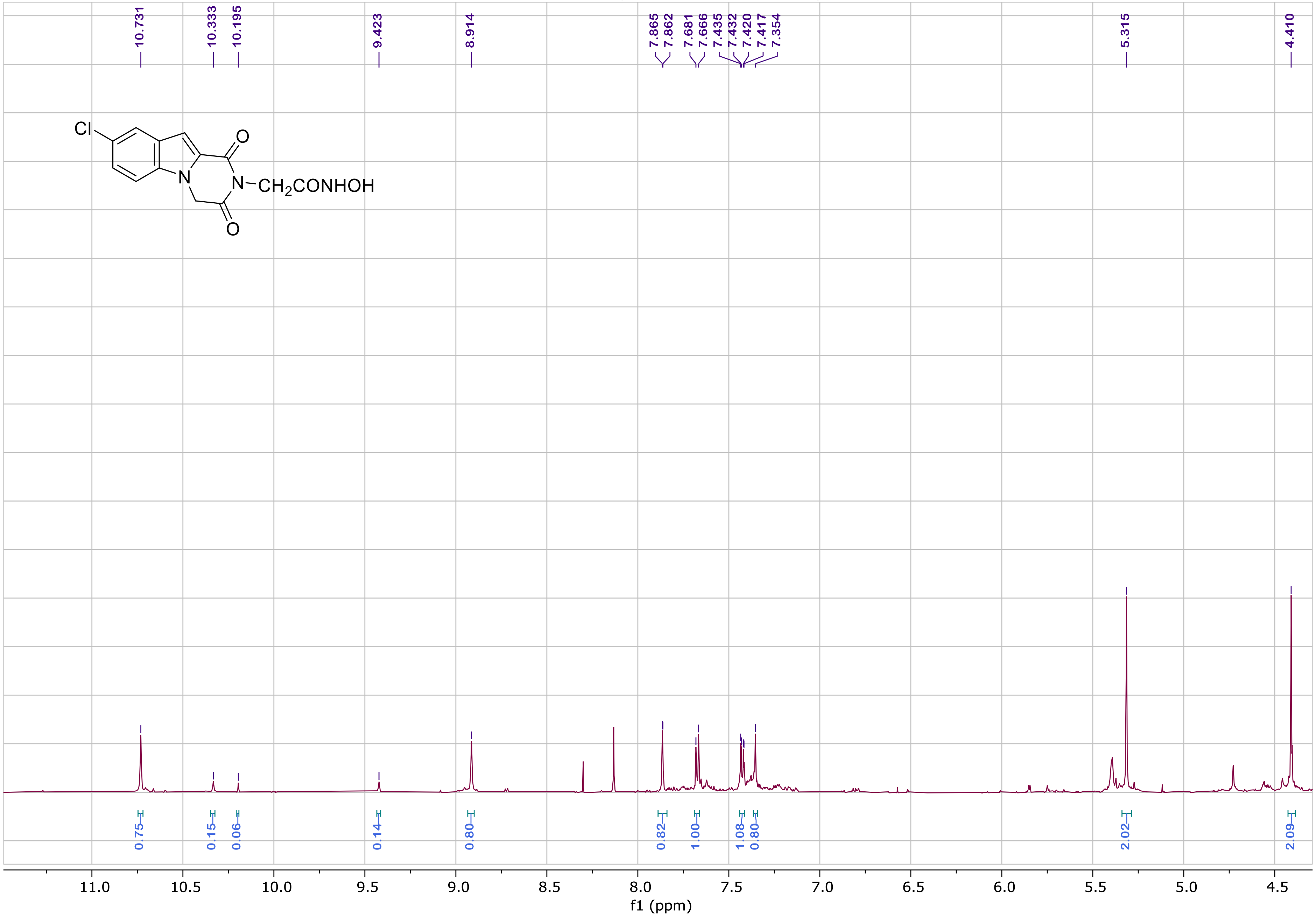

^{13}C NMR of **52** (50.32 MHz, DMSO-d_6)

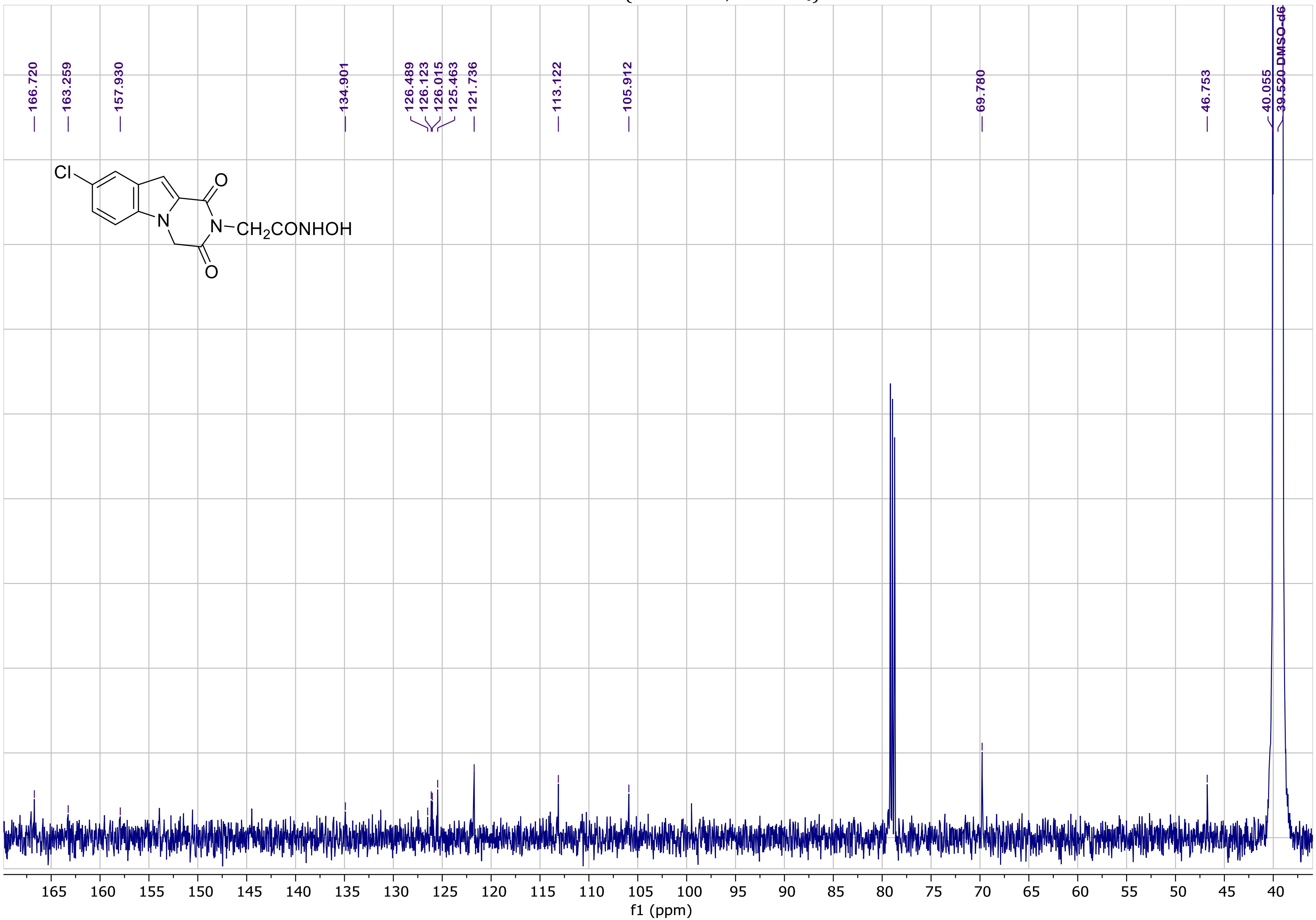

DEPT NMR of **48** (50.32 MHz, DMSO-d_6)

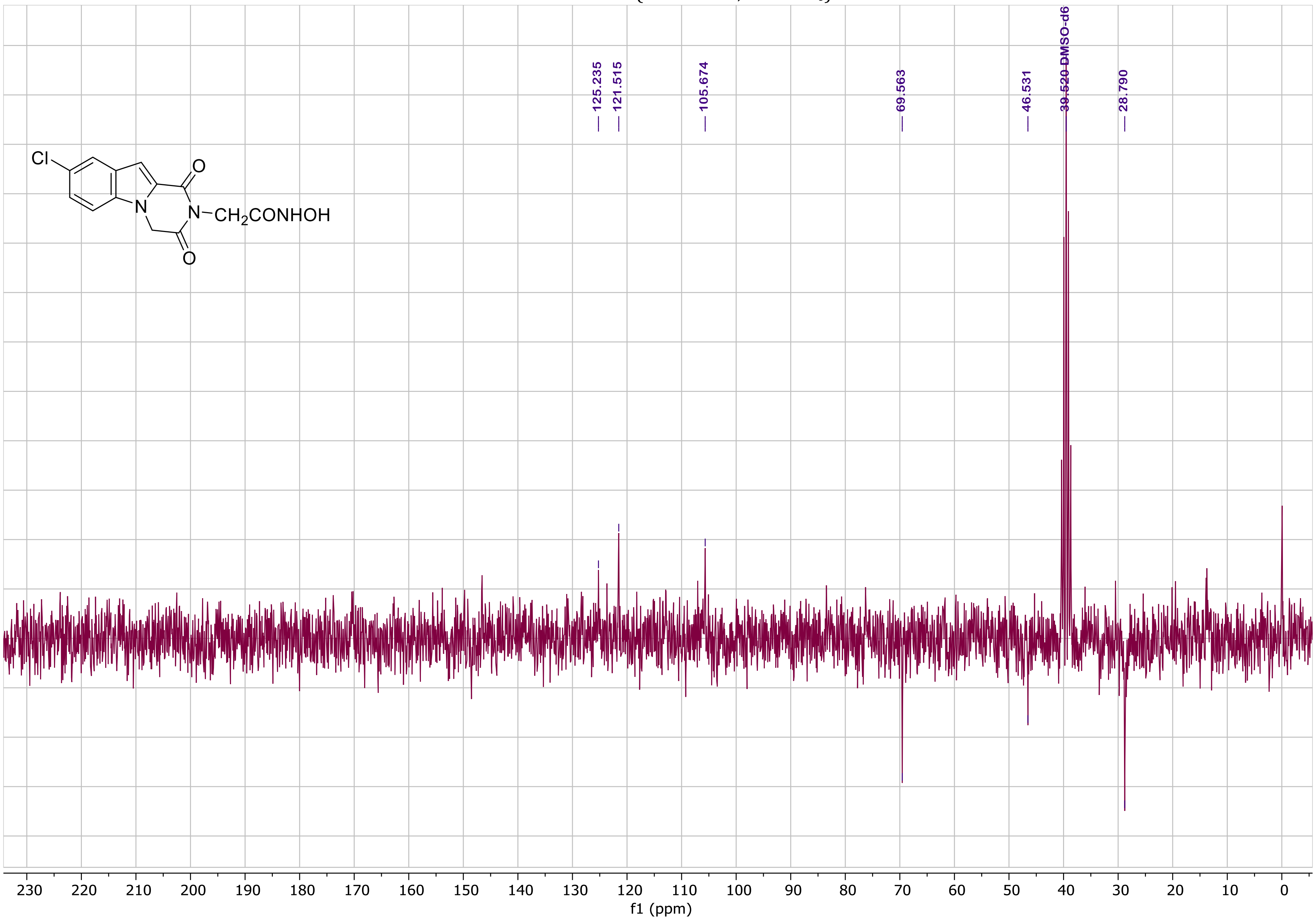

COSY NMR of **52** (600.11 MHz, DMSO-d_6)

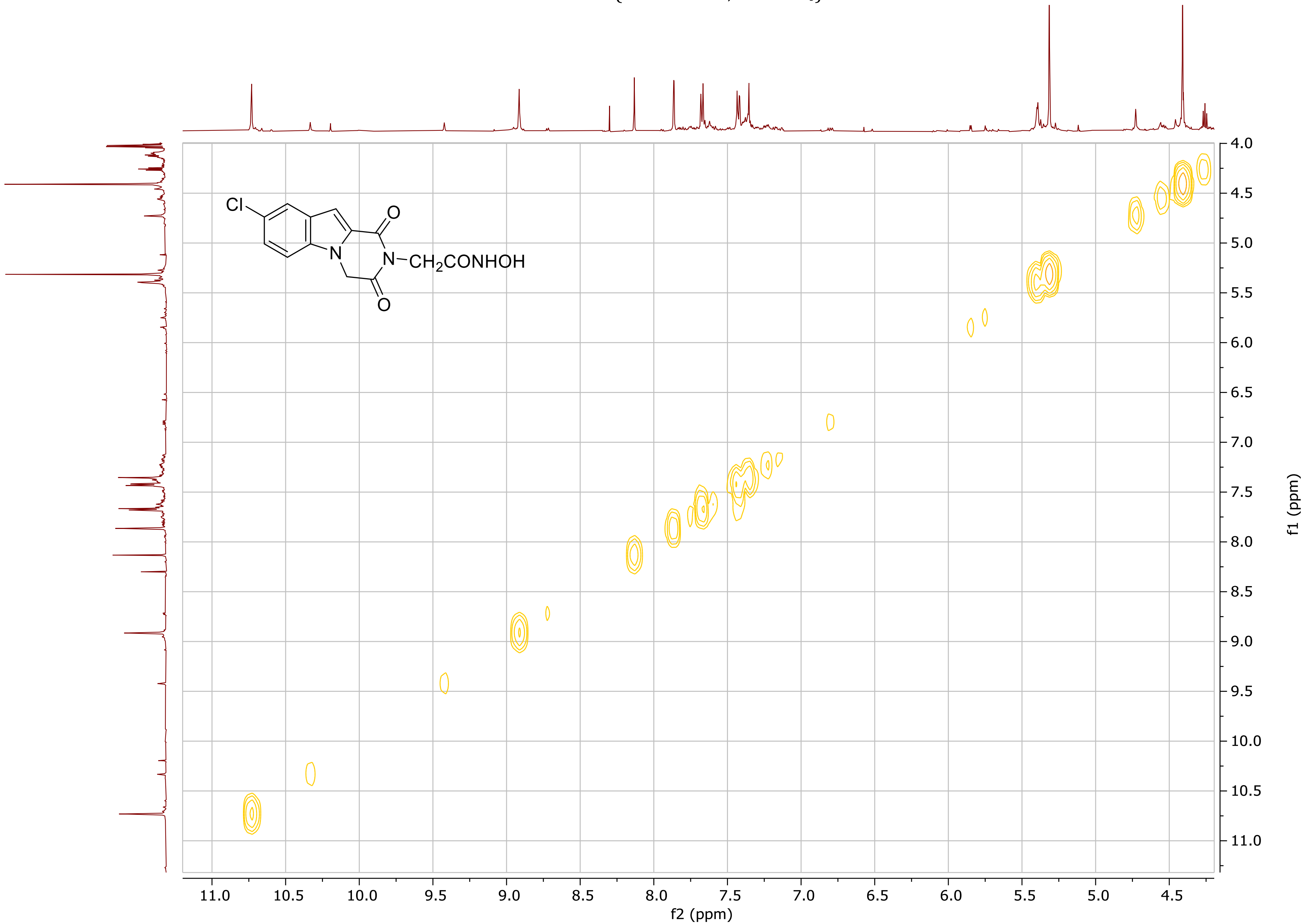

HSQC-DEPT NMR of **52** (600.11 MHz, DMSO-d_6)

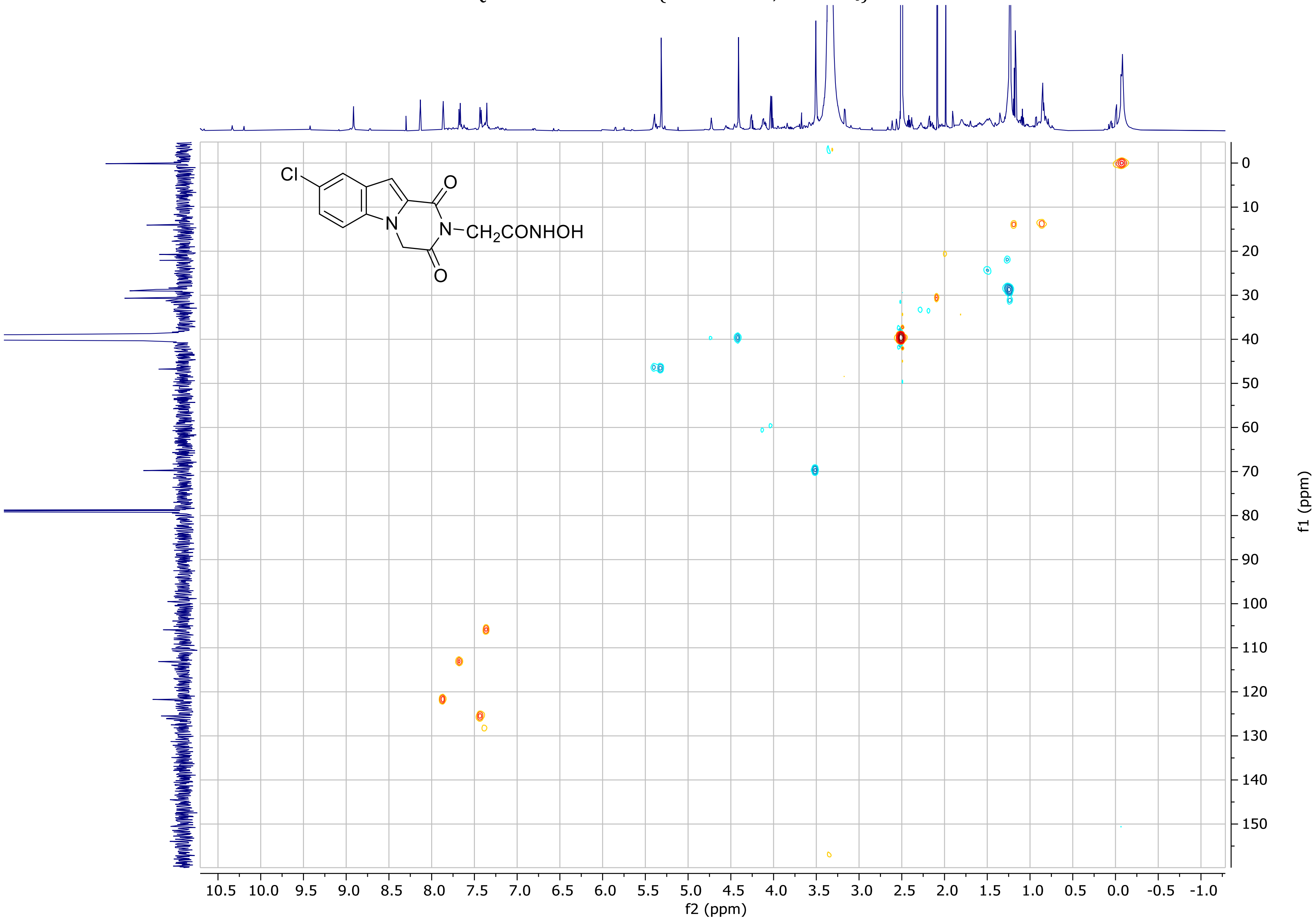

HMBC NMR of **52** (600.11 MHz, DMSO-d_6)

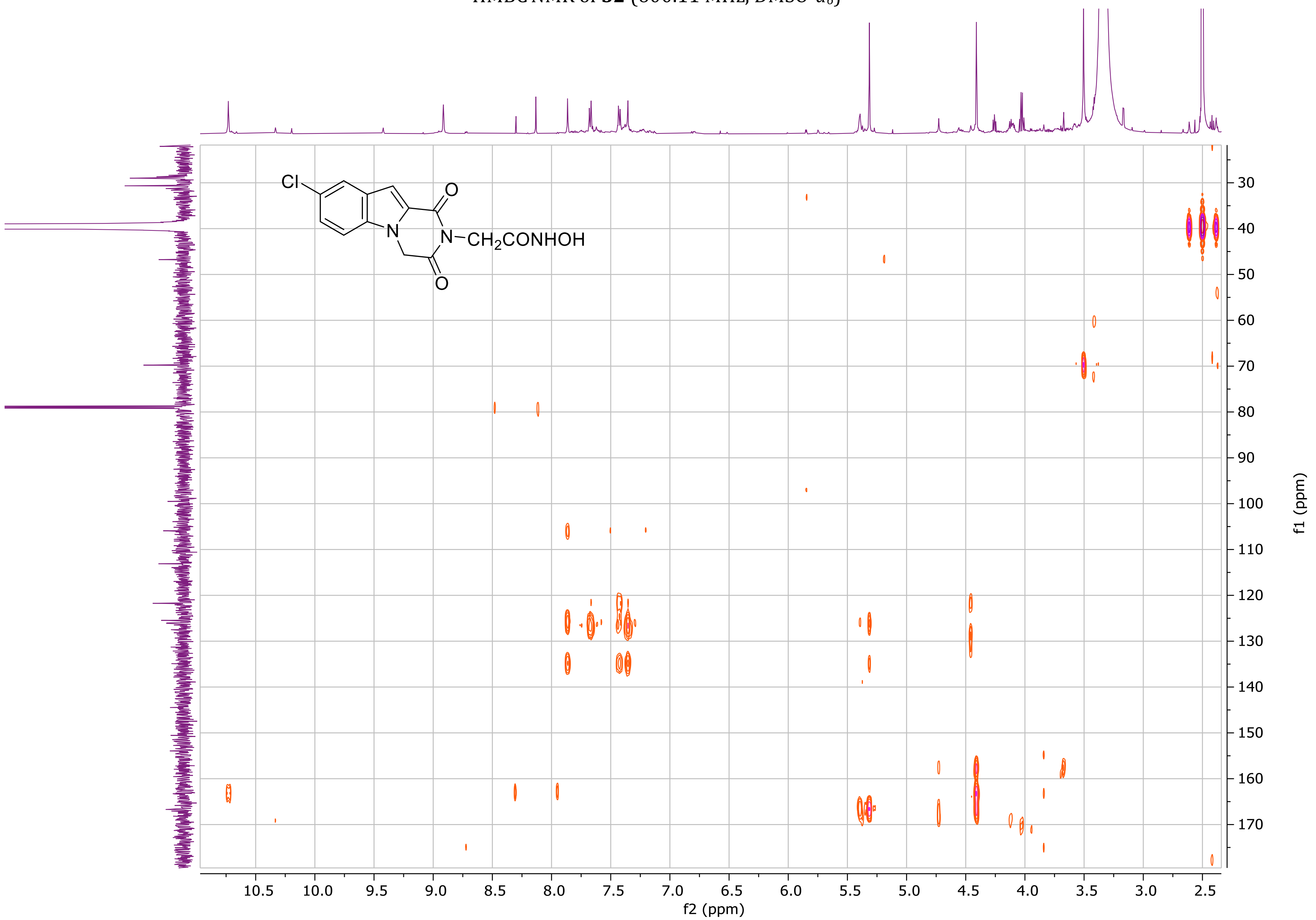

^{1}H NMR of **54** (600.11 MHz, $CDCl_3$)

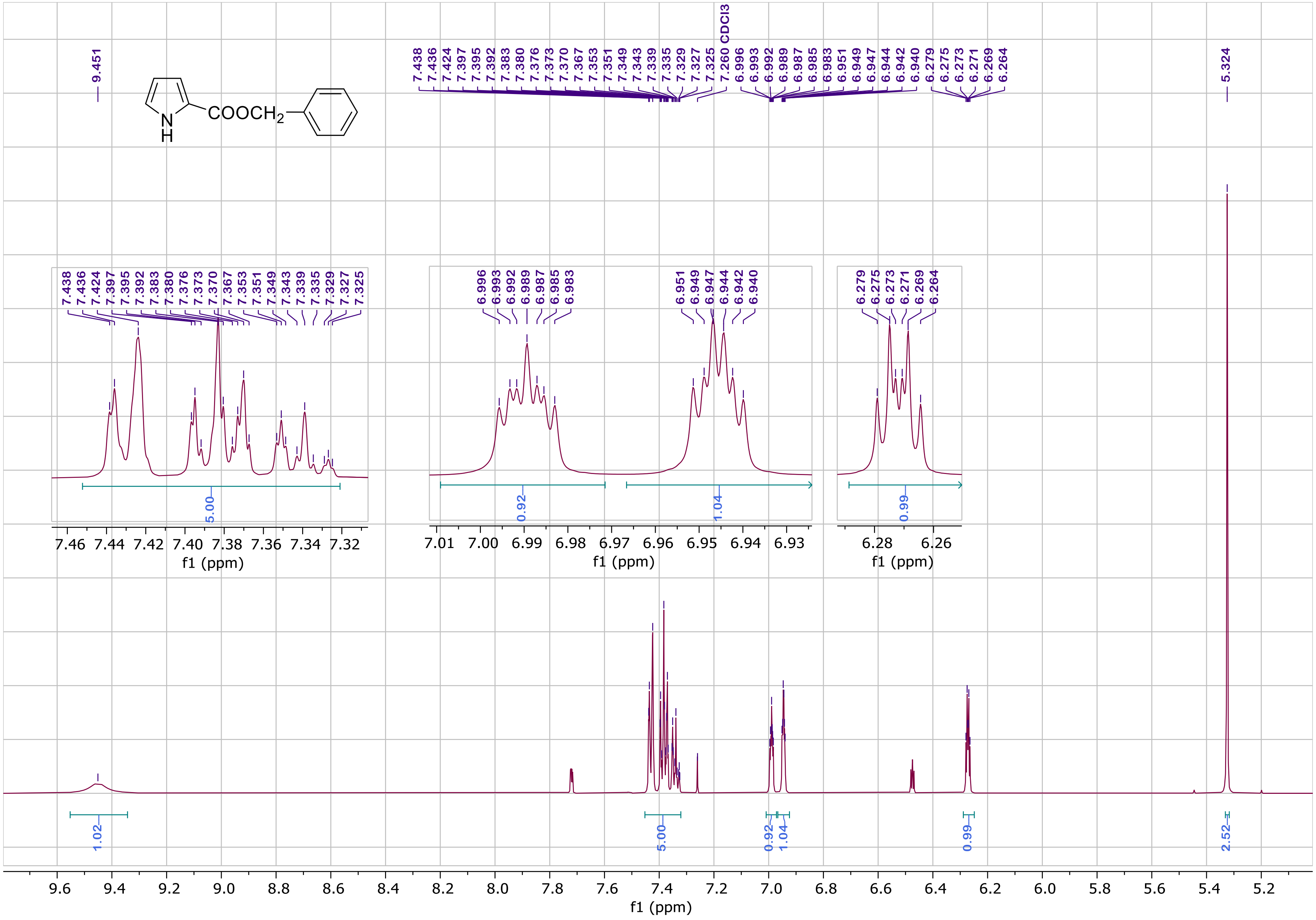

^{13}C NMR of **54** (50.32 MHz, $CDCl_3$)

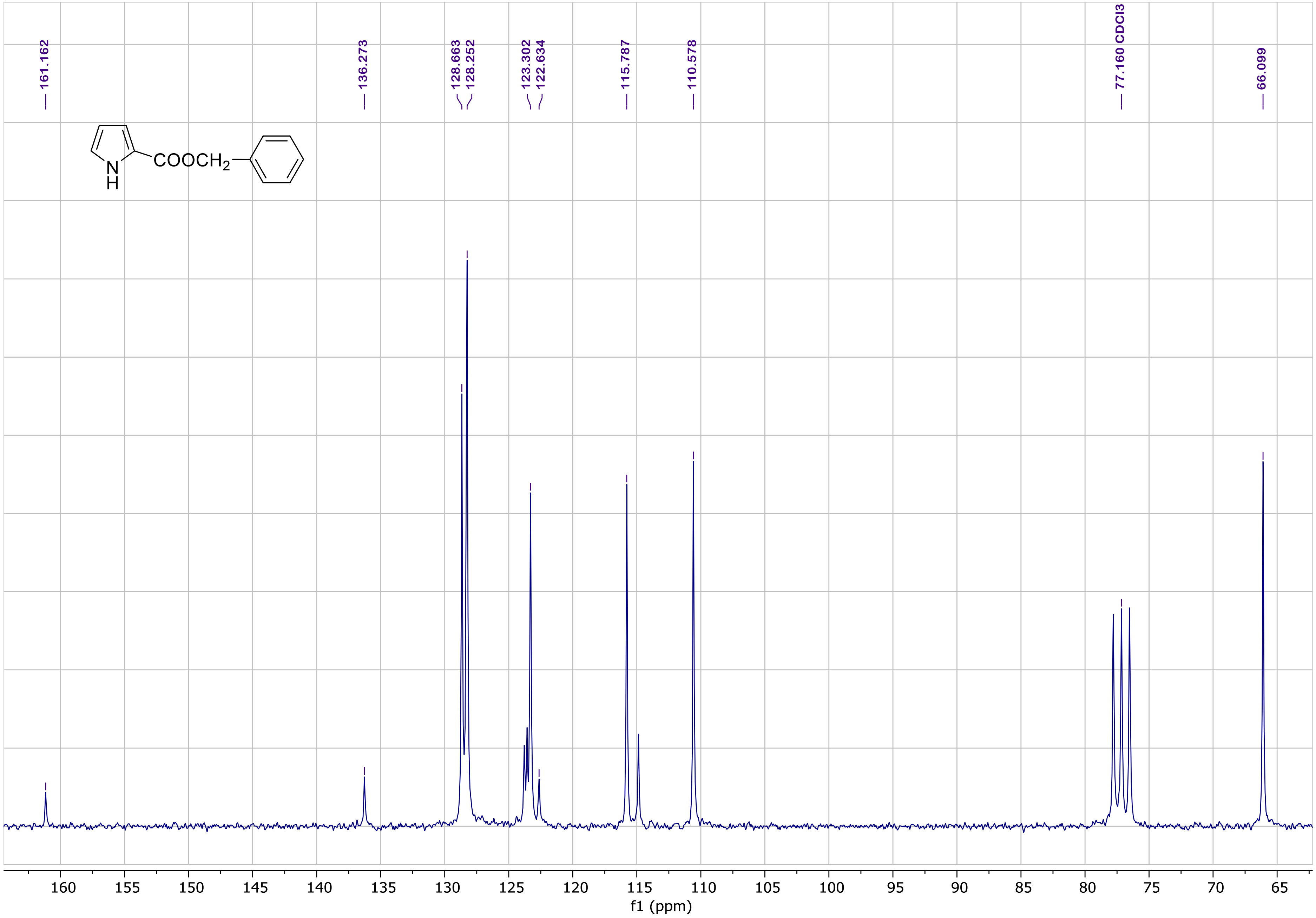

DEPT NMR of **54** (50.32 MHz, $CDCl_3$)

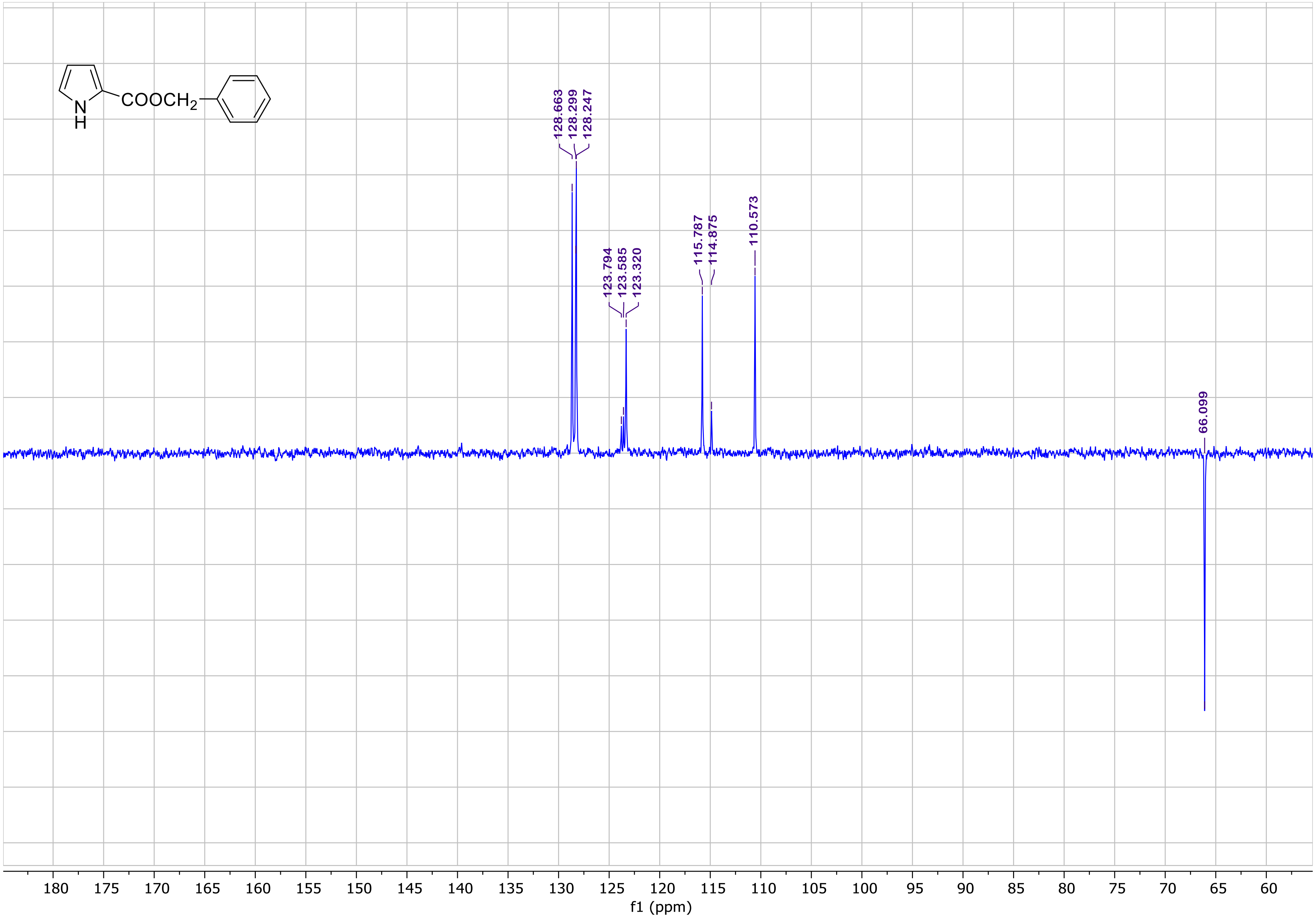

COSY NMR of **54** (600.11 MHz, $CDCl_3$)

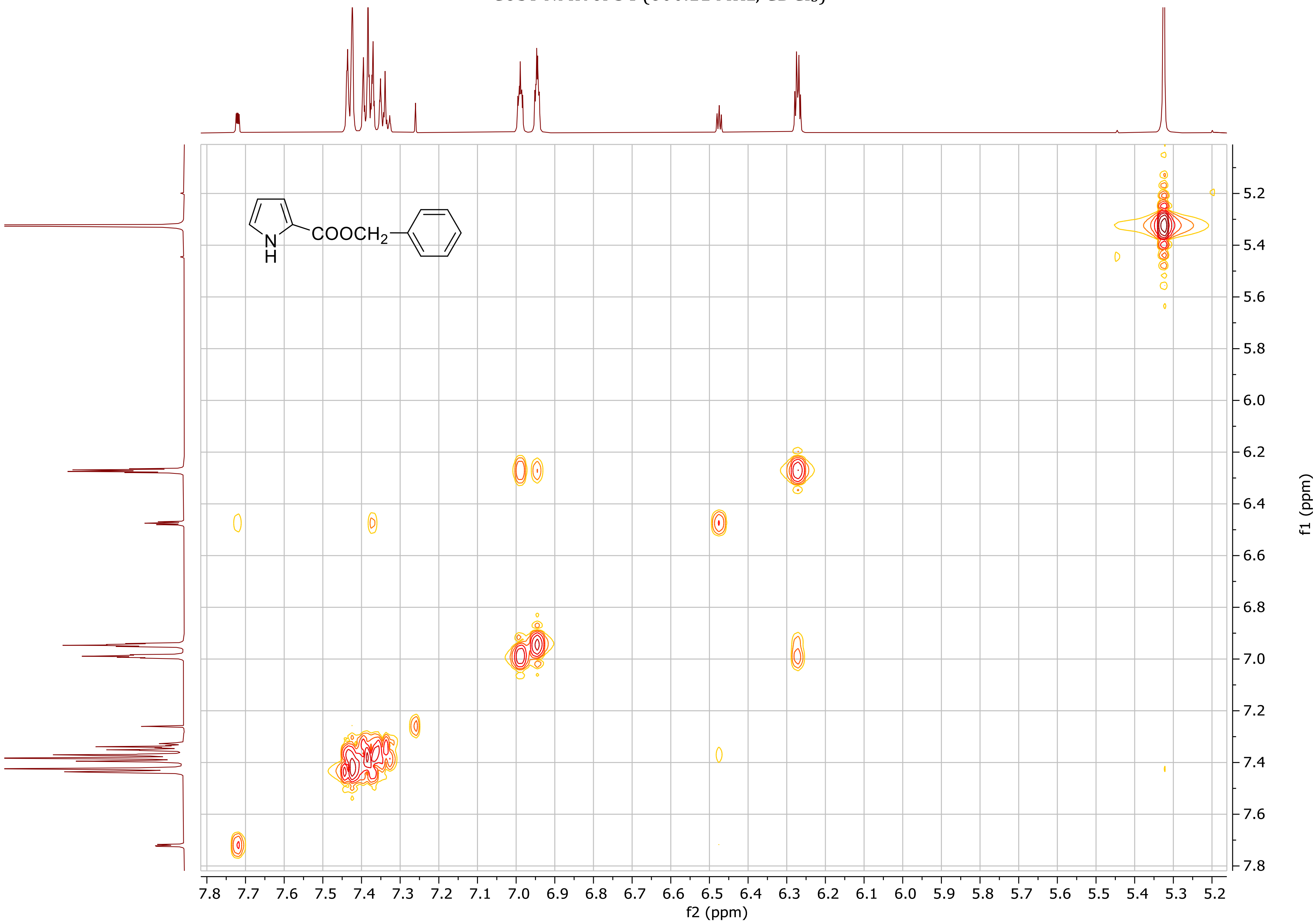

HSQC-DEPT NMR of **54** (600.11 MHz, $CDCl_3$)

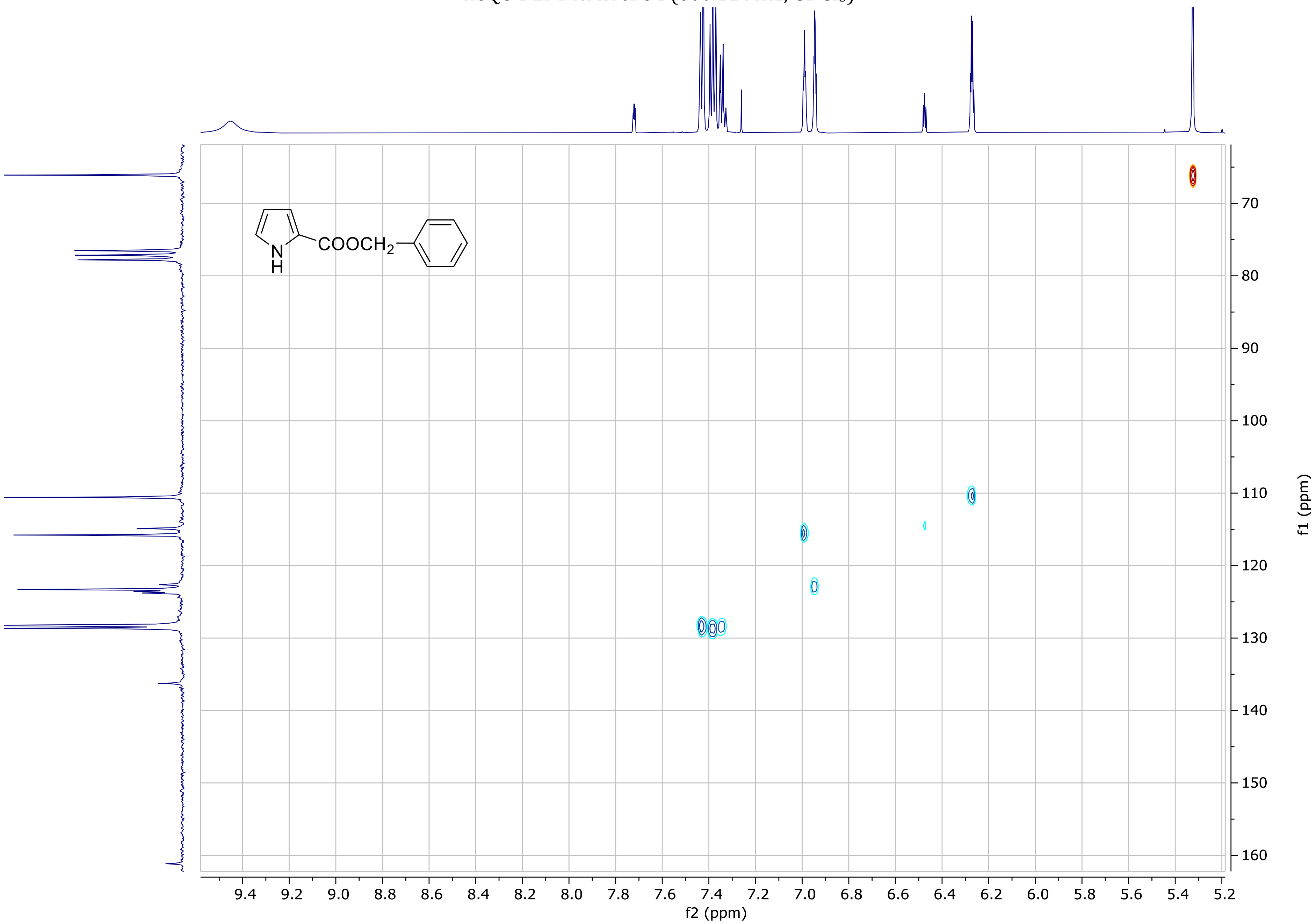

HMBC NMR of **54** (600.11 MHz, $CDCl_3$)

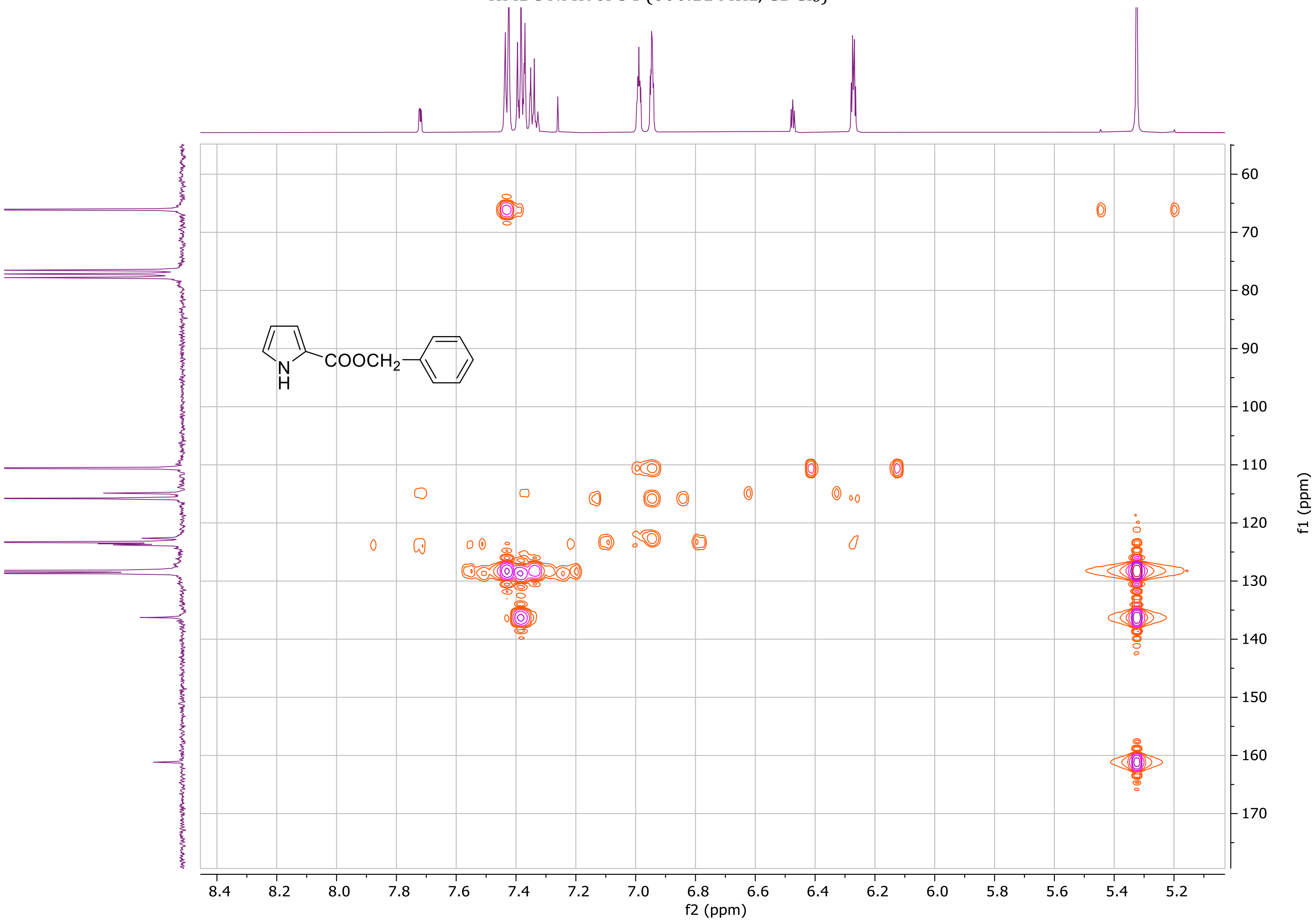

^{1}H NMR of **55** (600.11 MHz, $CDCl_3$)

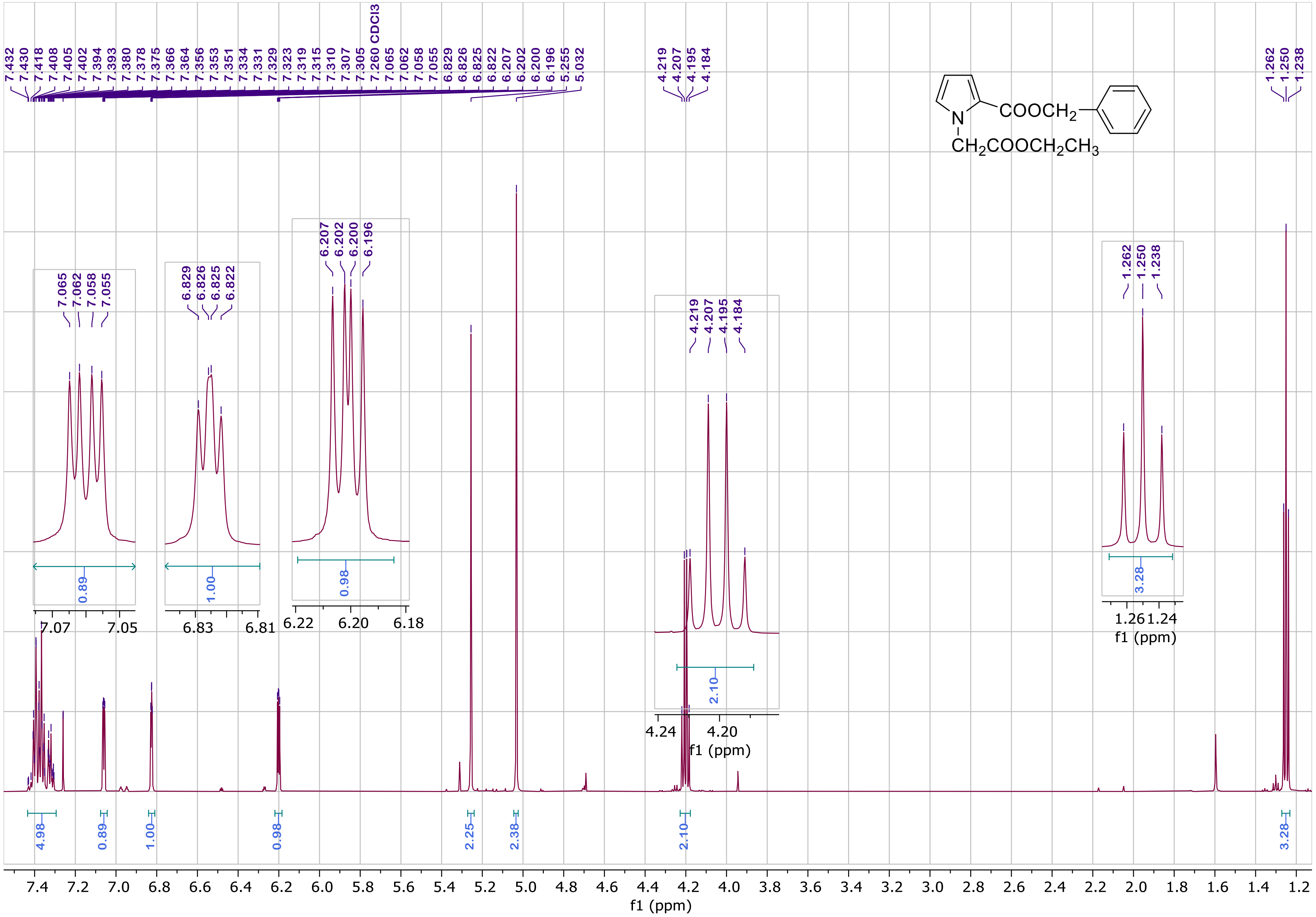

^{13}C NMR of **55** (50.32 MHz, $CDCl_3$)

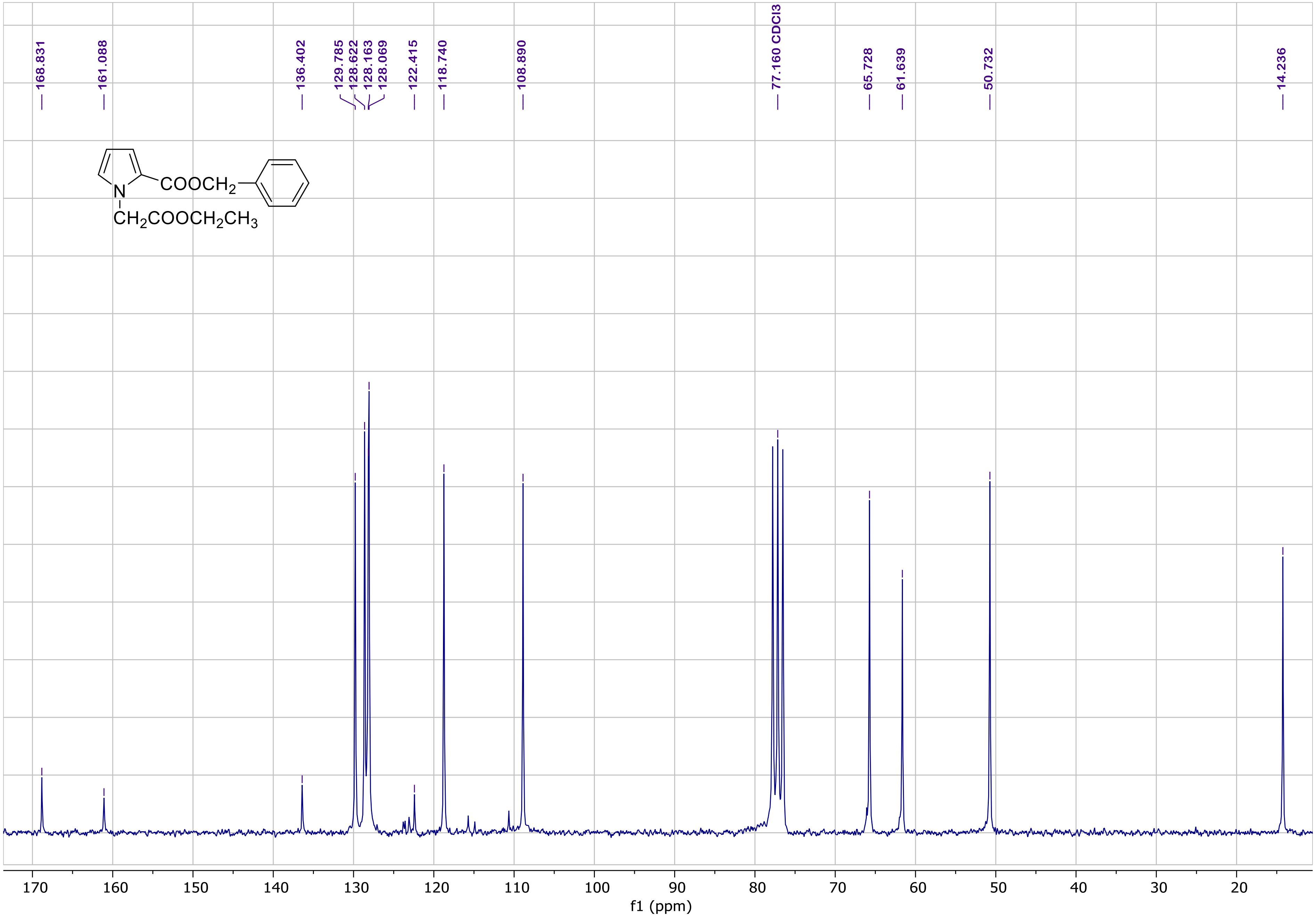

DEPT NMR of **55** (50.32 MHz, $CDCl_3$)

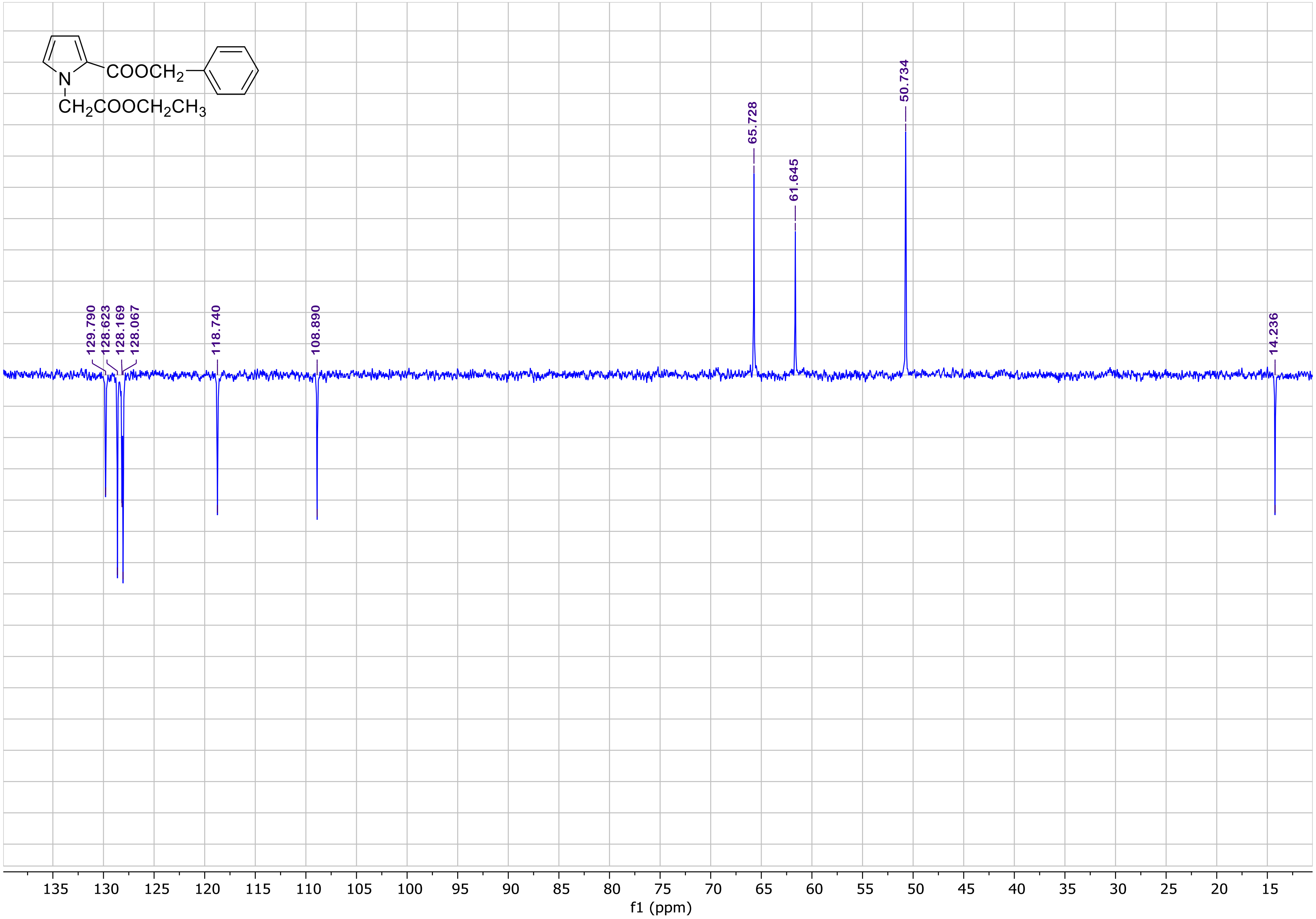

COSY NMR of **55** (600.11 MHz, CDCl3)

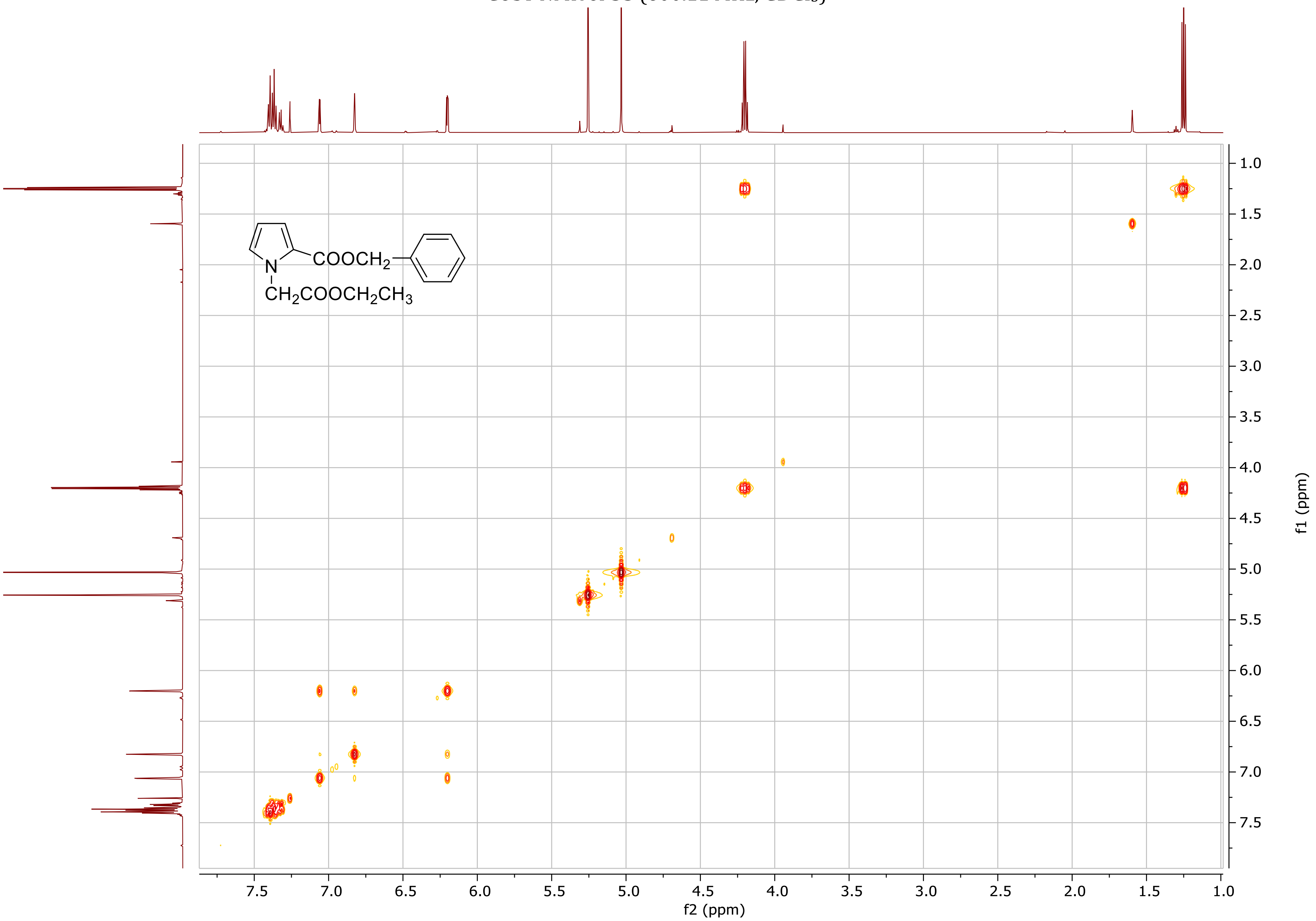

HSQC-DEPT NMR of **55** (600.11 MHz, $CDCl_3$)

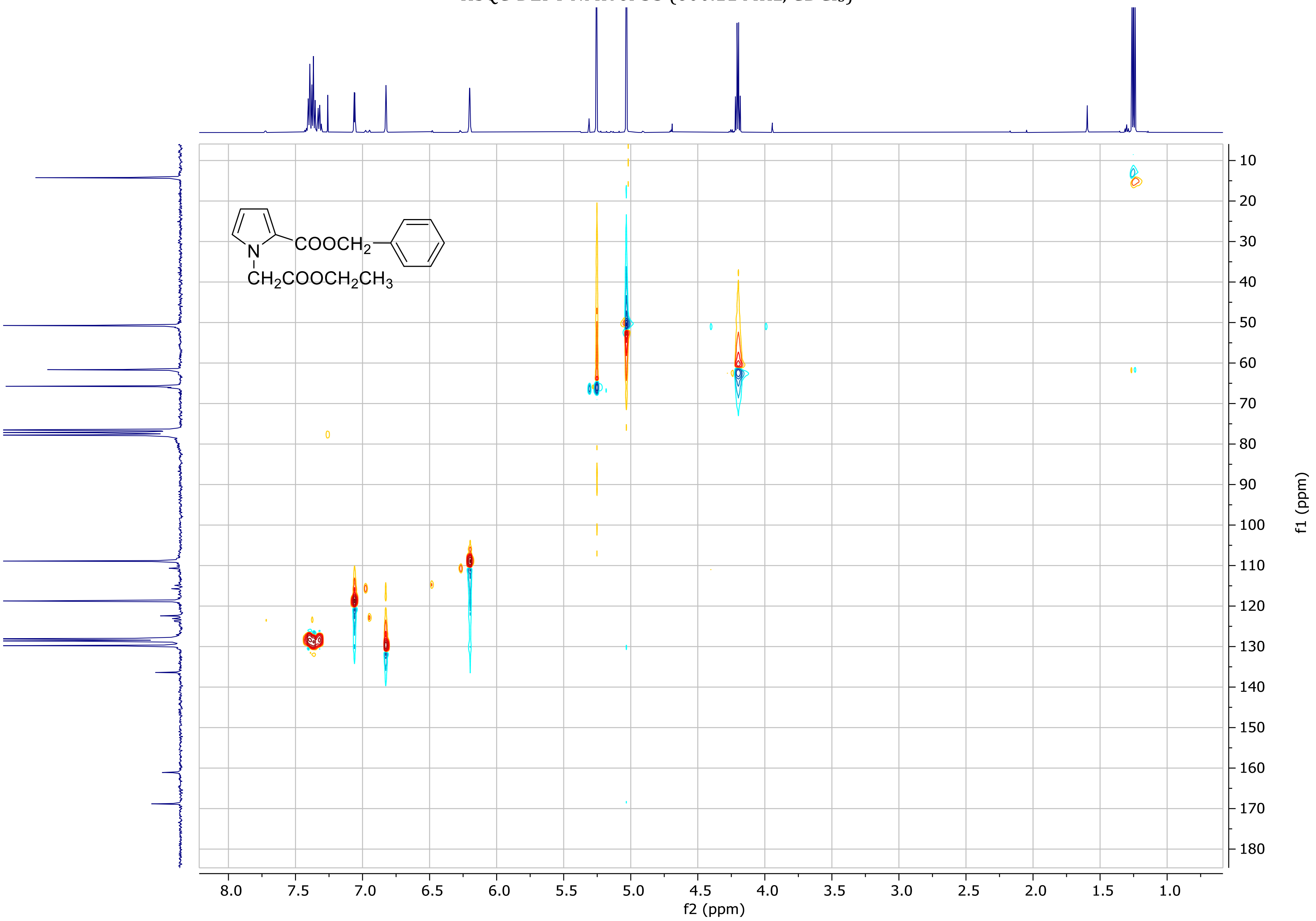

HMBC NMR of **55** (600.11 MHz, $CDCl_3$)

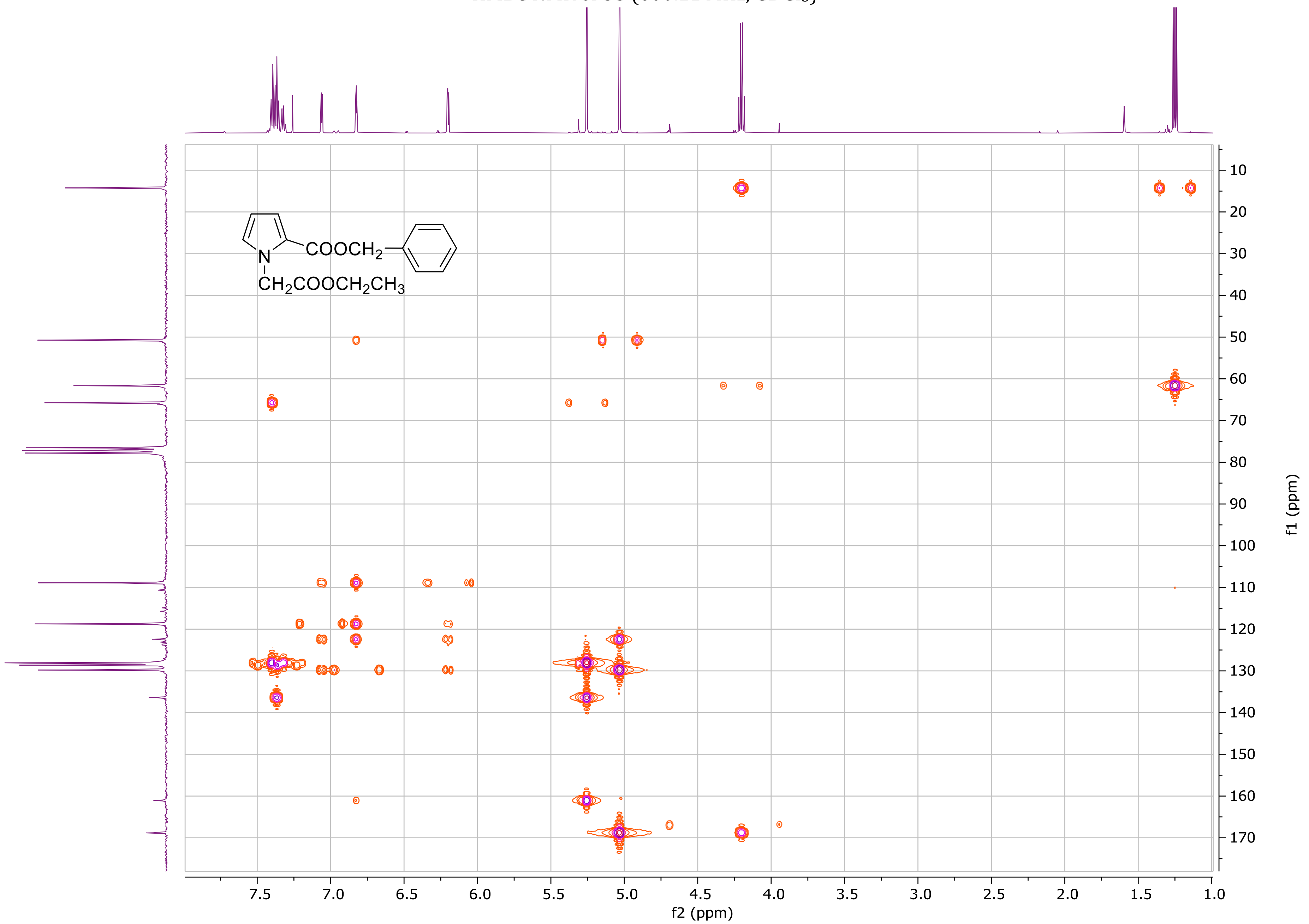

^{1}H NMR of **56** (600.11 MHz, $CDCl_3$)

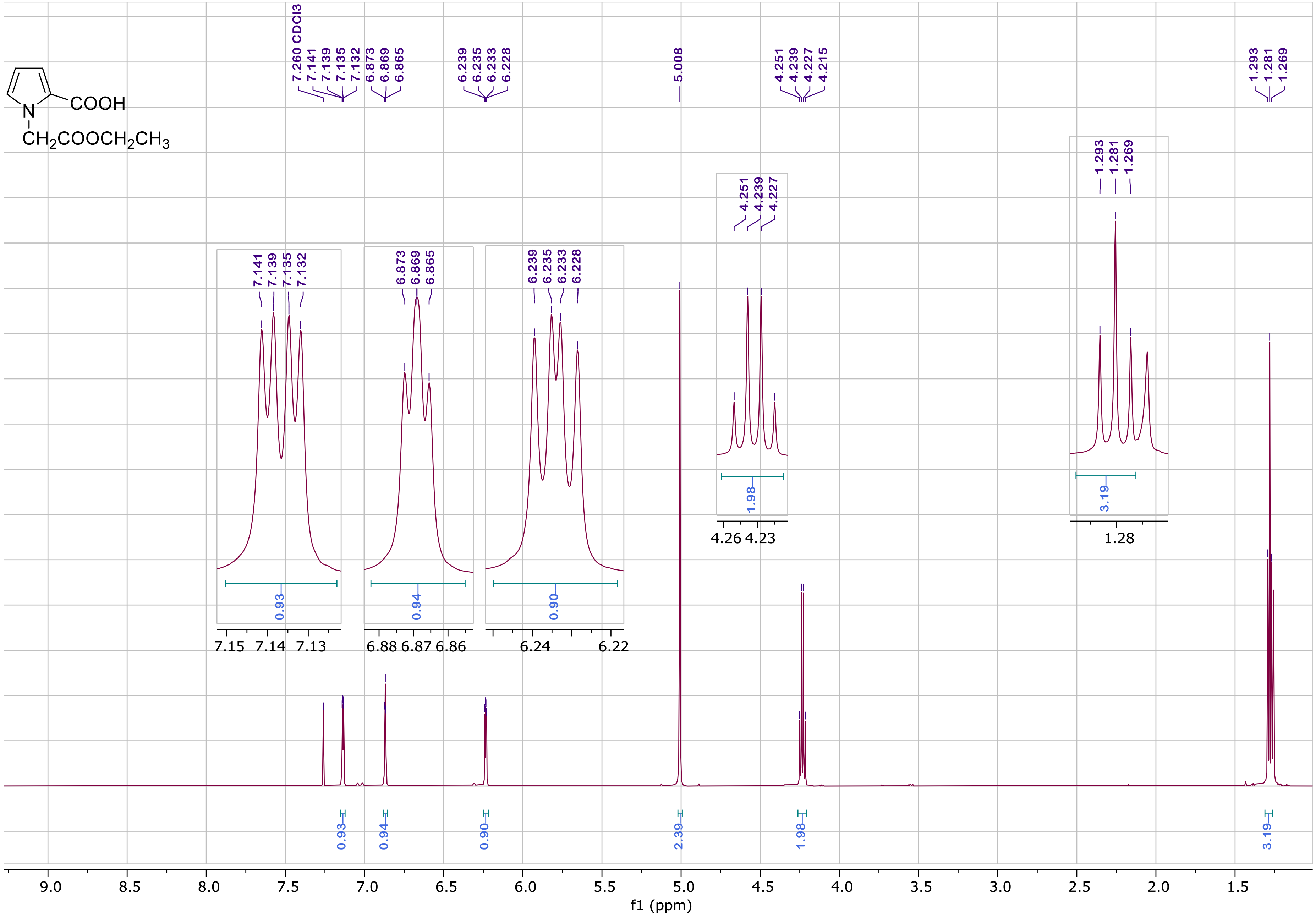

^{13}C NMR of **56** (50.32 MHz, $CDCl_3$)

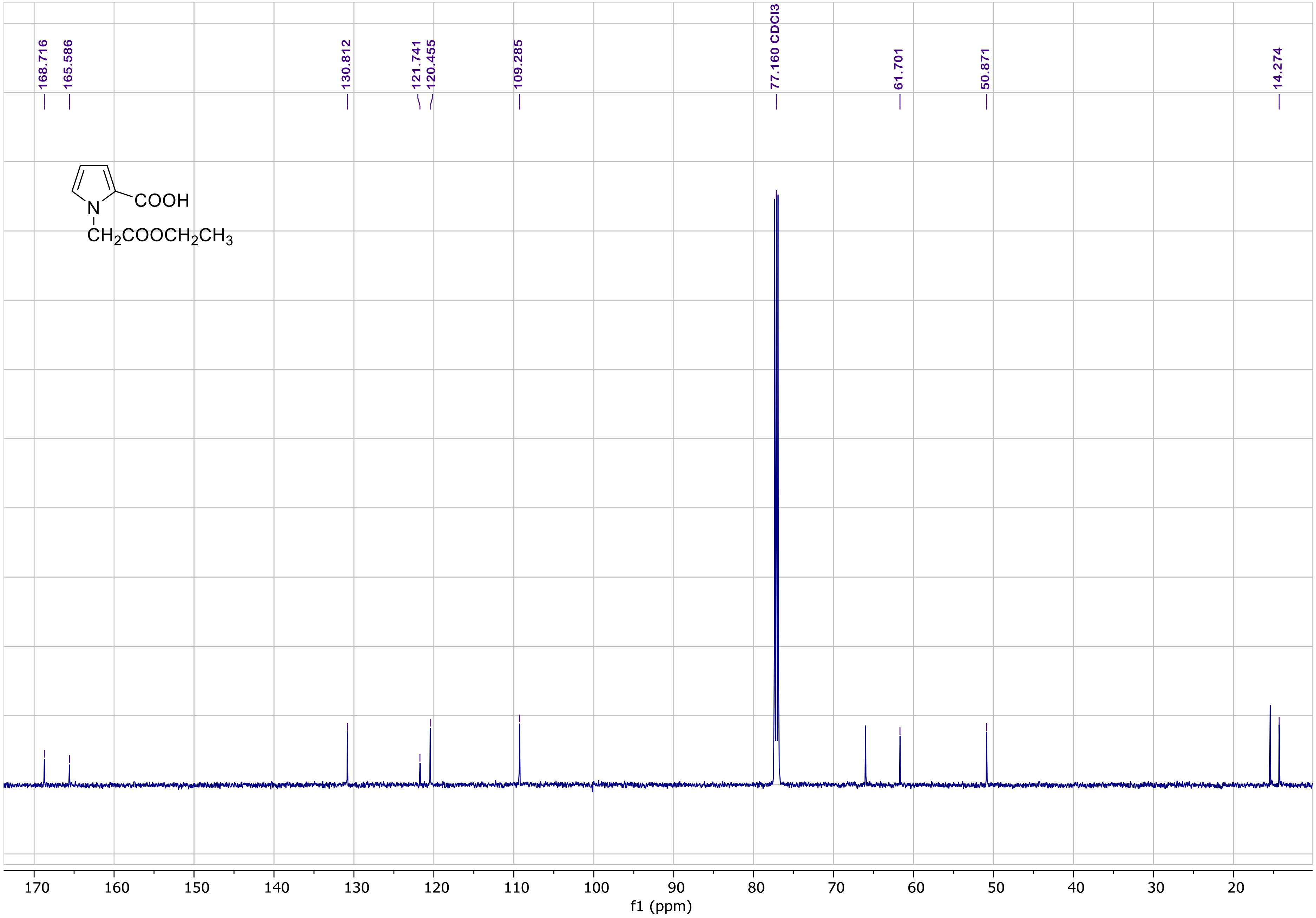

DEPT NMR of **56** (50.32 MHz, $CDCl_3$)

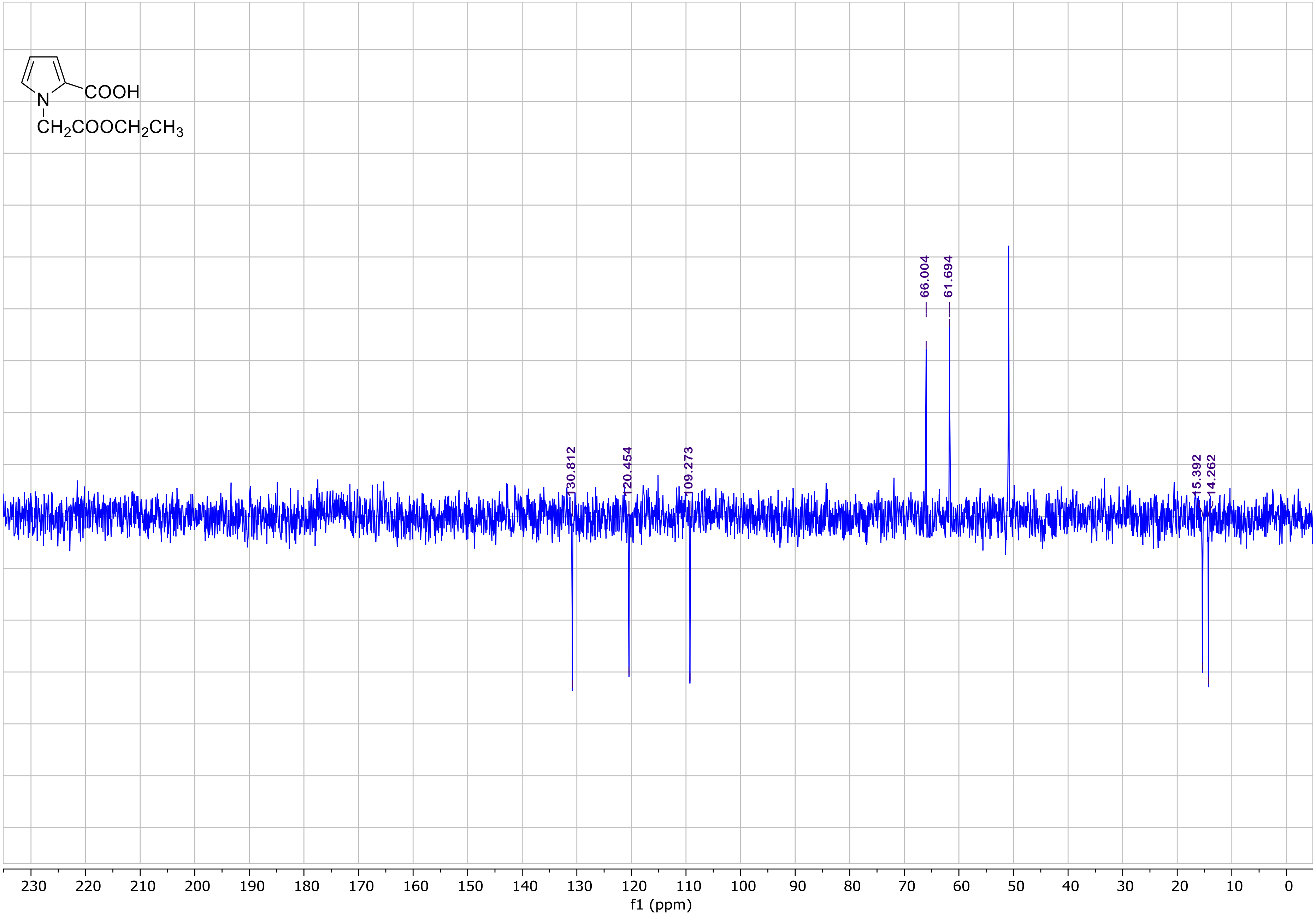

COSY NMR of **56** (600.11 MHz, $CDCl_3$)

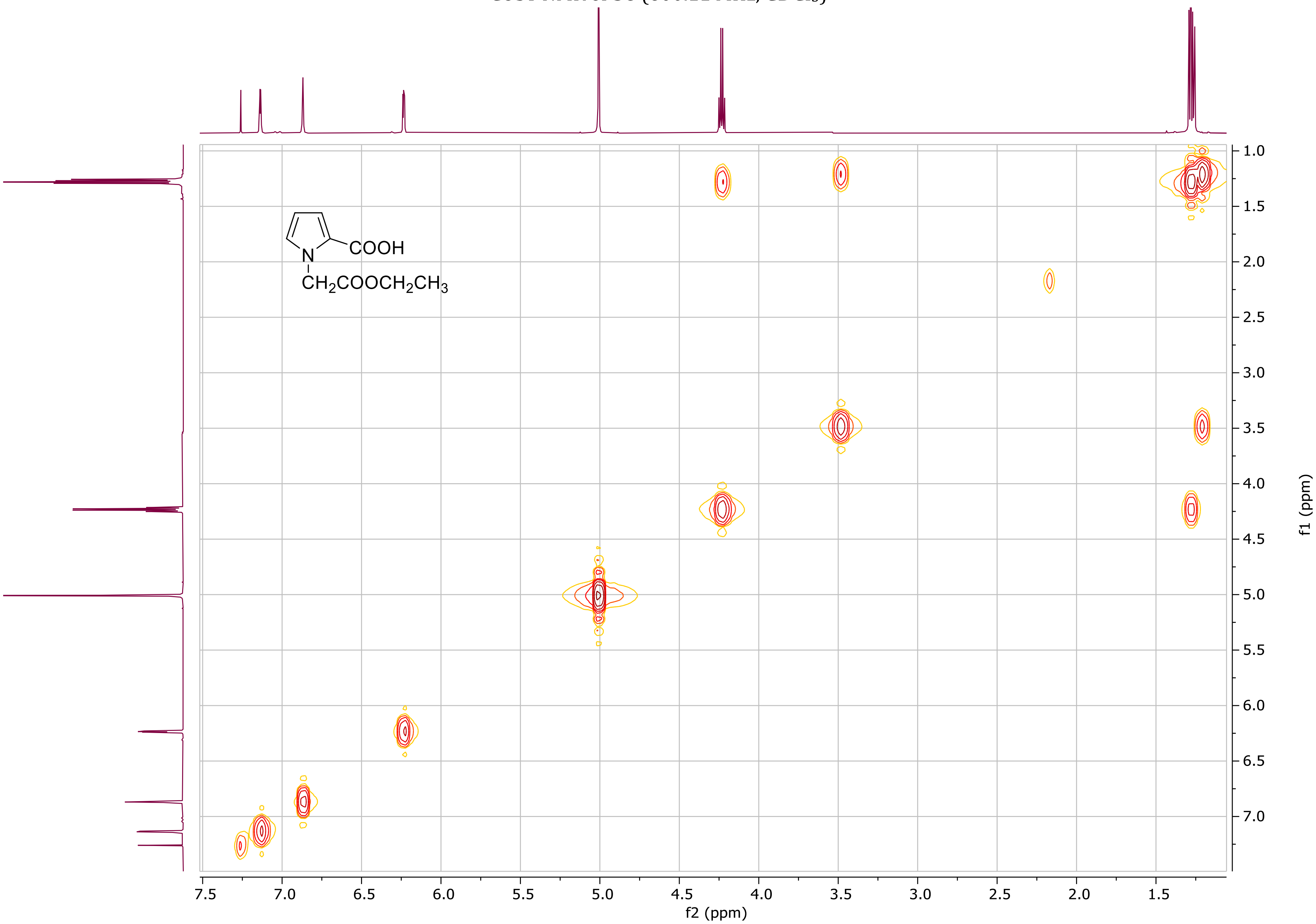

HSQC-DEPT NMR of **56** (600.11 MHz, $CDCl_3$)

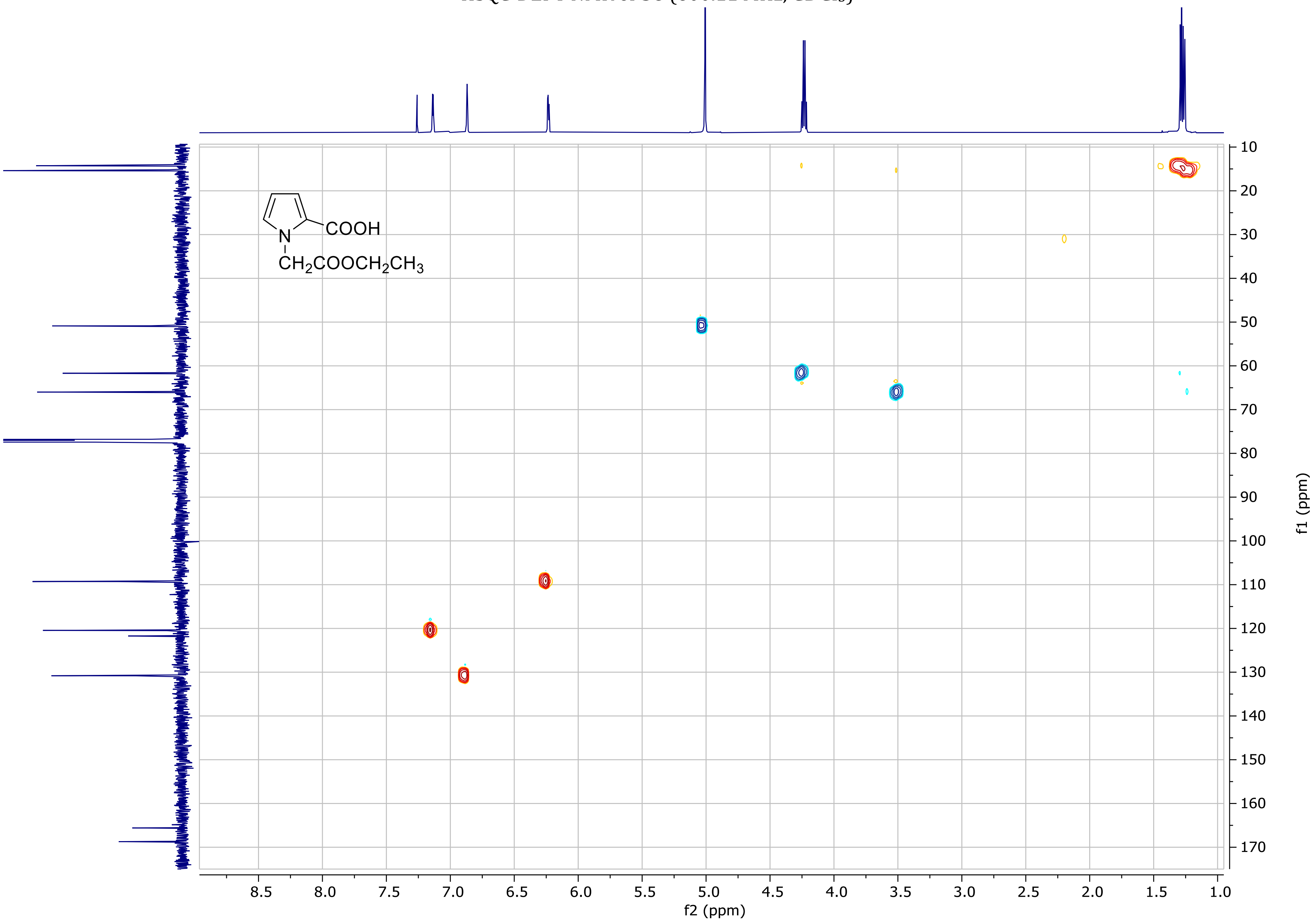

HMBC NMR of **56** (600.11 MHz, $CDCl_3$)

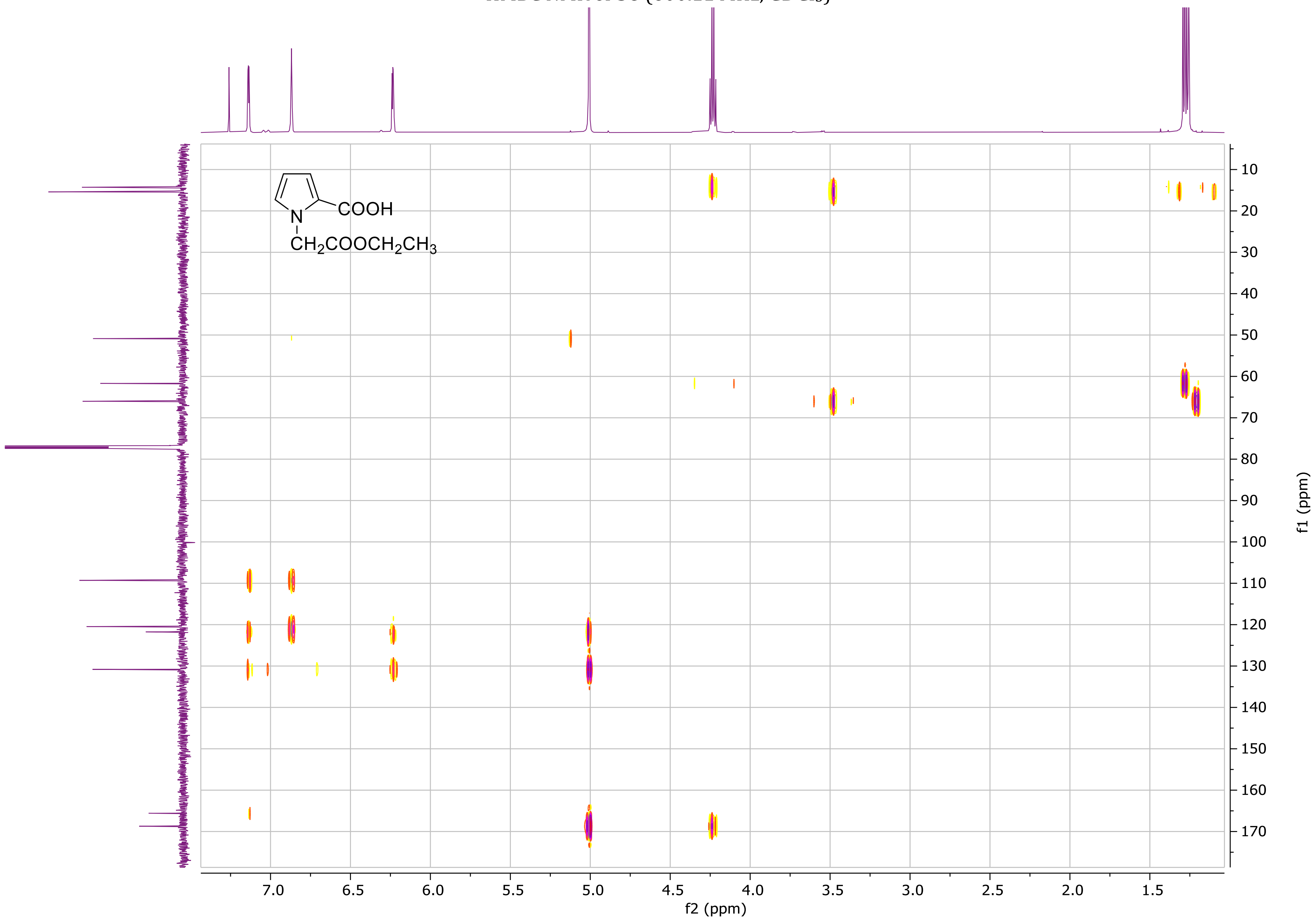

^{1}H NMR of **57** (600.11 MHz, $CDCl_3$)

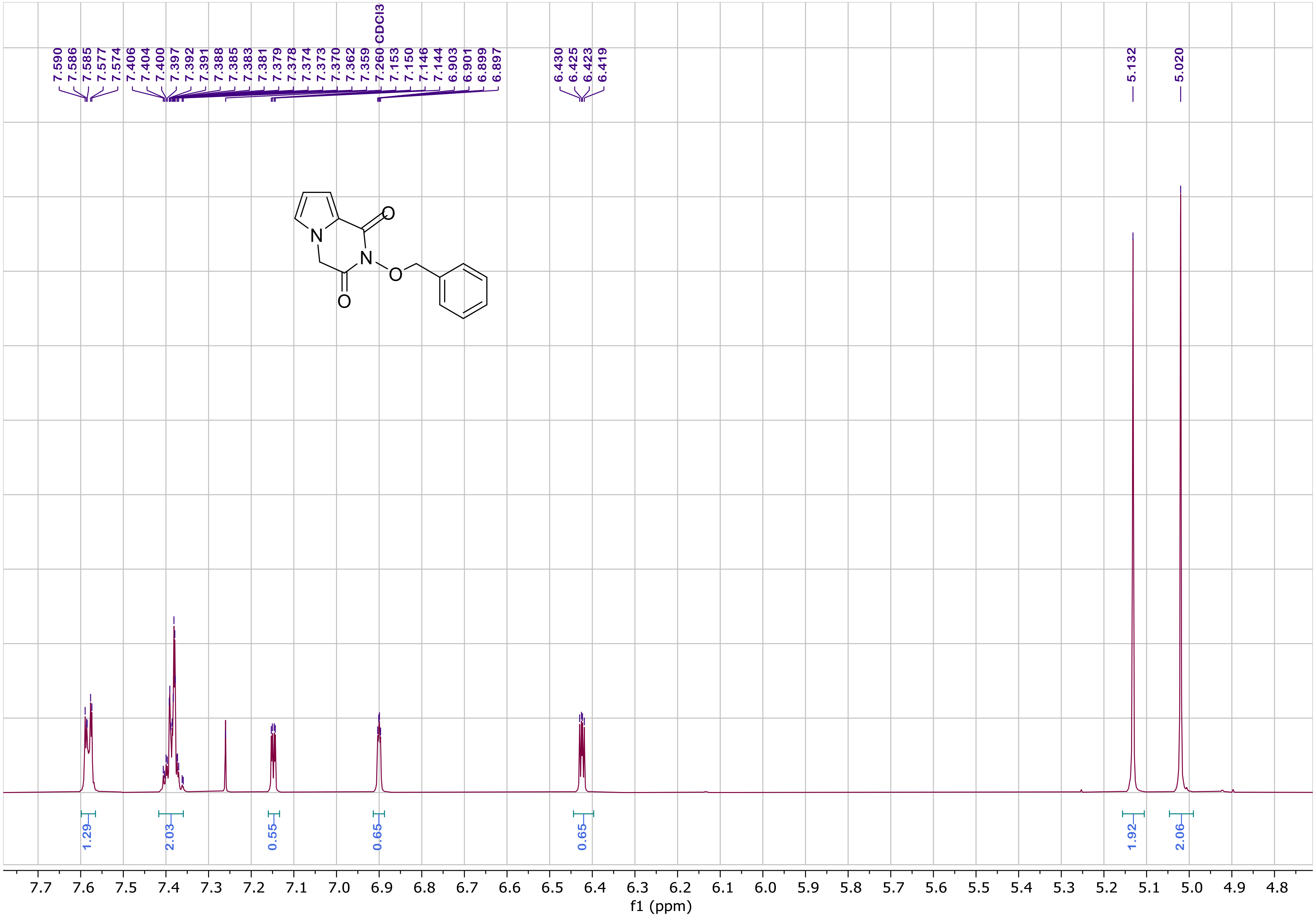

^{13}C NMR of **57** (150.9 MHz, $CDCl_3$)

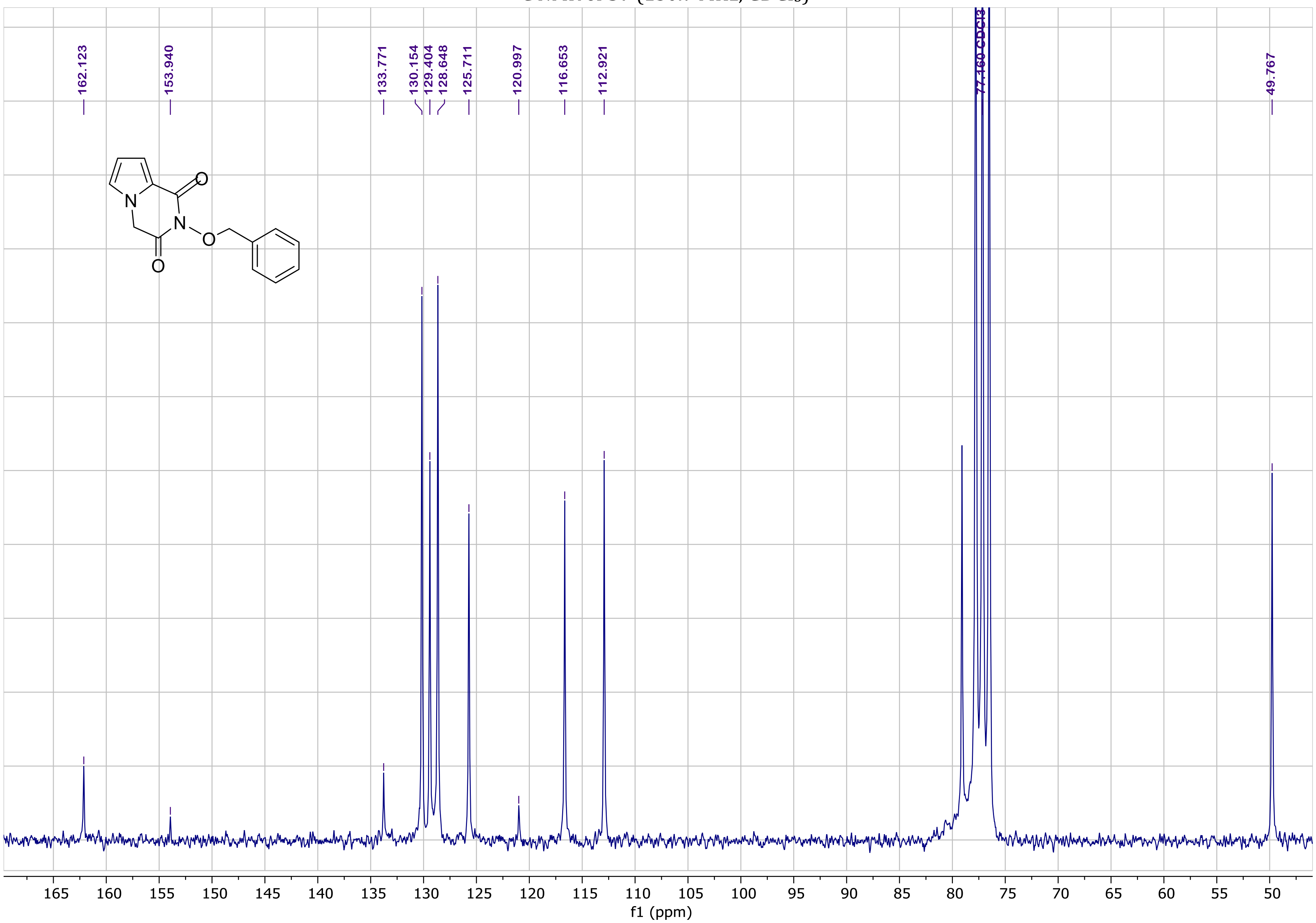

COSY NMR of **57** (400.13 MHz, $CDCl_3$)

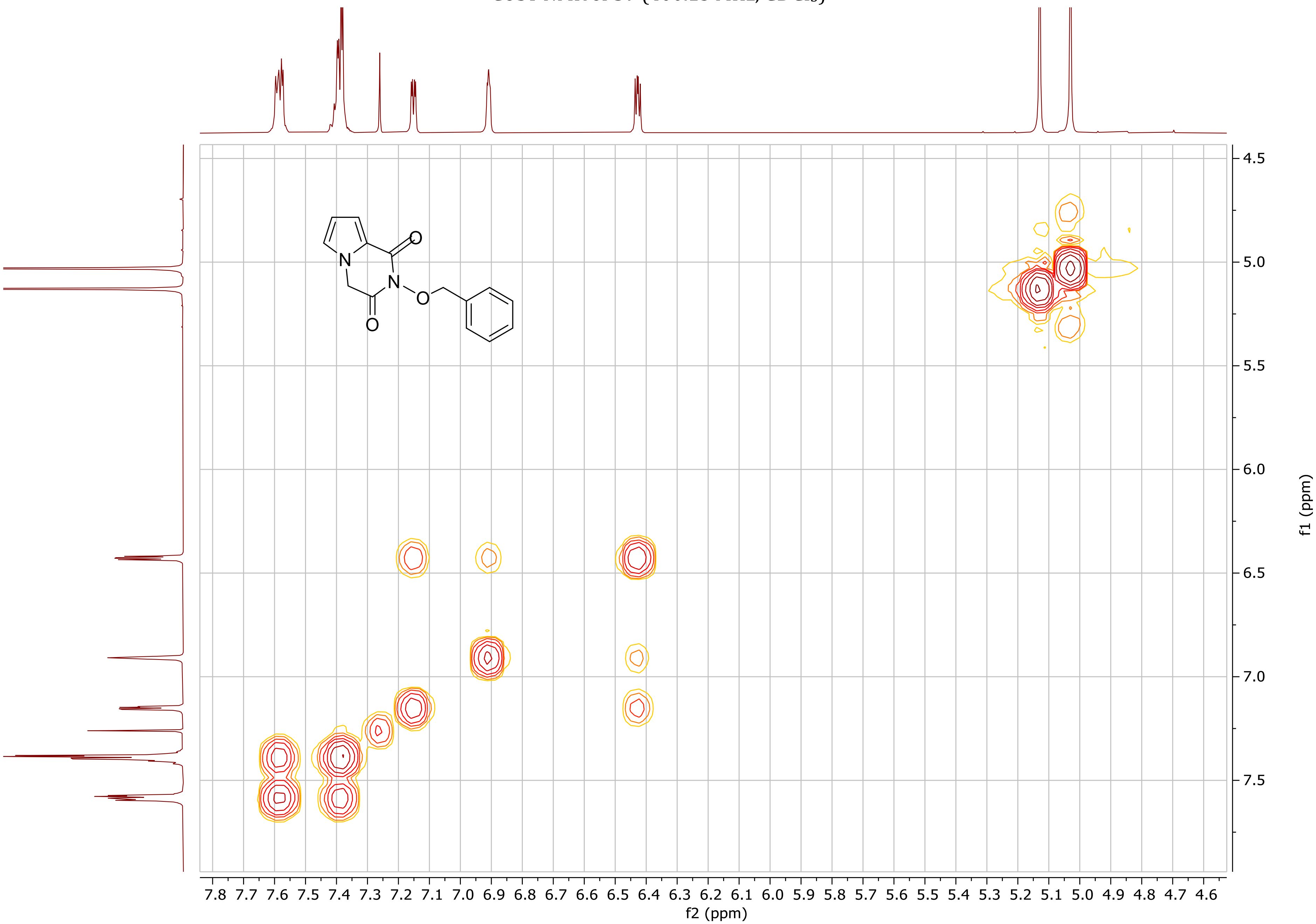

HSQC-DEPT NMR of **57** (600.11 MHz, $CDCl_3$)

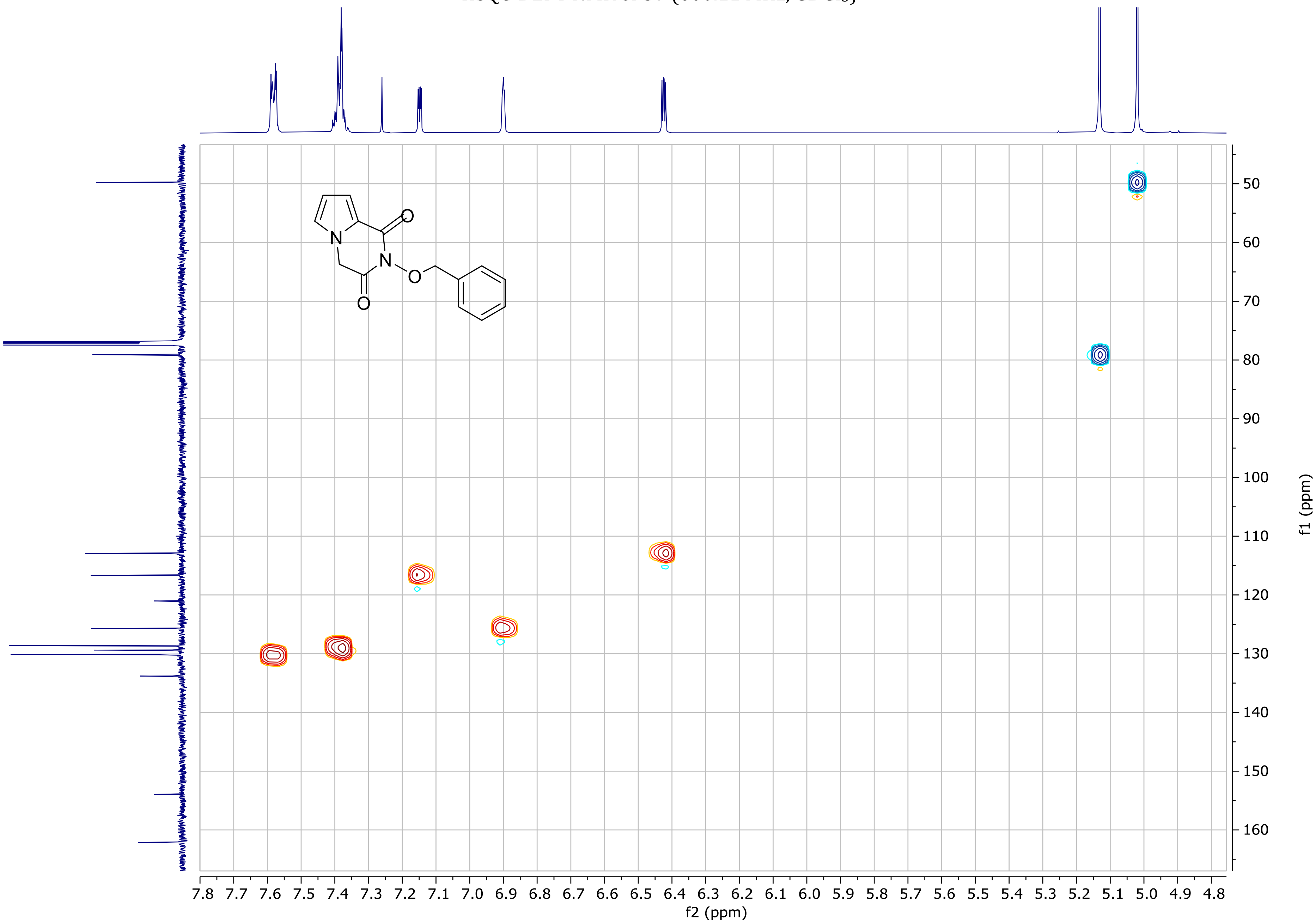

HMBC NMR of **57** (600.11 MHz, $CDCl_3$)

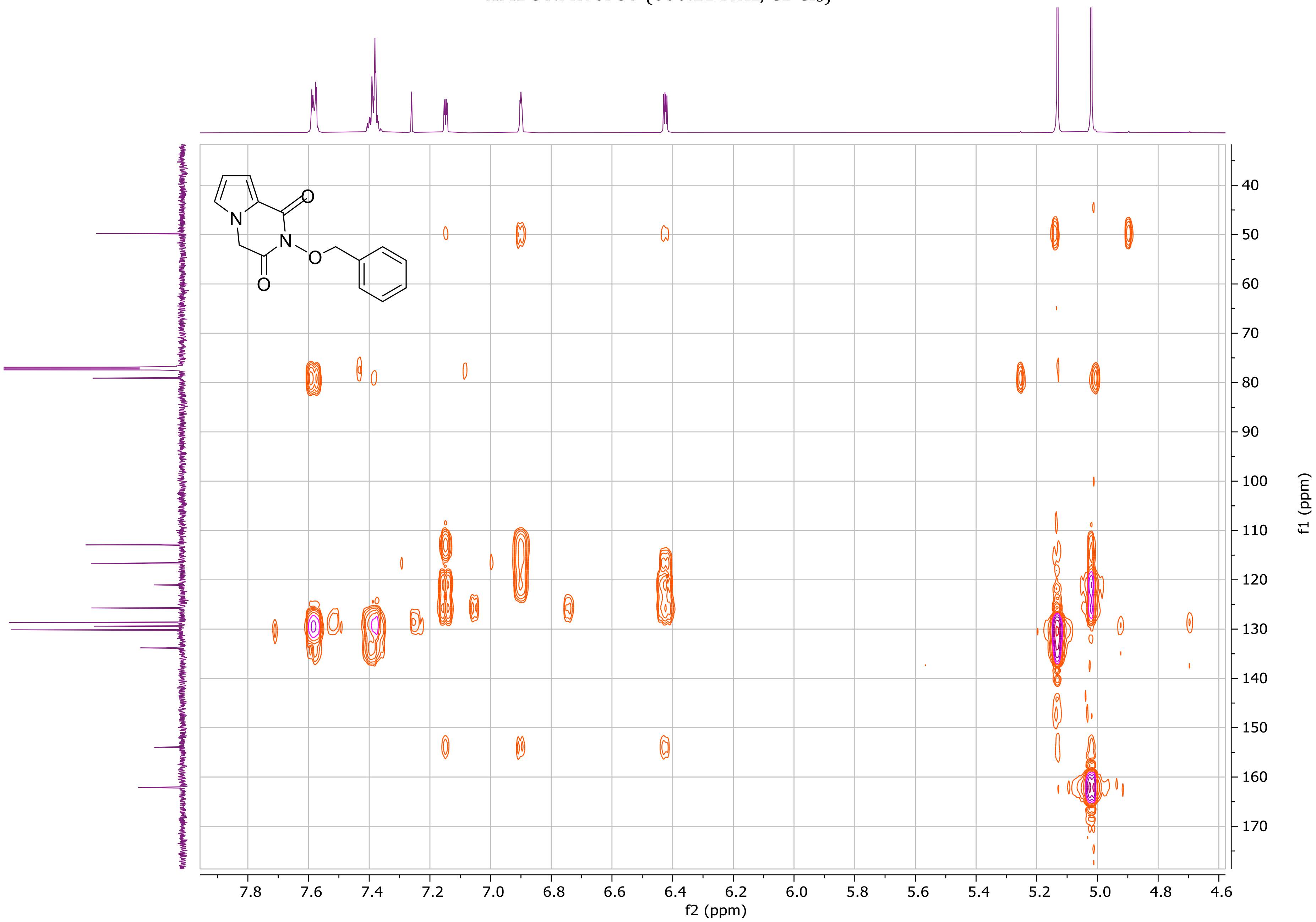

^{1}H NMR of **58** (600.11 MHz, DMSO-d_6)

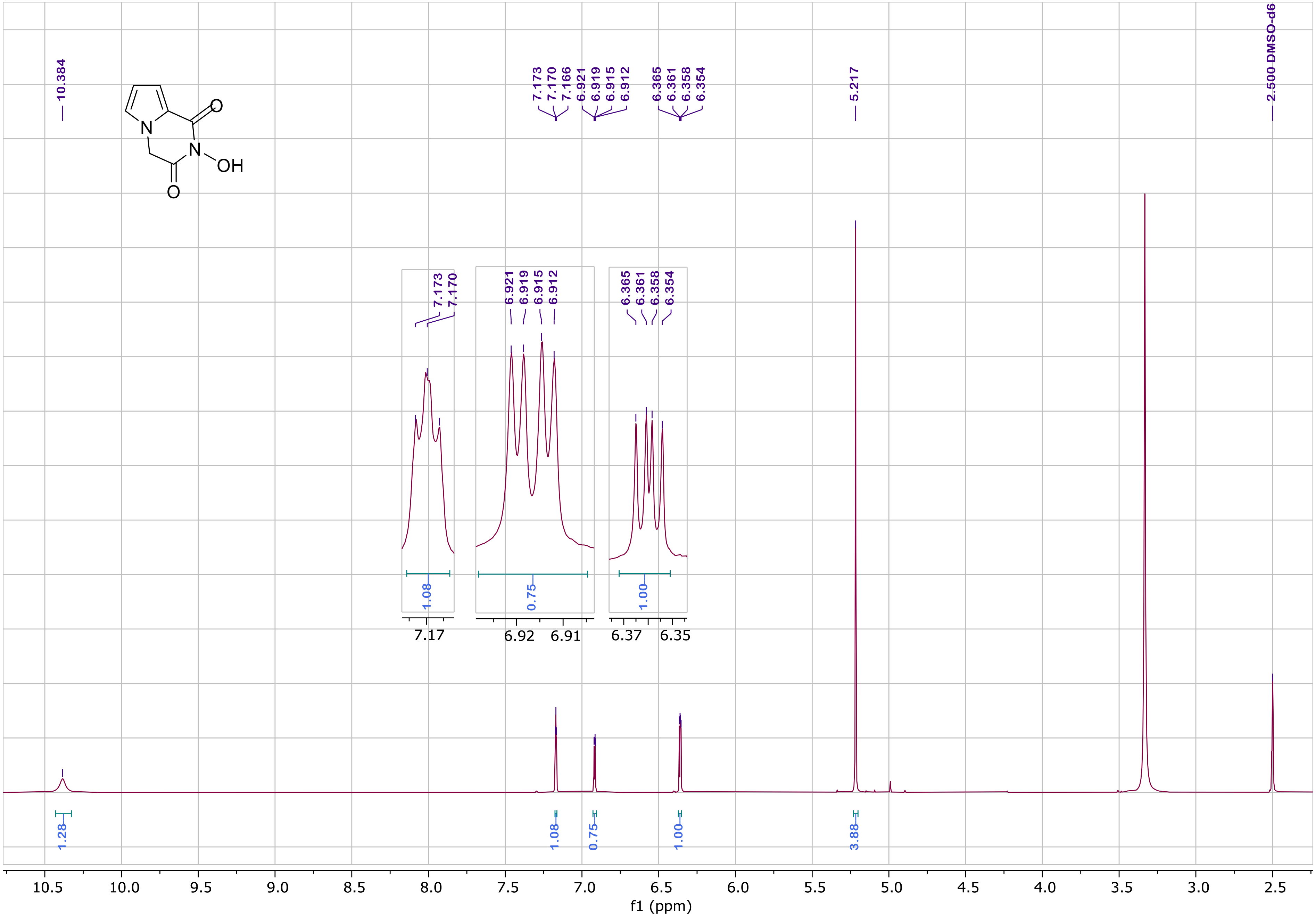

^{13}C NMR of **58** (150.9 MHz, DMSO-d_6)

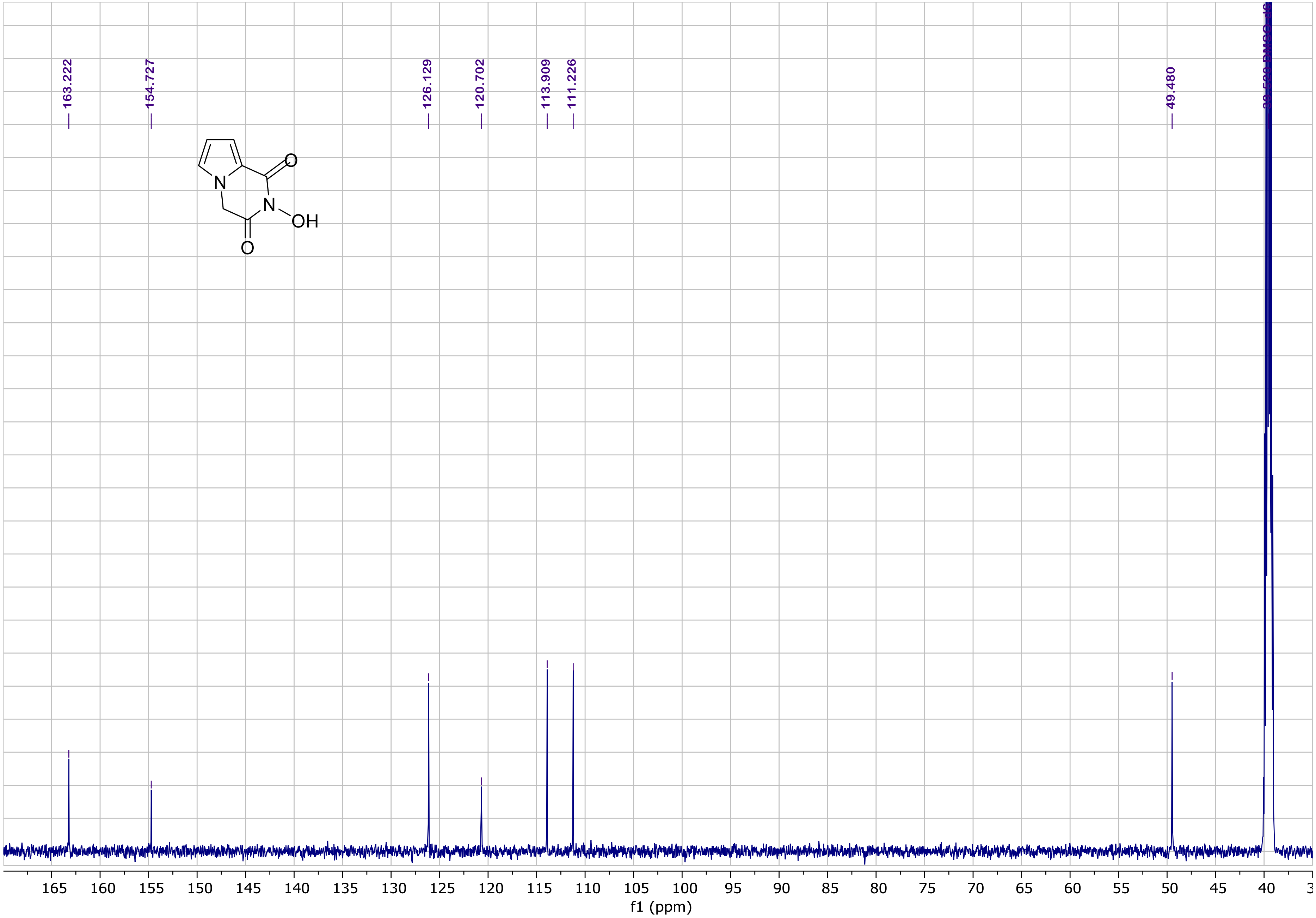

DEPT NMR of **58** (50.32 MHz, DMSO-d_6)

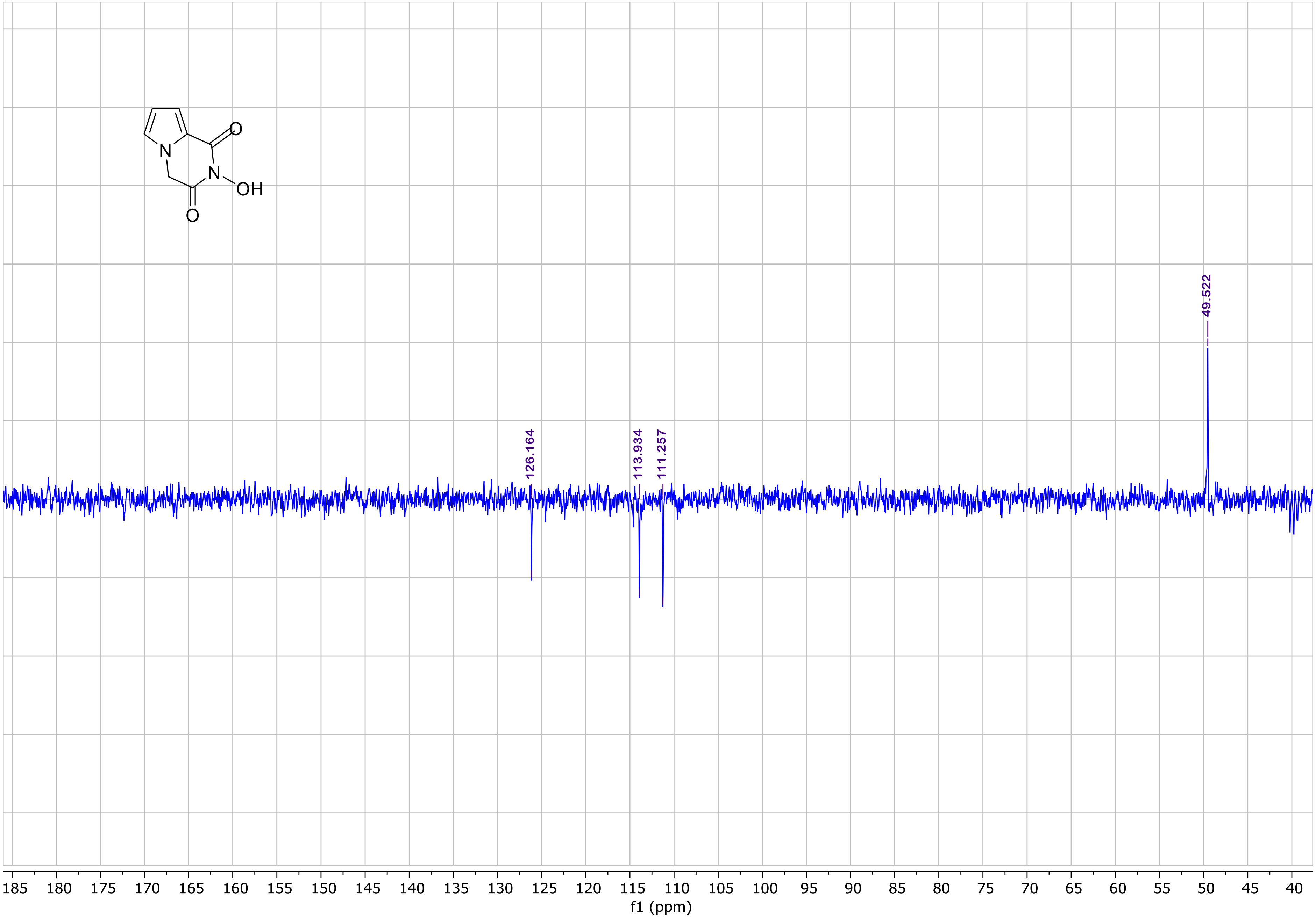

COSY NMR of **58** (600.11 MHz, DMSO-d_6)

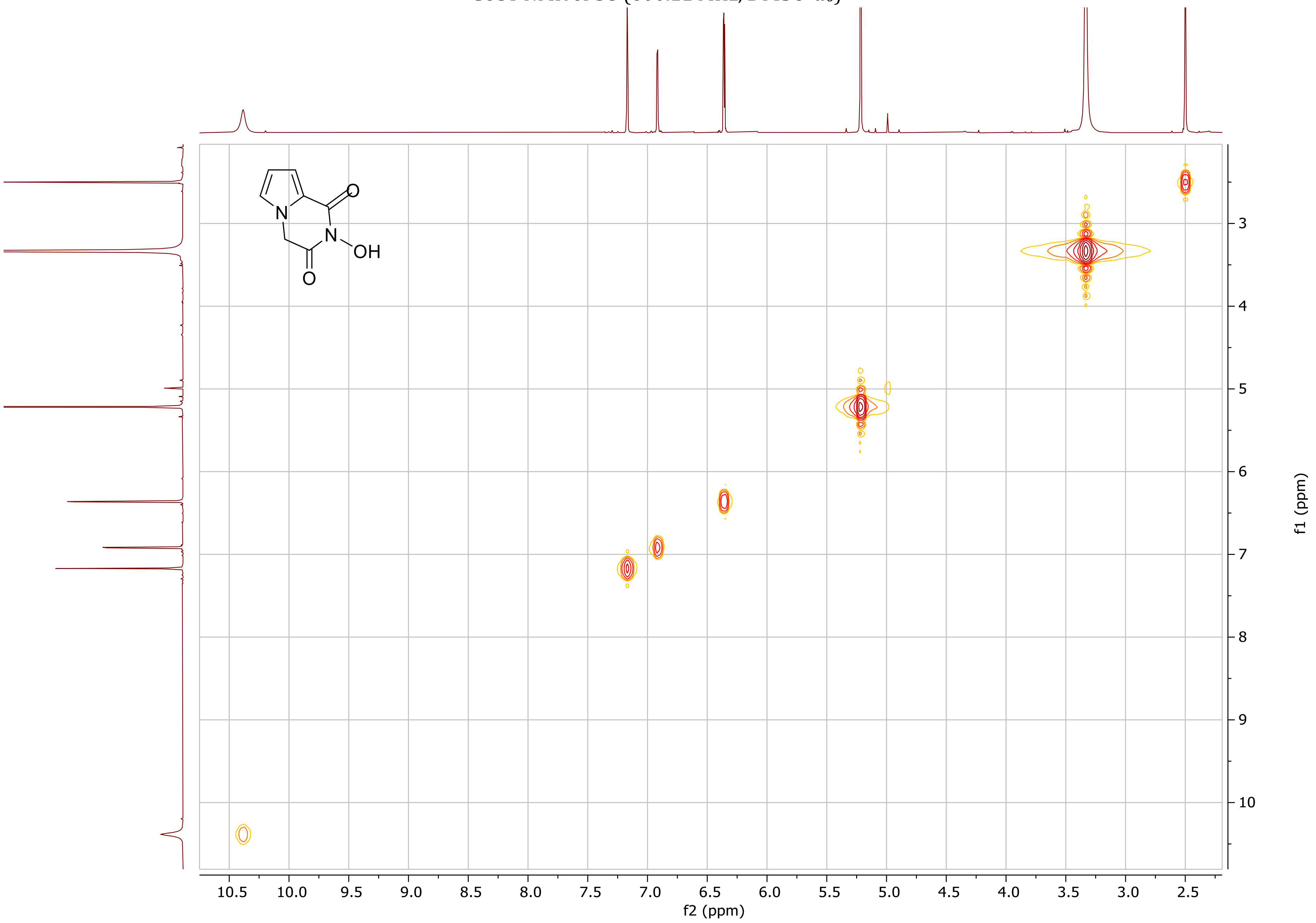

HSQC-DEPT NMR of **58** (600.11 MHz, DMSO-d_6)

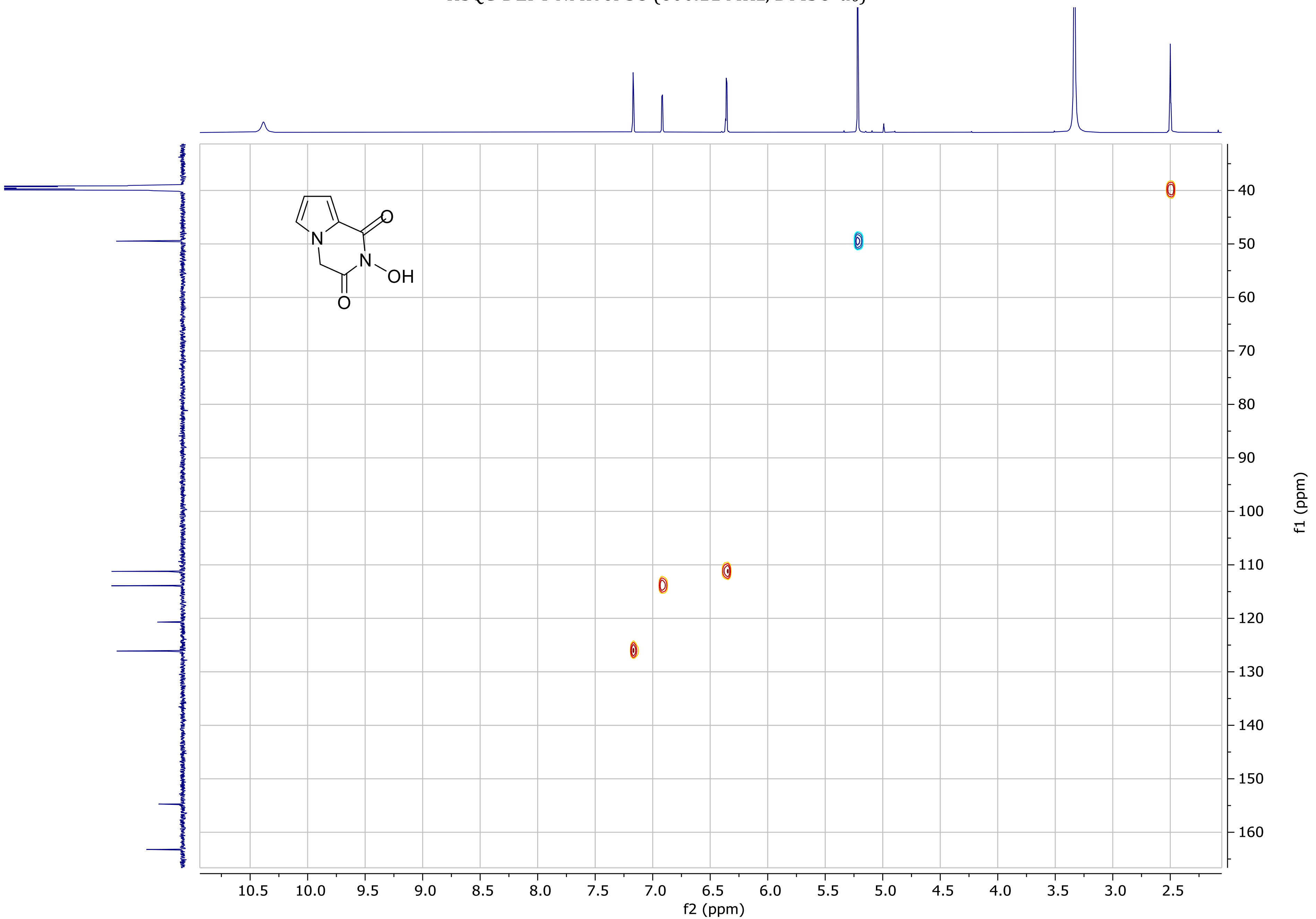

HMBC NMR of **58** (600.11 MHz, DMSO-d_6)

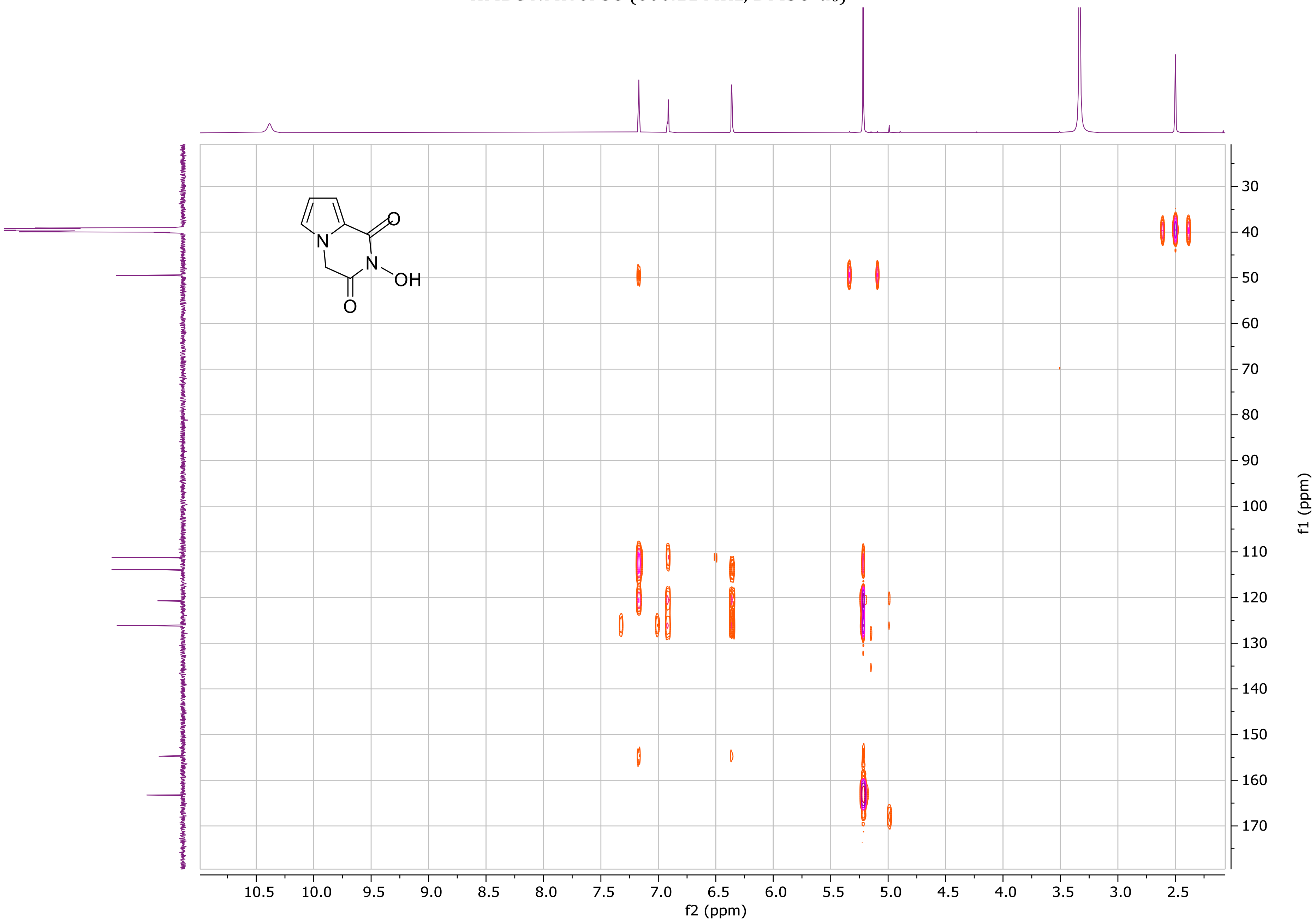

^{1}H NMR of **59** (600.11 MHz, $CDCl_3$)

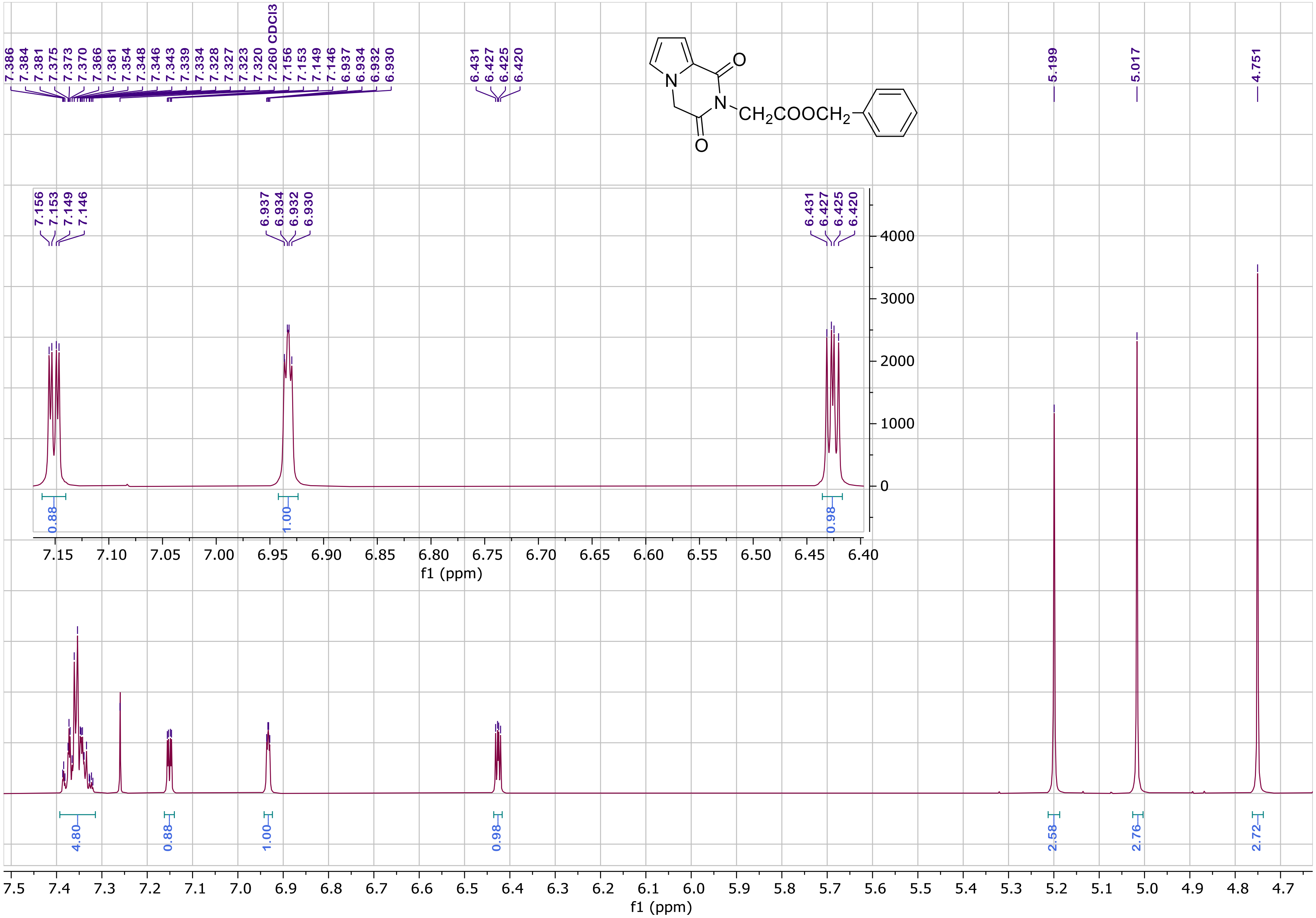

^{13}C NMR of **59** (50.32 MHz, CDCl3)

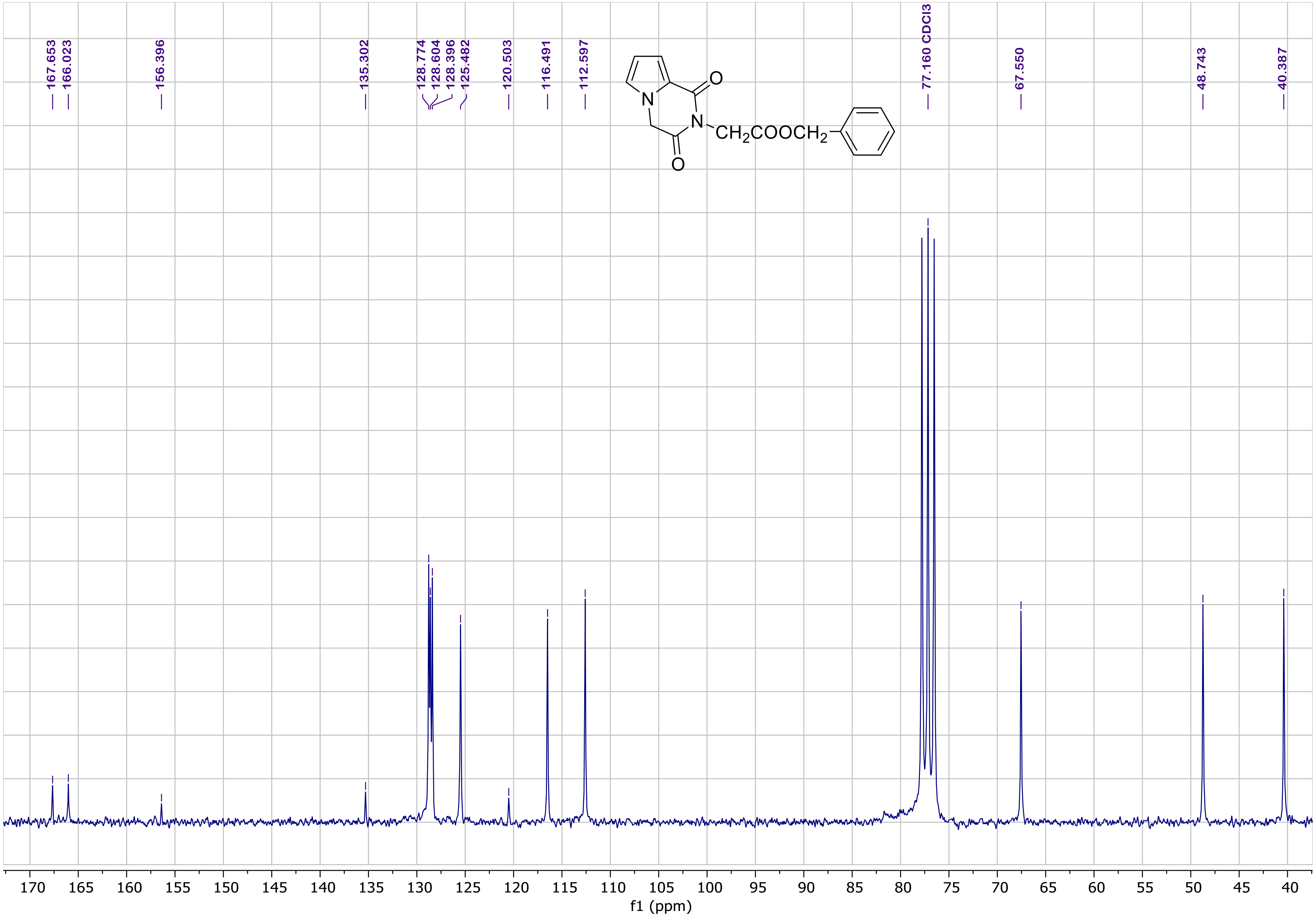

DEPT NMR of **59** (50.32 MHz, $CDCl_3$)

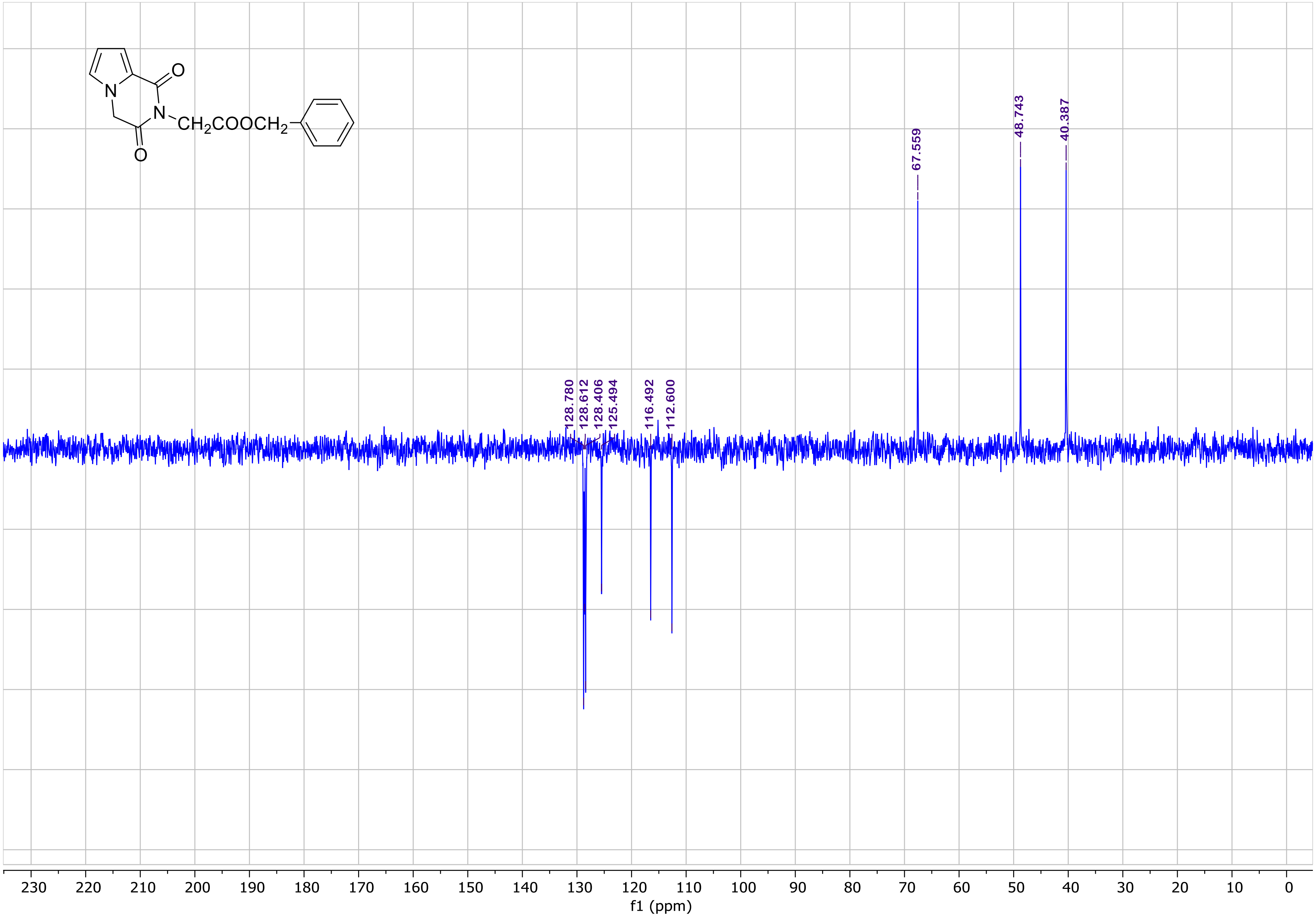

COSY NMR of **59** (600.11 MHz, $CDCl_3$)

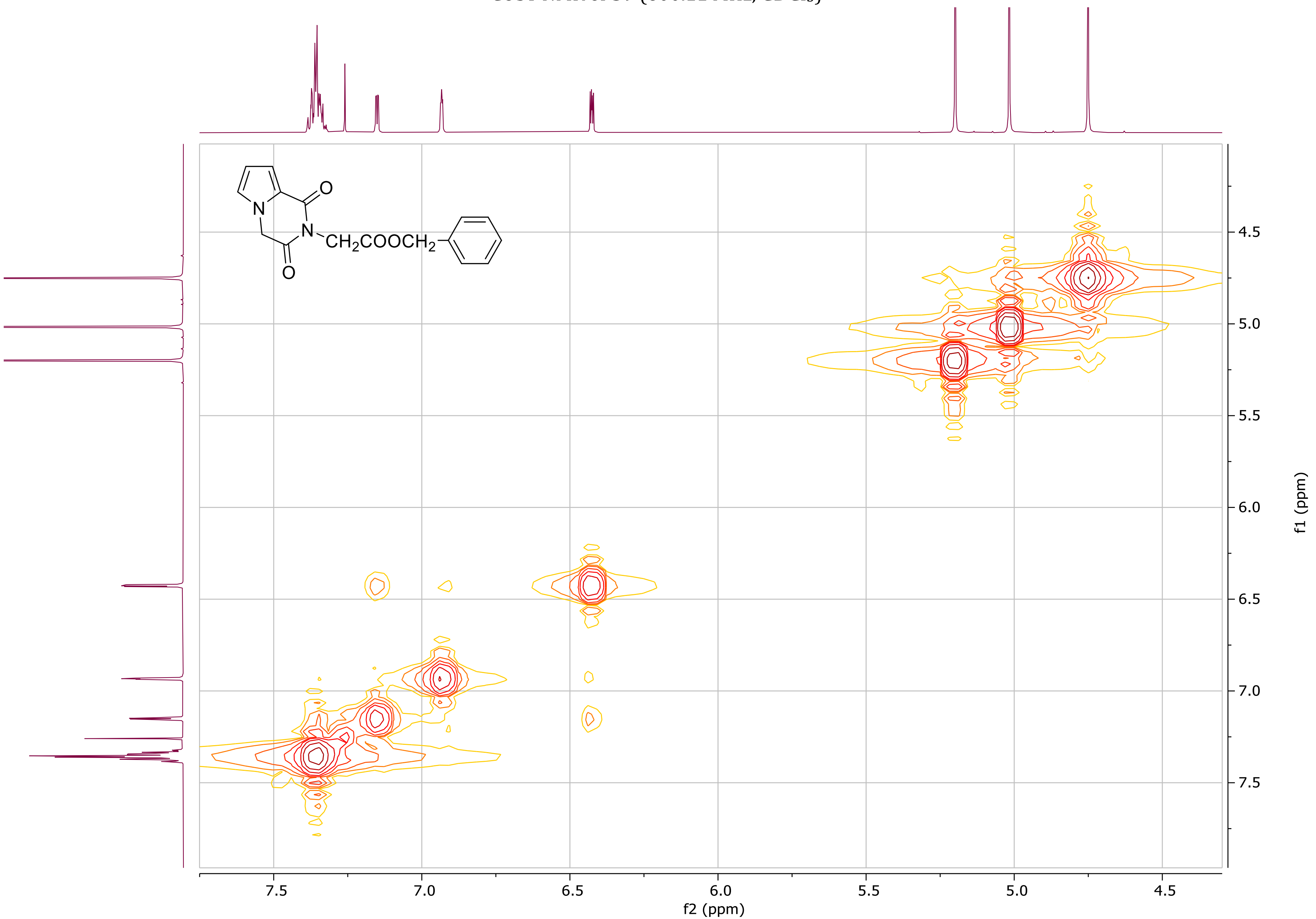

HSQC-DEPT NMR of **57** (600.11 MHz, $CDCl_3$)

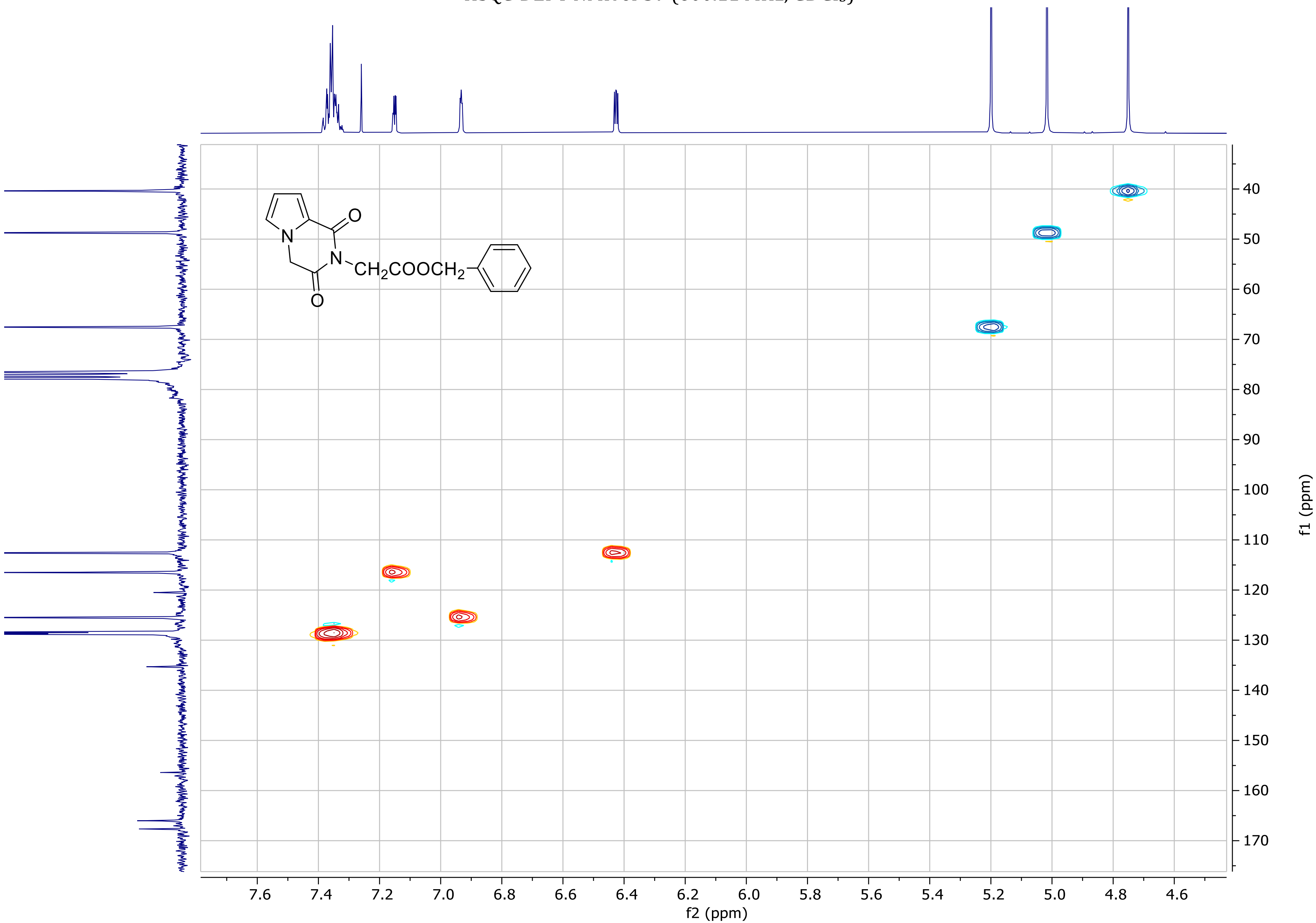

HMBC NMR of **59** (600.11 MHz, $CDCl_3$)

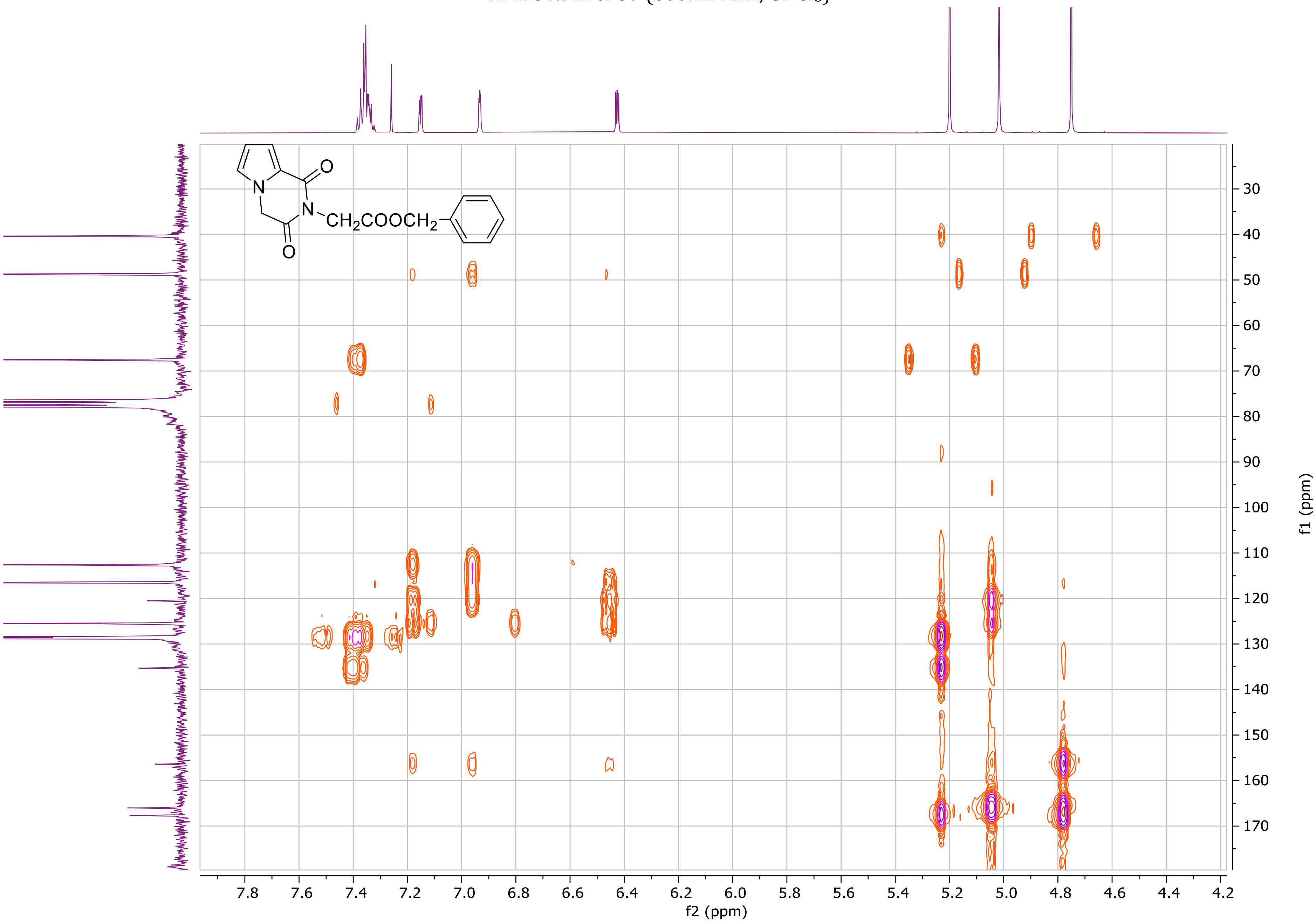

^{1}H NMR of **59a** (600.11 MHz, $CDCl_3$)

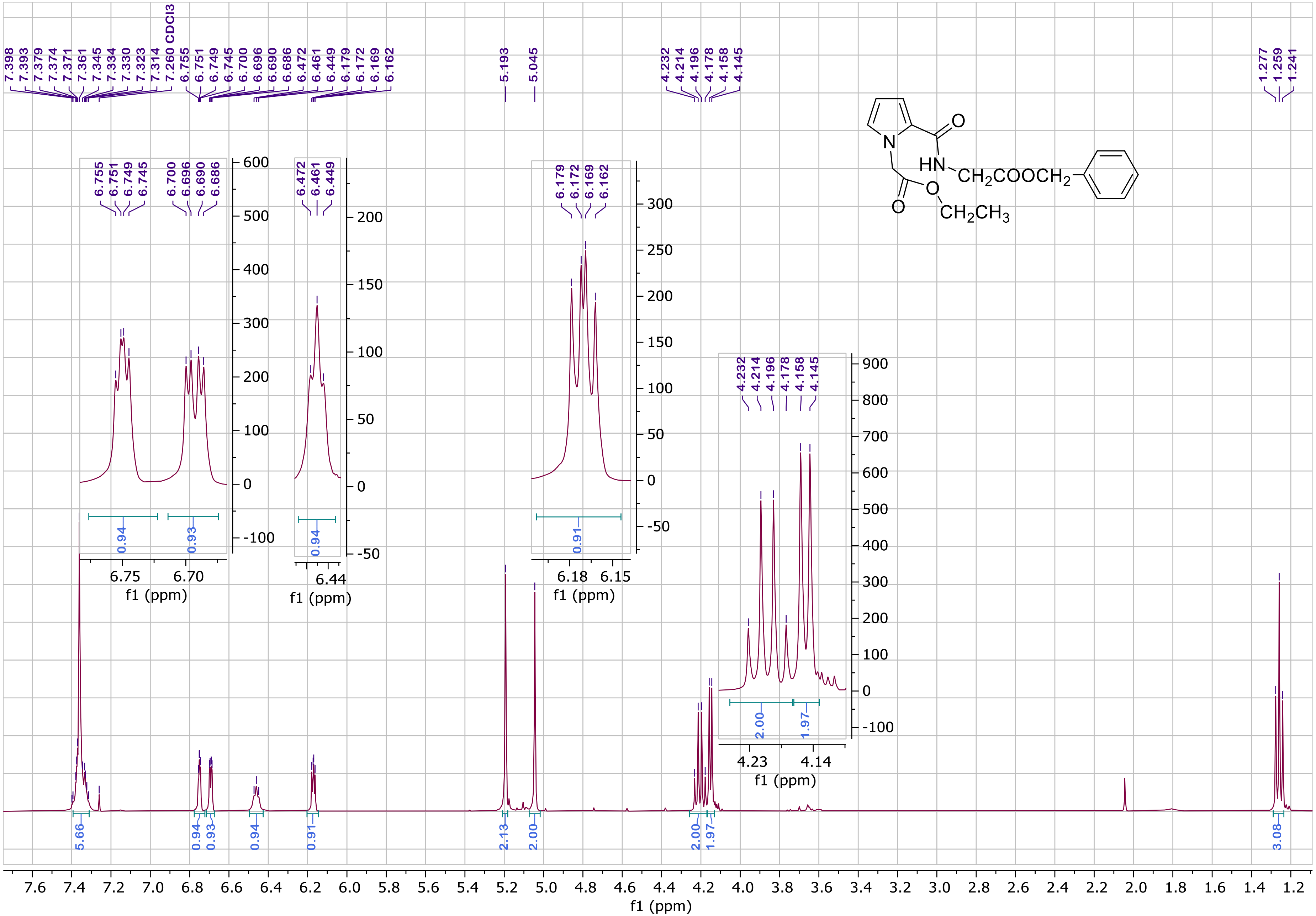

^{13}C NMR of **59a** (50.32 MHz, $CDCl_3$)

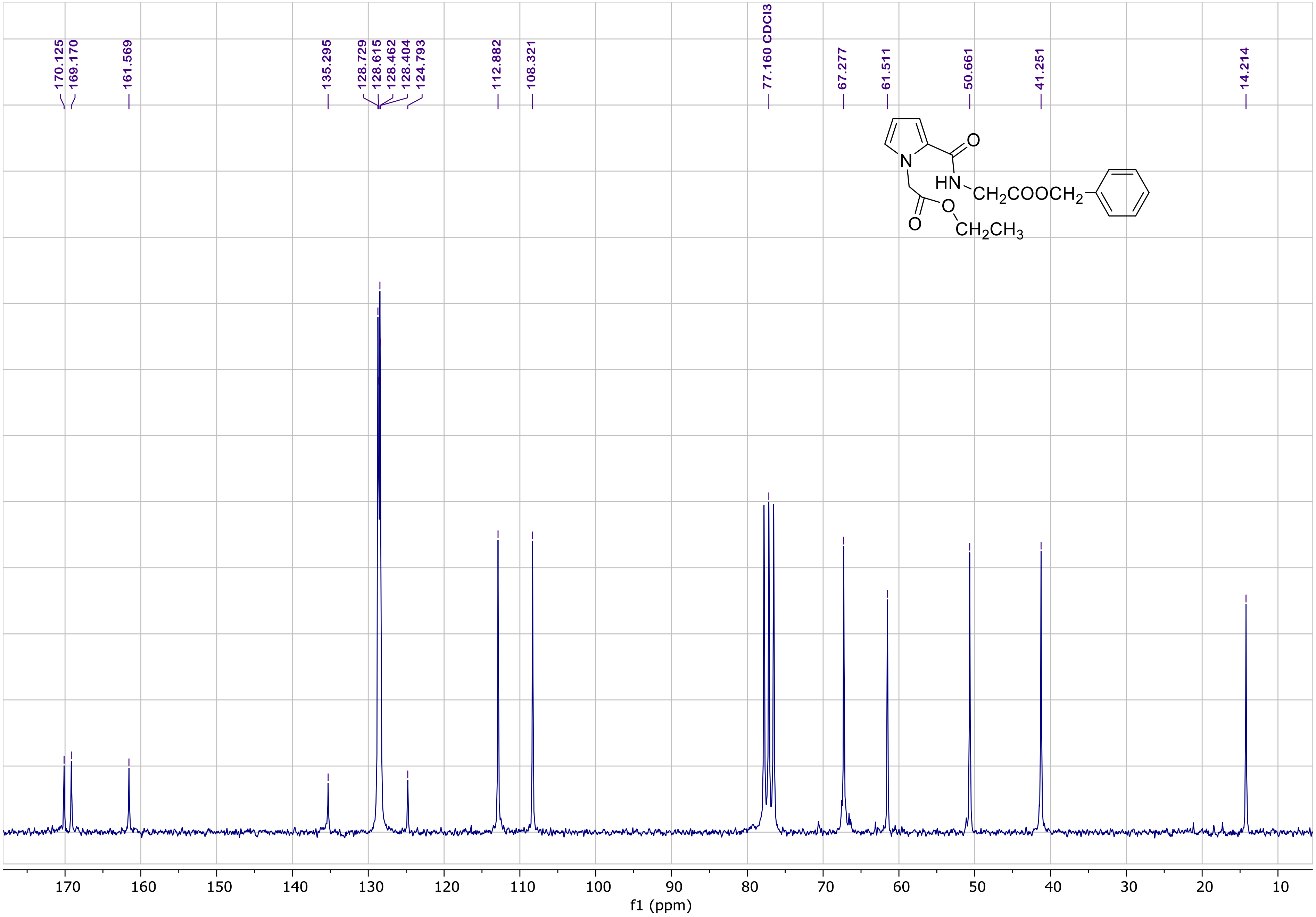

DEPT NMR of **59a** (50.32 MHz, $CDCl_3$)

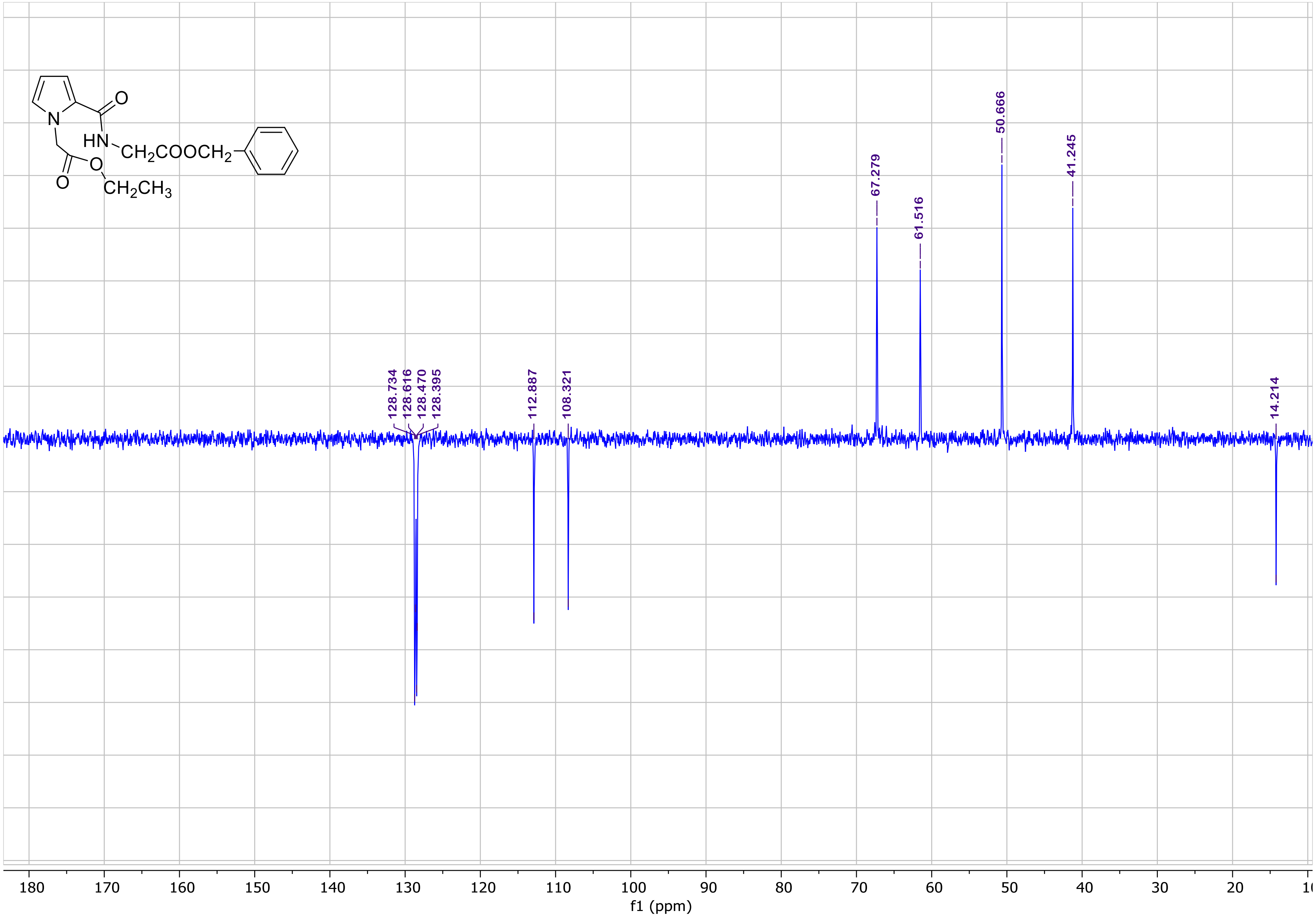

COSY NMR of **59a** (600.11 MHz, $CDCl_3$)

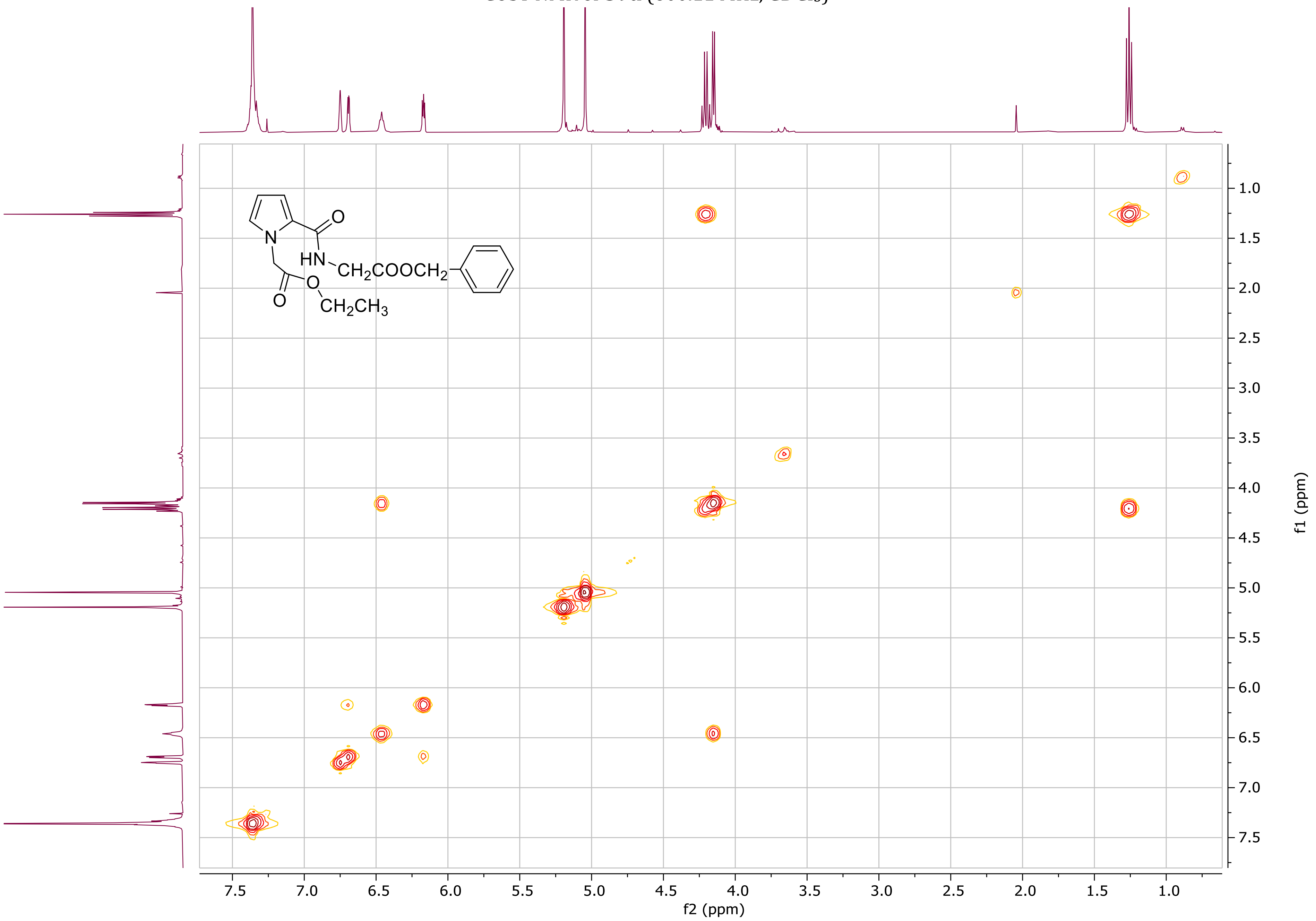

HSQC-DEPT NMR of **59a** (600.11 MHz, $CDCl_3$)

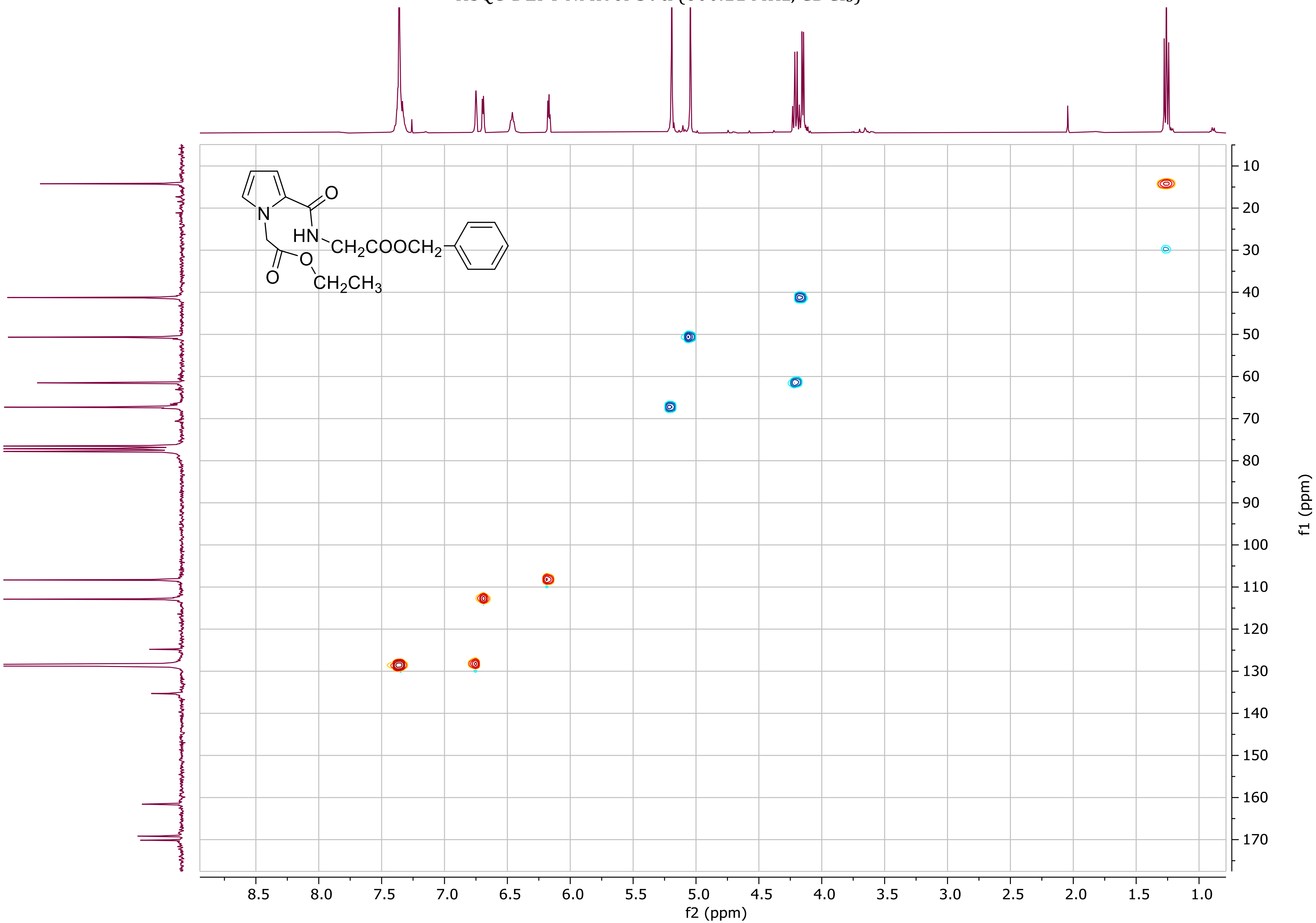

HMBC NMR of **59a** (600.11 MHz, $CDCl_3$)

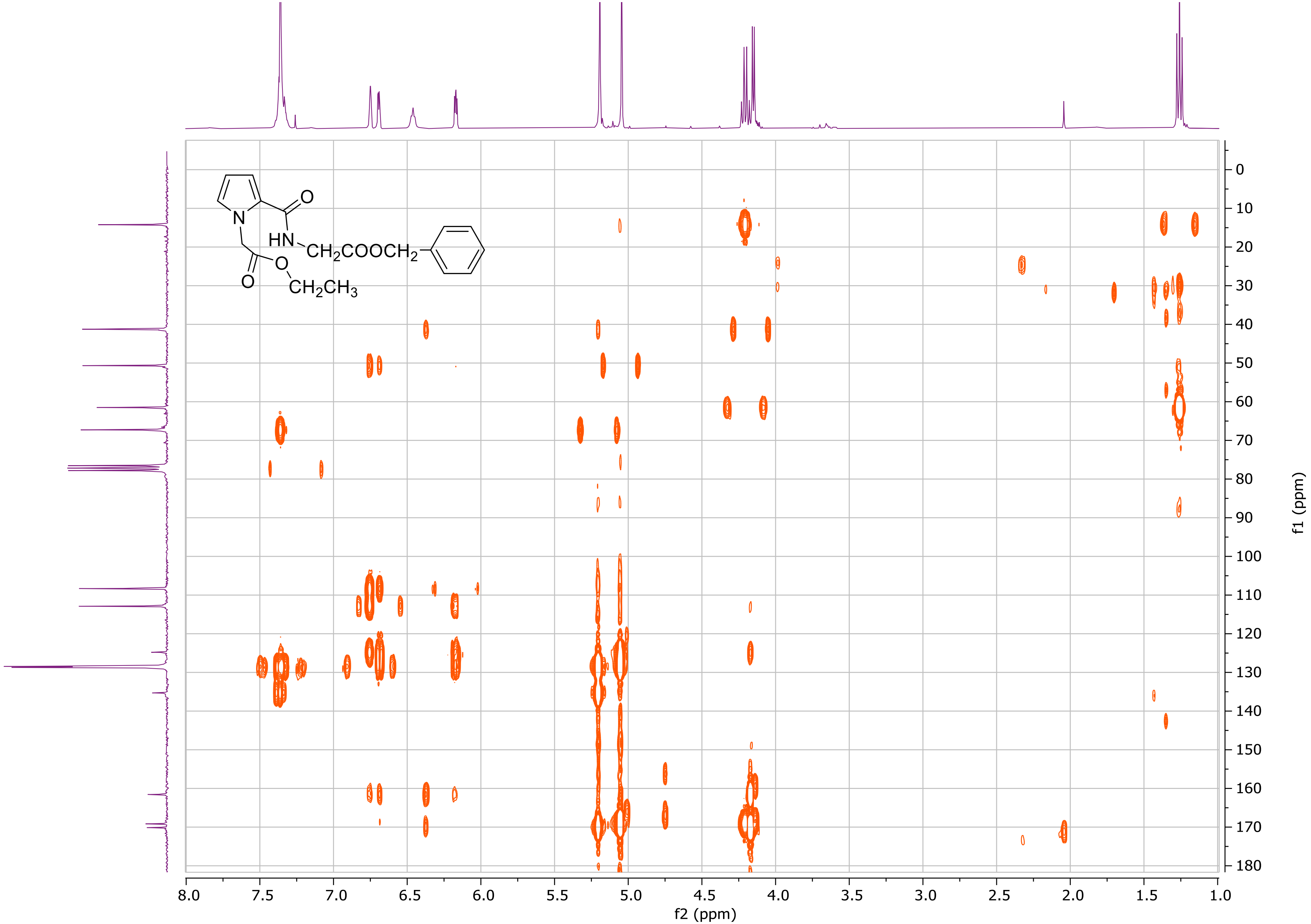

^1H NMR of **60** (600.11 MHz, $CDCl_3$)

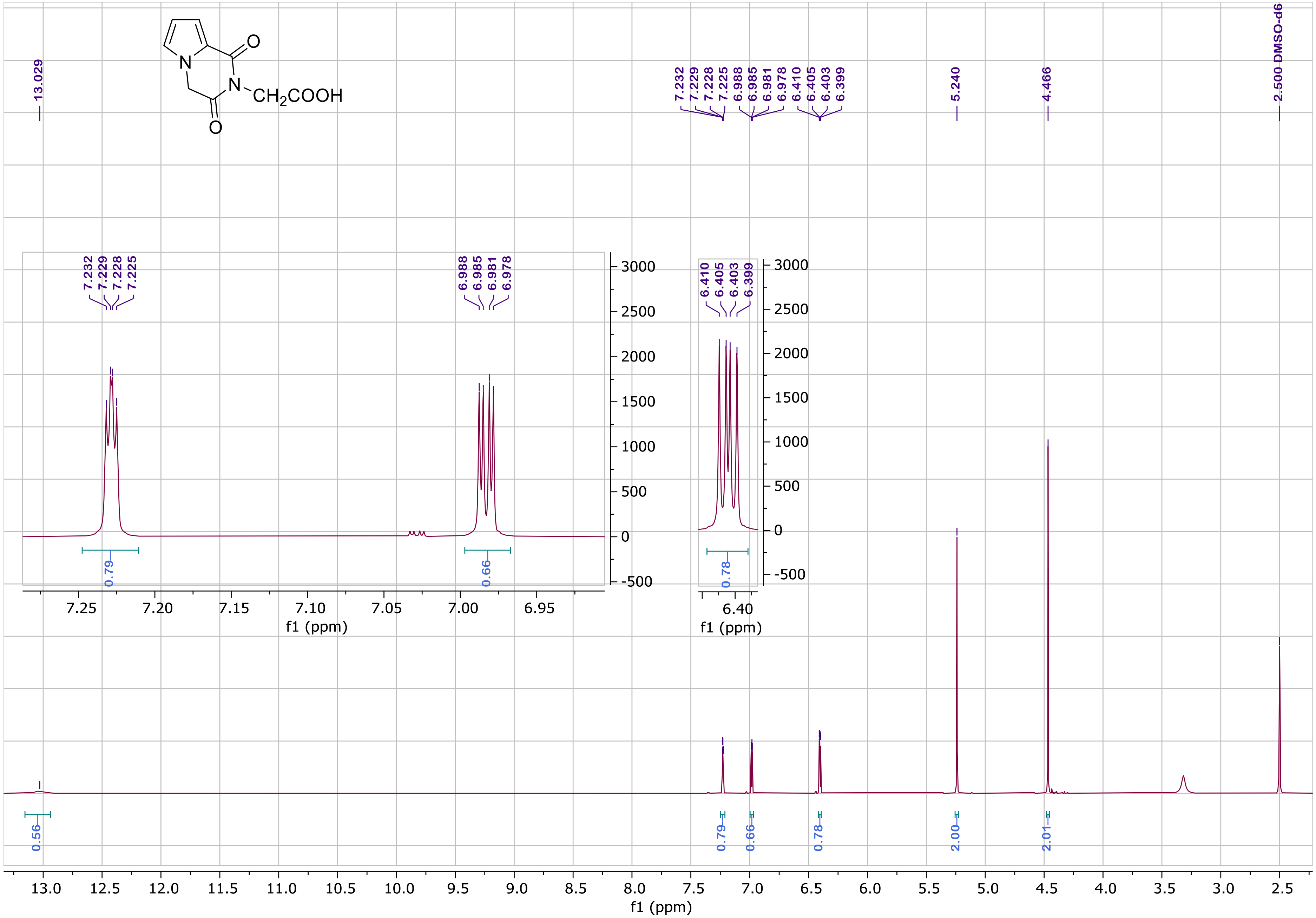

^{13}C NMR of **60** (50.32 MHz, $CDCl_3$)

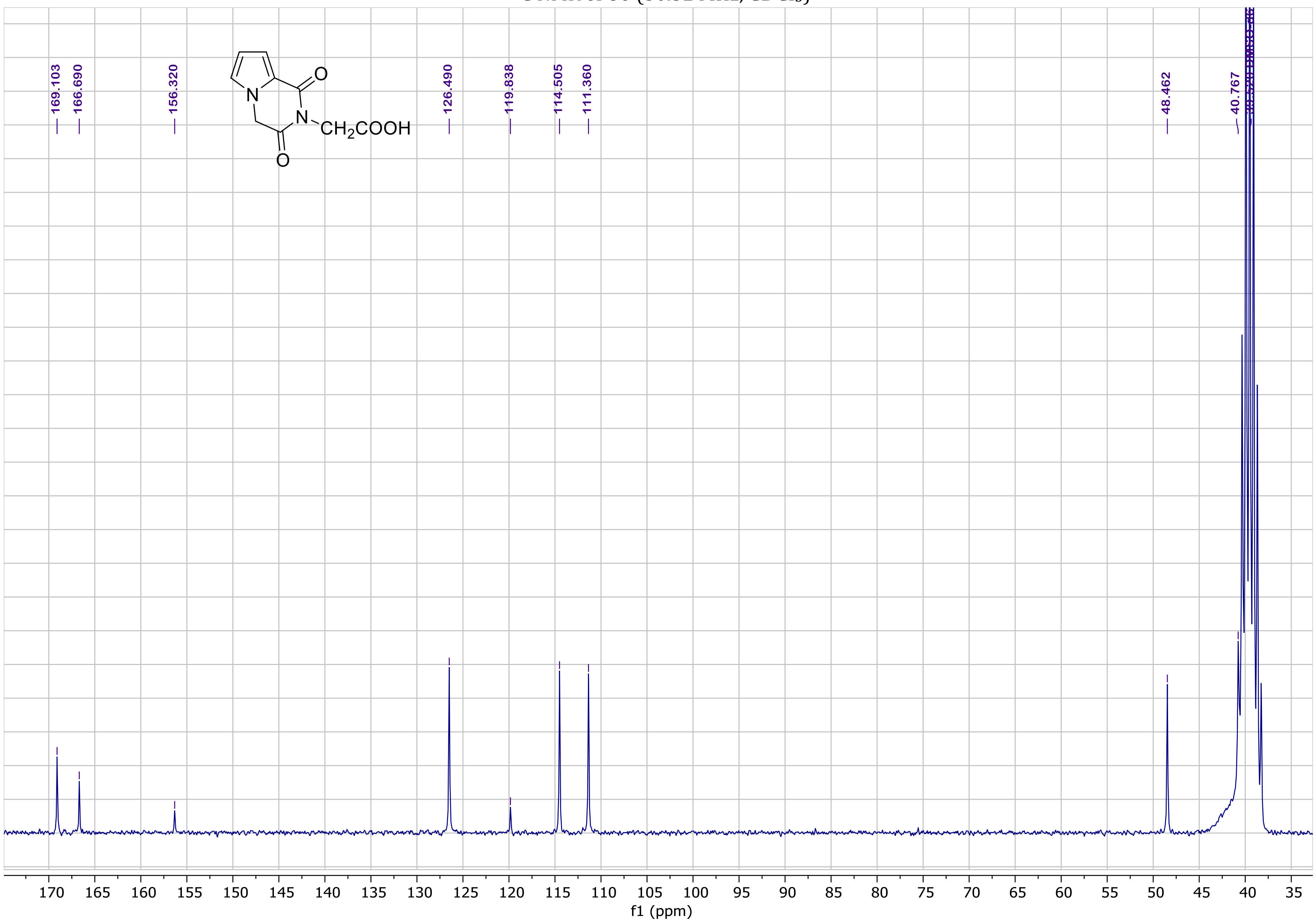

DEPT NMR of **60** (50.32 MHz, $CDCl_3$)

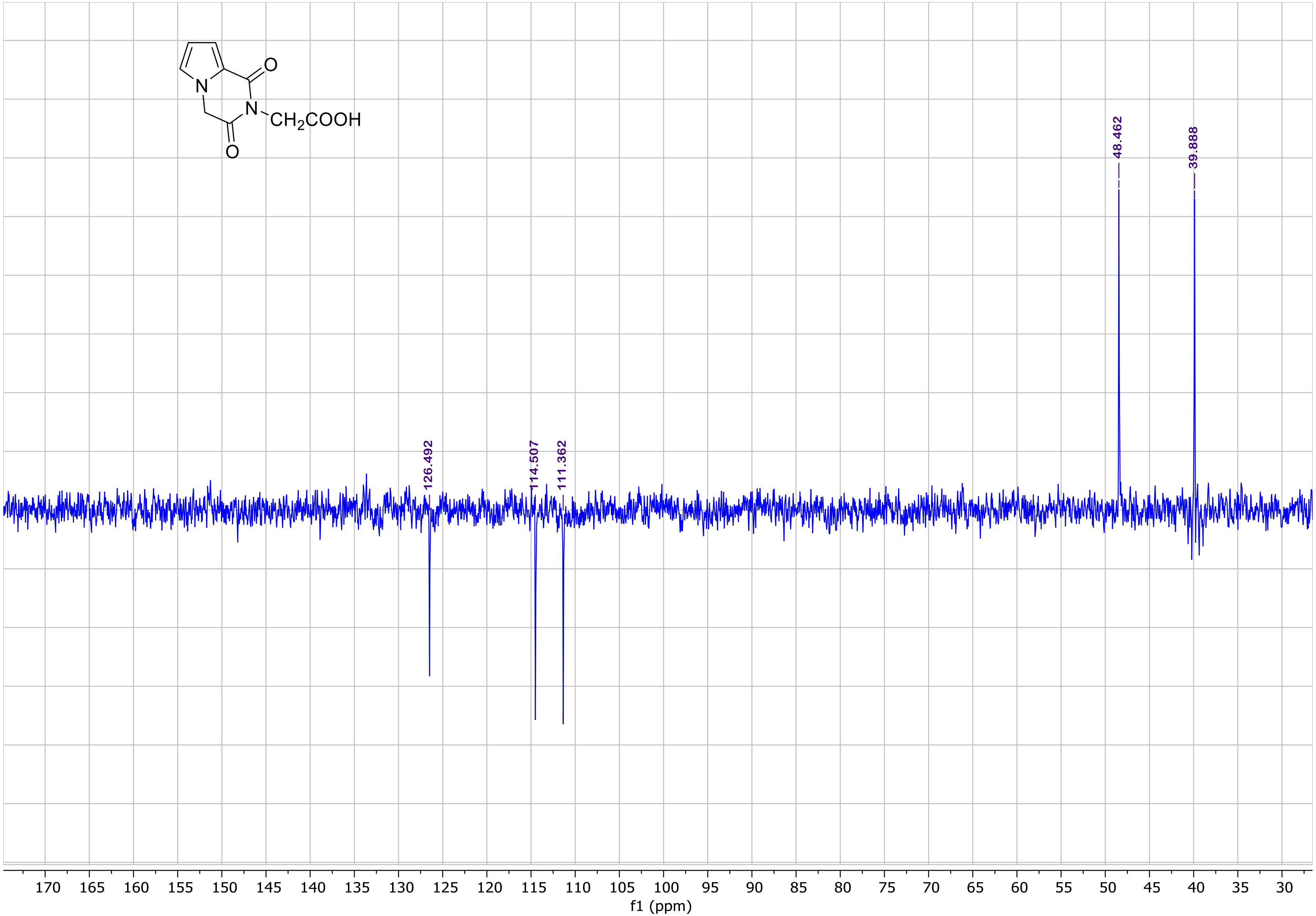

COSY NMR of **60** (600.11 MHz, $CDCl_3$)

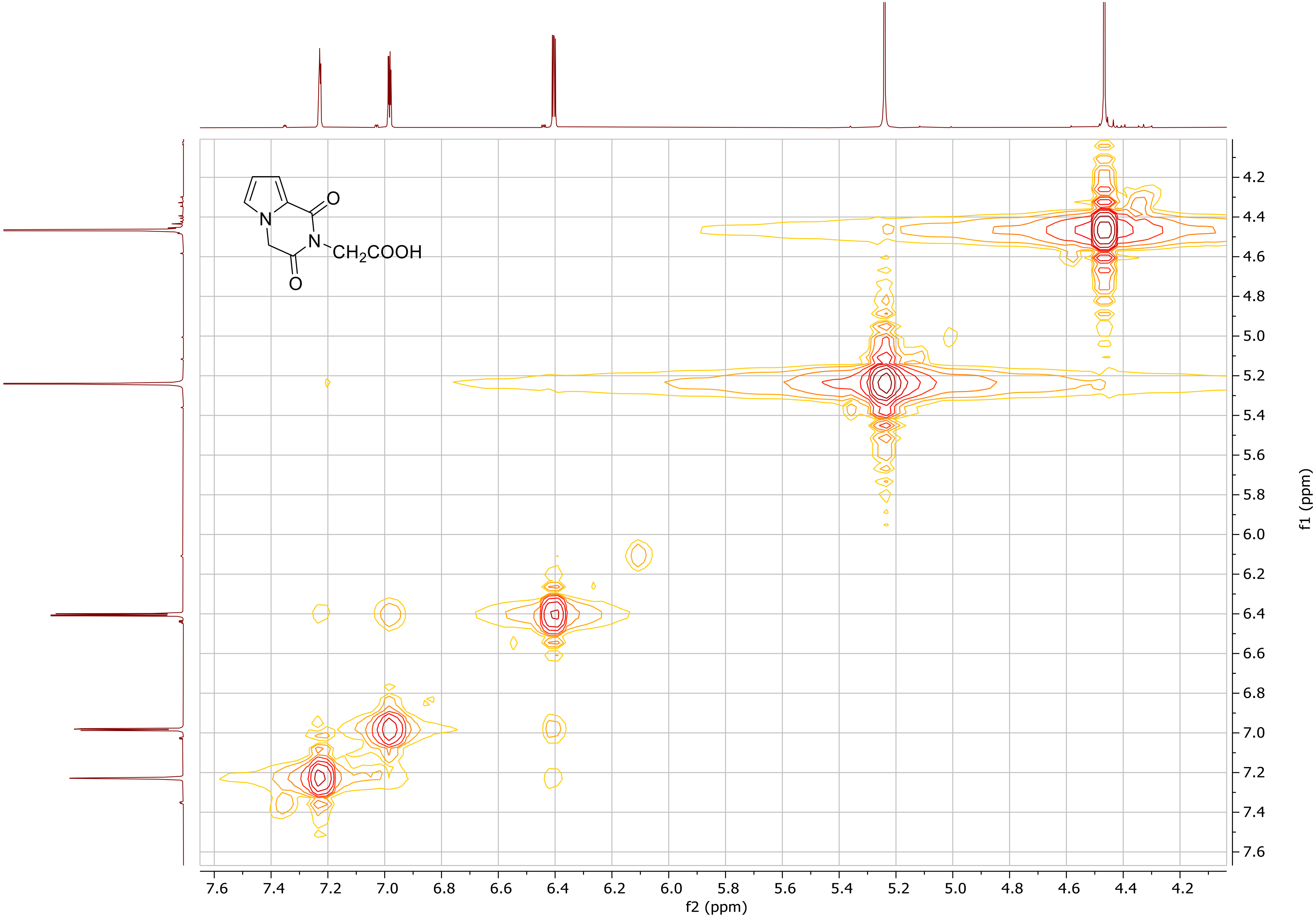

HSQC-DEPT NMR of **60** (600.11 MHz, $CDCl_3$)

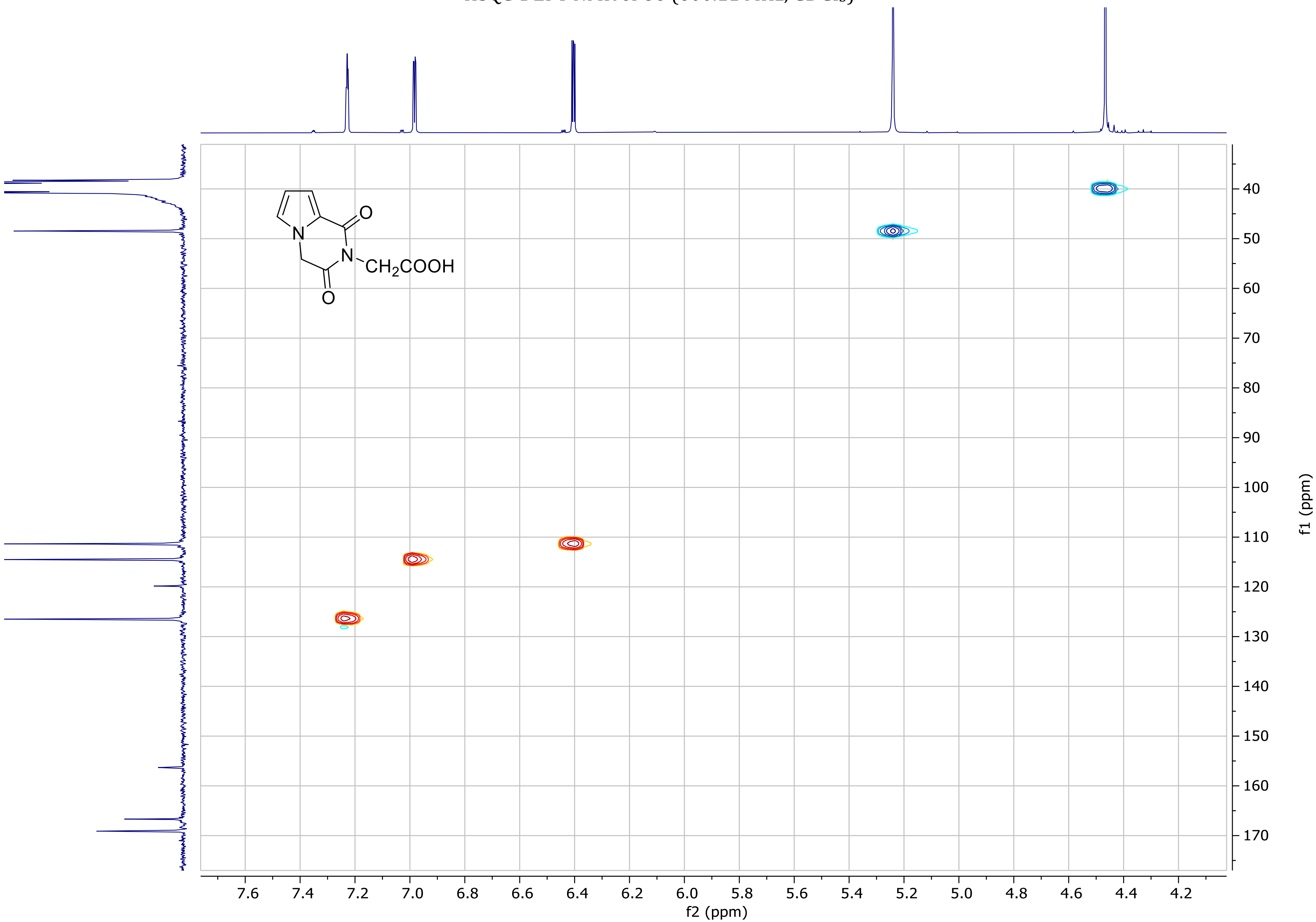

HMBC NMR of **60** (600.11 MHz, $CDCl_3$)

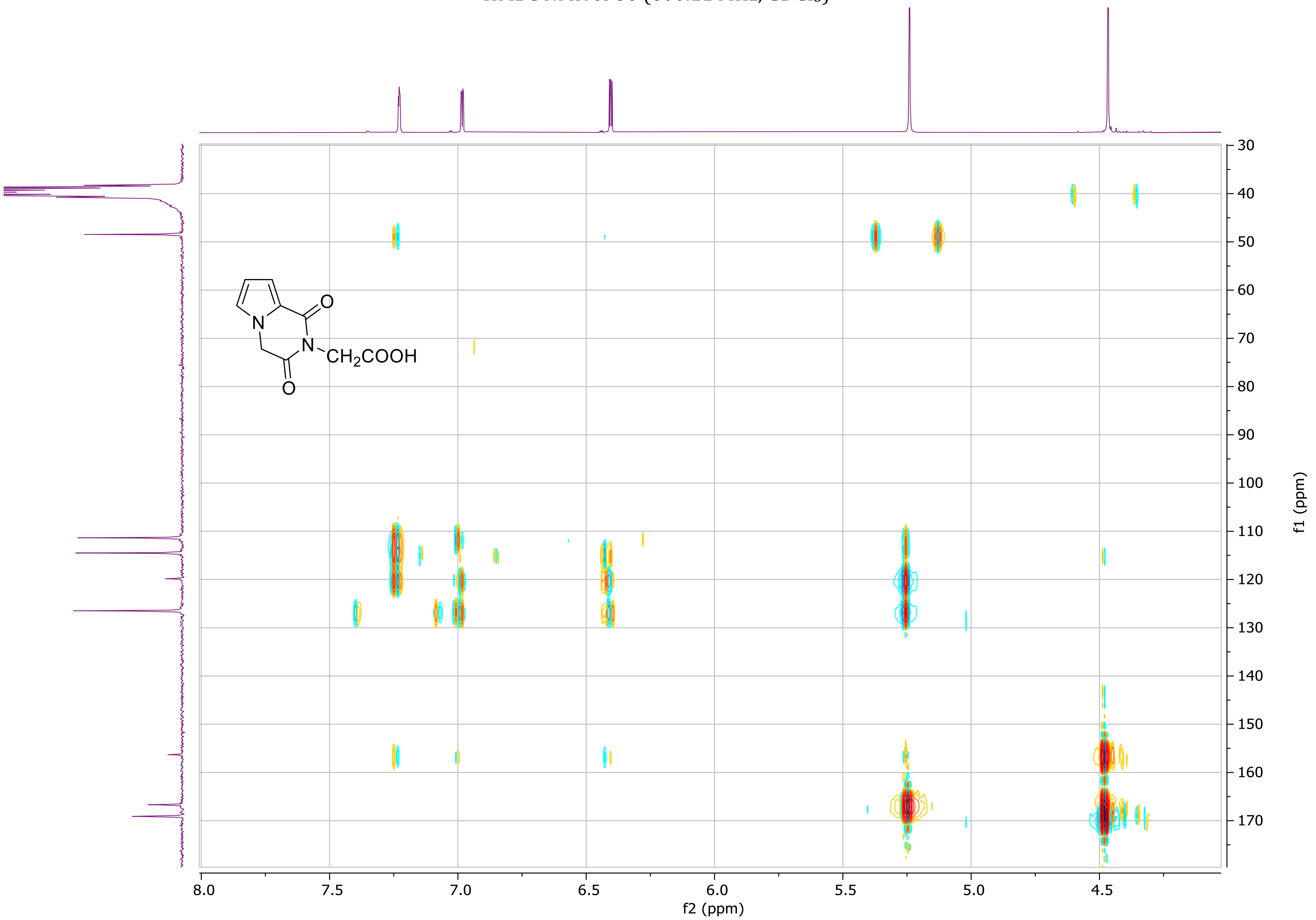

^{1}H NMR of **61** (600.11 MHz, Acetone-d_6)

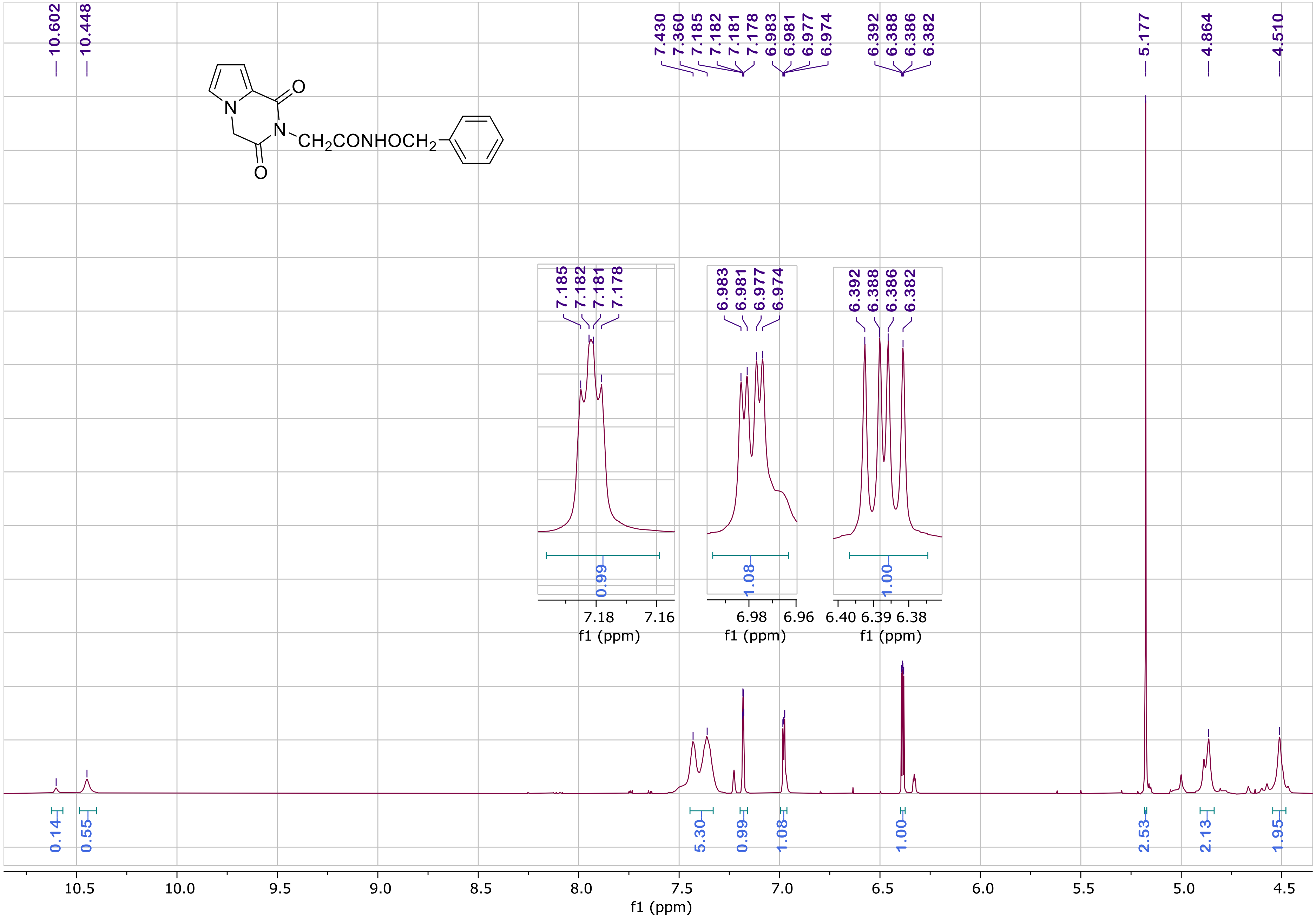

^{13}C NMR of **61** (50.32 MHz, Acetone-d_6)

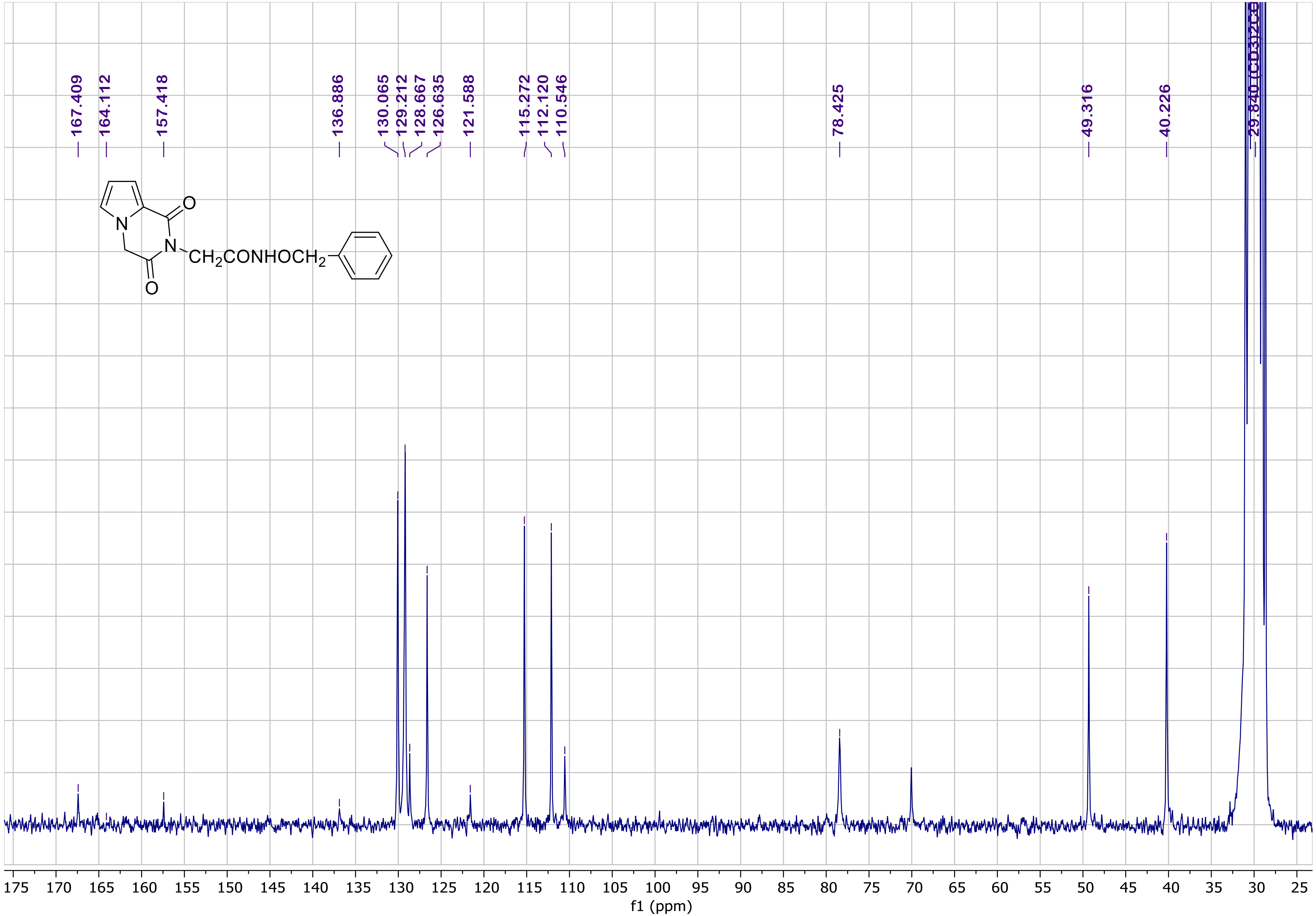

DEPT NMR of **61** (50.32 MHz, CD Acetone-d_6Cl$_3$)

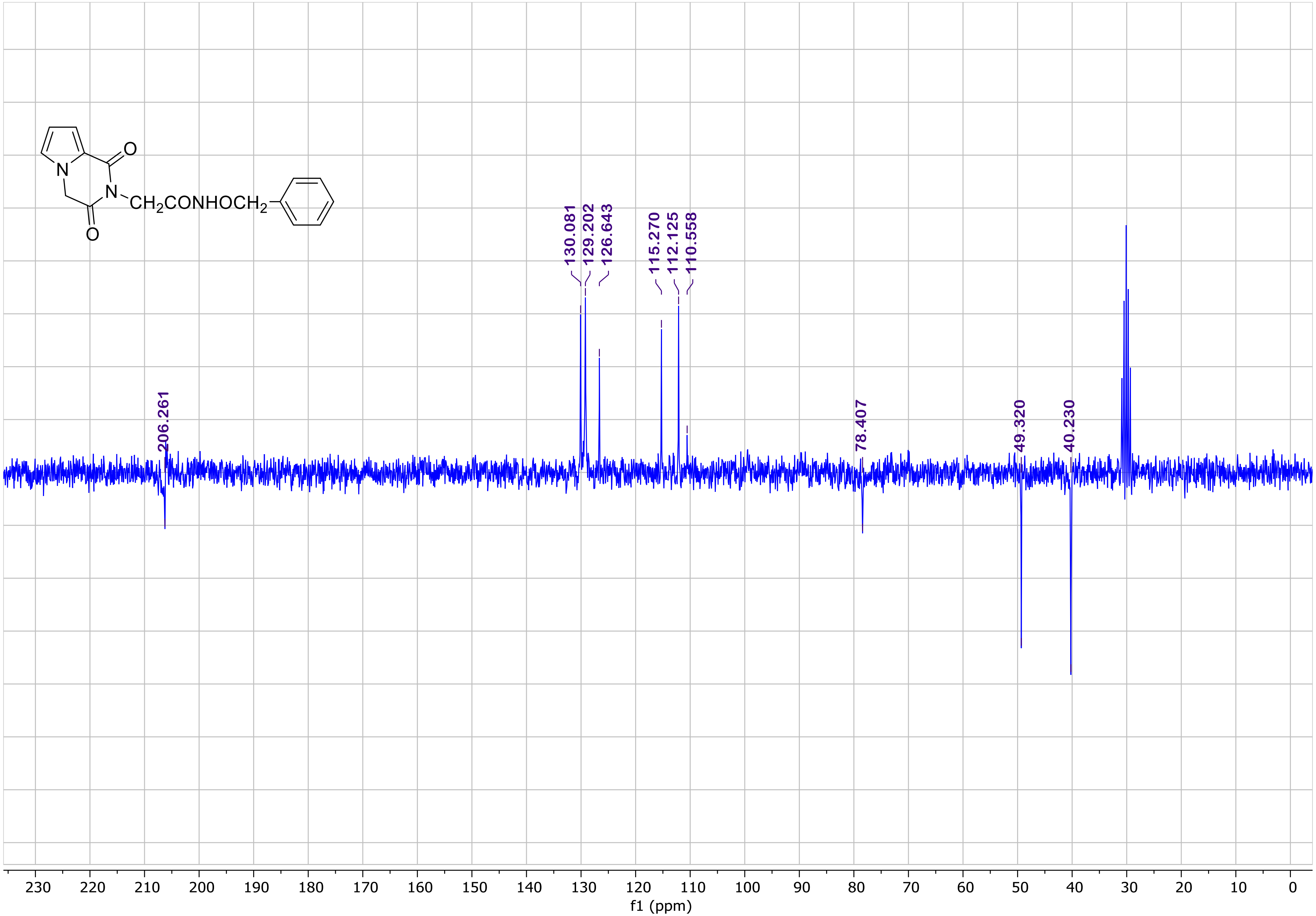

COSY NMR of **61** (600.11 MHz, Acetone-d_6)

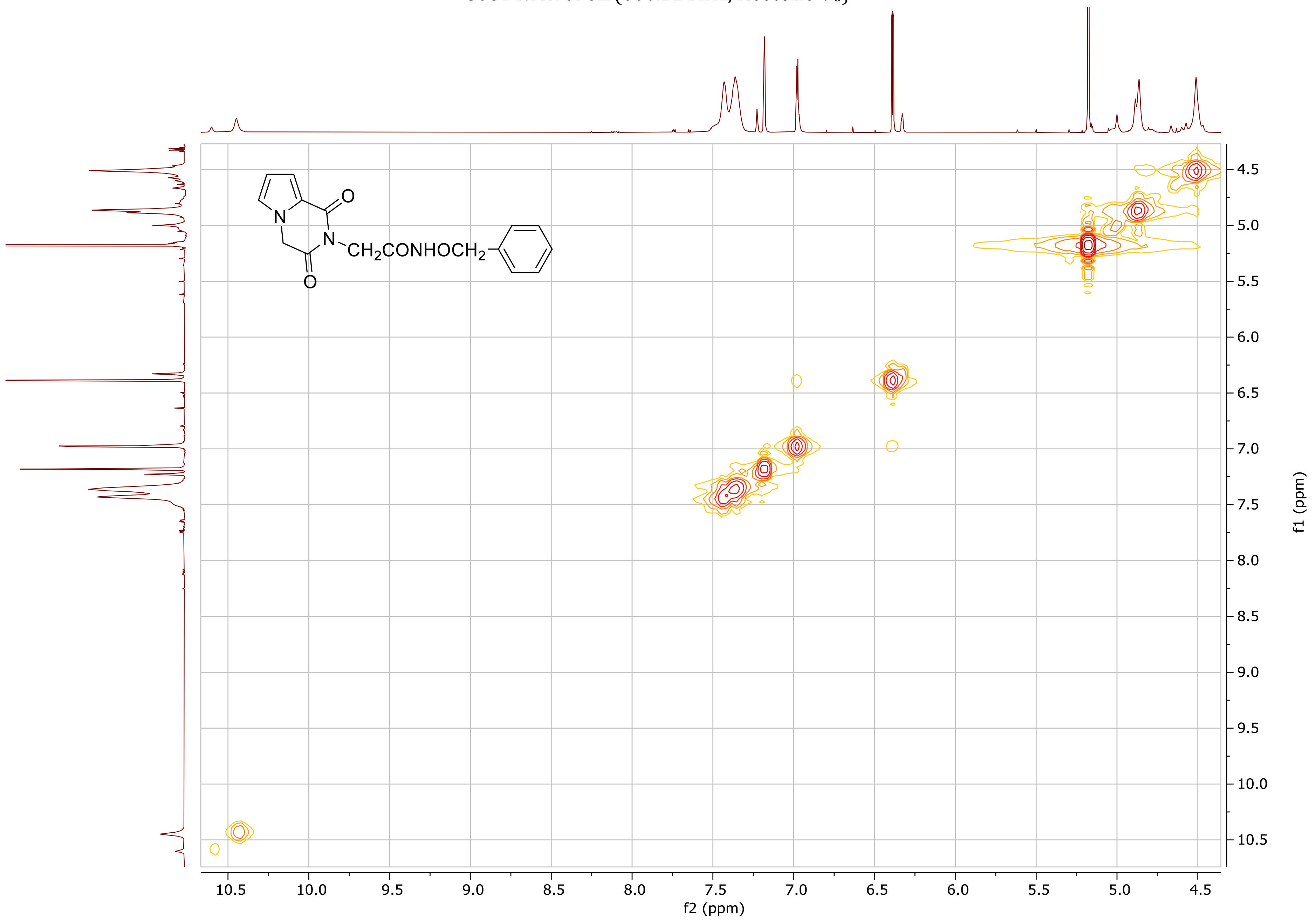

HSQC-DEPT NMR of **61** (600.11 MHz, Acetone-d_6)

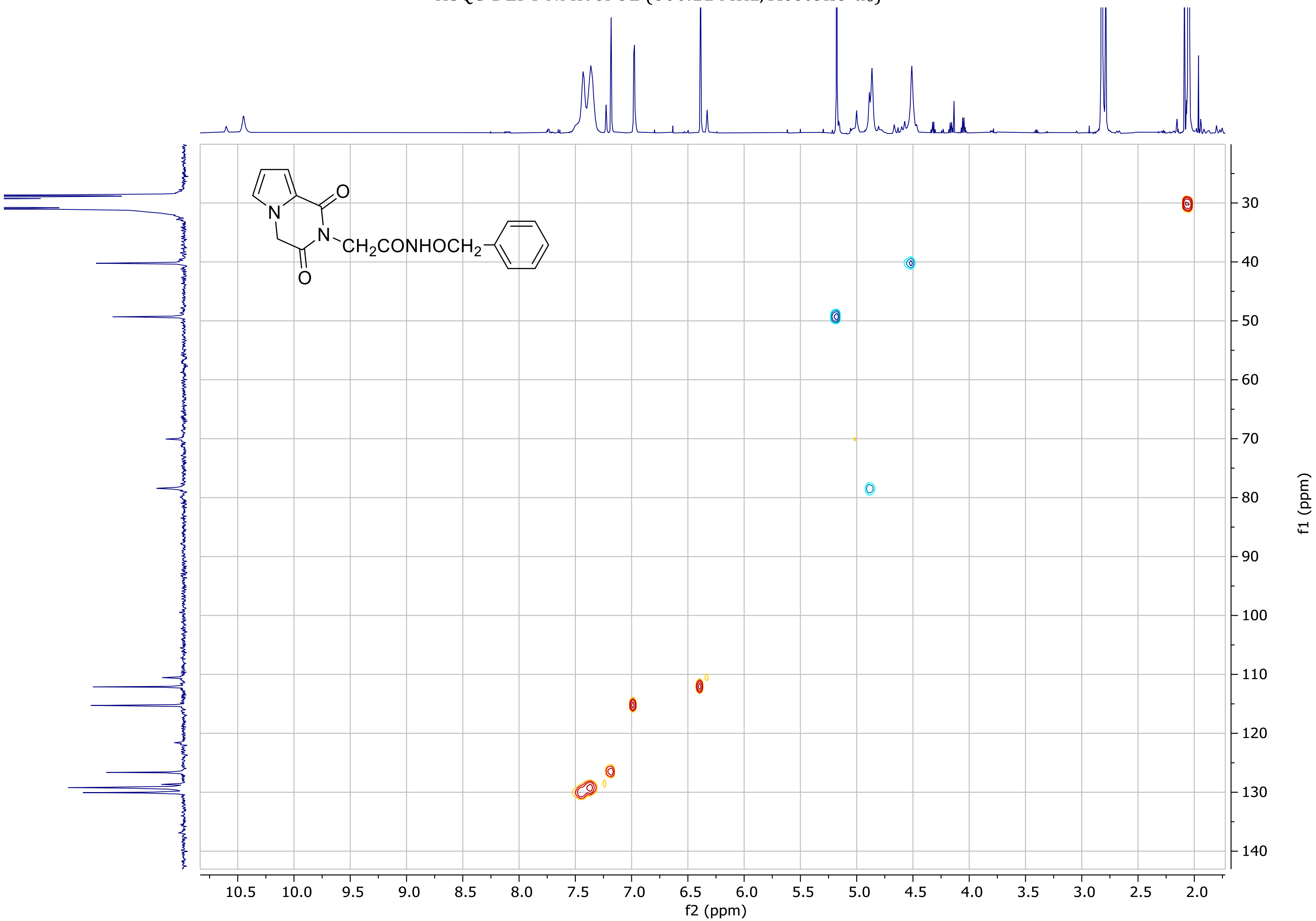

HMBC NMR of **61** (600.11 MHz, Acetone-d_6)

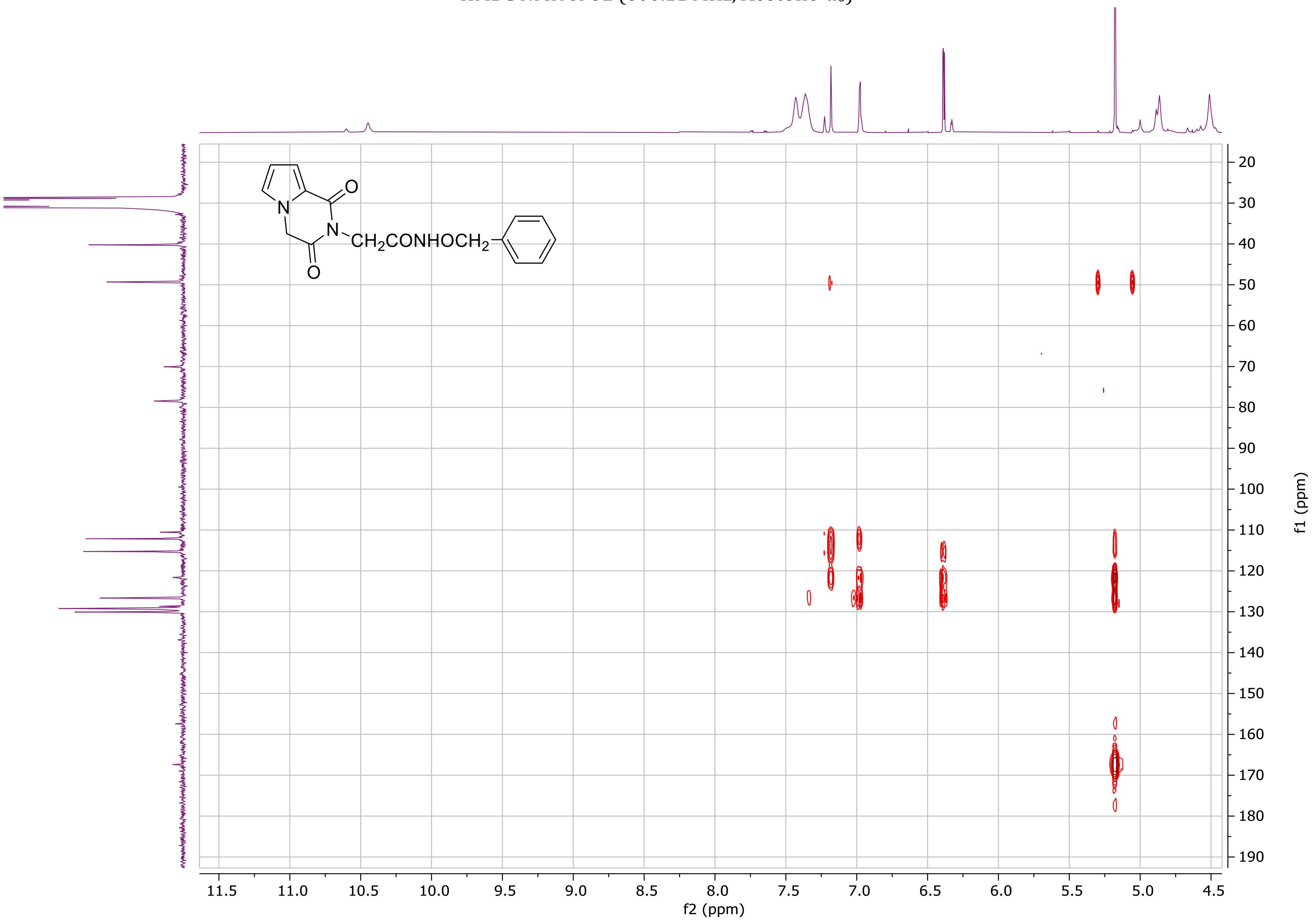

^{1}H NMR of **62** (600.11 MHz, DMSO-d_6)

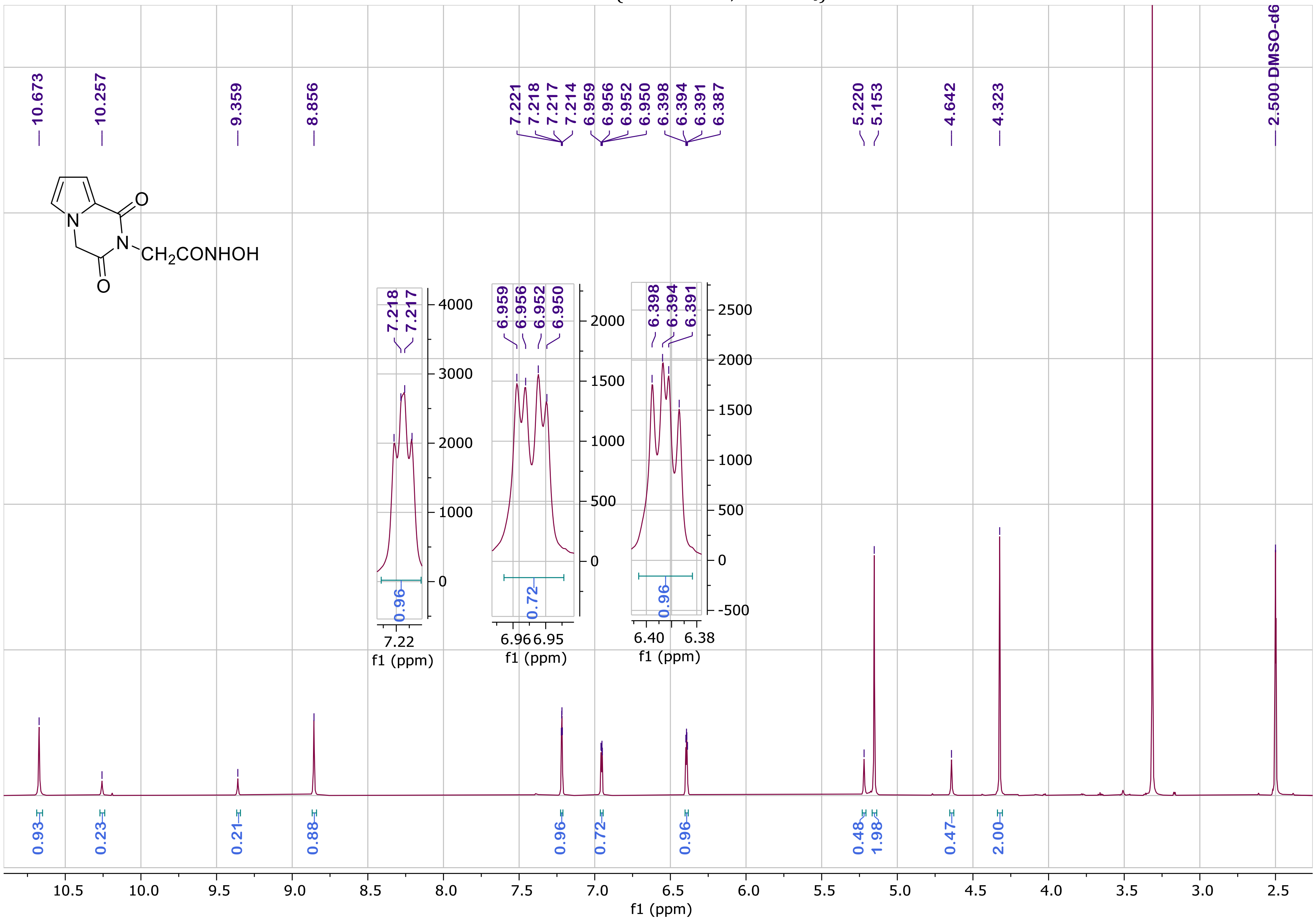

^{13}C NMR of **62** (150.9 MHz, DMSO-d_6)

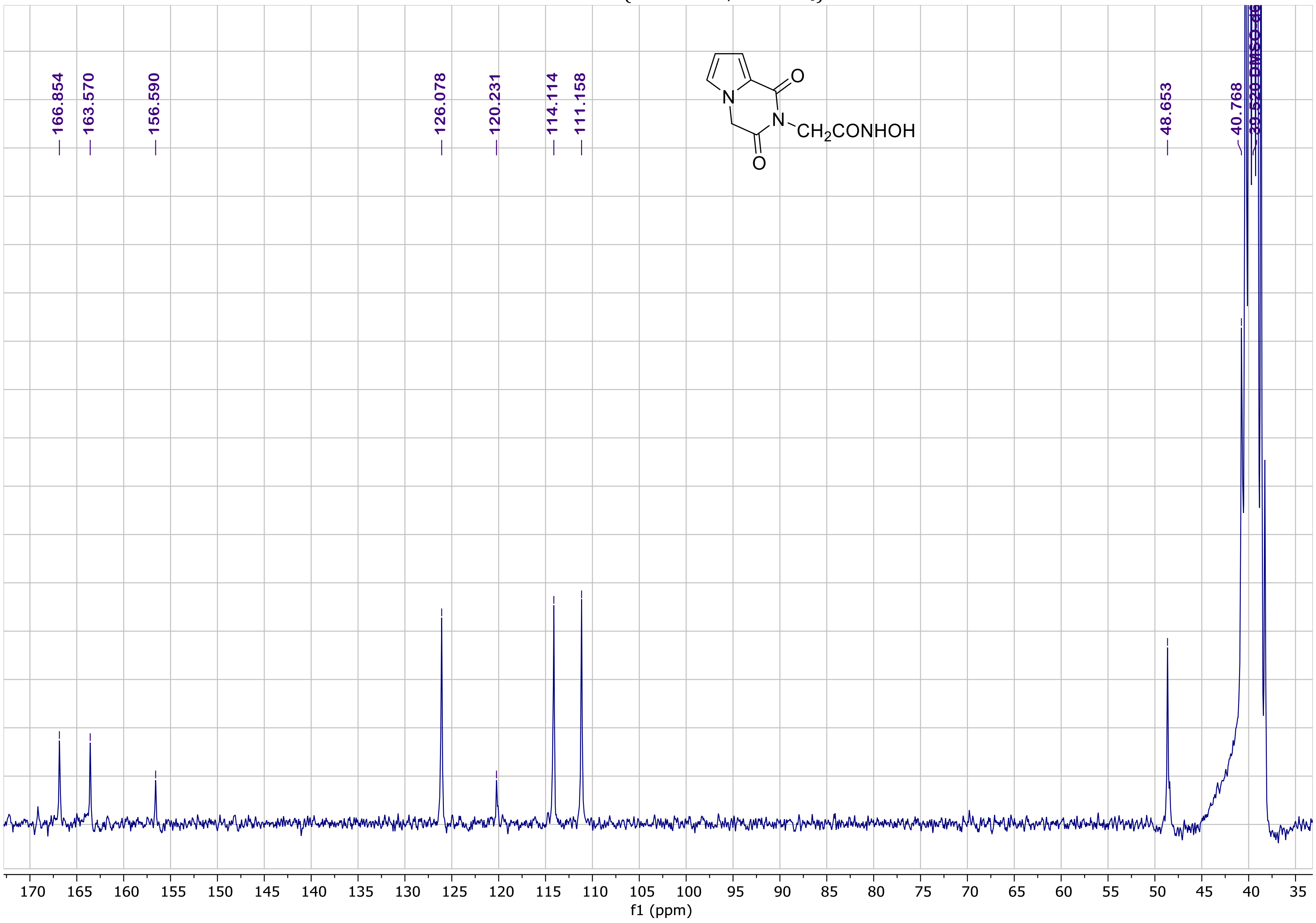

DEPT NMR of **61** (50.32 MHz, CD Acetone-d_6Cl_3)

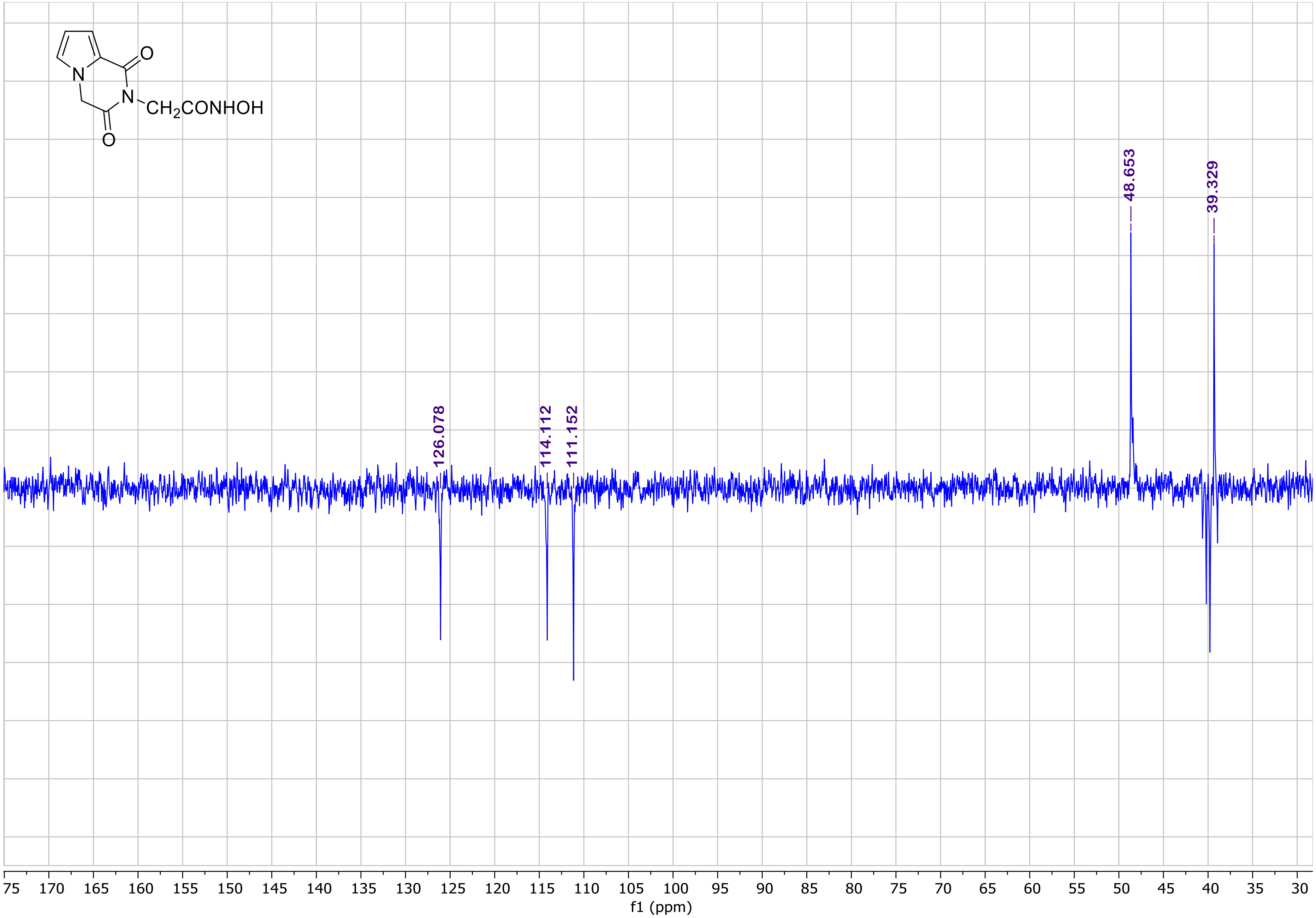

HSQC-DEPT NMR of **62** (600.11 MHz, DMSO-d_6)

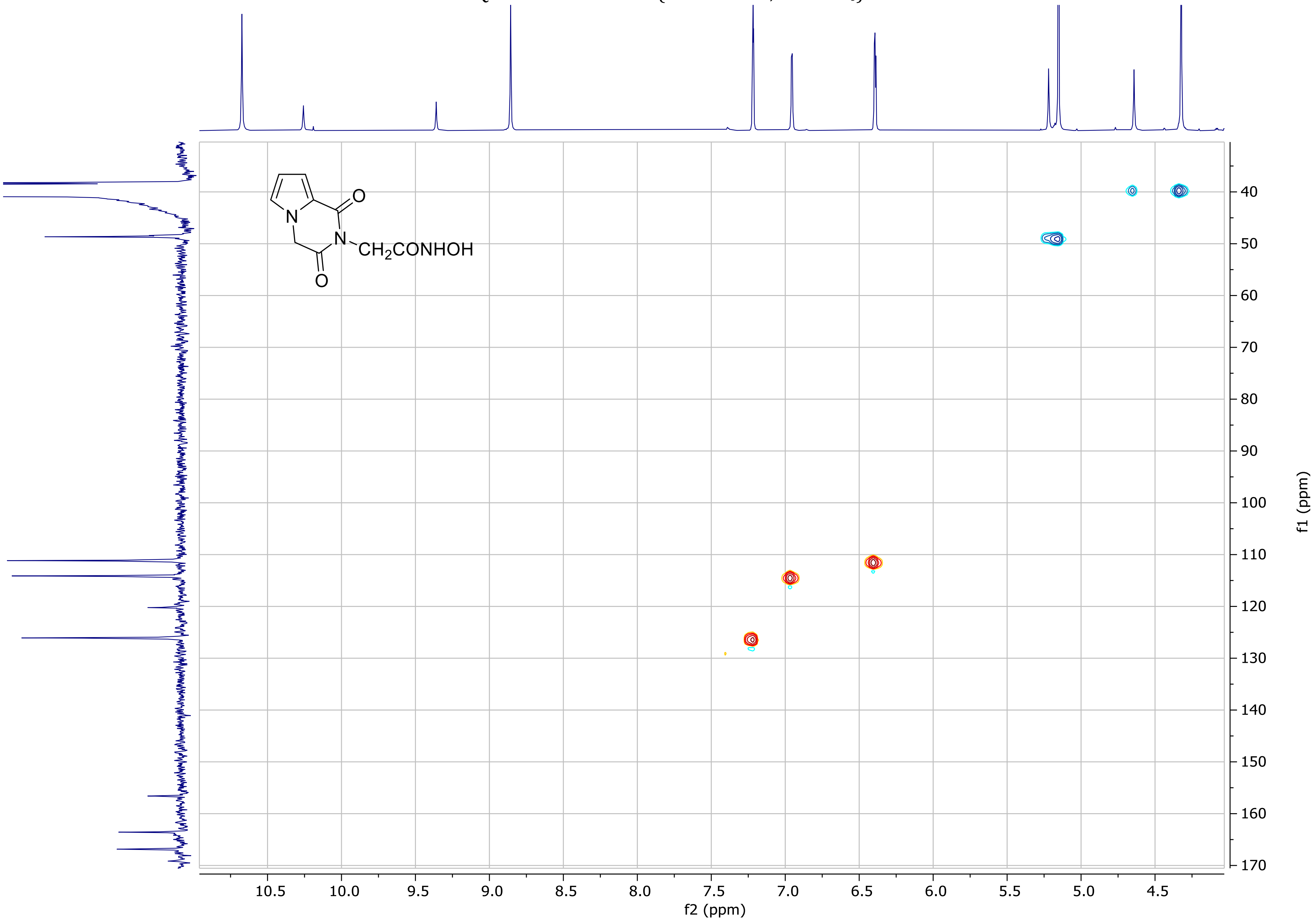

HMBC NMR of **62** (600.11 MHz, DMSO-d_6)

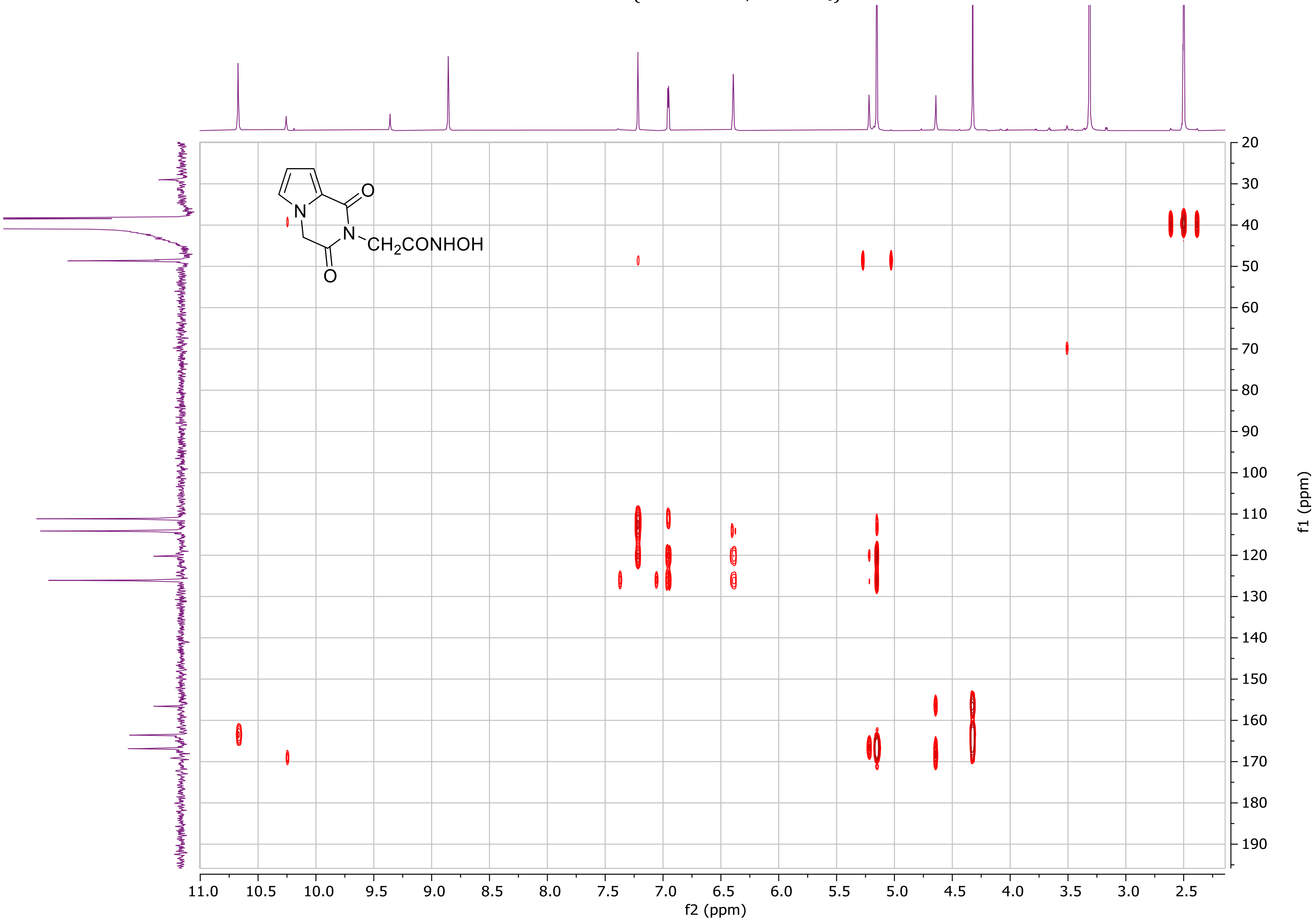

V. References

Bressanelli, S., Tomei, L., Rey, F.A., De Francesco, R., 2002. Structural Analysis of the Hepatitis C Virus RNA Polymerase in Complex with Ribonucleotides. Journal of Virology 76, 3482–3492. https://doi.org/10.1128/jvi.76.7.3482-3492.2002

Kempf, D.J., Condon, S.L., 1990. Synthesis of rigid, heterocyclic dipeptide analogs. J. Org. Chem. 55, 1390–1394. https://doi.org/10.1021/jo00291a060
